# A Study on *Gentiana dahurica* Fisch Ethanol Extract Alleviating Alcoholic Liver Disease in Mice: A Metabolomic Analysis of the Liver

**DOI:** 10.1155/2021/5569538

**Published:** 2021-06-29

**Authors:** Houkang Cao, Yanxiu Guo, Ling Jin

**Affiliations:** College of Pharmacy, Gansu University of Chinese Medicine, Lanzhou, Gansu 730000, China

## Abstract

We clarified the hepatoprotective effect of *Gentiana dahurica* Fisch ethanol extract (GDEE) in our previous study, and we further revealed the mechanism with the help of metabolomics technology in this study. The livers from Control group, Alcohol group, and Alcohol + GDEE group were analyzed by metabolomics. The metabolites in the liver were separated by ultra-high-performance liquid chromatography (UHPLC) and were tentatively identified using mass spectrometry (MS)/MS analysis. Differential metabolites were defined with VIP > 1 and *P* < 0.05. Principal component analysis (PCA) and orthogonal partial least square discriminant analysis (OPLS-DA) were applied to analyze differences among these groups. The results showed that the groups could be clearly distinguished by PCA and OPLS-DA analysis. Alcohol and GDEE could change the overall profile of liver metabolites. Alterations in liver tissues of ALD mice induced by alcohol were mainly involved in the dipeptides, purine and pyrimidine metabolism and glucose and lipid metabolism, which could be partly affected by GDEE. This study revealed that the mechanism of GDEE in alleviating ALD had the characteristics of multitarget and multipathway.

## 1. Introduction

With the increasing consumption of alcohol, the incidence of alcoholic liver disease (ALD) caused by long-term excessive drinking increases year by year. According to the pathological features, ALD is generally divided into alcoholic fatty liver, hepatitis, liver fibrosis, cirrhosis, and hepatocellular carcinoma [1]. Currently, it is believed that alcoholic fatty liver is in the early stage of ALD, which is the key stage in the inhibition and reversal of ALD [2]. The clinical treatment of advanced ALD is based on abstinence, supplemented by nutritional support and drug therapy [3]. Unfortunately, there are currently no approved clinical drugs for the treatment of ALD. Therefore, it is necessary to develop safe and effective new drugs to maintain public health.

It has become a research hotspot to obtain new drugs for the prevention and treatment of ALD from natural plants, and many studies have demonstrated the safety and effectiveness of natural plant extracts in alleviating ALD [4]. Our previous study was inspired by the “compendium of materia medica” and clarified the effect of GDEE in alleviating ALD [5]. In order to further clarify the mechanism of GDEE, this study applied metabolomics technology to reveal the mechanism of GDEE from the metabolite level.

## 2. Materials and Methods

### 2.1. A Brief Introduction of the Previous Study

In our previous study, the results showed that GDEE could inhibit alcohol-induced ALD in mice [5]. The mice of Control group were given water by gavage for 14 days; the mice of Alcohol group were given 56% alcohol by gavage for 14 days; the mice of Alcohol + GDEE group were given 56% alcohol plus GDEE by gavage for 14 days. In this study, the liver tissue samples were selected from Control group, Alcohol group, and Alcohol + GDEE group (4 samples for each group).

### 2.2. Sample (Quality Control Sample) Preparation

A small amount of liver tissue (25 mg) was put into 500 *μ*L extract solution (methanol : acetonitrile : water = 2 : 2 : 1, *V*/*V*; methanol: LC-MS grade, 67-56-1; acetonitrile: LC-MS grade, 75-05-8, CNW Technologies GmbH, Düsseldorf, Germany). Then, the samples were mixed for 30 s, homogenized at 35 Hz for 4 min in a grinder (JXFSTPRP-24, Shanghai Jingxin Industrial Development Co., Ltd, Shanghai, China), and then sonicated in an ice water bath (YM-080S, Shenzhen Fangao Microelectronics Co., Ltd., Shenzhen, China) for 5 min. The above process was repeated 3 times. After the sample was placed at −40 C for 1 h, it was centrifuged at 12,000 rpm for 15 min at 4 C (Heraeus Fresco 17, Thermo Fisher Scientific (China) Co., Shanghai, China). Subsequently, 400 *μ*L of supernatant was transferred into an EP tube and dried by the vacuum. Next, the dried samples were redissolved with 200 *μ*L of 50% acetonitrile, vortexed for 30 s, and sonicated for 10 min in an ice water bath. The samples were then centrifuged at 13,000 rpm for 15 min at 4 C. Finally, 10 *μ*L of the supernatant was taken from all samples and mixed into a quality control sample. After the quality control is passed, 75 *μ*L of the supernatant from each sample is transferred to the sample bottle and used for ultra-high-performance liquid chromatography (UHPLC)-mass spectrometry (MS)/MS.

### 2.3. UHPLC Analysis

The metabolites of the samples were separated by the UHPLC system (Agilent 1290, Agilent Technologies, Inc., Santa Clara, USA) equipped with a column (2.1 mm × 100 mm, 1.7 *μ*m; Acquity UPLC BEH Amide, Waters Corp, Milford, USA). The mobile phase was composed of A (water containing 25 mmol/L ammonium acetate and 25 mmol/L ammonia hydroxide) and B (acetonitrile). The gradient was as follows: 0 to 0.5 min, 95% B; 0.5 to 7 min, 95%∼65% B; 7 to 8 min, 65%∼40% B; 8 to 9 min, 40% B; 9 to 9.1 min, 40%∼95% B; 9.1 to 12 min, 95% B. Column temperature was 25 C; flow rate was 0.5 Ml/min; and injection volume was 1 *μ*L.

### 2.4. MS/MS Analysis

The metabolite structure was identified using high-resolution MS (Triple TOF 6600, AB Sciex, Boston, USA) combined with data analysis software (Analyst TF 1.7, AB Sciex). MS conditions were as follows: collision energy as 30 eV, cycle time as 0.56 s; secondary scanning ranges as 60 to 1200 m/z; electrospray ion source (ESI) including gas 1 as 60 psi, gas 2 as 60 psi, curtain gas as 35 psi, source temperature as 600 C, declustering potential as 60 V, and ion spray voltage floating as 5000 V or −4000 V in positive or negative modes, respectively.

### 2.5. Data Analysis and Differential Metabolite Screening

MS raw data (wiff) files were converted to the mzXML format by ProteoWizard and processed by *R* package XCMS (version 3.2). The process includes peak deconvolution, alignment, and integration. Minfrac and cutoff values were set as 0.5 and 0.3, respectively. Combined with the molecular weight and MS/MS spectra, the structures of metabolites were tentatively identified by matching with the secondary fragment ion information in the self-built database (Biomarker Biotechnology Co., Beijing, China). The metabolite content of this project was normalized and relatively quantified by peak area, that is, the peak area of each metabolite was divided by the peak area of internal standard. SIMCA 14.1 software was used for principal component analysis (PCA) and orthogonal partial least squares discrimination analysis (OPLS-DA). The variable importance in projection (VIP) > 1 obtained by OPLS-DA analysis and *P* values (*P* < 0.05) obtained by one-way ANOVA were used as the screening criteria for differential metabolites [6]. KEGG pathway enrichment analysis was performed by *R* package clusterProfiler.

## 3. Results

### 3.1. Quality Control Results

**Figure 1:**
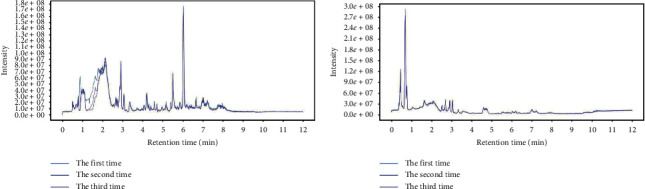
The total ion chromatogram of the quality control sample repeated 3 times. (a) Positive mode and (b) negative mode.

The total ion chromatogram of the quality control sample (Figure 1) showed that the retention time and peak area of quality control sample were well overlapped after repeated analysis for 3 times (Figure 1). The three samples could not be clearly separated by PCA analysis (Supplementary information 1). In addition, Pearson's correlation analysis showed that the correlation coefficients of the three samples were over 0.98 (Supplementary information 1). Therefore, the stability of the instrument and experimental conditions could basically ensure the reliability of subsequent experiments.

### 3.2. Results of PCA Score Plots

**Figure 2:**
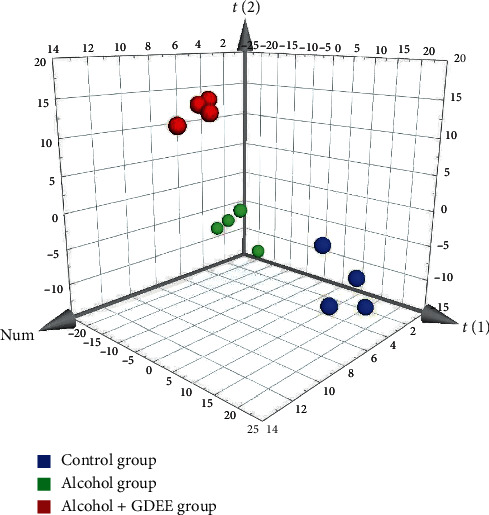
3D-PCA score plots for separation of three different groups.

The PCA score plots (Figure 2) presented that these three groups were separated clearly. No matter from the direction of the *t*[1] axis or the *t*[2] axis, the samples within the group were relatively close, while the samples between the groups were obviously separated. Moreover, the samples of Control group were distributed between the samples of Alcohol group and the samples of Alcohol + GDEE group. The unsupervised PCA of this study provided us with a preliminary conclusion, which indicated that there were some differences between the three groups, and it was worth further analysis.

### 3.3. Results of OPLS-DA Score Plots

**Figure 3:**
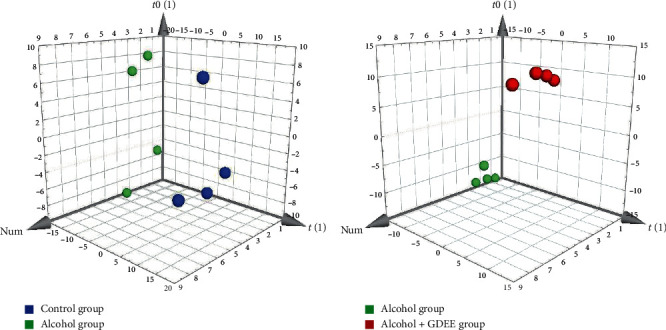
OPLS-DA score plots of (a) the Control group vs the Alcohol group or (b) the Alcohol group vs the Alcohol + GDEE group.

OPLS-DA, a supervised method, was used for the difference analysis between two groups. Regardless of the comparison between the Control group and the Alcohol group or between the Alcohol group and the Alcohol + GDEE group (Figure 3), the samples were well separated. This result further showed that there were significant differences in metabolites between different groups. The specific metabolites that caused the differences between the groups can be dug out by OPLS-DA, and these metabolites are assigned corresponding VIP values, which can be used as one of the screening conditions for differential metabolites [7]. In addition, the separation of Control group versus Alcohol group was clearer than that of Alcohol group versus Alcohol + GDEE group on the first principal component axis (*t*[1]).

### 3.4. Overview of Differentially Expressed Metabolites

**Figure 4:**
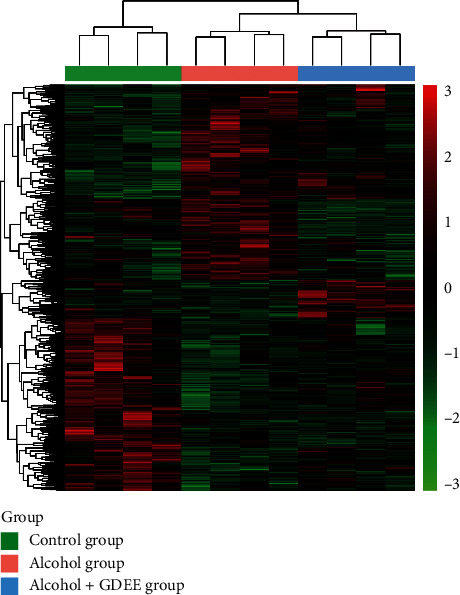
Heat map overviews of differentially expressed metabolites.

There were 2568 and 2545 metabolites in positive mode and negative mode, respectively, but only a few metabolites were identified. Compared with the Control group, 270 metabolites were downregulated and 255 metabolites were upregulated in the Alcohol group. Compared with the Alcohol group, 76 metabolites were downregulated and 148 metabolites were upregulated in the Alcohol + GDEE group. The systematic clustering results of these differential metabolites showed that the clustering results of the 12 samples were consistent with the grouping (Figure 4).

### 3.5. Correlation Analysis of Dipeptides

**Figure 5:**
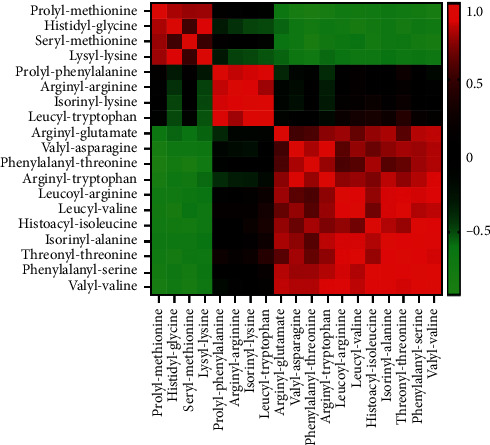
Pearson's correlation analysis of dipeptides.

There were many dipeptides with obvious changes, but they were not annotated by KEGG or other databases. Pearson's correlation analysis was performed to further reveal the relationship between these dipeptides. Correlation analysis showed that there were significant positive correlations or negative correlations between some dipeptides, as shown in Figure 5. The correlation coefficient between some dipeptides could be higher than 0.9 or lower than −0.9.

### 3.6. Results of Metabolic Pathway Analysis

**Figure 6:**
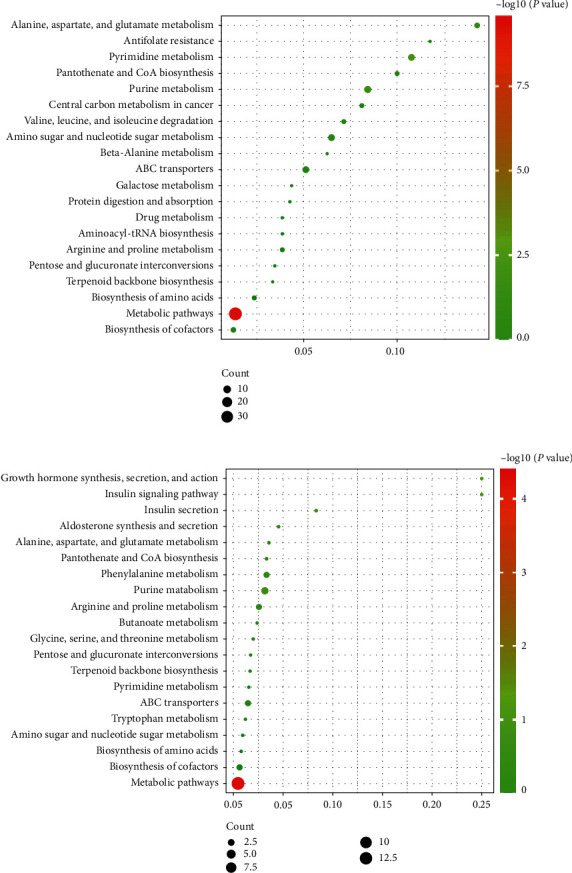
Results of metabolic pathway analysis. (a) The Control group vs the Alcohol group and (b) the Alcohol group vs the Alcohol + GDEE group. The size and color of each circle represented number of metabolites and *P* value, respectively; *X*-axis and *Y*-axis represent pathway enrichment factor and pathway name, respectively.

Compared with the Control group, the changed pathways in the Alcohol group included many different metabolic pathways, involving amino acids metabolism, purine and pyrimidine metabolism, and glucose and lipid metabolism (Figure 6(a)). Next, it was observed that most of the enrichment pathways in Alcohol group versus Alcohol + GDEE group were the same as those in Control group versus Alcohol group (Figure 6(b)). However, GDEE intervention caused some changes different from alcohol, such as phenylalanine metabolism and glycine, serine, and threonine metabolism.

### 3.7. Information on Some Differential Metabolites

The classification and signaling pathways of some different metabolites are presented in Table 1. These metabolites included nucleic acids, amino acids, monosaccharides and other classifications. Each metabolite participated in multiple signaling pathways mentioned in Figure 6. In addition, there were some common metabolites in different signaling pathways. Although many of the metabolite changes induced by alcohol could not be reversed by GDEE, GDEE had an effect on other metabolites in the same pathway. See “Supplementary information 2” for the content of all metabolites.

## 4. Discussion

ALD is a typical disease with disorders of substance and energy metabolism. ALD, especially advanced ALD, is often accompanied by obvious metabolic disorders of carbohydrate, lipid, and protein [8]. This study found that alcohol caused changes in the contents of many metabolites in the liver tissues of mice (Figure 4). In addition, GDEE also caused changes in the contents of many metabolites in ALD mice.

Although malnutrition is no longer considered to be the main cause of ALD, it does aggravate ALD [9]. Severe ALD patients are generally accompanied by significant malnutrition, so nutritional support (protein or amino acid supplementation, etc.) has been an important part of the standard treatment of ALD [10]. There are many different dipeptides in this study, and correlation analysis results showed that some dipeptides were highly correlated with other metabolites (Figure 5). Therefore, it was speculated that these dipeptides may be derived from the same metabolic pathways of endogenous or exogenous proteins [11]. In addition, KEGG pathway analysis showed that alcohol or GDEE could regulate the synthesis and degradation pathways of amino acids or proteins (Figure 6). In short, alcohol did cause dipeptide changes in mouse liver tissue, which may be a precursor to malnutrition. GDEE could partially reverse the changes of these dipeptides.

Purine and pyrimidine metabolism plays an important role in physiological activity, and they are the basic units for the synthesis of RNA and DNA [12, 13]. In this study, the purine metabolism and pyrimidine metabolism were disturbed under the influence of alcohol (Figure 6), which was consistent with the results of other study [14]. In this study, alcohol upregulated the expressions of UDP, AMP, CMP, and DGMP in purine and pyrimidine metabolism and down-regulated the expressions of uracil, thymidine, and dUMP (Table 1). Although GDEE had no obvious effect on these metabolites, it regulated the expression of 2′-deoxyuridine, cAMP, and adenylosuccinic acid. Although GDEE had no direct effect on alcohol-induced differential metabolites, they might affect the same metabolic process in the complex network of interactions. For example, alcohol and GDEE upregulated the contents of AMP and adenylosuccinic acid, respectively, and both were involved in the adenine nucleotide cycle (AMP⟶IMP⟶adenylosuccinic acid⟶AMP) [15]. Therefore, GDEE might regulate the purine and pyrimidine metabolism disorders caused by alcohol in an indirect way.

Carbohydrates and lipids are the main substances for energy supply in the body, and their metabolic disorder is the main factor leading to lipid accumulation in the liver [16]. Our previous study confirmed that the most obvious characteristic of mouse liver with ALD was steatosis, which could be reversed by GDEE [5]. In this study, glucose 6-phosphate, the center of multiple glucose metabolic pathways [17], was significantly upregulated by alcohol (Table 1). In addition, the glucosamine content was significantly reduced by alcohol in this study. Glucosamine, a natural amino monosaccharide, is not only a dietary supplement for a variety of diseases but also an essential substance for the synthesis of proteoglycan in the matrix of human articular cartilage [18]. However, the effect of GDEE on these two metabolites was not been observed. The metabolite that deserved our most attention was (R)-mevalonic acid 5-phosphate, and it is an important intermediate product in the cholesterol synthesis pathway [19]. This study found that alcohol caused a significant increase in the content of (R)-mevalonic acid 5-phosphate, while GDEE could reduce its content (Table 1), which might be an important reason for GDEE to improve alcohol-induced steatosis.

The results of this study need to be treated with caution due to some shortcomings: (1) The analysis of the quality control samples showed that the stability of the instrument and experimental conditions was good. However, the number of repetitions was only three, making the results less convincing. (2) The identification of metabolites in this study was not been confirmed by authentic standards. Therefore, the metabolites identified in this study were speculative results. Further work needs to be done to confirm these metabolites.

## 5. Conclusion

This study revealed that the mechanism of GDEE in alleviating ALD had the characteristics of multi-target and multi-pathway, which were mainly involved in the dipeptides, purine and pyrimidine metabolism, and glucose and lipid metabolism. This can provide a basis for the in-depth study of GDEE to improve ALD.

## Figures and Tables

**Table 1:** Information on some differential metabolites.

Number	Metabolites	Content change		Classification	KEGG pathways
		Control vs Alcohol	Alcohol vs Alcohol + GDEE	(KEGG brite)	
1	Uridine 5′-diphosphate (UDP)	Up	Unchanged	Nucleic acids	(1); (2); (4)
2	Adenosine monophosphate (AMP)	Up	Unchanged	Nucleic acids	(1); (3); (4); (5); (6); (10)
3	Cytidine 5′-monophosphate (CMP)	Up	Unchanged	Nucleic acids	(1); (2)
4	Uracil	Down	Unchanged	Nucleic acids	(1); (2); (11)
5	Thymidine	Down	Unchanged	Nucleic acids	(1); (2)
6	Deoxyguanosine 5′-monophosphate (dGMP)	Up	Unchanged	Nucleic acids	(1); (3)
7	Deoxyuridine monophosphate (dUMP)	Down	Unchanged	Nucleic acids	(1); (2); (5)
8	2′-Deoxyuridine	Unchanged	Down	Nucleic acids	(1); (2); (7)
9	Adenosine 3′,5′-cyclic phosphate (cAMP)	Unchanged	Up	Nucleic acids	(1); (3); (6); (8); (9)
10	Adenylosuccinic acid	Unchanged	Up	Nucleic acids	(1); (3); (4); (12)
11	L-Alanine	Down	Unchanged	Amino acids	(1); (7); (12); (13); (14); (15)
12	L-Valine	Up	Unchanged	Amino acids	(1); (4); (11); (13); (14); (16)
13	Argininosuccinic acid	Up	Unchanged	Amino acids	(1); (12); (13)
14	Glucose 6-phosphate	Up	Unchanged	—	(1); (9); (15)
15	D-Xylose	Up	Down	Monosaccharides	(1); (7); (17); (18)
16	Glucosamine	Down	Unchanged	Monosaccharides	(1); (18)
17	(R)-Mevalonic acid 5-phosphate	Up	Down	—	(1); (19)

Note: the meaning of the numbers in column “KEGG pathways.” (1) metabolic pathways; (2) pyrimidine metabolism; (3) purine metabolism; (4) biosynthesis of cofactors; (5) antifolate resistance; (6) regulation of lipolysis in adipocytes; (7) ABC transporters; (8) insulin signaling pathway; (9) insulin secretion; (10) aldosterone synthesis and secretion; (11) pantothenate and CoA biosynthesis; (12) alanine, aspartate, and glutamate metabolism; (13) biosynthesis of amino acids; (14) protein digestion and absorption; (15) central carbon metabolism in cancer; (16) valine, leucine, and isoleucine degradation; (17) pentose and glucuronate interconversions; (18) amino sugar and nucleotide sugar metabolism; and (19) terpenoid backbone biosynthesis.

## Data Availability

All data are included within the manuscript.

## Supplementary Materials

Supplementary Materials “Supplementary information 1” contains the PCA results and Pearson correlation analysis of quality control samples. “Supplementary information 2” provides information on the content of all metabolites.
