# Supplementary material for: A Study on Gentiana dahurica Fisch Ethanol Extract Alleviating Alcoholic Liver Disease in Mice: A Metabolomic Analysis of the Liver
Source: Evid Based Complement Alternat Med. 2021 Jun 29;2021:5569538. doi: 10.1155/2021/5569538 (PMC8260312; doi:10.1155/2021/5569538)
Supplement: Supplementary Materials — “Supplementary information 1” contains the PCA results and Pearson correlation analysis of quality control samples. “Supplementary information 2” provides information on the content of all metabolites. [file 5569538.f1.zip › 5569538.f1/Supplementary information 1.docx]

The data for the quality control in the PCA and Pearson correlation analysis showed that the stability of the instrument and experimental conditions was good.

See Supplementary information 2 for the content of all metabolites in QC samples.


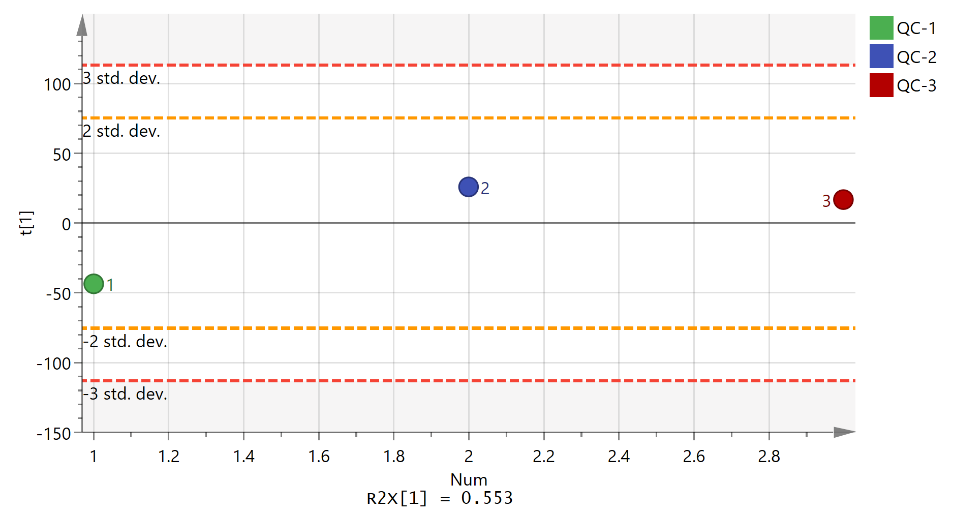


**Score scatter plot of PCA (positive ion mode)**


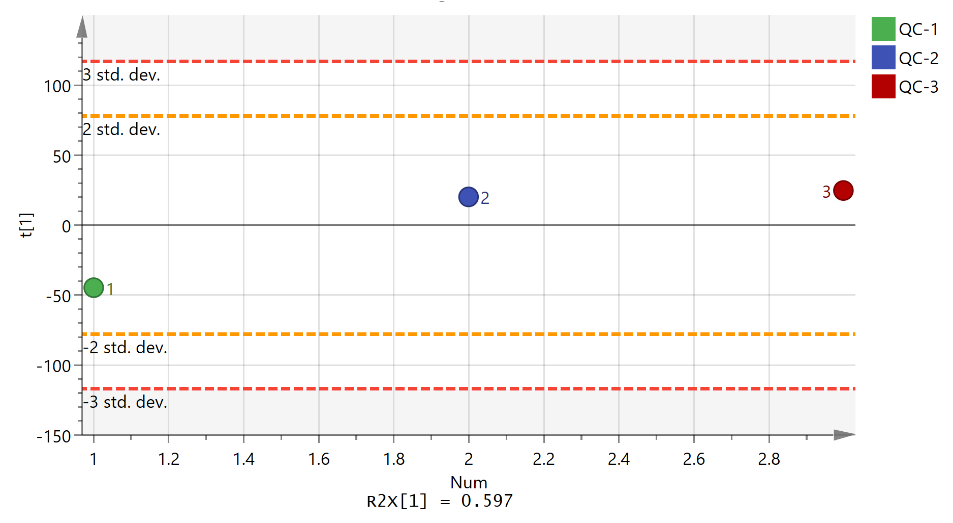


**Score scatter plot of PCA (negative ion mode)**

| **Pearson correlation analysis** | | | | | | | |
| --- | --- | --- | --- | --- | --- | --- | --- |
| Positive ion mode | | | | Negative ion mode | | | |
|  | QC-1 | QC-2 | QC-3 |  | QC-1 | QC-2 | QC-3 |
| QC-1 | 1 |  |  | QC-1 | 1 |  |  |
| QC-2 | 0.982 | 1 |  | QC-2 | 0.992 | 1 |  |
| QC-3 | 0.992 | 0.997 | 1 | QC-3 | 0.99 | 0.999 | 1 |
