# Supplementary material for: A Study on Gentiana dahurica Fisch Ethanol Extract Alleviating Alcoholic Liver Disease in Mice: A Metabolomic Analysis of the Liver
Source: Evid Based Complement Alternat Med. 2021 Jun 29;2021:5569538. doi: 10.1155/2021/5569538 (PMC8260312; doi:10.1155/2021/5569538)
Supplement: Supplementary Materials — “Supplementary information 1” contains the PCA results and Pearson correlation analysis of quality control samples. “Supplementary information 2” provides information on the content of all metabolites. [file 5569538.f1.zip › 5569538.f1/Supplementary information 2 (Negative ion mode).docx]

| **#ID** | **MS2 name** | **m/z (mass-to-charge ratio)** | **Rt (retention time,s)** | **QC-1** | **QC-2** | **QC-3** | **Control-1** | **Control-2** | **Control-3** | **Control-4** | **Alcohol-1** | **Alcohol-2** | **Alcohol-3** | **Alcohol-4** | **Alcohol+**  **GDEE-1** | **Alcohol+**  **GDEE-2** | **Alcohol+**  **GDEE-3** | **Alcohol+**  **GDEE-4** |
| --- | --- | --- | --- | --- | --- | --- | --- | --- | --- | --- | --- | --- | --- | --- | --- | --- | --- | --- |
| meta1 | Dihydroxyacetone | 71.013 | 86.456 | 0.031 | 0.031 | 0.040 | 0.081 | 0.031 | 0.060 | 0.036 | 0.033 | 0.029 | 0.034 | 0.029 | 0.056 | 0.033 | 0.032 | 0.035 |
| meta2 | Propionic acid | 73.029 | 372.420 | 0.034 | 0.029 | 0.034 | 0.038 | 0.037 | 0.017 | 0.039 | 0.033 | 0.031 | 0.029 | 0.033 | 0.025 | 0.042 | 0.021 | 0.029 |
| meta3 | Acetohydroxamic acid | 74.024 | 340.657 | 0.006 | 0.005 | 0.005 | 0.007 | 0.006 | 0.004 | 0.006 | 0.007 | 0.006 | 0.006 | 0.006 | 0.006 | 0.006 | 0.005 | 0.005 |
| meta4 |  | 76.970 | 50.037 | 0.574 | 0.569 | 0.532 | 0.639 | 0.509 | 0.420 | 0.516 | 0.480 | 0.501 | 0.518 | 0.495 | 0.439 | 0.518 | 0.459 | 0.457 |
| meta5 |  | 79.957 | 274.292 | 0.208 | 0.165 | 0.174 | 0.206 | 0.219 | 0.163 | 0.159 | 0.223 | 0.175 | 0.226 | 0.161 | 0.134 | 0.178 | 0.169 | 0.167 |
| meta6 |  | 80.965 | 98.168 | 0.142 | 0.131 | 0.137 | 0.008 | 0.024 | 0.006 | 0.241 | 0.345 | 0.227 | 0.149 | 0.051 | 0.083 | 0.133 | 0.013 | 0.044 |
| meta7 |  | 85.029 | 283.143 | 0.016 | 0.012 | 0.020 | 0.020 | 0.016 | 0.012 | 0.019 | 0.016 | 0.010 | 0.010 | 0.012 | 0.011 | 0.021 | 0.018 | 0.013 |
| meta8 |  | 87.008 | 129.716 | 0.087 | 0.079 | 0.106 | 0.097 | 0.070 | 0.066 | 0.085 | 0.058 | 0.052 | 0.209 | 0.051 | 0.056 | 0.070 | 0.062 | 0.074 |
| meta9 | L-Alanine | 88.040 | 388.832 | 0.007 | 0.004 | 0.005 | 0.007 | 0.005 | 0.003 | 0.004 | 0.006 | 0.005 | 0.008 | 0.005 | 0.006 | 0.006 | 0.004 | 0.005 |
| meta10 | Glyceraldehyde | 89.024 | 85.738 | 0.164 | 0.157 | 0.159 | 0.211 | 0.178 | 0.142 | 0.193 | 0.167 | 0.143 | 0.164 | 0.141 | 0.133 | 0.153 | 0.126 | 0.177 |
| meta11 | DL-lactate | 89.025 | 214.193 | 0.932 | 0.790 | 0.797 | 1.146 | 0.954 | 0.908 | 0.939 | 0.768 | 0.376 | 0.738 | 0.607 | 0.661 | 0.866 | 0.530 | 0.884 |
| meta12 |  | 91.956 | 49.998 | 0.022 | 0.031 | 0.021 | 0.027 | 0.023 | 0.017 | 0.023 | 0.020 | 0.022 | 0.024 | 0.020 | 0.019 | 0.021 | 0.019 | 0.018 |
| meta13 |  | 92.025 | 155.949 | 0.035 | 0.029 | 0.029 | 0.040 | 0.044 | 0.037 | 0.045 | 0.027 | 0.020 | 0.024 | 0.029 | 0.036 | 0.030 | 0.034 | 0.026 |
| meta14 |  | 99.006 | 318.133 | 0.025 | 0.022 | 0.024 | 0.028 | 0.024 | 0.026 | 0.025 | 0.022 | 0.027 | 0.023 | 0.023 | 0.022 | 0.027 | 0.027 | 0.022 |
| meta15 | 3-Hydroxyisovaleric acid | 99.044 | 360.743 | 0.001 | 0.000 | 0.001 | 0.001 | 0.003 | 0.002 | 0.003 | 0.001 | 0.001 | 0.001 | 0.001 | 0.001 | 0.001 | 0.000 | 0.002 |
| meta16 |  | 100.985 | 318.690 | 0.016 | 0.014 | 0.016 | 0.018 | 0.016 | 0.015 | 0.016 | 0.015 | 0.015 | 0.015 | 0.015 | 0.014 | 0.017 | 0.017 | 0.016 |
| meta17 |  | 101.024 | 283.167 | 0.017 | 0.015 | 0.025 | 0.023 | 0.019 | 0.018 | 0.023 | 0.030 | 0.012 | 0.016 | 0.014 | 0.016 | 0.019 | 0.015 | 0.014 |
| meta18 |  | 102.056 | 381.377 | 0.024 | 0.022 | 0.022 | 0.027 | 0.030 | 0.016 | 0.023 | 0.033 | 0.025 | 0.028 | 0.028 | 0.024 | 0.028 | 0.018 | 0.018 |
| meta19 | Malonic acid | 103.003 | 377.307 | 0.012 | 0.012 | 0.010 | 0.013 | 0.011 | 0.009 | 0.010 | 0.015 | 0.010 | 0.017 | 0.012 | 0.010 | 0.014 | 0.010 | 0.009 |
| meta20 | D(-)-beta-hydroxy butyric acid | 103.039 | 218.023 | 0.030 | 0.026 | 0.027 | 0.022 | 0.054 | 0.017 | 0.047 | 0.029 | 0.010 | 0.012 | 0.017 | 0.020 | 0.033 | 0.028 | 0.025 |
| meta21 | 2-hydroxy-butanoic acid | 103.039 | 181.838 | 0.018 | 0.015 | 0.017 | 0.011 | 0.028 | 0.011 | 0.032 | 0.028 | 0.009 | 0.012 | 0.017 | 0.012 | 0.013 | 0.008 | 0.014 |
| meta22 |  | 104.034 | 357.201 | 0.009 | 0.008 | 0.007 | 0.009 | 0.008 | 0.005 | 0.009 | 0.009 | 0.008 | 0.009 | 0.008 | 0.010 | 0.009 | 0.007 | 0.008 |
| meta23 |  | 108.019 | 197.107 | 0.018 | 0.016 | 0.017 | 0.023 | 0.022 | 0.015 | 0.022 | 0.018 | 0.012 | 0.015 | 0.017 | 0.018 | 0.020 | 0.017 | 0.017 |
| meta24 |  | 109.040 | 351.613 | 0.009 | 0.008 | 0.007 | 0.009 | 0.009 | 0.007 | 0.009 | 0.011 | 0.009 | 0.010 | 0.009 | 0.008 | 0.010 | 0.007 | 0.008 |
| meta25 | Pyrrole-2-carboxylic acid | 110.024 | 150.838 | 0.014 | 0.012 | 0.013 | 0.014 | 0.019 | 0.009 | 0.023 | 0.015 | 0.007 | 0.007 | 0.010 | 0.012 | 0.010 | 0.009 | 0.010 |
| meta26 | Uracil | 111.019 | 85.976 | 0.054 | 0.057 | 0.056 | 0.069 | 0.067 | 0.084 | 0.068 | 0.037 | 0.041 | 0.027 | 0.030 | 0.050 | 0.036 | 0.075 | 0.041 |
| meta27 |  | 112.039 | 196.540 | 0.011 | 0.009 | 0.010 | 0.012 | 0.003 | 0.010 | 0.004 | 0.008 | 0.004 | 0.009 | 0.014 | 0.009 | 0.005 | 0.005 | 0.017 |
| meta28 | Creatinine | 112.051 | 155.969 | 0.003 | 0.003 | 0.003 | 0.004 | 0.004 | 0.003 | 0.004 | 0.003 | 0.002 | 0.003 | 0.002 | 0.002 | 0.002 | 0.002 | 0.002 |
| meta29 |  | 113.023 | 282.536 | 0.065 | 0.053 | 0.092 | 0.079 | 0.073 | 0.054 | 0.079 | 0.073 | 0.042 | 0.050 | 0.051 | 0.057 | 0.066 | 0.051 | 0.050 |
| meta30 | L-Proline | 114.055 | 287.703 | 0.024 | 0.019 | 0.020 | 0.028 | 0.023 | 0.019 | 0.029 | 0.026 | 0.017 | 0.023 | 0.025 | 0.020 | 0.018 | 0.014 | 0.022 |
| meta31 | Fumarate | 115.003 | 52.056 | 0.011 | 0.007 | 0.010 | 0.016 | 0.008 | 0.011 | 0.012 | 0.030 | 0.009 | 0.015 | 0.013 | 0.011 | 0.013 | 0.009 | 0.008 |
| meta32 |  | 115.020 | 215.066 | 0.031 | 0.029 | 0.028 | 0.037 | 0.036 | 0.029 | 0.038 | 0.030 | 0.013 | 0.025 | 0.021 | 0.022 | 0.033 | 0.017 | 0.030 |
| meta33 |  | 116.034 | 389.601 | 0.004 | 0.004 | 0.002 | 0.004 | 0.005 | 0.003 | 0.003 | 0.003 | 0.002 | 0.003 | 0.003 | 0.004 | 0.005 | 0.004 | 0.003 |
| meta34 | Acetylglycine | 116.034 | 264.427 | 0.002 | 0.002 | 0.002 | 0.002 | 0.003 | 0.002 | 0.003 | 0.003 | 0.002 | 0.002 | 0.002 | 0.003 | 0.002 | 0.002 | 0.002 |
| meta35 | L-Valine | 116.071 | 279.352 | 0.035 | 0.027 | 0.030 | 0.035 | 0.039 | 0.024 | 0.041 | 0.041 | 0.025 | 0.037 | 0.041 | 0.034 | 0.027 | 0.021 | 0.031 |
| meta36 | Succinate | 117.019 | 372.420 | 0.143 | 0.107 | 0.105 | 0.153 | 0.150 | 0.066 | 0.157 | 0.120 | 0.116 | 0.112 | 0.131 | 0.092 | 0.169 | 0.079 | 0.111 |
| meta37 | Methylmalonic acid | 117.019 | 47.239 | 0.020 | 0.015 | 0.017 | 0.020 | 0.018 | 0.016 | 0.006 | 0.022 | 0.017 | 0.006 | 0.010 | 0.015 | 0.019 | 0.008 | 0.020 |
| meta38 | 2-Methyl-3-hydroxybutyric acid | 117.054 | 167.716 | 0.001 | 0.001 | 0.001 | 0.001 | 0.001 | 0.001 | 0.001 | 0.001 | 0.001 | 0.001 | 0.001 | 0.001 | 0.001 | 0.001 | 0.001 |
| meta39 |  | 119.975 | 40.077 | 0.365 | 0.349 | 0.353 | 0.393 | 0.374 | 0.295 | 0.320 | 0.613 | 0.410 | 0.536 | 0.408 | 0.197 | 0.521 | 0.516 | 0.436 |
| meta40 | Benzoic acid | 121.028 | 117.861 | 0.022 | 0.024 | 0.025 | 0.022 | 0.018 | 0.016 | 0.016 | 0.032 | 0.018 | 0.019 | 0.016 | 0.017 | 0.017 | 0.017 | 0.045 |
| meta41 |  | 121.044 | 372.230 | 0.056 | 0.044 | 0.045 | 0.051 | 0.047 | 0.049 | 0.048 | 0.053 | 0.051 | 0.053 | 0.050 | 0.050 | 0.053 | 0.049 | 0.049 |
| meta42 | Nicotinamide | 121.040 | 59.202 | 0.043 | 0.044 | 0.046 | 0.050 | 0.051 | 0.039 | 0.052 | 0.048 | 0.038 | 0.043 | 0.048 | 0.051 | 0.042 | 0.043 | 0.043 |
| meta43 |  | 122.966 | 318.308 | 0.023 | 0.019 | 0.022 | 0.024 | 0.022 | 0.022 | 0.021 | 0.021 | 0.022 | 0.021 | 0.021 | 0.020 | 0.024 | 0.023 | 0.020 |
| meta44 | Taurine | 124.007 | 274.366 | 2.081 | 1.727 | 1.759 | 2.318 | 2.281 | 1.699 | 1.826 | 2.357 | 1.633 | 2.231 | 1.659 | 1.461 | 1.799 | 1.700 | 1.739 |
| meta45 |  | 124.990 | 152.037 | 0.150 | 0.133 | 0.140 | 0.132 | 0.169 | 0.121 | 0.108 | 0.167 | 0.123 | 0.160 | 0.118 | 0.026 | 0.128 | 0.179 | 0.097 |
| meta46 |  | 124.990 | 33.880 | 0.004 | 0.004 | 0.004 | 0.002 | 0.001 | 0.002 | 0.002 | 0.006 | 0.004 | 0.007 | 0.013 | 0.006 | 0.002 | 0.005 | 0.006 |
| meta47 | Ammelide | 127.025 | 678.525 | 0.002 | 0.003 | 0.002 | 0.002 | 0.002 | 0.001 | 0.002 | 0.003 | 0.002 | 0.002 | 0.002 | 0.001 | 0.002 | 0.002 | 0.003 |
| meta48 | L-Pyroglutamic acid | 128.034 | 351.589 | 0.025 | 0.021 | 0.028 | 0.037 | 0.025 | 0.020 | 0.024 | 0.029 | 0.026 | 0.028 | 0.025 | 0.022 | 0.029 | 0.021 | 0.030 |
| meta49 | O-Acetyl-L-serine | 128.034 | 258.664 | 0.012 | 0.011 | 0.017 | 0.009 | 0.012 | 0.007 | 0.021 | 0.007 | 0.003 | 0.009 | 0.007 | 0.008 | 0.014 | 0.008 | 0.008 |
| meta50 | Cyanuric acid | 129.018 | 77.162 | 0.075 | 0.094 | 0.107 | 0.116 | 0.094 | 0.039 | 0.072 | 0.042 | 0.095 | 0.040 | 0.035 | 0.071 | 0.097 | 0.039 | 0.096 |
| meta51 |  | 129.091 | 62.715 | 0.026 | 0.027 | 0.027 | 0.041 | 0.029 | 0.047 | 0.030 | 0.029 | 0.044 | 0.023 | 0.023 | 0.025 | 0.025 | 0.026 | 0.024 |
| meta52 | N-Acetyl-L-alanine | 130.050 | 236.506 | 0.003 | 0.002 | 0.002 | 0.002 | 0.002 | 0.003 | 0.002 | 0.002 | 0.001 | 0.002 | 0.002 | 0.002 | 0.002 | 0.002 | 0.001 |
| meta53 | L-Isoleucine | 130.087 | 243.985 | 0.155 | 0.129 | 0.131 | 0.147 | 0.156 | 0.139 | 0.204 | 0.173 | 0.111 | 0.148 | 0.176 | 0.153 | 0.126 | 0.095 | 0.151 |
| meta54 |  | 130.086 | 176.673 | 0.003 | 0.004 | 0.004 | 0.004 | 0.003 | 0.004 | 0.005 | 0.003 | 0.002 | 0.004 | 0.005 | 0.003 | 0.002 | 0.001 | 0.005 |
| meta55 |  | 130.888 | 158.634 | 0.003 | 0.003 | 0.003 | 0.006 | 0.005 | 0.002 | 0.003 | 0.002 | 0.006 | 0.002 | 0.004 | 0.004 | 0.004 | 0.003 | 0.003 |
| meta56 | Pyruvaldehyde | 131.034 | 87.285 | 0.138 | 0.132 | 0.095 | 0.127 | 0.072 | 0.051 | 0.146 | 0.082 | 0.046 | 0.070 | 0.082 | 0.093 | 0.108 | 0.099 | 0.111 |
| meta57 | L-Asparagine | 131.045 | 359.001 | 0.009 | 0.008 | 0.008 | 0.009 | 0.007 | 0.008 | 0.008 | 0.009 | 0.007 | 0.010 | 0.009 | 0.010 | 0.010 | 0.007 | 0.007 |
| meta58 | 2-Ethyl-2-Hydroxybutyric acid | 131.070 | 131.438 | 0.006 | 0.007 | 0.009 | 0.006 | 0.006 | 0.004 | 0.007 | 0.007 | 0.003 | 0.004 | 0.005 | 0.008 | 0.005 | 0.001 | 0.006 |
| meta59 |  | 131.082 | 368.360 | 0.004 | 0.004 | 0.004 | 0.006 | 0.006 | 0.003 | 0.005 | 0.005 | 0.003 | 0.005 | 0.003 | 0.004 | 0.003 | 0.003 | 0.004 |
| meta60 | L-Aspartate | 132.029 | 438.129 | 0.003 | 0.003 | 0.002 | 0.003 | 0.003 | 0.002 | 0.002 | 0.003 | 0.003 | 0.004 | 0.002 | 0.004 | 0.003 | 0.002 | 0.002 |
| meta61 | D-Aspartic acid | 132.029 | 412.110 | 0.022 | 0.018 | 0.017 | 0.022 | 0.021 | 0.007 | 0.017 | 0.023 | 0.012 | 0.027 | 0.015 | 0.021 | 0.025 | 0.015 | 0.019 |
| meta62 | L-Malic acid | 133.013 | 425.755 | 0.214 | 0.172 | 0.163 | 0.225 | 0.201 | 0.170 | 0.160 | 0.122 | 0.199 | 0.402 | 0.242 | 0.083 | 0.099 | 0.139 | 0.163 |
| meta63 | Glyoxylate | 133.014 | 197.134 | 0.001 | 0.001 | 0.001 | 0.001 | 0.001 | 0.001 | 0.001 | 0.001 | 0.001 | 0.001 | 0.001 | 0.001 | 0.001 | 0.001 | 0.001 |
| meta64 |  | 133.991 | 44.736 | 0.012 | 0.012 | 0.013 | 0.034 | 0.019 | 0.011 | 0.007 | 0.006 | 0.007 | 0.006 | 0.005 | 0.004 | 0.006 | 0.004 | 0.003 |
| meta65 | Adenine | 134.046 | 192.456 | 0.061 | 0.055 | 0.057 | 0.095 | 0.058 | 0.069 | 0.086 | 0.053 | 0.057 | 0.052 | 0.051 | 0.062 | 0.061 | 0.051 | 0.055 |
| meta66 | Hypoxanthine | 135.030 | 200.071 | 0.053 | 0.049 | 0.050 | 0.059 | 0.070 | 0.052 | 0.073 | 0.041 | 0.031 | 0.036 | 0.043 | 0.055 | 0.050 | 0.054 | 0.043 |
| meta67 | L-Threonate | 135.029 | 324.606 | 0.011 | 0.011 | 0.017 | 0.033 | 0.015 | 0.013 | 0.010 | 0.027 | 0.009 | 0.015 | 0.014 | 0.019 | 0.025 | 0.011 | 0.010 |
| meta68 | Bovinocidin (3-Nitropropionic Acid) | 135.044 | 34.892 | 0.005 | 0.005 | 0.005 | 0.005 | 0.005 | 0.005 | 0.006 | 0.006 | 0.005 | 0.001 | 0.006 | 0.005 | 0.005 | 0.006 | 0.007 |
| meta69 |  | 136.007 | 98.082 | 2.116 | 2.022 | 1.849 | 0.058 | 0.273 | 0.052 | 3.337 | 4.696 | 3.170 | 2.125 | 0.747 | 1.175 | 1.860 | 0.121 | 0.637 |
| meta70 |  | 136.069 | 174.343 | 0.002 | 0.002 | 0.003 | 0.002 | 0.003 | 0.003 | 0.001 | 0.002 | 0.002 | 0.002 | 0.002 | 0.002 | 0.002 | 0.002 | 0.002 |
| meta71 |  | 136.963 | 433.927 | 0.009 | 0.006 | 0.006 | 0.008 | 0.008 | 0.005 | 0.007 | 0.014 | 0.005 | 0.016 | 0.019 | 0.020 | 0.016 | 0.006 | 0.017 |
| meta72 |  | 137.035 | 79.438 | 0.071 | 0.067 | 0.073 | 0.117 | 0.072 | 0.102 | 0.059 | 0.054 | 0.073 | 0.058 | 0.099 | 0.075 | 0.040 | 0.061 | 0.025 |
| meta73 | 4-Nitrophenol | 138.019 | 36.048 | 0.012 | 0.016 | 0.015 | 0.020 | 0.017 | 0.014 | 0.019 | 0.020 | 0.016 | 0.017 | 0.015 | 0.017 | 0.018 | 0.015 | 0.018 |
| meta74 | Acetyl phosphate | 138.979 | 427.488 | 0.020 | 0.017 | 0.017 | 0.024 | 0.019 | 0.013 | 0.017 | 0.018 | 0.015 | 0.017 | 0.018 | 0.019 | 0.018 | 0.017 | 0.017 |
| meta75 |  | 139.025 | 267.858 | 0.023 | 0.021 | 0.022 | 0.026 | 0.023 | 0.018 | 0.026 | 0.019 | 0.018 | 0.015 | 0.019 | 0.018 | 0.028 | 0.017 | 0.027 |
| meta76 |  | 139.050 | 293.504 | 0.001 | 0.001 | 0.001 | 0.001 | 0.001 | 0.001 | 0.002 | 0.002 | 0.001 | 0.002 | 0.001 | 0.002 | 0.002 | 0.001 | 0.001 |
| meta77 |  | 139.092 | 174.333 | 0.091 | 0.092 | 0.092 | 0.093 | 0.088 | 0.088 | 0.094 | 0.093 | 0.098 | 0.090 | 0.092 | 0.091 | 0.092 | 0.089 | 0.090 |
| meta78 |  | 140.034 | 243.291 | 0.008 | 0.007 | 0.008 | 0.009 | 0.010 | 0.007 | 0.009 | 0.007 | 0.006 | 0.007 | 0.007 | 0.007 | 0.009 | 0.006 | 0.008 |
| meta79 | 2-Oxoadipic acid | 141.016 | 318.096 | 0.102 | 0.088 | 0.111 | 0.116 | 0.101 | 0.118 | 0.103 | 0.095 | 0.117 | 0.094 | 0.093 | 0.091 | 0.121 | 0.127 | 0.093 |
| meta80 | Glyceric acid | 142.974 | 285.940 | 0.003 | 0.003 | 0.002 | 0.003 | 0.003 | 0.002 | 0.003 | 0.003 | 0.003 | 0.003 | 0.003 | 0.003 | 0.003 | 0.002 | 0.003 |
| meta81 | Erythritol | 143.034 | 282.536 | 0.034 | 0.030 | 0.048 | 0.048 | 0.038 | 0.029 | 0.043 | 0.040 | 0.023 | 0.025 | 0.024 | 0.027 | 0.035 | 0.026 | 0.029 |
| meta82 |  | 143.107 | 46.676 | 0.022 | 0.025 | 0.023 | 0.027 | 0.025 | 0.020 | 0.025 | 0.026 | 0.029 | 0.024 | 0.023 | 0.024 | 0.026 | 0.023 | 0.027 |
| meta83 |  | 144.029 | 278.449 | 0.027 | 0.022 | 0.028 | 0.028 | 0.026 | 0.019 | 0.027 | 0.036 | 0.029 | 0.021 | 0.022 | 0.031 | 0.030 | 0.028 | 0.028 |
| meta84 | Barbituric acid | 144.044 | 169.671 | 0.002 | 0.001 | 0.001 | 0.002 | 0.001 | 0.001 | 0.002 | 0.002 | 0.001 | 0.001 | 0.001 | 0.001 | 0.001 | 0.001 | 0.002 |
| meta85 | Isobutyrylglycine | 144.066 | 200.349 | 0.019 | 0.020 | 0.017 | 0.016 | 0.024 | 0.011 | 0.032 | 0.034 | 0.008 | 0.011 | 0.012 | 0.017 | 0.014 | 0.015 | 0.021 |
| meta86 | 4-Guanidinobutyric acid | 144.077 | 332.635 | 0.000 | 0.000 | 0.000 | 0.001 | 0.000 | 0.000 | 0.000 | 0.001 | 0.001 | 0.001 | 0.001 | 0.000 | 0.000 | 0.000 | 0.000 |
| meta87 |  | 145.014 | 356.501 | 0.005 | 0.006 | 0.006 | 0.006 | 0.006 | 0.004 | 0.005 | 0.008 | 0.005 | 0.006 | 0.006 | 0.005 | 0.007 | 0.005 | 0.004 |
| meta88 | L-Glutamine | 145.061 | 351.585 | 0.363 | 0.312 | 0.293 | 0.402 | 0.353 | 0.283 | 0.338 | 0.433 | 0.363 | 0.372 | 0.352 | 0.327 | 0.410 | 0.298 | 0.329 |
| meta89 |  | 146.045 | 280.056 | 0.022 | 0.017 | 0.018 | 0.020 | 0.018 | 0.016 | 0.019 | 0.022 | 0.013 | 0.020 | 0.023 | 0.025 | 0.018 | 0.012 | 0.020 |
| meta90 | L-Glutamate | 146.045 | 381.377 | 0.194 | 0.176 | 0.161 | 0.211 | 0.223 | 0.125 | 0.174 | 0.252 | 0.196 | 0.221 | 0.221 | 0.187 | 0.208 | 0.149 | 0.141 |
| meta91 |  | 146.081 | 329.808 | 0.006 | 0.004 | 0.005 | 0.007 | 0.006 | 0.005 | 0.006 | 0.006 | 0.004 | 0.005 | 0.005 | 0.005 | 0.005 | 0.004 | 0.005 |
| meta92 | (S)-2-Hydroxyglutarate | 147.029 | 383.939 | 0.008 | 0.007 | 0.008 | 0.010 | 0.008 | 0.007 | 0.009 | 0.010 | 0.007 | 0.010 | 0.007 | 0.009 | 0.010 | 0.006 | 0.007 |
| meta93 | D-Arabinono-1,4-lactone | 147.029 | 78.824 | 0.224 | 0.218 | 0.226 | 0.249 | 0.214 | 0.244 | 0.198 | 0.232 | 0.222 | 0.240 | 0.194 | 0.151 | 0.206 | 0.204 | 0.222 |
| meta94 |  | 147.044 | 237.953 | 0.011 | 0.010 | 0.010 | 0.011 | 0.012 | 0.009 | 0.015 | 0.012 | 0.008 | 0.011 | 0.013 | 0.012 | 0.009 | 0.007 | 0.012 |
| meta95 | L-Methionine | 148.043 | 263.323 | 0.004 | 0.004 | 0.004 | 0.006 | 0.004 | 0.005 | 0.005 | 0.005 | 0.003 | 0.004 | 0.005 | 0.004 | 0.003 | 0.003 | 0.003 |
| meta96 | D-Ribose | 149.044 | 283.143 | 0.027 | 0.023 | 0.025 | 0.034 | 0.029 | 0.024 | 0.033 | 0.030 | 0.019 | 0.022 | 0.020 | 0.022 | 0.029 | 0.021 | 0.022 |
| meta97 |  | 150.567 | 197.116 | 0.001 | 0.001 | 0.001 | 0.001 | 0.001 | 0.001 | 0.001 | 0.001 | 0.001 | 0.001 | 0.001 | 0.001 | 0.001 | 0.001 | 0.001 |
| meta98 | Xanthine | 151.026 | 196.906 | 0.319 | 0.306 | 0.317 | 0.443 | 0.413 | 0.303 | 0.389 | 0.328 | 0.223 | 0.266 | 0.290 | 0.327 | 0.382 | 0.314 | 0.292 |
| meta99 | N1-Methyl-2-pyridone-5-carboxamide | 151.050 | 80.900 | 0.005 | 0.005 | 0.006 | 0.006 | 0.003 | 0.005 | 0.006 | 0.006 | 0.005 | 0.008 | 0.006 | 0.006 | 0.006 | 0.004 | 0.007 |
| meta100 | Ribitol | 151.060 | 222.784 | 0.008 | 0.005 | 0.006 | 0.007 | 0.008 | 0.005 | 0.011 | 0.006 | 0.003 | 0.004 | 0.004 | 0.006 | 0.004 | 0.006 | 0.003 |
| meta101 |  | 152.002 | 165.702 | 0.214 | 0.188 | 0.189 | 0.128 | 0.185 | 0.100 | 0.279 | 0.625 | 0.235 | 0.224 | 0.243 | 0.162 | 0.181 | 0.085 | 0.149 |
| meta102 |  | 153.067 | 174.106 | 0.002 | 0.003 | 0.002 | 0.001 | 0.001 | 0.001 | 0.007 | 0.005 | 0.002 | 0.002 | 0.002 | 0.005 | 0.002 | 0.002 | 0.001 |
| meta103 | L-Histidine | 154.061 | 298.118 | 0.001 | 0.001 | 0.001 | 0.001 | 0.001 | 0.001 | 0.001 | 0.001 | 0.001 | 0.001 | 0.001 | 0.001 | 0.001 | 0.001 | 0.001 |
| meta104 |  | 154.946 | 50.661 | 0.102 | 0.092 | 0.089 | 0.114 | 0.090 | 0.073 | 0.088 | 0.083 | 0.089 | 0.096 | 0.087 | 0.076 | 0.085 | 0.079 | 0.087 |
| meta105 |  | 154.946 | 342.513 | 0.080 | 0.043 | 0.065 | 0.052 | 0.071 | 0.031 | 0.044 | 0.039 | 0.042 | 0.040 | 0.076 | 0.043 | 0.039 | 0.043 | 0.045 |
| meta106 |  | 154.947 | 503.562 | 0.414 | 0.379 | 0.342 | 0.471 | 0.391 | 0.279 | 0.380 | 0.139 | 0.343 | 0.413 | 0.367 | 0.056 | 0.335 | 0.326 | 0.707 |
| meta107 |  | 154.974 | 458.759 | 0.015 | 0.011 | 0.016 | 0.024 | 0.026 | 0.009 | 0.019 | 0.019 | 0.021 | 0.019 | 0.007 | 0.020 | 0.042 | 0.021 | 0.005 |
| meta108 |  | 156.029 | 383.555 | 0.010 | 0.009 | 0.008 | 0.010 | 0.010 | 0.007 | 0.006 | 0.009 | 0.007 | 0.010 | 0.011 | 0.010 | 0.016 | 0.011 | 0.008 |
| meta109 |  | 156.066 | 390.124 | 0.003 | 0.003 | 0.003 | 0.003 | 0.005 | 0.002 | 0.002 | 0.002 | 0.002 | 0.002 | 0.002 | 0.004 | 0.005 | 0.004 | 0.002 |
| meta110 |  | 156.991 | 286.377 | 0.555 | 0.435 | 0.421 | 0.666 | 0.615 | 0.466 | 0.619 | 0.583 | 0.525 | 0.515 | 0.516 | 0.530 | 0.600 | 0.481 | 0.481 |
| meta111 |  | 157.012 | 154.670 | 0.011 | 0.008 | 0.008 | 0.011 | 0.010 | 0.009 | 0.010 | 0.009 | 0.007 | 0.009 | 0.009 | 0.011 | 0.010 | 0.009 | 0.009 |
| meta112 |  | 157.013 | 440.171 | 0.016 | 0.014 | 0.014 | 0.023 | 0.007 | 0.025 | 0.007 | 0.010 | 0.010 | 0.011 | 0.009 | 0.016 | 0.012 | 0.015 | 0.008 |
| meta113 | Allantoin | 157.036 | 170.385 | 0.040 | 0.036 | 0.037 | 0.040 | 0.046 | 0.038 | 0.036 | 0.044 | 0.025 | 0.038 | 0.033 | 0.048 | 0.037 | 0.030 | 0.039 |
| meta114 |  | 157.061 | 250.848 | 0.007 | 0.007 | 0.007 | 0.001 | 0.002 | 0.000 | 0.014 | 0.014 | 0.013 | 0.005 | 0.004 | 0.008 | 0.007 | 0.001 | 0.003 |
| meta115 |  | 157.123 | 46.675 | 0.068 | 0.097 | 0.092 | 0.097 | 0.074 | 0.061 | 0.082 | 0.084 | 0.094 | 0.077 | 0.081 | 0.076 | 0.079 | 0.110 | 0.175 |
| meta116 | Alloxan | 158.019 | 309.559 | 0.000 | 0.000 | 0.000 | 0.000 | 0.000 | 0.000 | 0.000 | 0.000 | 0.000 | 0.000 | 0.000 | 0.000 | 0.000 | 0.000 | 0.000 |
| meta117 | 5-Hydroxymethyluracil | 158.060 | 34.020 | 0.028 | 0.028 | 0.033 | 0.039 | 0.039 | 0.030 | 0.038 | 0.031 | 0.031 | 0.035 | 0.040 | 0.032 | 0.031 | 0.028 | 0.037 |
| meta118 | Isovalerylglycine | 158.081 | 179.290 | 0.023 | 0.021 | 0.023 | 0.022 | 0.027 | 0.015 | 0.041 | 0.032 | 0.011 | 0.014 | 0.014 | 0.026 | 0.013 | 0.018 | 0.021 |
| meta119 | Oxoadipic acid | 159.029 | 133.297 | 0.005 | 0.004 | 0.004 | 0.006 | 0.010 | 0.004 | 0.005 | 0.004 | 0.003 | 0.002 | 0.003 | 0.003 | 0.003 | 0.003 | 0.004 |
| meta120 | D-Alanyl-D-alanine (D-Ala-D-Ala) | 159.076 | 305.614 | 0.003 | 0.002 | 0.002 | 0.005 | 0.002 | 0.004 | 0.003 | 0.001 | 0.001 | 0.002 | 0.001 | 0.002 | 0.003 | 0.002 | 0.002 |
| meta121 |  | 160.061 | 263.376 | 0.002 | 0.002 | 0.002 | 0.002 | 0.004 | 0.002 | 0.003 | 0.003 | 0.002 | 0.001 | 0.002 | 0.003 | 0.002 | 0.002 | 0.002 |
| meta122 | DL-2-Aminoadipic acid | 160.060 | 393.403 | 0.008 | 0.007 | 0.006 | 0.009 | 0.013 | 0.004 | 0.008 | 0.007 | 0.005 | 0.009 | 0.008 | 0.006 | 0.007 | 0.003 | 0.005 |
| meta123 |  | 160.948 | 288.888 | 0.017 | 0.016 | 0.018 | 0.022 | 0.019 | 0.017 | 0.020 | 0.018 | 0.022 | 0.019 | 0.017 | 0.019 | 0.021 | 0.018 | 0.018 |
| meta124 |  | 161.045 | 368.918 | 0.097 | 0.088 | 0.089 | 0.099 | 0.106 | 0.117 | 0.087 | 0.101 | 0.066 | 0.097 | 0.092 | 0.117 | 0.084 | 0.081 | 0.051 |
| meta125 | L-Sorbose | 161.044 | 380.742 | 0.014 | 0.012 | 0.011 | 0.016 | 0.015 | 0.013 | 0.011 | 0.012 | 0.010 | 0.012 | 0.011 | 0.012 | 0.015 | 0.013 | 0.010 |
| meta126 |  | 162.521 | 97.936 | 0.004 | 0.004 | 0.004 | 0.000 | 0.001 | 0.000 | 0.006 | 0.011 | 0.006 | 0.005 | 0.002 | 0.002 | 0.004 | 0.000 | 0.002 |
| meta127 |  | 163.018 | 97.586 | 1.767 | 1.740 | 1.706 | 0.036 | 0.215 | 0.036 | 3.177 | 4.117 | 2.824 | 1.831 | 0.598 | 0.900 | 1.655 | 0.087 | 0.543 |
| meta128 | 4-Hydroxycinnamic acid | 163.039 | 179.420 | 0.003 | 0.003 | 0.003 | 0.004 | 0.001 | 0.002 | 0.002 | 0.002 | 0.002 | 0.004 | 0.002 | 0.003 | 0.002 | 0.004 | 0.004 |
| meta129 | Phenylpyruvate | 163.039 | 90.728 | 0.004 | 0.003 | 0.005 | 0.005 | 0.011 | 0.010 | 0.044 | 0.005 | 0.016 | 0.004 | 0.004 | 0.005 | 0.003 | 0.018 | 0.006 |
| meta130 | L-Iditol | 163.060 | 190.205 | 0.008 | 0.007 | 0.008 | 0.009 | 0.007 | 0.007 | 0.009 | 0.011 | 0.005 | 0.009 | 0.008 | 0.010 | 0.007 | 0.007 | 0.006 |
| meta131 | D-Sorbitol | 163.060 | 265.970 | 0.001 | 0.001 | 0.001 | 0.001 | 0.001 | 0.001 | 0.000 | 0.000 | 0.001 | 0.000 | 0.000 | 0.000 | 0.000 | 0.001 | 0.000 |
| meta132 | N-formylanthranilic acid | 164.037 | 47.274 | 0.014 | 0.013 | 0.014 | 0.010 | 0.016 | 0.012 | 0.011 | 0.012 | 0.006 | 0.011 | 0.006 | 0.006 | 0.013 | 0.012 | 0.006 |
| meta133 | L-Phenylalanine | 164.071 | 237.992 | 0.069 | 0.056 | 0.058 | 0.065 | 0.071 | 0.056 | 0.088 | 0.076 | 0.051 | 0.064 | 0.078 | 0.072 | 0.057 | 0.047 | 0.067 |
| meta134 |  | 164.977 | 318.126 | 0.018 | 0.015 | 0.017 | 0.019 | 0.017 | 0.018 | 0.018 | 0.015 | 0.020 | 0.017 | 0.017 | 0.015 | 0.019 | 0.020 | 0.016 |
| meta135 | Dihydrothymine | 165.004 | 267.586 | 0.001 | 0.001 | 0.001 | 0.002 | 0.001 | 0.001 | 0.002 | 0.001 | 0.001 | 0.001 | 0.001 | 0.001 | 0.002 | 0.001 | 0.002 |
| meta136 | 1,2-Benzenedicarboxylic acid | 165.018 | 436.903 | 0.002 | 0.001 | 0.002 | 0.002 | 0.002 | 0.001 | 0.002 | 0.003 | 0.001 | 0.002 | 0.002 | 0.002 | 0.001 | 0.001 | 0.001 |
| meta137 |  | 165.033 | 250.848 | 0.025 | 0.022 | 0.020 | 0.024 | 0.016 | 0.018 | 0.011 | 0.021 | 0.023 | 0.031 | 0.023 | 0.015 | 0.020 | 0.018 | 0.019 |
| meta138 | D-(+)-3-Phenyllactic acid | 165.054 | 121.167 | 0.004 | 0.004 | 0.003 | 0.004 | 0.003 | 0.003 | 0.004 | 0.004 | 0.002 | 0.004 | 0.005 | 0.004 | 0.003 | 0.002 | 0.003 |
| meta139 |  | 166.017 | 74.297 | 0.017 | 0.016 | 0.017 | 0.017 | 0.010 | 0.015 | 0.019 | 0.020 | 0.017 | 0.022 | 0.022 | 0.013 | 0.020 | 0.019 | 0.023 |
| meta140 | Uric acid | 167.020 | 310.828 | 0.010 | 0.012 | 0.011 | 0.013 | 0.010 | 0.011 | 0.008 | 0.016 | 0.014 | 0.017 | 0.006 | 0.011 | 0.017 | 0.008 | 0.008 |
| meta141 |  | 167.978 | 61.003 | 0.021 | 0.025 | 0.021 | 0.010 | 0.019 | 0.007 | 0.015 | 0.022 | 0.020 | 0.028 | 0.011 | 0.012 | 0.022 | 0.013 | 0.021 |
| meta142 |  | 168.042 | 468.777 | 0.020 | 0.019 | 0.020 | 0.028 | 0.025 | 0.018 | 0.017 | 0.040 | 0.023 | 0.022 | 0.020 | 0.021 | 0.017 | 0.019 | 0.017 |
| meta143 | Dihydroxyacetone phosphate | 168.989 | 421.594 | 0.004 | 0.003 | 0.003 | 0.005 | 0.004 | 0.003 | 0.003 | 0.004 | 0.004 | 0.004 | 0.004 | 0.004 | 0.005 | 0.003 | 0.003 |
| meta144 |  | 168.990 | 285.067 | 0.011 | 0.008 | 0.008 | 0.013 | 0.012 | 0.007 | 0.012 | 0.010 | 0.007 | 0.009 | 0.010 | 0.010 | 0.010 | 0.007 | 0.009 |
| meta145 |  | 169.061 | 293.543 | 0.003 | 0.003 | 0.002 | 0.003 | 0.003 | 0.002 | 0.003 | 0.003 | 0.002 | 0.003 | 0.003 | 0.002 | 0.003 | 0.002 | 0.003 |
| meta146 |  | 169.097 | 115.936 | 0.011 | 0.009 | 0.011 | 0.003 | 0.001 | 0.002 | 0.033 | 0.022 | 0.015 | 0.006 | 0.005 | 0.015 | 0.007 | 0.002 | 0.004 |
| meta147 |  | 169.122 | 46.614 | 0.007 | 0.006 | 0.007 | 0.009 | 0.015 | 0.008 | 0.014 | 0.007 | 0.006 | 0.006 | 0.007 | 0.004 | 0.004 | 0.007 | 0.005 |
| meta148 |  | 170.045 | 375.042 | 0.012 | 0.011 | 0.011 | 0.011 | 0.015 | 0.007 | 0.011 | 0.014 | 0.009 | 0.013 | 0.014 | 0.011 | 0.011 | 0.008 | 0.011 |
| meta149 | Glycerol 3-phosphate | 171.005 | 421.502 | 0.221 | 0.195 | 0.192 | 0.254 | 0.222 | 0.149 | 0.210 | 0.257 | 0.221 | 0.241 | 0.235 | 0.235 | 0.320 | 0.197 | 0.172 |
| meta150 | Hexanoylglycine | 172.097 | 164.322 | 0.009 | 0.009 | 0.008 | 0.010 | 0.011 | 0.009 | 0.011 | 0.023 | 0.004 | 0.010 | 0.008 | 0.014 | 0.008 | 0.009 | 0.014 |
| meta151 | Acetyl-DL-Leucine | 172.097 | 176.705 | 0.009 | 0.009 | 0.011 | 0.013 | 0.008 | 0.010 | 0.013 | 0.009 | 0.006 | 0.010 | 0.014 | 0.009 | 0.006 | 0.004 | 0.012 |
| meta152 |  | 172.957 | 410.345 | 0.039 | 0.044 | 0.042 | 0.097 | 0.045 | 0.129 | 0.049 | 0.035 | 0.032 | 0.035 | 0.032 | 0.036 | 0.038 | 0.165 | 0.187 |
| meta153 |  | 172.957 | 50.664 | 0.074 | 0.073 | 0.068 | 0.088 | 0.068 | 0.056 | 0.069 | 0.066 | 0.069 | 0.070 | 0.082 | 0.075 | 0.074 | 0.064 | 0.066 |
| meta154 | Dehydroascorbic acid (Oxidized vitamin C) | 173.008 | 157.969 | 0.073 | 0.056 | 0.070 | 0.102 | 0.095 | 0.045 | 0.052 | 0.055 | 0.050 | 0.081 | 0.041 | 0.045 | 0.056 | 0.048 | 0.057 |
| meta155 | Aconitic acid | 173.009 | 422.730 | 0.006 | 0.005 | 0.006 | 0.006 | 0.006 | 0.005 | 0.005 | 0.006 | 0.005 | 0.006 | 0.005 | 0.006 | 0.007 | 0.004 | 0.007 |
| meta156 |  | 173.002 | 364.055 | 0.011 | 0.010 | 0.010 | 0.011 | 0.011 | 0.008 | 0.009 | 0.010 | 0.013 | 0.013 | 0.008 | 0.008 | 0.012 | 0.009 | 0.010 |
| meta157 | Vanillin | 173.025 | 79.374 | 0.002 | 0.002 | 0.002 | 0.026 | 0.023 | 0.018 | 0.023 | 0.020 | 0.025 | 0.001 | 0.001 | 0.001 | 0.001 | 0.001 | 0.025 |
| meta158 | Dihydrouracil | 173.055 | 298.794 | 0.012 | 0.010 | 0.011 | 0.010 | 0.010 | 0.007 | 0.013 | 0.021 | 0.012 | 0.011 | 0.014 | 0.014 | 0.009 | 0.007 | 0.012 |
| meta159 | Suberic acid | 173.081 | 344.141 | 0.002 | 0.001 | 0.001 | 0.002 | 0.002 | 0.001 | 0.002 | 0.002 | 0.002 | 0.002 | 0.001 | 0.002 | 0.002 | 0.002 | 0.001 |
| meta160 | L-Arabitol | 173.117 | 126.835 | 0.003 | 0.003 | 0.004 | 0.003 | 0.004 | 0.003 | 0.002 | 0.004 | 0.003 | 0.002 | 0.006 | 0.003 | 0.002 | 0.003 | 0.003 |
| meta161 | N-Acetyl-L-aspartic acid | 174.040 | 381.746 | 0.016 | 0.013 | 0.012 | 0.016 | 0.015 | 0.014 | 0.011 | 0.014 | 0.011 | 0.014 | 0.016 | 0.013 | 0.017 | 0.018 | 0.009 |
| meta162 | L-Citrulline | 174.087 | 367.952 | 0.002 | 0.002 | 0.002 | 0.003 | 0.003 | 0.002 | 0.002 | 0.003 | 0.002 | 0.002 | 0.002 | 0.002 | 0.002 | 0.002 | 0.002 |
| meta163 |  | 174.937 | 417.837 | 0.017 | 0.013 | 0.012 | 0.021 | 0.015 | 0.011 | 0.014 | 0.016 | 0.010 | 0.017 | 0.013 | 0.015 | 0.012 | 0.010 | 0.012 |
| meta164 | Fosfomycin | 174.953 | 565.648 | 0.019 | 0.018 | 0.031 | 0.035 | 0.035 | 0.028 | 0.035 | 0.037 | 0.033 | 0.034 | 0.035 | 0.037 | 0.036 | 0.043 | 0.043 |
| meta165 | Salicylic acid | 174.977 | 87.470 | 0.005 | 0.004 | 0.005 | 0.008 | 0.003 | 0.006 | 0.005 | 0.003 | 0.002 | 0.004 | 0.005 | 0.003 | 0.015 | 0.005 | 0.006 |
| meta166 | Maleic acid | 175.024 | 440.171 | 0.017 | 0.016 | 0.016 | 0.027 | 0.008 | 0.028 | 0.008 | 0.012 | 0.012 | 0.013 | 0.010 | 0.018 | 0.014 | 0.017 | 0.009 |
| meta167 |  | 175.018 | 47.593 | 0.014 | 0.020 | 0.022 | 0.016 | 0.025 | 0.015 | 0.037 | 0.295 | 0.023 | 0.026 | 0.084 | 0.043 | 0.019 | 0.014 | 0.036 |
| meta168 |  | 175.071 | 330.292 | 0.004 | 0.003 | 0.002 | 0.005 | 0.002 | 0.004 | 0.003 | 0.002 | 0.001 | 0.002 | 0.002 | 0.002 | 0.003 | 0.002 | 0.003 |
| meta169 | 1,2-Diacetylhydrazine | 175.071 | 313.139 | 0.001 | 0.001 | 0.001 | 0.001 | 0.001 | 0.002 | 0.001 | 0.001 | 0.000 | 0.001 | 0.000 | 0.001 | 0.001 | 0.001 | 0.001 |
| meta170 | Indole | 176.071 | 110.850 | 0.006 | 0.006 | 0.006 | 0.001 | 0.001 | 0.001 | 0.016 | 0.011 | 0.009 | 0.004 | 0.004 | 0.011 | 0.004 | 0.001 | 0.002 |
| meta171 | Hippuric acid | 178.050 | 174.063 | 0.007 | 0.008 | 0.006 | 0.009 | 0.003 | 0.009 | 0.005 | 0.011 | 0.008 | 0.009 | 0.011 | 0.004 | 0.003 | 0.006 | 0.005 |
| meta172 | Cyclohexylsulfamate | 178.052 | 69.609 | 0.004 | 0.002 | 0.003 | 0.003 | 0.002 | 0.004 | 0.013 | 0.001 | 0.001 | 0.002 | 0.001 | 0.001 | 0.002 | 0.001 | 0.007 |
| meta173 |  | 178.510 | 282.612 | 0.003 | 0.003 | 0.005 | 0.007 | 0.004 | 0.003 | 0.004 | 0.004 | 0.003 | 0.004 | 0.004 | 0.003 | 0.005 | 0.004 | 0.003 |
| meta174 | D-Fructose | 179.055 | 260.102 | 0.039 | 0.035 | 0.036 | 0.044 | 0.041 | 0.045 | 0.037 | 0.042 | 0.030 | 0.039 | 0.046 | 0.039 | 0.029 | 0.031 | 0.023 |
| meta175 | myo-Inositol | 179.055 | 368.915 | 0.074 | 0.067 | 0.069 | 0.085 | 0.105 | 0.080 | 0.069 | 0.080 | 0.058 | 0.071 | 0.087 | 0.079 | 0.075 | 0.072 | 0.049 |
| meta176 | Acamprosate | 180.032 | 125.149 | 0.014 | 0.012 | 0.012 | 0.014 | 0.017 | 0.009 | 0.013 | 0.017 | 0.008 | 0.011 | 0.010 | 0.006 | 0.008 | 0.016 | 0.010 |
| meta177 | Hydroxyphenyllactic acid | 181.049 | 176.874 | 0.010 | 0.009 | 0.010 | 0.013 | 0.008 | 0.009 | 0.013 | 0.012 | 0.006 | 0.009 | 0.009 | 0.011 | 0.008 | 0.005 | 0.011 |
| meta178 |  | 181.047 | 258.577 | 0.009 | 0.008 | 0.008 | 0.010 | 0.012 | 0.007 | 0.012 | 0.007 | 0.005 | 0.006 | 0.009 | 0.008 | 0.010 | 0.008 | 0.010 |
| meta179 | Saccharin | 181.994 | 56.601 | 0.010 | 0.009 | 0.010 | 0.010 | 0.008 | 0.007 | 0.005 | 0.008 | 0.007 | 0.012 | 0.007 | 0.004 | 0.009 | 0.010 | 0.008 |
| meta180 | L-homocysteic acid | 182.012 | 339.795 | 0.011 | 0.009 | 0.010 | 0.014 | 0.014 | 0.008 | 0.010 | 0.016 | 0.007 | 0.010 | 0.010 | 0.006 | 0.009 | 0.011 | 0.011 |
| meta181 |  | 182.031 | 267.873 | 0.058 | 0.051 | 0.054 | 0.065 | 0.055 | 0.047 | 0.065 | 0.046 | 0.042 | 0.037 | 0.046 | 0.047 | 0.067 | 0.038 | 0.064 |
| meta182 | 4-Pyridoxic acid | 182.045 | 43.294 | 0.024 | 0.023 | 0.025 | 0.037 | 0.018 | 0.029 | 0.040 | 0.023 | 0.016 | 0.013 | 0.018 | 0.013 | 0.013 | 0.011 | 0.012 |
| meta183 |  | 182.056 | 299.351 | 0.004 | 0.003 | 0.003 | 0.002 | 0.003 | 0.002 | 0.005 | 0.007 | 0.004 | 0.004 | 0.005 | 0.006 | 0.003 | 0.001 | 0.003 |
| meta184 | Phosphorylcholine | 182.057 | 361.367 | 0.001 | 0.000 | 0.000 | 0.000 | 0.001 | 0.000 | 0.001 | 0.000 | 0.000 | 0.001 | 0.001 | 0.000 | 0.000 | 0.000 | 0.001 |
| meta185 |  | 182.524 | 174.211 | 0.003 | 0.003 | 0.002 | 0.003 | 0.002 | 0.003 | 0.002 | 0.002 | 0.003 | 0.002 | 0.003 | 0.003 | 0.003 | 0.003 | 0.003 |
| meta186 |  | 182.929 | 157.949 | 0.001 | 0.001 | 0.001 | 0.003 | 0.001 | 0.001 | 0.001 | 0.001 | 0.002 | 0.001 | 0.002 | 0.001 | 0.001 | 0.001 | 0.001 |
| meta187 |  | 182.995 | 25.711 | 0.018 | 0.019 | 0.018 | 0.027 | 0.026 | 0.018 | 0.024 | 0.022 | 0.026 | 0.021 | 0.019 | 0.019 | 0.021 | 0.019 | 0.020 |
| meta188 | IS | 183.082 | 174.324 | 1.000 | 1.000 | 1.000 | 1.000 | 1.000 | 1.000 | 1.000 | 1.000 | 1.000 | 1.000 | 1.000 | 1.000 | 1.000 | 1.000 | 1.000 |
| meta189 |  | 184.071 | 250.882 | 0.014 | 0.013 | 0.012 | 0.001 | 0.004 | 0.001 | 0.029 | 0.029 | 0.025 | 0.011 | 0.006 | 0.015 | 0.014 | 0.002 | 0.005 |
| meta190 |  | 185.000 | 98.073 | 0.014 | 0.017 | 0.019 | 0.006 | 0.013 | 0.004 | 0.037 | 0.027 | 0.022 | 0.013 | 0.012 | 0.008 | 0.021 | 0.005 | 0.014 |
| meta191 |  | 185.021 | 278.076 | 0.071 | 0.058 | 0.055 | 0.056 | 0.096 | 0.035 | 0.053 | 0.079 | 0.026 | 0.055 | 0.098 | 0.088 | 0.030 | 0.040 | 0.064 |
| meta192 |  | 185.153 | 45.963 | 0.013 | 0.008 | 0.008 | 0.016 | 0.014 | 0.006 | 0.009 | 0.008 | 0.008 | 0.008 | 0.009 | 0.007 | 0.007 | 0.007 | 0.011 |
| meta193 |  | 186.471 | 214.421 | 0.005 | 0.004 | 0.004 | 0.006 | 0.004 | 0.005 | 0.004 | 0.004 | 0.001 | 0.003 | 0.003 | 0.003 | 0.003 | 0.002 | 0.004 |
| meta194 | 3-(3-Hydroxyphenyl)propanoic acid | 187.036 | 45.188 | 0.002 | 0.004 | 0.003 | 0.007 | 0.004 | 0.004 | 0.006 | 0.004 | 0.003 | 0.002 | 0.003 | 0.001 | 0.004 | 0.003 | 0.003 |
| meta195 |  | 187.042 | 213.870 | 1.749 | 1.595 | 1.630 | 2.236 | 1.651 | 1.836 | 1.680 | 1.531 | 0.614 | 1.368 | 1.114 | 1.188 | 1.915 | 0.894 | 1.624 |
| meta196 | 4-Methoxyphenylacetic acid | 187.036 | 113.554 | 0.006 | 0.006 | 0.005 | 0.006 | 0.006 | 0.003 | 0.004 | 0.037 | 0.004 | 0.009 | 0.041 | 0.026 | 0.005 | 0.002 | 0.016 |
| meta197 | alpha-N-Acetyl-L-glutamine | 187.071 | 293.631 | 0.033 | 0.029 | 0.026 | 0.032 | 0.031 | 0.026 | 0.030 | 0.029 | 0.020 | 0.032 | 0.035 | 0.024 | 0.033 | 0.020 | 0.029 |
| meta198 | Azelaic acid | 187.096 | 327.941 | 0.003 | 0.003 | 0.003 | 0.004 | 0.004 | 0.003 | 0.003 | 0.004 | 0.003 | 0.003 | 0.003 | 0.003 | 0.003 | 0.003 | 0.003 |
| meta199 | N6-Acetyl-L-lysine | 187.108 | 362.643 | 0.005 | 0.004 | 0.004 | 0.005 | 0.005 | 0.003 | 0.005 | 0.005 | 0.004 | 0.006 | 0.005 | 0.005 | 0.005 | 0.004 | 0.003 |
| meta200 | Glycyl-L-leucine | 187.107 | 266.423 | 0.002 | 0.002 | 0.002 | 0.003 | 0.002 | 0.003 | 0.002 | 0.001 | 0.001 | 0.001 | 0.001 | 0.001 | 0.002 | 0.001 | 0.002 |
| meta201 | 2-Hydroxyadenine | 187.999 | 66.358 | 0.002 | 0.002 | 0.002 | 0.049 | 0.046 | 0.035 | 0.040 | 0.003 | 0.002 | 0.003 | 0.003 | 0.025 | 0.002 | 0.038 | 0.002 |
| meta202 | N-Acetyl-L-glutamate | 188.056 | 375.042 | 0.110 | 0.086 | 0.082 | 0.099 | 0.130 | 0.059 | 0.100 | 0.118 | 0.069 | 0.111 | 0.129 | 0.094 | 0.085 | 0.059 | 0.089 |
| meta203 | N-Acetyl-D-phenylalanine | 188.074 | 293.782 | 0.003 | 0.002 | 0.002 | 0.002 | 0.003 | 0.002 | 0.003 | 0.002 | 0.001 | 0.002 | 0.003 | 0.002 | 0.003 | 0.002 | 0.002 |
| meta204 |  | 188.092 | 176.101 | 0.009 | 0.009 | 0.014 | 0.016 | 0.010 | 0.010 | 0.016 | 0.011 | 0.004 | 0.007 | 0.011 | 0.014 | 0.007 | 0.007 | 0.013 |
| meta205 |  | 188.092 | 253.885 | 0.006 | 0.005 | 0.006 | 0.005 | 0.006 | 0.005 | 0.005 | 0.006 | 0.002 | 0.004 | 0.006 | 0.005 | 0.004 | 0.004 | 0.005 |
| meta206 |  | 188.092 | 223.486 | 0.002 | 0.002 | 0.002 | 0.002 | 0.003 | 0.002 | 0.002 | 0.002 | 0.002 | 0.002 | 0.002 | 0.002 | 0.001 | 0.001 | 0.002 |
| meta207 |  | 188.427 | 86.007 | 0.002 | 0.002 | 0.002 | 0.002 | 0.003 | 0.002 | 0.003 | 0.002 | 0.001 | 0.002 | 0.002 | 0.002 | 0.002 | 0.001 | 0.002 |
| meta208 |  | 188.938 | 318.126 | 0.067 | 0.058 | 0.076 | 0.077 | 0.065 | 0.081 | 0.071 | 0.061 | 0.084 | 0.063 | 0.062 | 0.061 | 0.081 | 0.087 | 0.060 |
| meta209 |  | 188.938 | 50.052 | 0.010 | 0.009 | 0.009 | 0.011 | 0.008 | 0.008 | 0.007 | 0.007 | 0.009 | 0.008 | 0.007 | 0.007 | 0.009 | 0.008 | 0.008 |
| meta210 | Phosphoenolpyruvate | 188.961 | 413.533 | 0.010 | 0.008 | 0.008 | 0.012 | 0.009 | 0.006 | 0.007 | 0.013 | 0.006 | 0.010 | 0.010 | 0.010 | 0.007 | 0.006 | 0.007 |
| meta211 |  | 189.004 | 85.394 | 0.698 | 0.662 | 0.676 | 0.980 | 0.991 | 0.691 | 0.915 | 0.681 | 0.546 | 0.839 | 0.726 | 0.516 | 0.658 | 0.448 | 0.688 |
| meta212 | 3-Methyl-2-oxopentanoate | 189.076 | 367.100 | 0.001 | 0.001 | 0.001 | 0.001 | 0.001 | 0.001 | 0.001 | 0.001 | 0.001 | 0.001 | 0.001 | 0.001 | 0.001 | 0.001 | 0.001 |
| meta213 |  | 189.087 | 308.929 | 0.004 | 0.003 | 0.003 | 0.006 | 0.004 | 0.004 | 0.004 | 0.003 | 0.002 | 0.004 | 0.003 | 0.003 | 0.005 | 0.003 | 0.004 |
| meta214 |  | 190.017 | 196.571 | 0.045 | 0.042 | 0.042 | 0.055 | 0.011 | 0.046 | 0.014 | 0.038 | 0.014 | 0.041 | 0.066 | 0.039 | 0.022 | 0.025 | 0.085 |
| meta215 | N-Acetyl-DL-methionine | 190.053 | 182.047 | 0.019 | 0.018 | 0.018 | 0.015 | 0.011 | 0.025 | 0.013 | 0.013 | 0.006 | 0.021 | 0.022 | 0.015 | 0.026 | 0.022 | 0.020 |
| meta216 | 3-Methylhistidine | 190.060 | 375.042 | 0.001 | 0.001 | 0.001 | 0.001 | 0.001 | 0.001 | 0.001 | 0.001 | 0.001 | 0.001 | 0.001 | 0.001 | 0.001 | 0.001 | 0.001 |
| meta217 | Citric acid | 191.018 | 367.750 | 0.020 | 0.023 | 0.023 | 0.026 | 0.023 | 0.044 | 0.019 | 0.023 | 0.034 | 0.033 | 0.018 | 0.019 | 0.021 | 0.017 | 0.015 |
| meta218 | D-Galactarate | 191.019 | 197.451 | 0.017 | 0.015 | 0.017 | 0.021 | 0.015 | 0.018 | 0.012 | 0.013 | 0.012 | 0.018 | 0.014 | 0.011 | 0.015 | 0.014 | 0.017 |
| meta219 | Citrate | 191.019 | 485.272 | 0.076 | 0.067 | 0.041 | 0.050 | 0.055 | 0.042 | 0.043 | 0.099 | 0.033 | 0.052 | 0.030 | 0.045 | 0.060 | 0.041 | 0.039 |
| meta220 | N-Acetylaspartate | 191.066 | 339.180 | 0.004 | 0.004 | 0.004 | 0.006 | 0.003 | 0.005 | 0.004 | 0.003 | 0.002 | 0.004 | 0.003 | 0.004 | 0.004 | 0.003 | 0.004 |
| meta221 |  | 191.072 | 103.449 | 0.004 | 0.003 | 0.003 | 0.003 | 0.004 | 0.002 | 0.005 | 0.002 | 0.001 | 0.002 | 0.004 | 0.002 | 0.003 | 0.003 | 0.002 |
| meta222 |  | 191.179 | 40.251 | 0.005 | 0.006 | 0.006 | 0.006 | 0.008 | 0.006 | 0.007 | 0.006 | 0.005 | 0.007 | 0.008 | 0.008 | 0.003 | 0.007 | 0.007 |
| meta223 |  | 192.033 | 89.997 | 0.057 | 0.052 | 0.063 | 0.085 | 0.075 | 0.050 | 0.040 | 0.073 | 0.042 | 0.074 | 0.052 | 0.030 | 0.053 | 0.065 | 0.084 |
| meta224 | Phenylacetylglycine | 192.065 | 167.634 | 0.005 | 0.005 | 0.005 | 0.008 | 0.005 | 0.005 | 0.008 | 0.002 | 0.003 | 0.006 | 0.004 | 0.009 | 0.006 | 0.005 | 0.008 |
| meta225 |  | 192.956 | 288.020 | 0.005 | 0.004 | 0.005 | 0.007 | 0.006 | 0.004 | 0.005 | 0.006 | 0.006 | 0.005 | 0.005 | 0.005 | 0.006 | 0.005 | 0.005 |
| meta226 |  | 192.997 | 214.834 | 0.005 | 0.008 | 0.008 | 0.010 | 0.004 | 0.008 | 0.007 | 0.006 | 0.005 | 0.005 | 0.007 | 0.004 | 0.008 | 0.006 | 0.008 |
| meta227 | D-galacturonic acid | 193.034 | 371.817 | 0.027 | 0.025 | 0.024 | 0.029 | 0.024 | 0.020 | 0.023 | 0.036 | 0.025 | 0.028 | 0.031 | 0.036 | 0.029 | 0.023 | 0.022 |
| meta228 |  | 193.420 | 64.345 | 0.003 | 0.003 | 0.004 | 0.001 | 0.001 | 0.002 | 0.002 | 0.006 | 0.003 | 0.003 | 0.005 | 0.002 | 0.002 | 0.002 | 0.004 |
| meta229 | Indoxyl sulfate | 193.994 | 44.538 | 0.006 | 0.006 | 0.006 | 0.001 | 0.001 | 0.000 | 0.005 | 0.006 | 0.005 | 0.005 | 0.002 | 0.001 | 0.003 | 0.001 | 0.004 |
| meta230 |  | 194.012 | 64.526 | 1.668 | 1.540 | 1.571 | 0.575 | 0.204 | 0.527 | 0.778 | 2.697 | 1.385 | 2.281 | 1.803 | 1.117 | 1.492 | 0.562 | 1.664 |
| meta231 | L-dihydroorotate | 194.986 | 155.341 | 0.001 | 0.001 | 0.002 | 0.002 | 0.002 | 0.001 | 0.002 | 0.001 | 0.001 | 0.001 | 0.001 | 0.002 | 0.002 | 0.002 | 0.001 |
| meta232 | Galactonic acid | 195.050 | 343.126 | 0.249 | 0.196 | 0.193 | 0.291 | 0.220 | 0.192 | 0.248 | 0.298 | 0.177 | 0.261 | 0.190 | 0.230 | 0.286 | 0.134 | 0.170 |
| meta233 |  | 196.027 | 333.782 | 0.030 | 0.025 | 0.025 | 0.034 | 0.030 | 0.022 | 0.021 | 0.036 | 0.020 | 0.032 | 0.024 | 0.018 | 0.025 | 0.023 | 0.024 |
| meta234 | N-Acetyl-L-Histidine | 196.071 | 205.147 | 0.000 | 0.000 | 0.000 | 0.000 | 0.000 | 0.000 | 0.000 | 0.002 | 0.000 | 0.000 | 0.001 | 0.001 | 0.000 | 0.000 | 0.000 |
| meta235 |  | 196.925 | 288.351 | 0.013 | 0.011 | 0.013 | 0.016 | 0.014 | 0.012 | 0.015 | 0.015 | 0.014 | 0.014 | 0.013 | 0.013 | 0.016 | 0.013 | 0.013 |
| meta236 | 5,6,7,8-tetrahydro-2-Naphthoic Acid | 197.054 | 343.471 | 0.005 | 0.004 | 0.004 | 0.005 | 0.004 | 0.003 | 0.004 | 0.005 | 0.004 | 0.004 | 0.004 | 0.004 | 0.004 | 0.003 | 0.004 |
| meta237 | O-Phospho-L-homoserine | 198.016 | 396.748 | 0.002 | 0.002 | 0.002 | 0.003 | 0.002 | 0.002 | 0.003 | 0.002 | 0.001 | 0.002 | 0.003 | 0.002 | 0.002 | 0.001 | 0.002 |
| meta238 |  | 198.032 | 223.203 | 0.074 | 0.064 | 0.068 | 0.071 | 0.070 | 0.084 | 0.072 | 0.073 | 0.081 | 0.079 | 0.069 | 0.073 | 0.080 | 0.070 | 0.070 |
| meta239 |  | 198.933 | 86.286 | 0.001 | 0.001 | 0.001 | 0.002 | 0.002 | 0.001 | 0.002 | 0.001 | 0.001 | 0.001 | 0.001 | 0.001 | 0.001 | 0.001 | 0.001 |
| meta240 |  | 198.953 | 160.602 | 0.012 | 0.012 | 0.014 | 0.020 | 0.018 | 0.009 | 0.009 | 0.005 | 0.015 | 0.007 | 0.011 | 0.012 | 0.011 | 0.010 | 0.017 |
| meta241 | D-Erythrose 4-phosphate | 199.000 | 427.628 | 0.007 | 0.005 | 0.006 | 0.008 | 0.013 | 0.006 | 0.016 | 0.004 | 0.006 | 0.005 | 0.006 | 0.005 | 0.011 | 0.006 | 0.006 |
| meta242 | Gulonolactone | 199.019 | 396.772 | 0.007 | 0.006 | 0.006 | 0.000 | 0.006 | 0.004 | 0.000 | 0.006 | 0.005 | 0.000 | 0.006 | 0.005 | 0.005 | 0.005 | 0.006 |
| meta243 | Dodecanoic acid | 199.169 | 45.919 | 0.069 | 0.092 | 0.091 | 0.141 | 0.091 | 0.120 | 0.085 | 0.087 | 0.080 | 0.085 | 0.073 | 0.100 | 0.084 | 0.117 | 0.060 |
| meta244 |  | 199.987 | 25.492 | 0.016 | 0.018 | 0.015 | 0.016 | 0.014 | 0.013 | 0.016 | 0.021 | 0.020 | 0.033 | 0.023 | 0.009 | 0.021 | 0.020 | 0.016 |
| meta245 | O-Phosphoethanolamine | 200.032 | 366.267 | 0.004 | 0.004 | 0.004 | 0.006 | 0.005 | 0.003 | 0.005 | 0.004 | 0.002 | 0.005 | 0.006 | 0.004 | 0.004 | 0.003 | 0.004 |
| meta246 |  | 200.055 | 150.853 | 0.113 | 0.099 | 0.098 | 0.115 | 0.149 | 0.082 | 0.174 | 0.114 | 0.060 | 0.050 | 0.073 | 0.100 | 0.071 | 0.081 | 0.081 |
| meta247 |  | 201.057 | 218.028 | 0.058 | 0.053 | 0.057 | 0.043 | 0.095 | 0.034 | 0.093 | 0.060 | 0.019 | 0.024 | 0.034 | 0.040 | 0.068 | 0.052 | 0.047 |
| meta248 |  | 201.112 | 191.077 | 0.001 | 0.001 | 0.001 | 0.001 | 0.001 | 0.001 | 0.002 | 0.002 | 0.001 | 0.001 | 0.001 | 0.001 | 0.001 | 0.001 | 0.001 |
| meta249 | Sebacic acid | 201.112 | 306.734 | 0.001 | 0.001 | 0.001 | 0.001 | 0.002 | 0.001 | 0.001 | 0.001 | 0.001 | 0.001 | 0.001 | 0.001 | 0.001 | 0.002 | 0.001 |
| meta250 |  | 201.112 | 63.737 | 0.009 | 0.008 | 0.008 | 0.011 | 0.012 | 0.007 | 0.008 | 0.010 | 0.007 | 0.009 | 0.008 | 0.008 | 0.008 | 0.008 | 0.010 |
| meta251 |  | 203.066 | 361.373 | 0.010 | 0.008 | 0.012 | 0.009 | 0.012 | 0.010 | 0.013 | 0.018 | 0.008 | 0.008 | 0.014 | 0.008 | 0.010 | 0.008 | 0.008 |
| meta252 | L-Tryptophan | 203.081 | 238.436 | 0.011 | 0.009 | 0.011 | 0.013 | 0.011 | 0.011 | 0.011 | 0.013 | 0.009 | 0.013 | 0.011 | 0.012 | 0.010 | 0.008 | 0.010 |
| meta253 | D-Tryptophan | 203.081 | 109.229 | 0.009 | 0.008 | 0.007 | 0.002 | 0.001 | 0.001 | 0.026 | 0.016 | 0.011 | 0.005 | 0.004 | 0.013 | 0.006 | 0.002 | 0.003 |
| meta254 |  | 204.970 | 318.126 | 0.031 | 0.026 | 0.031 | 0.034 | 0.030 | 0.036 | 0.030 | 0.026 | 0.033 | 0.028 | 0.029 | 0.025 | 0.035 | 0.037 | 0.027 |
| meta255 |  | 205.064 | 276.114 | 0.006 | 0.006 | 0.006 | 0.008 | 0.006 | 0.006 | 0.009 | 0.005 | 0.004 | 0.004 | 0.005 | 0.006 | 0.009 | 0.005 | 0.007 |
| meta256 | 5-Methoxyindoleacetate | 205.070 | 392.723 | 0.003 | 0.003 | 0.002 | 0.002 | 0.002 | 0.002 | 0.002 | 0.004 | 0.003 | 0.004 | 0.002 | 0.002 | 0.002 | 0.002 | 0.001 |
| meta257 |  | 205.072 | 329.753 | 0.002 | 0.003 | 0.002 | 0.003 | 0.002 | 0.002 | 0.003 | 0.003 | 0.002 | 0.003 | 0.002 | 0.002 | 0.003 | 0.002 | 0.003 |
| meta258 | N-Acetyl-L-phenylalanine | 206.081 | 161.230 | 0.011 | 0.008 | 0.010 | 0.011 | 0.008 | 0.009 | 0.009 | 0.013 | 0.009 | 0.013 | 0.011 | 0.011 | 0.005 | 0.003 | 0.009 |
| meta259 |  | 206.948 | 318.133 | 0.002 | 0.002 | 0.002 | 0.002 | 0.002 | 0.002 | 0.002 | 0.002 | 0.003 | 0.002 | 0.002 | 0.002 | 0.002 | 0.003 | 0.002 |
| meta260 | Mevalonic acid | 207.086 | 141.578 | 0.000 | 0.000 | 0.000 | 0.001 | 0.000 | 0.000 | 0.000 | 0.001 | 0.000 | 0.002 | 0.002 | 0.000 | 0.000 | 0.000 | 0.000 |
| meta261 |  | 208.027 | 137.985 | 0.018 | 0.015 | 0.015 | 0.008 | 0.041 | 0.010 | 0.019 | 0.021 | 0.010 | 0.020 | 0.010 | 0.007 | 0.015 | 0.015 | 0.013 |
| meta262 |  | 208.985 | 157.972 | 0.019 | 0.022 | 0.021 | 0.030 | 0.061 | 0.048 | 0.014 | 0.016 | 0.017 | 0.021 | 0.019 | 0.013 | 0.016 | 0.017 | 0.017 |
| meta263 |  | 210.006 | 71.893 | 0.012 | 0.013 | 0.012 | 0.010 | 0.011 | 0.009 | 0.006 | 0.033 | 0.013 | 0.017 | 0.023 | 0.010 | 0.009 | 0.009 | 0.014 |
| meta264 |  | 210.043 | 119.755 | 0.012 | 0.011 | 0.011 | 0.018 | 0.019 | 0.012 | 0.007 | 0.005 | 0.005 | 0.007 | 0.009 | 0.004 | 0.009 | 0.010 | 0.004 |
| meta265 |  | 210.985 | 85.394 | 0.078 | 0.074 | 0.078 | 0.104 | 0.103 | 0.076 | 0.100 | 0.066 | 0.064 | 0.071 | 0.079 | 0.040 | 0.077 | 0.065 | 0.089 |
| meta266 | Phosphocreatine | 211.034 | 92.395 | 0.011 | 0.008 | 0.011 | 0.098 | 0.008 | 0.078 | 0.011 | 0.034 | 0.011 | 0.008 | 0.033 | 0.027 | 0.008 | 0.067 | 0.010 |
| meta267 |  | 211.034 | 43.267 | 0.025 | 0.021 | 0.020 | 0.004 | 0.003 | 0.004 | 0.043 | 0.048 | 0.024 | 0.011 | 0.021 | 0.034 | 0.013 | 0.003 | 0.009 |
| meta268 |  | 211.107 | 157.947 | 0.006 | 0.004 | 0.004 | 0.008 | 0.006 | 0.005 | 0.007 | 0.004 | 0.008 | 0.004 | 0.005 | 0.004 | 0.005 | 0.004 | 0.003 |
| meta269 |  | 212.032 | 287.061 | 0.003 | 0.002 | 0.003 | 0.004 | 0.003 | 0.003 | 0.004 | 0.004 | 0.002 | 0.003 | 0.003 | 0.003 | 0.003 | 0.002 | 0.003 |
| meta270 |  | 212.956 | 287.701 | 0.011 | 0.009 | 0.009 | 0.013 | 0.012 | 0.010 | 0.012 | 0.012 | 0.012 | 0.011 | 0.010 | 0.011 | 0.012 | 0.011 | 0.010 |
| meta271 |  | 212.961 | 421.594 | 0.028 | 0.024 | 0.022 | 0.032 | 0.026 | 0.015 | 0.025 | 0.028 | 0.023 | 0.025 | 0.029 | 0.025 | 0.029 | 0.023 | 0.024 |
| meta272 |  | 212.979 | 433.520 | 0.008 | 0.006 | 0.005 | 0.006 | 0.007 | 0.004 | 0.006 | 0.008 | 0.004 | 0.008 | 0.008 | 0.006 | 0.005 | 0.004 | 0.007 |
| meta273 | m-Chlorohippuric acid | 213.016 | 101.796 | 0.029 | 0.025 | 0.032 | 0.076 | 0.065 | 0.052 | 0.081 | 0.049 | 0.026 | 0.028 | 0.059 | 0.023 | 0.054 | 0.036 | 0.063 |
| meta274 | 1-Deoxy-D-xylulose 5-phosphate | 213.015 | 313.052 | 0.003 | 0.003 | 0.003 | 0.004 | 0.004 | 0.002 | 0.003 | 0.004 | 0.003 | 0.004 | 0.003 | 0.004 | 0.003 | 0.003 | 0.003 |
| meta275 |  | 213.050 | 73.763 | 0.017 | 0.016 | 0.017 | 0.001 | 0.005 | 0.001 | 0.012 | 0.107 | 0.019 | 0.013 | 0.037 | 0.033 | 0.010 | 0.007 | 0.013 |
| meta276 |  | 213.112 | 116.362 | 0.041 | 0.038 | 0.037 | 0.049 | 0.038 | 0.055 | 0.033 | 0.024 | 0.025 | 0.027 | 0.058 | 0.041 | 0.041 | 0.026 | 0.027 |
| meta277 | Tridecanoic acid (Tridecylic acid) | 213.184 | 44.539 | 0.011 | 0.009 | 0.010 | 0.017 | 0.018 | 0.011 | 0.018 | 0.013 | 0.012 | 0.012 | 0.012 | 0.012 | 0.018 | 0.018 | 0.009 |
| meta278 | sn-Glycerol 3-phosphoethanolamine | 214.048 | 371.516 | 0.196 | 0.178 | 0.157 | 0.230 | 0.204 | 0.137 | 0.224 | 0.223 | 0.122 | 0.225 | 0.255 | 0.159 | 0.165 | 0.125 | 0.185 |
| meta279 | Pectin (Galacturonic acid) | 215.013 | 157.976 | 0.004 | 0.004 | 0.004 | 0.012 | 0.006 | 0.003 | 0.006 | 0.004 | 0.004 | 0.003 | 0.004 | 0.004 | 0.004 | 0.004 | 0.005 |
| meta280 |  | 215.031 | 278.106 | 0.046 | 0.038 | 0.044 | 0.049 | 0.043 | 0.036 | 0.045 | 0.044 | 0.033 | 0.037 | 0.041 | 0.042 | 0.043 | 0.036 | 0.036 |
| meta281 | 3-Hydroxydodecanoic acid | 215.164 | 47.331 | 0.004 | 0.003 | 0.003 | 0.005 | 0.002 | 0.004 | 0.001 | 0.004 | 0.001 | 0.006 | 0.003 | 0.003 | 0.005 | 0.004 | 0.001 |
| meta282 |  | 216.097 | 338.839 | 0.010 | 0.009 | 0.009 | 0.015 | 0.009 | 0.008 | 0.011 | 0.007 | 0.007 | 0.008 | 0.007 | 0.007 | 0.013 | 0.007 | 0.010 |
| meta283 |  | 216.098 | 302.159 | 0.002 | 0.002 | 0.002 | 0.002 | 0.002 | 0.001 | 0.002 | 0.003 | 0.003 | 0.002 | 0.003 | 0.003 | 0.001 | 0.002 | 0.002 |
| meta284 |  | 216.943 | 85.540 | 0.002 | 0.002 | 0.002 | 0.003 | 0.003 | 0.002 | 0.002 | 0.002 | 0.001 | 0.002 | 0.003 | 0.001 | 0.002 | 0.001 | 0.001 |
| meta285 |  | 217.001 | 290.302 | 0.035 | 0.029 | 0.031 | 0.043 | 0.008 | 0.027 | 0.032 | 0.033 | 0.030 | 0.030 | 0.029 | 0.028 | 0.032 | 0.031 | 0.032 |
| meta286 |  | 217.082 | 280.626 | 0.013 | 0.009 | 0.010 | 0.016 | 0.014 | 0.014 | 0.015 | 0.010 | 0.004 | 0.007 | 0.007 | 0.006 | 0.011 | 0.006 | 0.009 |
| meta287 | 5-L-Glutamyl-L-alanine | 217.081 | 427.896 | 0.002 | 0.002 | 0.002 | 0.003 | 0.003 | 0.002 | 0.002 | 0.003 | 0.002 | 0.002 | 0.002 | 0.002 | 0.002 | 0.002 | 0.002 |
| meta288 |  | 217.118 | 248.616 | 0.003 | 0.004 | 0.004 | 0.008 | 0.004 | 0.005 | 0.004 | 0.001 | 0.001 | 0.001 | 0.001 | 0.002 | 0.003 | 0.002 | 0.003 |
| meta289 |  | 218.011 | 48.132 | 0.029 | 0.019 | 0.029 | 0.023 | 0.015 | 0.019 | 0.008 | 0.041 | 0.014 | 0.043 | 0.025 | 0.009 | 0.020 | 0.014 | 0.030 |
| meta290 |  | 218.009 | 333.956 | 0.015 | 0.015 | 0.014 | 0.018 | 0.016 | 0.013 | 0.011 | 0.019 | 0.012 | 0.016 | 0.013 | 0.009 | 0.014 | 0.013 | 0.012 |
| meta291 |  | 218.066 | 390.124 | 0.027 | 0.024 | 0.022 | 0.030 | 0.041 | 0.023 | 0.014 | 0.019 | 0.015 | 0.019 | 0.021 | 0.032 | 0.046 | 0.034 | 0.017 |
| meta292 | Pantothenate | 218.102 | 254.068 | 0.035 | 0.033 | 0.032 | 0.031 | 0.050 | 0.025 | 0.038 | 0.037 | 0.025 | 0.028 | 0.040 | 0.035 | 0.018 | 0.021 | 0.037 |
| meta293 |  | 218.995 | 33.385 | 0.006 | 0.005 | 0.005 | 0.007 | 0.001 | 0.008 | 0.001 | 0.006 | 0.009 | 0.012 | 0.011 | 0.002 | 0.001 | 0.005 | 0.001 |
| meta294 | BHT | 219.174 | 32.288 | 0.002 | 0.002 | 0.002 | 0.004 | 0.003 | 0.003 | 0.003 | 0.003 | 0.003 | 0.003 | 0.004 | 0.003 | 0.002 | 0.003 | 0.003 |
| meta295 | N-Acetylmannosamine | 220.081 | 330.812 | 0.003 | 0.003 | 0.003 | 0.003 | 0.005 | 0.002 | 0.002 | 0.002 | 0.003 | 0.003 | 0.002 | 0.002 | 0.005 | 0.004 | 0.002 |
| meta296 | L-Carnitine | 220.118 | 329.999 | 0.003 | 0.003 | 0.002 | 0.004 | 0.004 | 0.003 | 0.003 | 0.003 | 0.002 | 0.003 | 0.003 | 0.003 | 0.003 | 0.002 | 0.003 |
| meta297 | 3-Prenyl-4-Hydroxyacetophenone | 220.136 | 26.590 | 0.002 | 0.002 | 0.002 | 0.001 | 0.003 | 0.002 | 0.003 | 0.002 | 0.002 | 0.003 | 0.003 | 0.003 | 0.002 | 0.002 | 0.002 |
| meta298 |  | 220.886 | 291.298 | 0.008 | 0.008 | 0.011 | 0.011 | 0.009 | 0.009 | 0.009 | 0.011 | 0.010 | 0.009 | 0.009 | 0.011 | 0.013 | 0.010 | 0.009 |
| meta299 |  | 221.011 | 214.410 | 0.029 | 0.023 | 0.022 | 0.037 | 0.031 | 0.030 | 0.031 | 0.025 | 0.007 | 0.021 | 0.016 | 0.018 | 0.025 | 0.012 | 0.027 |
| meta300 |  | 221.004 | 44.321 | 0.015 | 0.014 | 0.018 | 0.004 | 0.003 | 0.000 | 0.008 | 0.017 | 0.009 | 0.023 | 0.007 | 0.006 | 0.007 | 0.003 | 0.010 |
| meta301 |  | 221.065 | 368.940 | 0.019 | 0.018 | 0.017 | 0.019 | 0.023 | 0.011 | 0.017 | 0.019 | 0.013 | 0.019 | 0.020 | 0.026 | 0.016 | 0.017 | 0.009 |
| meta302 | N-Acetyl-D-glucosamine | 221.091 | 245.526 | 0.001 | 0.001 | 0.001 | 0.002 | 0.001 | 0.002 | 0.001 | 0.001 | 0.000 | 0.001 | 0.001 | 0.001 | 0.001 | 0.001 | 0.001 |
| meta303 |  | 221.153 | 45.209 | 0.009 | 0.009 | 0.006 | 0.011 | 0.013 | 0.007 | 0.007 | 0.010 | 0.011 | 0.006 | 0.007 | 0.007 | 0.010 | 0.010 | 0.009 |
| meta304 |  | 221.517 | 34.998 | 0.024 | 0.023 | 0.023 | 0.019 | 0.012 | 0.014 | 0.026 | 0.081 | 0.042 | 0.028 | 0.028 | 0.009 | 0.029 | 0.034 | 0.005 |
| meta305 | N-Acetyl-L-tyrosine | 222.076 | 204.169 | 0.002 | 0.002 | 0.002 | 0.002 | 0.002 | 0.002 | 0.003 | 0.003 | 0.001 | 0.002 | 0.004 | 0.003 | 0.002 | 0.001 | 0.002 |
| meta306 |  | 222.133 | 243.331 | 0.010 | 0.007 | 0.008 | 0.010 | 0.013 | 0.007 | 0.012 | 0.010 | 0.007 | 0.007 | 0.007 | 0.010 | 0.007 | 0.008 | 0.008 |
| meta307 |  | 222.914 | 144.412 | 0.002 | 0.002 | 0.002 | 0.003 | 0.003 | 0.002 | 0.002 | 0.002 | 0.003 | 0.003 | 0.002 | 0.002 | 0.003 | 0.003 | 0.003 |
| meta308 | D-Quinovose | 223.081 | 190.197 | 0.007 | 0.006 | 0.006 | 0.008 | 0.006 | 0.006 | 0.007 | 0.009 | 0.004 | 0.007 | 0.006 | 0.009 | 0.006 | 0.006 | 0.006 |
| meta309 |  | 223.169 | 44.538 | 0.029 | 0.027 | 0.030 | 0.034 | 0.055 | 0.045 | 0.040 | 0.035 | 0.037 | 0.027 | 0.032 | 0.059 | 0.030 | 0.051 | 0.030 |
| meta310 |  | 224.022 | 352.681 | 0.010 | 0.008 | 0.008 | 0.008 | 0.008 | 0.007 | 0.009 | 0.011 | 0.007 | 0.012 | 0.009 | 0.007 | 0.013 | 0.008 | 0.006 |
| meta311 |  | 224.022 | 135.967 | 0.301 | 0.242 | 0.243 | 0.380 | 0.253 | 0.200 | 0.174 | 0.284 | 0.161 | 0.278 | 0.322 | 0.122 | 0.194 | 0.347 | 0.294 |
| meta312 |  | 224.066 | 419.380 | 0.014 | 0.012 | 0.019 | 0.014 | 0.012 | 0.015 | 0.011 | 0.014 | 0.012 | 0.016 | 0.013 | 0.012 | 0.018 | 0.010 | 0.012 |
| meta313 |  | 224.066 | 287.005 | 0.038 | 0.038 | 0.040 | 0.044 | 0.033 | 0.027 | 0.037 | 0.034 | 0.043 | 0.036 | 0.031 | 0.027 | 0.034 | 0.032 | 0.037 |
| meta314 |  | 225.050 | 405.917 | 0.005 | 0.005 | 0.005 | 0.006 | 0.004 | 0.004 | 0.005 | 0.005 | 0.006 | 0.006 | 0.004 | 0.004 | 0.005 | 0.004 | 0.005 |
| meta315 | D-Biotin | 225.070 | 419.445 | 0.001 | 0.001 | 0.001 | 0.001 | 0.001 | 0.001 | 0.001 | 0.002 | 0.001 | 0.001 | 0.001 | 0.001 | 0.001 | 0.001 | 0.001 |
| meta316 | L-Carnosine | 225.098 | 396.676 | 0.000 | 0.001 | 0.000 | 0.001 | 0.000 | 0.000 | 0.000 | 0.001 | 0.000 | 0.001 | 0.000 | 0.000 | 0.000 | 0.000 | 0.001 |
| meta317 | Myristoleic acid | 225.185 | 43.919 | 0.074 | 0.075 | 0.080 | 0.108 | 0.139 | 0.140 | 0.102 | 0.101 | 0.062 | 0.084 | 0.089 | 0.117 | 0.076 | 0.141 | 0.090 |
| meta318 | 3-Indolepropionic acid | 226.026 | 150.234 | 0.004 | 0.006 | 0.004 | 0.005 | 0.005 | 0.004 | 0.006 | 0.010 | 0.009 | 0.003 | 0.006 | 0.013 | 0.004 | 0.003 | 0.005 |
| meta319 |  | 226.037 | 278.027 | 0.006 | 0.005 | 0.005 | 0.003 | 0.015 | 0.003 | 0.010 | 0.005 | 0.002 | 0.005 | 0.001 | 0.003 | 0.006 | 0.004 | 0.003 |
| meta320 | Jasmonic acid | 226.143 | 130.380 | 0.008 | 0.009 | 0.007 | 0.008 | 0.007 | 0.006 | 0.009 | 0.009 | 0.010 | 0.008 | 0.010 | 0.012 | 0.005 | 0.007 | 0.005 |
| meta321 | 2'-Deoxyuridine | 227.066 | 110.180 | 0.007 | 0.006 | 0.006 | 0.009 | 0.006 | 0.007 | 0.007 | 0.010 | 0.007 | 0.007 | 0.008 | 0.005 | 0.005 | 0.004 | 0.007 |
| meta322 |  | 227.066 | 310.457 | 0.061 | 0.064 | 0.068 | 0.073 | 0.058 | 0.045 | 0.068 | 0.064 | 0.072 | 0.067 | 0.052 | 0.045 | 0.060 | 0.052 | 0.067 |
| meta323 | Myristic acid | 227.201 | 44.433 | 0.447 | 0.493 | 0.478 | 0.768 | 0.723 | 0.803 | 0.603 | 0.770 | 0.451 | 0.582 | 0.575 | 0.696 | 0.581 | 0.726 | 0.542 |
| meta324 | Zonisamide | 228.044 | 40.827 | 0.008 | 0.007 | 0.009 | 0.005 | 0.001 | 0.003 | 0.004 | 0.012 | 0.001 | 0.013 | 0.019 | 0.012 | 0.007 | 0.001 | 0.003 |
| meta325 |  | 228.050 | 337.596 | 0.015 | 0.014 | 0.015 | 0.017 | 0.021 | 0.008 | 0.014 | 0.020 | 0.011 | 0.014 | 0.019 | 0.014 | 0.014 | 0.011 | 0.010 |
| meta326 |  | 228.930 | 318.126 | 0.027 | 0.023 | 0.031 | 0.031 | 0.026 | 0.035 | 0.029 | 0.024 | 0.034 | 0.025 | 0.025 | 0.024 | 0.034 | 0.036 | 0.024 |
| meta327 |  | 228.992 | 158.134 | 0.034 | 0.040 | 0.027 | 0.119 | 0.036 | 0.025 | 0.025 | 0.031 | 0.026 | 0.062 | 0.022 | 0.034 | 0.048 | 0.028 | 0.027 |
| meta328 |  | 228.992 | 139.622 | 0.033 | 0.022 | 0.020 | 0.034 | 0.031 | 0.023 | 0.024 | 0.025 | 0.019 | 0.041 | 0.020 | 0.018 | 0.014 | 0.017 | 0.024 |
| meta329 |  | 229.081 | 412.883 | 0.003 | 0.002 | 0.002 | 0.003 | 0.002 | 0.002 | 0.002 | 0.003 | 0.002 | 0.002 | 0.002 | 0.002 | 0.003 | 0.002 | 0.002 |
| meta330 |  | 229.118 | 262.693 | 0.001 | 0.001 | 0.001 | 0.001 | 0.002 | 0.001 | 0.001 | 0.002 | 0.001 | 0.001 | 0.001 | 0.001 | 0.001 | 0.001 | 0.001 |
| meta331 |  | 229.195 | 40.251 | 0.011 | 0.011 | 0.013 | 0.016 | 0.027 | 0.027 | 0.018 | 0.017 | 0.016 | 0.013 | 0.018 | 0.019 | 0.013 | 0.016 | 0.020 |
| meta332 | D-Ribulose 5-phosphate | 230.018 | 361.444 | 0.001 | 0.001 | 0.001 | 0.001 | 0.001 | 0.001 | 0.001 | 0.001 | 0.001 | 0.001 | 0.001 | 0.001 | 0.001 | 0.000 | 0.001 |
| meta333 |  | 230.102 | 355.404 | 0.002 | 0.001 | 0.001 | 0.002 | 0.002 | 0.001 | 0.002 | 0.002 | 0.001 | 0.002 | 0.001 | 0.002 | 0.001 | 0.002 | 0.001 |
| meta334 |  | 230.175 | 50.584 | 0.003 | 0.002 | 0.003 | 0.004 | 0.003 | 0.002 | 0.004 | 0.002 | 0.002 | 0.001 | 0.002 | 0.002 | 0.002 | 0.002 | 0.004 |
| meta335 |  | 230.912 | 288.540 | 0.002 | 0.002 | 0.002 | 0.003 | 0.003 | 0.002 | 0.002 | 0.003 | 0.003 | 0.002 | 0.002 | 0.002 | 0.003 | 0.002 | 0.002 |
| meta336 | 2-keto-D-Gluconic acid | 230.995 | 227.071 | 0.004 | 0.004 | 0.003 | 0.002 | 0.002 | 0.002 | 0.006 | 0.002 | 0.003 | 0.005 | 0.006 | 0.006 | 0.004 | 0.002 | 0.003 |
| meta337 |  | 231.022 | 381.377 | 0.003 | 0.003 | 0.002 | 0.004 | 0.003 | 0.002 | 0.002 | 0.003 | 0.003 | 0.003 | 0.003 | 0.002 | 0.003 | 0.003 | 0.003 |
| meta338 | Sedoheptulose | 231.044 | 95.138 | 0.004 | 0.003 | 0.004 | 0.004 | 0.007 | 0.003 | 0.008 | 0.001 | 0.003 | 0.003 | 0.003 | 0.002 | 0.003 | 0.003 | 0.004 |
| meta339 |  | 231.061 | 382.034 | 0.002 | 0.002 | 0.001 | 0.002 | 0.002 | 0.001 | 0.002 | 0.003 | 0.002 | 0.002 | 0.002 | 0.002 | 0.002 | 0.001 | 0.001 |
| meta340 | Alpha-ketoisovaleric acid | 231.086 | 379.756 | 0.002 | 0.002 | 0.002 | 0.003 | 0.003 | 0.002 | 0.002 | 0.004 | 0.003 | 0.003 | 0.002 | 0.002 | 0.003 | 0.002 | 0.002 |
| meta341 |  | 232.913 | 25.702 | 0.018 | 0.017 | 0.016 | 0.025 | 0.017 | 0.017 | 0.024 | 0.023 | 0.100 | 0.025 | 0.021 | 0.023 | 0.024 | 0.018 | 0.022 |
| meta342 |  | 232.923 | 61.003 | 0.125 | 0.118 | 0.074 | 0.149 | 0.161 | 0.068 | 0.152 | 0.161 | 0.076 | 0.116 | 0.095 | 0.144 | 0.156 | 0.114 | 0.083 |
| meta343 | Perseitol | 233.059 | 215.719 | 0.000 | 0.000 | 0.000 | 0.000 | 0.000 | 0.000 | 0.000 | 0.001 | 0.000 | 0.000 | 0.000 | 0.000 | 0.000 | 0.000 | 0.000 |
| meta344 |  | 233.143 | 25.973 | 0.004 | 0.004 | 0.003 | 0.005 | 0.004 | 0.003 | 0.003 | 0.003 | 0.004 | 0.006 | 0.004 | 0.004 | 0.006 | 0.004 | 0.004 |
| meta345 | Confertifoline | 233.153 | 46.541 | 0.011 | 0.010 | 0.011 | 0.017 | 0.012 | 0.010 | 0.011 | 0.012 | 0.012 | 0.011 | 0.010 | 0.016 | 0.010 | 0.013 | 0.011 |
| meta346 |  | 234.912 | 483.891 | 0.031 | 0.028 | 0.025 | 0.053 | 0.035 | 0.029 | 0.031 | 0.035 | 0.040 | 0.027 | 0.018 | 0.045 | 0.040 | 0.037 | 0.030 |
| meta347 |  | 235.169 | 45.821 | 0.005 | 0.005 | 0.006 | 0.012 | 0.009 | 0.006 | 0.007 | 0.008 | 0.006 | 0.008 | 0.008 | 0.006 | 0.006 | 0.006 | 0.005 |
| meta348 | Biopterin | 236.077 | 234.014 | 0.002 | 0.002 | 0.002 | 0.002 | 0.002 | 0.002 | 0.001 | 0.002 | 0.002 | 0.003 | 0.002 | 0.002 | 0.002 | 0.002 | 0.002 |
| meta349 |  | 236.091 | 154.639 | 0.006 | 0.006 | 0.005 | 0.007 | 0.007 | 0.006 | 0.012 | 0.005 | 0.002 | 0.004 | 0.004 | 0.005 | 0.003 | 0.002 | 0.006 |
| meta350 |  | 236.860 | 289.082 | 0.083 | 0.079 | 0.084 | 0.104 | 0.089 | 0.078 | 0.094 | 0.083 | 0.097 | 0.083 | 0.081 | 0.080 | 0.094 | 0.087 | 0.086 |
| meta351 | L-Gulonic gamma-lactone | 237.060 | 89.945 | 0.012 | 0.017 | 0.012 | 0.011 | 0.021 | 0.008 | 0.021 | 0.017 | 0.012 | 0.012 | 0.021 | 0.013 | 0.018 | 0.024 | 0.009 |
| meta352 |  | 237.079 | 32.506 | 0.004 | 0.004 | 0.004 | 0.004 | 0.004 | 0.003 | 0.004 | 0.003 | 0.003 | 0.001 | 0.005 | 0.004 | 0.005 | 0.003 | 0.003 |
| meta353 | Nitrofurantoin | 238.037 | 169.589 | 0.004 | 0.003 | 0.003 | 0.008 | 0.019 | 0.004 | 0.023 | 0.000 | 0.001 | 0.001 | 0.005 | 0.005 | 0.003 | 0.001 | 0.005 |
| meta354 | Glucosamine | 238.092 | 285.124 | 0.001 | 0.002 | 0.002 | 0.002 | 0.002 | 0.002 | 0.002 | 0.002 | 0.002 | 0.002 | 0.002 | 0.002 | 0.002 | 0.002 | 0.002 |
| meta355 | L-Cystine | 239.014 | 154.672 | 0.001 | 0.001 | 0.001 | 0.004 | 0.004 | 0.003 | 0.003 | 0.003 | 0.001 | 0.004 | 0.003 | 0.001 | 0.004 | 0.001 | 0.004 |
| meta356 | 3-Deoxy-2-keto-6-phosphogluconic acid | 239.102 | 712.869 | 0.004 | 0.003 | 0.004 | 0.001 | 0.001 | 0.003 | 0.004 | 0.003 | 0.003 | 0.004 | 0.003 | 0.003 | 0.004 | 0.000 | 0.004 |
| meta357 |  | 239.164 | 77.835 | 0.008 | 0.007 | 0.009 | 0.008 | 0.014 | 0.013 | 0.012 | 0.020 | 0.006 | 0.008 | 0.006 | 0.007 | 0.007 | 0.008 | 0.006 |
| meta358 |  | 239.200 | 43.276 | 0.007 | 0.009 | 0.007 | 0.011 | 0.009 | 0.009 | 0.009 | 0.012 | 0.009 | 0.011 | 0.008 | 0.012 | 0.008 | 0.010 | 0.008 |
| meta359 |  | 240.014 | 43.279 | 0.022 | 0.017 | 0.018 | 0.004 | 0.004 | 0.002 | 0.003 | 0.161 | 0.020 | 0.019 | 0.078 | 0.061 | 0.018 | 0.001 | 0.018 |
| meta360 |  | 240.042 | 153.336 | 0.012 | 0.012 | 0.013 | 0.011 | 0.012 | 0.013 | 0.014 | 0.013 | 0.013 | 0.013 | 0.012 | 0.014 | 0.012 | 0.009 | 0.012 |
| meta361 |  | 240.097 | 208.620 | 0.030 | 0.022 | 0.025 | 0.026 | 0.033 | 0.018 | 0.027 | 0.024 | 0.021 | 0.031 | 0.018 | 0.031 | 0.033 | 0.021 | 0.015 |
| meta362 |  | 240.869 | 403.898 | 0.004 | 0.004 | 0.004 | 0.004 | 0.004 | 0.004 | 0.005 | 0.005 | 0.003 | 0.004 | 0.005 | 0.004 | 0.004 | 0.003 | 0.004 |
| meta363 | alpha-D-Glucose 1-phosphate | 241.010 | 327.129 | 0.008 | 0.007 | 0.007 | 0.008 | 0.007 | 0.006 | 0.006 | 0.008 | 0.008 | 0.010 | 0.008 | 0.009 | 0.008 | 0.008 | 0.008 |
| meta364 | Lumichrome | 241.072 | 48.174 | 0.025 | 0.024 | 0.024 | 0.036 | 0.044 | 0.051 | 0.062 | 0.028 | 0.023 | 0.026 | 0.048 | 0.034 | 0.037 | 0.034 | 0.050 |
| meta365 | Pentadecanoic Acid | 241.216 | 43.259 | 0.115 | 0.104 | 0.106 | 0.131 | 0.108 | 0.112 | 0.120 | 0.143 | 0.171 | 0.118 | 0.157 | 0.177 | 0.121 | 0.141 | 0.116 |
| meta366 |  | 242.050 | 277.506 | 0.039 | 0.042 | 0.041 | 0.057 | 0.044 | 0.032 | 0.040 | 0.049 | 0.031 | 0.034 | 0.041 | 0.048 | 0.045 | 0.035 | 0.041 |
| meta367 |  | 242.175 | 117.811 | 0.089 | 0.088 | 0.091 | 0.132 | 0.096 | 0.066 | 0.068 | 0.108 | 0.083 | 0.082 | 0.071 | 0.101 | 0.090 | 0.074 | 0.161 |
| meta368 |  | 242.174 | 33.330 | 0.002 | 0.002 | 0.002 | 0.003 | 0.003 | 0.001 | 0.002 | 0.002 | 0.003 | 0.000 | 0.002 | 0.003 | 0.004 | 0.003 | 0.004 |
| meta369 |  | 242.318 | 150.876 | 0.004 | 0.004 | 0.004 | 0.005 | 0.006 | 0.004 | 0.008 | 0.005 | 0.003 | 0.002 | 0.003 | 0.004 | 0.003 | 0.003 | 0.003 |
| meta370 |  | 242.995 | 180.147 | 0.013 | 0.011 | 0.012 | 0.013 | 0.002 | 0.009 | 0.005 | 0.005 | 0.009 | 0.025 | 0.011 | 0.013 | 0.008 | 0.026 | 0.020 |
| meta371 | DL-3,4-Dihydroxymandelic acid | 243.053 | 278.106 | 0.003 | 0.003 | 0.002 | 0.003 | 0.003 | 0.002 | 0.002 | 0.003 | 0.002 | 0.002 | 0.002 | 0.003 | 0.003 | 0.002 | 0.003 |
| meta372 | Pseudouridine | 243.061 | 224.835 | 0.019 | 0.016 | 0.015 | 0.020 | 0.023 | 0.015 | 0.020 | 0.016 | 0.012 | 0.017 | 0.015 | 0.014 | 0.013 | 0.012 | 0.013 |
| meta373 | Helenalin | 243.097 | 403.804 | 0.001 | 0.001 | 0.001 | 0.002 | 0.001 | 0.001 | 0.001 | 0.001 | 0.001 | 0.001 | 0.001 | 0.001 | 0.001 | 0.001 | 0.001 |
| meta374 |  | 243.101 | 100.137 | 0.046 | 0.051 | 0.045 | 0.110 | 0.120 | 0.085 | 0.136 | 0.057 | 0.044 | 0.051 | 0.042 | 0.030 | 0.019 | 0.051 | 0.076 |
| meta375 | alpha-hydroxy myristic acid | 243.195 | 47.234 | 0.013 | 0.011 | 0.013 | 0.019 | 0.012 | 0.015 | 0.013 | 0.015 | 0.008 | 0.014 | 0.015 | 0.014 | 0.015 | 0.020 | 0.013 |
| meta376 |  | 243.195 | 69.018 | 0.008 | 0.008 | 0.008 | 0.013 | 0.011 | 0.010 | 0.010 | 0.010 | 0.006 | 0.008 | 0.010 | 0.008 | 0.010 | 0.013 | 0.007 |
| meta377 | 3-Phosphoserine | 244.021 | 433.806 | 0.004 | 0.004 | 0.004 | 0.004 | 0.005 | 0.003 | 0.003 | 0.004 | 0.003 | 0.003 | 0.004 | 0.003 | 0.004 | 0.003 | 0.004 |
| meta378 |  | 244.081 | 370.910 | 0.010 | 0.008 | 0.008 | 0.007 | 0.013 | 0.004 | 0.004 | 0.005 | 0.003 | 0.010 | 0.006 | 0.005 | 0.007 | 0.009 | 0.004 |
| meta379 |  | 244.154 | 157.949 | 0.001 | 0.001 | 0.001 | 0.001 | 0.002 | 0.001 | 0.002 | 0.002 | 0.001 | 0.002 | 0.001 | 0.004 | 0.002 | 0.001 | 0.001 |
| meta380 |  | 245.042 | 329.679 | 0.210 | 0.175 | 0.150 | 0.208 | 0.223 | 0.151 | 0.179 | 0.218 | 0.157 | 0.267 | 0.210 | 0.144 | 0.153 | 0.141 | 0.193 |
| meta381 |  | 245.117 | 126.809 | 0.009 | 0.009 | 0.009 | 0.011 | 0.009 | 0.008 | 0.019 | 0.016 | 0.009 | 0.012 | 0.007 | 0.003 | 0.007 | 0.007 | 0.010 |
| meta382 | gamma-L-Glutamyl-L-valine | 245.113 | 363.997 | 0.005 | 0.003 | 0.003 | 0.006 | 0.004 | 0.005 | 0.004 | 0.004 | 0.004 | 0.005 | 0.004 | 0.004 | 0.005 | 0.003 | 0.004 |
| meta383 | 12-Oxo-2,3-dinor-10,15-phytodienoic acid | 245.153 | 45.927 | 0.000 | 0.000 | 0.001 | 0.001 | 0.001 | 0.000 | 0.001 | 0.001 | 0.001 | 0.000 | 0.001 | 0.000 | 0.000 | 0.000 | 0.000 |
| meta384 | Floxuridine | 246.060 | 338.477 | 0.002 | 0.002 | 0.002 | 0.003 | 0.004 | 0.001 | 0.003 | 0.004 | 0.002 | 0.002 | 0.003 | 0.003 | 0.003 | 0.002 | 0.002 |
| meta385 | Indoleacrylic acid | 246.078 | 45.141 | 0.001 | 0.001 | 0.001 | 0.001 | 0.001 | 0.001 | 0.001 | 0.001 | 0.001 | 0.001 | 0.002 | 0.001 | 0.001 | 0.001 | 0.002 |
| meta386 |  | 246.097 | 379.429 | 0.011 | 0.010 | 0.010 | 0.013 | 0.018 | 0.009 | 0.006 | 0.009 | 0.006 | 0.010 | 0.009 | 0.011 | 0.015 | 0.012 | 0.008 |
| meta387 |  | 246.980 | 318.126 | 0.027 | 0.021 | 0.027 | 0.028 | 0.025 | 0.030 | 0.025 | 0.022 | 0.028 | 0.025 | 0.024 | 0.022 | 0.030 | 0.033 | 0.022 |
| meta388 |  | 247.012 | 214.458 | 0.020 | 0.015 | 0.015 | 0.021 | 0.017 | 0.016 | 0.018 | 0.016 | 0.008 | 0.016 | 0.012 | 0.014 | 0.015 | 0.009 | 0.014 |
| meta389 |  | 247.092 | 402.528 | 0.003 | 0.003 | 0.003 | 0.004 | 0.003 | 0.004 | 0.004 | 0.003 | 0.003 | 0.003 | 0.003 | 0.004 | 0.004 | 0.001 | 0.002 |
| meta390 | Quadrone | 247.132 | 114.445 | 0.007 | 0.004 | 0.007 | 0.006 | 0.004 | 0.004 | 0.010 | 0.009 | 0.006 | 0.007 | 0.005 | 0.002 | 0.003 | 0.007 | 0.004 |
| meta391 |  | 247.169 | 44.717 | 0.003 | 0.002 | 0.002 | 0.003 | 0.005 | 0.004 | 0.004 | 0.003 | 0.002 | 0.003 | 0.002 | 0.005 | 0.002 | 0.004 | 0.002 |
| meta392 |  | 248.077 | 94.953 | 0.172 | 0.160 | 0.169 | 0.241 | 0.285 | 0.181 | 0.283 | 0.207 | 0.099 | 0.099 | 0.137 | 0.122 | 0.137 | 0.167 | 0.150 |
| meta393 |  | 248.972 | 21.224 | 1.378 | 1.443 | 1.364 | 1.862 | 0.917 | 1.227 | 1.500 | 2.223 | 1.351 | 0.876 | 1.921 | 1.853 | 1.601 | 0.713 | 1.516 |
| meta394 |  | 249.020 | 274.326 | 0.037 | 0.027 | 0.030 | 0.041 | 0.041 | 0.036 | 0.029 | 0.055 | 0.027 | 0.053 | 0.027 | 0.016 | 0.033 | 0.032 | 0.030 |
| meta395 |  | 249.017 | 40.914 | 1.525 | 1.389 | 1.376 | 1.646 | 1.412 | 1.152 | 1.400 | 0.011 | 0.006 | 1.404 | 1.343 | 0.006 | 1.381 | 1.325 | 0.003 |
| meta396 |  | 249.184 | 43.797 | 0.023 | 0.028 | 0.029 | 0.040 | 0.065 | 0.047 | 0.046 | 0.031 | 0.028 | 0.036 | 0.020 | 0.000 | 0.033 | 0.061 | 0.036 |
| meta397 |  | 250.056 | 394.125 | 0.010 | 0.009 | 0.007 | 0.011 | 0.013 | 0.006 | 0.007 | 0.006 | 0.008 | 0.007 | 0.008 | 0.009 | 0.010 | 0.008 | 0.008 |
| meta398 | 5'-Deoxyadenosine | 250.092 | 398.681 | 0.007 | 0.007 | 0.008 | 0.010 | 0.008 | 0.007 | 0.007 | 0.007 | 0.004 | 0.008 | 0.006 | 0.007 | 0.007 | 0.005 | 0.005 |
| meta399 |  | 251.002 | 78.885 | 0.006 | 0.005 | 0.006 | 0.007 | 0.005 | 0.007 | 0.006 | 0.005 | 0.006 | 0.006 | 0.005 | 0.004 | 0.006 | 0.005 | 0.005 |
| meta400 | Deoxyinosine | 251.077 | 166.320 | 0.003 | 0.002 | 0.002 | 0.004 | 0.003 | 0.003 | 0.003 | 0.002 | 0.001 | 0.001 | 0.002 | 0.002 | 0.002 | 0.002 | 0.002 |
| meta401 |  | 251.200 | 43.032 | 0.061 | 0.065 | 0.069 | 0.070 | 0.086 | 0.080 | 0.085 | 0.070 | 0.078 | 0.083 | 0.064 | 0.118 | 0.100 | 0.125 | 0.087 |
| meta402 |  | 252.913 | 417.472 | 0.007 | 0.007 | 0.006 | 0.009 | 0.006 | 0.005 | 0.006 | 0.007 | 0.004 | 0.006 | 0.006 | 0.006 | 0.004 | 0.005 | 0.005 |
| meta403 |  | 252.922 | 577.356 | 0.005 | 0.005 | 0.005 | 0.008 | 0.006 | 0.005 | 0.006 | 0.006 | 0.006 | 0.006 | 0.005 | 0.005 | 0.006 | 0.006 | 0.005 |
| meta404 |  | 252.923 | 484.052 | 0.021 | 0.016 | 0.024 | 0.036 | 0.020 | 0.019 | 0.018 | 0.019 | 0.018 | 0.016 | 0.011 | 0.028 | 0.023 | 0.021 | 0.021 |
| meta405 |  | 252.943 | 157.673 | 0.002 | 0.002 | 0.002 | 0.004 | 0.003 | 0.002 | 0.002 | 0.002 | 0.003 | 0.002 | 0.003 | 0.003 | 0.003 | 0.002 | 0.003 |
| meta406 |  | 253.037 | 421.088 | 0.013 | 0.011 | 0.009 | 0.013 | 0.012 | 0.007 | 0.010 | 0.009 | 0.012 | 0.003 | 0.014 | 0.012 | 0.012 | 0.009 | 0.014 |
| meta407 |  | 253.179 | 45.996 | 0.022 | 0.019 | 0.022 | 0.032 | 0.034 | 0.021 | 0.066 | 0.026 | 0.022 | 0.025 | 0.008 | 0.020 | 0.005 | 0.012 | 0.007 |
| meta408 | cis-9-Palmitoleic acid | 253.217 | 42.647 | 1.288 | 1.322 | 1.378 | 1.595 | 1.346 | 1.713 | 1.415 | 2.032 | 0.959 | 1.643 | 1.185 | 1.487 | 1.237 | 2.082 | 1.894 |
| meta409 |  | 254.889 | 288.020 | 0.005 | 0.004 | 0.005 | 0.006 | 0.005 | 0.005 | 0.006 | 0.006 | 0.006 | 0.006 | 0.005 | 0.006 | 0.006 | 0.005 | 0.005 |
| meta410 |  | 254.980 | 44.576 | 0.200 | 0.188 | 0.205 | 0.186 | 0.255 | 0.178 | 0.202 | 0.204 | 0.214 | 0.254 | 0.149 | 0.152 | 0.275 | 0.189 | 0.206 |
| meta411 | Primidone | 255.057 | 29.282 | 0.009 | 0.006 | 0.008 | 0.016 | 0.008 | 0.011 | 0.008 | 0.012 | 0.005 | 0.012 | 0.008 | 0.006 | 0.013 | 0.012 | 0.008 |
| meta412 |  | 255.159 | 219.973 | 0.002 | 0.002 | 0.002 | 0.003 | 0.003 | 0.002 | 0.002 | 0.002 | 0.001 | 0.002 | 0.002 | 0.002 | 0.002 | 0.003 | 0.002 |
| meta413 | Palmitic acid | 255.233 | 42.692 | 9.658 | 10.131 | 10.111 | 13.252 | 10.460 | 11.679 | 11.213 | 12.941 | 10.967 | 9.857 | 11.408 | 13.575 | 10.094 | 13.108 | 9.909 |
| meta414 |  | 256.048 | 301.299 | 0.014 | 0.010 | 0.012 | 0.013 | 0.016 | 0.013 | 0.010 | 0.015 | 0.007 | 0.014 | 0.013 | 0.008 | 0.009 | 0.013 | 0.010 |
| meta415 |  | 256.094 | 361.373 | 0.040 | 0.033 | 0.033 | 0.038 | 0.040 | 0.025 | 0.050 | 0.034 | 0.022 | 0.040 | 0.047 | 0.034 | 0.030 | 0.021 | 0.049 |
| meta416 |  | 256.968 | 421.502 | 0.003 | 0.002 | 0.002 | 0.003 | 0.002 | 0.002 | 0.002 | 0.003 | 0.002 | 0.003 | 0.003 | 0.003 | 0.004 | 0.002 | 0.002 |
| meta417 | 6-Phospho-D-gluconate | 257.005 | 406.972 | 0.006 | 0.006 | 0.006 | 0.012 | 0.006 | 0.006 | 0.005 | 0.007 | 0.005 | 0.008 | 0.007 | 0.006 | 0.006 | 0.005 | 0.005 |
| meta418 | Ribothymidine | 257.076 | 134.600 | 0.007 | 0.006 | 0.006 | 0.006 | 0.012 | 0.003 | 0.019 | 0.006 | 0.003 | 0.002 | 0.004 | 0.004 | 0.004 | 0.004 | 0.004 |
| meta419 | 2'-O-Methyluridine | 257.076 | 87.318 | 0.014 | 0.014 | 0.014 | 0.015 | 0.019 | 0.011 | 0.022 | 0.013 | 0.009 | 0.009 | 0.012 | 0.014 | 0.012 | 0.012 | 0.016 |
| meta420 |  | 257.113 | 435.513 | 0.002 | 0.002 | 0.002 | 0.002 | 0.003 | 0.001 | 0.002 | 0.002 | 0.001 | 0.002 | 0.003 | 0.002 | 0.001 | 0.001 | 0.001 |
| meta421 |  | 257.174 | 111.642 | 0.015 | 0.019 | 0.015 | 0.018 | 0.034 | 0.013 | 0.022 | 0.015 | 0.008 | 0.007 | 0.005 | 0.009 | 0.007 | 0.017 | 0.012 |
| meta422 |  | 257.174 | 218.950 | 0.003 | 0.003 | 0.004 | 0.004 | 0.004 | 0.003 | 0.005 | 0.005 | 0.002 | 0.004 | 0.004 | 0.003 | 0.004 | 0.004 | 0.003 |
| meta423 |  | 257.210 | 45.545 | 0.003 | 0.003 | 0.003 | 0.005 | 0.003 | 0.003 | 0.004 | 0.004 | 0.108 | 0.004 | 0.003 | 0.003 | 0.005 | 0.004 | 0.003 |
| meta424 |  | 258.021 | 198.201 | 0.002 | 0.002 | 0.002 | 0.003 | 0.003 | 0.002 | 0.003 | 0.002 | 0.001 | 0.002 | 0.002 | 0.002 | 0.002 | 0.002 | 0.002 |
| meta425 | D-Glucosamine 1-phosphate (Glucosamine-1P) | 258.037 | 402.702 | 0.002 | 0.001 | 0.002 | 0.002 | 0.002 | 0.001 | 0.002 | 0.002 | 0.001 | 0.002 | 0.001 | 0.001 | 0.002 | 0.001 | 0.002 |
| meta426 |  | 258.071 | 388.169 | 0.004 | 0.004 | 0.004 | 0.004 | 0.004 | 0.003 | 0.004 | 0.004 | 0.005 | 0.005 | 0.004 | 0.003 | 0.004 | 0.003 | 0.004 |
| meta427 | Aklomide | 259.011 | 170.358 | 0.001 | 0.000 | 0.001 | 0.001 | 0.001 | 0.001 | 0.000 | 0.001 | 0.001 | 0.001 | 0.001 | 0.001 | 0.001 | 0.000 | 0.001 |
| meta428 | D-Glucose 6-phosphate | 259.021 | 463.510 | 0.030 | 0.026 | 0.031 | 0.069 | 0.030 | 0.033 | 0.023 | 0.036 | 0.057 | 0.037 | 0.034 | 0.031 | 0.036 | 0.043 | 0.022 |
| meta429 | Mesaconic acid | 259.042 | 102.775 | 0.004 | 0.004 | 0.004 | 0.005 | 0.009 | 0.004 | 0.009 | 0.003 | 0.003 | 0.002 | 0.006 | 0.003 | 0.006 | 0.005 | 0.013 |
| meta430 |  | 259.128 | 398.711 | 0.001 | 0.001 | 0.001 | 0.001 | 0.001 | 0.001 | 0.001 | 0.001 | 0.001 | 0.001 | 0.001 | 0.001 | 0.001 | 0.001 | 0.001 |
| meta431 |  | 259.572 | 25.861 | 0.007 | 0.005 | 0.007 | 0.004 | 0.000 | 0.004 | 0.046 | 0.000 | 0.002 | 0.004 | 0.008 | 0.009 | 0.005 | 0.004 | 0.007 |
| meta432 |  | 259.979 | 104.693 | 0.004 | 0.004 | 0.004 | 0.002 | 0.001 | 0.001 | 0.002 | 0.012 | 0.001 | 0.002 | 0.013 | 0.005 | 0.001 | 0.002 | 0.005 |
| meta433 |  | 260.076 | 243.361 | 0.014 | 0.012 | 0.014 | 0.016 | 0.016 | 0.012 | 0.015 | 0.014 | 0.011 | 0.011 | 0.012 | 0.015 | 0.014 | 0.010 | 0.016 |
| meta434 |  | 260.149 | 237.286 | 0.001 | 0.001 | 0.001 | 0.001 | 0.002 | 0.001 | 0.003 | 0.002 | 0.001 | 0.001 | 0.001 | 0.001 | 0.001 | 0.001 | 0.000 |
| meta435 |  | 261.000 | 420.805 | 0.002 | 0.002 | 0.002 | 0.003 | 0.003 | 0.002 | 0.002 | 0.003 | 0.003 | 0.002 | 0.002 | 0.002 | 0.004 | 0.002 | 0.002 |
| meta436 |  | 261.006 | 233.950 | 0.002 | 0.001 | 0.001 | 0.002 | 0.001 | 0.001 | 0.002 | 0.002 | 0.002 | 0.003 | 0.002 | 0.001 | 0.002 | 0.001 | 0.001 |
| meta437 |  | 261.053 | 200.328 | 0.015 | 0.015 | 0.018 | 0.018 | 0.017 | 0.015 | 0.020 | 0.017 | 0.018 | 0.015 | 0.014 | 0.017 | 0.018 | 0.014 | 0.014 |
| meta438 |  | 261.071 | 384.521 | 0.003 | 0.002 | 0.002 | 0.003 | 0.003 | 0.002 | 0.002 | 0.004 | 0.001 | 0.004 | 0.002 | 0.003 | 0.003 | 0.001 | 0.002 |
| meta439 |  | 261.071 | 450.987 | 0.002 | 0.002 | 0.002 | 0.002 | 0.002 | 0.002 | 0.002 | 0.003 | 0.002 | 0.002 | 0.002 | 0.002 | 0.003 | 0.001 | 0.002 |
| meta440 |  | 262.091 | 396.748 | 0.006 | 0.005 | 0.005 | 0.006 | 0.007 | 0.004 | 0.004 | 0.006 | 0.005 | 0.006 | 0.005 | 0.006 | 0.008 | 0.004 | 0.003 |
| meta441 | Acetylcarnitine | 262.127 | 281.719 | 0.000 | 0.000 | 0.001 | 0.000 | 0.001 | 0.000 | 0.001 | 0.001 | 0.001 | 0.000 | 0.000 | 0.001 | 0.001 | 0.000 | 0.001 |
| meta442 | 3-Methoxy-4-Hydroxyphenylglycol Sulfate | 263.021 | 43.768 | 0.009 | 0.008 | 0.007 | 0.009 | 0.011 | 0.008 | 0.009 | 0.007 | 0.007 | 0.011 | 0.007 | 0.005 | 0.007 | 0.009 | 0.006 |
| meta443 |  | 263.063 | 30.128 | 0.001 | 0.001 | 0.001 | 0.002 | 0.001 | 0.001 | 0.001 | 0.002 | 0.000 | 0.001 | 0.001 | 0.001 | 0.001 | 0.001 | 0.001 |
| meta444 |  | 263.138 | 168.918 | 0.001 | 0.001 | 0.001 | 0.002 | 0.001 | 0.001 | 0.001 | 0.001 | 0.000 | 0.001 | 0.001 | 0.001 | 0.001 | 0.001 | 0.001 |
| meta445 |  | 264.039 | 318.741 | 0.004 | 0.003 | 0.003 | 0.003 | 0.004 | 0.002 | 0.002 | 0.005 | 0.003 | 0.005 | 0.004 | 0.002 | 0.003 | 0.002 | 0.005 |
| meta446 | (R)-mevalonic acid 5-Phosphate | 265.081 | 346.426 | 0.004 | 0.003 | 0.004 | 0.005 | 0.003 | 0.005 | 0.002 | 0.006 | 0.002 | 0.004 | 0.005 | 0.007 | 0.003 | 0.004 | 0.004 |
| meta447 |  | 265.117 | 196.421 | 0.001 | 0.001 | 0.001 | 0.002 | 0.001 | 0.001 | 0.001 | 0.000 | 0.000 | 0.000 | 0.001 | 0.001 | 0.001 | 0.001 | 0.001 |
| meta448 |  | 265.136 | 25.038 | 0.277 | 0.307 | 0.315 | 0.785 | 0.437 | 0.425 | 0.378 | 0.410 | 0.316 | 0.308 | 0.266 | 0.313 | 0.398 | 0.373 | 0.398 |
| meta449 |  | 265.146 | 589.007 | 0.067 | 0.044 | 0.047 | 0.021 | 0.067 | 0.043 | 0.056 | 0.060 | 0.021 | 0.055 | 0.028 | 0.053 | 0.021 | 0.017 | 0.058 |
| meta450 |  | 265.146 | 714.216 | 0.016 | 0.011 | 0.017 | 0.076 | 0.083 | 0.014 | 0.014 | 0.016 | 0.018 | 0.021 | 0.011 | 0.016 | 0.018 | 0.017 | 0.015 |
| meta451 |  | 265.215 | 42.009 | 0.010 | 0.008 | 0.009 | 0.011 | 0.008 | 0.010 | 0.010 | 0.009 | 0.013 | 0.014 | 0.010 | 0.012 | 0.011 | 0.012 | 0.011 |
| meta452 |  | 266.254 | 200.064 | 0.004 | 0.003 | 0.003 | 0.004 | 0.004 | 0.003 | 0.005 | 0.002 | 0.002 | 0.002 | 0.003 | 0.003 | 0.003 | 0.004 | 0.002 |
| meta453 |  | 266.938 | 413.295 | 0.009 | 0.007 | 0.006 | 0.010 | 0.009 | 0.005 | 0.006 | 0.011 | 0.006 | 0.008 | 0.010 | 0.008 | 0.007 | 0.004 | 0.006 |
| meta454 |  | 266.966 | 285.182 | 0.005 | 0.003 | 0.003 | 0.005 | 0.005 | 0.003 | 0.005 | 0.004 | 0.003 | 0.004 | 0.004 | 0.004 | 0.004 | 0.003 | 0.003 |
| meta455 |  | 267.023 | 329.610 | 0.002 | 0.002 | 0.002 | 0.002 | 0.002 | 0.002 | 0.002 | 0.002 | 0.002 | 0.002 | 0.002 | 0.001 | 0.002 | 0.002 | 0.002 |
| meta456 | Inosine | 267.073 | 200.054 | 1.420 | 1.278 | 1.345 | 1.584 | 1.979 | 1.350 | 1.883 | 1.163 | 0.818 | 0.981 | 1.221 | 1.528 | 1.301 | 1.391 | 1.182 |
| meta457 | 2'-Deoxy-D-ribose | 267.106 | 336.415 | 0.003 | 0.002 | 0.002 | 0.000 | 0.001 | 0.001 | 0.008 | 0.002 | 0.003 | 0.002 | 0.003 | 0.006 | 0.006 | 0.002 | 0.003 |
| meta458 | Hexadecanedioic acid | 267.194 | 45.935 | 0.017 | 0.014 | 0.016 | 0.018 | 0.019 | 0.021 | 0.020 | 0.023 | 0.010 | 0.015 | 0.016 | 0.020 | 0.016 | 0.025 | 0.019 |
| meta459 |  | 267.231 | 41.371 | 0.043 | 0.042 | 0.047 | 0.055 | 0.039 | 0.042 | 0.048 | 0.072 | 0.050 | 0.075 | 0.055 | 0.064 | 0.052 | 0.061 | 0.061 |
| meta460 |  | 268.948 | 25.103 | 0.003 | 0.004 | 0.003 | 0.004 | 0.004 | 0.002 | 0.003 | 0.002 | 0.005 | 0.003 | 0.003 | 0.002 | 0.004 | 0.002 | 0.003 |
| meta461 |  | 269.211 | 45.256 | 0.053 | 0.048 | 0.052 | 0.081 | 0.063 | 0.075 | 0.073 | 0.066 | 0.045 | 0.060 | 0.063 | 0.064 | 0.054 | 0.081 | 0.059 |
| meta462 | Heptadecanoic acid | 269.247 | 41.372 | 0.055 | 0.057 | 0.063 | 0.084 | 0.052 | 0.050 | 0.067 | 0.076 | 0.075 | 0.063 | 0.104 | 0.081 | 0.062 | 0.068 | 0.063 |
| meta463 |  | 269.942 | 63.206 | 0.004 | 0.004 | 0.004 | 0.004 | 0.004 | 0.003 | 0.004 | 0.003 | 0.003 | 0.004 | 0.002 | 0.003 | 0.003 | 0.003 | 0.005 |
| meta464 |  | 270.034 | 47.720 | 0.006 | 0.005 | 0.005 | 0.003 | 0.001 | 0.004 | 0.003 | 0.006 | 0.006 | 0.004 | 0.006 | 0.009 | 0.005 | 0.002 | 0.003 |
| meta465 |  | 270.059 | 94.458 | 0.005 | 0.006 | 0.006 | 0.011 | 0.015 | 0.008 | 0.013 | 0.005 | 0.003 | 0.003 | 0.006 | 0.002 | 0.005 | 0.007 | 0.009 |
| meta466 |  | 270.941 | 318.126 | 0.027 | 0.023 | 0.027 | 0.028 | 0.025 | 0.032 | 0.027 | 0.023 | 0.031 | 0.025 | 0.025 | 0.022 | 0.032 | 0.034 | 0.022 |
| meta467 |  | 270.974 | 213.985 | 0.033 | 0.043 | 0.041 | 0.049 | 0.035 | 0.043 | 0.037 | 0.031 | 0.024 | 0.028 | 0.025 | 0.026 | 0.032 | 0.022 | 0.033 |
| meta468 |  | 271.002 | 274.501 | 0.018 | 0.018 | 0.017 | 0.020 | 0.020 | 0.016 | 0.017 | 0.021 | 0.017 | 0.020 | 0.018 | 0.015 | 0.017 | 0.019 | 0.017 |
| meta469 |  | 271.055 | 437.951 | 0.013 | 0.009 | 0.010 | 0.010 | 0.010 | 0.008 | 0.012 | 0.011 | 0.010 | 0.013 | 0.010 | 0.012 | 0.014 | 0.008 | 0.011 |
| meta470 | 5,2'-O-dimethyluridine | 271.092 | 75.908 | 0.002 | 0.002 | 0.002 | 0.002 | 0.003 | 0.002 | 0.003 | 0.002 | 0.002 | 0.002 | 0.001 | 0.001 | 0.001 | 0.001 | 0.002 |
| meta471 |  | 271.136 | 32.238 | 0.003 | 0.003 | 0.003 | 0.004 | 0.003 | 0.003 | 0.002 | 0.004 | 0.003 | 0.003 | 0.004 | 0.004 | 0.002 | 0.002 | 0.004 |
| meta472 | 16-Hydroxypalmitic acid | 271.226 | 46.589 | 0.040 | 0.034 | 0.035 | 0.057 | 0.028 | 0.054 | 0.032 | 0.047 | 0.027 | 0.038 | 0.050 | 0.048 | 0.036 | 0.059 | 0.033 |
| meta473 |  | 272.087 | 433.921 | 0.023 | 0.020 | 0.021 | 0.023 | 0.019 | 0.015 | 0.022 | 0.022 | 0.023 | 0.024 | 0.020 | 0.017 | 0.021 | 0.016 | 0.020 |
| meta474 |  | 272.112 | 158.956 | 0.006 | 0.005 | 0.005 | 0.007 | 0.008 | 0.004 | 0.007 | 0.005 | 0.004 | 0.003 | 0.009 | 0.008 | 0.002 | 0.004 | 0.005 |
| meta475 |  | 272.732 | 23.469 | 0.001 | 0.001 | 0.001 | 0.001 | 0.001 | 0.001 | 0.001 | 0.011 | 0.005 | 0.003 | 0.013 | 0.013 | 0.001 | 0.001 | 0.003 |
| meta476 |  | 273.006 | 186.175 | 0.015 | 0.013 | 0.013 | 0.010 | 0.003 | 0.013 | 0.015 | 0.016 | 0.013 | 0.025 | 0.016 | 0.017 | 0.015 | 0.021 | 0.011 |
| meta477 |  | 273.184 | 45.242 | 0.004 | 0.005 | 0.004 | 0.010 | 0.007 | 0.005 | 0.005 | 0.005 | 0.005 | 0.004 | 0.005 | 0.004 | 0.003 | 0.003 | 0.003 |
| meta478 | gamma-L-Glutamyl-L-glutamic acid | 275.086 | 476.330 | 0.001 | 0.000 | 0.000 | 0.001 | 0.001 | 0.000 | 0.000 | 0.001 | 0.000 | 0.001 | 0.000 | 0.001 | 0.001 | 0.000 | 0.000 |
| meta479 | L-Saccharopine | 275.123 | 435.513 | 0.007 | 0.007 | 0.006 | 0.008 | 0.013 | 0.003 | 0.008 | 0.010 | 0.005 | 0.008 | 0.009 | 0.007 | 0.006 | 0.003 | 0.005 |
| meta480 |  | 275.131 | 158.632 | 0.001 | 0.001 | 0.001 | 0.002 | 0.003 | 0.001 | 0.001 | 0.001 | 0.001 | 0.001 | 0.002 | 0.002 | 0.001 | 0.002 | 0.001 |
| meta481 |  | 275.200 | 42.212 | 0.047 | 0.055 | 0.057 | 0.075 | 0.087 | 0.074 | 0.083 | 0.050 | 0.063 | 0.054 | 0.035 | 0.093 | 0.083 | 0.111 | 0.053 |
| meta482 |  | 277.031 | 284.465 | 0.035 | 0.027 | 0.024 | 0.039 | 0.036 | 0.025 | 0.038 | 0.032 | 0.019 | 0.023 | 0.026 | 0.027 | 0.029 | 0.021 | 0.024 |
| meta483 | O-Phosphotyrosine | 277.062 | 38.798 | 0.005 | 0.004 | 0.003 | 0.073 | 0.000 | 0.057 | 0.056 | 0.052 | 0.057 | 0.034 | 0.004 | 0.359 | 0.019 | 0.146 | 0.076 |
| meta484 | Pantetheine | 277.121 | 51.971 | 0.027 | 0.026 | 0.026 | 0.038 | 0.030 | 0.029 | 0.033 | 0.027 | 0.037 | 0.032 | 0.028 | 0.019 | 0.015 | 0.019 | 0.016 |
| meta485 | Phthalic acid Mono-2-ethylhexyl Ester | 277.143 | 45.228 | 0.063 | 0.061 | 0.064 | 0.074 | 0.012 | 0.057 | 0.056 | 0.050 | 0.054 | 0.034 | 0.042 | 0.360 | 0.018 | 0.142 | 0.077 |
| meta486 |  | 277.182 | 33.650 | 0.023 | 0.022 | 0.024 | 0.009 | 0.006 | 0.005 | 0.012 | 0.012 | 0.011 | 0.007 | 0.007 | 0.007 | 0.007 | 0.006 | 0.005 |
| meta487 | all cis-(6,9,12)-Linolenic acid | 277.216 | 41.633 | 0.702 | 0.766 | 0.812 | 0.954 | 0.870 | 1.028 | 0.905 | 0.899 | 0.867 | 0.956 | 0.737 | 1.220 | 1.043 | 1.306 | 0.937 |
| meta488 |  | 278.086 | 243.076 | 0.059 | 0.050 | 0.074 | 0.097 | 0.061 | 0.049 | 0.064 | 0.053 | 0.044 | 0.049 | 0.057 | 0.067 | 0.067 | 0.046 | 0.063 |
| meta489 |  | 278.105 | 100.708 | 0.024 | 0.021 | 0.021 | 0.031 | 0.033 | 0.027 | 0.018 | 0.027 | 0.033 | 0.005 | 0.014 | 0.006 | 0.013 | 0.020 | 0.009 |
| meta490 |  | 278.989 | 65.664 | 0.024 | 0.026 | 0.027 | 0.012 | 0.005 | 0.011 | 0.014 | 0.033 | 0.024 | 0.032 | 0.028 | 0.018 | 0.026 | 0.011 | 0.027 |
| meta491 | Thymidine | 279.037 | 150.822 | 0.331 | 0.277 | 0.364 | 0.325 | 0.367 | 0.244 | 0.385 | 0.304 | 0.211 | 0.165 | 0.235 | 0.294 | 0.234 | 0.241 | 0.246 |
| meta492 | Linoleic acid | 279.232 | 41.481 | 6.246 | 8.186 | 8.273 | 8.464 | 6.856 | 9.184 | 8.696 | 7.688 | 9.918 | 9.293 | 8.603 | 13.273 | 10.865 | 12.623 | 8.786 |
| meta493 |  | 280.901 | 615.240 | 0.030 | 0.026 | 0.023 | 0.031 | 0.029 | 0.020 | 0.030 | 0.019 | 0.029 | 0.022 | 0.023 | 0.017 | 0.026 | 0.024 | 0.023 |
| meta494 | 3'-O-Methylinosine | 281.087 | 136.101 | 0.004 | 0.003 | 0.003 | 0.004 | 0.005 | 0.003 | 0.006 | 0.004 | 0.003 | 0.003 | 0.004 | 0.004 | 0.003 | 0.002 | 0.004 |
| meta495 | Flavone | 281.085 | 347.042 | 0.003 | 0.002 | 0.002 | 0.003 | 0.002 | 0.003 | 0.003 | 0.002 | 0.002 | 0.002 | 0.003 | 0.003 | 0.002 | 0.000 | 0.001 |
| meta496 |  | 281.097 | 254.197 | 0.002 | 0.002 | 0.001 | 0.002 | 0.003 | 0.001 | 0.002 | 0.002 | 0.001 | 0.002 | 0.003 | 0.002 | 0.002 | 0.001 | 0.002 |
| meta497 | Salidroside | 281.097 | 269.276 | 0.001 | 0.001 | 0.001 | 0.001 | 0.001 | 0.001 | 0.001 | 0.001 | 0.001 | 0.000 | 0.000 | 0.000 | 0.001 | 0.000 | 0.000 |
| meta498 |  | 281.106 | 312.965 | 0.003 | 0.003 | 0.003 | 0.005 | 0.004 | 0.003 | 0.004 | 0.003 | 0.003 | 0.002 | 0.003 | 0.004 | 0.003 | 0.003 | 0.003 |
| meta499 | Oleic acid | 281.248 | 40.871 | 4.190 | 4.829 | 5.391 | 5.838 | 4.798 | 6.001 | 5.943 | 7.742 | 4.310 | 6.742 | 5.595 | 6.922 | 6.963 | 7.255 | 8.311 |
| meta500 |  | 282.027 | 89.903 | 0.521 | 0.450 | 0.529 | 0.564 | 0.553 | 0.383 | 0.346 | 0.543 | 0.338 | 0.474 | 0.491 | 0.272 | 0.444 | 0.704 | 0.604 |
| meta501 |  | 282.027 | 74.152 | 0.134 | 0.101 | 0.097 | 0.146 | 0.120 | 0.099 | 0.076 | 0.133 | 0.083 | 0.121 | 0.132 | 0.066 | 0.084 | 0.107 | 0.130 |
| meta502 | Guanosine | 282.082 | 242.108 | 0.013 | 0.010 | 0.011 | 0.011 | 0.017 | 0.011 | 0.013 | 0.012 | 0.010 | 0.010 | 0.009 | 0.015 | 0.013 | 0.012 | 0.012 |
| meta503 |  | 283.008 | 223.298 | 0.003 | 0.002 | 0.002 | 0.003 | 0.002 | 0.002 | 0.002 | 0.002 | 0.003 | 0.003 | 0.002 | 0.003 | 0.002 | 0.003 | 0.003 |
| meta504 | Xanthosine | 283.066 | 292.618 | 0.033 | 0.030 | 0.027 | 0.042 | 0.036 | 0.028 | 0.041 | 0.027 | 0.011 | 0.015 | 0.017 | 0.024 | 0.035 | 0.019 | 0.030 |
| meta505 |  | 283.076 | 256.556 | 0.010 | 0.008 | 0.008 | 0.010 | 0.010 | 0.008 | 0.013 | 0.010 | 0.006 | 0.007 | 0.008 | 0.009 | 0.009 | 0.009 | 0.006 |
| meta506 |  | 283.076 | 238.020 | 0.012 | 0.011 | 0.011 | 0.011 | 0.011 | 0.012 | 0.016 | 0.011 | 0.008 | 0.007 | 0.010 | 0.010 | 0.008 | 0.010 | 0.008 |
| meta507 |  | 283.080 | 206.001 | 0.005 | 0.005 | 0.005 | 0.008 | 0.003 | 0.005 | 0.004 | 0.002 | 0.004 | 0.005 | 0.002 | 0.009 | 0.004 | 0.006 | 0.005 |
| meta508 |  | 283.190 | 177.372 | 0.003 | 0.003 | 0.003 | 0.003 | 0.003 | 0.002 | 0.004 | 0.004 | 0.001 | 0.003 | 0.003 | 0.003 | 0.003 | 0.004 | 0.002 |
| meta509 |  | 284.043 | 302.125 | 0.074 | 0.059 | 0.053 | 0.080 | 0.076 | 0.052 | 0.046 | 0.082 | 0.040 | 0.070 | 0.110 | 0.034 | 0.047 | 0.053 | 0.065 |
| meta510 |  | 284.043 | 280.001 | 0.047 | 0.040 | 0.053 | 0.060 | 0.053 | 0.041 | 0.039 | 0.063 | 0.028 | 0.041 | 0.040 | 0.026 | 0.042 | 0.052 | 0.052 |
| meta511 |  | 284.976 | 285.182 | 0.008 | 0.005 | 0.005 | 0.009 | 0.008 | 0.005 | 0.008 | 0.007 | 0.005 | 0.006 | 0.007 | 0.006 | 0.007 | 0.005 | 0.006 |
| meta512 |  | 286.059 | 333.782 | 1.034 | 0.834 | 0.794 | 1.155 | 1.035 | 0.812 | 0.620 | 1.370 | 0.653 | 1.187 | 0.790 | 0.545 | 0.868 | 0.800 | 0.770 |
| meta513 |  | 286.091 | 390.043 | 0.006 | 0.005 | 0.005 | 0.007 | 0.006 | 0.006 | 0.005 | 0.006 | 0.004 | 0.005 | 0.005 | 0.006 | 0.006 | 0.005 | 0.002 |
| meta514 |  | 286.213 | 42.647 | 0.034 | 0.032 | 0.025 | 0.041 | 0.034 | 0.033 | 0.035 | 0.040 | 0.029 | 0.035 | 0.041 | 0.043 | 0.023 | 0.036 | 0.027 |
| meta515 |  | 286.923 | 157.284 | 0.002 | 0.002 | 0.002 | 0.003 | 0.002 | 0.002 | 0.002 | 0.002 | 0.002 | 0.002 | 0.002 | 0.002 | 0.002 | 0.003 | 0.002 |
| meta516 |  | 286.972 | 318.133 | 0.013 | 0.010 | 0.013 | 0.014 | 0.013 | 0.016 | 0.012 | 0.011 | 0.014 | 0.013 | 0.013 | 0.011 | 0.015 | 0.016 | 0.012 |
| meta517 |  | 287.015 | 420.923 | 0.007 | 0.006 | 0.007 | 0.009 | 0.007 | 0.006 | 0.006 | 0.009 | 0.005 | 0.007 | 0.007 | 0.007 | 0.006 | 0.006 | 0.006 |
| meta518 |  | 287.056 | 221.666 | 0.002 | 0.003 | 0.003 | 0.003 | 0.003 | 0.003 | 0.002 | 0.003 | 0.002 | 0.003 | 0.001 | 0.001 | 0.002 | 0.002 | 0.002 |
| meta519 | N-Tris[hydroxymethyl]methyl-2-aminoethanesulfonic acid [TES] | 288.074 | 243.724 | 0.002 | 0.002 | 0.002 | 0.002 | 0.002 | 0.001 | 0.003 | 0.002 | 0.001 | 0.002 | 0.002 | 0.002 | 0.002 | 0.001 | 0.002 |
| meta520 |  | 288.118 | 383.555 | 0.031 | 0.028 | 0.027 | 0.034 | 0.040 | 0.023 | 0.032 | 0.034 | 0.022 | 0.030 | 0.023 | 0.041 | 0.038 | 0.019 | 0.020 |
| meta521 |  | 288.125 | 43.887 | 0.007 | 0.004 | 0.005 | 0.009 | 0.007 | 0.006 | 0.001 | 0.007 | 0.005 | 0.004 | 0.003 | 0.003 | 0.002 | 0.004 | 0.003 |
| meta522 |  | 288.927 | 85.976 | 0.007 | 0.006 | 0.008 | 0.009 | 0.008 | 0.005 | 0.006 | 0.006 | 0.004 | 0.006 | 0.007 | 0.005 | 0.006 | 0.004 | 0.006 |
| meta523 |  | 288.947 | 413.998 | 0.031 | 0.028 | 0.027 | 0.039 | 0.020 | 0.024 | 0.010 | 0.020 | 0.015 | 0.033 | 0.028 | 0.016 | 0.028 | 0.015 | 0.023 |
| meta524 |  | 288.984 | 214.077 | 0.007 | 0.010 | 0.010 | 0.015 | 0.012 | 0.008 | 0.012 | 0.009 | 0.007 | 0.011 | 0.008 | 0.009 | 0.011 | 0.007 | 0.012 |
| meta525 | D-Ribose 5-phosphate | 289.037 | 355.097 | 0.001 | 0.001 | 0.001 | 0.001 | 0.001 | 0.001 | 0.001 | 0.001 | 0.001 | 0.001 | 0.001 | 0.001 | 0.001 | 0.001 | 0.001 |
| meta526 | Cimetidine | 289.059 | 285.124 | 0.009 | 0.008 | 0.009 | 0.009 | 0.007 | 0.006 | 0.010 | 0.008 | 0.009 | 0.009 | 0.007 | 0.006 | 0.008 | 0.007 | 0.008 |
| meta527 |  | 289.066 | 438.129 | 0.007 | 0.005 | 0.005 | 0.005 | 0.006 | 0.005 | 0.006 | 0.006 | 0.005 | 0.007 | 0.005 | 0.008 | 0.008 | 0.005 | 0.005 |
| meta528 | Argininosuccinic acid | 289.113 | 454.778 | 0.001 | 0.001 | 0.001 | 0.001 | 0.001 | 0.001 | 0.001 | 0.001 | 0.001 | 0.001 | 0.001 | 0.001 | 0.001 | 0.000 | 0.001 |
| meta529 |  | 290.018 | 346.426 | 0.003 | 0.002 | 0.003 | 0.004 | 0.004 | 0.002 | 0.002 | 0.002 | 0.002 | 0.002 | 0.002 | 0.001 | 0.002 | 0.002 | 0.003 |
| meta530 |  | 290.043 | 405.355 | 0.001 | 0.001 | 0.001 | 0.001 | 0.001 | 0.001 | 0.002 | 0.001 | 0.001 | 0.001 | 0.001 | 0.001 | 0.001 | 0.001 | 0.002 |
| meta531 |  | 290.086 | 342.799 | 0.088 | 0.069 | 0.071 | 0.077 | 0.077 | 0.055 | 0.075 | 0.120 | 0.062 | 0.067 | 0.068 | 0.060 | 0.079 | 0.054 | 0.077 |
| meta532 |  | 290.097 | 421.954 | 0.006 | 0.005 | 0.005 | 0.006 | 0.004 | 0.003 | 0.005 | 0.005 | 0.005 | 0.006 | 0.005 | 0.004 | 0.005 | 0.004 | 0.005 |
| meta533 | Suberylglycine | 290.122 | 277.666 | 0.006 | 0.006 | 0.009 | 0.005 | 0.013 | 0.009 | 0.011 | 0.012 | 0.006 | 0.004 | 0.007 | 0.012 | 0.008 | 0.006 | 0.012 |
| meta534 |  | 290.939 | 422.233 | 0.002 | 0.001 | 0.001 | 0.001 | 0.002 | 0.001 | 0.002 | 0.002 | 0.001 | 0.002 | 0.002 | 0.001 | 0.001 | 0.001 | 0.002 |
| meta535 |  | 291.027 | 417.535 | 0.005 | 0.004 | 0.003 | 0.004 | 0.003 | 0.002 | 0.004 | 0.004 | 0.004 | 0.004 | 0.004 | 0.003 | 0.004 | 0.003 | 0.004 |
| meta536 |  | 291.195 | 45.938 | 0.009 | 0.008 | 0.008 | 0.008 | 0.013 | 0.008 | 0.010 | 0.009 | 0.006 | 0.009 | 0.008 | 0.019 | 0.008 | 0.011 | 0.011 |
| meta537 |  | 291.198 | 32.903 | 0.014 | 0.017 | 0.023 | 0.011 | 0.008 | 0.004 | 0.010 | 0.013 | 0.011 | 0.006 | 0.005 | 0.008 | 0.005 | 0.006 | 0.008 |
| meta538 |  | 292.059 | 309.854 | 0.019 | 0.020 | 0.021 | 0.021 | 0.016 | 0.014 | 0.020 | 0.020 | 0.023 | 0.020 | 0.016 | 0.015 | 0.019 | 0.017 | 0.020 |
| meta539 |  | 292.825 | 289.065 | 0.007 | 0.007 | 0.007 | 0.009 | 0.007 | 0.006 | 0.008 | 0.007 | 0.008 | 0.007 | 0.007 | 0.007 | 0.008 | 0.008 | 0.008 |
| meta540 |  | 292.890 | 571.277 | 0.079 | 0.101 | 0.060 | 0.104 | 0.094 | 0.061 | 0.082 | 0.087 | 0.073 | 0.072 | 0.096 | 0.052 | 0.049 | 0.085 | 0.109 |
| meta541 |  | 293.001 | 24.989 | 0.003 | 0.003 | 0.003 | 0.006 | 0.001 | 0.004 | 0.002 | 0.005 | 0.007 | 0.006 | 0.005 | 0.001 | 0.001 | 0.001 | 0.001 |
| meta542 |  | 293.050 | 154.791 | 0.010 | 0.007 | 0.007 | 0.011 | 0.013 | 0.009 | 0.014 | 0.006 | 0.005 | 0.007 | 0.007 | 0.010 | 0.008 | 0.009 | 0.007 |
| meta543 | 5'-O-methylthymidine | 293.061 | 46.703 | 0.004 | 0.003 | 0.004 | 0.003 | 0.004 | 0.003 | 0.003 | 0.003 | 0.003 | 0.003 | 0.003 | 0.002 | 0.003 | 0.008 | 0.003 |
| meta544 |  | 293.097 | 378.894 | 0.003 | 0.003 | 0.003 | 0.003 | 0.004 | 0.002 | 0.003 | 0.004 | 0.003 | 0.004 | 0.005 | 0.004 | 0.003 | 0.003 | 0.003 |
| meta545 | gamma-L-Glutamyl-L-phenylalanine | 293.113 | 328.960 | 0.001 | 0.001 | 0.001 | 0.001 | 0.001 | 0.001 | 0.001 | 0.001 | 0.001 | 0.001 | 0.001 | 0.001 | 0.001 | 0.000 | 0.001 |
| meta546 |  | 293.166 | 25.028 | 0.233 | 0.249 | 0.248 | 0.608 | 0.418 | 0.332 | 0.415 | 0.310 | 0.278 | 0.325 | 0.320 | 0.349 | 0.406 | 0.388 | 0.430 |
| meta547 |  | 293.175 | 44.966 | 0.077 | 0.050 | 0.056 | 0.088 | 0.080 | 0.068 | 0.083 | 0.082 | 0.076 | 0.051 | 0.084 | 0.095 | 0.075 | 0.049 | 0.090 |
| meta548 | Embelin | 293.175 | 77.183 | 0.039 | 0.039 | 0.036 | 0.045 | 0.039 | 0.036 | 0.056 | 0.038 | 0.037 | 0.036 | 0.036 | 0.033 | 0.039 | 0.036 | 0.038 |
| meta549 |  | 294.052 | 24.388 | 0.002 | 0.002 | 0.002 | 0.003 | 0.003 | 0.003 | 0.003 | 0.002 | 0.002 | 0.002 | 0.002 | 0.002 | 0.003 | 0.002 | 0.003 |
| meta550 |  | 294.063 | 320.666 | 0.005 | 0.004 | 0.004 | 0.002 | 0.007 | 0.001 | 0.004 | 0.007 | 0.005 | 0.008 | 0.003 | 0.006 | 0.005 | 0.004 | 0.005 |
| meta551 |  | 294.901 | 287.343 | 0.040 | 0.030 | 0.032 | 0.048 | 0.044 | 0.036 | 0.043 | 0.044 | 0.047 | 0.040 | 0.038 | 0.039 | 0.048 | 0.042 | 0.037 |
| meta552 |  | 294.932 | 427.654 | 0.009 | 0.007 | 0.006 | 0.009 | 0.008 | 0.005 | 0.006 | 0.008 | 0.005 | 0.006 | 0.008 | 0.008 | 0.006 | 0.006 | 0.007 |
| meta553 | 9(S)-HODE | 295.226 | 45.369 | 0.113 | 0.110 | 0.110 | 0.117 | 0.120 | 0.139 | 0.151 | 0.149 | 0.096 | 0.110 | 0.158 | 0.185 | 0.127 | 0.197 | 0.153 |
| meta554 |  | 295.263 | 39.641 | 0.014 | 0.015 | 0.012 | 0.018 | 0.013 | 0.012 | 0.016 | 0.021 | 0.010 | 0.023 | 0.018 | 0.026 | 0.018 | 0.020 | 0.023 |
| meta555 |  | 295.919 | 63.715 | 0.002 | 0.002 | 0.003 | 0.001 | 0.000 | 0.001 | 0.002 | 0.004 | 0.002 | 0.003 | 0.003 | 0.002 | 0.002 | 0.001 | 0.003 |
| meta556 |  | 296.006 | 91.398 | 0.022 | 0.019 | 0.018 | 0.021 | 0.024 | 0.017 | 0.017 | 0.022 | 0.015 | 0.027 | 0.018 | 0.007 | 0.015 | 0.016 | 0.024 |
| meta557 |  | 296.052 | 279.366 | 0.005 | 0.004 | 0.005 | 0.006 | 0.005 | 0.003 | 0.006 | 0.005 | 0.002 | 0.003 | 0.006 | 0.004 | 0.004 | 0.003 | 0.005 |
| meta558 | S-Methyl-5'-thioadenosine | 296.080 | 96.044 | 0.074 | 0.077 | 0.077 | 0.088 | 0.062 | 0.076 | 0.069 | 0.082 | 0.087 | 0.075 | 0.096 | 0.121 | 0.090 | 0.081 | 0.163 |
| meta559 | 3'-O-methylguanosine | 296.098 | 180.590 | 0.001 | 0.000 | 0.000 | 0.001 | 0.001 | 0.000 | 0.001 | 0.001 | 0.001 | 0.000 | 0.001 | 0.001 | 0.000 | 0.000 | 0.001 |
| meta560 |  | 296.125 | 386.469 | 0.002 | 0.002 | 0.002 | 0.001 | 0.001 | 0.002 | 0.002 | 0.002 | 0.002 | 0.001 | 0.001 | 0.006 | 0.007 | 0.004 | 0.006 |
| meta561 |  | 296.133 | 279.352 | 0.007 | 0.006 | 0.006 | 0.007 | 0.008 | 0.006 | 0.010 | 0.009 | 0.004 | 0.007 | 0.008 | 0.007 | 0.006 | 0.003 | 0.006 |
| meta562 | D-Fructose-6-phosphate | 296.972 | 101.647 | 0.004 | 0.002 | 0.002 | 0.005 | 0.005 | 0.003 | 0.006 | 0.004 | 0.003 | 0.003 | 0.005 | 0.002 | 0.003 | 0.004 | 0.009 |
| meta563 |  | 296.976 | 285.136 | 0.001 | 0.001 | 0.001 | 0.001 | 0.001 | 0.001 | 0.001 | 0.001 | 0.001 | 0.001 | 0.001 | 0.001 | 0.001 | 0.000 | 0.001 |
| meta564 |  | 297.140 | 28.013 | 0.674 | 0.623 | 0.581 | 2.509 | 1.245 | 0.807 | 0.525 | 0.469 | 0.547 | 0.387 | 0.341 | 0.398 | 0.573 | 0.380 | 0.443 |
| meta565 |  | 297.151 | 56.241 | 0.051 | 0.046 | 0.045 | 0.063 | 0.050 | 0.050 | 0.044 | 0.043 | 0.049 | 0.040 | 0.044 | 0.038 | 0.044 | 0.041 | 0.048 |
| meta566 | Nname,cis-9,10-Epoxystearic acid | 297.242 | 45.996 | 0.064 | 0.060 | 0.066 | 0.092 | 0.078 | 0.096 | 0.075 | 0.092 | 0.054 | 0.074 | 0.093 | 0.100 | 0.069 | 0.121 | 0.094 |
| meta567 |  | 298.058 | 278.122 | 0.054 | 0.041 | 0.045 | 0.009 | 0.125 | 0.006 | 0.102 | 0.007 | 0.053 | 0.040 | 0.005 | 0.006 | 0.063 | 0.022 | 0.011 |
| meta568 |  | 298.058 | 304.230 | 0.008 | 0.006 | 0.007 | 0.002 | 0.025 | 0.001 | 0.019 | 0.001 | 0.007 | 0.010 | 0.001 | 0.001 | 0.012 | 0.003 | 0.002 |
| meta569 |  | 298.068 | 436.679 | 0.011 | 0.008 | 0.009 | 0.010 | 0.011 | 0.009 | 0.009 | 0.009 | 0.008 | 0.011 | 0.011 | 0.010 | 0.008 | 0.008 | 0.006 |
| meta570 |  | 298.859 | 289.265 | 0.008 | 0.009 | 0.009 | 0.010 | 0.009 | 0.008 | 0.009 | 0.009 | 0.010 | 0.009 | 0.009 | 0.009 | 0.010 | 0.008 | 0.009 |
| meta571 | D-Sorbitol 6-phosphate | 299.097 | 360.042 | 0.004 | 0.003 | 0.003 | 0.004 | 0.004 | 0.004 | 0.005 | 0.004 | 0.004 | 0.004 | 0.004 | 0.004 | 0.004 | 0.003 | 0.004 |
| meta572 |  | 299.118 | 25.551 | 0.068 | 0.072 | 0.073 | 0.107 | 0.104 | 0.082 | 0.131 | 0.119 | 0.111 | 0.110 | 0.109 | 0.101 | 0.097 | 0.065 | 0.113 |
| meta573 | Palmitaldehyde | 299.257 | 46.024 | 0.026 | 0.023 | 0.025 | 0.040 | 0.032 | 0.026 | 0.032 | 0.031 | 0.025 | 0.026 | 0.031 | 0.031 | 0.026 | 0.037 | 0.031 |
| meta574 |  | 300.038 | 85.945 | 0.047 | 0.034 | 0.041 | 0.068 | 0.029 | 0.053 | 0.037 | 0.032 | 0.023 | 0.023 | 0.012 | 0.019 | 0.028 | 0.030 | 0.046 |
| meta575 | N-Acetylglucosamine 1-phosphate | 300.047 | 431.842 | 0.010 | 0.007 | 0.009 | 0.009 | 0.009 | 0.006 | 0.008 | 0.010 | 0.011 | 0.012 | 0.007 | 0.008 | 0.009 | 0.008 | 0.011 |
| meta576 |  | 300.128 | 44.002 | 0.010 | 0.010 | 0.009 | 0.013 | 0.010 | 0.012 | 0.013 | 0.012 | 0.013 | 0.017 | 0.009 | 0.007 | 0.009 | 0.011 | 0.008 |
| meta577 |  | 301.019 | 150.817 | 0.008 | 0.008 | 0.007 | 0.010 | 0.009 | 0.006 | 0.011 | 0.008 | 0.006 | 0.005 | 0.007 | 0.008 | 0.007 | 0.007 | 0.007 |
| meta578 |  | 301.025 | 27.213 | 0.006 | 0.005 | 0.006 | 0.010 | 0.003 | 0.007 | 0.004 | 0.013 | 0.010 | 0.011 | 0.007 | 0.001 | 0.003 | 0.003 | 0.002 |
| meta579 |  | 301.138 | 326.324 | 0.001 | 0.001 | 0.001 | 0.001 | 0.001 | 0.001 | 0.001 | 0.001 | 0.001 | 0.001 | 0.001 | 0.001 | 0.001 | 0.001 | 0.001 |
| meta580 |  | 301.163 | 32.958 | 0.001 | 0.001 | 0.001 | 0.001 | 0.001 | 0.002 | 0.001 | 0.001 | 0.001 | 0.001 | 0.001 | 0.001 | 0.001 | 0.001 | 0.001 |
| meta581 |  | 301.200 | 130.820 | 0.002 | 0.002 | 0.002 | 0.003 | 0.002 | 0.002 | 0.004 | 0.003 | 0.002 | 0.001 | 0.001 | 0.001 | 0.000 | 0.000 | 0.000 |
| meta582 |  | 302.053 | 225.318 | 0.005 | 0.005 | 0.004 | 0.007 | 0.003 | 0.006 | 0.008 | 0.005 | 0.003 | 0.003 | 0.004 | 0.004 | 0.004 | 0.002 | 0.005 |
| meta583 |  | 302.053 | 301.090 | 0.035 | 0.028 | 0.026 | 0.050 | 0.045 | 0.033 | 0.030 | 0.037 | 0.019 | 0.032 | 0.030 | 0.021 | 0.021 | 0.041 | 0.044 |
| meta584 |  | 302.065 | 157.969 | 0.333 | 0.311 | 0.299 | 0.296 | 0.484 | 0.212 | 0.322 | 0.419 | 0.329 | 0.313 | 0.358 | 0.309 | 0.353 | 0.272 | 0.356 |
| meta585 |  | 302.099 | 361.425 | 0.004 | 0.003 | 0.003 | 0.004 | 0.004 | 0.002 | 0.005 | 0.003 | 0.002 | 0.004 | 0.004 | 0.003 | 0.002 | 0.002 | 0.005 |
| meta586 |  | 303.024 | 97.043 | 0.022 | 0.023 | 0.026 | 0.001 | 0.010 | 0.001 | 0.059 | 0.027 | 0.029 | 0.023 | 0.014 | 0.007 | 0.028 | 0.003 | 0.023 |
| meta587 | Niflumic Acid | 303.030 | 318.126 | 0.002 | 0.002 | 0.002 | 0.002 | 0.002 | 0.002 | 0.001 | 0.002 | 0.001 | 0.003 | 0.002 | 0.001 | 0.002 | 0.003 | 0.001 |
| meta588 |  | 303.048 | 200.268 | 0.007 | 0.005 | 0.005 | 0.007 | 0.007 | 0.005 | 0.006 | 0.004 | 0.004 | 0.004 | 0.005 | 0.006 | 0.005 | 0.006 | 0.004 |
| meta589 | 2-Deoxy-D-glucose 6-phosphate | 303.051 | 78.931 | 0.002 | 0.002 | 0.003 | 0.003 | 0.002 | 0.003 | 0.002 | 0.002 | 0.002 | 0.003 | 0.002 | 0.002 | 0.002 | 0.002 | 0.003 |
| meta590 | Oxypurinol | 303.057 | 300.759 | 0.004 | 0.003 | 0.002 | 0.003 | 0.003 | 0.002 | 0.002 | 0.004 | 0.002 | 0.003 | 0.003 | 0.001 | 0.002 | 0.003 | 0.003 |
| meta591 | Uridine | 303.079 | 253.635 | 0.000 | 0.001 | 0.000 | 0.000 | 0.001 | 0.000 | 0.000 | 0.000 | 0.000 | 0.000 | 0.000 | 0.000 | 0.000 | 0.000 | 0.000 |
| meta592 |  | 303.198 | 32.392 | 0.004 | 0.005 | 0.005 | 0.008 | 0.009 | 0.008 | 0.004 | 0.005 | 0.006 | 0.005 | 0.004 | 0.004 | 0.004 | 0.005 | 0.009 |
| meta593 | Arachidonic Acid (peroxide free) | 303.232 | 40.270 | 2.850 | 2.983 | 2.594 | 4.493 | 4.408 | 4.020 | 4.293 | 3.194 | 2.764 | 3.270 | 4.256 | 4.850 | 2.741 | 5.139 | 3.243 |
| meta594 |  | 304.069 | 275.399 | 0.056 | 0.051 | 0.053 | 0.064 | 0.063 | 0.050 | 0.062 | 0.063 | 0.044 | 0.048 | 0.043 | 0.044 | 0.056 | 0.044 | 0.047 |
| meta595 |  | 304.095 | 254.697 | 0.003 | 0.003 | 0.003 | 0.003 | 0.003 | 0.003 | 0.002 | 0.002 | 0.001 | 0.002 | 0.003 | 0.002 | 0.003 | 0.002 | 0.004 |
| meta596 |  | 304.113 | 411.280 | 0.008 | 0.007 | 0.008 | 0.009 | 0.011 | 0.006 | 0.009 | 0.009 | 0.006 | 0.008 | 0.007 | 0.008 | 0.009 | 0.005 | 0.006 |
| meta597 |  | 304.551 | 466.551 | 0.005 | 0.004 | 0.005 | 0.005 | 0.005 | 0.005 | 0.005 | 0.006 | 0.006 | 0.007 | 0.005 | 0.005 | 0.007 | 0.005 | 0.005 |
| meta598 |  | 305.043 | 381.262 | 0.032 | 0.027 | 0.027 | 0.031 | 0.037 | 0.025 | 0.032 | 0.036 | 0.034 | 0.041 | 0.028 | 0.027 | 0.035 | 0.023 | 0.059 |
| meta599 | 3-Hydroxyanthranilic acid | 305.071 | 275.468 | 0.006 | 0.005 | 0.005 | 0.007 | 0.007 | 0.005 | 0.007 | 0.008 | 0.005 | 0.005 | 0.005 | 0.004 | 0.005 | 0.005 | 0.005 |
| meta600 |  | 305.097 | 362.075 | 0.043 | 0.033 | 0.032 | 0.048 | 0.049 | 0.035 | 0.042 | 0.047 | 0.029 | 0.030 | 0.037 | 0.044 | 0.053 | 0.031 | 0.037 |
| meta601 | Dihomo-gamma-Linolenic Acid | 305.246 | 40.039 | 0.319 | 0.311 | 0.316 | 0.416 | 0.356 | 0.387 | 0.462 | 0.529 | 0.371 | 0.530 | 0.465 | 0.502 | 0.425 | 0.444 | 0.474 |
| meta602 |  | 306.038 | 329.158 | 0.019 | 0.016 | 0.016 | 0.019 | 0.015 | 0.012 | 0.017 | 0.019 | 0.019 | 0.020 | 0.016 | 0.013 | 0.016 | 0.014 | 0.016 |
| meta603 |  | 306.056 | 150.874 | 0.229 | 0.215 | 0.195 | 0.245 | 0.277 | 0.168 | 0.311 | 0.226 | 0.155 | 0.124 | 0.176 | 0.230 | 0.166 | 0.182 | 0.179 |
| meta604 | Glutathione | 306.074 | 387.953 | 0.042 | 0.043 | 0.046 | 0.042 | 0.044 | 0.032 | 0.062 | 0.053 | 0.060 | 0.059 | 0.036 | 0.035 | 0.058 | 0.034 | 0.042 |
| meta605 | Oxazepam | 307.022 | 341.525 | 0.001 | 0.001 | 0.001 | 0.001 | 0.002 | 0.001 | 0.001 | 0.001 | 0.001 | 0.002 | 0.001 | 0.001 | 0.001 | 0.001 | 0.001 |
| meta606 | Pyridoxamine 5'-phosphate | 307.066 | 328.240 | 0.001 | 0.001 | 0.001 | 0.001 | 0.001 | 0.001 | 0.001 | 0.001 | 0.001 | 0.002 | 0.001 | 0.001 | 0.001 | 0.001 | 0.001 |
| meta607 |  | 307.144 | 25.639 | 0.020 | 0.020 | 0.020 | 0.033 | 0.034 | 0.020 | 0.041 | 0.021 | 0.032 | 0.019 | 0.030 | 0.028 | 0.027 | 0.025 | 0.035 |
| meta608 | 11(Z),14(Z)-Eicosadienoic Acid | 307.262 | 40.091 | 0.111 | 0.110 | 0.107 | 0.146 | 0.096 | 0.112 | 0.121 | 0.224 | 0.138 | 0.180 | 0.181 | 0.193 | 0.134 | 0.146 | 0.186 |
| meta609 | Pyridoxine 5-phosphate | 308.054 | 422.718 | 0.003 | 0.002 | 0.003 | 0.002 | 0.003 | 0.001 | 0.002 | 0.002 | 0.002 | 0.002 | 0.002 | 0.002 | 0.002 | 0.002 | 0.004 |
| meta610 |  | 308.053 | 405.654 | 0.007 | 0.008 | 0.008 | 0.009 | 0.007 | 0.005 | 0.008 | 0.008 | 0.010 | 0.010 | 0.007 | 0.005 | 0.008 | 0.007 | 0.009 |
| meta611 |  | 308.097 | 280.626 | 0.079 | 0.062 | 0.069 | 0.077 | 0.076 | 0.064 | 0.091 | 0.098 | 0.052 | 0.058 | 0.074 | 0.077 | 0.083 | 0.059 | 0.062 |
| meta612 |  | 308.098 | 94.953 | 0.014 | 0.013 | 0.014 | 0.019 | 0.022 | 0.014 | 0.022 | 0.017 | 0.007 | 0.008 | 0.011 | 0.010 | 0.011 | 0.014 | 0.013 |
| meta613 | Picrotoxinin | 308.115 | 398.651 | 0.002 | 0.002 | 0.002 | 0.003 | 0.003 | 0.002 | 0.002 | 0.003 | 0.003 | 0.002 | 0.002 | 0.002 | 0.002 | 0.002 | 0.002 |
| meta614 | D-Ribulose 1,5-bisphosphate | 308.981 | 96.661 | 0.002 | 0.002 | 0.002 | 0.002 | 0.001 | 0.000 | 0.003 | 0.003 | 0.003 | 0.002 | 0.001 | 0.000 | 0.002 | 0.002 | 0.001 |
| meta615 |  | 309.018 | 154.686 | 0.001 | 0.001 | 0.001 | 0.002 | 0.002 | 0.002 | 0.002 | 0.001 | 0.001 | 0.001 | 0.001 | 0.002 | 0.001 | 0.001 | 0.001 |
| meta616 |  | 309.038 | 350.963 | 0.018 | 0.016 | 0.015 | 0.018 | 0.014 | 0.011 | 0.017 | 0.017 | 0.018 | 0.018 | 0.015 | 0.013 | 0.016 | 0.013 | 0.015 |
| meta617 |  | 309.064 | 174.333 | 0.005 | 0.004 | 0.006 | 0.002 | 0.004 | 0.001 | 0.007 | 0.018 | 0.005 | 0.006 | 0.009 | 0.014 | 0.006 | 0.001 | 0.002 |
| meta618 | D-Ribose 1,5-bisphosphate | 309.081 | 359.460 | 0.001 | 0.001 | 0.001 | 0.001 | 0.000 | 0.001 | 0.001 | 0.001 | 0.001 | 0.001 | 0.001 | 0.001 | 0.001 | 0.001 | 0.000 |
| meta619 |  | 309.143 | 296.648 | 0.001 | 0.001 | 0.001 | 0.001 | 0.001 | 0.000 | 0.001 | 0.002 | 0.002 | 0.002 | 0.001 | 0.000 | 0.000 | 0.000 | 0.000 |
| meta620 |  | 309.170 | 44.527 | 0.015 | 0.015 | 0.015 | 0.030 | 0.028 | 0.012 | 0.034 | 0.021 | 0.013 | 0.014 | 0.022 | 0.016 | 0.019 | 0.022 | 0.024 |
| meta621 | 2E-Eicosenoic acid | 309.278 | 39.461 | 0.081 | 0.076 | 0.071 | 0.078 | 0.060 | 0.065 | 0.079 | 0.154 | 0.090 | 0.121 | 0.130 | 0.116 | 0.085 | 0.119 | 0.146 |
| meta622 |  | 310.900 | 571.277 | 0.033 | 0.030 | 0.027 | 0.045 | 0.041 | 0.035 | 0.030 | 0.028 | 0.038 | 0.032 | 0.042 | 0.043 | 0.057 | 0.028 | 0.033 |
| meta623 |  | 310.932 | 287.061 | 0.020 | 0.014 | 0.015 | 0.025 | 0.024 | 0.019 | 0.022 | 0.023 | 0.024 | 0.020 | 0.019 | 0.021 | 0.027 | 0.020 | 0.018 |
| meta624 |  | 310.933 | 318.126 | 0.010 | 0.009 | 0.011 | 0.011 | 0.009 | 0.012 | 0.010 | 0.009 | 0.013 | 0.009 | 0.009 | 0.009 | 0.012 | 0.012 | 0.009 |
| meta625 |  | 310.992 | 423.445 | 0.003 | 0.003 | 0.003 | 0.004 | 0.004 | 0.002 | 0.003 | 0.005 | 0.004 | 0.004 | 0.004 | 0.005 | 0.008 | 0.003 | 0.003 |
| meta626 | Phloretin | 311.035 | 351.109 | 0.001 | 0.001 | 0.001 | 0.001 | 0.001 | 0.001 | 0.001 | 0.001 | 0.001 | 0.001 | 0.001 | 0.001 | 0.001 | 0.001 | 0.001 |
| meta627 |  | 311.131 | 31.782 | 0.001 | 0.002 | 0.002 | 0.003 | 0.002 | 0.002 | 0.002 | 0.003 | 0.001 | 0.002 | 0.002 | 0.001 | 0.004 | 0.003 | 0.002 |
| meta628 |  | 311.155 | 27.568 | 2.488 | 2.369 | 2.323 | 9.459 | 5.086 | 3.168 | 2.055 | 1.882 | 2.068 | 1.603 | 1.347 | 1.508 | 2.067 | 1.544 | 1.629 |
| meta629 |  | 311.220 | 152.689 | 0.003 | 0.004 | 0.004 | 0.003 | 0.004 | 0.004 | 0.007 | 0.004 | 0.002 | 0.004 | 0.005 | 0.006 | 0.004 | 0.003 | 0.003 |
| meta630 |  | 312.119 | 398.119 | 0.001 | 0.001 | 0.001 | 0.000 | 0.000 | 0.001 | 0.001 | 0.001 | 0.001 | 0.000 | 0.000 | 0.002 | 0.005 | 0.002 | 0.004 |
| meta631 |  | 312.906 | 161.220 | 0.003 | 0.003 | 0.002 | 0.004 | 0.004 | 0.003 | 0.002 | 0.001 | 0.004 | 0.001 | 0.003 | 0.003 | 0.003 | 0.002 | 0.003 |
| meta632 |  | 312.911 | 318.147 | 0.001 | 0.001 | 0.002 | 0.002 | 0.001 | 0.002 | 0.002 | 0.001 | 0.002 | 0.001 | 0.001 | 0.001 | 0.002 | 0.002 | 0.001 |
| meta633 |  | 312.985 | 85.738 | 0.007 | 0.005 | 0.005 | 0.007 | 0.008 | 0.005 | 0.006 | 0.008 | 0.004 | 0.008 | 0.006 | 0.003 | 0.005 | 0.004 | 0.005 |
| meta634 | Geranyl diphosphate | 313.057 | 431.494 | 0.001 | 0.001 | 0.001 | 0.001 | 0.001 | 0.000 | 0.001 | 0.001 | 0.001 | 0.001 | 0.001 | 0.001 | 0.001 | 0.001 | 0.001 |
| meta635 | Tosyllysine Chloromethyl Ketone | 313.084 | 112.954 | 0.009 | 0.008 | 0.008 | 0.008 | 0.009 | 0.007 | 0.008 | 0.012 | 0.005 | 0.013 | 0.014 | 0.005 | 0.007 | 0.006 | 0.009 |
| meta636 |  | 313.112 | 420.145 | 0.002 | 0.002 | 0.002 | 0.002 | 0.002 | 0.001 | 0.002 | 0.003 | 0.002 | 0.004 | 0.002 | 0.003 | 0.002 | 0.002 | 0.001 |
| meta637 |  | 313.111 | 351.666 | 0.049 | 0.040 | 0.039 | 0.052 | 0.047 | 0.035 | 0.045 | 0.053 | 0.049 | 0.051 | 0.046 | 0.040 | 0.053 | 0.039 | 0.042 |
| meta638 |  | 313.117 | 190.387 | 0.001 | 0.001 | 0.001 | 0.001 | 0.001 | 0.001 | 0.001 | 0.001 | 0.001 | 0.001 | 0.001 | 0.001 | 0.001 | 0.001 | 0.001 |
| meta639 | 9,10-DiHOME | 313.236 | 75.169 | 0.015 | 0.015 | 0.016 | 0.019 | 0.017 | 0.017 | 0.019 | 0.031 | 0.018 | 0.019 | 0.020 | 0.028 | 0.021 | 0.022 | 0.016 |
| meta640 |  | 313.928 | 568.677 | 0.028 | 0.024 | 0.021 | 0.029 | 0.024 | 0.019 | 0.026 | 0.027 | 0.023 | 0.024 | 0.026 | 0.022 | 0.026 | 0.016 | 0.034 |
| meta641 |  | 314.062 | 240.145 | 0.021 | 0.018 | 0.021 | 0.022 | 0.014 | 0.014 | 0.020 | 0.019 | 0.014 | 0.020 | 0.023 | 0.017 | 0.010 | 0.006 | 0.007 |
| meta642 |  | 314.963 | 433.376 | 0.009 | 0.007 | 0.005 | 0.008 | 0.007 | 0.005 | 0.007 | 0.012 | 0.010 | 0.016 | 0.009 | 0.010 | 0.017 | 0.005 | 0.008 |
| meta643 |  | 314.963 | 463.836 | 0.005 | 0.004 | 0.004 | 0.004 | 0.006 | 0.006 | 0.003 | 0.007 | 0.005 | 0.009 | 0.004 | 0.007 | 0.009 | 0.003 | 0.004 |
| meta644 |  | 314.986 | 285.124 | 0.011 | 0.008 | 0.007 | 0.012 | 0.011 | 0.008 | 0.011 | 0.010 | 0.007 | 0.009 | 0.010 | 0.010 | 0.009 | 0.007 | 0.008 |
| meta645 |  | 315.031 | 154.655 | 0.001 | 0.001 | 0.001 | 0.001 | 0.002 | 0.001 | 0.002 | 0.001 | 0.001 | 0.001 | 0.001 | 0.001 | 0.002 | 0.001 | 0.001 |
| meta646 |  | 315.079 | 381.299 | 0.012 | 0.011 | 0.009 | 0.013 | 0.015 | 0.008 | 0.011 | 0.016 | 0.012 | 0.014 | 0.014 | 0.011 | 0.013 | 0.010 | 0.009 |
| meta647 |  | 315.122 | 28.009 | 0.016 | 0.014 | 0.018 | 0.023 | 0.022 | 0.017 | 0.023 | 0.023 | 0.024 | 0.025 | 0.018 | 0.015 | 0.025 | 0.017 | 0.020 |
| meta648 |  | 315.173 | 158.627 | 0.001 | 0.001 | 0.001 | 0.002 | 0.000 | 0.001 | 0.000 | 0.000 | 0.000 | 0.000 | 0.001 | 0.001 | 0.000 | 0.001 | 0.001 |
| meta649 | 15-Deoxy-delta-12,14-PGJ2 | 315.194 | 46.541 | 0.008 | 0.007 | 0.010 | 0.012 | 0.015 | 0.007 | 0.011 | 0.010 | 0.010 | 0.007 | 0.009 | 0.010 | 0.005 | 0.009 | 0.007 |
| meta650 |  | 315.231 | 40.064 | 0.007 | 0.007 | 0.006 | 0.012 | 0.009 | 0.010 | 0.007 | 0.009 | 0.010 | 0.011 | 0.009 | 0.011 | 0.008 | 0.010 | 0.012 |
| meta651 |  | 316.059 | 355.757 | 0.006 | 0.005 | 0.005 | 0.002 | 0.011 | 0.001 | 0.018 | 0.002 | 0.012 | 0.004 | 0.002 | 0.002 | 0.008 | 0.004 | 0.003 |
| meta652 |  | 316.115 | 361.373 | 0.149 | 0.119 | 0.112 | 0.145 | 0.152 | 0.087 | 0.185 | 0.126 | 0.077 | 0.148 | 0.175 | 0.129 | 0.104 | 0.069 | 0.184 |
| meta653 |  | 317.018 | 25.052 | 0.004 | 0.005 | 0.004 | 0.008 | 0.002 | 0.007 | 0.003 | 0.006 | 0.005 | 0.009 | 0.003 | 0.001 | 0.001 | 0.001 | 0.001 |
| meta654 |  | 317.097 | 433.806 | 0.004 | 0.003 | 0.003 | 0.004 | 0.004 | 0.003 | 0.004 | 0.004 | 0.003 | 0.004 | 0.004 | 0.004 | 0.004 | 0.004 | 0.003 |
| meta655 | 3-Methyluridine | 317.097 | 79.316 | 0.001 | 0.001 | 0.001 | 0.001 | 0.002 | 0.001 | 0.002 | 0.001 | 0.001 | 0.001 | 0.001 | 0.001 | 0.001 | 0.001 | 0.000 |
| meta656 |  | 317.133 | 406.555 | 0.001 | 0.001 | 0.001 | 0.001 | 0.001 | 0.001 | 0.001 | 0.002 | 0.001 | 0.001 | 0.001 | 0.001 | 0.001 | 0.001 | 0.001 |
| meta657 | 3,5-Dibromo-L-tyrosine | 317.880 | 63.280 | 0.002 | 0.002 | 0.003 | 0.003 | 0.002 | 0.002 | 0.003 | 0.002 | 0.002 | 0.002 | 0.003 | 0.002 | 0.002 | 0.002 | 0.003 |
| meta658 |  | 318.056 | 174.772 | 0.003 | 0.003 | 0.004 | 0.002 | 0.002 | 0.002 | 0.005 | 0.002 | 0.007 | 0.003 | 0.004 | 0.003 | 0.003 | 0.003 | 0.001 |
| meta659 |  | 318.075 | 379.271 | 0.091 | 0.080 | 0.083 | 0.038 | 0.146 | 0.026 | 0.339 | 0.033 | 0.193 | 0.089 | 0.030 | 0.034 | 0.170 | 0.054 | 0.032 |
| meta660 | Hesperetin | 318.094 | 361.373 | 0.071 | 0.060 | 0.059 | 0.068 | 0.072 | 0.044 | 0.089 | 0.059 | 0.039 | 0.072 | 0.083 | 0.060 | 0.053 | 0.035 | 0.089 |
| meta661 |  | 318.885 | 581.396 | 0.012 | 0.011 | 0.009 | 0.011 | 0.012 | 0.008 | 0.011 | 0.011 | 0.009 | 0.011 | 0.010 | 0.011 | 0.009 | 0.010 | 0.010 |
| meta662 |  | 319.138 | 233.369 | 0.001 | 0.001 | 0.001 | 0.001 | 0.001 | 0.001 | 0.001 | 0.000 | 0.000 | 0.001 | 0.001 | 0.001 | 0.001 | 0.001 | 0.001 |
| meta663 |  | 319.192 | 33.549 | 0.003 | 0.003 | 0.002 | 0.004 | 0.003 | 0.003 | 0.004 | 0.003 | 0.006 | 0.004 | 0.004 | 0.003 | 0.004 | 0.004 | 0.003 |
| meta664 | 5(S)-HETE | 319.226 | 45.256 | 0.031 | 0.030 | 0.026 | 0.029 | 0.042 | 0.037 | 0.044 | 0.040 | 0.024 | 0.031 | 0.047 | 0.052 | 0.025 | 0.079 | 0.037 |
| meta665 |  | 320.009 | 381.592 | 0.002 | 0.002 | 0.001 | 0.002 | 0.002 | 0.001 | 0.001 | 0.002 | 0.002 | 0.001 | 0.002 | 0.001 | 0.002 | 0.001 | 0.002 |
| meta666 |  | 320.090 | 373.554 | 0.008 | 0.007 | 0.007 | 0.007 | 0.007 | 0.006 | 0.011 | 0.009 | 0.008 | 0.010 | 0.008 | 0.005 | 0.007 | 0.005 | 0.008 |
| meta667 |  | 320.147 | 159.287 | 0.002 | 0.002 | 0.002 | 0.002 | 0.002 | 0.002 | 0.001 | 0.002 | 0.003 | 0.002 | 0.003 | 0.001 | 0.002 | 0.001 | 0.002 |
| meta668 |  | 321.030 | 25.610 | 1.488 | 1.532 | 1.491 | 2.191 | 1.365 | 1.660 | 1.945 | 3.785 | 3.716 | 0.004 | 1.921 | 0.851 | 0.819 | 0.907 | 0.650 |
| meta669 |  | 321.085 | 405.206 | 0.049 | 0.045 | 0.049 | 0.045 | 0.038 | 0.034 | 0.052 | 0.054 | 0.061 | 0.068 | 0.043 | 0.036 | 0.054 | 0.033 | 0.045 |
| meta670 |  | 321.122 | 27.321 | 0.002 | 0.002 | 0.003 | 0.002 | 0.005 | 0.001 | 0.004 | 0.001 | 0.004 | 0.002 | 0.003 | 0.002 | 0.002 | 0.001 | 0.003 |
| meta671 | D-Mannitol 1-phosphate | 321.172 | 32.726 | 0.006 | 0.006 | 0.005 | 0.006 | 0.011 | 0.007 | 0.004 | 0.011 | 0.005 | 0.013 | 0.006 | 0.004 | 0.004 | 0.003 | 0.005 |
| meta672 |  | 321.195 | 24.390 | 0.007 | 0.010 | 0.011 | 0.013 | 0.025 | 0.009 | 0.023 | 0.007 | 0.021 | 0.008 | 0.017 | 0.027 | 0.015 | 0.012 | 0.028 |
| meta673 |  | 321.241 | 45.324 | 0.009 | 0.011 | 0.010 | 0.011 | 0.012 | 0.010 | 0.013 | 0.016 | 0.009 | 0.012 | 0.010 | 0.011 | 0.011 | 0.026 | 0.009 |
| meta674 | Cytidine 5'-monophosphate (CMP) | 322.042 | 447.648 | 0.006 | 0.006 | 0.007 | 0.006 | 0.006 | 0.006 | 0.007 | 0.008 | 0.008 | 0.009 | 0.007 | 0.008 | 0.008 | 0.006 | 0.007 |
| meta675 |  | 322.075 | 254.079 | 0.002 | 0.002 | 0.002 | 0.002 | 0.003 | 0.001 | 0.002 | 0.002 | 0.001 | 0.002 | 0.002 | 0.002 | 0.001 | 0.001 | 0.002 |
| meta676 |  | 322.112 | 190.842 | 0.002 | 0.002 | 0.002 | 0.002 | 0.001 | 0.002 | 0.001 | 0.002 | 0.002 | 0.002 | 0.003 | 0.004 | 0.002 | 0.002 | 0.004 |
| meta677 |  | 322.111 | 281.914 | 0.001 | 0.001 | 0.002 | 0.002 | 0.001 | 0.002 | 0.001 | 0.001 | 0.000 | 0.001 | 0.000 | 0.001 | 0.002 | 0.001 | 0.001 |
| meta678 | Uridine 5'-monophosphate (UMP) | 323.027 | 428.932 | 0.155 | 0.132 | 0.142 | 0.161 | 0.147 | 0.114 | 0.147 | 0.217 | 0.166 | 0.151 | 0.140 | 0.153 | 0.178 | 0.121 | 0.155 |
| meta679 |  | 323.060 | 277.404 | 0.041 | 0.039 | 0.039 | 0.043 | 0.038 | 0.042 | 0.014 | 0.049 | 0.056 | 0.014 | 0.046 | 0.038 | 0.068 | 0.056 | 0.027 |
| meta680 |  | 323.153 | 28.636 | 0.033 | 0.024 | 0.027 | 0.122 | 0.057 | 0.044 | 0.022 | 0.023 | 0.034 | 0.019 | 0.016 | 0.022 | 0.030 | 0.025 | 0.026 |
| meta681 |  | 324.091 | 382.175 | 0.011 | 0.011 | 0.010 | 0.012 | 0.009 | 0.010 | 0.009 | 0.012 | 0.009 | 0.012 | 0.010 | 0.010 | 0.013 | 0.008 | 0.010 |
| meta682 |  | 325.011 | 318.130 | 0.002 | 0.001 | 0.001 | 0.001 | 0.002 | 0.002 | 0.000 | 0.001 | 0.001 | 0.002 | 0.002 | 0.000 | 0.001 | 0.002 | 0.001 |
| meta683 | (+-)-Taxifolin | 325.033 | 432.200 | 0.066 | 0.049 | 0.046 | 0.051 | 0.043 | 0.034 | 0.045 | 0.060 | 0.063 | 0.071 | 0.054 | 0.048 | 0.052 | 0.044 | 0.055 |
| meta684 |  | 325.039 | 196.421 | 0.005 | 0.005 | 0.005 | 0.007 | 0.006 | 0.005 | 0.006 | 0.005 | 0.004 | 0.005 | 0.005 | 0.005 | 0.007 | 0.006 | 0.006 |
| meta685 |  | 325.063 | 150.812 | 0.038 | 0.032 | 0.029 | 0.036 | 0.044 | 0.029 | 0.051 | 0.036 | 0.025 | 0.020 | 0.026 | 0.033 | 0.028 | 0.030 | 0.029 |
| meta686 |  | 325.069 | 230.234 | 0.059 | 0.058 | 0.055 | 0.015 | 0.017 | 0.000 | 0.086 | 0.208 | 0.073 | 0.053 | 0.020 | 0.038 | 0.055 | 0.003 | 0.018 |
| meta687 |  | 325.079 | 447.499 | 0.001 | 0.001 | 0.001 | 0.001 | 0.001 | 0.001 | 0.001 | 0.003 | 0.002 | 0.002 | 0.001 | 0.001 | 0.001 | 0.001 | 0.001 |
| meta688 |  | 325.086 | 188.284 | 0.021 | 0.018 | 0.017 | 0.021 | 0.020 | 0.017 | 0.018 | 0.017 | 0.012 | 0.018 | 0.019 | 0.020 | 0.018 | 0.017 | 0.015 |
| meta689 | Maltitol | 325.112 | 308.614 | 0.001 | 0.001 | 0.001 | 0.001 | 0.001 | 0.001 | 0.001 | 0.001 | 0.000 | 0.001 | 0.001 | 0.001 | 0.000 | 0.000 | 0.000 |
| meta690 |  | 325.170 | 27.321 | 2.824 | 2.779 | 2.770 | 12.493 | 6.623 | 4.044 | 2.476 | 2.185 | 2.296 | 1.998 | 1.526 | 1.828 | 2.267 | 1.998 | 1.861 |
| meta691 |  | 325.182 | 41.600 | 0.117 | 0.119 | 0.112 | 0.133 | 0.113 | 0.094 | 0.110 | 0.106 | 0.105 | 0.111 | 0.115 | 0.106 | 0.107 | 0.120 | 0.115 |
| meta692 |  | 325.182 | 55.740 | 0.180 | 0.157 | 0.153 | 0.190 | 0.160 | 0.137 | 0.166 | 0.150 | 0.153 | 0.166 | 0.146 | 0.132 | 0.160 | 0.158 | 0.157 |
| meta693 |  | 325.939 | 26.681 | 0.015 | 0.017 | 0.016 | 0.003 | 0.004 | 0.003 | 0.004 | 0.004 | 0.011 | 0.016 | 0.005 | 0.006 | 0.015 | 0.005 | 0.016 |
| meta694 | Adenosine | 326.108 | 156.774 | 0.034 | 0.029 | 0.028 | 0.028 | 0.050 | 0.021 | 0.033 | 0.044 | 0.025 | 0.033 | 0.029 | 0.027 | 0.031 | 0.024 | 0.034 |
| meta695 |  | 326.107 | 280.120 | 0.001 | 0.001 | 0.001 | 0.002 | 0.000 | 0.001 | 0.001 | 0.001 | 0.001 | 0.001 | 0.001 | 0.002 | 0.001 | 0.001 | 0.001 |
| meta696 |  | 326.904 | 412.515 | 0.002 | 0.002 | 0.002 | 0.003 | 0.003 | 0.002 | 0.001 | 0.003 | 0.002 | 0.003 | 0.003 | 0.002 | 0.002 | 0.001 | 0.002 |
| meta697 |  | 326.958 | 422.233 | 0.016 | 0.014 | 0.013 | 0.019 | 0.015 | 0.008 | 0.013 | 0.015 | 0.013 | 0.013 | 0.016 | 0.014 | 0.013 | 0.013 | 0.014 |
| meta698 |  | 326.991 | 165.670 | 0.003 | 0.002 | 0.002 | 0.001 | 0.001 | 0.001 | 0.002 | 0.010 | 0.002 | 0.003 | 0.003 | 0.001 | 0.001 | 0.000 | 0.001 |
| meta699 |  | 327.041 | 97.105 | 0.023 | 0.020 | 0.019 | 0.000 | 0.001 | 0.000 | 0.078 | 0.130 | 0.065 | 0.022 | 0.003 | 0.005 | 0.022 | 0.000 | 0.003 |
| meta700 | (4Z,7Z,10Z,13Z,16Z,19Z)-4,7,10,13,1 6,19-Docosahexaenoic acid | 327.232 | 40.729 | 1.905 | 1.956 | 2.053 | 2.879 | 2.819 | 2.855 | 3.158 | 2.868 | 2.485 | 2.439 | 3.550 | 4.051 | 2.327 | 3.762 | 2.992 |
| meta701 |  | 327.288 | 45.996 | 0.002 | 0.002 | 0.002 | 0.003 | 0.002 | 0.002 | 0.003 | 0.003 | 0.005 | 0.003 | 0.003 | 0.002 | 0.002 | 0.003 | 0.002 |
| meta702 |  | 328.037 | 150.812 | 0.015 | 0.011 | 0.011 | 0.014 | 0.014 | 0.010 | 0.015 | 0.013 | 0.010 | 0.008 | 0.011 | 0.012 | 0.010 | 0.011 | 0.011 |
| meta703 | 2'-Deoxyguanosine 5'-monophosphate (dGMP) | 328.044 | 443.372 | 0.010 | 0.010 | 0.007 | 0.009 | 0.009 | 0.008 | 0.008 | 0.008 | 0.007 | 0.008 | 0.006 | 0.010 | 0.007 | 0.005 | 0.006 |
| meta704 | Adenosine 3',5'-cyclic phosphate (cAMP) | 328.043 | 267.790 | 0.000 | 0.001 | 0.000 | 0.001 | 0.000 | 0.001 | 0.000 | 0.001 | 0.000 | 0.000 | 0.000 | 0.000 | 0.001 | 0.001 | 0.001 |
| meta705 |  | 328.888 | 287.983 | 0.001 | 0.001 | 0.001 | 0.002 | 0.002 | 0.001 | 0.001 | 0.002 | 0.001 | 0.001 | 0.001 | 0.001 | 0.002 | 0.002 | 0.001 |
| meta706 |  | 328.981 | 318.133 | 0.007 | 0.005 | 0.007 | 0.007 | 0.006 | 0.008 | 0.006 | 0.006 | 0.007 | 0.007 | 0.006 | 0.006 | 0.007 | 0.008 | 0.006 |
| meta707 | Deoxyuridine monophosphate (dUMP) | 329.010 | 85.602 | 0.057 | 0.056 | 0.061 | 0.088 | 0.083 | 0.059 | 0.080 | 0.050 | 0.049 | 0.056 | 0.063 | 0.031 | 0.059 | 0.046 | 0.066 |
| meta708 |  | 329.028 | 113.366 | 0.002 | 0.002 | 0.002 | 0.001 | 0.002 | 0.001 | 0.005 | 0.001 | 0.009 | 0.004 | 0.002 | 0.001 | 0.001 | 0.002 | 0.002 |
| meta709 |  | 329.082 | 156.650 | 0.007 | 0.005 | 0.005 | 0.006 | 0.008 | 0.004 | 0.006 | 0.007 | 0.005 | 0.007 | 0.007 | 0.006 | 0.005 | 0.005 | 0.006 |
| meta710 |  | 329.188 | 151.196 | 0.004 | 0.004 | 0.004 | 0.011 | 0.001 | 0.005 | 0.002 | 0.003 | 0.002 | 0.003 | 0.009 | 0.006 | 0.001 | 0.005 | 0.012 |
| meta711 |  | 330.067 | 200.371 | 0.033 | 0.030 | 0.028 | 0.036 | 0.041 | 0.026 | 0.040 | 0.028 | 0.022 | 0.022 | 0.030 | 0.034 | 0.027 | 0.033 | 0.025 |
| meta712 |  | 330.128 | 362.367 | 0.003 | 0.002 | 0.002 | 0.003 | 0.004 | 0.002 | 0.003 | 0.002 | 0.002 | 0.003 | 0.002 | 0.004 | 0.003 | 0.002 | 0.002 |
| meta713 |  | 330.890 | 417.390 | 0.001 | 0.000 | 0.000 | 0.001 | 0.001 | 0.001 | 0.001 | 0.001 | 0.000 | 0.000 | 0.001 | 0.001 | 0.000 | 0.000 | 0.000 |
| meta714 |  | 330.899 | 577.548 | 0.002 | 0.002 | 0.002 | 0.003 | 0.003 | 0.002 | 0.002 | 0.002 | 0.002 | 0.002 | 0.002 | 0.002 | 0.002 | 0.002 | 0.002 |
| meta715 |  | 330.899 | 484.482 | 0.006 | 0.005 | 0.008 | 0.013 | 0.010 | 0.009 | 0.007 | 0.007 | 0.007 | 0.005 | 0.006 | 0.007 | 0.009 | 0.011 | 0.006 |
| meta716 |  | 330.916 | 161.830 | 0.004 | 0.004 | 0.003 | 0.006 | 0.004 | 0.003 | 0.002 | 0.001 | 0.006 | 0.002 | 0.005 | 0.004 | 0.004 | 0.003 | 0.005 |
| meta717 |  | 331.075 | 150.817 | 0.005 | 0.004 | 0.005 | 0.006 | 0.008 | 0.004 | 0.009 | 0.004 | 0.002 | 0.002 | 0.003 | 0.004 | 0.003 | 0.003 | 0.004 |
| meta718 |  | 331.123 | 420.163 | 0.008 | 0.006 | 0.006 | 0.006 | 0.006 | 0.004 | 0.006 | 0.011 | 0.007 | 0.012 | 0.005 | 0.009 | 0.005 | 0.005 | 0.005 |
| meta719 | Adrenic Acid | 331.263 | 39.445 | 0.197 | 0.165 | 0.171 | 0.251 | 0.366 | 0.243 | 0.342 | 0.205 | 0.178 | 0.215 | 0.380 | 0.256 | 0.221 | 0.258 | 0.285 |
| meta720 |  | 331.265 | 707.932 | 0.004 | 0.003 | 0.002 | 0.003 | 0.003 | 0.004 | 0.004 | 0.002 | 0.002 | 0.005 | 0.002 | 0.003 | 0.002 | 0.004 | 0.002 |
| meta721 | 2'-Deoxyinosine 5'-monophosphate | 332.056 | 95.554 | 0.014 | 0.011 | 0.012 | 0.016 | 0.014 | 0.014 | 0.011 | 0.008 | 0.011 | 0.012 | 0.018 | 0.018 | 0.013 | 0.014 | 0.028 |
| meta722 |  | 332.107 | 437.309 | 0.004 | 0.003 | 0.003 | 0.003 | 0.003 | 0.002 | 0.003 | 0.005 | 0.003 | 0.006 | 0.003 | 0.005 | 0.003 | 0.002 | 0.002 |
| meta723 |  | 332.151 | 45.148 | 0.008 | 0.007 | 0.008 | 0.011 | 0.015 | 0.009 | 0.007 | 0.009 | 0.006 | 0.009 | 0.006 | 0.005 | 0.006 | 0.006 | 0.007 |
| meta724 |  | 332.981 | 85.413 | 0.008 | 0.008 | 0.009 | 0.012 | 0.012 | 0.008 | 0.012 | 0.006 | 0.007 | 0.007 | 0.010 | 0.003 | 0.009 | 0.009 | 0.011 |
| meta725 |  | 333.005 | 31.677 | 0.021 | 0.015 | 0.019 | 0.025 | 0.003 | 0.023 | 0.008 | 0.052 | 0.041 | 0.021 | 0.028 | 0.004 | 0.002 | 0.003 | 0.002 |
| meta726 | Nicotinamide ribotide | 333.057 | 421.502 | 0.036 | 0.030 | 0.028 | 0.043 | 0.032 | 0.022 | 0.043 | 0.036 | 0.020 | 0.025 | 0.026 | 0.027 | 0.028 | 0.022 | 0.028 |
| meta727 |  | 333.110 | 174.789 | 0.001 | 0.001 | 0.001 | 0.002 | 0.001 | 0.001 | 0.001 | 0.001 | 0.001 | 0.001 | 0.002 | 0.001 | 0.002 | 0.001 | 0.001 |
| meta728 | 15-keto-PGE1 | 333.205 | 40.738 | 0.007 | 0.008 | 0.007 | 0.008 | 0.006 | 0.008 | 0.010 | 0.008 | 0.004 | 0.005 | 0.011 | 0.012 | 0.007 | 0.011 | 0.005 |
| meta729 | Docosatrienoic Acid | 333.277 | 38.811 | 0.013 | 0.011 | 0.010 | 0.016 | 0.017 | 0.015 | 0.021 | 0.024 | 0.015 | 0.016 | 0.023 | 0.018 | 0.014 | 0.020 | 0.021 |
| meta730 |  | 334.893 | 318.133 | 0.006 | 0.005 | 0.007 | 0.007 | 0.006 | 0.008 | 0.006 | 0.005 | 0.008 | 0.006 | 0.006 | 0.006 | 0.008 | 0.008 | 0.006 |
| meta731 |  | 334.932 | 285.612 | 0.000 | 0.000 | 0.000 | 0.000 | 0.000 | 0.000 | 0.000 | 0.000 | 0.000 | 0.000 | 0.000 | 0.000 | 0.000 | 0.001 | 0.000 |
| meta732 |  | 335.008 | 27.980 | 0.056 | 0.073 | 0.075 | 0.198 | 0.035 | 0.141 | 0.081 | 0.145 | 0.128 | 0.048 | 0.101 | 0.011 | 0.017 | 0.015 | 0.017 |
| meta733 |  | 335.092 | 352.111 | 0.004 | 0.003 | 0.004 | 0.004 | 0.004 | 0.003 | 0.004 | 0.004 | 0.004 | 0.004 | 0.004 | 0.003 | 0.004 | 0.003 | 0.004 |
| meta734 |  | 335.175 | 25.746 | 0.003 | 0.004 | 0.004 | 0.004 | 0.008 | 0.003 | 0.011 | 0.004 | 0.012 | 0.003 | 0.007 | 0.008 | 0.004 | 0.003 | 0.011 |
| meta735 | 5(S)-HpETE | 335.220 | 47.273 | 0.008 | 0.008 | 0.009 | 0.011 | 0.013 | 0.007 | 0.011 | 0.022 | 0.007 | 0.016 | 0.009 | 0.012 | 0.014 | 0.011 | 0.019 |
| meta736 |  | 336.146 | 109.602 | 0.008 | 0.007 | 0.007 | 0.008 | 0.006 | 0.007 | 0.014 | 0.008 | 0.006 | 0.008 | 0.003 | 0.006 | 0.004 | 0.004 | 0.002 |
| meta737 | Erucamide | 336.325 | 32.282 | 0.008 | 0.009 | 0.009 | 0.006 | 0.004 | 0.002 | 0.005 | 0.005 | 0.006 | 0.004 | 0.007 | 0.009 | 0.005 | 0.006 | 0.012 |
| meta738 |  | 336.815 | 289.082 | 0.013 | 0.012 | 0.014 | 0.016 | 0.014 | 0.012 | 0.014 | 0.013 | 0.015 | 0.012 | 0.012 | 0.012 | 0.015 | 0.014 | 0.013 |
| meta739 |  | 336.897 | 580.396 | 0.165 | 0.151 | 0.144 | 0.166 | 0.151 | 0.109 | 0.154 | 0.148 | 0.129 | 0.150 | 0.150 | 0.147 | 0.140 | 0.138 | 0.136 |
| meta740 |  | 336.998 | 463.592 | 0.003 | 0.002 | 0.002 | 0.003 | 0.003 | 0.004 | 0.002 | 0.003 | 0.003 | 0.004 | 0.003 | 0.004 | 0.004 | 0.002 | 0.002 |
| meta741 |  | 337.013 | 154.377 | 0.001 | 0.001 | 0.000 | 0.001 | 0.001 | 0.001 | 0.001 | 0.001 | 0.000 | 0.001 | 0.001 | 0.001 | 0.001 | 0.001 | 0.001 |
| meta742 |  | 337.023 | 24.581 | 0.143 | 0.108 | 0.103 | 0.287 | 0.092 | 0.208 | 0.083 | 0.236 | 0.183 | 0.276 | 0.139 | 0.034 | 0.081 | 0.054 | 0.035 |
| meta743 |  | 337.051 | 284.465 | 0.039 | 0.028 | 0.024 | 0.041 | 0.039 | 0.028 | 0.041 | 0.037 | 0.019 | 0.025 | 0.028 | 0.027 | 0.029 | 0.020 | 0.026 |
| meta744 |  | 337.060 | 381.299 | 0.005 | 0.004 | 0.004 | 0.005 | 0.006 | 0.003 | 0.005 | 0.007 | 0.005 | 0.005 | 0.006 | 0.005 | 0.005 | 0.004 | 0.004 |
| meta745 |  | 337.060 | 170.331 | 0.003 | 0.002 | 0.002 | 0.002 | 0.002 | 0.002 | 0.002 | 0.003 | 0.001 | 0.002 | 0.002 | 0.003 | 0.002 | 0.002 | 0.002 |
| meta746 |  | 337.081 | 433.921 | 0.134 | 0.116 | 0.120 | 0.126 | 0.105 | 0.084 | 0.130 | 0.126 | 0.143 | 0.137 | 0.109 | 0.090 | 0.119 | 0.092 | 0.127 |
| meta747 |  | 337.122 | 366.267 | 0.003 | 0.003 | 0.003 | 0.004 | 0.002 | 0.003 | 0.001 | 0.002 | 0.001 | 0.002 | 0.003 | 0.003 | 0.002 | 0.002 | 0.002 |
| meta748 |  | 337.168 | 27.856 | 0.024 | 0.023 | 0.020 | 0.094 | 0.048 | 0.032 | 0.024 | 0.024 | 0.020 | 0.020 | 0.016 | 0.016 | 0.026 | 0.018 | 0.020 |
| meta749 |  | 337.235 | 45.294 | 0.021 | 0.017 | 0.018 | 0.020 | 0.020 | 0.017 | 0.027 | 0.024 | 0.023 | 0.019 | 0.022 | 0.027 | 0.022 | 0.023 | 0.031 |
| meta750 | Erucic acid | 337.309 | 38.776 | 0.010 | 0.007 | 0.007 | 0.007 | 0.007 | 0.004 | 0.009 | 0.012 | 0.012 | 0.009 | 0.015 | 0.007 | 0.006 | 0.010 | 0.011 |
| meta751 |  | 338.037 | 438.372 | 0.009 | 0.008 | 0.009 | 0.010 | 0.009 | 0.007 | 0.011 | 0.010 | 0.007 | 0.016 | 0.008 | 0.009 | 0.012 | 0.008 | 0.006 |
| meta752 | 5'-Phosphoribosyl-5-amino-4-imidazolecarboxamide (AICAR) | 338.064 | 453.665 | 0.008 | 0.006 | 0.006 | 0.007 | 0.008 | 0.006 | 0.007 | 0.008 | 0.009 | 0.009 | 0.006 | 0.007 | 0.008 | 0.004 | 0.006 |
| meta753 |  | 338.078 | 349.574 | 0.001 | 0.001 | 0.001 | 0.001 | 0.001 | 0.001 | 0.001 | 0.001 | 0.002 | 0.002 | 0.001 | 0.002 | 0.001 | 0.001 | 0.001 |
| meta754 |  | 338.107 | 45.256 | 0.002 | 0.002 | 0.001 | 0.002 | 0.001 | 0.002 | 0.004 | 0.003 | 0.003 | 0.002 | 0.002 | 0.001 | 0.001 | 0.001 | 0.001 |
| meta755 |  | 339.163 | 242.629 | 0.001 | 0.001 | 0.001 | 0.001 | 0.001 | 0.001 | 0.000 | 0.001 | 0.001 | 0.001 | 0.000 | 0.001 | 0.001 | 0.001 | 0.001 |
| meta756 |  | 339.185 | 26.759 | 2.013 | 2.002 | 1.942 | 8.090 | 4.224 | 2.942 | 2.129 | 2.220 | 1.534 | 2.094 | 1.615 | 1.692 | 2.278 | 2.192 | 1.853 |
| meta757 | Norethindrone Acetate | 339.197 | 55.889 | 0.114 | 0.115 | 0.108 | 0.127 | 0.107 | 0.082 | 0.129 | 0.103 | 0.109 | 0.105 | 0.103 | 0.092 | 0.105 | 0.111 | 0.099 |
| meta758 |  | 340.056 | 378.918 | 0.003 | 0.003 | 0.003 | 0.002 | 0.004 | 0.001 | 0.008 | 0.002 | 0.005 | 0.002 | 0.001 | 0.001 | 0.005 | 0.002 | 0.001 |
| meta759 |  | 340.123 | 398.713 | 0.047 | 0.044 | 0.041 | 0.064 | 0.053 | 0.046 | 0.043 | 0.047 | 0.026 | 0.036 | 0.036 | 0.045 | 0.039 | 0.036 | 0.035 |
| meta760 |  | 341.031 | 150.803 | 0.005 | 0.005 | 0.005 | 0.005 | 0.007 | 0.004 | 0.006 | 0.005 | 0.004 | 0.004 | 0.004 | 0.005 | 0.004 | 0.005 | 0.004 |
| meta761 |  | 341.081 | 246.312 | 0.200 | 0.181 | 0.115 | 0.230 | 0.180 | 0.163 | 0.181 | 0.190 | 0.124 | 0.182 | 0.207 | 0.167 | 0.232 | 0.162 | 0.189 |
| meta762 | Sucrose | 341.106 | 344.769 | 0.034 | 0.029 | 0.030 | 0.031 | 0.031 | 0.040 | 0.030 | 0.032 | 0.021 | 0.027 | 0.030 | 0.037 | 0.026 | 0.024 | 0.018 |
| meta763 |  | 342.049 | 196.824 | 0.004 | 0.003 | 0.003 | 0.005 | 0.001 | 0.004 | 0.001 | 0.003 | 0.001 | 0.003 | 0.005 | 0.003 | 0.001 | 0.002 | 0.006 |
| meta764 |  | 343.009 | 25.175 | 0.007 | 0.010 | 0.012 | 0.009 | 0.007 | 0.009 | 0.013 | 0.018 | 0.016 | 0.017 | 0.011 | 0.005 | 0.006 | 0.006 | 0.004 |
| meta765 |  | 343.067 | 405.074 | 0.004 | 0.004 | 0.004 | 0.004 | 0.003 | 0.003 | 0.004 | 0.004 | 0.005 | 0.005 | 0.004 | 0.003 | 0.005 | 0.003 | 0.004 |
| meta766 | Pyrethrosin | 343.088 | 412.794 | 0.002 | 0.001 | 0.002 | 0.001 | 0.001 | 0.001 | 0.001 | 0.002 | 0.001 | 0.002 | 0.001 | 0.001 | 0.002 | 0.001 | 0.001 |
| meta767 |  | 343.094 | 104.695 | 0.003 | 0.003 | 0.003 | 0.006 | 0.002 | 0.004 | 0.004 | 0.003 | 0.001 | 0.003 | 0.004 | 0.002 | 0.003 | 0.002 | 0.006 |
| meta768 |  | 343.173 | 170.933 | 0.001 | 0.001 | 0.001 | 0.002 | 0.001 | 0.001 | 0.001 | 0.001 | 0.002 | 0.001 | 0.000 | 0.001 | 0.001 | 0.001 | 0.000 |
| meta769 |  | 343.225 | 45.256 | 0.016 | 0.017 | 0.018 | 0.017 | 0.023 | 0.020 | 0.033 | 0.028 | 0.016 | 0.015 | 0.034 | 0.036 | 0.019 | 0.033 | 0.023 |
| meta770 |  | 343.246 | 104.894 | 0.041 | 0.037 | 0.061 | 0.069 | 0.089 | 0.072 | 0.076 | 0.048 | 0.030 | 0.024 | 0.032 | 0.033 | 0.006 | 0.009 | 0.019 |
| meta771 | Stearic acid | 343.283 | 32.897 | 0.002 | 0.002 | 0.002 | 0.004 | 0.002 | 0.003 | 0.006 | 0.007 | 0.004 | 0.005 | 0.003 | 0.004 | 0.001 | 0.001 | 0.001 |
| meta772 |  | 344.983 | 86.115 | 0.006 | 0.005 | 0.005 | 0.010 | 0.008 | 0.006 | 0.008 | 0.005 | 0.005 | 0.005 | 0.006 | 0.003 | 0.005 | 0.004 | 0.007 |
| meta773 |  | 345.013 | 318.111 | 0.001 | 0.001 | 0.001 | 0.001 | 0.001 | 0.002 | 0.001 | 0.001 | 0.002 | 0.001 | 0.001 | 0.001 | 0.001 | 0.002 | 0.001 |
| meta774 |  | 345.084 | 335.860 | 0.001 | 0.001 | 0.001 | 0.000 | 0.000 | 0.000 | 0.001 | 0.002 | 0.003 | 0.001 | 0.000 | 0.001 | 0.001 | 0.000 | 0.000 |
| meta775 | Corticosterone | 345.205 | 115.400 | 0.002 | 0.001 | 0.001 | 0.002 | 0.004 | 0.001 | 0.002 | 0.002 | 0.001 | 0.001 | 0.001 | 0.001 | 0.000 | 0.001 | 0.001 |
| meta776 |  | 345.262 | 136.975 | 0.002 | 0.002 | 0.002 | 0.003 | 0.003 | 0.003 | 0.003 | 0.002 | 0.002 | 0.001 | 0.001 | 0.002 | 0.001 | 0.001 | 0.001 |
| meta777 | Adenosine monophosphate (AMP) | 346.055 | 417.535 | 0.642 | 0.607 | 0.643 | 0.671 | 0.571 | 0.545 | 0.613 | 0.797 | 0.789 | 0.749 | 0.663 | 0.664 | 0.858 | 0.607 | 0.711 |
| meta778 |  | 346.123 | 383.151 | 0.004 | 0.003 | 0.003 | 0.005 | 0.004 | 0.002 | 0.003 | 0.004 | 0.002 | 0.005 | 0.003 | 0.004 | 0.003 | 0.002 | 0.002 |
| meta779 |  | 346.131 | 45.235 | 0.005 | 0.005 | 0.005 | 0.007 | 0.005 | 0.007 | 0.005 | 0.004 | 0.005 | 0.009 | 0.008 | 0.004 | 0.003 | 0.004 | 0.005 |
| meta780 |  | 346.159 | 340.885 | 0.001 | 0.001 | 0.001 | 0.000 | 0.001 | 0.000 | 0.002 | 0.003 | 0.002 | 0.001 | 0.001 | 0.003 | 0.001 | 0.000 | 0.000 |
| meta781 |  | 346.167 | 45.281 | 0.016 | 0.013 | 0.015 | 0.017 | 0.017 | 0.014 | 0.031 | 0.019 | 0.013 | 0.015 | 0.008 | 0.017 | 0.008 | 0.010 | 0.009 |
| meta782 | Inosine 5'-monophosphate (IMP) | 347.037 | 434.719 | 0.045 | 0.043 | 0.045 | 0.047 | 0.049 | 0.041 | 0.042 | 0.047 | 0.038 | 0.059 | 0.050 | 0.056 | 0.057 | 0.041 | 0.045 |
| meta783 |  | 347.044 | 150.779 | 0.007 | 0.005 | 0.005 | 0.007 | 0.007 | 0.005 | 0.007 | 0.007 | 0.006 | 0.005 | 0.006 | 0.006 | 0.005 | 0.006 | 0.006 |
| meta784 |  | 347.132 | 196.151 | 0.001 | 0.001 | 0.001 | 0.001 | 0.001 | 0.000 | 0.001 | 0.001 | 0.000 | 0.000 | 0.000 | 0.000 | 0.000 | 0.001 | 0.000 |
| meta785 | trans-Dehydroandrosterone | 347.220 | 46.265 | 0.008 | 0.009 | 0.008 | 0.011 | 0.020 | 0.007 | 0.012 | 0.012 | 0.005 | 0.006 | 0.005 | 0.006 | 0.007 | 0.007 | 0.009 |
| meta786 |  | 347.256 | 45.974 | 0.003 | 0.003 | 0.003 | 0.004 | 0.007 | 0.004 | 0.006 | 0.005 | 0.002 | 0.003 | 0.004 | 0.004 | 0.004 | 0.005 | 0.004 |
| meta787 |  | 348.084 | 331.591 | 0.004 | 0.004 | 0.004 | 0.004 | 0.002 | 0.003 | 0.007 | 0.002 | 0.006 | 0.004 | 0.009 | 0.005 | 0.004 | 0.003 | 0.002 |
| meta788 |  | 348.901 | 417.733 | 0.002 | 0.001 | 0.001 | 0.002 | 0.001 | 0.001 | 0.001 | 0.002 | 0.001 | 0.001 | 0.001 | 0.001 | 0.001 | 0.001 | 0.001 |
| meta789 |  | 348.932 | 577.406 | 0.003 | 0.002 | 0.002 | 0.003 | 0.003 | 0.002 | 0.002 | 0.002 | 0.002 | 0.002 | 0.002 | 0.002 | 0.002 | 0.002 | 0.002 |
| meta790 |  | 348.986 | 26.332 | 0.018 | 0.023 | 0.025 | 0.039 | 0.003 | 0.046 | 0.012 | 0.094 | 0.093 | 0.067 | 0.033 | 0.007 | 0.004 | 0.004 | 0.003 |
| meta791 | Orotidine, 5'-phosphate (OMP) | 349.014 | 329.706 | 0.001 | 0.001 | 0.001 | 0.001 | 0.001 | 0.001 | 0.001 | 0.001 | 0.001 | 0.002 | 0.001 | 0.001 | 0.001 | 0.001 | 0.001 |
| meta792 |  | 349.023 | 97.442 | 0.037 | 0.037 | 0.038 | 0.001 | 0.002 | 0.001 | 0.139 | 0.130 | 0.084 | 0.042 | 0.011 | 0.009 | 0.045 | 0.000 | 0.012 |
| meta793 |  | 349.038 | 283.393 | 0.008 | 0.006 | 0.008 | 0.008 | 0.006 | 0.008 | 0.002 | 0.011 | 0.011 | 0.002 | 0.008 | 0.005 | 0.010 | 0.010 | 0.006 |
| meta794 |  | 349.074 | 200.268 | 0.007 | 0.008 | 0.007 | 0.008 | 0.009 | 0.008 | 0.009 | 0.007 | 0.005 | 0.006 | 0.006 | 0.008 | 0.007 | 0.008 | 0.006 |
| meta795 |  | 349.189 | 27.665 | 0.004 | 0.002 | 0.003 | 0.005 | 0.002 | 0.004 | 0.002 | 0.004 | 0.003 | 0.002 | 0.004 | 0.002 | 0.002 | 0.002 | 0.015 |
| meta796 |  | 349.199 | 182.179 | 0.003 | 0.003 | 0.004 | 0.003 | 0.001 | 0.002 | 0.002 | 0.003 | 0.005 | 0.006 | 0.003 | 0.003 | 0.001 | 0.001 | 0.003 |
| meta797 | Tetrahydrocorticosterone | 349.236 | 46.977 | 0.003 | 0.003 | 0.004 | 0.005 | 0.008 | 0.003 | 0.004 | 0.004 | 0.002 | 0.005 | 0.003 | 0.004 | 0.011 | 0.002 | 0.003 |
| meta798 |  | 350.104 | 46.023 | 0.010 | 0.010 | 0.011 | 0.002 | 0.011 | 0.003 | 0.023 | 0.010 | 0.008 | 0.010 | 0.005 | 0.000 | 0.008 | 0.012 | 0.007 |
| meta799 |  | 350.866 | 287.705 | 0.016 | 0.012 | 0.013 | 0.020 | 0.017 | 0.015 | 0.018 | 0.017 | 0.018 | 0.017 | 0.015 | 0.016 | 0.019 | 0.017 | 0.015 |
| meta800 |  | 350.992 | 85.413 | 0.007 | 0.007 | 0.007 | 0.010 | 0.010 | 0.008 | 0.010 | 0.005 | 0.006 | 0.006 | 0.007 | 0.003 | 0.007 | 0.006 | 0.009 |
| meta801 |  | 351.002 | 26.681 | 0.041 | 0.041 | 0.043 | 0.054 | 0.005 | 0.040 | 0.016 | 0.078 | 0.359 | 0.041 | 0.090 | 0.025 | 0.003 | 0.010 | 0.010 |
| meta802 |  | 351.008 | 155.975 | 0.004 | 0.003 | 0.003 | 0.003 | 0.003 | 0.002 | 0.003 | 0.003 | 0.002 | 0.002 | 0.002 | 0.002 | 0.002 | 0.002 | 0.004 |
| meta803 |  | 351.038 | 25.272 | 0.031 | 0.045 | 0.048 | 0.054 | 0.044 | 0.060 | 0.067 | 0.103 | 0.079 | 0.108 | 0.059 | 0.022 | 0.035 | 0.046 | 0.017 |
| meta804 | Glucuronolactone | 351.054 | 338.060 | 0.001 | 0.001 | 0.001 | 0.002 | 0.001 | 0.001 | 0.000 | 0.002 | 0.001 | 0.001 | 0.002 | 0.001 | 0.002 | 0.001 | 0.001 |
| meta805 |  | 351.074 | 26.917 | 0.026 | 0.023 | 0.030 | 0.044 | 0.030 | 0.027 | 0.053 | 0.023 | 0.025 | 0.051 | 0.019 | 0.015 | 0.015 | 0.037 | 0.064 |
| meta806 |  | 351.095 | 422.803 | 0.001 | 0.001 | 0.001 | 0.001 | 0.001 | 0.000 | 0.001 | 0.002 | 0.001 | 0.002 | 0.001 | 0.001 | 0.001 | 0.001 | 0.001 |
| meta807 |  | 351.138 | 344.832 | 0.002 | 0.002 | 0.002 | 0.003 | 0.002 | 0.002 | 0.001 | 0.002 | 0.001 | 0.002 | 0.001 | 0.002 | 0.002 | 0.002 | 0.002 |
| meta808 |  | 351.145 | 28.009 | 0.008 | 0.006 | 0.006 | 0.009 | 0.010 | 0.008 | 0.007 | 0.005 | 0.005 | 0.006 | 0.004 | 0.006 | 0.011 | 0.006 | 0.005 |
| meta809 |  | 351.180 | 46.674 | 0.006 | 0.005 | 0.006 | 0.002 | 0.008 | 0.011 | 0.007 | 0.007 | 0.006 | 0.001 | 0.001 | 0.008 | 0.007 | 0.016 | 0.007 |
| meta810 |  | 351.218 | 42.873 | 0.083 | 0.072 | 0.080 | 0.084 | 0.070 | 0.060 | 0.074 | 0.082 | 0.084 | 0.074 | 0.059 | 0.082 | 0.064 | 0.086 | 0.074 |
| meta811 | 2'-Deoxyadenosine 5'-monophosphate (dAMP) | 352.043 | 423.430 | 0.010 | 0.010 | 0.014 | 0.011 | 0.008 | 0.008 | 0.010 | 0.012 | 0.012 | 0.011 | 0.010 | 0.009 | 0.011 | 0.010 | 0.010 |
| meta812 |  | 352.048 | 201.954 | 0.010 | 0.010 | 0.009 | 0.013 | 0.011 | 0.008 | 0.011 | 0.010 | 0.008 | 0.008 | 0.009 | 0.010 | 0.009 | 0.009 | 0.009 |
| meta813 |  | 352.090 | 436.815 | 0.002 | 0.002 | 0.002 | 0.002 | 0.002 | 0.001 | 0.002 | 0.002 | 0.002 | 0.002 | 0.002 | 0.002 | 0.002 | 0.001 | 0.002 |
| meta814 |  | 352.120 | 71.376 | 0.011 | 0.010 | 0.010 | 0.009 | 0.008 | 0.012 | 0.027 | 0.016 | 0.010 | 0.012 | 0.006 | 0.002 | 0.010 | 0.007 | 0.013 |
| meta815 |  | 352.247 | 40.805 | 0.009 | 0.008 | 0.010 | 0.009 | 0.009 | 0.011 | 0.001 | 0.010 | 0.010 | 0.010 | 0.011 | 0.016 | 0.016 | 0.014 | 0.012 |
| meta816 |  | 352.943 | 318.133 | 0.011 | 0.008 | 0.011 | 0.011 | 0.010 | 0.012 | 0.009 | 0.009 | 0.011 | 0.010 | 0.009 | 0.008 | 0.011 | 0.012 | 0.009 |
| meta817 |  | 353.027 | 417.472 | 0.011 | 0.010 | 0.008 | 0.011 | 0.008 | 0.006 | 0.010 | 0.011 | 0.010 | 0.011 | 0.009 | 0.008 | 0.010 | 0.007 | 0.010 |
| meta818 |  | 353.030 | 381.217 | 0.001 | 0.001 | 0.001 | 0.001 | 0.001 | 0.001 | 0.001 | 0.001 | 0.001 | 0.001 | 0.001 | 0.001 | 0.001 | 0.001 | 0.001 |
| meta819 |  | 353.075 | 405.355 | 0.037 | 0.036 | 0.036 | 0.037 | 0.033 | 0.025 | 0.035 | 0.038 | 0.042 | 0.045 | 0.034 | 0.027 | 0.038 | 0.026 | 0.038 |
| meta820 |  | 353.117 | 389.535 | 0.003 | 0.003 | 0.003 | 0.005 | 0.003 | 0.004 | 0.002 | 0.003 | 0.002 | 0.003 | 0.003 | 0.004 | 0.003 | 0.003 | 0.003 |
| meta821 |  | 353.163 | 28.632 | 0.046 | 0.032 | 0.031 | 0.111 | 0.045 | 0.068 | 0.054 | 0.078 | 0.014 | 0.079 | 0.053 | 0.036 | 0.094 | 0.057 | 0.050 |
| meta822 |  | 353.199 | 26.752 | 0.022 | 0.019 | 0.024 | 0.094 | 0.042 | 0.036 | 0.030 | 0.026 | 0.054 | 0.028 | 0.084 | 0.017 | 0.031 | 0.030 | 0.023 |
| meta823 |  | 353.207 | 287.380 | 0.001 | 0.001 | 0.001 | 0.001 | 0.001 | 0.001 | 0.001 | 0.001 | 0.001 | 0.001 | 0.001 | 0.001 | 0.001 | 0.001 | 0.001 |
| meta824 |  | 354.055 | 372.290 | 0.001 | 0.001 | 0.001 | 0.001 | 0.001 | 0.000 | 0.001 | 0.001 | 0.001 | 0.001 | 0.001 | 0.001 | 0.001 | 0.000 | 0.001 |
| meta825 |  | 354.059 | 442.730 | 0.053 | 0.043 | 0.043 | 0.043 | 0.038 | 0.032 | 0.044 | 0.053 | 0.059 | 0.063 | 0.044 | 0.039 | 0.049 | 0.028 | 0.044 |
| meta826 |  | 354.921 | 318.133 | 0.001 | 0.001 | 0.002 | 0.002 | 0.001 | 0.002 | 0.001 | 0.001 | 0.002 | 0.001 | 0.001 | 0.001 | 0.002 | 0.002 | 0.001 |
| meta827 |  | 355.085 | 396.478 | 0.007 | 0.006 | 0.006 | 0.008 | 0.004 | 0.006 | 0.003 | 0.009 | 0.009 | 0.006 | 0.004 | 0.004 | 0.008 | 0.006 | 0.004 |
| meta828 |  | 355.144 | 23.908 | 0.002 | 0.002 | 0.002 | 0.005 | 0.002 | 0.002 | 0.003 | 0.003 | 0.002 | 0.003 | 0.002 | 0.002 | 0.002 | 0.004 | 0.002 |
| meta829 | 5-Oxo-ETE | 355.176 | 41.611 | 0.009 | 0.008 | 0.010 | 0.013 | 0.023 | 0.011 | 0.036 | 0.015 | 0.009 | 0.014 | 0.004 | 0.009 | 0.007 | 0.007 | 0.007 |
| meta830 |  | 355.192 | 32.628 | 0.036 | 0.037 | 0.042 | 0.079 | 0.043 | 0.049 | 0.041 | 0.062 | 0.018 | 0.056 | 0.044 | 0.032 | 0.061 | 0.066 | 0.045 |
| meta831 |  | 355.261 | 38.827 | 0.038 | 0.031 | 0.033 | 0.043 | 0.050 | 0.046 | 0.070 | 0.068 | 0.037 | 0.069 | 0.090 | 0.064 | 0.041 | 0.057 | 0.074 |
| meta832 |  | 355.282 | 33.452 | 0.001 | 0.001 | 0.002 | 0.002 | 0.002 | 0.002 | 0.004 | 0.002 | 0.002 | 0.002 | 0.002 | 0.003 | 0.002 | 0.002 | 0.003 |
| meta833 |  | 355.319 | 45.353 | 0.001 | 0.001 | 0.001 | 0.001 | 0.001 | 0.003 | 0.001 | 0.001 | 0.002 | 0.002 | 0.001 | 0.001 | 0.001 | 0.001 | 0.001 |
| meta834 |  | 356.073 | 157.898 | 0.002 | 0.001 | 0.002 | 0.002 | 0.001 | 0.002 | 0.002 | 0.001 | 0.002 | 0.002 | 0.000 | 0.001 | 0.004 | 0.000 | 0.004 |
| meta835 | Rosiglitazone | 356.107 | 415.474 | 0.003 | 0.003 | 0.002 | 0.003 | 0.003 | 0.003 | 0.002 | 0.003 | 0.001 | 0.002 | 0.004 | 0.004 | 0.002 | 0.003 | 0.003 |
| meta836 |  | 356.151 | 42.561 | 0.043 | 0.046 | 0.050 | 0.107 | 0.054 | 0.077 | 0.093 | 0.077 | 0.083 | 0.064 | 0.034 | 0.031 | 0.019 | 0.025 | 0.014 |
| meta837 |  | 356.216 | 160.599 | 0.009 | 0.009 | 0.009 | 0.017 | 0.006 | 0.018 | 0.011 | 0.004 | 0.002 | 0.008 | 0.004 | 0.010 | 0.008 | 0.007 | 0.008 |
| meta838 |  | 356.900 | 285.822 | 0.002 | 0.001 | 0.001 | 0.002 | 0.001 | 0.001 | 0.001 | 0.002 | 0.001 | 0.001 | 0.001 | 0.001 | 0.001 | 0.001 | 0.001 |
| meta839 |  | 356.980 | 161.849 | 0.003 | 0.003 | 0.005 | 0.004 | 0.004 | 0.003 | 0.004 | 0.002 | 0.005 | 0.002 | 0.001 | 0.003 | 0.004 | 0.004 | 0.005 |
| meta840 |  | 357.096 | 174.324 | 0.007 | 0.007 | 0.006 | 0.011 | 0.010 | 0.008 | 0.009 | 0.007 | 0.007 | 0.011 | 0.006 | 0.004 | 0.008 | 0.006 | 0.008 |
| meta841 |  | 357.262 | 145.935 | 0.002 | 0.002 | 0.002 | 0.002 | 0.001 | 0.003 | 0.003 | 0.003 | 0.004 | 0.002 | 0.002 | 0.001 | 0.001 | 0.001 | 0.000 |
| meta842 |  | 357.997 | 154.674 | 0.003 | 0.003 | 0.002 | 0.003 | 0.003 | 0.003 | 0.003 | 0.002 | 0.002 | 0.002 | 0.002 | 0.003 | 0.003 | 0.003 | 0.002 |
| meta843 |  | 358.167 | 32.544 | 0.019 | 0.020 | 0.023 | 0.029 | 0.037 | 0.024 | 0.025 | 0.037 | 0.026 | 0.030 | 0.012 | 0.019 | 0.012 | 0.022 | 0.009 |
| meta844 |  | 358.204 | 41.427 | 0.007 | 0.007 | 0.007 | 0.010 | 0.013 | 0.009 | 0.014 | 0.017 | 0.008 | 0.009 | 0.010 | 0.010 | 0.007 | 0.011 | 0.012 |
| meta845 |  | 359.042 | 381.377 | 0.003 | 0.003 | 0.003 | 0.004 | 0.004 | 0.002 | 0.003 | 0.004 | 0.003 | 0.003 | 0.004 | 0.003 | 0.003 | 0.003 | 0.003 |
| meta846 | Diosmetin | 359.070 | 361.367 | 0.001 | 0.001 | 0.001 | 0.001 | 0.001 | 0.001 | 0.001 | 0.001 | 0.001 | 0.001 | 0.001 | 0.001 | 0.001 | 0.001 | 0.001 |
| meta847 |  | 360.126 | 279.946 | 0.032 | 0.027 | 0.031 | 0.041 | 0.033 | 0.033 | 0.041 | 0.042 | 0.021 | 0.029 | 0.028 | 0.034 | 0.034 | 0.024 | 0.024 |
| meta848 |  | 360.146 | 45.919 | 0.004 | 0.004 | 0.004 | 0.005 | 0.005 | 0.004 | 0.008 | 0.006 | 0.003 | 0.004 | 0.003 | 0.003 | 0.003 | 0.004 | 0.003 |
| meta849 |  | 360.157 | 178.021 | 0.001 | 0.001 | 0.001 | 0.001 | 0.001 | 0.001 | 0.001 | 0.001 | 0.001 | 0.001 | 0.001 | 0.001 | 0.001 | 0.001 | 0.001 |
| meta850 |  | 360.297 | 38.718 | 0.004 | 0.004 | 0.003 | 0.005 | 0.009 | 0.005 | 0.009 | 0.007 | 0.003 | 0.004 | 0.008 | 0.005 | 0.004 | 0.005 | 0.005 |
| meta851 |  | 360.932 | 580.799 | 0.017 | 0.013 | 0.011 | 0.015 | 0.015 | 0.009 | 0.014 | 0.014 | 0.013 | 0.015 | 0.013 | 0.014 | 0.014 | 0.013 | 0.012 |
| meta852 |  | 361.064 | 431.602 | 0.034 | 0.031 | 0.033 | 0.037 | 0.030 | 0.024 | 0.031 | 0.037 | 0.032 | 0.031 | 0.034 | 0.033 | 0.035 | 0.033 | 0.037 |
| meta853 |  | 361.087 | 482.732 | 0.007 | 0.006 | 0.006 | 0.009 | 0.009 | 0.006 | 0.006 | 0.005 | 0.005 | 0.006 | 0.005 | 0.007 | 0.009 | 0.008 | 0.007 |
| meta854 |  | 361.087 | 43.279 | 0.008 | 0.007 | 0.008 | 0.001 | 0.002 | 0.001 | 0.003 | 0.004 | 0.010 | 0.011 | 0.004 | 0.002 | 0.008 | 0.002 | 0.007 |
| meta855 |  | 361.112 | 35.252 | 0.006 | 0.007 | 0.009 | 0.005 | 0.010 | 0.004 | 0.015 | 0.017 | 0.010 | 0.013 | 0.007 | 0.018 | 0.017 | 0.012 | 0.005 |
| meta856 |  | 361.127 | 174.202 | 0.004 | 0.003 | 0.003 | 0.005 | 0.005 | 0.004 | 0.005 | 0.004 | 0.003 | 0.003 | 0.003 | 0.003 | 0.004 | 0.003 | 0.004 |
| meta857 | L-Tyrosine | 361.134 | 361.350 | 0.001 | 0.000 | 0.000 | 0.001 | 0.000 | 0.000 | 0.000 | 0.000 | 0.000 | 0.000 | 0.001 | 0.001 | 0.000 | 0.000 | 0.001 |
| meta858 |  | 361.200 | 154.687 | 0.023 | 0.021 | 0.021 | 0.014 | 0.064 | 0.010 | 0.033 | 0.027 | 0.004 | 0.014 | 0.021 | 0.010 | 0.014 | 0.012 | 0.032 |
| meta859 | Eicosapentaenoic Acid | 361.235 | 46.577 | 0.021 | 0.018 | 0.017 | 0.019 | 0.023 | 0.014 | 0.038 | 0.027 | 0.016 | 0.015 | 0.023 | 0.027 | 0.028 | 0.021 | 0.020 |
| meta860 | Guanosine 5'-monophosphate (GMP) | 362.048 | 452.246 | 0.017 | 0.015 | 0.018 | 0.019 | 0.019 | 0.017 | 0.018 | 0.023 | 0.020 | 0.019 | 0.015 | 0.020 | 0.025 | 0.016 | 0.017 |
| meta861 |  | 362.111 | 388.241 | 0.002 | 0.003 | 0.003 | 0.002 | 0.001 | 0.002 | 0.001 | 0.002 | 0.003 | 0.003 | 0.002 | 0.003 | 0.003 | 0.002 | 0.002 |
| meta862 |  | 362.234 | 40.727 | 0.042 | 0.037 | 0.044 | 0.054 | 0.070 | 0.053 | 0.060 | 0.102 | 0.050 | 0.080 | 0.092 | 0.046 | 0.042 | 0.114 | 0.047 |
| meta863 |  | 362.951 | 177.360 | 0.006 | 0.007 | 0.007 | 0.010 | 0.008 | 0.006 | 0.012 | 0.007 | 0.007 | 0.006 | 0.004 | 0.004 | 0.006 | 0.005 | 0.006 |
| meta864 |  | 363.001 | 24.842 | 0.012 | 0.014 | 0.014 | 0.033 | 0.001 | 0.026 | 0.006 | 0.046 | 0.020 | 0.027 | 0.013 | 0.002 | 0.004 | 0.009 | 0.002 |
| meta865 |  | 363.017 | 150.367 | 0.002 | 0.002 | 0.001 | 0.002 | 0.002 | 0.001 | 0.002 | 0.002 | 0.001 | 0.001 | 0.002 | 0.001 | 0.002 | 0.002 | 0.002 |
| meta866 |  | 363.030 | 420.923 | 0.004 | 0.003 | 0.003 | 0.004 | 0.004 | 0.003 | 0.003 | 0.004 | 0.004 | 0.005 | 0.003 | 0.003 | 0.006 | 0.003 | 0.003 |
| meta867 | D-Lactose | 363.095 | 378.149 | 0.007 | 0.008 | 0.007 | 0.007 | 0.006 | 0.005 | 0.006 | 0.010 | 0.008 | 0.011 | 0.006 | 0.007 | 0.009 | 0.005 | 0.007 |
| meta868 | N-Acetylaspartylglutamate (NAAG) | 363.100 | 351.226 | 0.002 | 0.002 | 0.002 | 0.003 | 0.002 | 0.002 | 0.002 | 0.002 | 0.002 | 0.002 | 0.002 | 0.002 | 0.002 | 0.001 | 0.002 |
| meta869 | 1,3-Dipropyl-8-cyclopentylxanthine [DPCPX] | 363.207 | 40.337 | 0.010 | 0.008 | 0.015 | 0.009 | 0.014 | 0.008 | 0.012 | 0.008 | 0.007 | 0.009 | 0.008 | 0.010 | 0.007 | 0.009 | 0.006 |
| meta870 |  | 363.215 | 147.082 | 0.003 | 0.004 | 0.004 | 0.002 | 0.010 | 0.002 | 0.005 | 0.005 | 0.001 | 0.002 | 0.003 | 0.002 | 0.004 | 0.001 | 0.006 |
| meta871 |  | 363.216 | 42.004 | 0.010 | 0.015 | 0.011 | 0.000 | 0.000 | 0.020 | 0.017 | 0.030 | 0.015 | 0.022 | 0.023 | 0.019 | 0.002 | 0.032 | 0.017 |
| meta872 |  | 364.079 | 440.178 | 0.006 | 0.005 | 0.005 | 0.005 | 0.006 | 0.006 | 0.006 | 0.006 | 0.005 | 0.006 | 0.004 | 0.006 | 0.007 | 0.005 | 0.005 |
| meta873 |  | 364.083 | 31.605 | 0.028 | 0.023 | 0.035 | 0.040 | 0.022 | 0.006 | 0.029 | 0.052 | 0.054 | 0.010 | 0.015 | 0.008 | 0.027 | 0.031 | 0.006 |
| meta874 |  | 364.177 | 103.995 | 0.013 | 0.015 | 0.013 | 0.016 | 0.019 | 0.015 | 0.018 | 0.016 | 0.006 | 0.008 | 0.003 | 0.003 | 0.005 | 0.011 | 0.002 |
| meta875 |  | 364.996 | 97.442 | 0.003 | 0.003 | 0.003 | 0.000 | 0.000 | 0.000 | 0.010 | 0.009 | 0.005 | 0.003 | 0.001 | 0.001 | 0.003 | 0.000 | 0.001 |
| meta876 |  | 365.041 | 200.684 | 0.002 | 0.002 | 0.003 | 0.003 | 0.002 | 0.002 | 0.003 | 0.002 | 0.002 | 0.002 | 0.002 | 0.002 | 0.002 | 0.003 | 0.002 |
| meta877 |  | 365.038 | 431.928 | 0.001 | 0.001 | 0.001 | 0.001 | 0.001 | 0.001 | 0.001 | 0.001 | 0.001 | 0.001 | 0.002 | 0.001 | 0.001 | 0.001 | 0.001 |
| meta878 | Thiamine monophosphate | 365.054 | 25.825 | 0.002 | 0.002 | 0.002 | 0.007 | 0.002 | 0.004 | 0.003 | 0.002 | 0.002 | 0.001 | 0.007 | 0.001 | 0.002 | 0.001 | 0.001 |
| meta879 |  | 365.160 | 27.382 | 0.009 | 0.008 | 0.008 | 0.011 | 0.010 | 0.007 | 0.007 | 0.008 | 0.007 | 0.006 | 0.007 | 0.007 | 0.008 | 0.006 | 0.008 |
| meta880 | DL-Normetanephrine | 365.180 | 104.355 | 0.004 | 0.003 | 0.003 | 0.005 | 0.006 | 0.003 | 0.004 | 0.004 | 0.002 | 0.002 | 0.003 | 0.001 | 0.002 | 0.003 | 0.002 |
| meta881 |  | 365.230 | 173.673 | 0.005 | 0.005 | 0.005 | 0.002 | 0.019 | 0.001 | 0.005 | 0.004 | 0.001 | 0.001 | 0.004 | 0.003 | 0.006 | 0.001 | 0.009 |
| meta882 | Nervonic acid | 365.341 | 38.578 | 0.004 | 0.003 | 0.002 | 0.002 | 0.003 | 0.001 | 0.003 | 0.004 | 0.003 | 0.002 | 0.007 | 0.004 | 0.003 | 0.004 | 0.004 |
| meta883 |  | 365.975 | 281.906 | 0.003 | 0.003 | 0.003 | 0.004 | 0.003 | 0.003 | 0.004 | 0.003 | 0.002 | 0.003 | 0.003 | 0.003 | 0.002 | 0.001 | 0.003 |
| meta884 |  | 366.099 | 45.241 | 0.013 | 0.013 | 0.012 | 0.013 | 0.010 | 0.023 | 0.020 | 0.017 | 0.017 | 0.013 | 0.009 | 0.011 | 0.010 | 0.024 | 0.009 |
| meta885 |  | 366.963 | 420.822 | 0.005 | 0.004 | 0.003 | 0.005 | 0.005 | 0.002 | 0.004 | 0.006 | 0.005 | 0.005 | 0.005 | 0.006 | 0.010 | 0.004 | 0.003 |
| meta886 |  | 366.996 | 25.140 | 0.004 | 0.004 | 0.005 | 0.007 | 0.006 | 0.005 | 0.002 | 0.024 | 0.023 | 0.010 | 0.013 | 0.003 | 0.006 | 0.001 | 0.004 |
| meta887 |  | 367.032 | 24.493 | 0.004 | 0.005 | 0.005 | 0.009 | 0.006 | 0.007 | 0.011 | 0.011 | 0.012 | 0.011 | 0.025 | 0.003 | 0.006 | 0.005 | 0.003 |
| meta888 |  | 367.042 | 150.952 | 0.006 | 0.006 | 0.006 | 0.008 | 0.008 | 0.006 | 0.007 | 0.009 | 0.005 | 0.005 | 0.005 | 0.004 | 0.005 | 0.006 | 0.005 |
| meta889 |  | 367.090 | 412.479 | 0.002 | 0.002 | 0.002 | 0.001 | 0.001 | 0.001 | 0.001 | 0.004 | 0.003 | 0.005 | 0.001 | 0.003 | 0.002 | 0.003 | 0.001 |
| meta890 |  | 367.133 | 373.466 | 0.003 | 0.002 | 0.002 | 0.004 | 0.002 | 0.003 | 0.002 | 0.002 | 0.001 | 0.002 | 0.003 | 0.003 | 0.002 | 0.002 | 0.002 |
| meta891 |  | 367.169 | 174.259 | 0.006 | 0.006 | 0.006 | 0.006 | 0.005 | 0.007 | 0.007 | 0.005 | 0.007 | 0.006 | 0.006 | 0.006 | 0.007 | 0.006 | 0.006 |
| meta892 |  | 367.214 | 25.175 | 0.005 | 0.005 | 0.005 | 0.021 | 0.010 | 0.008 | 0.006 | 0.005 | 0.006 | 0.005 | 0.004 | 0.005 | 0.007 | 0.005 | 0.004 |
| meta893 | Perindopril | 367.230 | 40.548 | 0.003 | 0.002 | 0.002 | 0.002 | 0.001 | 0.002 | 0.002 | 0.003 | 0.003 | 0.002 | 0.002 | 0.004 | 0.002 | 0.002 | 0.002 |
| meta894 | Tetracosanoic acid | 367.355 | 689.607 | 0.001 | 0.001 | 0.001 | 0.001 | 0.001 | 0.000 | 0.001 | 0.001 | 0.001 | 0.001 | 0.001 | 0.001 | 0.001 | 0.001 | 0.001 |
| meta895 |  | 368.106 | 281.849 | 0.192 | 0.163 | 0.180 | 0.221 | 0.186 | 0.153 | 0.199 | 0.193 | 0.149 | 0.176 | 0.171 | 0.173 | 0.200 | 0.170 | 0.176 |
| meta896 |  | 368.241 | 40.738 | 0.005 | 0.005 | 0.007 | 0.006 | 0.004 | 0.007 | 0.005 | 0.007 | 0.007 | 0.006 | 0.006 | 0.009 | 0.010 | 0.010 | 0.008 |
| meta897 |  | 368.958 | 23.741 | 0.001 | 0.001 | 0.001 | 0.002 | 0.002 | 0.002 | 0.002 | 0.002 | 0.001 | 0.001 | 0.003 | 0.001 | 0.002 | 0.001 | 0.002 |
| meta898 |  | 368.974 | 318.133 | 0.006 | 0.005 | 0.005 | 0.006 | 0.005 | 0.006 | 0.005 | 0.005 | 0.006 | 0.006 | 0.006 | 0.005 | 0.006 | 0.007 | 0.005 |
| meta899 |  | 369.058 | 151.541 | 0.038 | 0.033 | 0.034 | 0.033 | 0.049 | 0.028 | 0.029 | 0.043 | 0.025 | 0.031 | 0.028 | 0.005 | 0.028 | 0.042 | 0.025 |
| meta900 |  | 369.064 | 433.520 | 0.008 | 0.006 | 0.007 | 0.007 | 0.006 | 0.007 | 0.003 | 0.011 | 0.012 | 0.004 | 0.009 | 0.007 | 0.012 | 0.009 | 0.005 |
| meta901 | Androsterone sulfate | 369.171 | 36.076 | 0.015 | 0.014 | 0.014 | 0.022 | 0.013 | 0.016 | 0.014 | 0.023 | 0.004 | 0.020 | 0.018 | 0.011 | 0.022 | 0.025 | 0.016 |
| meta902 | 20-hydroxy-PGF2a | 369.225 | 46.265 | 0.002 | 0.002 | 0.002 | 0.005 | 0.004 | 0.004 | 0.005 | 0.003 | 0.003 | 0.002 | 0.003 | 0.001 | 0.002 | 0.001 | 0.001 |
| meta903 | 6-Keto-PGF1a | 369.225 | 189.492 | 0.001 | 0.000 | 0.001 | 0.000 | 0.000 | 0.000 | 0.000 | 0.001 | 0.000 | 0.000 | 0.000 | 0.000 | 0.000 | 0.003 | 0.000 |
| meta904 | 3-Phospho-D-glycerate | 370.974 | 192.974 | 0.001 | 0.000 | 0.001 | 0.001 | 0.001 | 0.000 | 0.001 | 0.000 | 0.000 | 0.000 | 0.001 | 0.001 | 0.001 | 0.000 | 0.000 |
| meta905 |  | 371.035 | 334.622 | 0.010 | 0.009 | 0.008 | 0.012 | 0.010 | 0.007 | 0.007 | 0.012 | 0.008 | 0.011 | 0.009 | 0.007 | 0.009 | 0.009 | 0.010 |
| meta906 |  | 371.204 | 160.598 | 0.014 | 0.014 | 0.012 | 0.017 | 0.014 | 0.015 | 0.015 | 0.014 | 0.026 | 0.010 | 0.008 | 0.016 | 0.004 | 0.015 | 0.003 |
| meta907 |  | 371.241 | 103.008 | 0.018 | 0.014 | 0.023 | 0.023 | 0.029 | 0.015 | 0.032 | 0.014 | 0.017 | 0.013 | 0.013 | 0.022 | 0.008 | 0.009 | 0.011 |
| meta908 | beta-Nicotinamide D-ribonucleotide | 372.008 | 154.024 | 0.000 | 0.000 | 0.000 | 0.001 | 0.001 | 0.000 | 0.000 | 0.000 | 0.000 | 0.001 | 0.000 | 0.000 | 0.000 | 0.000 | 0.000 |
| meta909 |  | 372.182 | 43.916 | 0.046 | 0.042 | 0.045 | 0.080 | 0.036 | 0.063 | 0.071 | 0.044 | 0.102 | 0.049 | 0.026 | 0.038 | 0.037 | 0.081 | 0.009 |
| meta910 |  | 373.036 | 397.321 | 0.001 | 0.001 | 0.001 | 0.001 | 0.001 | 0.001 | 0.001 | 0.001 | 0.001 | 0.002 | 0.001 | 0.001 | 0.002 | 0.001 | 0.001 |
| meta911 |  | 373.052 | 157.980 | 0.017 | 0.014 | 0.015 | 0.010 | 0.009 | 0.009 | 0.007 | 0.005 | 0.011 | 0.004 | 0.007 | 0.019 | 0.005 | 0.006 | 0.004 |
| meta912 |  | 373.129 | 32.729 | 0.005 | 0.004 | 0.005 | 0.004 | 0.003 | 0.003 | 0.002 | 0.004 | 0.005 | 0.003 | 0.006 | 0.007 | 0.005 | 0.005 | 0.011 |
| meta913 | 6k-PGF1alpha-d4 | 373.257 | 104.055 | 0.021 | 0.020 | 0.019 | 0.047 | 0.074 | 0.035 | 0.018 | 0.028 | 0.013 | 0.028 | 0.016 | 0.026 | 0.017 | 0.023 | 0.008 |
| meta914 |  | 374.034 | 273.874 | 0.014 | 0.011 | 0.012 | 0.016 | 0.017 | 0.014 | 0.010 | 0.019 | 0.011 | 0.019 | 0.011 | 0.006 | 0.013 | 0.014 | 0.012 |
| meta915 |  | 374.064 | 411.137 | 0.040 | 0.029 | 0.027 | 0.032 | 0.031 | 0.023 | 0.018 | 0.054 | 0.028 | 0.080 | 0.056 | 0.045 | 0.033 | 0.018 | 0.026 |
| meta916 |  | 374.083 | 363.273 | 0.011 | 0.010 | 0.009 | 0.014 | 0.012 | 0.008 | 0.011 | 0.013 | 0.005 | 0.011 | 0.015 | 0.010 | 0.007 | 0.007 | 0.010 |
| meta917 |  | 374.242 | 32.897 | 0.019 | 0.020 | 0.021 | 0.054 | 0.024 | 0.016 | 0.026 | 0.024 | 0.025 | 0.019 | 0.024 | 0.030 | 0.020 | 0.024 | 0.024 |
| meta918 |  | 374.770 | 289.082 | 0.004 | 0.005 | 0.005 | 0.006 | 0.005 | 0.004 | 0.005 | 0.005 | 0.006 | 0.005 | 0.004 | 0.004 | 0.006 | 0.005 | 0.005 |
| meta919 |  | 375.007 | 285.121 | 0.017 | 0.012 | 0.010 | 0.018 | 0.017 | 0.011 | 0.017 | 0.016 | 0.010 | 0.013 | 0.014 | 0.014 | 0.014 | 0.010 | 0.011 |
| meta920 |  | 375.016 | 381.217 | 0.001 | 0.001 | 0.001 | 0.001 | 0.001 | 0.001 | 0.001 | 0.001 | 0.001 | 0.001 | 0.001 | 0.001 | 0.001 | 0.001 | 0.001 |
| meta921 |  | 375.106 | 174.478 | 0.007 | 0.006 | 0.006 | 0.009 | 0.008 | 0.007 | 0.007 | 0.007 | 0.006 | 0.011 | 0.005 | 0.004 | 0.007 | 0.006 | 0.007 |
| meta922 | Riboflavin | 375.128 | 195.537 | 0.001 | 0.001 | 0.001 | 0.001 | 0.001 | 0.001 | 0.001 | 0.001 | 0.001 | 0.001 | 0.001 | 0.001 | 0.001 | 0.001 | 0.001 |
| meta923 | 3-Hydroxycapric acid | 375.272 | 80.566 | 0.020 | 0.019 | 0.011 | 0.047 | 0.025 | 0.016 | 0.053 | 0.028 | 0.048 | 0.027 | 0.029 | 0.011 | 0.013 | 0.015 | 0.014 |
| meta924 |  | 376.040 | 442.741 | 0.003 | 0.003 | 0.003 | 0.003 | 0.003 | 0.002 | 0.003 | 0.003 | 0.003 | 0.004 | 0.003 | 0.003 | 0.003 | 0.002 | 0.003 |
| meta925 |  | 376.098 | 396.098 | 0.031 | 0.026 | 0.026 | 0.035 | 0.034 | 0.022 | 0.029 | 0.037 | 0.018 | 0.034 | 0.035 | 0.026 | 0.025 | 0.017 | 0.024 |
| meta926 |  | 376.116 | 357.891 | 0.002 | 0.001 | 0.001 | 0.001 | 0.003 | 0.001 | 0.003 | 0.001 | 0.001 | 0.001 | 0.001 | 0.001 | 0.002 | 0.001 | 0.001 |
| meta927 |  | 376.133 | 372.047 | 0.014 | 0.011 | 0.010 | 0.012 | 0.012 | 0.008 | 0.013 | 0.013 | 0.013 | 0.013 | 0.015 | 0.010 | 0.015 | 0.012 | 0.009 |
| meta928 |  | 376.863 | 63.643 | 0.001 | 0.002 | 0.002 | 0.002 | 0.002 | 0.002 | 0.002 | 0.002 | 0.002 | 0.002 | 0.002 | 0.002 | 0.002 | 0.001 | 0.002 |
| meta929 |  | 376.904 | 318.133 | 0.007 | 0.006 | 0.008 | 0.008 | 0.006 | 0.008 | 0.007 | 0.006 | 0.008 | 0.007 | 0.006 | 0.006 | 0.008 | 0.009 | 0.006 |
| meta930 |  | 377.064 | 427.602 | 0.087 | 0.071 | 0.074 | 0.089 | 0.073 | 0.048 | 0.050 | 0.123 | 0.064 | 0.146 | 0.112 | 0.103 | 0.066 | 0.050 | 0.077 |
| meta931 |  | 377.107 | 46.314 | 0.004 | 0.004 | 0.004 | 0.002 | 0.003 | 0.002 | 0.003 | 0.004 | 0.002 | 0.005 | 0.004 | 0.007 | 0.010 | 0.006 | 0.003 |
| meta932 |  | 377.233 | 40.729 | 0.020 | 0.026 | 0.028 | 0.026 | 0.017 | 0.023 | 0.021 | 0.034 | 0.020 | 0.028 | 0.016 | 0.023 | 0.028 | 0.026 | 0.027 |
| meta933 |  | 377.268 | 33.606 | 0.001 | 0.001 | 0.001 | 0.001 | 0.001 | 0.001 | 0.001 | 0.001 | 0.001 | 0.001 | 0.001 | 0.001 | 0.001 | 0.002 | 0.001 |
| meta934 | S-Lactoylglutathione | 378.095 | 376.175 | 0.007 | 0.007 | 0.007 | 0.009 | 0.022 | 0.005 | 0.009 | 0.003 | 0.006 | 0.007 | 0.004 | 0.004 | 0.007 | 0.006 | 0.006 |
| meta935 |  | 378.148 | 367.750 | 0.002 | 0.002 | 0.002 | 0.003 | 0.002 | 0.002 | 0.002 | 0.002 | 0.001 | 0.002 | 0.001 | 0.002 | 0.003 | 0.002 | 0.003 |
| meta936 |  | 378.883 | 318.126 | 0.002 | 0.002 | 0.003 | 0.002 | 0.002 | 0.003 | 0.002 | 0.002 | 0.003 | 0.002 | 0.002 | 0.002 | 0.003 | 0.003 | 0.002 |
| meta937 |  | 378.991 | 354.789 | 0.001 | 0.001 | 0.001 | 0.001 | 0.001 | 0.001 | 0.001 | 0.001 | 0.001 | 0.001 | 0.001 | 0.001 | 0.001 | 0.001 | 0.001 |
| meta938 |  | 378.986 | 25.061 | 0.002 | 0.001 | 0.001 | 0.002 | 0.001 | 0.001 | 0.001 | 0.003 | 0.003 | 0.002 | 0.001 | 0.001 | 0.001 | 0.003 | 0.000 |
| meta939 |  | 379.013 | 85.394 | 0.077 | 0.069 | 0.071 | 0.107 | 0.108 | 0.074 | 0.094 | 0.067 | 0.059 | 0.092 | 0.077 | 0.052 | 0.070 | 0.043 | 0.070 |
| meta940 |  | 379.053 | 199.433 | 0.002 | 0.002 | 0.002 | 0.001 | 0.003 | 0.001 | 0.002 | 0.000 | 0.001 | 0.002 | 0.000 | 0.001 | 0.002 | 0.001 | 0.002 |
| meta941 |  | 379.052 | 24.505 | 0.010 | 0.013 | 0.014 | 0.025 | 0.007 | 0.019 | 0.025 | 0.013 | 0.024 | 0.017 | 0.011 | 0.017 | 0.010 | 0.021 | 0.011 |
| meta942 |  | 379.090 | 405.355 | 0.006 | 0.005 | 0.006 | 0.005 | 0.005 | 0.004 | 0.005 | 0.006 | 0.006 | 0.008 | 0.006 | 0.004 | 0.007 | 0.004 | 0.007 |
| meta943 |  | 379.098 | 154.619 | 0.005 | 0.004 | 0.004 | 0.005 | 0.008 | 0.005 | 0.010 | 0.004 | 0.002 | 0.002 | 0.003 | 0.006 | 0.003 | 0.004 | 0.003 |
| meta944 |  | 379.175 | 26.917 | 0.006 | 0.005 | 0.005 | 0.006 | 0.007 | 0.005 | 0.007 | 0.007 | 0.004 | 0.008 | 0.005 | 0.005 | 0.006 | 0.006 | 0.007 |
| meta945 |  | 379.210 | 199.621 | 0.002 | 0.001 | 0.002 | 0.001 | 0.002 | 0.001 | 0.005 | 0.002 | 0.001 | 0.001 | 0.002 | 0.002 | 0.001 | 0.001 | 0.001 |
| meta946 |  | 379.249 | 56.146 | 0.018 | 0.013 | 0.014 | 0.021 | 0.019 | 0.010 | 0.012 | 0.017 | 0.017 | 0.016 | 0.017 | 0.014 | 0.017 | 0.017 | 0.015 |
| meta947 |  | 380.128 | 399.416 | 0.001 | 0.001 | 0.001 | 0.001 | 0.001 | 0.001 | 0.001 | 0.001 | 0.000 | 0.001 | 0.001 | 0.001 | 0.001 | 0.001 | 0.001 |
| meta948 |  | 380.902 | 613.886 | 0.043 | 0.031 | 0.038 | 0.049 | 0.013 | 0.027 | 0.035 | 0.052 | 0.011 | 0.043 | 0.072 | 0.016 | 0.069 | 0.016 | 0.056 |
| meta949 |  | 381.084 | 27.973 | 0.009 | 0.007 | 0.010 | 0.015 | 0.004 | 0.011 | 0.010 | 0.008 | 0.012 | 0.014 | 0.009 | 0.008 | 0.003 | 0.008 | 0.004 |
| meta950 |  | 381.148 | 356.426 | 0.001 | 0.001 | 0.001 | 0.002 | 0.001 | 0.001 | 0.001 | 0.001 | 0.001 | 0.001 | 0.001 | 0.001 | 0.001 | 0.001 | 0.001 |
| meta951 |  | 381.214 | 25.668 | 0.011 | 0.009 | 0.008 | 0.015 | 0.015 | 0.014 | 0.012 | 0.013 | 0.012 | 0.024 | 0.011 | 0.010 | 0.009 | 0.010 | 0.017 |
| meta952 |  | 381.225 | 239.280 | 0.001 | 0.002 | 0.002 | 0.001 | 0.002 | 0.001 | 0.003 | 0.003 | 0.001 | 0.001 | 0.002 | 0.001 | 0.002 | 0.001 | 0.002 |
| meta953 |  | 381.238 | 287.701 | 0.001 | 0.001 | 0.001 | 0.001 | 0.001 | 0.001 | 0.001 | 0.001 | 0.001 | 0.001 | 0.001 | 0.001 | 0.001 | 0.001 | 0.001 |
| meta954 |  | 382.042 | 196.571 | 0.004 | 0.003 | 0.003 | 0.005 | 0.001 | 0.004 | 0.001 | 0.003 | 0.001 | 0.005 | 0.004 | 0.003 | 0.002 | 0.001 | 0.007 |
| meta955 |  | 382.097 | 394.053 | 0.010 | 0.008 | 0.009 | 0.009 | 0.013 | 0.007 | 0.006 | 0.009 | 0.008 | 0.013 | 0.006 | 0.008 | 0.013 | 0.006 | 0.009 |
| meta956 |  | 382.097 | 479.080 | 0.001 | 0.001 | 0.001 | 0.002 | 0.002 | 0.001 | 0.001 | 0.001 | 0.001 | 0.001 | 0.001 | 0.001 | 0.002 | 0.001 | 0.001 |
| meta957 |  | 382.169 | 282.163 | 0.001 | 0.001 | 0.001 | 0.000 | 0.001 | 0.000 | 0.001 | 0.001 | 0.000 | 0.000 | 0.000 | 0.000 | 0.001 | 0.000 | 0.001 |
| meta958 |  | 382.992 | 429.499 | 0.005 | 0.004 | 0.004 | 0.005 | 0.005 | 0.003 | 0.004 | 0.006 | 0.005 | 0.004 | 0.005 | 0.005 | 0.004 | 0.004 | 0.005 |
| meta959 |  | 383.011 | 157.919 | 0.001 | 0.001 | 0.000 | 0.001 | 0.001 | 0.001 | 0.001 | 0.001 | 0.001 | 0.001 | 0.001 | 0.001 | 0.001 | 0.000 | 0.001 |
| meta960 | S-Adenosyl-L-homocysteine | 383.111 | 362.643 | 0.025 | 0.025 | 0.023 | 0.029 | 0.029 | 0.023 | 0.023 | 0.030 | 0.024 | 0.029 | 0.024 | 0.024 | 0.027 | 0.020 | 0.021 |
| meta961 |  | 383.116 | 428.016 | 0.006 | 0.006 | 0.006 | 0.007 | 0.006 | 0.008 | 0.006 | 0.008 | 0.004 | 0.007 | 0.006 | 0.009 | 0.006 | 0.008 | 0.003 |
| meta962 |  | 383.125 | 27.337 | 0.003 | 0.003 | 0.002 | 0.003 | 0.004 | 0.002 | 0.002 | 0.002 | 0.002 | 0.002 | 0.003 | 0.002 | 0.003 | 0.002 | 0.002 |
| meta963 |  | 383.289 | 142.224 | 0.000 | 0.000 | 0.000 | 0.000 | 0.000 | 0.000 | 0.000 | 0.000 | 0.001 | 0.000 | 0.001 | 0.000 | 0.000 | 0.001 | 0.000 |
| meta964 |  | 384.032 | 417.837 | 0.001 | 0.001 | 0.001 | 0.001 | 0.001 | 0.001 | 0.001 | 0.001 | 0.001 | 0.002 | 0.001 | 0.001 | 0.001 | 0.001 | 0.001 |
| meta965 |  | 384.095 | 329.048 | 0.002 | 0.002 | 0.002 | 0.001 | 0.001 | 0.000 | 0.003 | 0.009 | 0.003 | 0.002 | 0.003 | 0.004 | 0.002 | 0.001 | 0.001 |
| meta966 |  | 384.218 | 40.728 | 0.010 | 0.008 | 0.009 | 0.014 | 0.017 | 0.012 | 0.014 | 0.019 | 0.015 | 0.028 | 0.019 | 0.010 | 0.009 | 0.024 | 0.016 |
| meta967 |  | 384.974 | 420.805 | 0.005 | 0.003 | 0.003 | 0.004 | 0.005 | 0.003 | 0.004 | 0.005 | 0.004 | 0.005 | 0.005 | 0.005 | 0.008 | 0.003 | 0.003 |
| meta968 | XMP | 385.010 | 424.913 | 0.001 | 0.001 | 0.001 | 0.001 | 0.001 | 0.001 | 0.001 | 0.001 | 0.002 | 0.001 | 0.001 | 0.001 | 0.001 | 0.001 | 0.001 |
| meta969 |  | 385.130 | 43.259 | 0.013 | 0.013 | 0.012 | 0.022 | 0.007 | 0.016 | 0.012 | 0.007 | 0.017 | 0.007 | 0.012 | 0.020 | 0.008 | 0.014 | 0.004 |
| meta970 |  | 385.184 | 197.155 | 0.000 | 0.000 | 0.000 | 0.001 | 0.000 | 0.001 | 0.001 | 0.001 | 0.001 | 0.001 | 0.000 | 0.000 | 0.000 | 0.000 | 0.000 |
| meta971 |  | 385.220 | 154.134 | 0.022 | 0.020 | 0.021 | 0.029 | 0.031 | 0.027 | 0.036 | 0.035 | 0.040 | 0.013 | 0.010 | 0.021 | 0.006 | 0.021 | 0.005 |
| meta972 |  | 386.031 | 402.560 | 0.003 | 0.003 | 0.003 | 0.003 | 0.002 | 0.002 | 0.003 | 0.004 | 0.004 | 0.005 | 0.003 | 0.003 | 0.004 | 0.002 | 0.003 |
| meta973 |  | 386.064 | 29.770 | 0.000 | 0.000 | 0.001 | 0.001 | 0.004 | 0.005 | 0.000 | 0.009 | 0.001 | 0.007 | 0.001 | 0.004 | 0.007 | 0.008 | 0.005 |
| meta974 |  | 386.146 | 26.917 | 0.004 | 0.004 | 0.004 | 0.009 | 0.005 | 0.005 | 0.005 | 0.008 | 0.003 | 0.007 | 0.005 | 0.004 | 0.007 | 0.007 | 0.004 |
| meta975 |  | 386.234 | 40.108 | 0.073 | 0.077 | 0.077 | 0.094 | 0.116 | 0.086 | 0.078 | 0.144 | 0.132 | 0.220 | 0.189 | 0.071 | 0.078 | 0.158 | 0.119 |
| meta976 |  | 386.924 | 423.128 | 0.002 | 0.001 | 0.001 | 0.002 | 0.001 | 0.001 | 0.001 | 0.001 | 0.001 | 0.001 | 0.001 | 0.001 | 0.001 | 0.001 | 0.001 |
| meta977 |  | 387.085 | 26.563 | 0.003 | 0.002 | 0.002 | 0.006 | 0.001 | 0.004 | 0.001 | 0.007 | 0.010 | 0.005 | 0.001 | 0.002 | 0.001 | 0.004 | 0.001 |
| meta978 |  | 387.111 | 346.223 | 0.015 | 0.013 | 0.014 | 0.014 | 0.013 | 0.019 | 0.014 | 0.015 | 0.010 | 0.012 | 0.014 | 0.018 | 0.012 | 0.011 | 0.007 |
| meta979 |  | 387.200 | 221.666 | 0.003 | 0.003 | 0.003 | 0.005 | 0.001 | 0.005 | 0.005 | 0.002 | 0.004 | 0.005 | 0.001 | 0.000 | 0.000 | 0.001 | 0.000 |
| meta980 |  | 388.011 | 371.752 | 0.002 | 0.002 | 0.002 | 0.002 | 0.002 | 0.001 | 0.002 | 0.002 | 0.001 | 0.002 | 0.002 | 0.002 | 0.002 | 0.001 | 0.002 |
| meta981 |  | 388.092 | 413.717 | 0.007 | 0.005 | 0.006 | 0.006 | 0.006 | 0.005 | 0.006 | 0.006 | 0.005 | 0.006 | 0.007 | 0.007 | 0.004 | 0.006 | 0.004 |
| meta982 |  | 388.212 | 42.131 | 0.018 | 0.018 | 0.018 | 0.043 | 0.056 | 0.036 | 0.029 | 0.023 | 0.031 | 0.020 | 0.013 | 0.011 | 0.012 | 0.017 | 0.004 |
| meta983 |  | 388.908 | 287.387 | 0.001 | 0.001 | 0.001 | 0.001 | 0.001 | 0.001 | 0.001 | 0.001 | 0.001 | 0.001 | 0.001 | 0.001 | 0.001 | 0.001 | 0.001 |
| meta984 |  | 388.962 | 155.287 | 0.000 | 0.000 | 0.000 | 0.000 | 0.000 | 0.000 | 0.000 | 0.000 | 0.000 | 0.000 | 0.000 | 0.000 | 0.000 | 0.000 | 0.000 |
| meta985 |  | 389.150 | 174.713 | 0.004 | 0.003 | 0.003 | 0.006 | 0.005 | 0.005 | 0.004 | 0.003 | 0.005 | 0.005 | 0.003 | 0.003 | 0.004 | 0.005 | 0.004 |
| meta986 |  | 389.158 | 45.161 | 0.004 | 0.003 | 0.003 | 0.013 | 0.000 | 0.011 | 0.015 | 0.001 | 0.009 | 0.009 | 0.008 | 0.003 | 0.002 | 0.003 | 0.013 |
| meta987 |  | 389.243 | 33.677 | 0.006 | 0.005 | 0.005 | 0.009 | 0.009 | 0.007 | 0.008 | 0.008 | 0.007 | 0.009 | 0.010 | 0.010 | 0.012 | 0.011 | 0.007 |
| meta988 |  | 389.287 | 34.016 | 0.004 | 0.005 | 0.005 | 0.010 | 0.007 | 0.007 | 0.008 | 0.007 | 0.006 | 0.009 | 0.007 | 0.006 | 0.006 | 0.009 | 0.006 |
| meta989 |  | 390.032 | 333.924 | 0.005 | 0.004 | 0.004 | 0.006 | 0.004 | 0.004 | 0.003 | 0.006 | 0.003 | 0.006 | 0.004 | 0.003 | 0.004 | 0.004 | 0.003 |
| meta990 |  | 390.265 | 39.554 | 0.011 | 0.012 | 0.010 | 0.016 | 0.022 | 0.012 | 0.019 | 0.031 | 0.012 | 0.017 | 0.017 | 0.011 | 0.011 | 0.025 | 0.019 |
| meta991 |  | 390.801 | 288.540 | 0.003 | 0.003 | 0.003 | 0.004 | 0.004 | 0.004 | 0.004 | 0.003 | 0.004 | 0.004 | 0.003 | 0.004 | 0.004 | 0.004 | 0.003 |
| meta992 |  | 390.988 | 420.167 | 0.002 | 0.001 | 0.001 | 0.002 | 0.002 | 0.001 | 0.002 | 0.003 | 0.002 | 0.004 | 0.002 | 0.002 | 0.003 | 0.001 | 0.002 |
| meta993 |  | 391.040 | 151.483 | 0.004 | 0.005 | 0.005 | 0.004 | 0.005 | 0.004 | 0.003 | 0.006 | 0.004 | 0.004 | 0.004 | 0.001 | 0.005 | 0.006 | 0.003 |
| meta994 | Deoxycholic acid | 391.283 | 140.285 | 0.006 | 0.006 | 0.005 | 0.006 | 0.005 | 0.005 | 0.006 | 0.006 | 0.005 | 0.009 | 0.008 | 0.008 | 0.005 | 0.005 | 0.006 |
| meta995 |  | 392.069 | 481.402 | 0.007 | 0.007 | 0.007 | 0.008 | 0.008 | 0.007 | 0.007 | 0.008 | 0.008 | 0.007 | 0.006 | 0.007 | 0.009 | 0.007 | 0.006 |
| meta996 |  | 392.192 | 160.598 | 0.003 | 0.002 | 0.002 | 0.004 | 0.001 | 0.004 | 0.003 | 0.001 | 0.000 | 0.001 | 0.001 | 0.002 | 0.001 | 0.002 | 0.002 |
| meta997 |  | 392.877 | 287.086 | 0.024 | 0.018 | 0.019 | 0.032 | 0.030 | 0.024 | 0.029 | 0.027 | 0.029 | 0.026 | 0.023 | 0.026 | 0.032 | 0.026 | 0.023 |
| meta998 |  | 392.935 | 318.126 | 0.005 | 0.005 | 0.006 | 0.006 | 0.005 | 0.006 | 0.005 | 0.005 | 0.006 | 0.005 | 0.005 | 0.004 | 0.006 | 0.006 | 0.005 |
| meta999 |  | 393.047 | 25.610 | 0.034 | 0.038 | 0.037 | 0.067 | 0.022 | 0.051 | 0.084 | 0.036 | 0.039 | 0.049 | 0.051 | 0.033 | 0.033 | 0.039 | 0.049 |
| meta1000 |  | 393.058 | 471.311 | 0.016 | 0.015 | 0.016 | 0.019 | 0.019 | 0.016 | 0.012 | 0.025 | 0.017 | 0.035 | 0.016 | 0.022 | 0.020 | 0.015 | 0.014 |
| meta1001 | Pioglitazone | 393.072 | 481.359 | 0.001 | 0.001 | 0.001 | 0.001 | 0.001 | 0.001 | 0.001 | 0.001 | 0.001 | 0.001 | 0.001 | 0.001 | 0.001 | 0.001 | 0.001 |
| meta1002 |  | 393.261 | 215.770 | 0.001 | 0.001 | 0.001 | 0.001 | 0.000 | 0.002 | 0.001 | 0.000 | 0.000 | 0.000 | 0.000 | 0.000 | 0.000 | 0.000 | 0.001 |
| meta1003 |  | 394.100 | 387.383 | 0.001 | 0.001 | 0.001 | 0.001 | 0.001 | 0.001 | 0.001 | 0.001 | 0.001 | 0.001 | 0.001 | 0.001 | 0.001 | 0.001 | 0.001 |
| meta1004 |  | 394.144 | 389.531 | 0.001 | 0.001 | 0.001 | 0.002 | 0.001 | 0.001 | 0.001 | 0.001 | 0.001 | 0.001 | 0.001 | 0.001 | 0.001 | 0.001 | 0.001 |
| meta1005 | Ketanserin | 394.167 | 43.278 | 0.023 | 0.016 | 0.018 | 0.040 | 0.023 | 0.031 | 0.030 | 0.023 | 0.038 | 0.024 | 0.018 | 0.015 | 0.019 | 0.028 | 0.004 |
| meta1006 |  | 394.264 | 181.408 | 0.002 | 0.002 | 0.001 | 0.000 | 0.000 | 0.000 | 0.002 | 0.001 | 0.000 | 0.000 | 0.002 | 0.010 | 0.003 | 0.002 | 0.004 |
| meta1007 | Hexacosanoic acid | 395.387 | 7.730 | 0.000 | 0.001 | 0.001 | 0.001 | 0.000 | 0.000 | 0.000 | 0.001 | 0.000 | 0.000 | 0.000 | 0.001 | 0.001 | 0.000 | 0.001 |
| meta1008 |  | 396.006 | 274.149 | 0.002 | 0.002 | 0.002 | 0.003 | 0.002 | 0.002 | 0.002 | 0.002 | 0.001 | 0.002 | 0.001 | 0.002 | 0.003 | 0.002 | 0.002 |
| meta1009 |  | 396.068 | 428.849 | 0.006 | 0.004 | 0.005 | 0.005 | 0.005 | 0.003 | 0.005 | 0.006 | 0.005 | 0.007 | 0.005 | 0.005 | 0.007 | 0.004 | 0.006 |
| meta1010 |  | 396.111 | 403.582 | 0.001 | 0.001 | 0.001 | 0.001 | 0.001 | 0.001 | 0.001 | 0.002 | 0.001 | 0.001 | 0.001 | 0.001 | 0.002 | 0.001 | 0.001 |
| meta1011 |  | 396.113 | 94.575 | 0.003 | 0.003 | 0.003 | 0.005 | 0.005 | 0.003 | 0.003 | 0.003 | 0.001 | 0.002 | 0.003 | 0.002 | 0.002 | 0.003 | 0.003 |
| meta1012 |  | 396.128 | 27.852 | 0.012 | 0.010 | 0.010 | 0.013 | 0.010 | 0.009 | 0.009 | 0.011 | 0.010 | 0.009 | 0.009 | 0.009 | 0.011 | 0.008 | 0.010 |
| meta1013 |  | 396.835 | 288.327 | 0.006 | 0.005 | 0.006 | 0.007 | 0.005 | 0.006 | 0.007 | 0.006 | 0.006 | 0.006 | 0.006 | 0.005 | 0.006 | 0.006 | 0.006 |
| meta1014 |  | 396.855 | 285.323 | 0.000 | 0.000 | 0.000 | 0.001 | 0.000 | 0.001 | 0.001 | 0.001 | 0.000 | 0.001 | 0.000 | 0.001 | 0.001 | 0.000 | 0.001 |
| meta1015 |  | 396.862 | 578.359 | 0.059 | 0.051 | 0.047 | 0.067 | 0.056 | 0.041 | 0.055 | 0.056 | 0.050 | 0.055 | 0.055 | 0.051 | 0.053 | 0.049 | 0.049 |
| meta1016 |  | 397.001 | 355.018 | 0.002 | 0.001 | 0.001 | 0.002 | 0.002 | 0.001 | 0.001 | 0.002 | 0.001 | 0.002 | 0.002 | 0.001 | 0.001 | 0.001 | 0.002 |
| meta1017 |  | 397.175 | 157.947 | 0.001 | 0.001 | 0.001 | 0.001 | 0.004 | 0.001 | 0.002 | 0.002 | 0.000 | 0.001 | 0.001 | 0.001 | 0.001 | 0.001 | 0.002 |
| meta1018 | Sunitinib | 397.208 | 26.318 | 0.013 | 0.010 | 0.014 | 0.028 | 0.014 | 0.022 | 0.019 | 0.017 | 0.016 | 0.021 | 0.012 | 0.014 | 0.012 | 0.012 | 0.021 |
| meta1019 |  | 397.220 | 306.782 | 0.001 | 0.001 | 0.000 | 0.000 | 0.001 | 0.000 | 0.001 | 0.001 | 0.000 | 0.001 | 0.001 | 0.001 | 0.001 | 0.000 | 0.001 |
| meta1020 |  | 397.985 | 381.989 | 0.001 | 0.001 | 0.001 | 0.001 | 0.001 | 0.001 | 0.001 | 0.001 | 0.001 | 0.001 | 0.001 | 0.001 | 0.001 | 0.001 | 0.001 |
| meta1021 |  | 398.151 | 352.124 | 0.002 | 0.002 | 0.002 | 0.002 | 0.002 | 0.001 | 0.002 | 0.002 | 0.002 | 0.002 | 0.002 | 0.002 | 0.002 | 0.001 | 0.002 |
| meta1022 |  | 398.871 | 50.386 | 0.000 | 0.001 | 0.000 | 0.001 | 0.000 | 0.000 | 0.000 | 0.000 | 0.000 | 0.000 | 0.000 | 0.000 | 0.000 | 0.000 | 0.000 |
| meta1023 | Alpha-D-Glucose 1,6-bisphosphate | 399.006 | 432.581 | 0.001 | 0.001 | 0.001 | 0.001 | 0.001 | 0.001 | 0.001 | 0.001 | 0.001 | 0.002 | 0.002 | 0.002 | 0.002 | 0.002 | 0.001 |
| meta1024 |  | 399.034 | 23.742 | 0.002 | 0.003 | 0.003 | 0.005 | 0.001 | 0.004 | 0.005 | 0.002 | 0.004 | 0.004 | 0.002 | 0.003 | 0.002 | 0.005 | 0.002 |
| meta1025 |  | 399.272 | 33.727 | 0.001 | 0.002 | 0.001 | 0.003 | 0.002 | 0.001 | 0.002 | 0.003 | 0.002 | 0.002 | 0.003 | 0.003 | 0.004 | 0.003 | 0.002 |
| meta1026 |  | 400.143 | 398.116 | 0.006 | 0.005 | 0.005 | 0.008 | 0.006 | 0.005 | 0.006 | 0.005 | 0.003 | 0.004 | 0.004 | 0.005 | 0.005 | 0.004 | 0.005 |
| meta1027 |  | 400.161 | 26.759 | 0.008 | 0.007 | 0.009 | 0.020 | 0.010 | 0.013 | 0.008 | 0.017 | 0.002 | 0.016 | 0.010 | 0.008 | 0.014 | 0.014 | 0.010 |
| meta1028 |  | 400.213 | 42.007 | 0.113 | 0.109 | 0.112 | 0.315 | 0.108 | 0.249 | 0.226 | 0.166 | 0.152 | 0.119 | 0.080 | 0.083 | 0.091 | 0.125 | 0.032 |
| meta1029 |  | 400.865 | 318.133 | 0.006 | 0.005 | 0.006 | 0.006 | 0.005 | 0.007 | 0.006 | 0.005 | 0.007 | 0.005 | 0.005 | 0.005 | 0.007 | 0.007 | 0.005 |
| meta1030 |  | 400.982 | 25.038 | 0.002 | 0.003 | 0.003 | 0.002 | 0.002 | 0.002 | 0.002 | 0.005 | 0.003 | 0.006 | 0.003 | 0.001 | 0.002 | 0.002 | 0.002 |
| meta1031 |  | 400.994 | 85.976 | 0.028 | 0.025 | 0.030 | 0.041 | 0.041 | 0.031 | 0.041 | 0.023 | 0.023 | 0.026 | 0.030 | 0.014 | 0.028 | 0.021 | 0.031 |
| meta1032 |  | 401.002 | 429.463 | 0.014 | 0.011 | 0.011 | 0.015 | 0.012 | 0.008 | 0.013 | 0.016 | 0.012 | 0.011 | 0.012 | 0.013 | 0.012 | 0.010 | 0.014 |
| meta1033 |  | 401.048 | 150.812 | 0.006 | 0.006 | 0.005 | 0.006 | 0.007 | 0.005 | 0.007 | 0.006 | 0.004 | 0.003 | 0.004 | 0.005 | 0.005 | 0.004 | 0.005 |
| meta1034 |  | 401.079 | 154.012 | 0.004 | 0.004 | 0.004 | 0.005 | 0.006 | 0.005 | 0.007 | 0.004 | 0.002 | 0.002 | 0.003 | 0.005 | 0.004 | 0.004 | 0.003 |
| meta1035 | Cellobiose | 401.126 | 344.203 | 0.002 | 0.002 | 0.001 | 0.003 | 0.001 | 0.002 | 0.001 | 0.002 | 0.001 | 0.002 | 0.001 | 0.002 | 0.001 | 0.002 | 0.001 |
| meta1036 |  | 401.215 | 187.639 | 0.001 | 0.000 | 0.001 | 0.001 | 0.001 | 0.001 | 0.001 | 0.001 | 0.001 | 0.000 | 0.000 | 0.001 | 0.000 | 0.001 | 0.000 |
| meta1037 |  | 402.025 | 417.472 | 0.004 | 0.003 | 0.003 | 0.004 | 0.003 | 0.002 | 0.003 | 0.003 | 0.003 | 0.003 | 0.003 | 0.003 | 0.002 | 0.002 | 0.003 |
| meta1038 |  | 402.086 | 263.387 | 0.063 | 0.056 | 0.055 | 0.058 | 0.075 | 0.075 | 0.064 | 0.059 | 0.037 | 0.053 | 0.072 | 0.075 | 0.042 | 0.039 | 0.023 |
| meta1039 |  | 402.161 | 360.858 | 0.005 | 0.004 | 0.005 | 0.005 | 0.005 | 0.003 | 0.006 | 0.005 | 0.003 | 0.005 | 0.006 | 0.004 | 0.004 | 0.003 | 0.007 |
| meta1040 |  | 402.983 | 420.822 | 0.014 | 0.010 | 0.010 | 0.014 | 0.014 | 0.008 | 0.012 | 0.016 | 0.014 | 0.015 | 0.015 | 0.016 | 0.027 | 0.011 | 0.010 |
| meta1041 |  | 403.030 | 25.052 | 0.000 | 0.000 | 0.000 | 0.000 | 0.000 | 0.000 | 0.001 | 0.001 | 0.001 | 0.001 | 0.000 | 0.000 | 0.000 | 0.000 | 0.000 |
| meta1042 | Farnesyl pyrophosphate | 403.096 | 469.639 | 0.000 | 0.000 | 0.000 | 0.000 | 0.000 | 0.000 | 0.000 | 0.001 | 0.000 | 0.000 | 0.000 | 0.001 | 0.000 | 0.000 | 0.000 |
| meta1043 |  | 403.118 | 398.681 | 0.045 | 0.041 | 0.033 | 0.055 | 0.050 | 0.037 | 0.036 | 0.041 | 0.020 | 0.032 | 0.034 | 0.040 | 0.032 | 0.029 | 0.030 |
| meta1044 |  | 403.267 | 78.089 | 0.019 | 0.017 | 0.026 | 0.038 | 0.020 | 0.028 | 0.021 | 0.021 | 0.016 | 0.022 | 0.013 | 0.023 | 0.012 | 0.017 | 0.018 |
| meta1045 |  | 403.267 | 100.396 | 0.007 | 0.005 | 0.005 | 0.008 | 0.008 | 0.006 | 0.004 | 0.006 | 0.004 | 0.007 | 0.008 | 0.005 | 0.005 | 0.007 | 0.006 |
| meta1046 |  | 404.102 | 368.816 | 0.320 | 0.256 | 0.239 | 0.280 | 0.294 | 0.278 | 0.208 | 0.298 | 0.180 | 0.269 | 0.295 | 0.369 | 0.219 | 0.225 | 0.148 |
| meta1047 |  | 404.128 | 475.293 | 0.001 | 0.001 | 0.001 | 0.001 | 0.001 | 0.001 | 0.001 | 0.002 | 0.001 | 0.001 | 0.001 | 0.002 | 0.001 | 0.002 | 0.001 |
| meta1048 |  | 404.190 | 278.034 | 0.012 | 0.010 | 0.011 | 0.009 | 0.013 | 0.009 | 0.023 | 0.011 | 0.008 | 0.010 | 0.007 | 0.010 | 0.008 | 0.009 | 0.005 |
| meta1049 |  | 404.998 | 267.880 | 0.006 | 0.006 | 0.007 | 0.008 | 0.007 | 0.005 | 0.006 | 0.005 | 0.006 | 0.005 | 0.006 | 0.005 | 0.008 | 0.006 | 0.008 |
| meta1050 |  | 405.262 | 126.363 | 0.007 | 0.006 | 0.007 | 0.002 | 0.002 | 0.004 | 0.021 | 0.003 | 0.002 | 0.018 | 0.004 | 0.022 | 0.005 | 0.009 | 0.005 |
| meta1051 |  | 405.261 | 178.010 | 0.004 | 0.004 | 0.004 | 0.001 | 0.001 | 0.001 | 0.021 | 0.001 | 0.002 | 0.003 | 0.002 | 0.006 | 0.003 | 0.006 | 0.001 |
| meta1052 |  | 405.297 | 46.551 | 0.001 | 0.001 | 0.002 | 0.002 | 0.002 | 0.002 | 0.002 | 0.002 | 0.002 | 0.001 | 0.001 | 0.002 | 0.001 | 0.001 | 0.001 |
| meta1053 |  | 406.003 | 25.220 | 0.008 | 0.009 | 0.009 | 0.007 | 0.006 | 0.007 | 0.009 | 0.014 | 0.015 | 0.013 | 0.010 | 0.005 | 0.006 | 0.006 | 0.004 |
| meta1054 |  | 406.019 | 416.519 | 0.007 | 0.005 | 0.005 | 0.006 | 0.005 | 0.004 | 0.005 | 0.007 | 0.006 | 0.005 | 0.005 | 0.005 | 0.006 | 0.005 | 0.007 |
| meta1055 |  | 406.036 | 158.644 | 0.003 | 0.003 | 0.002 | 0.003 | 0.004 | 0.001 | 0.002 | 0.002 | 0.004 | 0.002 | 0.003 | 0.003 | 0.003 | 0.002 | 0.003 |
| meta1056 |  | 407.062 | 25.028 | 0.005 | 0.005 | 0.005 | 0.007 | 0.003 | 0.005 | 0.010 | 0.005 | 0.008 | 0.008 | 0.007 | 0.003 | 0.005 | 0.007 | 0.007 |
| meta1057 |  | 407.073 | 469.373 | 0.002 | 0.002 | 0.002 | 0.003 | 0.004 | 0.002 | 0.001 | 0.002 | 0.001 | 0.003 | 0.001 | 0.001 | 0.002 | 0.002 | 0.001 |
| meta1058 |  | 407.099 | 31.578 | 0.001 | 0.001 | 0.001 | 0.001 | 0.001 | 0.001 | 0.002 | 0.003 | 0.003 | 0.002 | 0.001 | 0.002 | 0.001 | 0.001 | 0.001 |
| meta1059 |  | 407.169 | 27.520 | 0.004 | 0.004 | 0.003 | 0.007 | 0.006 | 0.004 | 0.003 | 0.003 | 0.002 | 0.002 | 0.002 | 0.003 | 0.003 | 0.003 | 0.002 |
| meta1060 | Cholic acid | 407.277 | 203.891 | 0.019 | 0.017 | 0.018 | 0.003 | 0.005 | 0.007 | 0.082 | 0.004 | 0.009 | 0.023 | 0.016 | 0.018 | 0.020 | 0.036 | 0.009 |
| meta1061 |  | 407.986 | 161.841 | 0.002 | 0.002 | 0.003 | 0.003 | 0.002 | 0.002 | 0.002 | 0.000 | 0.004 | 0.001 | 0.002 | 0.002 | 0.003 | 0.002 | 0.002 |
| meta1062 |  | 408.041 | 274.249 | 0.002 | 0.003 | 0.003 | 0.003 | 0.003 | 0.003 | 0.002 | 0.003 | 0.003 | 0.003 | 0.003 | 0.002 | 0.003 | 0.003 | 0.003 |
| meta1063 |  | 408.087 | 326.358 | 0.007 | 0.005 | 0.006 | 0.004 | 0.005 | 0.004 | 0.010 | 0.003 | 0.014 | 0.008 | 0.006 | 0.006 | 0.004 | 0.004 | 0.002 |
| meta1064 |  | 408.876 | 485.122 | 0.001 | 0.002 | 0.002 | 0.003 | 0.002 | 0.003 | 0.002 | 0.002 | 0.002 | 0.001 | 0.001 | 0.002 | 0.003 | 0.002 | 0.002 |
| meta1065 |  | 408.898 | 576.687 | 0.001 | 0.001 | 0.001 | 0.001 | 0.001 | 0.001 | 0.001 | 0.001 | 0.001 | 0.001 | 0.001 | 0.001 | 0.001 | 0.001 | 0.001 |
| meta1066 |  | 408.908 | 287.061 | 0.014 | 0.009 | 0.010 | 0.018 | 0.016 | 0.014 | 0.016 | 0.017 | 0.017 | 0.014 | 0.014 | 0.015 | 0.021 | 0.014 | 0.013 |
| meta1067 | 1-Palmitoyl Lysophosphatidic Acid | 409.233 | 174.478 | 0.021 | 0.022 | 0.023 | 0.032 | 0.025 | 0.022 | 0.036 | 0.026 | 0.019 | 0.020 | 0.023 | 0.022 | 0.022 | 0.024 | 0.024 |
| meta1068 |  | 409.257 | 46.560 | 0.003 | 0.003 | 0.003 | 0.004 | 0.006 | 0.002 | 0.003 | 0.005 | 0.001 | 0.003 | 0.003 | 0.003 | 0.002 | 0.002 | 0.002 |
| meta1069 |  | 410.144 | 27.455 | 0.012 | 0.011 | 0.010 | 0.011 | 0.013 | 0.010 | 0.010 | 0.011 | 0.009 | 0.009 | 0.007 | 0.009 | 0.011 | 0.009 | 0.011 |
| meta1070 |  | 410.234 | 38.799 | 0.029 | 0.022 | 0.026 | 0.035 | 0.069 | 0.024 | 0.036 | 0.060 | 0.026 | 0.048 | 0.056 | 0.026 | 0.028 | 0.060 | 0.035 |
| meta1071 |  | 410.984 | 318.143 | 0.003 | 0.002 | 0.003 | 0.003 | 0.003 | 0.003 | 0.002 | 0.002 | 0.003 | 0.003 | 0.002 | 0.002 | 0.003 | 0.003 | 0.003 |
| meta1072 |  | 411.011 | 64.705 | 0.006 | 0.006 | 0.006 | 0.003 | 0.003 | 0.002 | 0.002 | 0.014 | 0.005 | 0.011 | 0.008 | 0.003 | 0.006 | 0.003 | 0.007 |
| meta1073 |  | 411.072 | 46.589 | 0.001 | 0.001 | 0.001 | 0.001 | 0.001 | 0.001 | 0.003 | 0.001 | 0.001 | 0.001 | 0.001 | 0.001 | 0.001 | 0.001 | 0.001 |
| meta1074 |  | 411.106 | 299.567 | 0.003 | 0.003 | 0.003 | 0.003 | 0.004 | 0.002 | 0.004 | 0.005 | 0.002 | 0.003 | 0.003 | 0.003 | 0.001 | 0.001 | 0.004 |
| meta1075 |  | 411.272 | 33.764 | 0.001 | 0.001 | 0.001 | 0.002 | 0.002 | 0.001 | 0.002 | 0.002 | 0.001 | 0.002 | 0.002 | 0.002 | 0.002 | 0.003 | 0.001 |
| meta1076 |  | 411.980 | 274.069 | 0.004 | 0.004 | 0.004 | 0.004 | 0.004 | 0.004 | 0.004 | 0.005 | 0.003 | 0.004 | 0.004 | 0.003 | 0.004 | 0.003 | 0.004 |
| meta1077 |  | 413.178 | 157.884 | 0.002 | 0.001 | 0.001 | 0.002 | 0.002 | 0.002 | 0.005 | 0.001 | 0.001 | 0.001 | 0.001 | 0.001 | 0.001 | 0.001 | 0.001 |
| meta1078 |  | 413.215 | 208.367 | 0.001 | 0.001 | 0.000 | 0.001 | 0.000 | 0.001 | 0.001 | 0.001 | 0.001 | 0.001 | 0.000 | 0.000 | 0.000 | 0.000 | 0.000 |
| meta1079 |  | 413.288 | 34.071 | 0.024 | 0.026 | 0.028 | 0.050 | 0.039 | 0.036 | 0.048 | 0.048 | 0.040 | 0.039 | 0.048 | 0.056 | 0.075 | 0.067 | 0.042 |
| meta1080 |  | 413.987 | 85.988 | 0.002 | 0.002 | 0.002 | 0.003 | 0.003 | 0.002 | 0.002 | 0.002 | 0.002 | 0.001 | 0.002 | 0.001 | 0.002 | 0.002 | 0.003 |
| meta1081 |  | 414.265 | 39.342 | 0.003 | 0.004 | 0.004 | 0.003 | 0.006 | 0.003 | 0.007 | 0.015 | 0.005 | 0.007 | 0.006 | 0.003 | 0.004 | 0.004 | 0.010 |
| meta1082 |  | 414.872 | 578.249 | 0.029 | 0.023 | 0.021 | 0.029 | 0.025 | 0.019 | 0.026 | 0.026 | 0.023 | 0.025 | 0.025 | 0.024 | 0.024 | 0.024 | 0.024 |
| meta1083 |  | 414.974 | 463.590 | 0.001 | 0.001 | 0.001 | 0.001 | 0.001 | 0.001 | 0.001 | 0.001 | 0.001 | 0.002 | 0.001 | 0.001 | 0.001 | 0.001 | 0.001 |
| meta1084 |  | 415.106 | 365.954 | 0.004 | 0.003 | 0.003 | 0.004 | 0.004 | 0.004 | 0.003 | 0.004 | 0.003 | 0.003 | 0.004 | 0.005 | 0.003 | 0.003 | 0.002 |
| meta1085 |  | 415.157 | 178.659 | 0.000 | 0.000 | 0.000 | 0.001 | 0.000 | 0.001 | 0.000 | 0.001 | 0.000 | 0.000 | 0.001 | 0.000 | 0.000 | 0.001 | 0.000 |
| meta1086 |  | 415.194 | 310.514 | 0.000 | 0.000 | 0.001 | 0.000 | 0.000 | 0.001 | 0.001 | 0.001 | 0.000 | 0.000 | 0.000 | 0.000 | 0.000 | 0.000 | 0.000 |
| meta1087 | Ramipril | 415.230 | 186.970 | 0.001 | 0.001 | 0.001 | 0.001 | 0.002 | 0.001 | 0.002 | 0.001 | 0.001 | 0.001 | 0.000 | 0.000 | 0.000 | 0.001 | 0.000 |
| meta1088 |  | 416.208 | 45.366 | 0.017 | 0.016 | 0.016 | 0.031 | 0.014 | 0.031 | 0.024 | 0.026 | 0.013 | 0.013 | 0.013 | 0.011 | 0.013 | 0.015 | 0.007 |
| meta1089 |  | 416.895 | 318.133 | 0.004 | 0.003 | 0.004 | 0.004 | 0.003 | 0.004 | 0.003 | 0.003 | 0.004 | 0.003 | 0.003 | 0.003 | 0.004 | 0.004 | 0.003 |
| meta1090 |  | 417.046 | 25.070 | 0.002 | 0.002 | 0.001 | 0.004 | 0.002 | 0.003 | 0.002 | 0.003 | 0.003 | 0.002 | 0.002 | 0.003 | 0.002 | 0.001 | 0.002 |
| meta1091 |  | 417.116 | 196.826 | 0.003 | 0.002 | 0.002 | 0.003 | 0.001 | 0.003 | 0.003 | 0.007 | 0.003 | 0.005 | 0.002 | 0.001 | 0.001 | 0.001 | 0.001 |
| meta1092 |  | 417.192 | 27.321 | 0.009 | 0.009 | 0.010 | 0.021 | 0.008 | 0.005 | 0.009 | 0.018 | 0.017 | 0.018 | 0.010 | 0.010 | 0.014 | 0.012 | 0.010 |
| meta1093 |  | 417.999 | 82.252 | 0.016 | 0.015 | 0.016 | 0.025 | 0.014 | 0.017 | 0.013 | 0.023 | 0.013 | 0.016 | 0.015 | 0.012 | 0.016 | 0.014 | 0.019 |
| meta1094 |  | 418.019 | 417.004 | 0.001 | 0.001 | 0.001 | 0.001 | 0.001 | 0.001 | 0.001 | 0.001 | 0.001 | 0.001 | 0.001 | 0.001 | 0.001 | 0.001 | 0.001 |
| meta1095 |  | 418.205 | 269.094 | 0.000 | 0.000 | 0.000 | 0.000 | 0.000 | 0.000 | 0.001 | 0.000 | 0.000 | 0.000 | 0.000 | 0.000 | 0.000 | 0.000 | 0.000 |
| meta1096 |  | 418.898 | 578.434 | 0.006 | 0.005 | 0.005 | 0.007 | 0.006 | 0.003 | 0.003 | 0.006 | 0.005 | 0.005 | 0.005 | 0.005 | 0.006 | 0.005 | 0.004 |
| meta1097 |  | 419.104 | 198.851 | 0.002 | 0.001 | 0.001 | 0.002 | 0.003 | 0.002 | 0.002 | 0.001 | 0.001 | 0.001 | 0.001 | 0.002 | 0.002 | 0.002 | 0.001 |
| meta1098 |  | 419.119 | 242.897 | 0.001 | 0.001 | 0.000 | 0.001 | 0.001 | 0.000 | 0.001 | 0.000 | 0.000 | 0.000 | 0.000 | 0.001 | 0.000 | 0.001 | 0.000 |
| meta1099 |  | 419.349 | 33.880 | 0.001 | 0.001 | 0.001 | 0.003 | 0.002 | 0.002 | 0.004 | 0.004 | 0.002 | 0.002 | 0.002 | 0.002 | 0.001 | 0.001 | 0.001 |
| meta1100 |  | 421.062 | 341.995 | 0.001 | 0.001 | 0.001 | 0.001 | 0.001 | 0.001 | 0.001 | 0.000 | 0.001 | 0.001 | 0.000 | 0.000 | 0.001 | 0.001 | 0.000 |
| meta1101 |  | 421.147 | 101.220 | 0.005 | 0.005 | 0.005 | 0.008 | 0.018 | 0.006 | 0.016 | 0.005 | 0.003 | 0.003 | 0.006 | 0.004 | 0.002 | 0.007 | 0.011 |
| meta1102 |  | 421.208 | 24.388 | 0.004 | 0.005 | 0.005 | 0.006 | 0.008 | 0.005 | 0.006 | 0.001 | 0.005 | 0.011 | 0.004 | 0.017 | 0.004 | 0.005 | 0.004 |
| meta1103 |  | 422.022 | 417.472 | 0.010 | 0.009 | 0.008 | 0.010 | 0.009 | 0.007 | 0.009 | 0.008 | 0.008 | 0.008 | 0.008 | 0.009 | 0.008 | 0.007 | 0.010 |
| meta1104 |  | 422.085 | 473.923 | 0.051 | 0.042 | 0.046 | 0.047 | 0.048 | 0.039 | 0.031 | 0.085 | 0.056 | 0.119 | 0.058 | 0.074 | 0.066 | 0.037 | 0.043 |
| meta1105 |  | 422.125 | 398.727 | 0.002 | 0.003 | 0.002 | 0.003 | 0.003 | 0.002 | 0.002 | 0.002 | 0.001 | 0.002 | 0.002 | 0.002 | 0.002 | 0.002 | 0.002 |
| meta1106 |  | 422.139 | 436.758 | 0.001 | 0.001 | 0.001 | 0.001 | 0.001 | 0.001 | 0.000 | 0.001 | 0.000 | 0.001 | 0.001 | 0.001 | 0.001 | 0.001 | 0.001 |
| meta1107 |  | 422.197 | 42.006 | 0.012 | 0.012 | 0.012 | 0.026 | 0.017 | 0.022 | 0.023 | 0.015 | 0.013 | 0.014 | 0.009 | 0.008 | 0.010 | 0.014 | 0.005 |
| meta1108 |  | 422.945 | 422.923 | 0.002 | 0.002 | 0.002 | 0.003 | 0.003 | 0.001 | 0.002 | 0.002 | 0.002 | 0.002 | 0.002 | 0.002 | 0.002 | 0.002 | 0.002 |
| meta1109 |  | 422.976 | 85.566 | 0.016 | 0.015 | 0.017 | 0.024 | 0.027 | 0.018 | 0.025 | 0.013 | 0.013 | 0.014 | 0.018 | 0.006 | 0.016 | 0.013 | 0.021 |
| meta1110 |  | 423.109 | 368.197 | 0.007 | 0.005 | 0.005 | 0.006 | 0.006 | 0.006 | 0.005 | 0.005 | 0.004 | 0.006 | 0.005 | 0.006 | 0.005 | 0.005 | 0.003 |
| meta1111 |  | 423.122 | 244.590 | 0.003 | 0.004 | 0.003 | 0.004 | 0.004 | 0.003 | 0.005 | 0.004 | 0.004 | 0.003 | 0.003 | 0.003 | 0.005 | 0.003 | 0.004 |
| meta1112 | Pravastatin | 423.242 | 47.247 | 0.002 | 0.002 | 0.003 | 0.002 | 0.003 | 0.001 | 0.003 | 0.003 | 0.002 | 0.004 | 0.004 | 0.003 | 0.000 | 0.003 | 0.001 |
| meta1113 |  | 423.272 | 212.603 | 0.001 | 0.001 | 0.001 | 0.001 | 0.001 | 0.000 | 0.002 | 0.001 | 0.000 | 0.001 | 0.002 | 0.004 | 0.002 | 0.002 | 0.001 |
| meta1114 |  | 424.152 | 189.562 | 0.000 | 0.000 | 0.000 | 0.000 | 0.000 | 0.000 | 0.000 | 0.000 | 0.000 | 0.001 | 0.001 | 0.000 | 0.000 | 0.001 | 0.000 |
| meta1115 |  | 424.159 | 26.815 | 0.009 | 0.008 | 0.008 | 0.008 | 0.009 | 0.007 | 0.009 | 0.008 | 0.006 | 0.010 | 0.009 | 0.009 | 0.011 | 0.010 | 0.010 |
| meta1116 |  | 424.194 | 154.674 | 0.003 | 0.003 | 0.002 | 0.002 | 0.007 | 0.001 | 0.003 | 0.003 | 0.000 | 0.002 | 0.003 | 0.001 | 0.002 | 0.001 | 0.004 |
| meta1117 |  | 425.059 | 200.306 | 0.003 | 0.002 | 0.003 | 0.003 | 0.003 | 0.002 | 0.003 | 0.002 | 0.002 | 0.002 | 0.002 | 0.003 | 0.003 | 0.002 | 0.002 |
| meta1118 |  | 425.122 | 298.713 | 0.008 | 0.007 | 0.007 | 0.008 | 0.010 | 0.006 | 0.009 | 0.008 | 0.004 | 0.006 | 0.008 | 0.007 | 0.005 | 0.004 | 0.005 |
| meta1119 |  | 425.126 | 427.920 | 0.005 | 0.004 | 0.005 | 0.005 | 0.004 | 0.006 | 0.004 | 0.007 | 0.002 | 0.006 | 0.006 | 0.007 | 0.004 | 0.003 | 0.003 |
| meta1120 | Linustatin | 425.178 | 143.574 | 0.004 | 0.004 | 0.003 | 0.008 | 0.003 | 0.006 | 0.004 | 0.006 | 0.004 | 0.004 | 0.003 | 0.003 | 0.002 | 0.002 | 0.001 |
| meta1121 |  | 425.239 | 25.820 | 0.007 | 0.006 | 0.006 | 0.010 | 0.008 | 0.007 | 0.009 | 0.008 | 0.008 | 0.017 | 0.006 | 0.006 | 0.004 | 0.005 | 0.012 |
| meta1122 |  | 425.302 | 32.479 | 0.001 | 0.001 | 0.001 | 0.000 | 0.001 | 0.000 | 0.001 | 0.001 | 0.001 | 0.000 | 0.001 | 0.001 | 0.001 | 0.000 | 0.001 |
| meta1123 | Adenosine 5'-diphosphate (ADP) | 426.019 | 483.270 | 0.022 | 0.021 | 0.021 | 0.027 | 0.024 | 0.023 | 0.023 | 0.029 | 0.027 | 0.026 | 0.017 | 0.025 | 0.026 | 0.025 | 0.026 |
| meta1124 |  | 426.123 | 94.591 | 0.006 | 0.005 | 0.006 | 0.008 | 0.009 | 0.006 | 0.011 | 0.004 | 0.002 | 0.003 | 0.005 | 0.004 | 0.004 | 0.006 | 0.005 |
| meta1125 |  | 426.193 | 45.233 | 0.007 | 0.007 | 0.006 | 0.012 | 0.006 | 0.010 | 0.008 | 0.007 | 0.009 | 0.008 | 0.008 | 0.007 | 0.008 | 0.006 | 0.007 |
| meta1126 |  | 427.132 | 438.129 | 0.001 | 0.001 | 0.001 | 0.001 | 0.001 | 0.001 | 0.001 | 0.001 | 0.000 | 0.001 | 0.001 | 0.001 | 0.000 | 0.001 | 0.001 |
| meta1127 |  | 427.148 | 174.202 | 0.001 | 0.001 | 0.001 | 0.002 | 0.003 | 0.001 | 0.004 | 0.002 | 0.001 | 0.001 | 0.001 | 0.001 | 0.001 | 0.002 | 0.001 |
| meta1128 |  | 427.319 | 46.577 | 0.002 | 0.002 | 0.002 | 0.004 | 0.004 | 0.003 | 0.004 | 0.002 | 0.002 | 0.002 | 0.003 | 0.003 | 0.002 | 0.002 | 0.002 |
| meta1129 |  | 427.932 | 25.860 | 0.009 | 0.014 | 0.014 | 0.012 | 0.009 | 0.009 | 0.010 | 0.014 | 0.013 | 0.017 | 0.009 | 0.008 | 0.014 | 0.012 | 0.016 |
| meta1130 |  | 428.023 | 426.779 | 0.003 | 0.003 | 0.004 | 0.005 | 0.004 | 0.003 | 0.004 | 0.004 | 0.003 | 0.003 | 0.003 | 0.003 | 0.004 | 0.003 | 0.003 |
| meta1131 |  | 428.106 | 361.373 | 0.006 | 0.005 | 0.004 | 0.005 | 0.007 | 0.003 | 0.008 | 0.005 | 0.003 | 0.006 | 0.008 | 0.006 | 0.004 | 0.002 | 0.008 |
| meta1132 |  | 429.123 | 323.178 | 0.010 | 0.009 | 0.008 | 0.009 | 0.016 | 0.010 | 0.012 | 0.008 | 0.005 | 0.007 | 0.009 | 0.015 | 0.007 | 0.011 | 0.005 |
| meta1133 |  | 429.246 | 138.186 | 0.006 | 0.006 | 0.005 | 0.005 | 0.004 | 0.005 | 0.004 | 0.013 | 0.005 | 0.011 | 0.013 | 0.013 | 0.005 | 0.006 | 0.010 |
| meta1134 |  | 429.247 | 42.263 | 0.053 | 0.048 | 0.044 | 0.105 | 0.077 | 0.087 | 0.084 | 0.038 | 0.033 | 0.050 | 0.038 | 0.050 | 0.048 | 0.049 | 0.034 |
| meta1135 | alpha-Tocopherol (Vitamin E) | 429.370 | 31.540 | 0.001 | 0.001 | 0.002 | 0.000 | 0.000 | 0.000 | 0.000 | 0.001 | 0.000 | 0.000 | 0.001 | 0.002 | 0.000 | 0.000 | 0.000 |
| meta1136 |  | 431.225 | 185.768 | 0.005 | 0.004 | 0.004 | 0.005 | 0.007 | 0.006 | 0.009 | 0.003 | 0.001 | 0.004 | 0.001 | 0.002 | 0.001 | 0.004 | 0.001 |
| meta1137 |  | 431.261 | 137.645 | 0.007 | 0.006 | 0.006 | 0.004 | 0.006 | 0.006 | 0.005 | 0.012 | 0.005 | 0.005 | 0.022 | 0.021 | 0.005 | 0.009 | 0.004 |
| meta1138 |  | 431.298 | 78.187 | 0.025 | 0.022 | 0.020 | 0.026 | 0.022 | 0.030 | 0.020 | 0.023 | 0.029 | 0.024 | 0.022 | 0.016 | 0.019 | 0.021 | 0.017 |
| meta1139 |  | 431.298 | 100.460 | 0.009 | 0.007 | 0.006 | 0.011 | 0.009 | 0.007 | 0.005 | 0.008 | 0.005 | 0.008 | 0.009 | 0.006 | 0.006 | 0.009 | 0.013 |
| meta1140 |  | 432.218 | 39.102 | 0.089 | 0.077 | 0.081 | 0.126 | 0.253 | 0.087 | 0.208 | 0.139 | 0.108 | 0.170 | 0.083 | 0.107 | 0.055 | 0.089 | 0.291 |
| meta1141 |  | 432.883 | 577.649 | 0.004 | 0.004 | 0.004 | 0.005 | 0.005 | 0.003 | 0.004 | 0.005 | 0.004 | 0.004 | 0.004 | 0.004 | 0.005 | 0.004 | 0.005 |
| meta1142 |  | 433.045 | 24.517 | 0.001 | 0.001 | 0.001 | 0.003 | 0.002 | 0.002 | 0.001 | 0.002 | 0.002 | 0.002 | 0.002 | 0.001 | 0.001 | 0.001 | 0.001 |
| meta1143 |  | 433.067 | 273.439 | 0.001 | 0.001 | 0.001 | 0.001 | 0.001 | 0.001 | 0.001 | 0.002 | 0.001 | 0.002 | 0.001 | 0.001 | 0.001 | 0.001 | 0.001 |
| meta1144 |  | 433.233 | 166.983 | 0.003 | 0.003 | 0.005 | 0.006 | 0.004 | 0.003 | 0.008 | 0.005 | 0.003 | 0.004 | 0.003 | 0.004 | 0.004 | 0.003 | 0.003 |
| meta1145 |  | 434.109 | 94.155 | 0.002 | 0.002 | 0.002 | 0.004 | 0.004 | 0.002 | 0.004 | 0.002 | 0.001 | 0.001 | 0.002 | 0.002 | 0.001 | 0.002 | 0.002 |
| meta1146 |  | 434.120 | 336.773 | 0.001 | 0.001 | 0.001 | 0.000 | 0.002 | 0.000 | 0.003 | 0.000 | 0.002 | 0.001 | 0.000 | 0.000 | 0.002 | 0.000 | 0.000 |
| meta1147 |  | 434.263 | 32.798 | 0.002 | 0.002 | 0.003 | 0.004 | 0.002 | 0.002 | 0.002 | 0.002 | 0.002 | 0.002 | 0.002 | 0.002 | 0.002 | 0.002 | 0.002 |
| meta1148 |  | 434.945 | 318.308 | 0.005 | 0.004 | 0.005 | 0.006 | 0.005 | 0.005 | 0.005 | 0.005 | 0.005 | 0.005 | 0.006 | 0.005 | 0.005 | 0.006 | 0.004 |
| meta1149 |  | 435.028 | 284.693 | 0.016 | 0.011 | 0.009 | 0.017 | 0.015 | 0.010 | 0.016 | 0.015 | 0.007 | 0.010 | 0.013 | 0.012 | 0.012 | 0.008 | 0.010 |
| meta1150 |  | 435.185 | 25.800 | 0.003 | 0.002 | 0.002 | 0.003 | 0.002 | 0.002 | 0.005 | 0.003 | 0.005 | 0.003 | 0.002 | 0.004 | 0.002 | 0.002 | 0.001 |
| meta1151 | 1-Oleoyl-L-.alpha.-lysophosphatidic acid | 435.248 | 173.194 | 0.004 | 0.005 | 0.005 | 0.007 | 0.006 | 0.005 | 0.008 | 0.006 | 0.004 | 0.005 | 0.006 | 0.006 | 0.005 | 0.006 | 0.007 |
| meta1152 |  | 436.048 | 417.837 | 0.002 | 0.002 | 0.002 | 0.003 | 0.002 | 0.002 | 0.002 | 0.002 | 0.002 | 0.002 | 0.002 | 0.002 | 0.002 | 0.002 | 0.002 |
| meta1153 |  | 436.099 | 471.546 | 0.003 | 0.002 | 0.002 | 0.004 | 0.005 | 0.002 | 0.002 | 0.003 | 0.001 | 0.004 | 0.001 | 0.002 | 0.002 | 0.003 | 0.002 |
| meta1154 |  | 436.176 | 45.924 | 0.002 | 0.001 | 0.001 | 0.002 | 0.001 | 0.002 | 0.001 | 0.002 | 0.003 | 0.002 | 0.001 | 0.001 | 0.001 | 0.001 | 0.001 |
| meta1155 |  | 436.770 | 289.065 | 0.005 | 0.005 | 0.006 | 0.007 | 0.005 | 0.005 | 0.006 | 0.006 | 0.007 | 0.005 | 0.005 | 0.005 | 0.006 | 0.006 | 0.006 |
| meta1156 |  | 436.924 | 318.138 | 0.001 | 0.001 | 0.001 | 0.001 | 0.001 | 0.001 | 0.001 | 0.001 | 0.001 | 0.001 | 0.001 | 0.001 | 0.001 | 0.001 | 0.001 |
| meta1157 | Vindoline | 437.210 | 253.645 | 0.000 | 0.000 | 0.000 | 0.000 | 0.000 | 0.000 | 0.000 | 0.000 | 0.000 | 0.000 | 0.000 | 0.000 | 0.000 | 0.000 | 0.000 |
| meta1158 |  | 437.264 | 170.993 | 0.009 | 0.010 | 0.010 | 0.012 | 0.011 | 0.007 | 0.014 | 0.011 | 0.009 | 0.009 | 0.010 | 0.010 | 0.009 | 0.008 | 0.014 |
| meta1159 |  | 437.288 | 33.568 | 0.004 | 0.005 | 0.006 | 0.011 | 0.009 | 0.006 | 0.011 | 0.008 | 0.007 | 0.007 | 0.010 | 0.009 | 0.012 | 0.015 | 0.008 |
| meta1160 |  | 438.079 | 478.536 | 0.001 | 0.001 | 0.001 | 0.001 | 0.001 | 0.001 | 0.001 | 0.001 | 0.001 | 0.002 | 0.001 | 0.001 | 0.001 | 0.001 | 0.001 |
| meta1161 |  | 438.909 | 579.499 | 0.005 | 0.004 | 0.003 | 0.005 | 0.005 | 0.003 | 0.004 | 0.004 | 0.004 | 0.004 | 0.004 | 0.004 | 0.004 | 0.004 | 0.004 |
| meta1162 |  | 438.950 | 85.461 | 0.004 | 0.003 | 0.004 | 0.006 | 0.006 | 0.004 | 0.006 | 0.003 | 0.003 | 0.003 | 0.004 | 0.002 | 0.004 | 0.003 | 0.005 |
| meta1163 |  | 438.973 | 36.840 | 0.001 | 0.001 | 0.001 | 0.000 | 0.000 | 0.000 | 0.001 | 0.001 | 0.003 | 0.004 | 0.001 | 0.003 | 0.000 | 0.001 | 0.000 |
| meta1164 |  | 440.033 | 417.472 | 0.004 | 0.003 | 0.003 | 0.004 | 0.004 | 0.003 | 0.004 | 0.003 | 0.003 | 0.002 | 0.003 | 0.003 | 0.004 | 0.003 | 0.004 |
| meta1165 |  | 440.857 | 318.126 | 0.002 | 0.002 | 0.002 | 0.002 | 0.002 | 0.002 | 0.002 | 0.002 | 0.002 | 0.002 | 0.002 | 0.002 | 0.002 | 0.003 | 0.002 |
| meta1166 |  | 440.869 | 589.298 | 0.026 | 0.027 | 0.022 | 0.031 | 0.053 | 0.044 | 0.055 | 0.056 | 0.021 | 0.054 | 0.050 | 0.021 | 0.054 | 0.025 | 0.026 |
| meta1167 |  | 441.021 | 165.717 | 0.001 | 0.001 | 0.001 | 0.001 | 0.001 | 0.000 | 0.001 | 0.002 | 0.001 | 0.001 | 0.001 | 0.000 | 0.001 | 0.000 | 0.001 |
| meta1168 |  | 441.029 | 484.890 | 0.004 | 0.003 | 0.003 | 0.004 | 0.004 | 0.004 | 0.004 | 0.004 | 0.004 | 0.004 | 0.003 | 0.004 | 0.005 | 0.004 | 0.004 |
| meta1169 | hydrocortisone 21-acetate | 441.173 | 159.261 | 0.002 | 0.002 | 0.002 | 0.004 | 0.002 | 0.003 | 0.003 | 0.003 | 0.003 | 0.002 | 0.002 | 0.003 | 0.001 | 0.001 | 0.001 |
| meta1170 |  | 441.234 | 27.235 | 0.005 | 0.005 | 0.004 | 0.010 | 0.009 | 0.010 | 0.008 | 0.008 | 0.008 | 0.009 | 0.008 | 0.007 | 0.006 | 0.005 | 0.009 |
| meta1171 |  | 441.264 | 39.778 | 0.001 | 0.001 | 0.001 | 0.002 | 0.002 | 0.001 | 0.002 | 0.001 | 0.001 | 0.002 | 0.002 | 0.002 | 0.001 | 0.001 | 0.001 |
| meta1172 |  | 442.187 | 46.048 | 0.004 | 0.004 | 0.004 | 0.005 | 0.003 | 0.006 | 0.005 | 0.003 | 0.004 | 0.004 | 0.005 | 0.003 | 0.005 | 0.003 | 0.004 |
| meta1173 |  | 443.137 | 427.876 | 0.007 | 0.007 | 0.007 | 0.008 | 0.006 | 0.010 | 0.007 | 0.009 | 0.005 | 0.008 | 0.007 | 0.010 | 0.007 | 0.005 | 0.004 |
| meta1174 |  | 443.163 | 243.081 | 0.000 | 0.000 | 0.000 | 0.000 | 0.000 | 0.000 | 0.001 | 0.000 | 0.000 | 0.000 | 0.000 | 0.000 | 0.000 | 0.000 | 0.000 |
| meta1175 |  | 443.254 | 170.293 | 0.000 | 0.000 | 0.000 | 0.000 | 0.000 | 0.000 | 0.001 | 0.000 | 0.000 | 0.001 | 0.000 | 0.001 | 0.001 | 0.002 | 0.000 |
| meta1176 |  | 443.298 | 147.722 | 0.001 | 0.001 | 0.002 | 0.003 | 0.003 | 0.002 | 0.002 | 0.002 | 0.001 | 0.001 | 0.001 | 0.002 | 0.001 | 0.001 | 0.002 |
| meta1177 |  | 444.020 | 417.472 | 0.038 | 0.034 | 0.033 | 0.040 | 0.031 | 0.029 | 0.032 | 0.044 | 0.034 | 0.039 | 0.035 | 0.037 | 0.038 | 0.030 | 0.037 |
| meta1178 |  | 444.066 | 473.669 | 0.001 | 0.001 | 0.001 | 0.001 | 0.001 | 0.001 | 0.001 | 0.001 | 0.001 | 0.002 | 0.001 | 0.001 | 0.001 | 0.001 | 0.001 |
| meta1179 |  | 444.239 | 44.723 | 0.003 | 0.002 | 0.002 | 0.006 | 0.002 | 0.005 | 0.003 | 0.006 | 0.003 | 0.003 | 0.003 | 0.001 | 0.001 | 0.002 | 0.002 |
| meta1180 |  | 444.958 | 86.595 | 0.003 | 0.003 | 0.003 | 0.004 | 0.004 | 0.004 | 0.005 | 0.001 | 0.002 | 0.002 | 0.004 | 0.001 | 0.003 | 0.003 | 0.004 |
| meta1181 | CDP-ethanolamine | 445.050 | 431.509 | 0.009 | 0.009 | 0.009 | 0.010 | 0.009 | 0.008 | 0.011 | 0.011 | 0.010 | 0.010 | 0.008 | 0.009 | 0.013 | 0.008 | 0.009 |
| meta1182 |  | 446.065 | 428.763 | 0.003 | 0.002 | 0.002 | 0.002 | 0.002 | 0.002 | 0.002 | 0.003 | 0.001 | 0.003 | 0.002 | 0.003 | 0.003 | 0.002 | 0.002 |
| meta1183 |  | 446.148 | 364.440 | 0.010 | 0.009 | 0.008 | 0.011 | 0.013 | 0.010 | 0.009 | 0.011 | 0.007 | 0.010 | 0.010 | 0.015 | 0.009 | 0.008 | 0.005 |
| meta1184 |  | 446.371 | 31.612 | 0.001 | 0.001 | 0.002 | 0.002 | 0.003 | 0.001 | 0.003 | 0.005 | 0.003 | 0.001 | 0.003 | 0.004 | 0.001 | 0.001 | 0.003 |
| meta1185 |  | 447.090 | 196.177 | 0.001 | 0.000 | 0.001 | 0.000 | 0.000 | 0.000 | 0.000 | 0.002 | 0.003 | 0.001 | 0.001 | 0.000 | 0.000 | 0.000 | 0.000 |
| meta1186 | S-(p-Azidophenacyl)glutathione | 447.115 | 442.335 | 0.001 | 0.000 | 0.000 | 0.000 | 0.001 | 0.000 | 0.001 | 0.000 | 0.001 | 0.000 | 0.000 | 0.000 | 0.001 | 0.000 | 0.000 |
| meta1187 |  | 447.131 | 47.354 | 0.002 | 0.002 | 0.001 | 0.005 | 0.004 | 0.000 | 0.005 | 0.001 | 0.001 | 0.000 | 0.001 | 0.000 | 0.000 | 0.001 | 0.000 |
| meta1188 | Stavudine | 447.162 | 331.707 | 0.001 | 0.001 | 0.001 | 0.001 | 0.001 | 0.001 | 0.001 | 0.002 | 0.000 | 0.001 | 0.001 | 0.001 | 0.001 | 0.001 | 0.001 |
| meta1189 |  | 447.948 | 165.050 | 0.000 | 0.000 | 0.000 | 0.000 | 0.000 | 0.000 | 0.000 | 0.001 | 0.000 | 0.000 | 0.000 | 0.000 | 0.000 | 0.000 | 0.000 |
| meta1190 |  | 448.037 | 157.980 | 0.001 | 0.001 | 0.000 | 0.001 | 0.001 | 0.000 | 0.001 | 0.001 | 0.001 | 0.001 | 0.001 | 0.001 | 0.001 | 0.001 | 0.001 |
| meta1191 |  | 448.110 | 385.464 | 0.046 | 0.037 | 0.037 | 0.047 | 0.046 | 0.038 | 0.024 | 0.069 | 0.022 | 0.042 | 0.030 | 0.028 | 0.036 | 0.039 | 0.032 |
| meta1192 |  | 448.843 | 287.343 | 0.008 | 0.006 | 0.006 | 0.010 | 0.009 | 0.007 | 0.009 | 0.008 | 0.009 | 0.008 | 0.007 | 0.008 | 0.010 | 0.008 | 0.007 |
| meta1193 |  | 449.324 | 48.130 | 0.001 | 0.001 | 0.001 | 0.001 | 0.001 | 0.002 | 0.002 | 0.001 | 0.002 | 0.002 | 0.001 | 0.002 | 0.001 | 0.001 | 0.001 |
| meta1194 |  | 450.028 | 417.004 | 0.004 | 0.003 | 0.004 | 0.005 | 0.003 | 0.003 | 0.003 | 0.004 | 0.004 | 0.004 | 0.004 | 0.004 | 0.004 | 0.003 | 0.004 |
| meta1195 |  | 450.203 | 139.618 | 0.006 | 0.006 | 0.006 | 0.005 | 0.006 | 0.004 | 0.006 | 0.005 | 0.003 | 0.005 | 0.007 | 0.007 | 0.005 | 0.005 | 0.005 |
| meta1196 |  | 450.259 | 181.960 | 0.005 | 0.004 | 0.005 | 0.008 | 0.007 | 0.004 | 0.008 | 0.007 | 0.003 | 0.005 | 0.004 | 0.005 | 0.003 | 0.006 | 0.006 |
| meta1197 |  | 450.881 | 180.690 | 0.000 | 0.000 | 0.000 | 0.000 | 0.000 | 0.000 | 0.000 | 0.000 | 0.000 | 0.000 | 0.000 | 0.000 | 0.000 | 0.000 | 0.000 |
| meta1198 |  | 450.976 | 318.683 | 0.003 | 0.002 | 0.003 | 0.003 | 0.003 | 0.002 | 0.003 | 0.003 | 0.003 | 0.003 | 0.003 | 0.003 | 0.003 | 0.003 | 0.002 |
| meta1199 |  | 451.083 | 371.640 | 0.004 | 0.003 | 0.003 | 0.004 | 0.004 | 0.002 | 0.004 | 0.004 | 0.002 | 0.004 | 0.004 | 0.002 | 0.002 | 0.002 | 0.004 |
| meta1200 |  | 451.231 | 43.278 | 0.007 | 0.006 | 0.007 | 0.014 | 0.011 | 0.013 | 0.012 | 0.007 | 0.007 | 0.009 | 0.005 | 0.006 | 0.006 | 0.006 | 0.007 |
| meta1201 |  | 452.120 | 157.261 | 0.002 | 0.001 | 0.001 | 0.002 | 0.002 | 0.001 | 0.002 | 0.002 | 0.001 | 0.001 | 0.002 | 0.001 | 0.001 | 0.001 | 0.002 |
| meta1202 | 1-Palmitoyl-2-hydroxy-sn-glycero-3-phosphoethanolamine | 452.276 | 180.767 | 0.102 | 0.093 | 0.099 | 0.119 | 0.094 | 0.071 | 0.143 | 0.149 | 0.080 | 0.085 | 0.122 | 0.110 | 0.078 | 0.090 | 0.116 |
| meta1203 |  | 452.896 | 286.210 | 0.003 | 0.002 | 0.002 | 0.003 | 0.003 | 0.002 | 0.003 | 0.004 | 0.003 | 0.003 | 0.003 | 0.002 | 0.003 | 0.002 | 0.003 |
| meta1204 |  | 453.071 | 437.561 | 0.001 | 0.000 | 0.000 | 0.001 | 0.001 | 0.000 | 0.001 | 0.001 | 0.000 | 0.001 | 0.001 | 0.001 | 0.001 | 0.001 | 0.000 |
| meta1205 |  | 453.164 | 174.211 | 0.000 | 0.000 | 0.000 | 0.001 | 0.001 | 0.001 | 0.001 | 0.001 | 0.000 | 0.000 | 0.001 | 0.000 | 0.000 | 0.001 | 0.000 |
| meta1206 |  | 453.173 | 138.227 | 0.003 | 0.003 | 0.003 | 0.002 | 0.004 | 0.001 | 0.002 | 0.003 | 0.003 | 0.001 | 0.004 | 0.016 | 0.002 | 0.006 | 0.005 |
| meta1207 |  | 453.246 | 137.315 | 0.006 | 0.005 | 0.005 | 0.005 | 0.005 | 0.005 | 0.004 | 0.008 | 0.007 | 0.010 | 0.013 | 0.016 | 0.008 | 0.006 | 0.008 |
| meta1208 |  | 453.280 | 78.299 | 0.001 | 0.001 | 0.001 | 0.001 | 0.001 | 0.002 | 0.001 | 0.001 | 0.001 | 0.001 | 0.001 | 0.001 | 0.001 | 0.002 | 0.001 |
| meta1209 |  | 453.297 | 33.103 | 0.001 | 0.002 | 0.001 | 0.001 | 0.001 | 0.001 | 0.004 | 0.003 | 0.003 | 0.001 | 0.003 | 0.006 | 0.002 | 0.001 | 0.003 |
| meta1210 |  | 454.049 | 331.030 | 0.006 | 0.006 | 0.007 | 0.007 | 0.006 | 0.007 | 0.008 | 0.006 | 0.004 | 0.006 | 0.005 | 0.007 | 0.006 | 0.007 | 0.004 |
| meta1211 |  | 454.108 | 351.521 | 0.002 | 0.002 | 0.001 | 0.002 | 0.002 | 0.001 | 0.001 | 0.003 | 0.001 | 0.002 | 0.001 | 0.001 | 0.002 | 0.002 | 0.002 |
| meta1212 |  | 454.224 | 44.717 | 0.003 | 0.002 | 0.002 | 0.004 | 0.002 | 0.003 | 0.002 | 0.002 | 0.004 | 0.004 | 0.003 | 0.002 | 0.002 | 0.004 | 0.002 |
| meta1213 | Flavin mononucleotide (FMN) | 455.094 | 395.417 | 0.001 | 0.001 | 0.001 | 0.001 | 0.001 | 0.001 | 0.001 | 0.001 | 0.001 | 0.001 | 0.001 | 0.001 | 0.001 | 0.001 | 0.001 |
| meta1214 |  | 455.165 | 27.402 | 0.003 | 0.002 | 0.002 | 0.013 | 0.006 | 0.003 | 0.003 | 0.002 | 0.001 | 0.001 | 0.002 | 0.003 | 0.003 | 0.001 | 0.003 |
| meta1215 |  | 455.262 | 135.936 | 0.026 | 0.025 | 0.025 | 0.019 | 0.019 | 0.024 | 0.015 | 0.039 | 0.034 | 0.041 | 0.086 | 0.086 | 0.033 | 0.028 | 0.031 |
| meta1216 | Estradiol Cypionate | 455.283 | 180.590 | 0.000 | 0.000 | 0.000 | 0.001 | 0.000 | 0.000 | 0.001 | 0.001 | 0.000 | 0.000 | 0.001 | 0.001 | 0.000 | 0.000 | 0.001 |
| meta1217 |  | 456.151 | 333.070 | 0.001 | 0.001 | 0.001 | 0.001 | 0.000 | 0.001 | 0.001 | 0.002 | 0.001 | 0.000 | 0.000 | 0.000 | 0.000 | 0.001 | 0.000 |
| meta1218 |  | 456.828 | 578.116 | 0.012 | 0.010 | 0.009 | 0.012 | 0.011 | 0.008 | 0.010 | 0.011 | 0.010 | 0.011 | 0.010 | 0.011 | 0.010 | 0.010 | 0.010 |
| meta1219 |  | 456.856 | 286.423 | 0.001 | 0.001 | 0.000 | 0.001 | 0.001 | 0.001 | 0.001 | 0.001 | 0.001 | 0.001 | 0.001 | 0.001 | 0.000 | 0.001 | 0.001 |
| meta1220 |  | 457.007 | 26.031 | 0.004 | 0.003 | 0.006 | 0.005 | 0.001 | 0.005 | 0.009 | 0.002 | 0.004 | 0.004 | 0.005 | 0.005 | 0.005 | 0.008 | 0.005 |
| meta1221 |  | 457.093 | 284.228 | 0.007 | 0.004 | 0.005 | 0.008 | 0.006 | 0.005 | 0.009 | 0.006 | 0.003 | 0.004 | 0.004 | 0.005 | 0.005 | 0.004 | 0.005 |
| meta1222 | 1-Oleoyl-sn-glycerol 3-phosphate | 457.232 | 169.843 | 0.001 | 0.002 | 0.002 | 0.003 | 0.002 | 0.002 | 0.001 | 0.003 | 0.002 | 0.002 | 0.002 | 0.002 | 0.002 | 0.002 | 0.002 |
| meta1223 |  | 457.277 | 132.816 | 0.012 | 0.011 | 0.011 | 0.008 | 0.009 | 0.011 | 0.008 | 0.025 | 0.010 | 0.016 | 0.042 | 0.036 | 0.011 | 0.016 | 0.015 |
| meta1224 |  | 458.078 | 420.274 | 0.002 | 0.001 | 0.001 | 0.001 | 0.001 | 0.001 | 0.001 | 0.002 | 0.001 | 0.003 | 0.001 | 0.001 | 0.002 | 0.001 | 0.001 |
| meta1225 |  | 458.234 | 40.251 | 0.004 | 0.003 | 0.005 | 0.008 | 0.009 | 0.006 | 0.012 | 0.017 | 0.009 | 0.009 | 0.006 | 0.002 | 0.002 | 0.004 | 0.012 |
| meta1226 | L-Palmitoylcarnitine | 458.345 | 154.614 | 0.000 | 0.000 | 0.000 | 0.000 | 0.001 | 0.000 | 0.001 | 0.000 | 0.000 | 0.000 | 0.000 | 0.000 | 0.000 | 0.001 | 0.001 |
| meta1227 |  | 458.906 | 318.388 | 0.004 | 0.004 | 0.005 | 0.004 | 0.004 | 0.005 | 0.004 | 0.004 | 0.005 | 0.004 | 0.004 | 0.004 | 0.005 | 0.005 | 0.004 |
| meta1228 |  | 459.063 | 482.553 | 0.002 | 0.002 | 0.003 | 0.003 | 0.003 | 0.003 | 0.002 | 0.002 | 0.002 | 0.002 | 0.001 | 0.003 | 0.004 | 0.003 | 0.002 |
| meta1229 |  | 459.132 | 371.649 | 0.006 | 0.005 | 0.005 | 0.006 | 0.007 | 0.004 | 0.006 | 0.006 | 0.004 | 0.005 | 0.006 | 0.005 | 0.006 | 0.004 | 0.003 |
| meta1230 |  | 460.022 | 334.389 | 0.003 | 0.003 | 0.002 | 0.003 | 0.003 | 0.002 | 0.001 | 0.003 | 0.002 | 0.003 | 0.002 | 0.001 | 0.002 | 0.002 | 0.002 |
| meta1231 |  | 460.163 | 364.682 | 0.001 | 0.001 | 0.001 | 0.001 | 0.001 | 0.001 | 0.001 | 0.001 | 0.001 | 0.001 | 0.001 | 0.001 | 0.001 | 0.001 | 0.000 |
| meta1232 |  | 460.886 | 318.161 | 0.001 | 0.001 | 0.001 | 0.001 | 0.001 | 0.001 | 0.001 | 0.001 | 0.001 | 0.001 | 0.001 | 0.001 | 0.001 | 0.001 | 0.001 |
| meta1233 |  | 461.039 | 368.197 | 0.001 | 0.001 | 0.001 | 0.001 | 0.001 | 0.001 | 0.001 | 0.001 | 0.001 | 0.001 | 0.001 | 0.001 | 0.001 | 0.001 | 0.001 |
| meta1234 |  | 461.124 | 267.377 | 0.001 | 0.001 | 0.001 | 0.001 | 0.001 | 0.001 | 0.001 | 0.001 | 0.000 | 0.001 | 0.001 | 0.001 | 0.001 | 0.001 | 0.001 |
| meta1235 |  | 461.192 | 215.183 | 0.000 | 0.000 | 0.000 | 0.000 | 0.000 | 0.000 | 0.000 | 0.000 | 0.000 | 0.000 | 0.000 | 0.000 | 0.000 | 0.000 | 0.000 |
| meta1236 |  | 461.253 | 25.540 | 0.004 | 0.004 | 0.004 | 0.007 | 0.010 | 0.004 | 0.010 | 0.005 | 0.005 | 0.004 | 0.008 | 0.009 | 0.006 | 0.006 | 0.008 |
| meta1237 |  | 461.287 | 33.648 | 0.006 | 0.006 | 0.008 | 0.018 | 0.012 | 0.009 | 0.016 | 0.014 | 0.010 | 0.009 | 0.014 | 0.012 | 0.012 | 0.013 | 0.010 |
| meta1238 |  | 461.360 | 32.695 | 0.002 | 0.002 | 0.002 | 0.004 | 0.006 | 0.003 | 0.005 | 0.007 | 0.004 | 0.003 | 0.006 | 0.007 | 0.002 | 0.002 | 0.004 |
| meta1239 |  | 462.027 | 23.741 | 0.002 | 0.002 | 0.003 | 0.003 | 0.001 | 0.002 | 0.003 | 0.002 | 0.004 | 0.003 | 0.003 | 0.002 | 0.002 | 0.004 | 0.002 |
| meta1240 | Adenylsuccinic acid | 462.063 | 479.080 | 0.013 | 0.012 | 0.014 | 0.016 | 0.019 | 0.014 | 0.012 | 0.012 | 0.014 | 0.014 | 0.008 | 0.017 | 0.023 | 0.016 | 0.015 |
| meta1241 |  | 462.188 | 313.729 | 0.001 | 0.001 | 0.001 | 0.000 | 0.000 | 0.001 | 0.001 | 0.001 | 0.001 | 0.001 | 0.001 | 0.000 | 0.001 | 0.001 | 0.000 |
| meta1242 |  | 462.951 | 420.970 | 0.001 | 0.001 | 0.000 | 0.001 | 0.001 | 0.000 | 0.000 | 0.001 | 0.000 | 0.001 | 0.001 | 0.001 | 0.001 | 0.001 | 0.001 |
| meta1243 |  | 462.986 | 161.850 | 0.003 | 0.003 | 0.003 | 0.003 | 0.003 | 0.004 | 0.003 | 0.002 | 0.005 | 0.002 | 0.002 | 0.003 | 0.004 | 0.004 | 0.003 |
| meta1244 |  | 463.012 | 429.404 | 0.002 | 0.002 | 0.002 | 0.002 | 0.002 | 0.002 | 0.002 | 0.004 | 0.003 | 0.002 | 0.002 | 0.003 | 0.004 | 0.002 | 0.002 |
| meta1245 |  | 463.157 | 371.649 | 0.002 | 0.002 | 0.002 | 0.002 | 0.002 | 0.002 | 0.002 | 0.002 | 0.002 | 0.002 | 0.002 | 0.003 | 0.002 | 0.002 | 0.001 |
| meta1246 |  | 463.165 | 250.885 | 0.001 | 0.000 | 0.000 | 0.000 | 0.000 | 0.000 | 0.002 | 0.001 | 0.001 | 0.001 | 0.000 | 0.001 | 0.001 | 0.000 | 0.000 |
| meta1247 |  | 463.269 | 25.452 | 0.053 | 0.058 | 0.061 | 0.077 | 0.041 | 0.045 | 0.101 | 0.079 | 0.083 | 0.077 | 0.128 | 0.091 | 0.079 | 0.059 | 0.089 |
| meta1248 | Ascorbyl stearate | 463.279 | 170.933 | 0.001 | 0.001 | 0.001 | 0.002 | 0.001 | 0.001 | 0.002 | 0.002 | 0.001 | 0.001 | 0.002 | 0.001 | 0.001 | 0.001 | 0.002 |
| meta1249 |  | 464.094 | 468.259 | 0.004 | 0.003 | 0.003 | 0.005 | 0.005 | 0.004 | 0.003 | 0.005 | 0.003 | 0.009 | 0.003 | 0.005 | 0.006 | 0.003 | 0.003 |
| meta1250 | Glycocholic acid | 464.298 | 233.379 | 0.002 | 0.002 | 0.003 | 0.001 | 0.001 | 0.004 | 0.005 | 0.002 | 0.001 | 0.003 | 0.002 | 0.004 | 0.004 | 0.004 | 0.001 |
| meta1251 |  | 465.041 | 25.052 | 0.004 | 0.003 | 0.003 | 0.005 | 0.003 | 0.004 | 0.006 | 0.007 | 0.007 | 0.009 | 0.005 | 0.002 | 0.002 | 0.002 | 0.001 |
| meta1252 |  | 465.107 | 272.889 | 0.001 | 0.001 | 0.001 | 0.001 | 0.002 | 0.001 | 0.001 | 0.001 | 0.001 | 0.001 | 0.002 | 0.002 | 0.001 | 0.001 | 0.001 |
| meta1253 |  | 465.178 | 27.010 | 0.002 | 0.002 | 0.002 | 0.004 | 0.002 | 0.003 | 0.007 | 0.006 | 0.006 | 0.003 | 0.003 | 0.002 | 0.003 | 0.002 | 0.001 |
| meta1254 |  | 465.286 | 25.052 | 0.560 | 0.540 | 0.461 | 0.639 | 0.459 | 0.344 | 0.715 | 0.702 | 0.487 | 0.643 | 0.919 | 0.872 | 0.739 | 0.652 | 1.106 |
| meta1255 |  | 465.987 | 371.793 | 0.001 | 0.001 | 0.001 | 0.001 | 0.001 | 0.000 | 0.001 | 0.001 | 0.001 | 0.001 | 0.001 | 0.001 | 0.001 | 0.001 | 0.001 |
| meta1256 |  | 466.002 | 417.004 | 0.003 | 0.003 | 0.003 | 0.004 | 0.003 | 0.003 | 0.003 | 0.004 | 0.003 | 0.003 | 0.003 | 0.003 | 0.003 | 0.003 | 0.003 |
| meta1257 |  | 466.021 | 34.897 | 0.001 | 0.001 | 0.001 | 0.001 | 0.001 | 0.001 | 0.001 | 0.004 | 0.002 | 0.002 | 0.002 | 0.000 | 0.002 | 0.002 | 0.000 |
| meta1258 |  | 466.223 | 45.929 | 0.005 | 0.004 | 0.005 | 0.006 | 0.008 | 0.005 | 0.009 | 0.007 | 0.005 | 0.008 | 0.005 | 0.004 | 0.005 | 0.005 | 0.005 |
| meta1259 | Buprenorphine | 466.290 | 178.692 | 0.002 | 0.002 | 0.003 | 0.002 | 0.002 | 0.002 | 0.004 | 0.003 | 0.002 | 0.002 | 0.003 | 0.003 | 0.003 | 0.002 | 0.002 |
| meta1260 |  | 466.326 | 178.730 | 0.001 | 0.001 | 0.001 | 0.001 | 0.001 | 0.001 | 0.001 | 0.001 | 0.001 | 0.001 | 0.001 | 0.001 | 0.001 | 0.001 | 0.001 |
| meta1261 |  | 467.188 | 301.161 | 0.000 | 0.000 | 0.000 | 0.001 | 0.000 | 0.001 | 0.000 | 0.000 | 0.000 | 0.000 | 0.000 | 0.000 | 0.000 | 0.000 | 0.001 |
| meta1262 |  | 468.030 | 397.450 | 0.014 | 0.013 | 0.013 | 0.013 | 0.011 | 0.010 | 0.014 | 0.014 | 0.012 | 0.013 | 0.013 | 0.014 | 0.014 | 0.010 | 0.012 |
| meta1263 |  | 468.032 | 96.782 | 0.002 | 0.002 | 0.002 | 0.000 | 0.001 | 0.000 | 0.003 | 0.003 | 0.002 | 0.002 | 0.001 | 0.000 | 0.002 | 0.000 | 0.002 |
| meta1264 |  | 468.124 | 410.864 | 0.007 | 0.005 | 0.005 | 0.006 | 0.007 | 0.004 | 0.007 | 0.006 | 0.005 | 0.006 | 0.006 | 0.006 | 0.007 | 0.004 | 0.006 |
| meta1265 |  | 469.138 | 158.629 | 0.001 | 0.001 | 0.000 | 0.001 | 0.001 | 0.000 | 0.001 | 0.001 | 0.001 | 0.001 | 0.001 | 0.001 | 0.001 | 0.001 | 0.001 |
| meta1266 |  | 469.168 | 163.697 | 0.001 | 0.001 | 0.001 | 0.001 | 0.000 | 0.000 | 0.001 | 0.000 | 0.000 | 0.001 | 0.001 | 0.004 | 0.001 | 0.002 | 0.001 |
| meta1267 |  | 469.263 | 26.759 | 0.003 | 0.002 | 0.003 | 0.006 | 0.004 | 0.003 | 0.004 | 0.004 | 0.004 | 0.007 | 0.003 | 0.005 | 0.004 | 0.003 | 0.005 |
| meta1268 |  | 469.291 | 34.800 | 0.002 | 0.003 | 0.002 | 0.002 | 0.004 | 0.001 | 0.009 | 0.004 | 0.002 | 0.002 | 0.005 | 0.007 | 0.004 | 0.003 | 0.008 |
| meta1269 |  | 469.329 | 46.577 | 0.002 | 0.002 | 0.002 | 0.001 | 0.001 | 0.001 | 0.005 | 0.002 | 0.006 | 0.003 | 0.003 | 0.003 | 0.003 | 0.001 | 0.001 |
| meta1270 |  | 470.044 | 432.032 | 0.002 | 0.002 | 0.002 | 0.002 | 0.002 | 0.002 | 0.002 | 0.002 | 0.002 | 0.002 | 0.002 | 0.002 | 0.002 | 0.001 | 0.002 |
| meta1271 |  | 470.093 | 150.291 | 0.001 | 0.001 | 0.001 | 0.001 | 0.001 | 0.001 | 0.002 | 0.003 | 0.001 | 0.001 | 0.002 | 0.003 | 0.001 | 0.000 | 0.001 |
| meta1272 |  | 470.272 | 170.333 | 0.001 | 0.001 | 0.001 | 0.000 | 0.001 | 0.000 | 0.002 | 0.001 | 0.000 | 0.003 | 0.001 | 0.002 | 0.001 | 0.003 | 0.001 |
| meta1273 |  | 471.132 | 338.649 | 0.006 | 0.005 | 0.005 | 0.005 | 0.004 | 0.007 | 0.004 | 0.004 | 0.004 | 0.004 | 0.005 | 0.008 | 0.005 | 0.004 | 0.002 |
| meta1274 |  | 471.271 | 128.132 | 0.001 | 0.001 | 0.001 | 0.003 | 0.002 | 0.003 | 0.002 | 0.004 | 0.001 | 0.001 | 0.002 | 0.006 | 0.002 | 0.002 | 0.002 |
| meta1275 |  | 471.308 | 43.192 | 0.011 | 0.011 | 0.011 | 0.009 | 0.011 | 0.006 | 0.028 | 0.020 | 0.005 | 0.005 | 0.017 | 0.016 | 0.007 | 0.005 | 0.019 |
| meta1276 |  | 472.037 | 88.534 | 0.003 | 0.003 | 0.003 | 0.004 | 0.005 | 0.003 | 0.002 | 0.003 | 0.002 | 0.004 | 0.003 | 0.001 | 0.002 | 0.003 | 0.004 |
| meta1277 |  | 472.212 | 45.999 | 0.002 | 0.001 | 0.001 | 0.002 | 0.002 | 0.002 | 0.001 | 0.001 | 0.001 | 0.002 | 0.001 | 0.001 | 0.002 | 0.002 | 0.001 |
| meta1278 |  | 472.746 | 288.708 | 0.003 | 0.002 | 0.002 | 0.003 | 0.003 | 0.002 | 0.003 | 0.003 | 0.003 | 0.003 | 0.002 | 0.002 | 0.003 | 0.002 | 0.003 |
| meta1279 |  | 473.096 | 469.529 | 0.001 | 0.001 | 0.001 | 0.001 | 0.001 | 0.001 | 0.000 | 0.001 | 0.001 | 0.001 | 0.001 | 0.001 | 0.001 | 0.001 | 0.000 |
| meta1280 |  | 473.142 | 174.823 | 0.004 | 0.004 | 0.003 | 0.006 | 0.001 | 0.004 | 0.008 | 0.004 | 0.003 | 0.005 | 0.003 | 0.003 | 0.002 | 0.003 | 0.002 |
| meta1281 |  | 473.159 | 364.440 | 0.010 | 0.009 | 0.009 | 0.011 | 0.011 | 0.011 | 0.009 | 0.011 | 0.007 | 0.011 | 0.011 | 0.015 | 0.010 | 0.008 | 0.005 |
| meta1282 |  | 473.236 | 280.203 | 0.001 | 0.001 | 0.001 | 0.001 | 0.001 | 0.001 | 0.001 | 0.001 | 0.001 | 0.001 | 0.001 | 0.001 | 0.001 | 0.001 | 0.001 |
| meta1283 |  | 473.262 | 25.452 | 0.012 | 0.012 | 0.012 | 0.023 | 0.023 | 0.016 | 0.023 | 0.023 | 0.022 | 0.023 | 0.020 | 0.018 | 0.018 | 0.018 | 0.021 |
| meta1284 |  | 473.359 | 46.761 | 0.001 | 0.001 | 0.001 | 0.002 | 0.002 | 0.001 | 0.002 | 0.002 | 0.002 | 0.001 | 0.002 | 0.002 | 0.001 | 0.001 | 0.000 |
| meta1285 |  | 474.127 | 95.344 | 0.004 | 0.004 | 0.004 | 0.005 | 0.004 | 0.005 | 0.004 | 0.003 | 0.003 | 0.003 | 0.005 | 0.006 | 0.004 | 0.005 | 0.010 |
| meta1286 |  | 474.229 | 46.019 | 0.009 | 0.008 | 0.008 | 0.011 | 0.009 | 0.008 | 0.011 | 0.008 | 0.010 | 0.009 | 0.014 | 0.014 | 0.014 | 0.013 | 0.016 |
| meta1287 |  | 474.258 | 180.131 | 0.002 | 0.002 | 0.002 | 0.002 | 0.003 | 0.001 | 0.003 | 0.002 | 0.002 | 0.002 | 0.002 | 0.002 | 0.002 | 0.002 | 0.002 |
| meta1288 |  | 474.725 | 289.582 | 0.001 | 0.001 | 0.001 | 0.001 | 0.001 | 0.002 | 0.001 | 0.001 | 0.001 | 0.001 | 0.001 | 0.001 | 0.002 | 0.001 | 0.001 |
| meta1289 |  | 474.840 | 578.086 | 0.034 | 0.028 | 0.026 | 0.038 | 0.033 | 0.024 | 0.030 | 0.034 | 0.028 | 0.032 | 0.029 | 0.031 | 0.030 | 0.028 | 0.028 |
| meta1290 |  | 474.879 | 287.362 | 0.001 | 0.001 | 0.001 | 0.001 | 0.001 | 0.001 | 0.001 | 0.001 | 0.001 | 0.001 | 0.001 | 0.001 | 0.001 | 0.001 | 0.001 |
| meta1291 |  | 474.938 | 318.126 | 0.003 | 0.003 | 0.003 | 0.003 | 0.003 | 0.003 | 0.003 | 0.003 | 0.003 | 0.003 | 0.003 | 0.003 | 0.004 | 0.003 | 0.003 |
| meta1292 |  | 475.302 | 47.278 | 0.003 | 0.003 | 0.003 | 0.004 | 0.004 | 0.003 | 0.007 | 0.005 | 0.002 | 0.002 | 0.004 | 0.008 | 0.004 | 0.004 | 0.005 |
| meta1293 |  | 476.244 | 46.019 | 0.029 | 0.028 | 0.027 | 0.032 | 0.029 | 0.026 | 0.034 | 0.025 | 0.040 | 0.042 | 0.030 | 0.042 | 0.041 | 0.038 | 0.032 |
| meta1294 |  | 476.275 | 179.127 | 0.021 | 0.018 | 0.020 | 0.022 | 0.016 | 0.014 | 0.022 | 0.024 | 0.023 | 0.025 | 0.029 | 0.031 | 0.043 | 0.033 | 0.026 |
| meta1295 |  | 477.065 | 154.089 | 0.001 | 0.000 | 0.000 | 0.001 | 0.001 | 0.000 | 0.001 | 0.000 | 0.000 | 0.000 | 0.000 | 0.001 | 0.000 | 0.000 | 0.000 |
| meta1296 |  | 477.246 | 133.995 | 0.003 | 0.002 | 0.002 | 0.003 | 0.003 | 0.003 | 0.002 | 0.006 | 0.004 | 0.006 | 0.006 | 0.009 | 0.003 | 0.003 | 0.005 |
| meta1297 |  | 477.247 | 26.917 | 0.003 | 0.004 | 0.004 | 0.006 | 0.017 | 0.005 | 0.009 | 0.018 | 0.007 | 0.003 | 0.006 | 0.023 | 0.004 | 0.003 | 0.005 |
| meta1298 |  | 477.284 | 25.178 | 0.012 | 0.013 | 0.014 | 0.023 | 0.015 | 0.014 | 0.024 | 0.016 | 0.016 | 0.020 | 0.021 | 0.020 | 0.022 | 0.018 | 0.022 |
| meta1299 |  | 477.354 | 32.928 | 0.001 | 0.001 | 0.001 | 0.002 | 0.002 | 0.002 | 0.003 | 0.002 | 0.002 | 0.001 | 0.002 | 0.003 | 0.001 | 0.002 | 0.001 |
| meta1300 |  | 478.120 | 278.805 | 0.001 | 0.001 | 0.001 | 0.000 | 0.003 | 0.000 | 0.002 | 0.000 | 0.001 | 0.001 | 0.000 | 0.000 | 0.001 | 0.000 | 0.000 |
| meta1301 |  | 478.199 | 42.692 | 0.006 | 0.008 | 0.008 | 0.011 | 0.006 | 0.007 | 0.007 | 0.007 | 0.009 | 0.006 | 0.008 | 0.007 | 0.007 | 0.009 | 0.006 |
| meta1302 |  | 478.291 | 178.184 | 0.047 | 0.046 | 0.049 | 0.055 | 0.051 | 0.039 | 0.068 | 0.063 | 0.039 | 0.049 | 0.060 | 0.066 | 0.050 | 0.062 | 0.060 |
| meta1303 |  | 478.319 | 46.631 | 0.029 | 0.026 | 0.025 | 0.001 | 0.000 | 0.033 | 0.020 | 0.022 | 0.001 | 0.039 | 0.030 | 0.027 | 0.044 | 0.000 | 0.042 |
| meta1304 |  | 478.980 | 429.394 | 0.003 | 0.003 | 0.003 | 0.004 | 0.003 | 0.002 | 0.003 | 0.004 | 0.003 | 0.003 | 0.003 | 0.003 | 0.003 | 0.002 | 0.003 |
| meta1305 |  | 478.988 | 26.427 | 0.010 | 0.010 | 0.009 | 0.009 | 0.004 | 0.008 | 0.020 | 0.005 | 0.009 | 0.009 | 0.009 | 0.015 | 0.013 | 0.016 | 0.015 |
| meta1306 |  | 479.134 | 382.674 | 0.000 | 0.000 | 0.000 | 0.000 | 0.000 | 0.000 | 0.000 | 0.000 | 0.000 | 0.000 | 0.000 | 0.000 | 0.000 | 0.000 | 0.000 |
| meta1307 |  | 479.214 | 138.796 | 0.001 | 0.001 | 0.001 | 0.001 | 0.001 | 0.001 | 0.001 | 0.001 | 0.001 | 0.001 | 0.001 | 0.001 | 0.001 | 0.001 | 0.001 |
| meta1308 |  | 479.263 | 26.982 | 0.014 | 0.010 | 0.012 | 0.028 | 0.017 | 0.019 | 0.029 | 0.021 | 0.023 | 0.019 | 0.022 | 0.018 | 0.014 | 0.010 | 0.016 |
| meta1309 |  | 480.089 | 479.400 | 0.001 | 0.001 | 0.002 | 0.002 | 0.002 | 0.002 | 0.001 | 0.003 | 0.002 | 0.002 | 0.001 | 0.002 | 0.003 | 0.002 | 0.002 |
| meta1310 |  | 480.125 | 415.190 | 0.008 | 0.007 | 0.006 | 0.003 | 0.011 | 0.002 | 0.017 | 0.005 | 0.011 | 0.007 | 0.003 | 0.005 | 0.011 | 0.005 | 0.003 |
| meta1311 |  | 480.307 | 175.626 | 0.123 | 0.125 | 0.129 | 0.165 | 0.145 | 0.112 | 0.208 | 0.173 | 0.108 | 0.119 | 0.156 | 0.144 | 0.110 | 0.119 | 0.142 |
| meta1312 |  | 480.919 | 156.629 | 0.001 | 0.001 | 0.001 | 0.002 | 0.001 | 0.001 | 0.001 | 0.001 | 0.001 | 0.001 | 0.001 | 0.001 | 0.001 | 0.002 | 0.001 |
| meta1313 |  | 481.161 | 351.554 | 0.005 | 0.004 | 0.004 | 0.005 | 0.005 | 0.004 | 0.005 | 0.006 | 0.006 | 0.005 | 0.005 | 0.004 | 0.006 | 0.004 | 0.004 |
| meta1314 |  | 481.204 | 294.101 | 0.002 | 0.002 | 0.002 | 0.004 | 0.001 | 0.004 | 0.002 | 0.002 | 0.001 | 0.001 | 0.002 | 0.001 | 0.002 | 0.002 | 0.002 |
| meta1315 |  | 481.232 | 168.341 | 0.001 | 0.001 | 0.001 | 0.001 | 0.001 | 0.001 | 0.001 | 0.001 | 0.001 | 0.001 | 0.001 | 0.001 | 0.001 | 0.001 | 0.001 |
| meta1316 |  | 481.254 | 134.505 | 0.004 | 0.003 | 0.004 | 0.007 | 0.005 | 0.005 | 0.007 | 0.002 | 0.002 | 0.004 | 0.006 | 0.004 | 0.003 | 0.003 | 0.003 |
| meta1317 |  | 481.279 | 26.870 | 0.025 | 0.023 | 0.026 | 0.051 | 0.040 | 0.034 | 0.043 | 0.032 | 0.019 | 0.035 | 0.030 | 0.032 | 0.035 | 0.020 | 0.049 |
| meta1318 |  | 482.200 | 46.524 | 0.003 | 0.003 | 0.002 | 0.003 | 0.002 | 0.002 | 0.002 | 0.001 | 0.002 | 0.002 | 0.002 | 0.001 | 0.002 | 0.002 | 0.003 |
| meta1319 | Taurolithocholic acid | 482.291 | 46.552 | 0.015 | 0.013 | 0.013 | 0.015 | 0.017 | 0.011 | 0.020 | 0.015 | 0.007 | 0.017 | 0.026 | 0.022 | 0.020 | 0.017 | 0.028 |
| meta1320 |  | 482.868 | 318.133 | 0.003 | 0.002 | 0.003 | 0.003 | 0.003 | 0.003 | 0.003 | 0.003 | 0.003 | 0.003 | 0.003 | 0.002 | 0.003 | 0.003 | 0.003 |
| meta1321 |  | 483.040 | 386.922 | 0.001 | 0.001 | 0.001 | 0.001 | 0.001 | 0.001 | 0.001 | 0.001 | 0.001 | 0.001 | 0.001 | 0.001 | 0.001 | 0.001 | 0.001 |
| meta1322 |  | 483.036 | 33.622 | 0.000 | 0.000 | 0.000 | 0.001 | 0.000 | 0.000 | 0.001 | 0.000 | 0.000 | 0.000 | 0.000 | 0.000 | 0.000 | 0.000 | 0.000 |
| meta1323 |  | 483.293 | 211.612 | 0.001 | 0.001 | 0.001 | 0.000 | 0.001 | 0.000 | 0.001 | 0.001 | 0.000 | 0.001 | 0.001 | 0.003 | 0.001 | 0.001 | 0.001 |
| meta1324 |  | 483.308 | 46.684 | 0.028 | 0.027 | 0.025 | 0.026 | 0.015 | 0.017 | 0.038 | 0.022 | 0.095 | 0.049 | 0.065 | 0.026 | 0.097 | 0.027 | 0.038 |
| meta1325 |  | 484.024 | 430.839 | 0.003 | 0.002 | 0.003 | 0.003 | 0.002 | 0.002 | 0.002 | 0.003 | 0.002 | 0.003 | 0.003 | 0.003 | 0.003 | 0.002 | 0.003 |
| meta1326 |  | 484.110 | 152.801 | 0.002 | 0.001 | 0.001 | 0.001 | 0.002 | 0.001 | 0.002 | 0.001 | 0.001 | 0.001 | 0.001 | 0.002 | 0.001 | 0.001 | 0.001 |
| meta1327 |  | 484.876 | 613.226 | 0.012 | 0.009 | 0.011 | 0.014 | 0.004 | 0.007 | 0.012 | 0.004 | 0.015 | 0.006 | 0.008 | 0.011 | 0.016 | 0.008 | 0.005 |
| meta1328 |  | 485.130 | 587.724 | 0.027 | 0.027 | 0.022 | 0.035 | 0.029 | 0.016 | 0.029 | 0.031 | 0.024 | 0.031 | 0.031 | 0.023 | 0.025 | 0.021 | 0.026 |
| meta1329 |  | 485.323 | 93.375 | 0.004 | 0.004 | 0.004 | 0.005 | 0.001 | 0.003 | 0.006 | 0.003 | 0.025 | 0.010 | 0.007 | 0.005 | 0.006 | 0.002 | 0.003 |
| meta1330 |  | 485.972 | 86.066 | 0.004 | 0.004 | 0.005 | 0.006 | 0.006 | 0.005 | 0.005 | 0.003 | 0.004 | 0.003 | 0.004 | 0.002 | 0.004 | 0.004 | 0.006 |
| meta1331 | Adenosine 5'-phosphosulfate (APS) | 486.039 | 424.133 | 0.002 | 0.001 | 0.002 | 0.002 | 0.001 | 0.001 | 0.003 | 0.002 | 0.002 | 0.002 | 0.002 | 0.002 | 0.003 | 0.002 | 0.002 |
| meta1332 |  | 486.180 | 139.467 | 0.001 | 0.001 | 0.001 | 0.001 | 0.001 | 0.001 | 0.001 | 0.001 | 0.000 | 0.001 | 0.001 | 0.001 | 0.001 | 0.001 | 0.001 |
| meta1333 | Cytidine 5'-diphosphocholine (CDP-choline) | 487.097 | 426.779 | 0.011 | 0.011 | 0.012 | 0.015 | 0.012 | 0.010 | 0.014 | 0.012 | 0.011 | 0.010 | 0.010 | 0.010 | 0.012 | 0.010 | 0.010 |
| meta1334 |  | 487.127 | 369.567 | 0.001 | 0.001 | 0.002 | 0.001 | 0.002 | 0.002 | 0.001 | 0.001 | 0.001 | 0.001 | 0.001 | 0.002 | 0.002 | 0.001 | 0.001 |
| meta1335 | 3,3',4,5'-Tetrahydroxy-trans-stilbene | 487.129 | 150.817 | 0.115 | 0.090 | 0.095 | 0.112 | 0.202 | 0.082 | 0.256 | 0.129 | 0.046 | 0.031 | 0.058 | 0.107 | 0.060 | 0.069 | 0.072 |
| meta1336 |  | 487.175 | 363.920 | 0.053 | 0.047 | 0.051 | 0.060 | 0.055 | 0.064 | 0.043 | 0.053 | 0.039 | 0.056 | 0.053 | 0.059 | 0.048 | 0.045 | 0.024 |
| meta1337 |  | 487.192 | 45.294 | 0.002 | 0.002 | 0.003 | 0.003 | 0.002 | 0.004 | 0.003 | 0.007 | 0.006 | 0.006 | 0.003 | 0.001 | 0.001 | 0.001 | 0.001 |
| meta1338 |  | 487.234 | 258.183 | 0.001 | 0.001 | 0.001 | 0.000 | 0.000 | 0.000 | 0.002 | 0.000 | 0.001 | 0.002 | 0.000 | 0.001 | 0.001 | 0.002 | 0.000 |
| meta1339 |  | 487.339 | 115.071 | 0.006 | 0.006 | 0.005 | 0.008 | 0.002 | 0.006 | 0.012 | 0.010 | 0.036 | 0.013 | 0.009 | 0.009 | 0.011 | 0.002 | 0.004 |
| meta1340 |  | 488.778 | 288.238 | 0.002 | 0.002 | 0.002 | 0.002 | 0.002 | 0.002 | 0.002 | 0.002 | 0.002 | 0.002 | 0.002 | 0.002 | 0.002 | 0.002 | 0.002 |
| meta1341 |  | 489.235 | 182.541 | 0.001 | 0.001 | 0.001 | 0.001 | 0.002 | 0.000 | 0.001 | 0.001 | 0.001 | 0.000 | 0.001 | 0.002 | 0.000 | 0.001 | 0.001 |
| meta1342 |  | 489.318 | 134.770 | 0.003 | 0.003 | 0.004 | 0.005 | 0.004 | 0.004 | 0.005 | 0.006 | 0.001 | 0.001 | 0.003 | 0.010 | 0.004 | 0.002 | 0.002 |
| meta1343 |  | 490.011 | 397.413 | 0.002 | 0.002 | 0.002 | 0.002 | 0.002 | 0.001 | 0.002 | 0.002 | 0.002 | 0.002 | 0.002 | 0.002 | 0.002 | 0.001 | 0.002 |
| meta1344 |  | 490.853 | 287.061 | 0.011 | 0.007 | 0.008 | 0.014 | 0.014 | 0.010 | 0.012 | 0.013 | 0.013 | 0.012 | 0.010 | 0.012 | 0.016 | 0.011 | 0.010 |
| meta1345 |  | 491.066 | 97.442 | 0.002 | 0.001 | 0.002 | 0.000 | 0.000 | 0.000 | 0.014 | 0.021 | 0.008 | 0.002 | 0.000 | 0.000 | 0.001 | 0.000 | 0.000 |
| meta1346 |  | 491.211 | 33.547 | 0.001 | 0.001 | 0.001 | 0.001 | 0.001 | 0.001 | 0.002 | 0.001 | 0.001 | 0.001 | 0.001 | 0.001 | 0.001 | 0.001 | 0.001 |
| meta1347 |  | 491.290 | 104.536 | 0.002 | 0.002 | 0.002 | 0.001 | 0.001 | 0.001 | 0.006 | 0.003 | 0.002 | 0.002 | 0.002 | 0.002 | 0.002 | 0.001 | 0.002 |
| meta1348 |  | 491.299 | 24.905 | 0.003 | 0.005 | 0.005 | 0.007 | 0.004 | 0.003 | 0.007 | 0.005 | 0.006 | 0.004 | 0.007 | 0.007 | 0.006 | 0.005 | 0.006 |
| meta1349 |  | 492.116 | 275.524 | 0.007 | 0.007 | 0.007 | 0.009 | 0.008 | 0.007 | 0.007 | 0.007 | 0.006 | 0.008 | 0.006 | 0.006 | 0.007 | 0.007 | 0.008 |
| meta1350 |  | 492.118 | 322.711 | 0.004 | 0.003 | 0.002 | 0.003 | 0.005 | 0.003 | 0.003 | 0.002 | 0.001 | 0.003 | 0.003 | 0.005 | 0.002 | 0.003 | 0.002 |
| meta1351 |  | 492.133 | 24.549 | 0.001 | 0.001 | 0.000 | 0.001 | 0.000 | 0.001 | 0.001 | 0.001 | 0.001 | 0.001 | 0.001 | 0.001 | 0.001 | 0.001 | 0.000 |
| meta1352 |  | 492.224 | 135.286 | 0.002 | 0.001 | 0.002 | 0.003 | 0.001 | 0.002 | 0.003 | 0.004 | 0.004 | 0.001 | 0.001 | 0.001 | 0.001 | 0.002 | 0.001 |
| meta1353 |  | 492.239 | 119.611 | 0.008 | 0.006 | 0.006 | 0.008 | 0.004 | 0.007 | 0.008 | 0.009 | 0.003 | 0.008 | 0.008 | 0.008 | 0.008 | 0.008 | 0.015 |
| meta1354 |  | 492.276 | 45.294 | 0.029 | 0.027 | 0.031 | 0.035 | 0.051 | 0.032 | 0.026 | 0.042 | 0.038 | 0.029 | 0.040 | 0.037 | 0.031 | 0.044 | 0.059 |
| meta1355 |  | 492.850 | 578.604 | 0.005 | 0.005 | 0.004 | 0.006 | 0.005 | 0.004 | 0.005 | 0.005 | 0.005 | 0.004 | 0.004 | 0.004 | 0.005 | 0.005 | 0.005 |
| meta1356 |  | 493.350 | 46.053 | 0.001 | 0.001 | 0.001 | 0.002 | 0.001 | 0.001 | 0.002 | 0.001 | 0.002 | 0.001 | 0.001 | 0.001 | 0.001 | 0.001 | 0.001 |
| meta1357 |  | 494.322 | 174.155 | 0.004 | 0.004 | 0.004 | 0.005 | 0.004 | 0.004 | 0.007 | 0.005 | 0.004 | 0.005 | 0.005 | 0.005 | 0.004 | 0.004 | 0.005 |
| meta1358 |  | 495.040 | 343.810 | 0.001 | 0.001 | 0.000 | 0.000 | 0.001 | 0.000 | 0.000 | 0.008 | 0.001 | 0.001 | 0.003 | 0.004 | 0.000 | 0.000 | 0.001 |
| meta1359 |  | 495.039 | 428.707 | 0.004 | 0.005 | 0.006 | 0.005 | 0.004 | 0.004 | 0.005 | 0.007 | 0.006 | 0.005 | 0.004 | 0.007 | 0.009 | 0.004 | 0.006 |
| meta1360 |  | 495.048 | 284.465 | 0.003 | 0.002 | 0.002 | 0.003 | 0.003 | 0.002 | 0.003 | 0.003 | 0.002 | 0.002 | 0.002 | 0.002 | 0.002 | 0.002 | 0.002 |
| meta1361 |  | 495.115 | 150.855 | 0.015 | 0.014 | 0.012 | 0.013 | 0.020 | 0.009 | 0.019 | 0.016 | 0.006 | 0.005 | 0.009 | 0.013 | 0.008 | 0.010 | 0.010 |
| meta1362 |  | 495.115 | 332.005 | 0.003 | 0.003 | 0.003 | 0.002 | 0.001 | 0.002 | 0.005 | 0.001 | 0.004 | 0.003 | 0.007 | 0.003 | 0.003 | 0.002 | 0.001 |
| meta1363 |  | 495.191 | 398.605 | 0.001 | 0.001 | 0.001 | 0.002 | 0.002 | 0.002 | 0.001 | 0.002 | 0.001 | 0.001 | 0.001 | 0.002 | 0.001 | 0.001 | 0.001 |
| meta1364 |  | 495.258 | 26.563 | 0.014 | 0.015 | 0.016 | 0.028 | 0.018 | 0.020 | 0.043 | 0.030 | 0.021 | 0.025 | 0.041 | 0.042 | 0.032 | 0.018 | 0.032 |
| meta1365 | Enterostatin human | 495.275 | 32.613 | 0.005 | 0.006 | 0.006 | 0.010 | 0.007 | 0.007 | 0.010 | 0.007 | 0.006 | 0.008 | 0.006 | 0.007 | 0.008 | 0.006 | 0.007 |
| meta1366 |  | 496.175 | 367.887 | 0.006 | 0.008 | 0.009 | 0.010 | 0.013 | 0.012 | 0.010 | 0.013 | 0.013 | 0.014 | 0.008 | 0.015 | 0.014 | 0.010 | 0.005 |
| meta1367 |  | 496.265 | 225.862 | 0.002 | 0.002 | 0.002 | 0.002 | 0.002 | 0.002 | 0.003 | 0.003 | 0.001 | 0.002 | 0.002 | 0.002 | 0.002 | 0.002 | 0.002 |
| meta1368 |  | 496.270 | 132.659 | 0.059 | 0.049 | 0.055 | 0.014 | 0.018 | 0.042 | 0.072 | 0.068 | 0.029 | 0.145 | 0.084 | 0.049 | 0.106 | 0.110 | 0.041 |
| meta1369 |  | 496.751 | 136.101 | 0.001 | 0.001 | 0.002 | 0.001 | 0.001 | 0.002 | 0.002 | 0.002 | 0.001 | 0.003 | 0.002 | 0.001 | 0.001 | 0.002 | 0.001 |
| meta1370 |  | 496.791 | 288.699 | 0.001 | 0.002 | 0.002 | 0.002 | 0.001 | 0.001 | 0.002 | 0.001 | 0.001 | 0.002 | 0.001 | 0.002 | 0.002 | 0.002 | 0.002 |
| meta1371 |  | 496.990 | 429.463 | 0.002 | 0.002 | 0.002 | 0.002 | 0.002 | 0.001 | 0.002 | 0.002 | 0.002 | 0.001 | 0.002 | 0.001 | 0.001 | 0.001 | 0.002 |
| meta1372 |  | 497.072 | 223.672 | 0.001 | 0.001 | 0.001 | 0.001 | 0.001 | 0.002 | 0.002 | 0.004 | 0.002 | 0.002 | 0.001 | 0.001 | 0.001 | 0.001 | 0.001 |
| meta1373 |  | 497.162 | 94.836 | 0.006 | 0.004 | 0.005 | 0.009 | 0.014 | 0.008 | 0.014 | 0.006 | 0.002 | 0.002 | 0.004 | 0.003 | 0.004 | 0.005 | 0.005 |
| meta1374 |  | 497.195 | 253.503 | 0.000 | 0.000 | 0.000 | 0.000 | 0.000 | 0.000 | 0.000 | 0.000 | 0.000 | 0.000 | 0.000 | 0.000 | 0.000 | 0.000 | 0.000 |
| meta1375 |  | 497.237 | 155.334 | 0.001 | 0.000 | 0.000 | 0.000 | 0.002 | 0.000 | 0.001 | 0.000 | 0.000 | 0.000 | 0.000 | 0.000 | 0.000 | 0.000 | 0.000 |
| meta1376 |  | 498.040 | 414.050 | 0.005 | 0.004 | 0.004 | 0.005 | 0.005 | 0.003 | 0.005 | 0.005 | 0.004 | 0.004 | 0.004 | 0.006 | 0.005 | 0.003 | 0.004 |
| meta1377 |  | 498.233 | 46.524 | 0.001 | 0.001 | 0.001 | 0.001 | 0.000 | 0.000 | 0.001 | 0.001 | 0.001 | 0.001 | 0.001 | 0.001 | 0.001 | 0.001 | 0.001 |
| meta1378 |  | 498.259 | 178.038 | 0.001 | 0.001 | 0.001 | 0.002 | 0.001 | 0.001 | 0.001 | 0.002 | 0.002 | 0.003 | 0.003 | 0.002 | 0.002 | 0.002 | 0.004 |
| meta1379 | Taurochenodeoxycholate | 498.287 | 135.997 | 0.684 | 0.693 | 0.686 | 0.447 | 0.285 | 0.739 | 1.216 | 0.753 | 0.240 | 1.319 | 1.217 | 0.488 | 0.871 | 0.930 | 0.619 |
| meta1380 |  | 498.898 | 318.143 | 0.002 | 0.002 | 0.002 | 0.002 | 0.002 | 0.002 | 0.002 | 0.002 | 0.002 | 0.002 | 0.002 | 0.002 | 0.002 | 0.002 | 0.002 |
| meta1381 |  | 498.927 | 21.720 | 0.002 | 0.001 | 0.002 | 0.004 | 0.004 | 0.002 | 0.003 | 0.003 | 0.003 | 0.002 | 0.003 | 0.004 | 0.004 | 0.003 | 0.003 |
| meta1382 |  | 499.048 | 273.911 | 0.027 | 0.022 | 0.021 | 0.030 | 0.031 | 0.024 | 0.020 | 0.034 | 0.020 | 0.039 | 0.021 | 0.013 | 0.024 | 0.024 | 0.022 |
| meta1383 |  | 499.146 | 587.208 | 0.029 | 0.031 | 0.025 | 0.039 | 0.032 | 0.023 | 0.030 | 0.035 | 0.008 | 0.031 | 0.035 | 0.026 | 0.025 | 0.024 | 0.026 |
| meta1384 |  | 499.197 | 40.064 | 0.005 | 0.006 | 0.006 | 0.011 | 0.011 | 0.007 | 0.006 | 0.003 | 0.002 | 0.004 | 0.003 | 0.008 | 0.003 | 0.014 | 0.003 |
| meta1385 |  | 499.304 | 47.969 | 0.117 | 0.115 | 0.116 | 0.071 | 0.055 | 0.072 | 0.149 | 0.068 | 0.111 | 0.064 | 0.190 | 0.160 | 0.333 | 0.126 | 0.104 |
| meta1386 |  | 499.303 | 215.055 | 0.002 | 0.002 | 0.002 | 0.002 | 0.000 | 0.003 | 0.006 | 0.001 | 0.002 | 0.006 | 0.003 | 0.007 | 0.010 | 0.002 | 0.001 |
| meta1387 |  | 499.303 | 90.658 | 0.018 | 0.020 | 0.023 | 0.021 | 0.023 | 0.016 | 0.040 | 0.016 | 0.025 | 0.014 | 0.045 | 0.032 | 0.154 | 0.029 | 0.062 |
| meta1388 |  | 499.364 | 45.839 | 0.001 | 0.001 | 0.001 | 0.001 | 0.001 | 0.001 | 0.001 | 0.001 | 0.001 | 0.001 | 0.001 | 0.001 | 0.000 | 0.001 | 0.000 |
| meta1389 |  | 500.275 | 175.928 | 0.058 | 0.056 | 0.061 | 0.073 | 0.068 | 0.061 | 0.064 | 0.078 | 0.068 | 0.078 | 0.099 | 0.084 | 0.091 | 0.097 | 0.083 |
| meta1390 |  | 501.128 | 465.350 | 0.002 | 0.002 | 0.002 | 0.002 | 0.003 | 0.002 | 0.002 | 0.003 | 0.002 | 0.002 | 0.001 | 0.002 | 0.004 | 0.002 | 0.001 |
| meta1391 |  | 501.143 | 345.786 | 0.028 | 0.031 | 0.031 | 0.037 | 0.026 | 0.038 | 0.028 | 0.028 | 0.019 | 0.025 | 0.027 | 0.035 | 0.024 | 0.023 | 0.015 |
| meta1392 |  | 501.143 | 378.313 | 0.005 | 0.004 | 0.005 | 0.005 | 0.004 | 0.007 | 0.005 | 0.005 | 0.003 | 0.005 | 0.005 | 0.006 | 0.004 | 0.004 | 0.003 |
| meta1393 |  | 501.177 | 85.540 | 0.005 | 0.005 | 0.005 | 0.006 | 0.006 | 0.004 | 0.004 | 0.003 | 0.003 | 0.004 | 0.005 | 0.003 | 0.004 | 0.003 | 0.005 |
| meta1394 |  | 501.191 | 364.720 | 0.001 | 0.001 | 0.000 | 0.001 | 0.001 | 0.000 | 0.001 | 0.001 | 0.000 | 0.001 | 0.001 | 0.001 | 0.001 | 0.000 | 0.000 |
| meta1395 |  | 501.319 | 102.203 | 0.200 | 0.135 | 0.185 | 0.109 | 0.056 | 0.041 | 0.334 | 0.313 | 0.253 | 0.172 | 0.254 | 0.303 | 0.268 | 0.115 | 0.112 |
| meta1396 |  | 501.319 | 79.703 | 0.037 | 0.036 | 0.030 | 0.016 | 0.004 | 0.010 | 0.009 | 0.074 | 0.086 | 0.102 | 0.028 | 0.074 | 0.075 | 0.016 | 0.006 |
| meta1397 |  | 501.392 | 46.638 | 0.001 | 0.001 | 0.001 | 0.002 | 0.001 | 0.002 | 0.001 | 0.002 | 0.002 | 0.001 | 0.001 | 0.001 | 0.001 | 0.001 | 0.001 |
| meta1398 |  | 502.007 | 416.153 | 0.002 | 0.002 | 0.003 | 0.002 | 0.002 | 0.002 | 0.003 | 0.004 | 0.002 | 0.002 | 0.003 | 0.004 | 0.002 | 0.003 | 0.004 |
| meta1399 |  | 502.107 | 436.163 | 0.002 | 0.002 | 0.002 | 0.002 | 0.002 | 0.002 | 0.002 | 0.002 | 0.002 | 0.002 | 0.002 | 0.002 | 0.002 | 0.002 | 0.001 |
| meta1400 |  | 502.199 | 41.377 | 0.006 | 0.009 | 0.010 | 0.009 | 0.005 | 0.009 | 0.007 | 0.006 | 0.009 | 0.009 | 0.007 | 0.011 | 0.012 | 0.013 | 0.008 |
| meta1401 |  | 503.144 | 350.963 | 0.002 | 0.002 | 0.003 | 0.002 | 0.002 | 0.002 | 0.002 | 0.002 | 0.003 | 0.002 | 0.002 | 0.002 | 0.002 | 0.002 | 0.002 |
| meta1402 | Raffinose | 503.158 | 468.881 | 0.002 | 0.001 | 0.002 | 0.002 | 0.001 | 0.002 | 0.002 | 0.002 | 0.001 | 0.002 | 0.002 | 0.002 | 0.002 | 0.001 | 0.001 |
| meta1403 |  | 503.262 | 128.283 | 0.010 | 0.006 | 0.010 | 0.010 | 0.012 | 0.010 | 0.008 | 0.020 | 0.008 | 0.010 | 0.036 | 0.015 | 0.007 | 0.013 | 0.014 |
| meta1404 |  | 503.298 | 48.606 | 0.006 | 0.006 | 0.006 | 0.008 | 0.007 | 0.006 | 0.009 | 0.007 | 0.003 | 0.005 | 0.006 | 0.010 | 0.006 | 0.006 | 0.007 |
| meta1405 |  | 504.089 | 459.337 | 0.002 | 0.002 | 0.002 | 0.002 | 0.002 | 0.002 | 0.001 | 0.004 | 0.002 | 0.004 | 0.002 | 0.003 | 0.003 | 0.001 | 0.002 |
| meta1406 |  | 504.143 | 482.082 | 0.001 | 0.001 | 0.001 | 0.001 | 0.001 | 0.001 | 0.001 | 0.001 | 0.000 | 0.000 | 0.000 | 0.001 | 0.001 | 0.001 | 0.001 |
| meta1407 |  | 505.131 | 47.247 | 0.001 | 0.001 | 0.000 | 0.001 | 0.001 | 0.001 | 0.001 | 0.001 | 0.000 | 0.001 | 0.001 | 0.001 | 0.001 | 0.001 | 0.001 |
| meta1408 |  | 505.136 | 279.334 | 0.001 | 0.001 | 0.001 | 0.002 | 0.001 | 0.001 | 0.001 | 0.001 | 0.001 | 0.002 | 0.001 | 0.001 | 0.002 | 0.002 | 0.001 |
| meta1409 |  | 505.149 | 94.776 | 0.000 | 0.001 | 0.000 | 0.001 | 0.001 | 0.001 | 0.002 | 0.000 | 0.000 | 0.000 | 0.000 | 0.000 | 0.001 | 0.001 | 0.001 |
| meta1410 |  | 505.277 | 25.140 | 0.001 | 0.002 | 0.002 | 0.004 | 0.002 | 0.003 | 0.005 | 0.004 | 0.003 | 0.003 | 0.004 | 0.003 | 0.002 | 0.002 | 0.004 |
| meta1411 |  | 506.099 | 411.129 | 0.004 | 0.004 | 0.004 | 0.005 | 0.004 | 0.004 | 0.004 | 0.004 | 0.004 | 0.004 | 0.005 | 0.004 | 0.004 | 0.003 | 0.003 |
| meta1412 |  | 506.322 | 171.560 | 0.007 | 0.006 | 0.007 | 0.007 | 0.007 | 0.006 | 0.009 | 0.009 | 0.005 | 0.008 | 0.007 | 0.009 | 0.007 | 0.009 | 0.009 |
| meta1413 |  | 506.829 | 318.126 | 0.002 | 0.001 | 0.002 | 0.002 | 0.002 | 0.002 | 0.002 | 0.002 | 0.002 | 0.002 | 0.002 | 0.002 | 0.002 | 0.003 | 0.002 |
| meta1414 | 17.beta.-Estradiol 3-.beta.-D-glucuronide | 507.221 | 278.395 | 0.000 | 0.000 | 0.000 | 0.000 | 0.000 | 0.000 | 0.000 | 0.000 | 0.000 | 0.000 | 0.000 | 0.000 | 0.000 | 0.000 | 0.000 |
| meta1415 |  | 507.269 | 132.177 | 0.046 | 0.043 | 0.047 | 0.073 | 0.064 | 0.048 | 0.080 | 0.050 | 0.040 | 0.058 | 0.065 | 0.070 | 0.069 | 0.048 | 0.040 |
| meta1416 |  | 507.366 | 144.292 | 0.003 | 0.002 | 0.003 | 0.003 | 0.002 | 0.003 | 0.003 | 0.003 | 0.003 | 0.002 | 0.004 | 0.003 | 0.002 | 0.002 | 0.002 |
| meta1417 |  | 508.194 | 352.165 | 0.001 | 0.001 | 0.001 | 0.001 | 0.001 | 0.000 | 0.001 | 0.001 | 0.001 | 0.001 | 0.001 | 0.001 | 0.001 | 0.001 | 0.001 |
| meta1418 |  | 508.338 | 170.961 | 0.025 | 0.026 | 0.027 | 0.032 | 0.030 | 0.021 | 0.040 | 0.033 | 0.023 | 0.023 | 0.029 | 0.028 | 0.025 | 0.020 | 0.039 |
| meta1419 |  | 509.110 | 150.812 | 0.028 | 0.024 | 0.024 | 0.032 | 0.047 | 0.023 | 0.053 | 0.030 | 0.013 | 0.009 | 0.017 | 0.026 | 0.018 | 0.020 | 0.019 |
| meta1420 |  | 509.236 | 26.756 | 0.002 | 0.003 | 0.002 | 0.003 | 0.004 | 0.002 | 0.003 | 0.004 | 0.001 | 0.003 | 0.004 | 0.003 | 0.002 | 0.002 | 0.004 |
| meta1421 |  | 509.309 | 26.982 | 0.008 | 0.009 | 0.010 | 0.024 | 0.014 | 0.015 | 0.026 | 0.019 | 0.020 | 0.016 | 0.021 | 0.024 | 0.016 | 0.012 | 0.019 |
| meta1422 |  | 509.327 | 31.598 | 0.005 | 0.003 | 0.005 | 0.004 | 0.008 | 0.003 | 0.004 | 0.007 | 0.005 | 0.003 | 0.006 | 0.006 | 0.002 | 0.004 | 0.007 |
| meta1423 |  | 510.250 | 100.560 | 0.041 | 0.025 | 0.026 | 0.011 | 0.024 | 0.047 | 0.068 | 0.076 | 0.006 | 0.090 | 0.026 | 0.040 | 0.076 | 0.028 | 0.053 |
| meta1424 |  | 510.685 | 124.973 | 0.003 | 0.003 | 0.003 | 0.001 | 0.000 | 0.002 | 0.008 | 0.004 | 0.000 | 0.005 | 0.003 | 0.005 | 0.006 | 0.003 | 0.002 |
| meta1425 | 3-Acetyl-11-keto-.beta.-boswellic acid | 511.342 | 32.141 | 0.002 | 0.002 | 0.003 | 0.007 | 0.004 | 0.002 | 0.004 | 0.003 | 0.005 | 0.002 | 0.003 | 0.005 | 0.002 | 0.004 | 0.004 |
| meta1426 |  | 512.266 | 163.068 | 0.121 | 0.111 | 0.105 | 0.014 | 0.027 | 0.071 | 0.390 | 0.089 | 0.049 | 0.111 | 0.189 | 0.150 | 0.158 | 0.184 | 0.061 |
| meta1427 |  | 512.267 | 124.973 | 1.508 | 1.315 | 1.308 | 0.272 | 0.188 | 1.549 | 3.301 | 1.673 | 0.216 | 2.564 | 1.377 | 2.592 | 2.642 | 2.235 | 0.817 |
| meta1428 |  | 512.305 | 186.615 | 0.000 | 0.001 | 0.001 | 0.001 | 0.001 | 0.001 | 0.000 | 0.001 | 0.000 | 0.001 | 0.001 | 0.000 | 0.001 | 0.001 | 0.002 |
| meta1429 |  | 512.317 | 251.416 | 0.000 | 0.000 | 0.000 | 0.001 | 0.000 | 0.000 | 0.000 | 0.000 | 0.000 | 0.000 | 0.000 | 0.000 | 0.000 | 0.000 | 0.000 |
| meta1430 |  | 512.694 | 161.224 | 0.006 | 0.006 | 0.005 | 0.001 | 0.001 | 0.005 | 0.007 | 0.010 | 0.003 | 0.011 | 0.006 | 0.004 | 0.009 | 0.010 | 0.004 |
| meta1431 |  | 512.697 | 182.892 | 0.005 | 0.006 | 0.005 | 0.001 | 0.002 | 0.007 | 0.013 | 0.005 | 0.002 | 0.009 | 0.006 | 0.002 | 0.009 | 0.010 | 0.003 |
| meta1432 |  | 513.048 | 97.254 | 0.001 | 0.001 | 0.001 | 0.000 | 0.001 | 0.000 | 0.005 | 0.004 | 0.002 | 0.001 | 0.000 | 0.000 | 0.001 | 0.001 | 0.001 |
| meta1433 |  | 513.162 | 587.329 | 0.028 | 0.018 | 0.018 | 0.029 | 0.024 | 0.017 | 0.022 | 0.026 | 0.015 | 0.025 | 0.027 | 0.019 | 0.020 | 0.018 | 0.021 |
| meta1434 |  | 513.195 | 361.424 | 0.006 | 0.005 | 0.004 | 0.006 | 0.007 | 0.004 | 0.010 | 0.005 | 0.003 | 0.007 | 0.009 | 0.005 | 0.004 | 0.002 | 0.010 |
| meta1435 |  | 513.199 | 139.782 | 0.006 | 0.005 | 0.005 | 0.004 | 0.005 | 0.003 | 0.005 | 0.004 | 0.003 | 0.004 | 0.006 | 0.006 | 0.004 | 0.005 | 0.005 |
| meta1436 |  | 513.285 | 43.214 | 0.016 | 0.018 | 0.012 | 0.009 | 0.010 | 0.016 | 0.019 | 0.017 | 0.009 | 0.036 | 0.024 | 0.017 | 0.030 | 0.022 | 0.012 |
| meta1437 |  | 514.283 | 161.220 | 2.657 | 2.601 | 2.392 | 0.437 | 0.732 | 2.651 | 3.169 | 4.895 | 0.933 | 5.493 | 2.485 | 1.829 | 4.310 | 5.077 | 1.757 |
| meta1438 |  | 515.083 | 91.366 | 0.003 | 0.003 | 0.004 | 0.010 | 0.006 | 0.009 | 0.005 | 0.002 | 0.001 | 0.000 | 0.002 | 0.000 | 0.001 | 0.001 | 0.001 |
| meta1439 |  | 515.099 | 320.751 | 0.023 | 0.023 | 0.021 | 0.025 | 0.020 | 0.016 | 0.020 | 0.020 | 0.022 | 0.024 | 0.021 | 0.018 | 0.019 | 0.021 | 0.023 |
| meta1440 |  | 515.192 | 85.319 | 0.004 | 0.005 | 0.005 | 0.007 | 0.007 | 0.005 | 0.005 | 0.004 | 0.004 | 0.005 | 0.005 | 0.003 | 0.005 | 0.004 | 0.006 |
| meta1441 |  | 515.265 | 45.266 | 0.041 | 0.035 | 0.033 | 0.002 | 0.003 | 0.004 | 0.035 | 0.047 | 0.017 | 0.098 | 0.004 | 0.002 | 0.004 | 0.043 | 0.003 |
| meta1442 | Adynerin | 515.298 | 233.379 | 0.001 | 0.001 | 0.001 | 0.000 | 0.000 | 0.000 | 0.004 | 0.001 | 0.001 | 0.001 | 0.002 | 0.002 | 0.004 | 0.001 | 0.001 |
| meta1443 |  | 516.051 | 413.017 | 0.012 | 0.010 | 0.009 | 0.011 | 0.009 | 0.008 | 0.013 | 0.012 | 0.011 | 0.011 | 0.010 | 0.011 | 0.015 | 0.008 | 0.010 |
| meta1444 |  | 516.127 | 24.388 | 0.001 | 0.001 | 0.001 | 0.001 | 0.001 | 0.000 | 0.001 | 0.001 | 0.001 | 0.000 | 0.001 | 0.001 | 0.001 | 0.001 | 0.001 |
| meta1445 |  | 516.201 | 368.197 | 0.004 | 0.004 | 0.004 | 0.005 | 0.006 | 0.004 | 0.005 | 0.005 | 0.003 | 0.005 | 0.003 | 0.005 | 0.004 | 0.002 | 0.002 |
| meta1446 |  | 516.948 | 318.304 | 0.003 | 0.003 | 0.003 | 0.004 | 0.003 | 0.003 | 0.003 | 0.003 | 0.004 | 0.003 | 0.003 | 0.003 | 0.003 | 0.003 | 0.003 |
| meta1447 |  | 517.313 | 143.515 | 0.005 | 0.005 | 0.004 | 0.003 | 0.001 | 0.004 | 0.010 | 0.008 | 0.009 | 0.005 | 0.007 | 0.004 | 0.008 | 0.004 | 0.001 |
| meta1448 |  | 518.066 | 420.690 | 0.023 | 0.022 | 0.023 | 0.025 | 0.022 | 0.017 | 0.022 | 0.030 | 0.030 | 0.027 | 0.025 | 0.026 | 0.039 | 0.023 | 0.024 |
| meta1449 |  | 518.099 | 333.070 | 0.063 | 0.062 | 0.062 | 0.074 | 0.059 | 0.050 | 0.060 | 0.065 | 0.069 | 0.068 | 0.058 | 0.049 | 0.059 | 0.055 | 0.059 |
| meta1450 |  | 518.126 | 280.634 | 0.000 | 0.000 | 0.001 | 0.000 | 0.000 | 0.000 | 0.001 | 0.001 | 0.000 | 0.000 | 0.000 | 0.000 | 0.000 | 0.000 | 0.000 |
| meta1451 |  | 518.142 | 156.641 | 0.002 | 0.001 | 0.001 | 0.001 | 0.003 | 0.001 | 0.002 | 0.002 | 0.001 | 0.001 | 0.001 | 0.001 | 0.001 | 0.001 | 0.001 |
| meta1452 |  | 519.212 | 37.422 | 0.004 | 0.004 | 0.004 | 0.006 | 0.012 | 0.004 | 0.009 | 0.005 | 0.003 | 0.004 | 0.003 | 0.002 | 0.002 | 0.002 | 0.000 |
| meta1453 |  | 520.018 | 415.002 | 0.002 | 0.002 | 0.002 | 0.002 | 0.003 | 0.001 | 0.002 | 0.002 | 0.002 | 0.002 | 0.002 | 0.002 | 0.002 | 0.002 | 0.002 |
| meta1454 |  | 520.100 | 28.189 | 0.001 | 0.001 | 0.002 | 0.004 | 0.002 | 0.001 | 0.001 | 0.001 | 0.001 | 0.001 | 0.001 | 0.001 | 0.001 | 0.001 | 0.001 |
| meta1455 |  | 521.029 | 273.881 | 0.003 | 0.004 | 0.003 | 0.004 | 0.004 | 0.003 | 0.004 | 0.003 | 0.003 | 0.004 | 0.003 | 0.002 | 0.003 | 0.004 | 0.003 |
| meta1456 |  | 521.098 | 350.963 | 0.039 | 0.043 | 0.039 | 0.051 | 0.041 | 0.035 | 0.042 | 0.048 | 0.023 | 0.049 | 0.040 | 0.039 | 0.043 | 0.040 | 0.038 |
| meta1457 |  | 521.099 | 432.829 | 0.003 | 0.003 | 0.003 | 0.003 | 0.003 | 0.002 | 0.003 | 0.004 | 0.003 | 0.003 | 0.003 | 0.003 | 0.003 | 0.003 | 0.003 |
| meta1458 |  | 521.181 | 361.444 | 0.001 | 0.001 | 0.001 | 0.001 | 0.001 | 0.000 | 0.001 | 0.001 | 0.000 | 0.001 | 0.001 | 0.001 | 0.001 | 0.001 | 0.001 |
| meta1459 |  | 521.997 | 417.400 | 0.001 | 0.001 | 0.001 | 0.002 | 0.001 | 0.001 | 0.001 | 0.002 | 0.001 | 0.002 | 0.001 | 0.002 | 0.002 | 0.001 | 0.002 |
| meta1460 |  | 522.030 | 479.080 | 0.002 | 0.002 | 0.002 | 0.002 | 0.003 | 0.002 | 0.002 | 0.002 | 0.002 | 0.002 | 0.001 | 0.002 | 0.003 | 0.002 | 0.002 |
| meta1461 |  | 522.129 | 267.857 | 0.003 | 0.002 | 0.002 | 0.002 | 0.003 | 0.003 | 0.003 | 0.002 | 0.001 | 0.001 | 0.002 | 0.003 | 0.002 | 0.001 | 0.002 |
| meta1462 |  | 522.257 | 176.675 | 0.001 | 0.001 | 0.001 | 0.001 | 0.001 | 0.001 | 0.001 | 0.001 | 0.001 | 0.001 | 0.001 | 0.001 | 0.002 | 0.001 | 0.001 |
| meta1463 |  | 522.322 | 44.574 | 0.010 | 0.008 | 0.010 | 0.014 | 0.012 | 0.011 | 0.014 | 0.020 | 0.011 | 0.011 | 0.014 | 0.014 | 0.011 | 0.008 | 0.017 |
| meta1464 |  | 522.353 | 170.896 | 0.001 | 0.001 | 0.002 | 0.002 | 0.002 | 0.001 | 0.002 | 0.002 | 0.001 | 0.001 | 0.002 | 0.002 | 0.002 | 0.001 | 0.002 |
| meta1465 |  | 522.860 | 318.308 | 0.001 | 0.001 | 0.001 | 0.001 | 0.001 | 0.001 | 0.001 | 0.001 | 0.001 | 0.001 | 0.001 | 0.001 | 0.001 | 0.001 | 0.001 |
| meta1466 | Deoxyguanosine triphosphate (dGTP) | 523.025 | 273.810 | 0.001 | 0.001 | 0.001 | 0.001 | 0.000 | 0.000 | 0.001 | 0.001 | 0.001 | 0.001 | 0.001 | 0.000 | 0.000 | 0.001 | 0.000 |
| meta1467 |  | 523.104 | 150.806 | 0.001 | 0.001 | 0.001 | 0.001 | 0.001 | 0.001 | 0.001 | 0.001 | 0.001 | 0.000 | 0.001 | 0.001 | 0.001 | 0.001 | 0.001 |
| meta1468 |  | 523.279 | 45.946 | 0.012 | 0.006 | 0.008 | 0.016 | 0.010 | 0.009 | 0.011 | 0.013 | 0.003 | 0.015 | 0.012 | 0.022 | 0.008 | 0.010 | 0.018 |
| meta1469 |  | 524.135 | 365.800 | 0.022 | 0.021 | 0.018 | 0.025 | 0.026 | 0.019 | 0.020 | 0.021 | 0.011 | 0.022 | 0.028 | 0.024 | 0.015 | 0.013 | 0.012 |
| meta1470 |  | 524.296 | 222.114 | 0.009 | 0.010 | 0.009 | 0.009 | 0.010 | 0.007 | 0.011 | 0.014 | 0.007 | 0.009 | 0.010 | 0.010 | 0.008 | 0.007 | 0.010 |
| meta1471 |  | 525.003 | 268.566 | 0.001 | 0.001 | 0.001 | 0.002 | 0.002 | 0.001 | 0.003 | 0.002 | 0.001 | 0.001 | 0.001 | 0.001 | 0.001 | 0.001 | 0.001 |
| meta1472 |  | 525.094 | 471.443 | 0.001 | 0.001 | 0.001 | 0.001 | 0.001 | 0.001 | 0.001 | 0.001 | 0.001 | 0.002 | 0.001 | 0.001 | 0.001 | 0.001 | 0.001 |
| meta1473 |  | 525.362 | 164.313 | 0.003 | 0.003 | 0.003 | 0.010 | 0.001 | 0.009 | 0.003 | 0.001 | 0.001 | 0.001 | 0.003 | 0.002 | 0.002 | 0.002 | 0.002 |
| meta1474 |  | 525.381 | 47.011 | 0.000 | 0.000 | 0.000 | 0.001 | 0.000 | 0.000 | 0.000 | 0.000 | 0.000 | 0.000 | 0.000 | 0.000 | 0.000 | 0.000 | 0.000 |
| meta1475 |  | 526.052 | 420.597 | 0.001 | 0.001 | 0.001 | 0.001 | 0.001 | 0.000 | 0.001 | 0.001 | 0.001 | 0.001 | 0.001 | 0.001 | 0.001 | 0.001 | 0.001 |
| meta1476 |  | 526.149 | 97.575 | 0.001 | 0.001 | 0.001 | 0.000 | 0.001 | 0.000 | 0.002 | 0.002 | 0.002 | 0.001 | 0.001 | 0.001 | 0.001 | 0.000 | 0.000 |
| meta1477 |  | 526.199 | 40.076 | 0.005 | 0.006 | 0.007 | 0.006 | 0.006 | 0.008 | 0.007 | 0.005 | 0.005 | 0.006 | 0.007 | 0.006 | 0.007 | 0.009 | 0.005 |
| meta1478 |  | 526.311 | 178.692 | 0.002 | 0.002 | 0.002 | 0.003 | 0.003 | 0.002 | 0.004 | 0.003 | 0.001 | 0.002 | 0.002 | 0.002 | 0.002 | 0.002 | 0.003 |
| meta1479 |  | 527.054 | 154.670 | 0.000 | 0.000 | 0.000 | 0.001 | 0.001 | 0.000 | 0.001 | 0.000 | 0.000 | 0.000 | 0.000 | 0.000 | 0.000 | 0.000 | 0.000 |
| meta1480 |  | 527.118 | 239.592 | 0.000 | 0.000 | 0.000 | 0.000 | 0.000 | 0.000 | 0.000 | 0.000 | 0.000 | 0.000 | 0.000 | 0.000 | 0.000 | 0.001 | 0.000 |
| meta1481 |  | 527.283 | 24.744 | 0.002 | 0.002 | 0.002 | 0.002 | 0.003 | 0.002 | 0.003 | 0.003 | 0.002 | 0.001 | 0.003 | 0.003 | 0.002 | 0.003 | 0.003 |
| meta1482 |  | 527.338 | 34.301 | 0.001 | 0.001 | 0.001 | 0.002 | 0.001 | 0.001 | 0.001 | 0.001 | 0.002 | 0.001 | 0.001 | 0.002 | 0.003 | 0.001 | 0.002 |
| meta1483 |  | 528.201 | 350.914 | 0.002 | 0.002 | 0.002 | 0.002 | 0.002 | 0.001 | 0.002 | 0.003 | 0.002 | 0.002 | 0.001 | 0.002 | 0.002 | 0.001 | 0.002 |
| meta1484 |  | 529.042 | 364.831 | 0.006 | 0.007 | 0.006 | 0.007 | 0.006 | 0.005 | 0.007 | 0.007 | 0.008 | 0.006 | 0.006 | 0.005 | 0.007 | 0.006 | 0.007 |
| meta1485 |  | 529.042 | 381.303 | 0.005 | 0.005 | 0.007 | 0.006 | 0.008 | 0.005 | 0.008 | 0.007 | 0.007 | 0.007 | 0.005 | 0.005 | 0.008 | 0.005 | 0.005 |
| meta1486 |  | 529.155 | 427.491 | 0.001 | 0.001 | 0.001 | 0.001 | 0.001 | 0.001 | 0.001 | 0.001 | 0.001 | 0.001 | 0.001 | 0.001 | 0.001 | 0.001 | 0.000 |
| meta1487 |  | 529.208 | 85.316 | 0.003 | 0.003 | 0.003 | 0.004 | 0.003 | 0.003 | 0.003 | 0.003 | 0.002 | 0.003 | 0.003 | 0.002 | 0.003 | 0.002 | 0.003 |
| meta1488 |  | 529.222 | 363.374 | 0.004 | 0.003 | 0.003 | 0.004 | 0.004 | 0.004 | 0.003 | 0.005 | 0.003 | 0.005 | 0.004 | 0.005 | 0.003 | 0.003 | 0.001 |
| meta1489 |  | 529.254 | 129.438 | 0.002 | 0.002 | 0.003 | 0.004 | 0.004 | 0.002 | 0.003 | 0.003 | 0.002 | 0.004 | 0.003 | 0.002 | 0.003 | 0.003 | 0.003 |
| meta1490 |  | 529.281 | 44.571 | 0.023 | 0.019 | 0.016 | 0.011 | 0.010 | 0.036 | 0.029 | 0.026 | 0.009 | 0.046 | 0.030 | 0.015 | 0.044 | 0.022 | 0.012 |
| meta1491 |  | 530.170 | 374.106 | 0.001 | 0.001 | 0.001 | 0.001 | 0.002 | 0.001 | 0.001 | 0.002 | 0.000 | 0.001 | 0.001 | 0.001 | 0.001 | 0.001 | 0.001 |
| meta1492 |  | 530.276 | 190.707 | 0.015 | 0.015 | 0.014 | 0.006 | 0.006 | 0.029 | 0.012 | 0.033 | 0.003 | 0.014 | 0.022 | 0.024 | 0.024 | 0.015 | 0.024 |
| meta1493 |  | 530.276 | 221.666 | 0.002 | 0.002 | 0.003 | 0.002 | 0.003 | 0.004 | 0.004 | 0.004 | 0.000 | 0.002 | 0.002 | 0.001 | 0.003 | 0.004 | 0.002 |
| meta1494 |  | 530.298 | 174.202 | 0.011 | 0.011 | 0.012 | 0.014 | 0.013 | 0.011 | 0.018 | 0.010 | 0.010 | 0.011 | 0.011 | 0.011 | 0.012 | 0.015 | 0.012 |
| meta1495 |  | 531.092 | 150.812 | 0.007 | 0.006 | 0.007 | 0.008 | 0.010 | 0.006 | 0.010 | 0.007 | 0.004 | 0.003 | 0.004 | 0.006 | 0.005 | 0.006 | 0.005 |
| meta1496 |  | 531.297 | 102.379 | 0.050 | 0.051 | 0.051 | 0.035 | 0.024 | 0.211 | 0.146 | 0.058 | 0.016 | 0.145 | 0.075 | 0.024 | 0.158 | 0.079 | 0.061 |
| meta1497 |  | 531.329 | 40.083 | 0.013 | 0.008 | 0.012 | 0.021 | 0.026 | 0.045 | 0.035 | 0.050 | 0.005 | 0.045 | 0.034 | 0.035 | 0.043 | 0.034 | 0.027 |
| meta1498 | 1,3-Dicaffeoylquinic acid | 532.149 | 398.448 | 0.001 | 0.001 | 0.002 | 0.002 | 0.002 | 0.002 | 0.001 | 0.001 | 0.001 | 0.002 | 0.001 | 0.001 | 0.001 | 0.001 | 0.001 |
| meta1499 |  | 532.347 | 95.138 | 0.004 | 0.003 | 0.003 | 0.004 | 0.005 | 0.003 | 0.004 | 0.003 | 0.001 | 0.002 | 0.003 | 0.003 | 0.002 | 0.003 | 0.002 |
| meta1500 |  | 532.767 | 288.272 | 0.004 | 0.003 | 0.003 | 0.004 | 0.004 | 0.004 | 0.004 | 0.003 | 0.005 | 0.005 | 0.004 | 0.004 | 0.005 | 0.004 | 0.004 |
| meta1501 |  | 532.979 | 318.388 | 0.002 | 0.002 | 0.002 | 0.002 | 0.002 | 0.002 | 0.002 | 0.002 | 0.002 | 0.002 | 0.002 | 0.002 | 0.002 | 0.002 | 0.002 |
| meta1502 |  | 533.088 | 158.645 | 0.002 | 0.002 | 0.002 | 0.003 | 0.002 | 0.003 | 0.002 | 0.001 | 0.002 | 0.001 | 0.002 | 0.003 | 0.002 | 0.001 | 0.001 |
| meta1503 |  | 533.110 | 346.994 | 0.010 | 0.008 | 0.009 | 0.009 | 0.007 | 0.006 | 0.008 | 0.010 | 0.011 | 0.011 | 0.007 | 0.007 | 0.008 | 0.006 | 0.009 |
| meta1504 |  | 533.133 | 368.176 | 0.012 | 0.011 | 0.011 | 0.013 | 0.014 | 0.014 | 0.010 | 0.011 | 0.009 | 0.015 | 0.010 | 0.013 | 0.012 | 0.010 | 0.006 |
| meta1505 |  | 533.170 | 483.730 | 0.001 | 0.001 | 0.001 | 0.001 | 0.001 | 0.001 | 0.001 | 0.001 | 0.001 | 0.001 | 0.001 | 0.002 | 0.002 | 0.001 | 0.001 |
| meta1506 |  | 533.453 | 42.645 | 0.015 | 0.018 | 0.018 | 0.020 | 0.015 | 0.021 | 0.016 | 0.025 | 0.021 | 0.016 | 0.016 | 0.023 | 0.018 | 0.021 | 0.016 |
| meta1507 |  | 534.094 | 407.900 | 0.031 | 0.029 | 0.029 | 0.032 | 0.027 | 0.024 | 0.028 | 0.036 | 0.036 | 0.041 | 0.029 | 0.026 | 0.031 | 0.022 | 0.028 |
| meta1508 |  | 534.116 | 27.568 | 0.006 | 0.007 | 0.006 | 0.013 | 0.009 | 0.006 | 0.005 | 0.004 | 0.004 | 0.004 | 0.004 | 0.004 | 0.005 | 0.004 | 0.004 |
| meta1509 |  | 534.128 | 339.574 | 0.003 | 0.003 | 0.003 | 0.004 | 0.003 | 0.003 | 0.003 | 0.003 | 0.002 | 0.003 | 0.004 | 0.004 | 0.002 | 0.002 | 0.002 |
| meta1510 |  | 534.247 | 124.971 | 0.003 | 0.002 | 0.002 | 0.001 | 0.002 | 0.005 | 0.007 | 0.005 | 0.001 | 0.009 | 0.002 | 0.003 | 0.012 | 0.009 | 0.002 |
| meta1511 |  | 534.277 | 180.548 | 0.001 | 0.001 | 0.001 | 0.002 | 0.001 | 0.000 | 0.001 | 0.001 | 0.001 | 0.001 | 0.001 | 0.001 | 0.001 | 0.001 | 0.001 |
| meta1512 |  | 534.304 | 38.138 | 0.008 | 0.008 | 0.008 | 0.007 | 0.007 | 0.006 | 0.018 | 0.006 | 0.005 | 0.010 | 0.006 | 0.018 | 0.008 | 0.009 | 0.009 |
| meta1513 |  | 534.984 | 284.770 | 0.001 | 0.001 | 0.001 | 0.001 | 0.001 | 0.001 | 0.001 | 0.001 | 0.001 | 0.001 | 0.001 | 0.000 | 0.001 | 0.001 | 0.001 |
| meta1514 |  | 535.030 | 97.423 | 0.002 | 0.002 | 0.003 | 0.000 | 0.000 | 0.000 | 0.019 | 0.011 | 0.007 | 0.003 | 0.001 | 0.000 | 0.004 | 0.000 | 0.001 |
| meta1515 |  | 535.033 | 425.127 | 0.002 | 0.002 | 0.002 | 0.002 | 0.002 | 0.002 | 0.002 | 0.002 | 0.002 | 0.003 | 0.002 | 0.002 | 0.002 | 0.002 | 0.002 |
| meta1516 |  | 535.148 | 370.473 | 0.011 | 0.010 | 0.009 | 0.010 | 0.011 | 0.010 | 0.008 | 0.014 | 0.009 | 0.011 | 0.012 | 0.015 | 0.011 | 0.009 | 0.005 |
| meta1517 |  | 535.151 | 200.068 | 0.062 | 0.059 | 0.054 | 0.066 | 0.102 | 0.064 | 0.095 | 0.043 | 0.025 | 0.032 | 0.048 | 0.068 | 0.049 | 0.064 | 0.042 |
| meta1518 |  | 535.397 | 132.974 | 0.001 | 0.002 | 0.002 | 0.004 | 0.003 | 0.002 | 0.002 | 0.002 | 0.002 | 0.002 | 0.002 | 0.002 | 0.002 | 0.001 | 0.002 |
| meta1519 |  | 536.263 | 182.987 | 0.010 | 0.010 | 0.009 | 0.004 | 0.005 | 0.010 | 0.011 | 0.009 | 0.005 | 0.011 | 0.009 | 0.005 | 0.013 | 0.011 | 0.007 |
| meta1520 |  | 536.302 | 45.359 | 0.004 | 0.004 | 0.004 | 0.004 | 0.004 | 0.005 | 0.005 | 0.007 | 0.004 | 0.007 | 0.004 | 0.003 | 0.004 | 0.005 | 0.004 |
| meta1521 |  | 536.501 | 33.500 | 0.006 | 0.005 | 0.006 | 0.001 | 0.001 | 0.001 | 0.001 | 0.002 | 0.001 | 0.001 | 0.004 | 0.004 | 0.002 | 0.000 | 0.001 |
| meta1522 |  | 536.728 | 289.082 | 0.002 | 0.002 | 0.003 | 0.003 | 0.002 | 0.002 | 0.002 | 0.002 | 0.003 | 0.002 | 0.002 | 0.001 | 0.003 | 0.003 | 0.002 |
| meta1523 |  | 537.041 | 482.210 | 0.000 | 0.000 | 0.000 | 0.000 | 0.000 | 0.000 | 0.000 | 0.000 | 0.000 | 0.000 | 0.000 | 0.000 | 0.001 | 0.000 | 0.000 |
| meta1524 | Amentoflavone | 537.094 | 421.340 | 0.070 | 0.067 | 0.062 | 0.082 | 0.058 | 0.045 | 0.060 | 0.073 | 0.068 | 0.069 | 0.067 | 0.055 | 0.055 | 0.052 | 0.062 |
| meta1525 |  | 537.164 | 367.237 | 0.008 | 0.007 | 0.008 | 0.010 | 0.010 | 0.010 | 0.008 | 0.010 | 0.006 | 0.008 | 0.008 | 0.010 | 0.009 | 0.005 | 0.004 |
| meta1526 |  | 537.328 | 174.250 | 0.022 | 0.022 | 0.022 | 0.033 | 0.029 | 0.023 | 0.039 | 0.027 | 0.019 | 0.019 | 0.024 | 0.024 | 0.023 | 0.025 | 0.025 |
| meta1527 |  | 537.999 | 334.685 | 0.002 | 0.001 | 0.001 | 0.002 | 0.002 | 0.001 | 0.001 | 0.002 | 0.001 | 0.002 | 0.001 | 0.001 | 0.001 | 0.001 | 0.001 |
| meta1528 |  | 539.135 | 428.124 | 0.002 | 0.002 | 0.002 | 0.003 | 0.002 | 0.003 | 0.002 | 0.002 | 0.001 | 0.002 | 0.002 | 0.003 | 0.001 | 0.001 | 0.001 |
| meta1529 |  | 539.149 | 333.988 | 0.002 | 0.002 | 0.001 | 0.002 | 0.002 | 0.001 | 0.001 | 0.002 | 0.001 | 0.002 | 0.001 | 0.001 | 0.002 | 0.001 | 0.002 |
| meta1530 |  | 539.284 | 47.472 | 0.006 | 0.006 | 0.008 | 0.002 | 0.003 | 0.005 | 0.021 | 0.012 | 0.011 | 0.006 | 0.005 | 0.015 | 0.013 | 0.006 | 0.006 |
| meta1531 | Cyclic adenosine diphosphate ribose | 540.050 | 421.340 | 0.012 | 0.011 | 0.012 | 0.012 | 0.011 | 0.008 | 0.012 | 0.017 | 0.016 | 0.012 | 0.010 | 0.012 | 0.021 | 0.010 | 0.015 |
| meta1532 |  | 540.114 | 269.060 | 0.000 | 0.000 | 0.000 | 0.000 | 0.000 | 0.000 | 0.000 | 0.001 | 0.000 | 0.000 | 0.000 | 0.000 | 0.000 | 0.000 | 0.000 |
| meta1533 |  | 540.278 | 161.222 | 0.002 | 0.002 | 0.002 | 0.000 | 0.001 | 0.001 | 0.002 | 0.003 | 0.001 | 0.002 | 0.001 | 0.001 | 0.002 | 0.003 | 0.001 |
| meta1534 |  | 540.364 | 177.996 | 0.005 | 0.005 | 0.005 | 0.007 | 0.006 | 0.005 | 0.006 | 0.005 | 0.003 | 0.004 | 0.006 | 0.005 | 0.006 | 0.006 | 0.006 |
| meta1535 |  | 540.909 | 318.111 | 0.002 | 0.002 | 0.002 | 0.003 | 0.002 | 0.002 | 0.002 | 0.002 | 0.002 | 0.002 | 0.002 | 0.002 | 0.003 | 0.003 | 0.002 |
| meta1536 |  | 541.169 | 157.275 | 0.006 | 0.004 | 0.005 | 0.004 | 0.011 | 0.003 | 0.006 | 0.007 | 0.004 | 0.005 | 0.005 | 0.005 | 0.005 | 0.003 | 0.006 |
| meta1537 |  | 542.011 | 420.753 | 0.001 | 0.001 | 0.001 | 0.001 | 0.001 | 0.000 | 0.001 | 0.001 | 0.001 | 0.001 | 0.001 | 0.001 | 0.001 | 0.001 | 0.001 |
| meta1538 |  | 542.146 | 365.800 | 0.004 | 0.004 | 0.003 | 0.005 | 0.005 | 0.004 | 0.004 | 0.004 | 0.002 | 0.004 | 0.006 | 0.004 | 0.003 | 0.003 | 0.002 |
| meta1539 |  | 543.061 | 401.415 | 0.001 | 0.001 | 0.001 | 0.001 | 0.001 | 0.001 | 0.002 | 0.001 | 0.001 | 0.001 | 0.001 | 0.001 | 0.002 | 0.001 | 0.001 |
| meta1540 |  | 543.137 | 200.069 | 0.006 | 0.006 | 0.005 | 0.006 | 0.007 | 0.005 | 0.009 | 0.004 | 0.003 | 0.004 | 0.005 | 0.007 | 0.005 | 0.006 | 0.005 |
| meta1541 |  | 543.991 | 25.492 | 0.006 | 0.006 | 0.007 | 0.007 | 0.004 | 0.006 | 0.007 | 0.010 | 0.012 | 0.010 | 0.006 | 0.003 | 0.004 | 0.003 | 0.003 |
| meta1542 |  | 544.047 | 381.211 | 0.001 | 0.001 | 0.001 | 0.001 | 0.001 | 0.000 | 0.001 | 0.001 | 0.001 | 0.001 | 0.001 | 0.001 | 0.001 | 0.001 | 0.000 |
| meta1543 |  | 545.168 | 468.730 | 0.001 | 0.001 | 0.001 | 0.001 | 0.001 | 0.002 | 0.001 | 0.001 | 0.001 | 0.001 | 0.001 | 0.001 | 0.001 | 0.001 | 0.001 |
| meta1544 |  | 545.165 | 94.953 | 0.007 | 0.006 | 0.006 | 0.010 | 0.011 | 0.008 | 0.011 | 0.007 | 0.003 | 0.004 | 0.008 | 0.008 | 0.006 | 0.008 | 0.016 |
| meta1545 |  | 545.305 | 45.144 | 0.008 | 0.007 | 0.010 | 0.003 | 0.007 | 0.004 | 0.010 | 0.020 | 0.006 | 0.007 | 0.018 | 0.013 | 0.011 | 0.010 | 0.015 |
| meta1546 |  | 546.820 | 287.380 | 0.004 | 0.003 | 0.003 | 0.005 | 0.005 | 0.004 | 0.005 | 0.005 | 0.005 | 0.004 | 0.004 | 0.004 | 0.006 | 0.005 | 0.004 |
| meta1547 |  | 546.959 | 85.602 | 0.002 | 0.002 | 0.002 | 0.002 | 0.003 | 0.002 | 0.002 | 0.002 | 0.002 | 0.002 | 0.002 | 0.001 | 0.002 | 0.002 | 0.003 |
| meta1548 |  | 547.062 | 150.812 | 0.003 | 0.003 | 0.003 | 0.003 | 0.004 | 0.002 | 0.004 | 0.002 | 0.002 | 0.001 | 0.002 | 0.003 | 0.002 | 0.002 | 0.003 |
| meta1549 |  | 547.166 | 278.951 | 0.099 | 0.092 | 0.098 | 0.119 | 0.096 | 0.083 | 0.109 | 0.099 | 0.075 | 0.079 | 0.084 | 0.093 | 0.109 | 0.092 | 0.090 |
| meta1550 |  | 547.324 | 46.649 | 0.037 | 0.037 | 0.040 | 0.057 | 0.044 | 0.039 | 0.055 | 0.056 | 0.016 | 0.035 | 0.039 | 0.096 | 0.045 | 0.050 | 0.085 |
| meta1551 |  | 547.995 | 473.996 | 0.002 | 0.002 | 0.002 | 0.003 | 0.002 | 0.002 | 0.002 | 0.002 | 0.002 | 0.002 | 0.002 | 0.003 | 0.003 | 0.002 | 0.002 |
| meta1552 |  | 548.110 | 420.073 | 0.005 | 0.005 | 0.005 | 0.006 | 0.005 | 0.004 | 0.004 | 0.006 | 0.005 | 0.006 | 0.005 | 0.005 | 0.005 | 0.004 | 0.005 |
| meta1553 |  | 549.171 | 279.334 | 0.006 | 0.004 | 0.006 | 0.007 | 0.006 | 0.005 | 0.006 | 0.006 | 0.004 | 0.005 | 0.005 | 0.005 | 0.006 | 0.005 | 0.005 |
| meta1554 | Manumycin A | 549.267 | 245.072 | 0.000 | 0.000 | 0.000 | 0.001 | 0.000 | 0.001 | 0.000 | 0.000 | 0.000 | 0.000 | 0.000 | 0.000 | 0.000 | 0.000 | 0.000 |
| meta1555 |  | 550.179 | 371.560 | 0.001 | 0.000 | 0.000 | 0.001 | 0.000 | 0.000 | 0.001 | 0.000 | 0.000 | 0.001 | 0.001 | 0.000 | 0.000 | 0.000 | 0.001 |
| meta1556 |  | 550.199 | 40.726 | 0.014 | 0.015 | 0.016 | 0.016 | 0.014 | 0.017 | 0.017 | 0.014 | 0.014 | 0.016 | 0.013 | 0.018 | 0.014 | 0.023 | 0.014 |
| meta1557 |  | 550.298 | 46.024 | 0.007 | 0.006 | 0.007 | 0.010 | 0.008 | 0.009 | 0.009 | 0.008 | 0.003 | 0.007 | 0.007 | 0.013 | 0.008 | 0.010 | 0.014 |
| meta1558 |  | 551.143 | 368.533 | 0.004 | 0.003 | 0.003 | 0.004 | 0.004 | 0.005 | 0.003 | 0.004 | 0.003 | 0.004 | 0.003 | 0.004 | 0.004 | 0.003 | 0.002 |
| meta1559 |  | 551.250 | 158.629 | 0.001 | 0.001 | 0.001 | 0.001 | 0.001 | 0.001 | 0.001 | 0.001 | 0.000 | 0.001 | 0.001 | 0.001 | 0.001 | 0.001 | 0.001 |
| meta1560 |  | 552.104 | 426.849 | 0.007 | 0.006 | 0.006 | 0.008 | 0.005 | 0.004 | 0.006 | 0.007 | 0.007 | 0.007 | 0.006 | 0.005 | 0.005 | 0.005 | 0.007 |
| meta1561 | Taurocholate | 552.232 | 182.625 | 0.002 | 0.002 | 0.002 | 0.001 | 0.002 | 0.001 | 0.002 | 0.002 | 0.001 | 0.003 | 0.002 | 0.002 | 0.002 | 0.002 | 0.002 |
| meta1562 |  | 552.291 | 216.325 | 0.000 | 0.001 | 0.001 | 0.001 | 0.001 | 0.001 | 0.000 | 0.000 | 0.001 | 0.000 | 0.000 | 0.000 | 0.000 | 0.001 | 0.000 |
| meta1563 |  | 552.307 | 163.105 | 0.002 | 0.003 | 0.002 | 0.007 | 0.007 | 0.003 | 0.006 | 0.003 | 0.003 | 0.002 | 0.003 | 0.004 | 0.004 | 0.003 | 0.003 |
| meta1564 |  | 552.328 | 175.426 | 0.016 | 0.016 | 0.019 | 0.023 | 0.029 | 0.018 | 0.034 | 0.025 | 0.012 | 0.019 | 0.014 | 0.017 | 0.016 | 0.023 | 0.021 |
| meta1565 |  | 552.632 | 174.287 | 0.002 | 0.002 | 0.002 | 0.003 | 0.003 | 0.002 | 0.004 | 0.003 | 0.002 | 0.002 | 0.002 | 0.002 | 0.002 | 0.002 | 0.003 |
| meta1566 |  | 552.816 | 577.181 | 0.003 | 0.002 | 0.002 | 0.003 | 0.003 | 0.002 | 0.002 | 0.003 | 0.002 | 0.002 | 0.002 | 0.002 | 0.002 | 0.002 | 0.002 |
| meta1567 |  | 552.857 | 286.138 | 0.001 | 0.001 | 0.001 | 0.001 | 0.001 | 0.001 | 0.001 | 0.001 | 0.001 | 0.001 | 0.001 | 0.001 | 0.001 | 0.001 | 0.000 |
| meta1568 |  | 553.075 | 150.853 | 0.001 | 0.001 | 0.001 | 0.001 | 0.002 | 0.001 | 0.001 | 0.001 | 0.001 | 0.001 | 0.001 | 0.001 | 0.001 | 0.001 | 0.001 |
| meta1569 |  | 553.089 | 468.266 | 0.013 | 0.011 | 0.013 | 0.015 | 0.014 | 0.015 | 0.013 | 0.018 | 0.019 | 0.020 | 0.010 | 0.016 | 0.020 | 0.012 | 0.012 |
| meta1570 |  | 553.099 | 218.643 | 0.001 | 0.001 | 0.001 | 0.001 | 0.000 | 0.001 | 0.002 | 0.001 | 0.001 | 0.001 | 0.001 | 0.001 | 0.001 | 0.001 | 0.001 |
| meta1571 |  | 553.151 | 94.076 | 0.001 | 0.001 | 0.001 | 0.001 | 0.001 | 0.001 | 0.001 | 0.000 | 0.000 | 0.000 | 0.001 | 0.001 | 0.001 | 0.001 | 0.001 |
| meta1572 |  | 554.120 | 477.429 | 0.002 | 0.002 | 0.002 | 0.003 | 0.002 | 0.002 | 0.002 | 0.003 | 0.003 | 0.003 | 0.002 | 0.002 | 0.003 | 0.002 | 0.002 |
| meta1573 |  | 554.133 | 423.554 | 0.001 | 0.001 | 0.001 | 0.001 | 0.001 | 0.000 | 0.001 | 0.001 | 0.001 | 0.001 | 0.001 | 0.001 | 0.001 | 0.000 | 0.001 |
| meta1574 |  | 554.345 | 174.202 | 0.976 | 0.944 | 1.022 | 1.461 | 1.290 | 0.996 | 1.748 | 1.222 | 0.802 | 0.884 | 1.030 | 1.087 | 0.933 | 1.070 | 1.104 |
| meta1575 |  | 555.069 | 284.491 | 0.004 | 0.003 | 0.002 | 0.004 | 0.004 | 0.003 | 0.004 | 0.004 | 0.002 | 0.002 | 0.003 | 0.003 | 0.003 | 0.002 | 0.002 |
| meta1576 |  | 555.152 | 276.243 | 0.007 | 0.006 | 0.007 | 0.007 | 0.007 | 0.005 | 0.007 | 0.006 | 0.006 | 0.007 | 0.006 | 0.007 | 0.007 | 0.007 | 0.007 |
| meta1577 |  | 555.270 | 126.595 | 0.083 | 0.053 | 0.064 | 0.136 | 0.129 | 0.069 | 0.133 | 0.044 | 0.044 | 0.100 | 0.141 | 0.080 | 0.064 | 0.056 | 0.109 |
| meta1578 |  | 556.046 | 421.899 | 0.001 | 0.001 | 0.001 | 0.001 | 0.001 | 0.001 | 0.001 | 0.001 | 0.001 | 0.001 | 0.001 | 0.001 | 0.002 | 0.001 | 0.001 |
| meta1579 |  | 556.163 | 371.048 | 0.137 | 0.115 | 0.094 | 0.138 | 0.139 | 0.099 | 0.125 | 0.137 | 0.062 | 0.138 | 0.171 | 0.131 | 0.091 | 0.080 | 0.074 |
| meta1580 |  | 556.941 | 318.683 | 0.002 | 0.001 | 0.002 | 0.002 | 0.002 | 0.002 | 0.002 | 0.001 | 0.002 | 0.002 | 0.002 | 0.001 | 0.002 | 0.002 | 0.002 |
| meta1581 |  | 557.120 | 368.450 | 0.001 | 0.001 | 0.001 | 0.001 | 0.002 | 0.000 | 0.001 | 0.000 | 0.001 | 0.001 | 0.000 | 0.001 | 0.001 | 0.001 | 0.000 |
| meta1582 |  | 557.133 | 200.241 | 0.019 | 0.018 | 0.018 | 0.024 | 0.025 | 0.018 | 0.025 | 0.017 | 0.012 | 0.013 | 0.017 | 0.019 | 0.018 | 0.018 | 0.015 |
| meta1583 |  | 557.454 | 42.011 | 0.016 | 0.018 | 0.020 | 0.023 | 0.013 | 0.018 | 0.012 | 0.017 | 0.024 | 0.010 | 0.018 | 0.029 | 0.019 | 0.025 | 0.043 |
| meta1584 | ADP-ribose | 558.062 | 410.049 | 0.007 | 0.006 | 0.006 | 0.006 | 0.005 | 0.005 | 0.007 | 0.007 | 0.009 | 0.007 | 0.006 | 0.007 | 0.008 | 0.005 | 0.006 |
| meta1585 |  | 558.288 | 182.582 | 0.003 | 0.003 | 0.003 | 0.001 | 0.001 | 0.006 | 0.005 | 0.003 | 0.001 | 0.005 | 0.004 | 0.001 | 0.005 | 0.004 | 0.002 |
| meta1586 |  | 559.063 | 318.773 | 0.000 | 0.000 | 0.000 | 0.000 | 0.000 | 0.000 | 0.000 | 0.000 | 0.001 | 0.000 | 0.000 | 0.000 | 0.000 | 0.000 | 0.000 |
| meta1587 |  | 559.313 | 46.325 | 0.003 | 0.002 | 0.002 | 0.004 | 0.002 | 0.003 | 0.002 | 0.006 | 0.003 | 0.003 | 0.005 | 0.003 | 0.002 | 0.002 | 0.005 |
| meta1588 |  | 560.077 | 412.515 | 0.015 | 0.012 | 0.012 | 0.012 | 0.017 | 0.009 | 0.011 | 0.016 | 0.012 | 0.016 | 0.020 | 0.020 | 0.011 | 0.012 | 0.010 |
| meta1589 |  | 560.119 | 382.524 | 0.001 | 0.001 | 0.001 | 0.001 | 0.002 | 0.000 | 0.002 | 0.001 | 0.001 | 0.001 | 0.000 | 0.001 | 0.001 | 0.001 | 0.000 |
| meta1590 |  | 560.273 | 189.327 | 0.000 | 0.000 | 0.000 | 0.000 | 0.000 | 0.000 | 0.001 | 0.001 | 0.000 | 0.001 | 0.001 | 0.001 | 0.001 | 0.001 | 0.000 |
| meta1591 |  | 560.293 | 178.623 | 0.001 | 0.001 | 0.001 | 0.001 | 0.001 | 0.001 | 0.001 | 0.001 | 0.001 | 0.001 | 0.001 | 0.001 | 0.001 | 0.001 | 0.001 |
| meta1592 |  | 560.432 | 32.217 | 0.001 | 0.001 | 0.001 | 0.000 | 0.000 | 0.000 | 0.001 | 0.001 | 0.001 | 0.002 | 0.001 | 0.005 | 0.001 | 0.001 | 0.000 |
| meta1593 |  | 561.015 | 96.887 | 0.002 | 0.002 | 0.002 | 0.000 | 0.000 | 0.000 | 0.010 | 0.003 | 0.004 | 0.002 | 0.001 | 0.000 | 0.003 | 0.000 | 0.002 |
| meta1594 | Protoporphyrin IX | 561.248 | 157.929 | 0.001 | 0.001 | 0.001 | 0.002 | 0.002 | 0.001 | 0.002 | 0.001 | 0.001 | 0.001 | 0.001 | 0.001 | 0.001 | 0.001 | 0.001 |
| meta1595 |  | 561.381 | 419.319 | 0.001 | 0.001 | 0.001 | 0.001 | 0.001 | 0.001 | 0.001 | 0.001 | 0.001 | 0.001 | 0.001 | 0.001 | 0.001 | 0.000 | 0.001 |
| meta1596 |  | 562.088 | 388.989 | 0.018 | 0.015 | 0.017 | 0.018 | 0.015 | 0.014 | 0.015 | 0.019 | 0.020 | 0.019 | 0.016 | 0.016 | 0.014 | 0.014 | 0.015 |
| meta1597 |  | 562.146 | 26.917 | 0.005 | 0.005 | 0.006 | 0.012 | 0.007 | 0.005 | 0.004 | 0.005 | 0.004 | 0.005 | 0.004 | 0.004 | 0.006 | 0.005 | 0.005 |
| meta1598 |  | 563.029 | 400.788 | 0.003 | 0.002 | 0.002 | 0.002 | 0.002 | 0.002 | 0.002 | 0.003 | 0.002 | 0.005 | 0.004 | 0.003 | 0.003 | 0.003 | 0.003 |
| meta1599 |  | 563.122 | 419.266 | 0.328 | 0.284 | 0.279 | 0.348 | 0.286 | 0.238 | 0.284 | 0.352 | 0.324 | 0.368 | 0.315 | 0.281 | 0.297 | 0.238 | 0.277 |
| meta1600 | Maltotriose | 563.179 | 428.763 | 0.016 | 0.014 | 0.016 | 0.016 | 0.013 | 0.023 | 0.017 | 0.017 | 0.010 | 0.013 | 0.015 | 0.022 | 0.015 | 0.011 | 0.008 |
| meta1601 |  | 563.319 | 49.416 | 0.003 | 0.003 | 0.004 | 0.006 | 0.003 | 0.004 | 0.021 | 0.005 | 0.012 | 0.003 | 0.002 | 0.007 | 0.003 | 0.004 | 0.003 |
| meta1602 |  | 563.970 | 416.813 | 0.001 | 0.001 | 0.001 | 0.001 | 0.000 | 0.001 | 0.001 | 0.001 | 0.001 | 0.001 | 0.001 | 0.001 | 0.001 | 0.000 | 0.000 |
| meta1603 |  | 564.138 | 378.304 | 0.004 | 0.004 | 0.004 | 0.005 | 0.004 | 0.003 | 0.003 | 0.004 | 0.003 | 0.004 | 0.005 | 0.005 | 0.003 | 0.003 | 0.003 |
| meta1604 |  | 564.139 | 344.527 | 0.099 | 0.072 | 0.076 | 0.085 | 0.082 | 0.108 | 0.075 | 0.090 | 0.052 | 0.074 | 0.089 | 0.116 | 0.063 | 0.059 | 0.044 |
| meta1605 |  | 564.258 | 137.330 | 0.003 | 0.002 | 0.002 | 0.001 | 0.001 | 0.003 | 0.005 | 0.003 | 0.001 | 0.006 | 0.005 | 0.002 | 0.004 | 0.005 | 0.003 |
| meta1606 |  | 564.286 | 46.644 | 0.003 | 0.005 | 0.005 | 0.004 | 0.004 | 0.003 | 0.008 | 0.005 | 0.007 | 0.003 | 0.004 | 0.007 | 0.008 | 0.002 | 0.004 |
| meta1607 |  | 564.373 | 434.191 | 0.001 | 0.001 | 0.001 | 0.001 | 0.001 | 0.001 | 0.001 | 0.001 | 0.001 | 0.001 | 0.001 | 0.001 | 0.001 | 0.001 | 0.001 |
| meta1608 |  | 564.369 | 34.825 | 0.001 | 0.001 | 0.002 | 0.003 | 0.003 | 0.003 | 0.003 | 0.005 | 0.003 | 0.002 | 0.003 | 0.003 | 0.001 | 0.002 | 0.003 |
| meta1609 |  | 564.871 | 318.133 | 0.002 | 0.001 | 0.002 | 0.002 | 0.002 | 0.002 | 0.001 | 0.001 | 0.002 | 0.002 | 0.002 | 0.001 | 0.002 | 0.002 | 0.002 |
| meta1610 | UDP-D-Galactose | 565.045 | 433.591 | 0.064 | 0.049 | 0.056 | 0.035 | 0.035 | 0.046 | 0.034 | 0.057 | 0.071 | 0.143 | 0.089 | 0.073 | 0.087 | 0.060 | 0.071 |
| meta1611 |  | 565.284 | 177.978 | 0.001 | 0.001 | 0.000 | 0.001 | 0.001 | 0.000 | 0.001 | 0.001 | 0.000 | 0.000 | 0.001 | 0.001 | 0.000 | 0.000 | 0.001 |
| meta1612 |  | 565.359 | 171.193 | 0.012 | 0.011 | 0.012 | 0.015 | 0.015 | 0.010 | 0.017 | 0.015 | 0.011 | 0.010 | 0.013 | 0.013 | 0.011 | 0.010 | 0.017 |
| meta1613 |  | 566.091 | 416.584 | 0.010 | 0.012 | 0.011 | 0.013 | 0.012 | 0.011 | 0.005 | 0.018 | 0.011 | 0.015 | 0.012 | 0.005 | 0.013 | 0.007 | 0.005 |
| meta1614 |  | 566.121 | 433.643 | 0.351 | 0.299 | 0.309 | 0.363 | 0.336 | 0.275 | 0.333 | 0.411 | 0.356 | 0.373 | 0.328 | 0.330 | 0.348 | 0.261 | 0.305 |
| meta1615 |  | 566.155 | 427.519 | 0.032 | 0.026 | 0.027 | 0.042 | 0.024 | 0.032 | 0.025 | 0.028 | 0.014 | 0.023 | 0.030 | 0.036 | 0.010 | 0.017 | 0.014 |
| meta1616 |  | 566.233 | 24.994 | 0.001 | 0.001 | 0.000 | 0.000 | 0.001 | 0.000 | 0.000 | 0.001 | 0.000 | 0.000 | 0.001 | 0.001 | 0.001 | 0.001 | 0.001 |
| meta1617 |  | 566.324 | 168.983 | 0.001 | 0.001 | 0.001 | 0.001 | 0.001 | 0.000 | 0.001 | 0.001 | 0.001 | 0.001 | 0.001 | 0.001 | 0.000 | 0.001 | 0.001 |
| meta1618 |  | 566.386 | 33.880 | 0.002 | 0.002 | 0.002 | 0.008 | 0.004 | 0.005 | 0.004 | 0.009 | 0.004 | 0.004 | 0.004 | 0.003 | 0.002 | 0.001 | 0.004 |
| meta1619 |  | 566.380 | 175.481 | 0.002 | 0.002 | 0.002 | 0.004 | 0.003 | 0.003 | 0.002 | 0.002 | 0.001 | 0.002 | 0.003 | 0.003 | 0.003 | 0.002 | 0.004 |
| meta1620 |  | 567.069 | 150.800 | 0.002 | 0.001 | 0.001 | 0.002 | 0.002 | 0.001 | 0.002 | 0.001 | 0.001 | 0.001 | 0.001 | 0.001 | 0.001 | 0.001 | 0.001 |
| meta1621 |  | 567.241 | 157.926 | 0.000 | 0.000 | 0.000 | 0.000 | 0.000 | 0.000 | 0.000 | 0.000 | 0.000 | 0.000 | 0.000 | 0.001 | 0.000 | 0.000 | 0.001 |
| meta1622 |  | 567.276 | 28.169 | 0.003 | 0.003 | 0.002 | 0.003 | 0.002 | 0.004 | 0.003 | 0.003 | 0.007 | 0.009 | 0.003 | 0.004 | 0.004 | 0.003 | 0.001 |
| meta1623 |  | 569.120 | 150.812 | 0.001 | 0.001 | 0.001 | 0.001 | 0.001 | 0.001 | 0.002 | 0.001 | 0.000 | 0.000 | 0.001 | 0.001 | 0.000 | 0.001 | 0.001 |
| meta1624 |  | 569.226 | 46.578 | 0.000 | 0.000 | 0.000 | 0.000 | 0.000 | 0.000 | 0.001 | 0.001 | 0.000 | 0.000 | 0.000 | 0.001 | 0.000 | 0.001 | 0.000 |
| meta1625 |  | 569.251 | 150.822 | 0.001 | 0.001 | 0.001 | 0.001 | 0.001 | 0.001 | 0.001 | 0.001 | 0.001 | 0.000 | 0.001 | 0.001 | 0.001 | 0.001 | 0.001 |
| meta1626 |  | 569.312 | 36.005 | 0.005 | 0.005 | 0.006 | 0.005 | 0.003 | 0.006 | 0.007 | 0.007 | 0.008 | 0.019 | 0.007 | 0.012 | 0.006 | 0.006 | 0.001 |
| meta1627 | ADP-glucose | 570.073 | 390.918 | 0.001 | 0.001 | 0.001 | 0.001 | 0.001 | 0.000 | 0.001 | 0.001 | 0.001 | 0.001 | 0.000 | 0.001 | 0.001 | 0.001 | 0.001 |
| meta1628 |  | 570.224 | 163.083 | 0.002 | 0.001 | 0.002 | 0.000 | 0.000 | 0.001 | 0.004 | 0.002 | 0.000 | 0.002 | 0.003 | 0.002 | 0.002 | 0.002 | 0.001 |
| meta1629 |  | 570.307 | 139.582 | 0.003 | 0.003 | 0.003 | 0.001 | 0.001 | 0.002 | 0.003 | 0.003 | 0.001 | 0.011 | 0.002 | 0.002 | 0.003 | 0.007 | 0.001 |
| meta1630 |  | 570.338 | 190.279 | 0.001 | 0.001 | 0.001 | 0.001 | 0.001 | 0.001 | 0.001 | 0.001 | 0.001 | 0.001 | 0.001 | 0.001 | 0.001 | 0.001 | 0.001 |
| meta1631 |  | 570.723 | 288.335 | 0.001 | 0.001 | 0.001 | 0.002 | 0.001 | 0.001 | 0.001 | 0.002 | 0.002 | 0.002 | 0.001 | 0.001 | 0.002 | 0.002 | 0.001 |
| meta1632 |  | 571.053 | 356.774 | 0.001 | 0.001 | 0.001 | 0.001 | 0.001 | 0.001 | 0.001 | 0.001 | 0.001 | 0.001 | 0.001 | 0.001 | 0.001 | 0.000 | 0.001 |
| meta1633 |  | 571.128 | 200.241 | 0.002 | 0.001 | 0.002 | 0.002 | 0.002 | 0.001 | 0.002 | 0.001 | 0.001 | 0.001 | 0.002 | 0.002 | 0.001 | 0.002 | 0.001 |
| meta1634 |  | 571.286 | 231.702 | 0.013 | 0.012 | 0.012 | 0.014 | 0.012 | 0.010 | 0.020 | 0.022 | 0.008 | 0.012 | 0.014 | 0.012 | 0.010 | 0.013 | 0.008 |
| meta1635 |  | 572.077 | 379.989 | 0.012 | 0.011 | 0.011 | 0.013 | 0.014 | 0.007 | 0.014 | 0.017 | 0.012 | 0.011 | 0.007 | 0.009 | 0.011 | 0.007 | 0.013 |
| meta1636 |  | 572.240 | 161.217 | 0.028 | 0.028 | 0.026 | 0.007 | 0.011 | 0.027 | 0.027 | 0.042 | 0.012 | 0.045 | 0.026 | 0.020 | 0.038 | 0.038 | 0.019 |
| meta1637 |  | 572.479 | 33.550 | 0.002 | 0.001 | 0.002 | 0.000 | 0.000 | 0.000 | 0.000 | 0.001 | 0.000 | 0.000 | 0.001 | 0.001 | 0.001 | 0.000 | 0.000 |
| meta1638 |  | 573.072 | 408.824 | 0.002 | 0.002 | 0.002 | 0.002 | 0.002 | 0.002 | 0.002 | 0.002 | 0.002 | 0.002 | 0.002 | 0.002 | 0.002 | 0.001 | 0.002 |
| meta1639 |  | 573.125 | 333.782 | 0.015 | 0.011 | 0.010 | 0.017 | 0.016 | 0.012 | 0.006 | 0.032 | 0.007 | 0.023 | 0.009 | 0.005 | 0.011 | 0.010 | 0.009 |
| meta1640 |  | 573.217 | 361.373 | 0.004 | 0.003 | 0.003 | 0.004 | 0.004 | 0.002 | 0.006 | 0.003 | 0.002 | 0.004 | 0.005 | 0.004 | 0.003 | 0.001 | 0.006 |
| meta1641 |  | 573.377 | 46.723 | 0.000 | 0.001 | 0.001 | 0.001 | 0.001 | 0.001 | 0.001 | 0.001 | 0.000 | 0.000 | 0.001 | 0.002 | 0.001 | 0.000 | 0.001 |
| meta1642 |  | 573.412 | 47.461 | 0.000 | 0.000 | 0.000 | 0.001 | 0.001 | 0.001 | 0.001 | 0.000 | 0.000 | 0.001 | 0.001 | 0.002 | 0.001 | 0.000 | 0.000 |
| meta1643 |  | 574.288 | 46.632 | 0.002 | 0.001 | 0.001 | 0.002 | 0.002 | 0.002 | 0.003 | 0.002 | 0.002 | 0.002 | 0.002 | 0.002 | 0.002 | 0.002 | 0.003 |
| meta1644 |  | 574.449 | 32.049 | 0.003 | 0.004 | 0.003 | 0.001 | 0.001 | 0.001 | 0.004 | 0.002 | 0.003 | 0.004 | 0.004 | 0.010 | 0.003 | 0.002 | 0.002 |
| meta1645 |  | 574.683 | 289.005 | 0.001 | 0.001 | 0.001 | 0.001 | 0.001 | 0.001 | 0.001 | 0.001 | 0.001 | 0.001 | 0.001 | 0.001 | 0.001 | 0.001 | 0.001 |
| meta1646 |  | 574.968 | 429.314 | 0.001 | 0.001 | 0.001 | 0.001 | 0.001 | 0.000 | 0.001 | 0.001 | 0.001 | 0.001 | 0.001 | 0.001 | 0.001 | 0.001 | 0.001 |
| meta1647 |  | 575.392 | 46.314 | 0.001 | 0.001 | 0.002 | 0.002 | 0.001 | 0.001 | 0.003 | 0.001 | 0.001 | 0.001 | 0.001 | 0.009 | 0.002 | 0.000 | 0.001 |
| meta1648 |  | 576.327 | 173.873 | 0.004 | 0.004 | 0.007 | 0.005 | 0.006 | 0.007 | 0.006 | 0.005 | 0.005 | 0.004 | 0.004 | 0.006 | 0.006 | 0.008 | 0.005 |
| meta1649 |  | 576.370 | 34.468 | 0.001 | 0.001 | 0.001 | 0.002 | 0.001 | 0.001 | 0.002 | 0.002 | 0.001 | 0.001 | 0.001 | 0.001 | 0.000 | 0.000 | 0.002 |
| meta1650 |  | 577.008 | 458.232 | 0.001 | 0.001 | 0.001 | 0.001 | 0.001 | 0.001 | 0.001 | 0.002 | 0.001 | 0.001 | 0.001 | 0.001 | 0.002 | 0.001 | 0.001 |
| meta1651 |  | 577.371 | 158.634 | 0.001 | 0.002 | 0.002 | 0.003 | 0.004 | 0.003 | 0.001 | 0.001 | 0.001 | 0.003 | 0.002 | 0.001 | 0.001 | 0.001 | 0.004 |
| meta1652 |  | 578.034 | 420.569 | 0.006 | 0.005 | 0.005 | 0.006 | 0.005 | 0.004 | 0.004 | 0.006 | 0.006 | 0.007 | 0.006 | 0.006 | 0.008 | 0.006 | 0.006 |
| meta1653 |  | 578.093 | 417.227 | 0.003 | 0.002 | 0.002 | 0.002 | 0.002 | 0.001 | 0.005 | 0.002 | 0.002 | 0.002 | 0.001 | 0.002 | 0.003 | 0.001 | 0.001 |
| meta1654 |  | 578.237 | 123.911 | 0.001 | 0.001 | 0.001 | 0.000 | 0.000 | 0.002 | 0.001 | 0.001 | 0.000 | 0.002 | 0.000 | 0.001 | 0.002 | 0.003 | 0.001 |
| meta1655 |  | 578.300 | 163.090 | 0.060 | 0.054 | 0.048 | 0.083 | 0.118 | 0.069 | 0.140 | 0.042 | 0.058 | 0.047 | 0.063 | 0.075 | 0.067 | 0.062 | 0.174 |
| meta1656 | Uridine 5'-diphosphoglucuronic acid (UDP-D-glucuronate) | 579.024 | 467.231 | 0.008 | 0.006 | 0.006 | 0.005 | 0.006 | 0.004 | 0.005 | 0.012 | 0.011 | 0.010 | 0.009 | 0.009 | 0.014 | 0.006 | 0.008 |
| meta1657 |  | 579.115 | 202.033 | 0.001 | 0.001 | 0.001 | 0.001 | 0.001 | 0.001 | 0.001 | 0.001 | 0.001 | 0.001 | 0.001 | 0.001 | 0.001 | 0.001 | 0.001 |
| meta1658 |  | 579.149 | 338.472 | 0.011 | 0.010 | 0.009 | 0.013 | 0.008 | 0.013 | 0.009 | 0.010 | 0.007 | 0.008 | 0.012 | 0.014 | 0.008 | 0.008 | 0.006 |
| meta1659 |  | 579.386 | 159.984 | 0.003 | 0.003 | 0.003 | 0.005 | 0.004 | 0.003 | 0.004 | 0.003 | 0.002 | 0.007 | 0.003 | 0.003 | 0.002 | 0.002 | 0.002 |
| meta1660 |  | 580.027 | 467.360 | 0.001 | 0.001 | 0.001 | 0.001 | 0.001 | 0.001 | 0.001 | 0.002 | 0.002 | 0.002 | 0.002 | 0.002 | 0.002 | 0.001 | 0.001 |
| meta1661 |  | 580.055 | 446.141 | 0.009 | 0.008 | 0.008 | 0.006 | 0.005 | 0.007 | 0.006 | 0.008 | 0.009 | 0.016 | 0.012 | 0.010 | 0.011 | 0.010 | 0.011 |
| meta1662 |  | 580.099 | 427.444 | 0.011 | 0.010 | 0.008 | 0.009 | 0.010 | 0.006 | 0.010 | 0.012 | 0.010 | 0.014 | 0.012 | 0.009 | 0.012 | 0.008 | 0.008 |
| meta1663 |  | 580.289 | 136.753 | 0.009 | 0.007 | 0.008 | 0.006 | 0.005 | 0.011 | 0.015 | 0.010 | 0.003 | 0.016 | 0.013 | 0.006 | 0.014 | 0.013 | 0.010 |
| meta1664 |  | 580.359 | 171.641 | 0.104 | 0.080 | 0.104 | 0.123 | 0.156 | 0.109 | 0.190 | 0.130 | 0.078 | 0.084 | 0.104 | 0.135 | 0.113 | 0.111 | 0.134 |
| meta1665 |  | 580.580 | 170.993 | 0.001 | 0.001 | 0.001 | 0.001 | 0.000 | 0.000 | 0.001 | 0.000 | 0.001 | 0.000 | 0.001 | 0.000 | 0.001 | 0.000 | 0.001 |
| meta1666 |  | 580.902 | 318.683 | 0.001 | 0.001 | 0.001 | 0.001 | 0.001 | 0.001 | 0.001 | 0.001 | 0.001 | 0.001 | 0.001 | 0.001 | 0.001 | 0.001 | 0.001 |
| meta1667 |  | 581.083 | 463.165 | 0.023 | 0.016 | 0.020 | 0.026 | 0.028 | 0.030 | 0.021 | 0.035 | 0.033 | 0.035 | 0.015 | 0.031 | 0.048 | 0.021 | 0.018 |
| meta1668 |  | 581.453 | 41.372 | 0.008 | 0.010 | 0.012 | 0.009 | 0.006 | 0.010 | 0.007 | 0.010 | 0.012 | 0.009 | 0.007 | 0.010 | 0.014 | 0.014 | 0.009 |
| meta1669 |  | 582.116 | 472.231 | 0.061 | 0.053 | 0.054 | 0.061 | 0.061 | 0.048 | 0.062 | 0.076 | 0.071 | 0.082 | 0.052 | 0.057 | 0.064 | 0.052 | 0.054 |
| meta1670 |  | 582.234 | 168.341 | 0.000 | 0.000 | 0.000 | 0.000 | 0.000 | 0.000 | 0.000 | 0.000 | 0.000 | 0.000 | 0.000 | 0.000 | 0.001 | 0.000 | 0.000 |
| meta1671 |  | 582.343 | 41.188 | 0.002 | 0.002 | 0.002 | 0.004 | 0.006 | 0.004 | 0.004 | 0.004 | 0.001 | 0.004 | 0.007 | 0.002 | 0.003 | 0.002 | 0.006 |
| meta1672 | 1-Stearoyl-sn-glycerol 3-phosphocholine | 582.376 | 170.993 | 0.491 | 0.494 | 0.547 | 0.675 | 0.634 | 0.419 | 0.799 | 0.665 | 0.455 | 0.473 | 0.653 | 0.567 | 0.533 | 0.428 | 0.818 |
| meta1673 |  | 583.264 | 137.229 | 0.004 | 0.004 | 0.004 | 0.003 | 0.003 | 0.007 | 0.010 | 0.006 | 0.003 | 0.010 | 0.007 | 0.003 | 0.007 | 0.009 | 0.005 |
| meta1674 |  | 583.291 | 43.285 | 0.005 | 0.005 | 0.004 | 0.004 | 0.005 | 0.007 | 0.007 | 0.006 | 0.003 | 0.011 | 0.010 | 0.011 | 0.007 | 0.007 | 0.003 |
| meta1675 |  | 584.136 | 480.754 | 0.003 | 0.003 | 0.003 | 0.004 | 0.004 | 0.003 | 0.002 | 0.006 | 0.003 | 0.006 | 0.003 | 0.005 | 0.005 | 0.003 | 0.003 |
| meta1676 |  | 585.096 | 150.812 | 0.005 | 0.004 | 0.004 | 0.004 | 0.005 | 0.003 | 0.007 | 0.004 | 0.002 | 0.001 | 0.003 | 0.004 | 0.003 | 0.003 | 0.003 |
| meta1677 |  | 585.102 | 419.257 | 0.007 | 0.007 | 0.006 | 0.008 | 0.006 | 0.005 | 0.006 | 0.007 | 0.006 | 0.007 | 0.007 | 0.006 | 0.006 | 0.005 | 0.006 |
| meta1678 |  | 585.161 | 427.896 | 0.003 | 0.002 | 0.003 | 0.003 | 0.003 | 0.003 | 0.003 | 0.003 | 0.002 | 0.003 | 0.003 | 0.003 | 0.002 | 0.002 | 0.001 |
| meta1679 |  | 585.301 | 229.542 | 0.001 | 0.001 | 0.001 | 0.001 | 0.001 | 0.001 | 0.001 | 0.001 | 0.001 | 0.001 | 0.001 | 0.001 | 0.001 | 0.001 | 0.000 |
| meta1680 |  | 585.354 | 170.993 | 0.015 | 0.016 | 0.015 | 0.020 | 0.018 | 0.012 | 0.021 | 0.019 | 0.014 | 0.015 | 0.018 | 0.016 | 0.016 | 0.013 | 0.022 |
| meta1681 |  | 585.951 | 416.947 | 0.001 | 0.001 | 0.001 | 0.001 | 0.001 | 0.001 | 0.001 | 0.001 | 0.001 | 0.001 | 0.000 | 0.001 | 0.001 | 0.001 | 0.001 |
| meta1682 |  | 586.056 | 383.989 | 0.001 | 0.001 | 0.001 | 0.001 | 0.001 | 0.001 | 0.001 | 0.001 | 0.001 | 0.001 | 0.001 | 0.002 | 0.001 | 0.001 | 0.001 |
| meta1683 |  | 586.333 | 186.278 | 0.001 | 0.001 | 0.001 | 0.002 | 0.001 | 0.001 | 0.001 | 0.001 | 0.001 | 0.001 | 0.001 | 0.001 | 0.001 | 0.001 | 0.001 |
| meta1684 |  | 587.001 | 96.782 | 0.002 | 0.001 | 0.002 | 0.000 | 0.002 | 0.000 | 0.006 | 0.001 | 0.001 | 0.001 | 0.001 | 0.000 | 0.002 | 0.000 | 0.004 |
| meta1685 | Uridine diphosphate glucose(UDP-D-Glucose) | 587.026 | 434.169 | 0.004 | 0.003 | 0.003 | 0.003 | 0.003 | 0.003 | 0.003 | 0.004 | 0.004 | 0.007 | 0.005 | 0.005 | 0.005 | 0.004 | 0.004 |
| meta1686 |  | 587.180 | 468.777 | 0.001 | 0.001 | 0.001 | 0.001 | 0.001 | 0.001 | 0.001 | 0.001 | 0.001 | 0.001 | 0.001 | 0.001 | 0.001 | 0.000 | 0.000 |
| meta1687 |  | 587.344 | 45.925 | 0.001 | 0.002 | 0.001 | 0.004 | 0.000 | 0.002 | 0.001 | 0.006 | 0.002 | 0.002 | 0.007 | 0.004 | 0.002 | 0.002 | 0.004 |
| meta1688 |  | 587.391 | 46.510 | 0.000 | 0.000 | 0.000 | 0.000 | 0.001 | 0.000 | 0.000 | 0.000 | 0.001 | 0.000 | 0.001 | 0.001 | 0.001 | 0.000 | 0.000 |
| meta1689 |  | 588.072 | 442.405 | 0.001 | 0.001 | 0.001 | 0.001 | 0.001 | 0.001 | 0.001 | 0.001 | 0.001 | 0.001 | 0.001 | 0.001 | 0.001 | 0.001 | 0.000 |
| meta1690 |  | 588.102 | 434.283 | 0.013 | 0.011 | 0.011 | 0.013 | 0.012 | 0.009 | 0.012 | 0.013 | 0.012 | 0.012 | 0.012 | 0.012 | 0.011 | 0.009 | 0.011 |
| meta1691 |  | 588.214 | 160.605 | 0.003 | 0.003 | 0.002 | 0.000 | 0.001 | 0.003 | 0.002 | 0.006 | 0.001 | 0.005 | 0.002 | 0.002 | 0.003 | 0.005 | 0.002 |
| meta1692 |  | 588.426 | 33.461 | 0.000 | 0.000 | 0.000 | 0.000 | 0.000 | 0.000 | 0.000 | 0.000 | 0.000 | 0.001 | 0.001 | 0.002 | 0.001 | 0.001 | 0.000 |
| meta1693 |  | 588.465 | 31.402 | 0.002 | 0.001 | 0.001 | 0.000 | 0.000 | 0.000 | 0.000 | 0.000 | 0.000 | 0.001 | 0.001 | 0.003 | 0.001 | 0.000 | 0.000 |
| meta1694 |  | 590.311 | 174.202 | 0.001 | 0.001 | 0.001 | 0.001 | 0.001 | 0.001 | 0.001 | 0.001 | 0.001 | 0.001 | 0.001 | 0.001 | 0.001 | 0.001 | 0.001 |
| meta1695 |  | 590.383 | 45.359 | 0.001 | 0.001 | 0.001 | 0.002 | 0.001 | 0.001 | 0.000 | 0.002 | 0.001 | 0.001 | 0.001 | 0.002 | 0.000 | 0.001 | 0.000 |
| meta1696 |  | 590.444 | 38.001 | 0.001 | 0.001 | 0.001 | 0.000 | 0.000 | 0.000 | 0.002 | 0.001 | 0.001 | 0.002 | 0.002 | 0.004 | 0.001 | 0.001 | 0.001 |
| meta1697 |  | 591.005 | 85.857 | 0.001 | 0.001 | 0.001 | 0.002 | 0.002 | 0.001 | 0.002 | 0.001 | 0.001 | 0.001 | 0.001 | 0.000 | 0.001 | 0.001 | 0.002 |
| meta1698 |  | 591.112 | 150.812 | 0.005 | 0.004 | 0.004 | 0.005 | 0.006 | 0.004 | 0.007 | 0.005 | 0.003 | 0.002 | 0.003 | 0.004 | 0.003 | 0.004 | 0.003 |
| meta1699 |  | 591.115 | 414.595 | 0.002 | 0.001 | 0.001 | 0.002 | 0.001 | 0.001 | 0.001 | 0.003 | 0.002 | 0.002 | 0.002 | 0.002 | 0.002 | 0.001 | 0.001 |
| meta1700 |  | 591.188 | 371.557 | 0.000 | 0.000 | 0.000 | 0.000 | 0.000 | 0.000 | 0.000 | 0.000 | 0.000 | 0.000 | 0.000 | 0.000 | 0.000 | 0.000 | 0.000 |
| meta1701 |  | 592.099 | 468.777 | 0.001 | 0.001 | 0.001 | 0.002 | 0.002 | 0.002 | 0.002 | 0.002 | 0.002 | 0.003 | 0.001 | 0.002 | 0.003 | 0.001 | 0.001 |
| meta1702 |  | 592.252 | 135.190 | 0.001 | 0.001 | 0.001 | 0.001 | 0.001 | 0.001 | 0.002 | 0.001 | 0.000 | 0.001 | 0.001 | 0.001 | 0.001 | 0.001 | 0.001 |
| meta1703 |  | 592.321 | 214.833 | 0.000 | 0.000 | 0.000 | 0.001 | 0.000 | 0.000 | 0.000 | 0.000 | 0.000 | 0.000 | 0.000 | 0.000 | 0.000 | 0.000 | 0.000 |
| meta1704 |  | 592.401 | 33.622 | 0.000 | 0.000 | 0.000 | 0.001 | 0.001 | 0.001 | 0.001 | 0.001 | 0.000 | 0.001 | 0.001 | 0.001 | 0.000 | 0.000 | 0.001 |
| meta1705 |  | 592.792 | 287.985 | 0.002 | 0.002 | 0.001 | 0.002 | 0.003 | 0.002 | 0.002 | 0.002 | 0.003 | 0.002 | 0.002 | 0.002 | 0.003 | 0.002 | 0.002 |
| meta1706 |  | 593.039 | 443.381 | 0.001 | 0.001 | 0.001 | 0.001 | 0.001 | 0.001 | 0.001 | 0.001 | 0.001 | 0.001 | 0.001 | 0.001 | 0.001 | 0.001 | 0.001 |
| meta1707 |  | 593.154 | 427.602 | 0.007 | 0.006 | 0.007 | 0.008 | 0.007 | 0.009 | 0.007 | 0.009 | 0.006 | 0.007 | 0.006 | 0.010 | 0.007 | 0.005 | 0.003 |
| meta1708 |  | 593.303 | 24.989 | 0.002 | 0.002 | 0.002 | 0.002 | 0.002 | 0.001 | 0.002 | 0.003 | 0.003 | 0.002 | 0.003 | 0.004 | 0.003 | 0.003 | 0.004 |
| meta1709 |  | 593.355 | 174.343 | 0.002 | 0.002 | 0.002 | 0.002 | 0.002 | 0.001 | 0.002 | 0.003 | 0.002 | 0.002 | 0.003 | 0.002 | 0.002 | 0.002 | 0.001 |
| meta1710 |  | 594.035 | 472.113 | 0.001 | 0.001 | 0.001 | 0.001 | 0.001 | 0.001 | 0.001 | 0.002 | 0.002 | 0.002 | 0.002 | 0.002 | 0.002 | 0.001 | 0.001 |
| meta1711 |  | 594.115 | 425.710 | 0.003 | 0.002 | 0.003 | 0.002 | 0.002 | 0.002 | 0.003 | 0.005 | 0.003 | 0.003 | 0.003 | 0.003 | 0.003 | 0.002 | 0.002 |
| meta1712 |  | 594.238 | 243.323 | 0.008 | 0.007 | 0.008 | 0.004 | 0.004 | 0.007 | 0.012 | 0.010 | 0.006 | 0.012 | 0.008 | 0.005 | 0.011 | 0.011 | 0.004 |
| meta1713 |  | 594.268 | 125.002 | 0.005 | 0.004 | 0.005 | 0.002 | 0.001 | 0.009 | 0.014 | 0.008 | 0.001 | 0.011 | 0.004 | 0.007 | 0.013 | 0.011 | 0.003 |
| meta1714 |  | 594.301 | 132.816 | 0.004 | 0.004 | 0.005 | 0.005 | 0.004 | 0.003 | 0.006 | 0.006 | 0.002 | 0.002 | 0.006 | 0.005 | 0.003 | 0.004 | 0.006 |
| meta1715 |  | 594.338 | 227.485 | 0.001 | 0.001 | 0.001 | 0.003 | 0.002 | 0.002 | 0.001 | 0.001 | 0.002 | 0.001 | 0.001 | 0.001 | 0.002 | 0.001 | 0.001 |
| meta1716 |  | 594.372 | 170.641 | 0.001 | 0.001 | 0.001 | 0.001 | 0.001 | 0.000 | 0.001 | 0.001 | 0.000 | 0.001 | 0.001 | 0.001 | 0.001 | 0.001 | 0.001 |
| meta1717 |  | 595.106 | 333.924 | 0.016 | 0.012 | 0.011 | 0.019 | 0.016 | 0.013 | 0.007 | 0.024 | 0.008 | 0.020 | 0.011 | 0.006 | 0.013 | 0.011 | 0.011 |
| meta1718 |  | 595.198 | 361.425 | 0.001 | 0.001 | 0.001 | 0.001 | 0.001 | 0.001 | 0.002 | 0.001 | 0.001 | 0.001 | 0.001 | 0.001 | 0.001 | 0.001 | 0.002 |
| meta1719 |  | 595.315 | 242.477 | 0.001 | 0.000 | 0.001 | 0.001 | 0.001 | 0.001 | 0.001 | 0.001 | 0.001 | 0.001 | 0.000 | 0.000 | 0.000 | 0.001 | 0.001 |
| meta1720 |  | 596.283 | 182.987 | 0.016 | 0.015 | 0.015 | 0.006 | 0.007 | 0.018 | 0.024 | 0.017 | 0.006 | 0.021 | 0.015 | 0.009 | 0.018 | 0.022 | 0.010 |
| meta1721 |  | 596.390 | 169.653 | 0.008 | 0.008 | 0.008 | 0.010 | 0.006 | 0.006 | 0.012 | 0.009 | 0.009 | 0.009 | 0.013 | 0.010 | 0.009 | 0.007 | 0.008 |
| meta1722 |  | 596.524 | 33.526 | 0.002 | 0.002 | 0.002 | 0.000 | 0.000 | 0.000 | 0.000 | 0.001 | 0.000 | 0.001 | 0.001 | 0.001 | 0.001 | 0.000 | 0.000 |
| meta1723 |  | 596.824 | 582.091 | 0.004 | 0.003 | 0.003 | 0.004 | 0.003 | 0.003 | 0.003 | 0.004 | 0.003 | 0.004 | 0.004 | 0.004 | 0.003 | 0.003 | 0.003 |
| meta1724 |  | 597.125 | 401.844 | 0.001 | 0.001 | 0.001 | 0.001 | 0.001 | 0.001 | 0.001 | 0.001 | 0.001 | 0.001 | 0.001 | 0.001 | 0.001 | 0.001 | 0.001 |
| meta1725 |  | 597.167 | 458.096 | 0.000 | 0.000 | 0.000 | 0.000 | 0.001 | 0.000 | 0.001 | 0.000 | 0.000 | 0.000 | 0.000 | 0.000 | 0.001 | 0.000 | 0.000 |
| meta1726 |  | 597.245 | 136.248 | 0.001 | 0.002 | 0.001 | 0.001 | 0.001 | 0.003 | 0.003 | 0.002 | 0.001 | 0.002 | 0.002 | 0.001 | 0.002 | 0.002 | 0.002 |
| meta1727 |  | 597.301 | 228.776 | 0.006 | 0.007 | 0.007 | 0.007 | 0.006 | 0.004 | 0.008 | 0.010 | 0.004 | 0.008 | 0.006 | 0.008 | 0.010 | 0.010 | 0.006 |
| meta1728 |  | 597.995 | 415.556 | 0.001 | 0.000 | 0.001 | 0.001 | 0.000 | 0.000 | 0.000 | 0.001 | 0.001 | 0.000 | 0.001 | 0.001 | 0.000 | 0.000 | 0.001 |
| meta1729 |  | 598.147 | 200.241 | 0.003 | 0.003 | 0.003 | 0.003 | 0.004 | 0.003 | 0.004 | 0.002 | 0.002 | 0.002 | 0.003 | 0.003 | 0.002 | 0.003 | 0.002 |
| meta1730 |  | 598.164 | 333.377 | 0.001 | 0.001 | 0.001 | 0.001 | 0.001 | 0.001 | 0.001 | 0.001 | 0.000 | 0.001 | 0.001 | 0.001 | 0.001 | 0.001 | 0.001 |
| meta1731 |  | 598.166 | 371.506 | 0.001 | 0.000 | 0.000 | 0.001 | 0.001 | 0.000 | 0.001 | 0.001 | 0.000 | 0.001 | 0.001 | 0.000 | 0.000 | 0.000 | 0.001 |
| meta1732 |  | 598.369 | 186.278 | 0.001 | 0.001 | 0.001 | 0.001 | 0.001 | 0.001 | 0.001 | 0.001 | 0.001 | 0.001 | 0.001 | 0.001 | 0.001 | 0.001 | 0.002 |
| meta1733 |  | 598.729 | 289.174 | 0.000 | 0.000 | 0.001 | 0.001 | 0.000 | 0.000 | 0.000 | 0.000 | 0.001 | 0.000 | 0.001 | 0.001 | 0.001 | 0.001 | 0.001 |
| meta1734 |  | 598.952 | 318.698 | 0.002 | 0.001 | 0.002 | 0.002 | 0.001 | 0.001 | 0.001 | 0.002 | 0.001 | 0.002 | 0.002 | 0.001 | 0.001 | 0.002 | 0.002 |
| meta1735 |  | 599.259 | 183.506 | 0.008 | 0.007 | 0.006 | 0.005 | 0.005 | 0.006 | 0.007 | 0.008 | 0.005 | 0.008 | 0.010 | 0.007 | 0.008 | 0.007 | 0.006 |
| meta1736 |  | 599.318 | 227.680 | 0.055 | 0.049 | 0.053 | 0.067 | 0.061 | 0.043 | 0.093 | 0.075 | 0.030 | 0.041 | 0.050 | 0.056 | 0.035 | 0.038 | 0.045 |
| meta1737 |  | 599.498 | 33.549 | 0.002 | 0.002 | 0.002 | 0.000 | 0.000 | 0.000 | 0.000 | 0.001 | 0.000 | 0.001 | 0.001 | 0.001 | 0.001 | 0.000 | 0.000 |
| meta1738 |  | 600.015 | 23.889 | 0.001 | 0.001 | 0.001 | 0.001 | 0.000 | 0.001 | 0.001 | 0.000 | 0.001 | 0.001 | 0.001 | 0.001 | 0.001 | 0.001 | 0.001 |
| meta1739 |  | 600.132 | 479.079 | 0.001 | 0.001 | 0.001 | 0.001 | 0.001 | 0.001 | 0.001 | 0.001 | 0.001 | 0.001 | 0.001 | 0.001 | 0.001 | 0.000 | 0.001 |
| meta1740 |  | 600.291 | 113.771 | 0.003 | 0.003 | 0.003 | 0.004 | 0.004 | 0.004 | 0.004 | 0.003 | 0.002 | 0.002 | 0.005 | 0.005 | 0.003 | 0.007 | 0.006 |
| meta1741 |  | 601.073 | 419.323 | 0.001 | 0.001 | 0.001 | 0.001 | 0.001 | 0.001 | 0.001 | 0.001 | 0.001 | 0.001 | 0.001 | 0.001 | 0.001 | 0.001 | 0.001 |
| meta1742 |  | 601.409 | 147.656 | 0.000 | 0.000 | 0.000 | 0.000 | 0.000 | 0.000 | 0.000 | 0.001 | 0.001 | 0.000 | 0.000 | 0.001 | 0.000 | 0.000 | 0.000 |
| meta1743 |  | 602.038 | 446.173 | 0.001 | 0.001 | 0.001 | 0.001 | 0.001 | 0.001 | 0.001 | 0.001 | 0.001 | 0.002 | 0.002 | 0.001 | 0.001 | 0.001 | 0.001 |
| meta1744 |  | 602.298 | 162.457 | 0.027 | 0.025 | 0.022 | 0.025 | 0.039 | 0.033 | 0.046 | 0.028 | 0.022 | 0.021 | 0.031 | 0.027 | 0.028 | 0.025 | 0.017 |
| meta1745 |  | 602.344 | 169.639 | 0.092 | 0.097 | 0.103 | 0.170 | 0.141 | 0.102 | 0.144 | 0.188 | 0.113 | 0.118 | 0.124 | 0.142 | 0.100 | 0.109 | 0.173 |
| meta1746 |  | 602.444 | 33.103 | 0.001 | 0.001 | 0.001 | 0.000 | 0.000 | 0.000 | 0.001 | 0.000 | 0.001 | 0.001 | 0.002 | 0.003 | 0.001 | 0.001 | 0.000 |
| meta1747 |  | 603.387 | 119.869 | 0.004 | 0.003 | 0.003 | 0.007 | 0.006 | 0.004 | 0.005 | 0.006 | 0.003 | 0.004 | 0.005 | 0.004 | 0.001 | 0.001 | 0.005 |
| meta1748 | Guanosine diphosphate mannose | 604.072 | 432.032 | 0.004 | 0.003 | 0.003 | 0.005 | 0.003 | 0.002 | 0.003 | 0.004 | 0.003 | 0.003 | 0.003 | 0.003 | 0.003 | 0.003 | 0.003 |
| meta1749 |  | 605.099 | 471.561 | 0.001 | 0.001 | 0.001 | 0.001 | 0.001 | 0.001 | 0.001 | 0.001 | 0.001 | 0.001 | 0.001 | 0.001 | 0.001 | 0.001 | 0.001 |
| meta1750 |  | 605.132 | 408.853 | 0.004 | 0.004 | 0.004 | 0.004 | 0.004 | 0.003 | 0.003 | 0.005 | 0.004 | 0.006 | 0.004 | 0.004 | 0.004 | 0.003 | 0.003 |
| meta1751 |  | 605.140 | 463.297 | 0.003 | 0.002 | 0.003 | 0.002 | 0.006 | 0.001 | 0.006 | 0.003 | 0.004 | 0.004 | 0.001 | 0.002 | 0.006 | 0.002 | 0.001 |
| meta1752 |  | 605.190 | 468.531 | 0.001 | 0.001 | 0.001 | 0.001 | 0.001 | 0.001 | 0.001 | 0.001 | 0.001 | 0.001 | 0.001 | 0.001 | 0.001 | 0.001 | 0.001 |
| meta1753 |  | 605.248 | 137.678 | 0.001 | 0.000 | 0.001 | 0.001 | 0.000 | 0.001 | 0.002 | 0.001 | 0.001 | 0.001 | 0.001 | 0.000 | 0.001 | 0.001 | 0.001 |
| meta1754 |  | 605.269 | 153.380 | 0.002 | 0.002 | 0.002 | 0.001 | 0.007 | 0.001 | 0.004 | 0.003 | 0.000 | 0.001 | 0.002 | 0.001 | 0.001 | 0.001 | 0.002 |
| meta1755 |  | 605.403 | 116.090 | 0.037 | 0.053 | 0.037 | 0.093 | 0.063 | 0.058 | 0.060 | 0.096 | 0.051 | 0.054 | 0.036 | 0.087 | 0.035 | 0.016 | 0.053 |
| meta1756 |  | 605.453 | 40.728 | 0.005 | 0.006 | 0.008 | 0.005 | 0.005 | 0.008 | 0.006 | 0.007 | 0.007 | 0.005 | 0.005 | 0.009 | 0.006 | 0.010 | 0.006 |
| meta1757 | UDP-N-acetylglucosamine | 606.073 | 420.040 | 0.260 | 0.211 | 0.205 | 0.242 | 0.247 | 0.197 | 0.262 | 0.264 | 0.231 | 0.235 | 0.214 | 0.255 | 0.290 | 0.154 | 0.242 |
| meta1758 |  | 606.373 | 170.250 | 0.003 | 0.003 | 0.003 | 0.005 | 0.003 | 0.003 | 0.004 | 0.007 | 0.003 | 0.004 | 0.004 | 0.004 | 0.003 | 0.004 | 0.005 |
| meta1759 |  | 606.381 | 38.719 | 0.003 | 0.002 | 0.002 | 0.003 | 0.004 | 0.002 | 0.004 | 0.004 | 0.002 | 0.002 | 0.003 | 0.004 | 0.002 | 0.001 | 0.003 |
| meta1760 |  | 607.082 | 150.822 | 0.002 | 0.001 | 0.001 | 0.002 | 0.003 | 0.002 | 0.003 | 0.002 | 0.001 | 0.001 | 0.001 | 0.002 | 0.001 | 0.002 | 0.001 |
| meta1761 |  | 607.382 | 145.112 | 0.001 | 0.002 | 0.001 | 0.003 | 0.001 | 0.003 | 0.002 | 0.003 | 0.001 | 0.002 | 0.001 | 0.002 | 0.001 | 0.001 | 0.001 |
| meta1762 |  | 608.130 | 421.752 | 0.011 | 0.010 | 0.009 | 0.013 | 0.009 | 0.007 | 0.007 | 0.012 | 0.008 | 0.012 | 0.011 | 0.010 | 0.009 | 0.008 | 0.010 |
| meta1763 |  | 608.353 | 228.789 | 0.001 | 0.002 | 0.002 | 0.003 | 0.002 | 0.002 | 0.001 | 0.001 | 0.003 | 0.001 | 0.001 | 0.001 | 0.001 | 0.001 | 0.001 |
| meta1764 |  | 609.185 | 365.303 | 0.001 | 0.000 | 0.000 | 0.000 | 0.001 | 0.001 | 0.000 | 0.000 | 0.000 | 0.001 | 0.000 | 0.001 | 0.000 | 0.000 | 0.000 |
| meta1765 |  | 609.300 | 26.031 | 0.003 | 0.002 | 0.002 | 0.008 | 0.001 | 0.002 | 0.002 | 0.002 | 0.002 | 0.000 | 0.003 | 0.003 | 0.002 | 0.002 | 0.004 |
| meta1766 |  | 609.328 | 168.341 | 0.001 | 0.001 | 0.001 | 0.002 | 0.002 | 0.001 | 0.002 | 0.002 | 0.001 | 0.001 | 0.001 | 0.001 | 0.001 | 0.001 | 0.002 |
| meta1767 |  | 610.111 | 466.700 | 0.049 | 0.039 | 0.046 | 0.058 | 0.062 | 0.065 | 0.051 | 0.082 | 0.074 | 0.083 | 0.041 | 0.072 | 0.104 | 0.050 | 0.044 |
| meta1768 |  | 610.162 | 433.806 | 0.003 | 0.003 | 0.003 | 0.003 | 0.003 | 0.004 | 0.002 | 0.004 | 0.003 | 0.005 | 0.004 | 0.003 | 0.003 | 0.002 | 0.002 |
| meta1769 |  | 610.265 | 46.662 | 0.001 | 0.001 | 0.001 | 0.001 | 0.001 | 0.001 | 0.001 | 0.001 | 0.001 | 0.001 | 0.001 | 0.001 | 0.001 | 0.001 | 0.001 |
| meta1770 |  | 610.405 | 168.768 | 0.003 | 0.004 | 0.003 | 0.005 | 0.002 | 0.003 | 0.004 | 0.006 | 0.007 | 0.004 | 0.004 | 0.004 | 0.003 | 0.003 | 0.003 |
| meta1771 |  | 611.080 | 333.961 | 0.001 | 0.001 | 0.001 | 0.002 | 0.001 | 0.001 | 0.001 | 0.003 | 0.001 | 0.002 | 0.001 | 0.001 | 0.001 | 0.001 | 0.001 |
| meta1772 | Glutathione disulfide | 611.143 | 478.291 | 0.143 | 0.128 | 0.150 | 0.159 | 0.163 | 0.163 | 0.150 | 0.218 | 0.216 | 0.217 | 0.130 | 0.166 | 0.218 | 0.131 | 0.134 |
| meta1773 | Glutathione, oxidized | 612.146 | 478.291 | 0.032 | 0.030 | 0.035 | 0.036 | 0.038 | 0.037 | 0.033 | 0.049 | 0.049 | 0.050 | 0.031 | 0.038 | 0.050 | 0.030 | 0.031 |
| meta1774 |  | 612.256 | 182.987 | 0.003 | 0.004 | 0.004 | 0.001 | 0.002 | 0.004 | 0.005 | 0.003 | 0.000 | 0.005 | 0.004 | 0.002 | 0.004 | 0.005 | 0.002 |
| meta1775 |  | 612.793 | 318.304 | 0.002 | 0.001 | 0.002 | 0.002 | 0.001 | 0.002 | 0.002 | 0.001 | 0.002 | 0.001 | 0.002 | 0.001 | 0.002 | 0.002 | 0.001 |
| meta1776 |  | 612.876 | 286.372 | 0.001 | 0.001 | 0.001 | 0.001 | 0.001 | 0.001 | 0.001 | 0.001 | 0.001 | 0.001 | 0.001 | 0.001 | 0.001 | 0.001 | 0.001 |
| meta1777 |  | 612.988 | 85.394 | 0.022 | 0.020 | 0.024 | 0.039 | 0.041 | 0.027 | 0.036 | 0.019 | 0.018 | 0.021 | 0.027 | 0.009 | 0.024 | 0.017 | 0.030 |
| meta1778 |  | 613.066 | 418.132 | 0.001 | 0.001 | 0.001 | 0.001 | 0.001 | 0.001 | 0.001 | 0.001 | 0.001 | 0.001 | 0.001 | 0.001 | 0.001 | 0.001 | 0.001 |
| meta1779 |  | 613.093 | 150.812 | 0.001 | 0.001 | 0.001 | 0.001 | 0.001 | 0.001 | 0.002 | 0.001 | 0.001 | 0.000 | 0.001 | 0.001 | 0.001 | 0.001 | 0.001 |
| meta1780 | Cytidine monophosphate N-acetylneuraminic acid | 613.136 | 429.123 | 0.001 | 0.001 | 0.001 | 0.001 | 0.001 | 0.001 | 0.001 | 0.001 | 0.001 | 0.001 | 0.001 | 0.001 | 0.001 | 0.001 | 0.001 |
| meta1781 |  | 613.480 | 46.649 | 0.000 | 0.000 | 0.000 | 0.000 | 0.000 | 0.000 | 0.001 | 0.001 | 0.001 | 0.001 | 0.001 | 0.000 | 0.003 | 0.001 | 0.001 |
| meta1782 |  | 614.328 | 214.018 | 0.006 | 0.005 | 0.005 | 0.006 | 0.005 | 0.005 | 0.007 | 0.009 | 0.003 | 0.004 | 0.007 | 0.007 | 0.005 | 0.005 | 0.005 |
| meta1783 |  | 615.091 | 284.465 | 0.001 | 0.001 | 0.001 | 0.001 | 0.001 | 0.001 | 0.001 | 0.001 | 0.000 | 0.000 | 0.001 | 0.001 | 0.001 | 0.001 | 0.001 |
| meta1784 |  | 615.092 | 200.328 | 0.001 | 0.001 | 0.001 | 0.001 | 0.001 | 0.000 | 0.001 | 0.001 | 0.001 | 0.001 | 0.001 | 0.001 | 0.001 | 0.001 | 0.001 |
| meta1785 |  | 615.306 | 174.134 | 0.001 | 0.001 | 0.001 | 0.001 | 0.001 | 0.001 | 0.001 | 0.001 | 0.001 | 0.001 | 0.001 | 0.001 | 0.001 | 0.001 | 0.001 |
| meta1786 |  | 616.066 | 396.118 | 0.003 | 0.002 | 0.002 | 0.002 | 0.003 | 0.002 | 0.002 | 0.003 | 0.002 | 0.002 | 0.002 | 0.004 | 0.002 | 0.002 | 0.002 |
| meta1787 |  | 616.219 | 243.259 | 0.000 | 0.000 | 0.000 | 0.000 | 0.000 | 0.000 | 0.000 | 0.000 | 0.000 | 0.000 | 0.000 | 0.000 | 0.000 | 0.000 | 0.000 |
| meta1788 |  | 616.459 | 32.903 | 0.001 | 0.001 | 0.001 | 0.000 | 0.000 | 0.000 | 0.001 | 0.000 | 0.000 | 0.001 | 0.001 | 0.002 | 0.001 | 0.000 | 0.000 |
| meta1789 |  | 616.957 | 156.625 | 0.000 | 0.000 | 0.000 | 0.001 | 0.000 | 0.000 | 0.000 | 0.000 | 0.000 | 0.000 | 0.000 | 0.000 | 0.000 | 0.000 | 0.000 |
| meta1790 |  | 616.970 | 25.089 | 0.001 | 0.001 | 0.001 | 0.001 | 0.001 | 0.001 | 0.001 | 0.002 | 0.002 | 0.002 | 0.001 | 0.000 | 0.001 | 0.000 | 0.000 |
| meta1791 |  | 617.088 | 334.110 | 0.002 | 0.002 | 0.002 | 0.003 | 0.002 | 0.002 | 0.001 | 0.002 | 0.001 | 0.002 | 0.001 | 0.001 | 0.002 | 0.002 | 0.002 |
| meta1792 |  | 617.133 | 416.947 | 0.013 | 0.012 | 0.011 | 0.014 | 0.012 | 0.011 | 0.011 | 0.015 | 0.012 | 0.013 | 0.016 | 0.015 | 0.009 | 0.012 | 0.009 |
| meta1793 |  | 617.141 | 200.682 | 0.001 | 0.001 | 0.001 | 0.001 | 0.001 | 0.001 | 0.001 | 0.000 | 0.001 | 0.001 | 0.001 | 0.001 | 0.001 | 0.001 | 0.001 |
| meta1794 |  | 617.356 | 32.259 | 0.001 | 0.000 | 0.000 | 0.001 | 0.000 | 0.001 | 0.001 | 0.001 | 0.000 | 0.000 | 0.000 | 0.001 | 0.000 | 0.000 | 0.001 |
| meta1795 |  | 618.255 | 161.218 | 0.002 | 0.002 | 0.002 | 0.000 | 0.001 | 0.002 | 0.001 | 0.002 | 0.001 | 0.003 | 0.001 | 0.002 | 0.002 | 0.002 | 0.002 |
| meta1796 |  | 618.301 | 132.040 | 0.002 | 0.001 | 0.001 | 0.002 | 0.001 | 0.001 | 0.001 | 0.002 | 0.001 | 0.001 | 0.003 | 0.003 | 0.002 | 0.003 | 0.002 |
| meta1797 |  | 618.579 | 32.934 | 0.001 | 0.001 | 0.001 | 0.000 | 0.000 | 0.000 | 0.000 | 0.000 | 0.000 | 0.000 | 0.001 | 0.001 | 0.000 | 0.000 | 0.000 |
| meta1798 |  | 619.067 | 393.461 | 0.004 | 0.003 | 0.003 | 0.006 | 0.003 | 0.003 | 0.003 | 0.003 | 0.003 | 0.003 | 0.003 | 0.003 | 0.004 | 0.003 | 0.003 |
| meta1799 |  | 619.287 | 220.270 | 0.042 | 0.040 | 0.042 | 0.075 | 0.089 | 0.055 | 0.099 | 0.060 | 0.033 | 0.061 | 0.059 | 0.050 | 0.037 | 0.074 | 0.048 |
| meta1800 |  | 619.315 | 242.272 | 0.000 | 0.000 | 0.000 | 0.001 | 0.001 | 0.001 | 0.000 | 0.000 | 0.001 | 0.001 | 0.000 | 0.000 | 0.000 | 0.001 | 0.001 |
| meta1801 |  | 620.127 | 200.678 | 0.001 | 0.001 | 0.001 | 0.001 | 0.001 | 0.001 | 0.001 | 0.001 | 0.001 | 0.001 | 0.001 | 0.001 | 0.001 | 0.001 | 0.001 |
| meta1802 |  | 620.201 | 342.498 | 0.002 | 0.001 | 0.001 | 0.002 | 0.002 | 0.002 | 0.002 | 0.002 | 0.001 | 0.001 | 0.002 | 0.002 | 0.001 | 0.001 | 0.001 |
| meta1803 |  | 620.237 | 366.295 | 0.001 | 0.001 | 0.001 | 0.001 | 0.001 | 0.001 | 0.001 | 0.001 | 0.000 | 0.001 | 0.001 | 0.002 | 0.001 | 0.000 | 0.000 |
| meta1804 |  | 621.035 | 449.606 | 0.001 | 0.001 | 0.001 | 0.001 | 0.001 | 0.001 | 0.001 | 0.001 | 0.001 | 0.001 | 0.001 | 0.001 | 0.001 | 0.001 | 0.001 |
| meta1805 |  | 621.110 | 27.235 | 0.001 | 0.001 | 0.002 | 0.002 | 0.003 | 0.002 | 0.001 | 0.001 | 0.001 | 0.001 | 0.001 | 0.001 | 0.001 | 0.001 | 0.001 |
| meta1806 |  | 621.385 | 171.077 | 0.001 | 0.001 | 0.001 | 0.001 | 0.001 | 0.001 | 0.001 | 0.001 | 0.001 | 0.001 | 0.001 | 0.001 | 0.001 | 0.001 | 0.001 |
| meta1807 |  | 622.912 | 318.698 | 0.001 | 0.001 | 0.001 | 0.001 | 0.001 | 0.001 | 0.001 | 0.001 | 0.001 | 0.001 | 0.001 | 0.001 | 0.001 | 0.001 | 0.001 |
| meta1808 |  | 623.045 | 417.083 | 0.000 | 0.001 | 0.000 | 0.001 | 0.000 | 0.000 | 0.000 | 0.000 | 0.000 | 0.000 | 0.000 | 0.000 | 0.000 | 0.000 | 0.000 |
| meta1809 |  | 623.321 | 27.856 | 0.002 | 0.003 | 0.003 | 0.014 | 0.007 | 0.005 | 0.002 | 0.001 | 0.003 | 0.001 | 0.001 | 0.002 | 0.003 | 0.002 | 0.001 |
| meta1810 |  | 623.355 | 46.001 | 0.000 | 0.000 | 0.000 | 0.000 | 0.000 | 0.000 | 0.001 | 0.001 | 0.001 | 0.000 | 0.001 | 0.001 | 0.000 | 0.000 | 0.000 |
| meta1811 |  | 623.412 | 145.112 | 0.005 | 0.005 | 0.004 | 0.012 | 0.004 | 0.008 | 0.008 | 0.009 | 0.004 | 0.004 | 0.005 | 0.006 | 0.003 | 0.002 | 0.004 |
| meta1812 |  | 623.994 | 161.870 | 0.000 | 0.000 | 0.000 | 0.001 | 0.001 | 0.000 | 0.001 | 0.000 | 0.001 | 0.000 | 0.000 | 0.000 | 0.000 | 0.001 | 0.001 |
| meta1813 |  | 624.063 | 273.874 | 0.011 | 0.009 | 0.009 | 0.012 | 0.013 | 0.010 | 0.008 | 0.017 | 0.008 | 0.016 | 0.008 | 0.005 | 0.010 | 0.010 | 0.009 |
| meta1814 |  | 624.181 | 160.599 | 0.004 | 0.003 | 0.003 | 0.001 | 0.001 | 0.003 | 0.003 | 0.006 | 0.001 | 0.006 | 0.003 | 0.002 | 0.005 | 0.005 | 0.003 |
| meta1815 | Leukotriene C4 | 624.291 | 113.735 | 0.003 | 0.003 | 0.003 | 0.003 | 0.004 | 0.003 | 0.005 | 0.003 | 0.002 | 0.002 | 0.005 | 0.003 | 0.005 | 0.007 | 0.005 |
| meta1816 |  | 624.327 | 169.596 | 0.000 | 0.000 | 0.000 | 0.001 | 0.001 | 0.000 | 0.000 | 0.001 | 0.000 | 0.000 | 0.000 | 0.000 | 0.000 | 0.001 | 0.000 |
| meta1817 |  | 625.480 | 45.908 | 0.000 | 0.000 | 0.001 | 0.000 | 0.001 | 0.000 | 0.001 | 0.001 | 0.001 | 0.001 | 0.001 | 0.000 | 0.001 | 0.001 | 0.002 |
| meta1818 |  | 626.078 | 472.234 | 0.001 | 0.000 | 0.000 | 0.001 | 0.000 | 0.000 | 0.001 | 0.001 | 0.001 | 0.001 | 0.001 | 0.000 | 0.001 | 0.000 | 0.001 |
| meta1819 |  | 626.344 | 168.848 | 0.038 | 0.037 | 0.045 | 0.072 | 0.064 | 0.042 | 0.060 | 0.080 | 0.051 | 0.042 | 0.055 | 0.055 | 0.042 | 0.047 | 0.056 |
| meta1820 |  | 627.497 | 47.420 | 0.029 | 0.029 | 0.036 | 0.024 | 0.033 | 0.014 | 0.026 | 0.035 | 0.060 | 0.045 | 0.036 | 0.011 | 0.136 | 0.076 | 0.116 |
| meta1821 |  | 628.053 | 420.347 | 0.006 | 0.005 | 0.005 | 0.006 | 0.006 | 0.004 | 0.006 | 0.005 | 0.005 | 0.005 | 0.005 | 0.005 | 0.005 | 0.004 | 0.005 |
| meta1822 |  | 628.173 | 367.750 | 0.002 | 0.001 | 0.001 | 0.002 | 0.002 | 0.002 | 0.001 | 0.002 | 0.001 | 0.002 | 0.001 | 0.001 | 0.001 | 0.001 | 0.001 |
| meta1823 |  | 628.766 | 287.665 | 0.003 | 0.002 | 0.002 | 0.004 | 0.004 | 0.003 | 0.003 | 0.004 | 0.004 | 0.003 | 0.003 | 0.003 | 0.005 | 0.003 | 0.003 |
| meta1824 |  | 628.962 | 85.498 | 0.008 | 0.007 | 0.007 | 0.011 | 0.012 | 0.008 | 0.010 | 0.006 | 0.005 | 0.007 | 0.009 | 0.003 | 0.007 | 0.005 | 0.008 |
| meta1825 |  | 629.064 | 150.809 | 0.001 | 0.001 | 0.001 | 0.001 | 0.001 | 0.001 | 0.001 | 0.001 | 0.001 | 0.000 | 0.001 | 0.000 | 0.001 | 0.001 | 0.001 |
| meta1826 |  | 629.132 | 443.483 | 0.009 | 0.007 | 0.008 | 0.009 | 0.008 | 0.008 | 0.007 | 0.011 | 0.008 | 0.012 | 0.009 | 0.009 | 0.011 | 0.006 | 0.007 |
| meta1827 |  | 629.290 | 153.706 | 0.001 | 0.001 | 0.001 | 0.002 | 0.003 | 0.002 | 0.002 | 0.003 | 0.002 | 0.001 | 0.000 | 0.001 | 0.000 | 0.001 | 0.000 |
| meta1828 |  | 629.329 | 157.972 | 0.003 | 0.003 | 0.003 | 0.004 | 0.003 | 0.003 | 0.010 | 0.006 | 0.002 | 0.001 | 0.003 | 0.007 | 0.004 | 0.004 | 0.004 |
| meta1829 |  | 629.453 | 40.431 | 0.014 | 0.013 | 0.013 | 0.014 | 0.011 | 0.017 | 0.012 | 0.015 | 0.016 | 0.014 | 0.014 | 0.018 | 0.016 | 0.022 | 0.013 |
| meta1830 |  | 630.116 | 429.987 | 0.002 | 0.001 | 0.002 | 0.002 | 0.001 | 0.001 | 0.002 | 0.002 | 0.001 | 0.002 | 0.002 | 0.002 | 0.002 | 0.001 | 0.002 |
| meta1831 |  | 630.197 | 159.991 | 0.001 | 0.001 | 0.001 | 0.000 | 0.000 | 0.001 | 0.001 | 0.002 | 0.000 | 0.002 | 0.001 | 0.001 | 0.002 | 0.001 | 0.001 |
| meta1832 |  | 630.375 | 168.310 | 0.001 | 0.001 | 0.001 | 0.002 | 0.002 | 0.001 | 0.002 | 0.002 | 0.002 | 0.001 | 0.002 | 0.002 | 0.001 | 0.002 | 0.002 |
| meta1833 |  | 630.747 | 287.985 | 0.003 | 0.002 | 0.002 | 0.003 | 0.003 | 0.003 | 0.003 | 0.003 | 0.003 | 0.003 | 0.003 | 0.002 | 0.003 | 0.003 | 0.003 |
| meta1834 |  | 631.287 | 28.011 | 0.002 | 0.002 | 0.002 | 0.006 | 0.004 | 0.002 | 0.001 | 0.001 | 0.001 | 0.001 | 0.000 | 0.001 | 0.001 | 0.001 | 0.001 |
| meta1835 |  | 632.063 | 418.113 | 0.003 | 0.003 | 0.002 | 0.003 | 0.002 | 0.002 | 0.002 | 0.003 | 0.002 | 0.003 | 0.002 | 0.002 | 0.002 | 0.002 | 0.002 |
| meta1836 |  | 632.201 | 412.645 | 0.001 | 0.001 | 0.001 | 0.001 | 0.001 | 0.002 | 0.001 | 0.001 | 0.001 | 0.001 | 0.001 | 0.002 | 0.001 | 0.001 | 0.001 |
| meta1837 |  | 633.119 | 200.549 | 0.001 | 0.001 | 0.001 | 0.002 | 0.002 | 0.001 | 0.002 | 0.001 | 0.001 | 0.001 | 0.001 | 0.001 | 0.001 | 0.001 | 0.001 |
| meta1838 |  | 633.186 | 367.820 | 0.002 | 0.002 | 0.003 | 0.002 | 0.003 | 0.003 | 0.003 | 0.004 | 0.002 | 0.003 | 0.002 | 0.004 | 0.003 | 0.002 | 0.001 |
| meta1839 |  | 633.185 | 398.216 | 0.004 | 0.003 | 0.004 | 0.004 | 0.003 | 0.005 | 0.004 | 0.004 | 0.003 | 0.004 | 0.004 | 0.005 | 0.004 | 0.003 | 0.002 |
| meta1840 |  | 634.253 | 367.237 | 0.001 | 0.001 | 0.001 | 0.000 | 0.001 | 0.001 | 0.001 | 0.001 | 0.000 | 0.001 | 0.001 | 0.002 | 0.001 | 0.000 | 0.000 |
| meta1841 |  | 634.369 | 224.064 | 0.001 | 0.001 | 0.001 | 0.001 | 0.001 | 0.001 | 0.000 | 0.000 | 0.001 | 0.001 | 0.000 | 0.000 | 0.001 | 0.000 | 0.000 |
| meta1842 |  | 634.380 | 170.977 | 0.000 | 0.000 | 0.000 | 0.001 | 0.000 | 0.000 | 0.001 | 0.000 | 0.000 | 0.000 | 0.001 | 0.000 | 0.000 | 0.000 | 0.001 |
| meta1843 |  | 635.061 | 418.710 | 0.010 | 0.011 | 0.010 | 0.012 | 0.008 | 0.008 | 0.009 | 0.012 | 0.010 | 0.012 | 0.008 | 0.009 | 0.009 | 0.008 | 0.009 |
| meta1844 |  | 635.126 | 26.984 | 0.001 | 0.001 | 0.001 | 0.003 | 0.001 | 0.001 | 0.001 | 0.001 | 0.001 | 0.001 | 0.001 | 0.001 | 0.001 | 0.001 | 0.001 |
| meta1845 |  | 635.321 | 31.392 | 0.000 | 0.001 | 0.001 | 0.001 | 0.003 | 0.000 | 0.001 | 0.001 | 0.000 | 0.001 | 0.000 | 0.001 | 0.000 | 0.001 | 0.001 |
| meta1846 |  | 635.359 | 46.674 | 0.001 | 0.001 | 0.001 | 0.001 | 0.001 | 0.000 | 0.002 | 0.001 | 0.001 | 0.001 | 0.001 | 0.007 | 0.001 | 0.000 | 0.001 |
| meta1847 |  | 635.412 | 47.279 | 0.001 | 0.001 | 0.001 | 0.002 | 0.001 | 0.001 | 0.002 | 0.001 | 0.001 | 0.001 | 0.001 | 0.007 | 0.001 | 0.000 | 0.001 |
| meta1848 |  | 636.094 | 200.679 | 0.001 | 0.001 | 0.001 | 0.001 | 0.001 | 0.001 | 0.001 | 0.001 | 0.001 | 0.001 | 0.001 | 0.001 | 0.001 | 0.001 | 0.001 |
| meta1849 |  | 636.259 | 163.073 | 0.002 | 0.002 | 0.003 | 0.003 | 0.003 | 0.002 | 0.003 | 0.002 | 0.002 | 0.002 | 0.003 | 0.003 | 0.003 | 0.003 | 0.002 |
| meta1850 |  | 636.346 | 174.343 | 0.006 | 0.006 | 0.006 | 0.009 | 0.007 | 0.007 | 0.010 | 0.007 | 0.006 | 0.006 | 0.007 | 0.006 | 0.006 | 0.006 | 0.007 |
| meta1851 |  | 636.688 | 288.888 | 0.001 | 0.001 | 0.002 | 0.002 | 0.002 | 0.002 | 0.002 | 0.001 | 0.002 | 0.002 | 0.001 | 0.001 | 0.002 | 0.001 | 0.002 |
| meta1852 |  | 637.180 | 343.522 | 0.001 | 0.001 | 0.001 | 0.001 | 0.001 | 0.001 | 0.001 | 0.001 | 0.000 | 0.001 | 0.001 | 0.001 | 0.001 | 0.000 | 0.000 |
| meta1853 |  | 637.180 | 427.654 | 0.009 | 0.007 | 0.007 | 0.009 | 0.007 | 0.010 | 0.006 | 0.010 | 0.005 | 0.012 | 0.009 | 0.011 | 0.006 | 0.004 | 0.004 |
| meta1854 |  | 637.336 | 27.544 | 0.004 | 0.005 | 0.005 | 0.027 | 0.017 | 0.008 | 0.003 | 0.003 | 0.003 | 0.002 | 0.001 | 0.002 | 0.004 | 0.002 | 0.002 |
| meta1855 |  | 637.393 | 161.231 | 0.002 | 0.003 | 0.003 | 0.006 | 0.004 | 0.005 | 0.005 | 0.003 | 0.002 | 0.002 | 0.002 | 0.004 | 0.002 | 0.002 | 0.002 |
| meta1856 |  | 637.538 | 33.715 | 0.000 | 0.000 | 0.000 | 0.000 | 0.000 | 0.000 | 0.000 | 0.000 | 0.000 | 0.000 | 0.000 | 0.001 | 0.001 | 0.000 | 0.000 |
| meta1857 |  | 638.164 | 371.084 | 0.001 | 0.001 | 0.001 | 0.001 | 0.001 | 0.001 | 0.001 | 0.001 | 0.000 | 0.001 | 0.001 | 0.001 | 0.001 | 0.001 | 0.000 |
| meta1858 |  | 638.328 | 212.480 | 0.003 | 0.002 | 0.002 | 0.003 | 0.002 | 0.002 | 0.003 | 0.004 | 0.001 | 0.003 | 0.003 | 0.005 | 0.005 | 0.004 | 0.002 |
| meta1859 |  | 638.364 | 154.794 | 0.002 | 0.002 | 0.002 | 0.003 | 0.004 | 0.003 | 0.002 | 0.001 | 0.002 | 0.002 | 0.001 | 0.001 | 0.001 | 0.002 | 0.001 |
| meta1860 |  | 639.132 | 201.619 | 0.003 | 0.003 | 0.002 | 0.003 | 0.003 | 0.002 | 0.003 | 0.002 | 0.002 | 0.002 | 0.003 | 0.003 | 0.003 | 0.002 | 0.003 |
| meta1861 |  | 639.408 | 163.686 | 0.003 | 0.003 | 0.003 | 0.006 | 0.003 | 0.006 | 0.005 | 0.003 | 0.002 | 0.002 | 0.002 | 0.003 | 0.002 | 0.002 | 0.002 |
| meta1862 |  | 640.137 | 461.896 | 0.006 | 0.005 | 0.006 | 0.007 | 0.005 | 0.006 | 0.005 | 0.005 | 0.005 | 0.006 | 0.005 | 0.006 | 0.008 | 0.004 | 0.005 |
| meta1863 |  | 640.214 | 361.727 | 0.000 | 0.000 | 0.000 | 0.001 | 0.000 | 0.000 | 0.000 | 0.001 | 0.000 | 0.001 | 0.000 | 0.000 | 0.000 | 0.000 | 0.000 |
| meta1864 |  | 640.344 | 211.300 | 0.003 | 0.003 | 0.003 | 0.003 | 0.003 | 0.002 | 0.003 | 0.004 | 0.001 | 0.003 | 0.004 | 0.005 | 0.002 | 0.004 | 0.003 |
| meta1865 |  | 642.110 | 201.481 | 0.002 | 0.002 | 0.001 | 0.002 | 0.002 | 0.001 | 0.002 | 0.002 | 0.001 | 0.002 | 0.002 | 0.002 | 0.002 | 0.001 | 0.002 |
| meta1866 |  | 642.302 | 129.438 | 0.006 | 0.006 | 0.005 | 0.007 | 0.006 | 0.007 | 0.004 | 0.007 | 0.003 | 0.006 | 0.010 | 0.007 | 0.006 | 0.009 | 0.008 |
| meta1867 |  | 642.358 | 210.343 | 0.003 | 0.002 | 0.003 | 0.003 | 0.002 | 0.002 | 0.003 | 0.005 | 0.002 | 0.002 | 0.004 | 0.003 | 0.002 | 0.002 | 0.003 |
| meta1868 |  | 643.064 | 430.839 | 0.001 | 0.001 | 0.001 | 0.001 | 0.001 | 0.001 | 0.001 | 0.001 | 0.001 | 0.001 | 0.001 | 0.001 | 0.001 | 0.001 | 0.001 |
| meta1869 |  | 643.070 | 24.944 | 0.002 | 0.001 | 0.002 | 0.003 | 0.001 | 0.002 | 0.003 | 0.009 | 0.008 | 0.008 | 0.003 | 0.001 | 0.001 | 0.001 | 0.000 |
| meta1870 |  | 643.146 | 427.602 | 0.011 | 0.008 | 0.009 | 0.013 | 0.009 | 0.010 | 0.008 | 0.012 | 0.006 | 0.009 | 0.011 | 0.014 | 0.007 | 0.006 | 0.005 |
| meta1871 |  | 643.167 | 345.208 | 0.001 | 0.001 | 0.001 | 0.001 | 0.001 | 0.002 | 0.001 | 0.001 | 0.001 | 0.001 | 0.001 | 0.002 | 0.001 | 0.001 | 0.001 |
| meta1872 |  | 643.194 | 351.587 | 0.000 | 0.000 | 0.000 | 0.001 | 0.000 | 0.000 | 0.001 | 0.001 | 0.001 | 0.001 | 0.000 | 0.000 | 0.001 | 0.001 | 0.000 |
| meta1873 |  | 643.286 | 218.943 | 0.000 | 0.001 | 0.001 | 0.001 | 0.001 | 0.000 | 0.001 | 0.002 | 0.001 | 0.001 | 0.001 | 0.001 | 0.000 | 0.000 | 0.000 |
| meta1874 |  | 643.315 | 239.466 | 0.000 | 0.000 | 0.000 | 0.001 | 0.001 | 0.001 | 0.000 | 0.000 | 0.000 | 0.000 | 0.000 | 0.000 | 0.000 | 0.000 | 0.000 |
| meta1875 |  | 643.379 | 142.775 | 0.000 | 0.000 | 0.000 | 0.001 | 0.001 | 0.001 | 0.000 | 0.001 | 0.000 | 0.000 | 0.000 | 0.001 | 0.000 | 0.000 | 0.000 |
| meta1876 |  | 644.027 | 420.706 | 0.001 | 0.001 | 0.001 | 0.001 | 0.001 | 0.001 | 0.001 | 0.001 | 0.001 | 0.001 | 0.001 | 0.001 | 0.001 | 0.001 | 0.001 |
| meta1877 |  | 644.142 | 453.672 | 0.001 | 0.001 | 0.001 | 0.001 | 0.002 | 0.001 | 0.001 | 0.001 | 0.001 | 0.002 | 0.001 | 0.001 | 0.001 | 0.001 | 0.001 |
| meta1878 |  | 644.595 | 32.903 | 0.002 | 0.002 | 0.002 | 0.000 | 0.000 | 0.000 | 0.000 | 0.001 | 0.000 | 0.000 | 0.001 | 0.002 | 0.001 | 0.000 | 0.000 |
| meta1879 |  | 644.797 | 287.193 | 0.002 | 0.001 | 0.001 | 0.003 | 0.002 | 0.002 | 0.003 | 0.002 | 0.002 | 0.002 | 0.002 | 0.002 | 0.003 | 0.002 | 0.002 |
| meta1880 |  | 645.181 | 427.806 | 0.002 | 0.002 | 0.002 | 0.002 | 0.002 | 0.002 | 0.002 | 0.003 | 0.002 | 0.002 | 0.002 | 0.003 | 0.002 | 0.001 | 0.001 |
| meta1881 |  | 645.301 | 27.951 | 0.005 | 0.005 | 0.004 | 0.015 | 0.009 | 0.006 | 0.003 | 0.003 | 0.003 | 0.002 | 0.002 | 0.003 | 0.003 | 0.003 | 0.003 |
| meta1882 |  | 645.330 | 46.493 | 0.001 | 0.001 | 0.000 | 0.000 | 0.001 | 0.001 | 0.001 | 0.001 | 0.001 | 0.000 | 0.001 | 0.001 | 0.001 | 0.000 | 0.000 |
| meta1883 |  | 645.425 | 40.431 | 0.001 | 0.001 | 0.001 | 0.001 | 0.001 | 0.001 | 0.002 | 0.001 | 0.001 | 0.001 | 0.001 | 0.001 | 0.001 | 0.002 | 0.001 |
| meta1884 |  | 645.507 | 48.777 | 0.002 | 0.001 | 0.002 | 0.001 | 0.001 | 0.001 | 0.000 | 0.002 | 0.004 | 0.003 | 0.001 | 0.001 | 0.005 | 0.002 | 0.000 |
| meta1885 |  | 646.044 | 273.665 | 0.001 | 0.001 | 0.001 | 0.002 | 0.001 | 0.001 | 0.001 | 0.001 | 0.001 | 0.002 | 0.001 | 0.001 | 0.001 | 0.001 | 0.001 |
| meta1886 |  | 646.612 | 32.983 | 0.005 | 0.004 | 0.004 | 0.001 | 0.000 | 0.000 | 0.000 | 0.002 | 0.001 | 0.001 | 0.003 | 0.005 | 0.001 | 0.000 | 0.001 |
| meta1887 |  | 647.133 | 199.229 | 0.001 | 0.001 | 0.001 | 0.000 | 0.001 | 0.000 | 0.001 | 0.000 | 0.000 | 0.001 | 0.000 | 0.001 | 0.001 | 0.001 | 0.001 |
| meta1888 |  | 647.213 | 350.821 | 0.036 | 0.026 | 0.027 | 0.034 | 0.031 | 0.043 | 0.028 | 0.036 | 0.021 | 0.033 | 0.032 | 0.045 | 0.030 | 0.022 | 0.016 |
| meta1889 |  | 647.332 | 168.016 | 0.001 | 0.001 | 0.001 | 0.001 | 0.001 | 0.000 | 0.001 | 0.001 | 0.001 | 0.001 | 0.001 | 0.001 | 0.001 | 0.001 | 0.001 |
| meta1890 |  | 647.341 | 187.679 | 0.002 | 0.002 | 0.002 | 0.002 | 0.001 | 0.001 | 0.006 | 0.001 | 0.000 | 0.000 | 0.003 | 0.010 | 0.004 | 0.001 | 0.003 |
| meta1891 |  | 647.341 | 79.372 | 0.003 | 0.002 | 0.002 | 0.001 | 0.001 | 0.001 | 0.005 | 0.001 | 0.002 | 0.002 | 0.003 | 0.003 | 0.006 | 0.002 | 0.003 |
| meta1892 |  | 647.411 | 47.250 | 0.000 | 0.000 | 0.000 | 0.001 | 0.000 | 0.000 | 0.000 | 0.000 | 0.000 | 0.000 | 0.001 | 0.000 | 0.000 | 0.000 | 0.000 |
| meta1893 |  | 648.196 | 380.561 | 0.002 | 0.002 | 0.002 | 0.002 | 0.002 | 0.002 | 0.001 | 0.003 | 0.001 | 0.002 | 0.002 | 0.003 | 0.002 | 0.001 | 0.001 |
| meta1894 |  | 648.196 | 438.532 | 0.001 | 0.001 | 0.001 | 0.001 | 0.001 | 0.001 | 0.001 | 0.001 | 0.001 | 0.001 | 0.001 | 0.001 | 0.001 | 0.001 | 0.001 |
| meta1895 |  | 648.243 | 398.740 | 0.001 | 0.000 | 0.000 | 0.001 | 0.001 | 0.001 | 0.000 | 0.001 | 0.000 | 0.000 | 0.000 | 0.001 | 0.001 | 0.000 | 0.000 |
| meta1896 |  | 648.385 | 219.024 | 0.001 | 0.000 | 0.001 | 0.001 | 0.001 | 0.001 | 0.000 | 0.000 | 0.001 | 0.001 | 0.000 | 0.000 | 0.000 | 0.000 | 0.000 |
| meta1897 |  | 648.420 | 141.582 | 0.000 | 0.000 | 0.000 | 0.001 | 0.001 | 0.000 | 0.000 | 0.000 | 0.001 | 0.000 | 0.000 | 0.000 | 0.000 | 0.000 | 0.000 |
| meta1898 |  | 649.428 | 47.324 | 0.000 | 0.000 | 0.000 | 0.001 | 0.000 | 0.001 | 0.001 | 0.000 | 0.002 | 0.001 | 0.001 | 0.001 | 0.001 | 0.001 | 0.000 |
| meta1899 |  | 650.060 | 21.129 | 0.000 | 0.000 | 0.000 | 0.000 | 0.000 | 0.000 | 0.000 | 0.001 | 0.000 | 0.000 | 0.000 | 0.000 | 0.000 | 0.000 | 0.000 |
| meta1900 |  | 650.153 | 463.821 | 0.001 | 0.001 | 0.001 | 0.000 | 0.000 | 0.000 | 0.001 | 0.001 | 0.001 | 0.001 | 0.000 | 0.001 | 0.001 | 0.000 | 0.000 |
| meta1901 |  | 650.354 | 170.919 | 0.000 | 0.000 | 0.000 | 0.001 | 0.001 | 0.000 | 0.001 | 0.001 | 0.000 | 0.000 | 0.000 | 0.000 | 0.000 | 0.000 | 0.000 |
| meta1902 |  | 650.839 | 286.434 | 0.000 | 0.000 | 0.000 | 0.000 | 0.000 | 0.000 | 0.000 | 0.001 | 0.000 | 0.000 | 0.000 | 0.000 | 0.001 | 0.000 | 0.000 |
| meta1903 |  | 651.045 | 150.192 | 0.000 | 0.000 | 0.000 | 0.000 | 0.000 | 0.000 | 0.000 | 0.000 | 0.000 | 0.000 | 0.000 | 0.000 | 0.000 | 0.001 | 0.000 |
| meta1904 |  | 651.119 | 383.767 | 0.001 | 0.001 | 0.001 | 0.001 | 0.001 | 0.000 | 0.001 | 0.001 | 0.000 | 0.001 | 0.001 | 0.001 | 0.001 | 0.001 | 0.001 |
| meta1905 |  | 651.352 | 27.544 | 0.004 | 0.005 | 0.005 | 0.030 | 0.017 | 0.009 | 0.005 | 0.005 | 0.003 | 0.003 | 0.001 | 0.003 | 0.004 | 0.004 | 0.003 |
| meta1906 |  | 652.043 | 465.516 | 0.001 | 0.001 | 0.001 | 0.002 | 0.002 | 0.001 | 0.001 | 0.001 | 0.002 | 0.002 | 0.001 | 0.001 | 0.002 | 0.001 | 0.002 |
| meta1907 |  | 652.168 | 474.871 | 0.006 | 0.006 | 0.007 | 0.005 | 0.004 | 0.005 | 0.004 | 0.007 | 0.008 | 0.010 | 0.007 | 0.009 | 0.009 | 0.006 | 0.005 |
| meta1908 |  | 652.319 | 174.523 | 0.001 | 0.001 | 0.001 | 0.001 | 0.001 | 0.001 | 0.001 | 0.001 | 0.001 | 0.001 | 0.001 | 0.001 | 0.001 | 0.001 | 0.002 |
| meta1909 |  | 652.367 | 170.977 | 0.004 | 0.004 | 0.004 | 0.005 | 0.005 | 0.004 | 0.004 | 0.006 | 0.002 | 0.004 | 0.004 | 0.006 | 0.004 | 0.003 | 0.004 |
| meta1910 |  | 653.153 | 470.782 | 0.007 | 0.005 | 0.005 | 0.006 | 0.006 | 0.006 | 0.005 | 0.007 | 0.008 | 0.009 | 0.005 | 0.007 | 0.011 | 0.005 | 0.006 |
| meta1911 |  | 653.288 | 231.804 | 0.000 | 0.000 | 0.000 | 0.000 | 0.000 | 0.000 | 0.000 | 0.001 | 0.000 | 0.000 | 0.000 | 0.000 | 0.000 | 0.000 | 0.000 |
| meta1912 |  | 653.299 | 34.078 | 0.001 | 0.001 | 0.001 | 0.006 | 0.004 | 0.001 | 0.003 | 0.001 | 0.000 | 0.001 | 0.000 | 0.002 | 0.001 | 0.001 | 0.000 |
| meta1913 |  | 653.451 | 40.095 | 0.008 | 0.010 | 0.010 | 0.011 | 0.008 | 0.013 | 0.010 | 0.011 | 0.010 | 0.010 | 0.008 | 0.012 | 0.010 | 0.017 | 0.009 |
| meta1914 |  | 653.511 | 46.663 | 0.001 | 0.001 | 0.002 | 0.001 | 0.002 | 0.000 | 0.003 | 0.002 | 0.002 | 0.002 | 0.003 | 0.000 | 0.006 | 0.004 | 0.001 |
| meta1915 |  | 655.092 | 97.341 | 0.002 | 0.001 | 0.001 | 0.000 | 0.000 | 0.000 | 0.015 | 0.022 | 0.007 | 0.001 | 0.000 | 0.000 | 0.001 | 0.000 | 0.000 |
| meta1916 |  | 655.105 | 478.291 | 0.002 | 0.002 | 0.002 | 0.002 | 0.002 | 0.002 | 0.002 | 0.002 | 0.003 | 0.003 | 0.002 | 0.002 | 0.003 | 0.002 | 0.002 |
| meta1917 |  | 655.105 | 202.584 | 0.001 | 0.001 | 0.001 | 0.001 | 0.001 | 0.001 | 0.001 | 0.001 | 0.001 | 0.001 | 0.001 | 0.001 | 0.001 | 0.001 | 0.001 |
| meta1918 |  | 655.419 | 31.605 | 0.000 | 0.001 | 0.001 | 0.002 | 0.001 | 0.001 | 0.001 | 0.001 | 0.001 | 0.001 | 0.002 | 0.001 | 0.001 | 0.001 | 0.001 |
| meta1919 |  | 656.277 | 136.615 | 0.001 | 0.001 | 0.001 | 0.001 | 0.001 | 0.001 | 0.003 | 0.001 | 0.000 | 0.003 | 0.001 | 0.001 | 0.002 | 0.002 | 0.001 |
| meta1920 |  | 658.193 | 371.229 | 0.000 | 0.000 | 0.000 | 0.001 | 0.001 | 0.000 | 0.000 | 0.001 | 0.000 | 0.000 | 0.001 | 0.000 | 0.000 | 0.000 | 0.000 |
| meta1921 |  | 659.264 | 361.087 | 0.001 | 0.001 | 0.001 | 0.001 | 0.001 | 0.000 | 0.001 | 0.001 | 0.001 | 0.001 | 0.001 | 0.001 | 0.001 | 0.000 | 0.001 |
| meta1922 |  | 659.317 | 27.583 | 0.007 | 0.005 | 0.006 | 0.022 | 0.014 | 0.008 | 0.004 | 0.005 | 0.006 | 0.004 | 0.002 | 0.004 | 0.006 | 0.004 | 0.003 |
| meta1923 |  | 659.466 | 30.437 | 0.001 | 0.001 | 0.001 | 0.000 | 0.000 | 0.000 | 0.000 | 0.000 | 0.001 | 0.000 | 0.001 | 0.002 | 0.001 | 0.000 | 0.000 |
| meta1924 |  | 660.097 | 25.028 | 0.002 | 0.003 | 0.003 | 0.003 | 0.002 | 0.004 | 0.004 | 0.013 | 0.010 | 0.011 | 0.004 | 0.001 | 0.001 | 0.001 | 0.000 |
| meta1925 |  | 660.346 | 168.907 | 0.003 | 0.003 | 0.002 | 0.003 | 0.002 | 0.002 | 0.005 | 0.004 | 0.003 | 0.003 | 0.004 | 0.004 | 0.005 | 0.003 | 0.003 |
| meta1926 |  | 661.156 | 427.602 | 0.003 | 0.002 | 0.003 | 0.003 | 0.003 | 0.003 | 0.002 | 0.003 | 0.002 | 0.003 | 0.003 | 0.004 | 0.003 | 0.002 | 0.002 |
| meta1927 |  | 661.272 | 250.384 | 0.000 | 0.000 | 0.000 | 0.000 | 0.001 | 0.000 | 0.000 | 0.001 | 0.001 | 0.000 | 0.001 | 0.000 | 0.000 | 0.000 | 0.000 |
| meta1928 |  | 661.356 | 199.474 | 0.001 | 0.001 | 0.001 | 0.000 | 0.000 | 0.000 | 0.002 | 0.001 | 0.001 | 0.001 | 0.001 | 0.001 | 0.002 | 0.001 | 0.001 |
| meta1929 | Nicotinamide adenine dinucleotide (NAD) | 662.096 | 419.505 | 0.022 | 0.020 | 0.020 | 0.023 | 0.021 | 0.016 | 0.021 | 0.029 | 0.028 | 0.022 | 0.016 | 0.021 | 0.035 | 0.017 | 0.025 |
| meta1930 |  | 662.154 | 469.911 | 0.004 | 0.004 | 0.005 | 0.005 | 0.005 | 0.006 | 0.004 | 0.006 | 0.005 | 0.005 | 0.005 | 0.006 | 0.005 | 0.006 | 0.003 |
| meta1931 |  | 662.328 | 209.114 | 0.008 | 0.008 | 0.008 | 0.011 | 0.009 | 0.009 | 0.010 | 0.011 | 0.006 | 0.008 | 0.011 | 0.015 | 0.011 | 0.011 | 0.007 |
| meta1932 |  | 662.360 | 171.617 | 0.001 | 0.001 | 0.001 | 0.001 | 0.001 | 0.001 | 0.003 | 0.001 | 0.001 | 0.001 | 0.001 | 0.001 | 0.001 | 0.001 | 0.001 |
| meta1933 |  | 663.091 | 418.653 | 0.008 | 0.007 | 0.007 | 0.008 | 0.006 | 0.004 | 0.007 | 0.010 | 0.009 | 0.009 | 0.007 | 0.007 | 0.007 | 0.006 | 0.009 |
| meta1934 |  | 663.196 | 405.902 | 0.035 | 0.029 | 0.031 | 0.038 | 0.033 | 0.046 | 0.031 | 0.037 | 0.025 | 0.032 | 0.033 | 0.039 | 0.032 | 0.019 | 0.016 |
| meta1935 |  | 663.255 | 46.461 | 0.001 | 0.000 | 0.001 | 0.000 | 0.000 | 0.000 | 0.001 | 0.000 | 0.001 | 0.001 | 0.001 | 0.001 | 0.000 | 0.000 | 0.000 |
| meta1936 |  | 663.370 | 307.212 | 0.000 | 0.000 | 0.000 | 0.000 | 0.000 | 0.000 | 0.000 | 0.000 | 0.000 | 0.000 | 0.000 | 0.000 | 0.000 | 0.000 | 0.000 |
| meta1937 |  | 663.380 | 45.353 | 0.001 | 0.000 | 0.001 | 0.001 | 0.001 | 0.001 | 0.001 | 0.002 | 0.001 | 0.001 | 0.002 | 0.002 | 0.000 | 0.001 | 0.001 |
| meta1938 | Reduced nicotinamide adenine dinucleotide (NADH) | 664.122 | 457.043 | 0.001 | 0.000 | 0.000 | 0.000 | 0.001 | 0.000 | 0.000 | 0.001 | 0.001 | 0.001 | 0.000 | 0.001 | 0.001 | 0.000 | 0.000 |
| meta1939 |  | 664.377 | 170.961 | 0.003 | 0.003 | 0.003 | 0.004 | 0.004 | 0.002 | 0.004 | 0.004 | 0.003 | 0.003 | 0.004 | 0.003 | 0.003 | 0.003 | 0.004 |
| meta1940 |  | 664.416 | 216.587 | 0.001 | 0.001 | 0.001 | 0.002 | 0.001 | 0.001 | 0.001 | 0.001 | 0.001 | 0.001 | 0.001 | 0.001 | 0.001 | 0.001 | 0.001 |
| meta1941 |  | 665.065 | 433.205 | 0.008 | 0.006 | 0.007 | 0.007 | 0.007 | 0.006 | 0.007 | 0.009 | 0.008 | 0.009 | 0.006 | 0.005 | 0.008 | 0.006 | 0.006 |
| meta1942 |  | 665.212 | 468.777 | 0.049 | 0.042 | 0.048 | 0.044 | 0.040 | 0.074 | 0.047 | 0.061 | 0.044 | 0.060 | 0.047 | 0.065 | 0.053 | 0.028 | 0.022 |
| meta1943 |  | 665.272 | 46.019 | 0.002 | 0.001 | 0.002 | 0.001 | 0.001 | 0.001 | 0.001 | 0.001 | 0.002 | 0.002 | 0.001 | 0.002 | 0.002 | 0.002 | 0.001 |
| meta1944 |  | 665.367 | 27.235 | 0.003 | 0.003 | 0.003 | 0.022 | 0.012 | 0.006 | 0.003 | 0.003 | 0.002 | 0.002 | 0.002 | 0.002 | 0.005 | 0.003 | 0.002 |
| meta1945 |  | 666.057 | 21.080 | 0.017 | 0.016 | 0.016 | 0.024 | 0.014 | 0.013 | 0.015 | 0.082 | 0.022 | 0.038 | 0.020 | 0.013 | 0.136 | 0.025 | 0.029 |
| meta1946 |  | 666.301 | 128.132 | 0.004 | 0.004 | 0.004 | 0.005 | 0.006 | 0.005 | 0.009 | 0.005 | 0.003 | 0.004 | 0.007 | 0.006 | 0.008 | 0.011 | 0.006 |
| meta1947 |  | 666.381 | 171.560 | 0.001 | 0.001 | 0.001 | 0.001 | 0.001 | 0.001 | 0.001 | 0.000 | 0.001 | 0.001 | 0.001 | 0.001 | 0.001 | 0.001 | 0.001 |
| meta1948 |  | 668.701 | 288.058 | 0.001 | 0.001 | 0.001 | 0.001 | 0.001 | 0.001 | 0.001 | 0.001 | 0.001 | 0.001 | 0.001 | 0.001 | 0.001 | 0.001 | 0.001 |
| meta1949 |  | 669.021 | 434.191 | 0.001 | 0.001 | 0.001 | 0.001 | 0.001 | 0.001 | 0.001 | 0.001 | 0.001 | 0.002 | 0.001 | 0.001 | 0.001 | 0.001 | 0.001 |
| meta1950 |  | 669.147 | 479.768 | 0.004 | 0.004 | 0.006 | 0.006 | 0.006 | 0.006 | 0.006 | 0.007 | 0.006 | 0.005 | 0.004 | 0.006 | 0.008 | 0.005 | 0.005 |
| meta1951 |  | 669.191 | 25.799 | 0.001 | 0.001 | 0.002 | 0.002 | 0.001 | 0.001 | 0.001 | 0.002 | 0.002 | 0.002 | 0.001 | 0.001 | 0.001 | 0.000 | 0.000 |
| meta1952 |  | 669.473 | 30.974 | 0.001 | 0.001 | 0.001 | 0.000 | 0.000 | 0.000 | 0.000 | 0.001 | 0.000 | 0.000 | 0.001 | 0.001 | 0.000 | 0.000 | 0.000 |
| meta1953 |  | 670.089 | 429.471 | 0.002 | 0.001 | 0.001 | 0.001 | 0.001 | 0.001 | 0.001 | 0.003 | 0.002 | 0.002 | 0.002 | 0.002 | 0.002 | 0.001 | 0.002 |
| meta1954 |  | 670.277 | 387.615 | 0.000 | 0.000 | 0.000 | 0.000 | 0.000 | 0.000 | 0.000 | 0.000 | 0.000 | 0.000 | 0.000 | 0.000 | 0.000 | 0.000 | 0.000 |
| meta1955 |  | 670.503 | 47.151 | 0.001 | 0.001 | 0.001 | 0.001 | 0.001 | 0.001 | 0.001 | 0.001 | 0.002 | 0.002 | 0.001 | 0.000 | 0.003 | 0.002 | 0.003 |
| meta1956 |  | 670.836 | 318.698 | 0.001 | 0.001 | 0.001 | 0.001 | 0.001 | 0.001 | 0.001 | 0.001 | 0.001 | 0.001 | 0.001 | 0.001 | 0.001 | 0.001 | 0.001 |
| meta1957 |  | 671.378 | 147.584 | 0.001 | 0.001 | 0.001 | 0.000 | 0.001 | 0.000 | 0.001 | 0.001 | 0.001 | 0.001 | 0.000 | 0.001 | 0.001 | 0.000 | 0.000 |
| meta1958 |  | 671.429 | 40.083 | 0.002 | 0.002 | 0.002 | 0.002 | 0.006 | 0.002 | 0.003 | 0.006 | 0.001 | 0.001 | 0.002 | 0.003 | 0.001 | 0.004 | 0.001 |
| meta1959 | 1-Palmitoyl-2-linoleoyl-sn-glycero-3-phosphate | 671.463 | 127.827 | 0.008 | 0.008 | 0.009 | 0.009 | 0.010 | 0.004 | 0.010 | 0.006 | 0.007 | 0.006 | 0.016 | 0.022 | 0.006 | 0.004 | 0.008 |
| meta1960 |  | 672.223 | 164.421 | 0.000 | 0.000 | 0.000 | 0.000 | 0.000 | 0.000 | 0.000 | 0.000 | 0.000 | 0.000 | 0.000 | 0.000 | 0.000 | 0.000 | 0.000 |
| meta1961 |  | 672.270 | 182.980 | 0.003 | 0.003 | 0.003 | 0.001 | 0.002 | 0.005 | 0.005 | 0.003 | 0.001 | 0.004 | 0.004 | 0.002 | 0.004 | 0.005 | 0.002 |
| meta1962 |  | 673.333 | 27.520 | 0.006 | 0.006 | 0.006 | 0.023 | 0.014 | 0.008 | 0.006 | 0.006 | 0.004 | 0.006 | 0.003 | 0.003 | 0.005 | 0.005 | 0.004 |
| meta1963 |  | 673.347 | 165.719 | 0.001 | 0.001 | 0.001 | 0.001 | 0.001 | 0.001 | 0.002 | 0.002 | 0.001 | 0.001 | 0.001 | 0.001 | 0.001 | 0.000 | 0.001 |
| meta1964 |  | 674.149 | 474.871 | 0.000 | 0.000 | 0.000 | 0.000 | 0.000 | 0.000 | 0.000 | 0.001 | 0.001 | 0.001 | 0.001 | 0.001 | 0.001 | 0.000 | 0.000 |
| meta1965 |  | 674.401 | 213.488 | 0.000 | 0.000 | 0.000 | 0.001 | 0.001 | 0.000 | 0.000 | 0.000 | 0.001 | 0.000 | 0.000 | 0.000 | 0.000 | 0.000 | 0.000 |
| meta1966 |  | 675.173 | 425.624 | 0.019 | 0.017 | 0.021 | 0.022 | 0.018 | 0.023 | 0.019 | 0.025 | 0.015 | 0.021 | 0.020 | 0.028 | 0.021 | 0.014 | 0.010 |
| meta1967 |  | 675.335 | 187.872 | 0.001 | 0.001 | 0.001 | 0.000 | 0.000 | 0.000 | 0.001 | 0.001 | 0.001 | 0.000 | 0.002 | 0.002 | 0.003 | 0.000 | 0.001 |
| meta1968 |  | 676.367 | 170.929 | 0.002 | 0.002 | 0.002 | 0.003 | 0.002 | 0.003 | 0.003 | 0.004 | 0.002 | 0.003 | 0.002 | 0.004 | 0.003 | 0.002 | 0.002 |
| meta1969 |  | 677.073 | 97.224 | 0.001 | 0.001 | 0.001 | 0.000 | 0.000 | 0.000 | 0.010 | 0.009 | 0.004 | 0.001 | 0.000 | 0.000 | 0.001 | 0.000 | 0.000 |
| meta1970 |  | 677.087 | 478.291 | 0.001 | 0.001 | 0.001 | 0.001 | 0.001 | 0.001 | 0.001 | 0.001 | 0.001 | 0.001 | 0.001 | 0.001 | 0.001 | 0.001 | 0.001 |
| meta1971 |  | 677.109 | 334.022 | 0.001 | 0.000 | 0.000 | 0.001 | 0.001 | 0.000 | 0.000 | 0.001 | 0.000 | 0.001 | 0.001 | 0.000 | 0.001 | 0.001 | 0.001 |
| meta1972 |  | 677.110 | 442.128 | 0.003 | 0.002 | 0.003 | 0.003 | 0.003 | 0.002 | 0.003 | 0.003 | 0.003 | 0.002 | 0.002 | 0.003 | 0.005 | 0.002 | 0.003 |
| meta1973 |  | 677.351 | 213.127 | 0.001 | 0.001 | 0.001 | 0.000 | 0.000 | 0.000 | 0.003 | 0.002 | 0.002 | 0.001 | 0.001 | 0.002 | 0.003 | 0.001 | 0.001 |
| meta1974 |  | 677.352 | 91.328 | 0.003 | 0.002 | 0.003 | 0.002 | 0.002 | 0.002 | 0.009 | 0.002 | 0.003 | 0.002 | 0.006 | 0.004 | 0.009 | 0.004 | 0.003 |
| meta1975 |  | 678.412 | 174.758 | 0.037 | 0.037 | 0.039 | 0.050 | 0.045 | 0.048 | 0.058 | 0.044 | 0.038 | 0.038 | 0.040 | 0.041 | 0.039 | 0.042 | 0.041 |
| meta1976 |  | 678.600 | 32.928 | 0.000 | 0.000 | 0.000 | 0.000 | 0.000 | 0.000 | 0.000 | 0.000 | 0.000 | 0.000 | 0.000 | 0.000 | 0.000 | 0.000 | 0.000 |
| meta1977 |  | 679.181 | 361.373 | 0.000 | 0.001 | 0.000 | 0.000 | 0.000 | 0.000 | 0.001 | 0.001 | 0.000 | 0.000 | 0.001 | 0.000 | 0.000 | 0.000 | 0.001 |
| meta1978 |  | 679.367 | 103.615 | 0.015 | 0.014 | 0.014 | 0.007 | 0.006 | 0.004 | 0.032 | 0.012 | 0.015 | 0.008 | 0.021 | 0.011 | 0.031 | 0.010 | 0.011 |
| meta1979 |  | 680.119 | 421.351 | 0.001 | 0.000 | 0.001 | 0.001 | 0.001 | 0.000 | 0.001 | 0.001 | 0.001 | 0.001 | 0.000 | 0.001 | 0.001 | 0.001 | 0.000 |
| meta1980 |  | 680.212 | 363.418 | 0.000 | 0.000 | 0.000 | 0.000 | 0.000 | 0.000 | 0.000 | 0.000 | 0.000 | 0.000 | 0.000 | 0.000 | 0.000 | 0.000 | 0.000 |
| meta1981 |  | 680.302 | 169.677 | 0.000 | 0.000 | 0.000 | 0.001 | 0.001 | 0.000 | 0.001 | 0.000 | 0.000 | 0.000 | 0.001 | 0.000 | 0.001 | 0.001 | 0.000 |
| meta1982 |  | 680.398 | 170.901 | 0.026 | 0.024 | 0.026 | 0.029 | 0.037 | 0.023 | 0.033 | 0.035 | 0.016 | 0.024 | 0.029 | 0.036 | 0.025 | 0.017 | 0.035 |
| meta1983 |  | 680.452 | 45.919 | 0.001 | 0.000 | 0.001 | 0.000 | 0.001 | 0.000 | 0.001 | 0.000 | 0.000 | 0.000 | 0.000 | 0.000 | 0.001 | 0.002 | 0.003 |
| meta1984 |  | 681.143 | 433.520 | 0.001 | 0.001 | 0.001 | 0.001 | 0.001 | 0.001 | 0.001 | 0.001 | 0.001 | 0.001 | 0.001 | 0.001 | 0.001 | 0.001 | 0.001 |
| meta1985 |  | 681.206 | 472.113 | 0.003 | 0.002 | 0.002 | 0.003 | 0.002 | 0.003 | 0.002 | 0.003 | 0.002 | 0.003 | 0.002 | 0.004 | 0.002 | 0.002 | 0.001 |
| meta1986 |  | 681.254 | 398.748 | 0.000 | 0.000 | 0.000 | 0.000 | 0.000 | 0.000 | 0.000 | 0.000 | 0.000 | 0.000 | 0.000 | 0.000 | 0.000 | 0.000 | 0.000 |
| meta1987 |  | 681.304 | 45.996 | 0.002 | 0.002 | 0.002 | 0.001 | 0.003 | 0.001 | 0.001 | 0.002 | 0.002 | 0.002 | 0.002 | 0.002 | 0.001 | 0.003 | 0.003 |
| meta1988 |  | 681.320 | 227.852 | 0.001 | 0.001 | 0.001 | 0.001 | 0.001 | 0.001 | 0.002 | 0.001 | 0.001 | 0.001 | 0.001 | 0.001 | 0.001 | 0.001 | 0.001 |
| meta1989 |  | 682.586 | 32.897 | 0.003 | 0.002 | 0.002 | 0.000 | 0.000 | 0.000 | 0.000 | 0.001 | 0.000 | 0.001 | 0.001 | 0.002 | 0.000 | 0.000 | 0.000 |
| meta1990 |  | 683.207 | 26.004 | 0.001 | 0.001 | 0.001 | 0.002 | 0.002 | 0.002 | 0.001 | 0.003 | 0.002 | 0.003 | 0.002 | 0.001 | 0.001 | 0.001 | 0.000 |
| meta1991 |  | 683.222 | 369.070 | 0.012 | 0.010 | 0.011 | 0.011 | 0.015 | 0.022 | 0.009 | 0.013 | 0.006 | 0.011 | 0.011 | 0.020 | 0.009 | 0.009 | 0.003 |
| meta1992 |  | 684.048 | 420.040 | 0.002 | 0.002 | 0.002 | 0.002 | 0.002 | 0.002 | 0.002 | 0.002 | 0.002 | 0.002 | 0.002 | 0.002 | 0.002 | 0.001 | 0.002 |
| meta1993 |  | 684.394 | 142.137 | 0.001 | 0.001 | 0.001 | 0.002 | 0.001 | 0.002 | 0.001 | 0.001 | 0.001 | 0.001 | 0.001 | 0.001 | 0.001 | 0.001 | 0.001 |
| meta1994 |  | 685.108 | 428.696 | 0.002 | 0.001 | 0.001 | 0.002 | 0.001 | 0.001 | 0.001 | 0.002 | 0.002 | 0.001 | 0.002 | 0.002 | 0.002 | 0.001 | 0.002 |
| meta1995 |  | 685.480 | 41.013 | 0.008 | 0.007 | 0.007 | 0.005 | 0.000 | 0.005 | 0.001 | 0.003 | 0.001 | 0.002 | 0.024 | 0.028 | 0.004 | 0.001 | 0.004 |
| meta1996 | Dephosphocoenzyme A (Dephospho-CoA) | 686.139 | 338.272 | 0.002 | 0.002 | 0.002 | 0.002 | 0.002 | 0.002 | 0.003 | 0.002 | 0.002 | 0.002 | 0.001 | 0.002 | 0.002 | 0.002 | 0.002 |
| meta1997 |  | 687.194 | 468.777 | 0.000 | 0.000 | 0.000 | 0.000 | 0.000 | 0.001 | 0.000 | 0.001 | 0.000 | 0.000 | 0.000 | 0.001 | 0.000 | 0.000 | 0.000 |
| meta1998 |  | 687.331 | 207.991 | 0.003 | 0.003 | 0.003 | 0.004 | 0.004 | 0.003 | 0.004 | 0.004 | 0.002 | 0.003 | 0.004 | 0.004 | 0.006 | 0.005 | 0.003 |
| meta1999 |  | 687.348 | 27.067 | 0.004 | 0.003 | 0.004 | 0.014 | 0.009 | 0.006 | 0.003 | 0.003 | 0.002 | 0.003 | 0.002 | 0.003 | 0.004 | 0.003 | 0.003 |
| meta2000 |  | 688.122 | 371.560 | 0.001 | 0.001 | 0.001 | 0.001 | 0.001 | 0.000 | 0.001 | 0.001 | 0.000 | 0.001 | 0.001 | 0.001 | 0.000 | 0.000 | 0.001 |
| meta2001 |  | 688.129 | 25.028 | 0.001 | 0.002 | 0.002 | 0.002 | 0.001 | 0.002 | 0.003 | 0.006 | 0.005 | 0.006 | 0.002 | 0.001 | 0.001 | 0.001 | 0.000 |
| meta2002 |  | 688.200 | 163.732 | 0.000 | 0.000 | 0.000 | 0.001 | 0.001 | 0.000 | 0.000 | 0.000 | 0.000 | 0.000 | 0.000 | 0.000 | 0.000 | 0.000 | 0.000 |
| meta2003 |  | 688.248 | 24.989 | 0.001 | 0.001 | 0.001 | 0.001 | 0.001 | 0.001 | 0.001 | 0.001 | 0.001 | 0.001 | 0.001 | 0.002 | 0.002 | 0.001 | 0.002 |
| meta2004 |  | 688.297 | 136.175 | 0.003 | 0.002 | 0.002 | 0.001 | 0.001 | 0.004 | 0.004 | 0.003 | 0.001 | 0.008 | 0.003 | 0.001 | 0.004 | 0.003 | 0.003 |
| meta2005 |  | 688.330 | 47.416 | 0.003 | 0.003 | 0.002 | 0.002 | 0.002 | 0.001 | 0.003 | 0.001 | 0.003 | 0.003 | 0.004 | 0.002 | 0.007 | 0.002 | 0.005 |
| meta2006 |  | 688.416 | 209.918 | 0.000 | 0.000 | 0.000 | 0.001 | 0.001 | 0.000 | 0.000 | 0.000 | 0.001 | 0.000 | 0.000 | 0.000 | 0.000 | 0.000 | 0.000 |
| meta2007 |  | 688.489 | 134.045 | 0.002 | 0.001 | 0.001 | 0.000 | 0.001 | 0.000 | 0.000 | 0.001 | 0.000 | 0.000 | 0.001 | 0.001 | 0.000 | 0.000 | 0.000 |
| meta2008 |  | 689.273 | 165.680 | 0.000 | 0.000 | 0.000 | 0.000 | 0.000 | 0.000 | 0.000 | 0.005 | 0.000 | 0.002 | 0.001 | 0.000 | 0.001 | 0.000 | 0.000 |
| meta2009 |  | 690.108 | 24.196 | 0.000 | 0.001 | 0.000 | 0.001 | 0.000 | 0.000 | 0.000 | 0.001 | 0.002 | 0.002 | 0.000 | 0.000 | 0.002 | 0.001 | 0.001 |
| meta2010 |  | 691.208 | 369.567 | 0.003 | 0.003 | 0.003 | 0.003 | 0.004 | 0.005 | 0.003 | 0.004 | 0.002 | 0.003 | 0.004 | 0.006 | 0.002 | 0.002 | 0.001 |
| meta2011 |  | 691.209 | 468.703 | 0.000 | 0.000 | 0.001 | 0.000 | 0.000 | 0.001 | 0.001 | 0.001 | 0.000 | 0.001 | 0.001 | 0.001 | 0.001 | 0.000 | 0.000 |
| meta2012 |  | 691.276 | 424.809 | 0.000 | 0.000 | 0.000 | 0.000 | 0.000 | 0.000 | 0.000 | 0.000 | 0.000 | 0.000 | 0.000 | 0.000 | 0.000 | 0.000 | 0.000 |
| meta2013 |  | 692.120 | 22.370 | 0.005 | 0.004 | 0.003 | 0.004 | 0.001 | 0.002 | 0.004 | 0.023 | 0.005 | 0.002 | 0.004 | 0.004 | 0.009 | 0.007 | 0.007 |
| meta2014 |  | 692.750 | 287.985 | 0.001 | 0.001 | 0.001 | 0.002 | 0.002 | 0.001 | 0.001 | 0.002 | 0.002 | 0.001 | 0.001 | 0.001 | 0.002 | 0.001 | 0.001 |
| meta2015 |  | 692.982 | 285.081 | 0.001 | 0.001 | 0.000 | 0.001 | 0.001 | 0.000 | 0.000 | 0.000 | 0.000 | 0.000 | 0.000 | 0.000 | 0.000 | 0.000 | 0.000 |
| meta2016 | deoxyguanosine 5'-monophosphate (dGMP) | 693.116 | 417.535 | 0.010 | 0.009 | 0.009 | 0.009 | 0.008 | 0.008 | 0.009 | 0.014 | 0.013 | 0.013 | 0.012 | 0.012 | 0.015 | 0.009 | 0.012 |
| meta2017 |  | 693.147 | 461.704 | 0.011 | 0.009 | 0.010 | 0.013 | 0.013 | 0.011 | 0.011 | 0.016 | 0.011 | 0.013 | 0.011 | 0.012 | 0.014 | 0.008 | 0.011 |
| meta2018 |  | 693.170 | 344.726 | 0.006 | 0.004 | 0.005 | 0.005 | 0.006 | 0.008 | 0.004 | 0.005 | 0.003 | 0.006 | 0.005 | 0.006 | 0.004 | 0.003 | 0.003 |
| meta2019 |  | 693.207 | 398.088 | 0.002 | 0.001 | 0.002 | 0.002 | 0.002 | 0.002 | 0.001 | 0.002 | 0.001 | 0.002 | 0.002 | 0.002 | 0.001 | 0.001 | 0.001 |
| meta2020 |  | 694.102 | 23.233 | 0.000 | 0.001 | 0.001 | 0.001 | 0.001 | 0.000 | 0.001 | 0.006 | 0.000 | 0.002 | 0.001 | 0.001 | 0.004 | 0.001 | 0.002 |
| meta2021 |  | 694.215 | 164.308 | 0.000 | 0.000 | 0.000 | 0.001 | 0.000 | 0.000 | 0.000 | 0.000 | 0.000 | 0.000 | 0.000 | 0.001 | 0.000 | 0.000 | 0.000 |
| meta2022 |  | 694.378 | 105.360 | 0.004 | 0.004 | 0.006 | 0.003 | 0.002 | 0.001 | 0.017 | 0.005 | 0.007 | 0.004 | 0.009 | 0.005 | 0.008 | 0.003 | 0.005 |
| meta2023 |  | 694.413 | 170.290 | 0.001 | 0.000 | 0.000 | 0.001 | 0.001 | 0.001 | 0.001 | 0.001 | 0.000 | 0.000 | 0.001 | 0.001 | 0.001 | 0.000 | 0.001 |
| meta2024 |  | 694.470 | 45.820 | 0.001 | 0.001 | 0.001 | 0.000 | 0.002 | 0.000 | 0.001 | 0.000 | 0.000 | 0.000 | 0.000 | 0.001 | 0.003 | 0.003 | 0.005 |
| meta2025 |  | 695.186 | 428.016 | 0.003 | 0.003 | 0.003 | 0.004 | 0.003 | 0.004 | 0.003 | 0.004 | 0.002 | 0.003 | 0.003 | 0.003 | 0.002 | 0.002 | 0.002 |
| meta2026 |  | 695.321 | 96.878 | 0.001 | 0.001 | 0.001 | 0.000 | 0.000 | 0.000 | 0.003 | 0.002 | 0.000 | 0.002 | 0.000 | 0.000 | 0.002 | 0.000 | 0.000 |
| meta2027 |  | 695.591 | 32.890 | 0.000 | 0.000 | 0.000 | 0.000 | 0.000 | 0.000 | 0.000 | 0.000 | 0.000 | 0.000 | 0.000 | 0.000 | 0.000 | 0.000 | 0.000 |
| meta2028 |  | 696.358 | 173.776 | 0.001 | 0.001 | 0.001 | 0.002 | 0.001 | 0.001 | 0.002 | 0.001 | 0.001 | 0.001 | 0.001 | 0.001 | 0.001 | 0.001 | 0.001 |
| meta2029 |  | 697.111 | 381.076 | 0.000 | 0.000 | 0.000 | 0.000 | 0.001 | 0.000 | 0.000 | 0.001 | 0.000 | 0.000 | 0.001 | 0.000 | 0.000 | 0.000 | 0.000 |
| meta2030 |  | 697.142 | 481.399 | 0.000 | 0.000 | 0.001 | 0.001 | 0.001 | 0.000 | 0.001 | 0.001 | 0.001 | 0.001 | 0.000 | 0.001 | 0.001 | 0.000 | 0.000 |
| meta2031 |  | 697.152 | 433.303 | 0.001 | 0.001 | 0.001 | 0.001 | 0.001 | 0.001 | 0.001 | 0.001 | 0.001 | 0.001 | 0.001 | 0.001 | 0.001 | 0.000 | 0.001 |
| meta2032 |  | 697.202 | 344.097 | 0.011 | 0.009 | 0.009 | 0.011 | 0.010 | 0.015 | 0.010 | 0.015 | 0.006 | 0.011 | 0.009 | 0.014 | 0.010 | 0.005 | 0.004 |
| meta2033 |  | 697.283 | 132.893 | 0.000 | 0.000 | 0.000 | 0.001 | 0.001 | 0.001 | 0.001 | 0.000 | 0.000 | 0.001 | 0.000 | 0.000 | 0.001 | 0.000 | 0.000 |
| meta2034 |  | 697.338 | 243.189 | 0.001 | 0.001 | 0.001 | 0.000 | 0.001 | 0.001 | 0.002 | 0.001 | 0.001 | 0.001 | 0.001 | 0.001 | 0.001 | 0.001 | 0.000 |
| meta2035 |  | 697.352 | 203.949 | 0.001 | 0.001 | 0.001 | 0.001 | 0.001 | 0.001 | 0.001 | 0.000 | 0.001 | 0.000 | 0.000 | 0.000 | 0.001 | 0.001 | 0.000 |
| meta2036 |  | 697.478 | 128.448 | 0.012 | 0.020 | 0.018 | 0.010 | 0.007 | 0.009 | 0.010 | 0.006 | 0.007 | 0.018 | 0.017 | 0.010 | 0.007 | 0.006 | 0.006 |
| meta2037 |  | 697.966 | 86.074 | 0.004 | 0.003 | 0.004 | 0.006 | 0.006 | 0.004 | 0.005 | 0.003 | 0.003 | 0.003 | 0.004 | 0.001 | 0.004 | 0.004 | 0.005 |
| meta2038 |  | 698.555 | 49.341 | 0.002 | 0.002 | 0.002 | 0.000 | 0.000 | 0.000 | 0.000 | 0.000 | 0.000 | 0.000 | 0.001 | 0.001 | 0.001 | 0.000 | 0.000 |
| meta2039 |  | 699.054 | 97.484 | 0.000 | 0.001 | 0.001 | 0.000 | 0.000 | 0.000 | 0.007 | 0.004 | 0.002 | 0.001 | 0.000 | 0.000 | 0.001 | 0.000 | 0.000 |
| meta2040 |  | 699.090 | 334.340 | 0.001 | 0.001 | 0.001 | 0.001 | 0.001 | 0.001 | 0.001 | 0.001 | 0.001 | 0.001 | 0.001 | 0.001 | 0.001 | 0.001 | 0.001 |
| meta2041 |  | 699.173 | 404.891 | 0.001 | 0.001 | 0.001 | 0.001 | 0.001 | 0.002 | 0.001 | 0.001 | 0.001 | 0.001 | 0.001 | 0.002 | 0.001 | 0.001 | 0.001 |
| meta2042 |  | 699.218 | 427.876 | 0.001 | 0.001 | 0.001 | 0.001 | 0.001 | 0.001 | 0.001 | 0.001 | 0.000 | 0.001 | 0.001 | 0.001 | 0.001 | 0.000 | 0.000 |
| meta2043 |  | 700.366 | 170.290 | 0.005 | 0.005 | 0.005 | 0.007 | 0.009 | 0.005 | 0.007 | 0.008 | 0.005 | 0.005 | 0.007 | 0.010 | 0.006 | 0.005 | 0.008 |
| meta2044 |  | 700.396 | 174.088 | 0.000 | 0.001 | 0.001 | 0.001 | 0.001 | 0.001 | 0.001 | 0.002 | 0.001 | 0.001 | 0.001 | 0.000 | 0.000 | 0.001 | 0.001 |
| meta2045 |  | 701.150 | 358.202 | 0.001 | 0.001 | 0.001 | 0.001 | 0.001 | 0.001 | 0.001 | 0.001 | 0.001 | 0.001 | 0.001 | 0.001 | 0.001 | 0.001 | 0.001 |
| meta2046 |  | 701.188 | 468.881 | 0.001 | 0.000 | 0.000 | 0.000 | 0.000 | 0.001 | 0.000 | 0.000 | 0.000 | 0.001 | 0.001 | 0.001 | 0.000 | 0.000 | 0.000 |
| meta2047 |  | 701.289 | 220.130 | 0.001 | 0.001 | 0.001 | 0.002 | 0.002 | 0.001 | 0.002 | 0.001 | 0.001 | 0.001 | 0.001 | 0.001 | 0.001 | 0.001 | 0.002 |
| meta2048 |  | 701.363 | 26.958 | 0.001 | 0.001 | 0.001 | 0.006 | 0.004 | 0.002 | 0.001 | 0.003 | 0.001 | 0.001 | 0.001 | 0.001 | 0.003 | 0.002 | 0.002 |
| meta2049 |  | 701.532 | 31.540 | 0.002 | 0.002 | 0.002 | 0.001 | 0.001 | 0.001 | 0.001 | 0.002 | 0.000 | 0.000 | 0.002 | 0.002 | 0.001 | 0.002 | 0.001 |
| meta2050 |  | 702.412 | 173.542 | 0.003 | 0.003 | 0.003 | 0.004 | 0.003 | 0.004 | 0.004 | 0.006 | 0.006 | 0.003 | 0.004 | 0.004 | 0.005 | 0.006 | 0.005 |
| meta2051 |  | 703.167 | 427.984 | 0.003 | 0.002 | 0.002 | 0.002 | 0.005 | 0.002 | 0.007 | 0.002 | 0.002 | 0.002 | 0.001 | 0.003 | 0.004 | 0.001 | 0.001 |
| meta2052 |  | 704.329 | 46.614 | 0.001 | 0.001 | 0.001 | 0.001 | 0.001 | 0.001 | 0.001 | 0.001 | 0.001 | 0.003 | 0.001 | 0.000 | 0.002 | 0.001 | 0.001 |
| meta2053 |  | 704.409 | 144.185 | 0.000 | 0.000 | 0.000 | 0.001 | 0.001 | 0.001 | 0.000 | 0.000 | 0.000 | 0.000 | 0.000 | 0.000 | 0.000 | 0.000 | 0.000 |
| meta2054 |  | 704.616 | 32.897 | 0.001 | 0.001 | 0.001 | 0.000 | 0.000 | 0.000 | 0.000 | 0.000 | 0.000 | 0.000 | 0.001 | 0.001 | 0.000 | 0.000 | 0.000 |
| meta2055 |  | 705.306 | 136.956 | 0.001 | 0.000 | 0.000 | 0.000 | 0.000 | 0.001 | 0.001 | 0.001 | 0.000 | 0.001 | 0.001 | 0.000 | 0.001 | 0.001 | 0.001 |
| meta2056 |  | 706.012 | 88.152 | 0.001 | 0.001 | 0.001 | 0.002 | 0.003 | 0.002 | 0.001 | 0.001 | 0.001 | 0.001 | 0.002 | 0.000 | 0.001 | 0.002 | 0.003 |
| meta2057 |  | 706.107 | 432.763 | 0.002 | 0.001 | 0.001 | 0.001 | 0.001 | 0.001 | 0.001 | 0.002 | 0.003 | 0.002 | 0.002 | 0.002 | 0.002 | 0.001 | 0.001 |
| meta2058 |  | 706.282 | 46.289 | 0.000 | 0.000 | 0.000 | 0.000 | 0.000 | 0.000 | 0.001 | 0.000 | 0.001 | 0.001 | 0.001 | 0.000 | 0.001 | 0.001 | 0.001 |
| meta2059 |  | 706.469 | 45.241 | 0.000 | 0.000 | 0.000 | 0.000 | 0.001 | 0.000 | 0.001 | 0.001 | 0.000 | 0.000 | 0.000 | 0.001 | 0.001 | 0.002 | 0.003 |
| meta2060 |  | 707.095 | 420.758 | 0.001 | 0.001 | 0.001 | 0.001 | 0.001 | 0.001 | 0.001 | 0.001 | 0.002 | 0.001 | 0.001 | 0.001 | 0.001 | 0.001 | 0.001 |
| meta2061 |  | 707.591 | 32.894 | 0.001 | 0.001 | 0.001 | 0.000 | 0.000 | 0.000 | 0.000 | 0.000 | 0.000 | 0.000 | 0.000 | 0.001 | 0.000 | 0.000 | 0.000 |
| meta2062 |  | 707.635 | 32.903 | 0.001 | 0.001 | 0.001 | 0.000 | 0.000 | 0.000 | 0.000 | 0.000 | 0.000 | 0.000 | 0.001 | 0.001 | 0.000 | 0.000 | 0.000 |
| meta2063 |  | 708.322 | 46.675 | 0.000 | 0.000 | 0.000 | 0.000 | 0.001 | 0.000 | 0.000 | 0.000 | 0.001 | 0.001 | 0.000 | 0.000 | 0.000 | 0.000 | 0.000 |
| meta2064 |  | 708.442 | 141.880 | 0.004 | 0.004 | 0.004 | 0.009 | 0.008 | 0.005 | 0.003 | 0.003 | 0.004 | 0.003 | 0.003 | 0.002 | 0.003 | 0.003 | 0.003 |
| meta2065 |  | 708.485 | 45.935 | 0.011 | 0.010 | 0.012 | 0.005 | 0.027 | 0.004 | 0.007 | 0.003 | 0.003 | 0.001 | 0.002 | 0.012 | 0.040 | 0.045 | 0.074 |
| meta2066 |  | 709.107 | 421.271 | 0.012 | 0.011 | 0.010 | 0.013 | 0.011 | 0.007 | 0.011 | 0.013 | 0.013 | 0.012 | 0.012 | 0.010 | 0.013 | 0.010 | 0.011 |
| meta2067 |  | 709.118 | 478.322 | 0.001 | 0.001 | 0.001 | 0.001 | 0.001 | 0.002 | 0.001 | 0.002 | 0.002 | 0.002 | 0.001 | 0.001 | 0.002 | 0.001 | 0.001 |
| meta2068 |  | 709.481 | 40.048 | 0.005 | 0.003 | 0.004 | 0.003 | 0.013 | 0.004 | 0.004 | 0.002 | 0.001 | 0.002 | 0.019 | 0.016 | 0.019 | 0.001 | 0.033 |
| meta2069 |  | 710.046 | 420.347 | 0.003 | 0.002 | 0.002 | 0.003 | 0.003 | 0.002 | 0.003 | 0.003 | 0.002 | 0.002 | 0.002 | 0.003 | 0.002 | 0.002 | 0.003 |
| meta2070 |  | 710.280 | 136.928 | 0.002 | 0.001 | 0.001 | 0.001 | 0.001 | 0.002 | 0.003 | 0.002 | 0.001 | 0.005 | 0.002 | 0.001 | 0.002 | 0.002 | 0.002 |
| meta2071 |  | 710.323 | 169.074 | 0.000 | 0.000 | 0.000 | 0.000 | 0.000 | 0.000 | 0.000 | 0.000 | 0.000 | 0.000 | 0.000 | 0.000 | 0.000 | 0.000 | 0.000 |
| meta2072 |  | 710.487 | 42.936 | 0.002 | 0.002 | 0.002 | 0.001 | 0.004 | 0.003 | 0.001 | 0.001 | 0.001 | 0.001 | 0.008 | 0.009 | 0.006 | 0.007 | 0.011 |
| meta2073 |  | 711.350 | 45.946 | 0.001 | 0.000 | 0.000 | 0.001 | 0.000 | 0.000 | 0.001 | 0.001 | 0.000 | 0.001 | 0.001 | 0.000 | 0.000 | 0.001 | 0.000 |
| meta2074 |  | 712.106 | 418.721 | 0.001 | 0.001 | 0.001 | 0.001 | 0.001 | 0.000 | 0.001 | 0.001 | 0.001 | 0.001 | 0.001 | 0.001 | 0.001 | 0.001 | 0.001 |
| meta2075 |  | 712.194 | 163.715 | 0.000 | 0.000 | 0.000 | 0.001 | 0.001 | 0.000 | 0.000 | 0.000 | 0.000 | 0.000 | 0.000 | 0.000 | 0.000 | 0.000 | 0.000 |
| meta2076 |  | 712.333 | 174.478 | 0.002 | 0.002 | 0.002 | 0.003 | 0.003 | 0.002 | 0.002 | 0.003 | 0.002 | 0.002 | 0.002 | 0.002 | 0.002 | 0.002 | 0.002 |
| meta2077 |  | 712.834 | 286.389 | 0.001 | 0.001 | 0.000 | 0.001 | 0.000 | 0.000 | 0.001 | 0.001 | 0.000 | 0.001 | 0.001 | 0.001 | 0.001 | 0.000 | 0.000 |
| meta2078 |  | 713.079 | 381.074 | 0.000 | 0.000 | 0.000 | 0.000 | 0.000 | 0.000 | 0.000 | 0.001 | 0.000 | 0.000 | 0.001 | 0.000 | 0.000 | 0.000 | 0.000 |
| meta2079 |  | 713.196 | 428.180 | 0.002 | 0.002 | 0.002 | 0.002 | 0.002 | 0.002 | 0.002 | 0.002 | 0.001 | 0.002 | 0.001 | 0.002 | 0.001 | 0.001 | 0.001 |
| meta2080 |  | 713.939 | 86.388 | 0.002 | 0.002 | 0.002 | 0.003 | 0.003 | 0.002 | 0.003 | 0.001 | 0.002 | 0.001 | 0.002 | 0.001 | 0.002 | 0.002 | 0.002 |
| meta2081 |  | 714.383 | 155.004 | 0.000 | 0.000 | 0.000 | 0.001 | 0.001 | 0.000 | 0.000 | 0.000 | 0.000 | 0.000 | 0.000 | 0.000 | 0.000 | 0.000 | 0.000 |
| meta2082 |  | 715.098 | 417.004 | 0.003 | 0.003 | 0.003 | 0.003 | 0.003 | 0.003 | 0.003 | 0.004 | 0.003 | 0.003 | 0.004 | 0.003 | 0.003 | 0.003 | 0.003 |
| meta2083 |  | 716.198 | 371.155 | 0.001 | 0.001 | 0.001 | 0.001 | 0.001 | 0.001 | 0.001 | 0.001 | 0.000 | 0.001 | 0.001 | 0.001 | 0.001 | 0.001 | 0.001 |
| meta2084 |  | 716.329 | 47.199 | 0.000 | 0.000 | 0.000 | 0.000 | 0.000 | 0.000 | 0.001 | 0.000 | 0.000 | 0.000 | 0.001 | 0.001 | 0.001 | 0.000 | 0.000 |
| meta2085 |  | 716.374 | 170.961 | 0.001 | 0.000 | 0.001 | 0.001 | 0.001 | 0.000 | 0.000 | 0.001 | 0.000 | 0.000 | 0.000 | 0.001 | 0.001 | 0.000 | 0.000 |
| meta2086 |  | 717.144 | 391.552 | 0.002 | 0.002 | 0.002 | 0.002 | 0.002 | 0.001 | 0.002 | 0.001 | 0.001 | 0.001 | 0.002 | 0.001 | 0.001 | 0.001 | 0.001 |
| meta2087 |  | 717.469 | 45.263 | 0.001 | 0.002 | 0.002 | 0.003 | 0.002 | 0.002 | 0.003 | 0.003 | 0.002 | 0.002 | 0.002 | 0.002 | 0.001 | 0.002 | 0.001 |
| meta2088 |  | 718.118 | 419.778 | 0.001 | 0.000 | 0.000 | 0.001 | 0.000 | 0.000 | 0.001 | 0.001 | 0.001 | 0.000 | 0.001 | 0.001 | 0.001 | 0.000 | 0.000 |
| meta2089 |  | 718.249 | 339.479 | 0.000 | 0.000 | 0.000 | 0.000 | 0.000 | 0.001 | 0.000 | 0.000 | 0.000 | 0.000 | 0.000 | 0.000 | 0.000 | 0.000 | 0.000 |
| meta2090 |  | 719.093 | 380.983 | 0.000 | 0.000 | 0.000 | 0.001 | 0.000 | 0.000 | 0.000 | 0.001 | 0.001 | 0.000 | 0.001 | 0.001 | 0.000 | 0.000 | 0.000 |
| meta2091 |  | 719.428 | 208.001 | 0.000 | 0.000 | 0.000 | 0.000 | 0.000 | 0.000 | 0.000 | 0.000 | 0.000 | 0.000 | 0.000 | 0.000 | 0.000 | 0.000 | 0.000 |
| meta2092 |  | 719.483 | 46.589 | 0.006 | 0.005 | 0.005 | 0.005 | 0.004 | 0.003 | 0.003 | 0.009 | 0.004 | 0.005 | 0.005 | 0.005 | 0.002 | 0.004 | 0.003 |
| meta2093 |  | 721.039 | 97.484 | 0.001 | 0.001 | 0.001 | 0.000 | 0.000 | 0.000 | 0.010 | 0.004 | 0.002 | 0.001 | 0.000 | 0.000 | 0.001 | 0.000 | 0.000 |
| meta2094 |  | 721.123 | 427.881 | 0.003 | 0.002 | 0.002 | 0.003 | 0.002 | 0.002 | 0.002 | 0.003 | 0.002 | 0.003 | 0.003 | 0.003 | 0.002 | 0.002 | 0.001 |
| meta2095 |  | 721.367 | 45.241 | 0.000 | 0.000 | 0.000 | 0.001 | 0.000 | 0.000 | 0.001 | 0.000 | 0.000 | 0.000 | 0.000 | 0.000 | 0.001 | 0.001 | 0.000 |
| meta2096 |  | 722.273 | 47.278 | 0.000 | 0.001 | 0.000 | 0.000 | 0.000 | 0.000 | 0.001 | 0.001 | 0.001 | 0.000 | 0.001 | 0.001 | 0.002 | 0.001 | 0.000 |
| meta2097 |  | 723.158 | 436.133 | 0.002 | 0.002 | 0.002 | 0.002 | 0.002 | 0.001 | 0.002 | 0.003 | 0.002 | 0.002 | 0.002 | 0.002 | 0.003 | 0.002 | 0.002 |
| meta2098 |  | 723.217 | 405.917 | 0.014 | 0.013 | 0.014 | 0.014 | 0.013 | 0.020 | 0.013 | 0.016 | 0.011 | 0.013 | 0.014 | 0.019 | 0.014 | 0.009 | 0.007 |
| meta2099 |  | 724.366 | 169.622 | 0.002 | 0.002 | 0.002 | 0.002 | 0.003 | 0.002 | 0.002 | 0.003 | 0.002 | 0.002 | 0.002 | 0.003 | 0.002 | 0.002 | 0.002 |
| meta2100 |  | 725.067 | 474.706 | 0.003 | 0.002 | 0.002 | 0.003 | 0.003 | 0.002 | 0.002 | 0.003 | 0.002 | 0.003 | 0.003 | 0.003 | 0.003 | 0.002 | 0.002 |
| meta2101 |  | 725.227 | 365.261 | 0.002 | 0.002 | 0.002 | 0.002 | 0.003 | 0.002 | 0.002 | 0.002 | 0.001 | 0.002 | 0.002 | 0.002 | 0.002 | 0.002 | 0.000 |
| meta2102 |  | 725.233 | 468.881 | 0.005 | 0.004 | 0.005 | 0.004 | 0.004 | 0.007 | 0.005 | 0.006 | 0.004 | 0.006 | 0.004 | 0.006 | 0.005 | 0.003 | 0.002 |
| meta2103 |  | 726.192 | 404.872 | 0.037 | 0.033 | 0.032 | 0.037 | 0.030 | 0.046 | 0.029 | 0.041 | 0.023 | 0.033 | 0.036 | 0.044 | 0.033 | 0.019 | 0.018 |
| meta2104 |  | 726.209 | 475.706 | 0.000 | 0.000 | 0.000 | 0.001 | 0.001 | 0.000 | 0.001 | 0.000 | 0.000 | 0.000 | 0.001 | 0.001 | 0.001 | 0.001 | 0.001 |
| meta2105 |  | 726.742 | 287.380 | 0.001 | 0.001 | 0.001 | 0.002 | 0.002 | 0.001 | 0.001 | 0.002 | 0.002 | 0.001 | 0.001 | 0.002 | 0.002 | 0.001 | 0.001 |
| meta2106 |  | 728.208 | 468.956 | 0.010 | 0.008 | 0.008 | 0.008 | 0.007 | 0.010 | 0.008 | 0.011 | 0.007 | 0.010 | 0.012 | 0.011 | 0.006 | 0.004 | 0.005 |
| meta2107 |  | 730.309 | 46.609 | 0.000 | 0.000 | 0.000 | 0.000 | 0.000 | 0.000 | 0.000 | 0.000 | 0.000 | 0.000 | 0.000 | 0.000 | 0.000 | 0.000 | 0.000 |
| meta2108 |  | 730.704 | 288.020 | 0.002 | 0.001 | 0.001 | 0.002 | 0.001 | 0.001 | 0.002 | 0.002 | 0.002 | 0.002 | 0.002 | 0.002 | 0.002 | 0.002 | 0.002 |
| meta2109 |  | 731.100 | 478.195 | 0.001 | 0.001 | 0.001 | 0.001 | 0.001 | 0.001 | 0.001 | 0.002 | 0.002 | 0.001 | 0.001 | 0.001 | 0.002 | 0.001 | 0.001 |
| meta2110 |  | 731.162 | 428.628 | 0.001 | 0.001 | 0.001 | 0.001 | 0.001 | 0.001 | 0.001 | 0.001 | 0.001 | 0.001 | 0.001 | 0.001 | 0.001 | 0.001 | 0.001 |
| meta2111 |  | 731.215 | 351.585 | 0.001 | 0.001 | 0.001 | 0.001 | 0.001 | 0.001 | 0.001 | 0.001 | 0.001 | 0.001 | 0.001 | 0.001 | 0.001 | 0.001 | 0.001 |
| meta2112 |  | 731.472 | 264.994 | 0.000 | 0.000 | 0.000 | 0.000 | 0.000 | 0.000 | 0.000 | 0.000 | 0.000 | 0.000 | 0.000 | 0.001 | 0.003 | 0.001 | 0.002 |
| meta2113 |  | 732.686 | 288.355 | 0.002 | 0.001 | 0.002 | 0.002 | 0.002 | 0.001 | 0.002 | 0.001 | 0.001 | 0.002 | 0.002 | 0.002 | 0.001 | 0.002 | 0.002 |
| meta2114 |  | 733.478 | 38.799 | 0.003 | 0.002 | 0.002 | 0.004 | 0.003 | 0.004 | 0.001 | 0.002 | 0.000 | 0.001 | 0.017 | 0.012 | 0.002 | 0.001 | 0.004 |
| meta2115 |  | 734.324 | 45.974 | 0.000 | 0.000 | 0.001 | 0.001 | 0.000 | 0.000 | 0.000 | 0.001 | 0.001 | 0.001 | 0.001 | 0.000 | 0.000 | 0.000 | 0.000 |
| meta2116 | PS(16:0/16:0) | 734.501 | 45.323 | 0.009 | 0.007 | 0.008 | 0.012 | 0.013 | 0.008 | 0.021 | 0.011 | 0.008 | 0.008 | 0.011 | 0.007 | 0.016 | 0.014 | 0.028 |
| meta2117 |  | 735.062 | 381.074 | 0.000 | 0.000 | 0.000 | 0.000 | 0.000 | 0.000 | 0.000 | 0.000 | 0.000 | 0.000 | 0.000 | 0.000 | 0.000 | 0.000 | 0.000 |
| meta2118 |  | 735.134 | 420.274 | 0.011 | 0.009 | 0.007 | 0.011 | 0.010 | 0.006 | 0.009 | 0.012 | 0.010 | 0.011 | 0.011 | 0.011 | 0.012 | 0.008 | 0.008 |
| meta2119 |  | 735.390 | 45.861 | 0.000 | 0.000 | 0.000 | 0.000 | 0.000 | 0.000 | 0.000 | 0.000 | 0.000 | 0.000 | 0.000 | 0.000 | 0.000 | 0.000 | 0.000 |
| meta2120 |  | 735.421 | 213.237 | 0.000 | 0.000 | 0.000 | 0.000 | 0.000 | 0.000 | 0.000 | 0.000 | 0.000 | 0.000 | 0.000 | 0.000 | 0.000 | 0.000 | 0.001 |
| meta2121 |  | 735.518 | 160.597 | 0.001 | 0.001 | 0.001 | 0.001 | 0.001 | 0.001 | 0.001 | 0.000 | 0.001 | 0.000 | 0.001 | 0.001 | 0.001 | 0.000 | 0.000 |
| meta2122 |  | 736.336 | 168.337 | 0.001 | 0.001 | 0.001 | 0.001 | 0.001 | 0.001 | 0.001 | 0.001 | 0.001 | 0.001 | 0.001 | 0.001 | 0.001 | 0.001 | 0.001 |
| meta2123 |  | 736.370 | 174.478 | 0.000 | 0.000 | 0.000 | 0.000 | 0.000 | 0.001 | 0.000 | 0.000 | 0.001 | 0.001 | 0.001 | 0.000 | 0.001 | 0.001 | 0.001 |
| meta2124 |  | 736.489 | 119.755 | 0.045 | 0.043 | 0.039 | 0.024 | 0.027 | 0.015 | 0.012 | 0.035 | 0.018 | 0.032 | 0.033 | 0.035 | 0.027 | 0.029 | 0.026 |
| meta2125 |  | 736.518 | 45.908 | 0.381 | 0.388 | 0.395 | 0.561 | 0.879 | 0.355 | 0.573 | 0.557 | 0.520 | 0.231 | 0.122 | 0.175 | 0.844 | 0.725 | 1.644 |
| meta2126 |  | 736.648 | 288.836 | 0.001 | 0.001 | 0.001 | 0.001 | 0.001 | 0.001 | 0.001 | 0.001 | 0.001 | 0.001 | 0.001 | 0.001 | 0.001 | 0.001 | 0.001 |
| meta2127 |  | 737.534 | 161.234 | 0.012 | 0.010 | 0.011 | 0.003 | 0.003 | 0.003 | 0.002 | 0.003 | 0.004 | 0.002 | 0.007 | 0.008 | 0.005 | 0.002 | 0.003 |
| meta2128 |  | 738.355 | 170.961 | 0.001 | 0.001 | 0.001 | 0.001 | 0.001 | 0.001 | 0.001 | 0.001 | 0.001 | 0.001 | 0.001 | 0.001 | 0.001 | 0.001 | 0.001 |
| meta2129 |  | 738.505 | 119.846 | 0.274 | 0.248 | 0.148 | 0.098 | 0.032 | 0.037 | 0.039 | 0.112 | 0.075 | 0.103 | 0.120 | 0.184 | 0.087 | 0.038 | 0.078 |
| meta2130 |  | 739.066 | 417.907 | 0.000 | 0.000 | 0.000 | 0.000 | 0.000 | 0.000 | 0.000 | 0.000 | 0.000 | 0.000 | 0.000 | 0.000 | 0.000 | 0.000 | 0.000 |
| meta2131 |  | 739.343 | 47.307 | 0.001 | 0.001 | 0.001 | 0.000 | 0.000 | 0.000 | 0.001 | 0.004 | 0.001 | 0.001 | 0.008 | 0.006 | 0.002 | 0.000 | 0.001 |
| meta2132 |  | 740.232 | 363.402 | 0.000 | 0.000 | 0.000 | 0.000 | 0.000 | 0.001 | 0.000 | 0.000 | 0.000 | 0.000 | 0.000 | 0.000 | 0.000 | 0.000 | 0.000 |
| meta2133 |  | 740.519 | 126.881 | 0.034 | 0.030 | 0.038 | 0.013 | 0.011 | 0.009 | 0.018 | 0.021 | 0.016 | 0.019 | 0.058 | 0.038 | 0.023 | 0.011 | 0.026 |
| meta2134 |  | 741.167 | 478.353 | 0.001 | 0.001 | 0.001 | 0.002 | 0.002 | 0.001 | 0.002 | 0.002 | 0.002 | 0.002 | 0.002 | 0.002 | 0.002 | 0.001 | 0.001 |
| meta2135 |  | 741.202 | 398.073 | 0.005 | 0.004 | 0.004 | 0.004 | 0.004 | 0.005 | 0.003 | 0.005 | 0.002 | 0.004 | 0.004 | 0.005 | 0.004 | 0.002 | 0.002 |
| meta2136 | Nicotinamide adenine dinucleotide phosphate (NADP) | 742.065 | 480.110 | 0.002 | 0.002 | 0.002 | 0.002 | 0.002 | 0.002 | 0.002 | 0.002 | 0.003 | 0.002 | 0.002 | 0.002 | 0.005 | 0.003 | 0.003 |
| meta2137 |  | 742.164 | 312.990 | 0.000 | 0.000 | 0.000 | 0.000 | 0.000 | 0.000 | 0.000 | 0.000 | 0.000 | 0.000 | 0.000 | 0.000 | 0.000 | 0.000 | 0.000 |
| meta2138 |  | 742.381 | 212.016 | 0.000 | 0.000 | 0.000 | 0.000 | 0.000 | 0.000 | 0.000 | 0.000 | 0.000 | 0.000 | 0.000 | 0.000 | 0.000 | 0.000 | 0.000 |
| meta2139 |  | 743.402 | 350.631 | 0.000 | 0.000 | 0.000 | 0.000 | 0.000 | 0.000 | 0.000 | 0.000 | 0.000 | 0.000 | 0.000 | 0.000 | 0.000 | 0.000 | 0.000 |
| meta2140 |  | 743.484 | 44.488 | 0.020 | 0.018 | 0.014 | 0.024 | 0.027 | 0.020 | 0.032 | 0.031 | 0.020 | 0.026 | 0.022 | 0.028 | 0.025 | 0.031 | 0.021 |
| meta2141 |  | 744.325 | 46.053 | 0.000 | 0.000 | 0.000 | 0.000 | 0.000 | 0.000 | 0.000 | 0.000 | 0.000 | 0.001 | 0.000 | 0.000 | 0.000 | 0.000 | 0.000 |
| meta2142 |  | 745.199 | 405.512 | 0.002 | 0.001 | 0.002 | 0.002 | 0.002 | 0.002 | 0.002 | 0.002 | 0.001 | 0.002 | 0.002 | 0.002 | 0.002 | 0.001 | 0.001 |
| meta2143 |  | 745.204 | 46.024 | 0.001 | 0.001 | 0.001 | 0.000 | 0.000 | 0.000 | 0.000 | 0.001 | 0.000 | 0.001 | 0.001 | 0.001 | 0.001 | 0.000 | 0.001 |
| meta2144 |  | 745.295 | 137.330 | 0.001 | 0.000 | 0.001 | 0.000 | 0.001 | 0.001 | 0.001 | 0.001 | 0.000 | 0.002 | 0.001 | 0.000 | 0.001 | 0.001 | 0.001 |
| meta2145 |  | 745.501 | 46.010 | 0.174 | 0.154 | 0.151 | 0.143 | 0.093 | 0.076 | 0.087 | 0.177 | 0.246 | 0.155 | 0.212 | 0.231 | 0.114 | 0.123 | 0.098 |
| meta2146 |  | 745.500 | 83.312 | 0.010 | 0.013 | 0.016 | 0.010 | 0.008 | 0.007 | 0.010 | 0.013 | 0.016 | 0.009 | 0.012 | 0.018 | 0.009 | 0.011 | 0.010 |
| meta2147 |  | 747.023 | 97.341 | 0.001 | 0.001 | 0.001 | 0.000 | 0.000 | 0.000 | 0.006 | 0.001 | 0.001 | 0.000 | 0.000 | 0.000 | 0.001 | 0.000 | 0.001 |
| meta2148 |  | 747.214 | 468.777 | 0.001 | 0.001 | 0.001 | 0.001 | 0.001 | 0.001 | 0.001 | 0.001 | 0.001 | 0.001 | 0.001 | 0.001 | 0.001 | 0.001 | 0.001 |
| meta2149 |  | 747.265 | 445.741 | 0.000 | 0.000 | 0.001 | 0.001 | 0.000 | 0.000 | 0.001 | 0.001 | 0.000 | 0.001 | 0.001 | 0.001 | 0.001 | 0.000 | 0.000 |
| meta2150 | 1-Palmitoyl-2-oleoyl-phosphatidylglycerol | 747.517 | 47.247 | 0.397 | 0.362 | 0.330 | 0.177 | 0.111 | 0.103 | 0.124 | 0.229 | 0.159 | 0.190 | 0.297 | 0.451 | 0.162 | 0.121 | 0.173 |
| meta2151 |  | 749.078 | 273.874 | 0.013 | 0.011 | 0.010 | 0.015 | 0.014 | 0.012 | 0.009 | 0.017 | 0.010 | 0.018 | 0.010 | 0.006 | 0.010 | 0.011 | 0.010 |
| meta2152 |  | 750.406 | 94.664 | 0.001 | 0.001 | 0.001 | 0.000 | 0.001 | 0.000 | 0.002 | 0.001 | 0.001 | 0.000 | 0.001 | 0.001 | 0.001 | 0.000 | 0.000 |
| meta2153 |  | 753.083 | 417.472 | 0.001 | 0.001 | 0.001 | 0.001 | 0.001 | 0.001 | 0.001 | 0.002 | 0.001 | 0.002 | 0.001 | 0.001 | 0.001 | 0.001 | 0.001 |
| meta2154 |  | 753.081 | 478.291 | 0.001 | 0.001 | 0.001 | 0.001 | 0.001 | 0.001 | 0.001 | 0.001 | 0.001 | 0.001 | 0.001 | 0.001 | 0.001 | 0.001 | 0.001 |
| meta2155 |  | 753.150 | 425.613 | 0.004 | 0.003 | 0.004 | 0.004 | 0.004 | 0.004 | 0.003 | 0.005 | 0.003 | 0.004 | 0.004 | 0.006 | 0.004 | 0.003 | 0.002 |
| meta2156 |  | 753.168 | 478.353 | 0.004 | 0.003 | 0.004 | 0.005 | 0.005 | 0.004 | 0.004 | 0.007 | 0.005 | 0.005 | 0.004 | 0.005 | 0.006 | 0.004 | 0.004 |
| meta2157 |  | 753.179 | 150.806 | 0.002 | 0.002 | 0.002 | 0.002 | 0.004 | 0.002 | 0.007 | 0.002 | 0.000 | 0.000 | 0.001 | 0.002 | 0.001 | 0.001 | 0.001 |
| meta2158 |  | 755.207 | 468.881 | 0.001 | 0.000 | 0.001 | 0.001 | 0.001 | 0.001 | 0.001 | 0.001 | 0.001 | 0.001 | 0.000 | 0.001 | 0.001 | 0.000 | 0.000 |
| meta2159 |  | 757.076 | 481.793 | 0.001 | 0.001 | 0.001 | 0.001 | 0.001 | 0.001 | 0.001 | 0.001 | 0.001 | 0.001 | 0.001 | 0.001 | 0.002 | 0.001 | 0.001 |
| meta2160 |  | 757.116 | 420.569 | 0.001 | 0.001 | 0.001 | 0.001 | 0.001 | 0.001 | 0.001 | 0.001 | 0.001 | 0.001 | 0.001 | 0.001 | 0.001 | 0.001 | 0.001 |
| meta2161 |  | 757.305 | 227.680 | 0.000 | 0.000 | 0.000 | 0.000 | 0.000 | 0.000 | 0.000 | 0.000 | 0.000 | 0.000 | 0.000 | 0.000 | 0.000 | 0.000 | 0.000 |
| meta2162 |  | 758.576 | 49.295 | 0.000 | 0.000 | 0.000 | 0.000 | 0.000 | 0.000 | 0.000 | 0.000 | 0.000 | 0.000 | 0.000 | 0.000 | 0.000 | 0.000 | 0.000 |
| meta2163 |  | 758.952 | 85.967 | 0.001 | 0.001 | 0.001 | 0.002 | 0.002 | 0.001 | 0.001 | 0.001 | 0.001 | 0.001 | 0.001 | 0.000 | 0.001 | 0.001 | 0.001 |
| meta2164 |  | 759.563 | 166.350 | 0.001 | 0.001 | 0.001 | 0.001 | 0.001 | 0.001 | 0.000 | 0.000 | 0.000 | 0.000 | 0.001 | 0.001 | 0.000 | 0.000 | 0.001 |
| meta2165 |  | 760.415 | 173.642 | 0.001 | 0.001 | 0.001 | 0.001 | 0.001 | 0.001 | 0.001 | 0.001 | 0.001 | 0.001 | 0.001 | 0.001 | 0.001 | 0.001 | 0.001 |
| meta2166 |  | 760.489 | 118.084 | 0.049 | 0.034 | 0.044 | 0.054 | 0.045 | 0.033 | 0.020 | 0.040 | 0.027 | 0.036 | 0.025 | 0.040 | 0.040 | 0.029 | 0.018 |
| meta2167 |  | 761.163 | 417.219 | 0.000 | 0.000 | 0.000 | 0.000 | 0.000 | 0.001 | 0.000 | 0.000 | 0.000 | 0.001 | 0.000 | 0.001 | 0.000 | 0.000 | 0.000 |
| meta2168 |  | 761.363 | 174.202 | 0.002 | 0.002 | 0.002 | 0.003 | 0.003 | 0.002 | 0.002 | 0.003 | 0.001 | 0.003 | 0.002 | 0.001 | 0.002 | 0.003 | 0.003 |
| meta2169 |  | 761.579 | 167.003 | 0.011 | 0.011 | 0.012 | 0.005 | 0.003 | 0.003 | 0.002 | 0.006 | 0.004 | 0.004 | 0.007 | 0.007 | 0.007 | 0.002 | 0.005 |
| meta2170 |  | 762.205 | 330.195 | 0.001 | 0.001 | 0.000 | 0.000 | 0.000 | 0.000 | 0.000 | 0.000 | 0.000 | 0.001 | 0.001 | 0.000 | 0.000 | 0.000 | 0.000 |
| meta2171 |  | 762.506 | 119.041 | 0.933 | 0.602 | 0.793 | 0.272 | 0.158 | 0.161 | 0.149 | 0.379 | 0.329 | 0.452 | 0.436 | 0.642 | 0.386 | 0.178 | 0.152 |
| meta2172 |  | 762.532 | 45.193 | 0.021 | 0.021 | 0.022 | 0.012 | 0.044 | 0.009 | 0.083 | 0.054 | 0.013 | 0.013 | 0.018 | 0.018 | 0.064 | 0.067 | 0.047 |
| meta2173 |  | 763.188 | 468.881 | 0.002 | 0.002 | 0.002 | 0.002 | 0.002 | 0.004 | 0.002 | 0.003 | 0.002 | 0.003 | 0.002 | 0.004 | 0.003 | 0.002 | 0.001 |
| meta2174 |  | 763.463 | 250.885 | 0.000 | 0.000 | 0.000 | 0.000 | 0.000 | 0.000 | 0.000 | 0.000 | 0.000 | 0.000 | 0.000 | 0.000 | 0.000 | 0.000 | 0.000 |
| meta2175 |  | 764.549 | 45.193 | 0.108 | 0.100 | 0.101 | 0.100 | 0.140 | 0.060 | 0.106 | 0.131 | 0.098 | 0.118 | 0.120 | 0.049 | 0.196 | 0.176 | 0.417 |
| meta2176 |  | 765.186 | 470.856 | 0.001 | 0.001 | 0.001 | 0.001 | 0.001 | 0.001 | 0.001 | 0.001 | 0.001 | 0.001 | 0.001 | 0.001 | 0.001 | 0.000 | 0.000 |
| meta2177 |  | 765.225 | 368.648 | 0.001 | 0.001 | 0.001 | 0.001 | 0.002 | 0.002 | 0.001 | 0.001 | 0.001 | 0.001 | 0.001 | 0.002 | 0.001 | 0.001 | 0.000 |
| meta2178 |  | 765.375 | 170.896 | 0.001 | 0.001 | 0.001 | 0.001 | 0.001 | 0.001 | 0.001 | 0.001 | 0.001 | 0.001 | 0.001 | 0.001 | 0.001 | 0.001 | 0.001 |
| meta2179 |  | 765.469 | 42.746 | 0.004 | 0.002 | 0.002 | 0.006 | 0.007 | 0.004 | 0.006 | 0.003 | 0.002 | 0.004 | 0.003 | 0.006 | 0.005 | 0.005 | 0.003 |
| meta2180 |  | 766.677 | 288.039 | 0.000 | 0.000 | 0.000 | 0.001 | 0.000 | 0.000 | 0.000 | 0.000 | 0.000 | 0.000 | 0.000 | 0.000 | 0.001 | 0.001 | 0.000 |
| meta2181 |  | 769.074 | 420.690 | 0.001 | 0.001 | 0.001 | 0.001 | 0.001 | 0.000 | 0.001 | 0.001 | 0.001 | 0.001 | 0.001 | 0.001 | 0.001 | 0.001 | 0.001 |
| meta2182 |  | 769.147 | 150.809 | 0.002 | 0.001 | 0.001 | 0.002 | 0.003 | 0.001 | 0.004 | 0.002 | 0.001 | 0.000 | 0.001 | 0.002 | 0.001 | 0.001 | 0.001 |
| meta2183 |  | 769.500 | 43.916 | 0.088 | 0.083 | 0.089 | 0.131 | 0.114 | 0.085 | 0.157 | 0.097 | 0.087 | 0.111 | 0.125 | 0.178 | 0.185 | 0.145 | 0.099 |
| meta2184 |  | 770.148 | 474.951 | 0.001 | 0.000 | 0.001 | 0.001 | 0.001 | 0.001 | 0.000 | 0.001 | 0.001 | 0.001 | 0.000 | 0.001 | 0.001 | 0.001 | 0.000 |
| meta2185 |  | 770.199 | 408.579 | 0.001 | 0.001 | 0.000 | 0.001 | 0.001 | 0.001 | 0.000 | 0.001 | 0.000 | 0.001 | 0.001 | 0.001 | 0.000 | 0.000 | 0.000 |
| meta2186 |  | 770.299 | 361.373 | 0.001 | 0.001 | 0.001 | 0.001 | 0.001 | 0.000 | 0.001 | 0.001 | 0.000 | 0.001 | 0.001 | 0.001 | 0.001 | 0.000 | 0.001 |
| meta2187 |  | 770.639 | 288.385 | 0.000 | 0.000 | 0.000 | 0.001 | 0.001 | 0.000 | 0.000 | 0.000 | 0.000 | 0.000 | 0.000 | 0.000 | 0.001 | 0.000 | 0.001 |
| meta2188 |  | 771.058 | 273.911 | 0.002 | 0.002 | 0.002 | 0.003 | 0.002 | 0.002 | 0.002 | 0.003 | 0.002 | 0.003 | 0.002 | 0.002 | 0.002 | 0.002 | 0.002 |
| meta2189 |  | 771.179 | 479.023 | 0.002 | 0.002 | 0.002 | 0.003 | 0.003 | 0.002 | 0.002 | 0.003 | 0.002 | 0.002 | 0.002 | 0.003 | 0.003 | 0.002 | 0.002 |
| meta2190 |  | 773.009 | 97.105 | 0.000 | 0.001 | 0.001 | 0.000 | 0.000 | 0.000 | 0.003 | 0.000 | 0.000 | 0.000 | 0.000 | 0.000 | 0.001 | 0.000 | 0.001 |
| meta2191 |  | 773.194 | 483.030 | 0.009 | 0.009 | 0.010 | 0.012 | 0.012 | 0.010 | 0.010 | 0.013 | 0.011 | 0.011 | 0.009 | 0.011 | 0.014 | 0.010 | 0.010 |
| meta2192 |  | 773.532 | 45.266 | 0.136 | 0.119 | 0.112 | 0.056 | 0.041 | 0.038 | 0.050 | 0.096 | 0.066 | 0.057 | 0.117 | 0.143 | 0.070 | 0.042 | 0.054 |
| meta2193 |  | 775.161 | 150.815 | 0.002 | 0.001 | 0.002 | 0.002 | 0.004 | 0.002 | 0.005 | 0.002 | 0.001 | 0.000 | 0.001 | 0.002 | 0.001 | 0.001 | 0.001 |
| meta2194 |  | 775.188 | 408.824 | 0.001 | 0.001 | 0.001 | 0.001 | 0.001 | 0.001 | 0.001 | 0.002 | 0.001 | 0.001 | 0.001 | 0.002 | 0.001 | 0.000 | 0.000 |
| meta2195 |  | 776.373 | 45.976 | 0.000 | 0.000 | 0.000 | 0.000 | 0.000 | 0.000 | 0.000 | 0.000 | 0.000 | 0.000 | 0.001 | 0.000 | 0.000 | 0.000 | 0.000 |
| meta2196 |  | 777.275 | 220.337 | 0.000 | 0.000 | 0.000 | 0.000 | 0.000 | 0.000 | 0.001 | 0.000 | 0.000 | 0.000 | 0.000 | 0.000 | 0.000 | 0.000 | 0.000 |
| meta2197 |  | 779.094 | 420.701 | 0.001 | 0.001 | 0.001 | 0.001 | 0.001 | 0.000 | 0.001 | 0.001 | 0.001 | 0.001 | 0.001 | 0.001 | 0.001 | 0.001 | 0.001 |
| meta2198 |  | 781.037 | 417.660 | 0.000 | 0.000 | 0.000 | 0.001 | 0.000 | 0.000 | 0.000 | 0.000 | 0.000 | 0.000 | 0.000 | 0.000 | 0.000 | 0.000 | 0.000 |
| meta2199 |  | 781.322 | 182.812 | 0.001 | 0.002 | 0.002 | 0.001 | 0.001 | 0.002 | 0.002 | 0.002 | 0.000 | 0.002 | 0.002 | 0.000 | 0.002 | 0.002 | 0.002 |
| meta2200 |  | 782.132 | 363.877 | 0.000 | 0.000 | 0.000 | 0.000 | 0.000 | 0.000 | 0.000 | 0.000 | 0.000 | 0.000 | 0.000 | 0.000 | 0.000 | 0.000 | 0.000 |
| meta2201 |  | 782.495 | 174.993 | 0.010 | 0.009 | 0.009 | 0.008 | 0.005 | 0.004 | 0.006 | 0.008 | 0.008 | 0.009 | 0.010 | 0.012 | 0.009 | 0.006 | 0.008 |
| meta2202 |  | 783.515 | 100.554 | 0.016 | 0.017 | 0.016 | 0.004 | 0.006 | 0.003 | 0.004 | 0.008 | 0.007 | 0.007 | 0.016 | 0.017 | 0.011 | 0.005 | 0.017 |
| meta2203 |  | 783.513 | 44.514 | 0.003 | 0.003 | 0.003 | 0.003 | 0.001 | 0.001 | 0.001 | 0.002 | 0.002 | 0.001 | 0.003 | 0.005 | 0.002 | 0.002 | 0.003 |
| meta2204 |  | 784.147 | 383.939 | 0.018 | 0.016 | 0.016 | 0.018 | 0.018 | 0.015 | 0.017 | 0.018 | 0.017 | 0.019 | 0.016 | 0.018 | 0.021 | 0.016 | 0.019 |
| meta2205 |  | 784.489 | 116.725 | 0.004 | 0.005 | 0.003 | 0.006 | 0.004 | 0.004 | 0.003 | 0.004 | 0.005 | 0.001 | 0.003 | 0.006 | 0.006 | 0.005 | 0.002 |
| meta2206 | Flavin adenine dinucleotide (FAD) | 785.150 | 383.939 | 0.006 | 0.006 | 0.005 | 0.006 | 0.006 | 0.005 | 0.006 | 0.006 | 0.006 | 0.006 | 0.005 | 0.006 | 0.007 | 0.005 | 0.006 |
| meta2207 |  | 786.478 | 29.282 | 0.001 | 0.001 | 0.001 | 0.000 | 0.000 | 0.000 | 0.000 | 0.001 | 0.001 | 0.000 | 0.001 | 0.001 | 0.000 | 0.000 | 0.001 |
| meta2208 |  | 786.505 | 115.942 | 0.022 | 0.026 | 0.024 | 0.020 | 0.014 | 0.018 | 0.015 | 0.013 | 0.029 | 0.012 | 0.023 | 0.032 | 0.027 | 0.016 | 0.010 |
| meta2209 |  | 786.525 | 177.937 | 0.001 | 0.001 | 0.001 | 0.000 | 0.000 | 0.000 | 0.000 | 0.001 | 0.001 | 0.000 | 0.001 | 0.001 | 0.000 | 0.000 | 0.000 |
| meta2210 |  | 787.026 | 274.006 | 0.001 | 0.001 | 0.001 | 0.001 | 0.001 | 0.001 | 0.001 | 0.001 | 0.001 | 0.001 | 0.001 | 0.001 | 0.001 | 0.001 | 0.001 |
| meta2211 |  | 787.329 | 24.771 | 0.001 | 0.001 | 0.001 | 0.001 | 0.001 | 0.001 | 0.001 | 0.003 | 0.001 | 0.002 | 0.002 | 0.001 | 0.001 | 0.001 | 0.001 |
| meta2212 |  | 787.453 | 41.904 | 0.001 | 0.001 | 0.002 | 0.003 | 0.008 | 0.003 | 0.004 | 0.004 | 0.002 | 0.002 | 0.002 | 0.005 | 0.002 | 0.004 | 0.002 |
| meta2213 |  | 788.022 | 420.274 | 0.001 | 0.000 | 0.000 | 0.001 | 0.001 | 0.000 | 0.001 | 0.000 | 0.000 | 0.001 | 0.001 | 0.000 | 0.000 | 0.000 | 0.001 |
| meta2214 |  | 788.522 | 115.033 | 0.177 | 0.156 | 0.189 | 0.083 | 0.084 | 0.051 | 0.046 | 0.123 | 0.099 | 0.058 | 0.112 | 0.271 | 0.134 | 0.103 | 0.055 |
| meta2215 |  | 788.521 | 127.500 | 0.054 | 0.049 | 0.046 | 0.027 | 0.033 | 0.022 | 0.030 | 0.031 | 0.035 | 0.027 | 0.047 | 0.053 | 0.046 | 0.030 | 0.035 |
| meta2216 |  | 788.749 | 287.547 | 0.001 | 0.001 | 0.001 | 0.001 | 0.001 | 0.001 | 0.001 | 0.001 | 0.001 | 0.001 | 0.001 | 0.001 | 0.002 | 0.001 | 0.001 |
| meta2217 |  | 789.214 | 341.328 | 0.001 | 0.001 | 0.001 | 0.001 | 0.001 | 0.002 | 0.001 | 0.002 | 0.001 | 0.002 | 0.001 | 0.001 | 0.001 | 0.001 | 0.000 |
| meta2218 |  | 789.395 | 170.919 | 0.001 | 0.001 | 0.002 | 0.002 | 0.002 | 0.001 | 0.002 | 0.002 | 0.001 | 0.002 | 0.002 | 0.001 | 0.001 | 0.002 | 0.002 |
| meta2219 |  | 790.560 | 44.877 | 0.001 | 0.002 | 0.002 | 0.001 | 0.001 | 0.001 | 0.001 | 0.003 | 0.001 | 0.001 | 0.002 | 0.002 | 0.003 | 0.005 | 0.002 |
| meta2220 |  | 791.083 | 417.137 | 0.001 | 0.001 | 0.001 | 0.001 | 0.001 | 0.001 | 0.001 | 0.001 | 0.001 | 0.001 | 0.001 | 0.001 | 0.001 | 0.001 | 0.001 |
| meta2221 |  | 791.132 | 150.803 | 0.002 | 0.001 | 0.001 | 0.002 | 0.002 | 0.001 | 0.003 | 0.001 | 0.000 | 0.000 | 0.001 | 0.001 | 0.001 | 0.001 | 0.001 |
| meta2222 |  | 791.485 | 42.478 | 0.039 | 0.037 | 0.037 | 0.096 | 0.070 | 0.064 | 0.084 | 0.057 | 0.027 | 0.069 | 0.058 | 0.071 | 0.045 | 0.072 | 0.068 |
| meta2223 |  | 792.238 | 343.832 | 0.005 | 0.004 | 0.004 | 0.003 | 0.004 | 0.005 | 0.004 | 0.007 | 0.002 | 0.004 | 0.004 | 0.005 | 0.004 | 0.002 | 0.002 |
| meta2224 |  | 792.533 | 130.014 | 0.036 | 0.041 | 0.035 | 0.038 | 0.048 | 0.040 | 0.039 | 0.037 | 0.026 | 0.063 | 0.044 | 0.031 | 0.059 | 0.018 | 0.043 |
| meta2225 |  | 792.571 | 139.585 | 0.008 | 0.009 | 0.008 | 0.002 | 0.001 | 0.002 | 0.001 | 0.004 | 0.002 | 0.002 | 0.004 | 0.006 | 0.004 | 0.001 | 0.002 |
| meta2226 |  | 792.711 | 288.182 | 0.001 | 0.001 | 0.001 | 0.001 | 0.001 | 0.001 | 0.001 | 0.001 | 0.001 | 0.001 | 0.001 | 0.001 | 0.001 | 0.001 | 0.001 |
| meta2227 |  | 793.199 | 371.153 | 0.001 | 0.001 | 0.001 | 0.002 | 0.002 | 0.001 | 0.002 | 0.002 | 0.001 | 0.002 | 0.002 | 0.001 | 0.001 | 0.001 | 0.001 |
| meta2228 |  | 794.206 | 477.778 | 0.000 | 0.001 | 0.001 | 0.001 | 0.001 | 0.001 | 0.000 | 0.001 | 0.001 | 0.001 | 0.001 | 0.001 | 0.001 | 0.001 | 0.000 |
| meta2229 |  | 795.177 | 483.203 | 0.001 | 0.001 | 0.001 | 0.001 | 0.001 | 0.001 | 0.001 | 0.001 | 0.001 | 0.001 | 0.001 | 0.001 | 0.001 | 0.001 | 0.001 |
| meta2230 |  | 796.139 | 433.226 | 0.001 | 0.001 | 0.001 | 0.001 | 0.001 | 0.001 | 0.001 | 0.001 | 0.001 | 0.001 | 0.001 | 0.001 | 0.001 | 0.001 | 0.001 |
| meta2231 |  | 796.233 | 404.833 | 0.002 | 0.001 | 0.001 | 0.001 | 0.001 | 0.002 | 0.001 | 0.002 | 0.001 | 0.002 | 0.002 | 0.003 | 0.002 | 0.001 | 0.001 |
| meta2232 |  | 797.141 | 150.800 | 0.001 | 0.000 | 0.000 | 0.001 | 0.001 | 0.000 | 0.001 | 0.000 | 0.000 | 0.000 | 0.000 | 0.000 | 0.000 | 0.000 | 0.000 |
| meta2233 |  | 798.338 | 182.707 | 0.001 | 0.001 | 0.001 | 0.000 | 0.000 | 0.001 | 0.003 | 0.003 | 0.000 | 0.001 | 0.002 | 0.001 | 0.001 | 0.001 | 0.000 |
| meta2234 |  | 798.648 | 288.974 | 0.001 | 0.000 | 0.001 | 0.001 | 0.000 | 0.000 | 0.001 | 0.000 | 0.001 | 0.000 | 0.000 | 0.000 | 0.001 | 0.001 | 0.001 |
| meta2235 |  | 799.158 | 399.291 | 0.001 | 0.001 | 0.001 | 0.002 | 0.002 | 0.001 | 0.001 | 0.001 | 0.001 | 0.001 | 0.001 | 0.001 | 0.002 | 0.001 | 0.002 |
| meta2236 |  | 799.234 | 468.777 | 0.001 | 0.001 | 0.001 | 0.000 | 0.001 | 0.001 | 0.000 | 0.001 | 0.000 | 0.001 | 0.001 | 0.001 | 0.001 | 0.000 | 0.000 |
| meta2237 |  | 800.067 | 420.805 | 0.001 | 0.001 | 0.001 | 0.001 | 0.001 | 0.001 | 0.001 | 0.001 | 0.001 | 0.001 | 0.001 | 0.001 | 0.001 | 0.001 | 0.001 |
| meta2238 |  | 800.141 | 388.254 | 0.001 | 0.001 | 0.000 | 0.001 | 0.001 | 0.000 | 0.000 | 0.000 | 0.001 | 0.000 | 0.001 | 0.001 | 0.001 | 0.000 | 0.001 |
| meta2239 |  | 802.491 | 45.263 | 0.002 | 0.002 | 0.002 | 0.002 | 0.003 | 0.002 | 0.003 | 0.004 | 0.002 | 0.001 | 0.001 | 0.002 | 0.003 | 0.003 | 0.004 |
| meta2240 |  | 803.183 | 468.547 | 0.000 | 0.000 | 0.000 | 0.000 | 0.000 | 0.000 | 0.000 | 0.000 | 0.000 | 0.000 | 0.000 | 0.001 | 0.000 | 0.000 | 0.000 |
| meta2241 |  | 803.221 | 334.740 | 0.004 | 0.003 | 0.003 | 0.004 | 0.004 | 0.005 | 0.002 | 0.005 | 0.002 | 0.004 | 0.004 | 0.003 | 0.003 | 0.002 | 0.002 |
| meta2242 |  | 803.232 | 200.063 | 0.011 | 0.010 | 0.010 | 0.013 | 0.018 | 0.012 | 0.017 | 0.007 | 0.004 | 0.006 | 0.008 | 0.013 | 0.009 | 0.014 | 0.008 |
| meta2243 |  | 803.305 | 160.595 | 0.001 | 0.001 | 0.001 | 0.000 | 0.000 | 0.001 | 0.001 | 0.002 | 0.000 | 0.001 | 0.001 | 0.000 | 0.001 | 0.001 | 0.001 |
| meta2244 |  | 803.528 | 151.440 | 0.001 | 0.000 | 0.000 | 0.000 | 0.000 | 0.000 | 0.000 | 0.001 | 0.001 | 0.001 | 0.000 | 0.000 | 0.001 | 0.001 | 0.004 |
| meta2245 |  | 804.515 | 45.235 | 0.004 | 0.004 | 0.005 | 0.010 | 0.009 | 0.004 | 0.008 | 0.005 | 0.005 | 0.004 | 0.005 | 0.009 | 0.006 | 0.007 | 0.005 |
| meta2246 |  | 805.167 | 333.502 | 0.001 | 0.001 | 0.001 | 0.001 | 0.001 | 0.001 | 0.000 | 0.001 | 0.001 | 0.001 | 0.001 | 0.000 | 0.001 | 0.001 | 0.001 |
| meta2247 |  | 805.221 | 405.512 | 0.001 | 0.001 | 0.001 | 0.001 | 0.001 | 0.002 | 0.001 | 0.002 | 0.001 | 0.001 | 0.001 | 0.002 | 0.001 | 0.001 | 0.001 |
| meta2248 |  | 806.129 | 383.989 | 0.002 | 0.002 | 0.002 | 0.002 | 0.002 | 0.002 | 0.002 | 0.002 | 0.002 | 0.002 | 0.002 | 0.002 | 0.002 | 0.002 | 0.002 |
| meta2249 |  | 806.495 | 174.156 | 0.016 | 0.015 | 0.017 | 0.017 | 0.012 | 0.011 | 0.013 | 0.016 | 0.015 | 0.012 | 0.017 | 0.020 | 0.016 | 0.013 | 0.012 |
| meta2250 |  | 807.235 | 468.730 | 0.001 | 0.001 | 0.001 | 0.001 | 0.001 | 0.001 | 0.001 | 0.001 | 0.001 | 0.001 | 0.001 | 0.002 | 0.001 | 0.001 | 0.001 |
| meta2251 |  | 807.250 | 352.169 | 0.001 | 0.001 | 0.001 | 0.001 | 0.001 | 0.001 | 0.001 | 0.001 | 0.000 | 0.001 | 0.001 | 0.001 | 0.001 | 0.000 | 0.000 |
| meta2252 | Acetyl coenzyme A (Acetyl-CoA) | 808.116 | 416.060 | 0.001 | 0.001 | 0.001 | 0.001 | 0.001 | 0.001 | 0.001 | 0.001 | 0.001 | 0.001 | 0.001 | 0.001 | 0.002 | 0.001 | 0.001 |
| meta2253 |  | 809.310 | 25.028 | 0.001 | 0.001 | 0.001 | 0.001 | 0.001 | 0.001 | 0.001 | 0.002 | 0.001 | 0.002 | 0.002 | 0.001 | 0.001 | 0.001 | 0.001 |
| meta2254 |  | 810.231 | 182.775 | 0.002 | 0.001 | 0.001 | 0.000 | 0.001 | 0.001 | 0.001 | 0.001 | 0.001 | 0.001 | 0.002 | 0.001 | 0.001 | 0.001 | 0.001 |
| meta2255 |  | 811.281 | 428.016 | 0.001 | 0.000 | 0.000 | 0.001 | 0.001 | 0.001 | 0.000 | 0.001 | 0.000 | 0.001 | 0.001 | 0.001 | 0.000 | 0.000 | 0.000 |
| meta2256 |  | 812.793 | 286.366 | 0.000 | 0.000 | 0.000 | 0.000 | 0.000 | 0.000 | 0.000 | 0.000 | 0.000 | 0.000 | 0.000 | 0.000 | 0.000 | 0.000 | 0.000 |
| meta2257 |  | 813.065 | 416.865 | 0.001 | 0.001 | 0.001 | 0.001 | 0.001 | 0.001 | 0.001 | 0.001 | 0.001 | 0.001 | 0.001 | 0.001 | 0.001 | 0.001 | 0.001 |
| meta2258 |  | 813.529 | 40.099 | 0.002 | 0.001 | 0.002 | 0.002 | 0.000 | 0.002 | 0.006 | 0.003 | 0.001 | 0.002 | 0.002 | 0.006 | 0.003 | 0.004 | 0.005 |
| meta2259 |  | 814.557 | 128.169 | 0.065 | 0.064 | 0.054 | 0.147 | 0.148 | 0.050 | 0.092 | 0.075 | 0.121 | 0.077 | 0.055 | 0.071 | 0.118 | 0.134 | 0.068 |
| meta2260 |  | 815.263 | 350.825 | 0.001 | 0.001 | 0.001 | 0.001 | 0.001 | 0.001 | 0.001 | 0.001 | 0.000 | 0.001 | 0.001 | 0.001 | 0.001 | 0.000 | 0.000 |
| meta2261 |  | 815.484 | 41.530 | 0.024 | 0.027 | 0.031 | 0.066 | 0.062 | 0.048 | 0.071 | 0.040 | 0.031 | 0.053 | 0.060 | 0.078 | 0.053 | 0.065 | 0.060 |
| meta2262 |  | 816.531 | 104.206 | 0.016 | 0.010 | 0.011 | 0.013 | 0.011 | 0.006 | 0.010 | 0.009 | 0.006 | 0.011 | 0.011 | 0.004 | 0.005 | 0.008 | 0.010 |
| meta2263 |  | 816.573 | 129.740 | 0.764 | 1.219 | 1.281 | 0.545 | 0.406 | 0.357 | 0.481 | 0.967 | 0.844 | 0.476 | 1.062 | 1.434 | 0.480 | 0.682 | 0.443 |
| meta2264 |  | 817.500 | 41.377 | 0.262 | 0.262 | 0.295 | 0.635 | 0.564 | 0.415 | 0.651 | 0.340 | 0.321 | 0.463 | 0.656 | 0.835 | 0.533 | 0.627 | 0.593 |
| meta2265 |  | 821.193 | 468.292 | 0.000 | 0.000 | 0.000 | 0.000 | 0.000 | 0.001 | 0.001 | 0.000 | 0.000 | 0.001 | 0.000 | 0.001 | 0.001 | 0.000 | 0.000 |
| meta2266 |  | 822.049 | 420.822 | 0.001 | 0.001 | 0.001 | 0.001 | 0.001 | 0.000 | 0.001 | 0.001 | 0.001 | 0.001 | 0.001 | 0.001 | 0.001 | 0.001 | 0.001 |
| meta2267 |  | 824.721 | 318.126 | 0.001 | 0.001 | 0.001 | 0.001 | 0.001 | 0.001 | 0.001 | 0.001 | 0.001 | 0.001 | 0.001 | 0.001 | 0.001 | 0.001 | 0.001 |
| meta2268 |  | 824.981 | 85.738 | 0.008 | 0.008 | 0.008 | 0.015 | 0.018 | 0.011 | 0.015 | 0.007 | 0.006 | 0.007 | 0.011 | 0.003 | 0.009 | 0.006 | 0.012 |
| meta2269 |  | 825.213 | 200.141 | 0.007 | 0.007 | 0.007 | 0.009 | 0.009 | 0.008 | 0.010 | 0.006 | 0.004 | 0.005 | 0.006 | 0.008 | 0.006 | 0.008 | 0.006 |
| meta2270 |  | 825.248 | 452.859 | 0.006 | 0.004 | 0.005 | 0.005 | 0.006 | 0.006 | 0.006 | 0.007 | 0.004 | 0.005 | 0.005 | 0.007 | 0.005 | 0.003 | 0.003 |
| meta2271 |  | 825.500 | 118.756 | 0.006 | 0.004 | 0.006 | 0.002 | 0.001 | 0.001 | 0.002 | 0.003 | 0.002 | 0.004 | 0.005 | 0.002 | 0.003 | 0.002 | 0.001 |
| meta2272 |  | 827.118 | 461.154 | 0.001 | 0.001 | 0.001 | 0.001 | 0.001 | 0.001 | 0.001 | 0.001 | 0.001 | 0.001 | 0.001 | 0.001 | 0.001 | 0.001 | 0.001 |
| meta2273 |  | 827.195 | 428.849 | 0.009 | 0.007 | 0.007 | 0.009 | 0.007 | 0.008 | 0.008 | 0.012 | 0.006 | 0.009 | 0.008 | 0.012 | 0.007 | 0.004 | 0.005 |
| meta2274 |  | 827.264 | 485.412 | 0.004 | 0.004 | 0.004 | 0.005 | 0.004 | 0.006 | 0.005 | 0.007 | 0.004 | 0.005 | 0.004 | 0.006 | 0.005 | 0.003 | 0.002 |
| meta2275 |  | 827.485 | 40.731 | 0.002 | 0.002 | 0.003 | 0.005 | 0.008 | 0.003 | 0.006 | 0.002 | 0.004 | 0.002 | 0.002 | 0.006 | 0.002 | 0.005 | 0.003 |
| meta2276 |  | 827.559 | 116.231 | 0.022 | 0.032 | 0.030 | 0.035 | 0.014 | 0.001 | 0.009 | 0.028 | 0.033 | 0.029 | 0.027 | 0.009 | 0.012 | 0.001 | 0.008 |
| meta2277 |  | 830.320 | 361.367 | 0.001 | 0.001 | 0.001 | 0.001 | 0.001 | 0.000 | 0.001 | 0.001 | 0.000 | 0.001 | 0.001 | 0.001 | 0.000 | 0.000 | 0.001 |
| meta2278 |  | 830.528 | 45.281 | 0.002 | 0.002 | 0.002 | 0.003 | 0.003 | 0.001 | 0.002 | 0.001 | 0.002 | 0.002 | 0.002 | 0.003 | 0.002 | 0.003 | 0.002 |
| meta2279 |  | 830.588 | 128.925 | 0.022 | 0.022 | 0.021 | 0.012 | 0.008 | 0.003 | 0.004 | 0.011 | 0.014 | 0.014 | 0.019 | 0.026 | 0.015 | 0.008 | 0.008 |
| meta2280 |  | 830.663 | 287.983 | 0.001 | 0.001 | 0.001 | 0.001 | 0.001 | 0.001 | 0.001 | 0.001 | 0.001 | 0.001 | 0.001 | 0.001 | 0.001 | 0.001 | 0.001 |
| meta2281 |  | 832.298 | 361.367 | 0.003 | 0.002 | 0.002 | 0.003 | 0.003 | 0.002 | 0.005 | 0.002 | 0.001 | 0.003 | 0.004 | 0.003 | 0.002 | 0.001 | 0.005 |
| meta2282 |  | 833.200 | 200.205 | 0.000 | 0.000 | 0.000 | 0.001 | 0.001 | 0.001 | 0.001 | 0.001 | 0.001 | 0.000 | 0.001 | 0.000 | 0.001 | 0.000 | 0.001 |
| meta2283 |  | 833.363 | 168.428 | 0.000 | 0.000 | 0.000 | 0.000 | 0.000 | 0.000 | 0.000 | 0.000 | 0.000 | 0.000 | 0.000 | 0.000 | 0.000 | 0.000 | 0.000 |
| meta2284 |  | 834.112 | 421.502 | 0.001 | 0.001 | 0.001 | 0.001 | 0.001 | 0.001 | 0.001 | 0.001 | 0.001 | 0.001 | 0.001 | 0.001 | 0.002 | 0.001 | 0.001 |
| meta2285 |  | 834.526 | 173.003 | 0.044 | 0.039 | 0.042 | 0.025 | 0.014 | 0.015 | 0.018 | 0.034 | 0.031 | 0.013 | 0.042 | 0.052 | 0.025 | 0.016 | 0.018 |
| meta2286 |  | 834.626 | 288.676 | 0.001 | 0.001 | 0.001 | 0.001 | 0.001 | 0.001 | 0.001 | 0.001 | 0.001 | 0.001 | 0.001 | 0.001 | 0.001 | 0.001 | 0.001 |
| meta2287 |  | 835.047 | 416.772 | 0.001 | 0.001 | 0.001 | 0.001 | 0.001 | 0.001 | 0.001 | 0.001 | 0.001 | 0.001 | 0.001 | 0.001 | 0.001 | 0.001 | 0.001 |
| meta2288 |  | 835.509 | 44.471 | 0.001 | 0.001 | 0.002 | 0.003 | 0.003 | 0.002 | 0.004 | 0.002 | 0.001 | 0.002 | 0.003 | 0.004 | 0.002 | 0.002 | 0.002 |
| meta2289 |  | 836.006 | 162.477 | 0.000 | 0.000 | 0.000 | 0.001 | 0.001 | 0.000 | 0.000 | 0.000 | 0.001 | 0.000 | 0.000 | 0.000 | 0.000 | 0.000 | 0.000 |
| meta2290 | Isobutyryl-CoA | 836.147 | 393.006 | 0.001 | 0.001 | 0.001 | 0.001 | 0.001 | 0.001 | 0.001 | 0.001 | 0.001 | 0.001 | 0.001 | 0.001 | 0.001 | 0.001 | 0.001 |
| meta2291 |  | 836.371 | 136.730 | 0.000 | 0.000 | 0.000 | 0.000 | 0.000 | 0.001 | 0.001 | 0.000 | 0.000 | 0.001 | 0.001 | 0.000 | 0.001 | 0.001 | 0.000 |
| meta2292 |  | 836.427 | 151.404 | 0.000 | 0.000 | 0.000 | 0.001 | 0.001 | 0.000 | 0.000 | 0.000 | 0.000 | 0.000 | 0.000 | 0.000 | 0.000 | 0.000 | 0.000 |
| meta2293 |  | 836.542 | 114.994 | 0.012 | 0.011 | 0.012 | 0.019 | 0.025 | 0.016 | 0.013 | 0.020 | 0.014 | 0.022 | 0.018 | 0.004 | 0.018 | 0.018 | 0.016 |
| meta2294 |  | 838.387 | 170.901 | 0.000 | 0.000 | 0.000 | 0.000 | 0.000 | 0.000 | 0.000 | 0.000 | 0.000 | 0.000 | 0.000 | 0.000 | 0.000 | 0.000 | 0.000 |
| meta2295 |  | 838.441 | 170.862 | 0.001 | 0.001 | 0.001 | 0.002 | 0.002 | 0.001 | 0.001 | 0.002 | 0.001 | 0.001 | 0.001 | 0.002 | 0.001 | 0.001 | 0.002 |
| meta2296 |  | 838.557 | 115.723 | 0.089 | 0.056 | 0.091 | 0.084 | 0.074 | 0.055 | 0.044 | 0.120 | 0.073 | 0.142 | 0.063 | 0.131 | 0.061 | 0.075 | 0.054 |
| meta2297 |  | 839.484 | 40.726 | 0.011 | 0.009 | 0.012 | 0.024 | 0.028 | 0.016 | 0.018 | 0.014 | 0.015 | 0.026 | 0.031 | 0.036 | 0.015 | 0.030 | 0.040 |
| meta2298 |  | 840.574 | 125.896 | 0.525 | 0.637 | 0.539 | 1.213 | 0.575 | 0.721 | 0.795 | 0.434 | 0.688 | 0.861 | 0.603 | 0.492 | 0.525 | 0.901 | 0.789 |
| meta2299 |  | 840.955 | 85.498 | 0.004 | 0.003 | 0.004 | 0.006 | 0.007 | 0.004 | 0.007 | 0.003 | 0.003 | 0.003 | 0.005 | 0.001 | 0.004 | 0.003 | 0.005 |
| meta2300 |  | 841.182 | 200.071 | 0.003 | 0.003 | 0.003 | 0.005 | 0.005 | 0.003 | 0.004 | 0.003 | 0.002 | 0.002 | 0.003 | 0.003 | 0.003 | 0.003 | 0.003 |
| meta2301 |  | 841.500 | 40.403 | 0.075 | 0.066 | 0.075 | 0.202 | 0.264 | 0.126 | 0.204 | 0.087 | 0.084 | 0.119 | 0.175 | 0.180 | 0.108 | 0.140 | 0.156 |
| meta2302 |  | 847.196 | 200.306 | 0.006 | 0.005 | 0.005 | 0.007 | 0.007 | 0.005 | 0.007 | 0.005 | 0.004 | 0.004 | 0.005 | 0.006 | 0.006 | 0.006 | 0.005 |
| meta2303 |  | 847.447 | 250.888 | 0.000 | 0.000 | 0.000 | 0.000 | 0.000 | 0.000 | 0.000 | 0.000 | 0.000 | 0.000 | 0.000 | 0.000 | 0.000 | 0.000 | 0.000 |
| meta2304 |  | 847.486 | 119.509 | 0.006 | 0.005 | 0.008 | 0.006 | 0.002 | 0.002 | 0.002 | 0.007 | 0.007 | 0.005 | 0.004 | 0.007 | 0.006 | 0.003 | 0.002 |
| meta2305 |  | 848.241 | 371.397 | 0.001 | 0.001 | 0.001 | 0.001 | 0.001 | 0.001 | 0.001 | 0.001 | 0.001 | 0.001 | 0.001 | 0.001 | 0.001 | 0.001 | 0.001 |
| meta2306 |  | 849.489 | 43.897 | 0.003 | 0.002 | 0.003 | 0.003 | 0.006 | 0.004 | 0.012 | 0.004 | 0.003 | 0.002 | 0.007 | 0.011 | 0.005 | 0.005 | 0.007 |
| meta2307 | 3-Methylbutanoyl-CoA | 850.162 | 383.939 | 0.001 | 0.001 | 0.001 | 0.001 | 0.001 | 0.001 | 0.002 | 0.001 | 0.001 | 0.001 | 0.001 | 0.001 | 0.001 | 0.001 | 0.001 |
| meta2308 |  | 850.221 | 426.141 | 0.004 | 0.004 | 0.005 | 0.005 | 0.003 | 0.006 | 0.004 | 0.006 | 0.004 | 0.005 | 0.005 | 0.006 | 0.004 | 0.003 | 0.003 |
| meta2309 |  | 851.146 | 150.806 | 0.001 | 0.001 | 0.000 | 0.001 | 0.001 | 0.001 | 0.002 | 0.001 | 0.000 | 0.000 | 0.000 | 0.001 | 0.000 | 0.000 | 0.000 |
| meta2310 |  | 851.560 | 112.346 | 0.023 | 0.034 | 0.032 | 0.042 | 0.031 | 0.023 | 0.026 | 0.012 | 0.032 | 0.009 | 0.034 | 0.003 | 0.028 | 0.027 | 0.022 |
| meta2311 | DL-3-Hydroxybutyryl-CoA | 852.141 | 396.926 | 0.001 | 0.000 | 0.001 | 0.000 | 0.001 | 0.000 | 0.001 | 0.000 | 0.000 | 0.000 | 0.000 | 0.000 | 0.001 | 0.000 | 0.000 |
| meta2312 |  | 852.301 | 361.373 | 0.001 | 0.001 | 0.000 | 0.001 | 0.001 | 0.000 | 0.001 | 0.001 | 0.000 | 0.001 | 0.001 | 0.001 | 0.000 | 0.000 | 0.001 |
| meta2313 |  | 854.365 | 182.256 | 0.001 | 0.001 | 0.001 | 0.000 | 0.000 | 0.001 | 0.002 | 0.002 | 0.000 | 0.001 | 0.001 | 0.000 | 0.001 | 0.001 | 0.000 |
| meta2314 |  | 854.588 | 124.961 | 0.016 | 0.020 | 0.018 | 0.016 | 0.015 | 0.014 | 0.017 | 0.025 | 0.015 | 0.023 | 0.014 | 0.015 | 0.026 | 0.016 | 0.008 |
| meta2315 |  | 855.222 | 405.188 | 0.001 | 0.001 | 0.001 | 0.002 | 0.001 | 0.002 | 0.001 | 0.001 | 0.001 | 0.002 | 0.001 | 0.002 | 0.001 | 0.001 | 0.001 |
| meta2316 |  | 855.252 | 483.030 | 0.000 | 0.000 | 0.000 | 0.001 | 0.001 | 0.000 | 0.001 | 0.000 | 0.001 | 0.000 | 0.000 | 0.001 | 0.001 | 0.001 | 0.001 |
| meta2317 |  | 855.501 | 175.595 | 0.002 | 0.002 | 0.002 | 0.002 | 0.002 | 0.001 | 0.001 | 0.003 | 0.002 | 0.002 | 0.002 | 0.002 | 0.002 | 0.002 | 0.002 |
| meta2318 |  | 855.499 | 45.721 | 0.001 | 0.001 | 0.001 | 0.002 | 0.002 | 0.001 | 0.002 | 0.001 | 0.001 | 0.001 | 0.002 | 0.002 | 0.001 | 0.001 | 0.001 |
| meta2319 |  | 857.159 | 150.784 | 0.001 | 0.001 | 0.000 | 0.001 | 0.001 | 0.000 | 0.002 | 0.001 | 0.000 | 0.000 | 0.000 | 0.000 | 0.000 | 0.000 | 0.000 |
| meta2320 |  | 857.239 | 468.754 | 0.000 | 0.001 | 0.000 | 0.000 | 0.000 | 0.001 | 0.000 | 0.001 | 0.000 | 0.001 | 0.000 | 0.001 | 0.000 | 0.000 | 0.000 |
| meta2321 |  | 857.516 | 175.429 | 0.053 | 0.052 | 0.051 | 0.038 | 0.021 | 0.024 | 0.030 | 0.054 | 0.040 | 0.046 | 0.059 | 0.060 | 0.037 | 0.031 | 0.033 |
| meta2322 |  | 858.407 | 170.333 | 0.000 | 0.000 | 0.001 | 0.001 | 0.001 | 0.001 | 0.001 | 0.001 | 0.000 | 0.000 | 0.001 | 0.001 | 0.000 | 0.000 | 0.001 |
| meta2323 |  | 859.526 | 176.059 | 0.016 | 0.016 | 0.015 | 0.009 | 0.005 | 0.006 | 0.006 | 0.018 | 0.012 | 0.014 | 0.016 | 0.018 | 0.013 | 0.009 | 0.009 |
| meta2324 |  | 860.191 | 333.759 | 0.001 | 0.001 | 0.001 | 0.002 | 0.001 | 0.001 | 0.000 | 0.003 | 0.001 | 0.002 | 0.001 | 0.000 | 0.001 | 0.001 | 0.001 |
| meta2325 |  | 860.422 | 170.906 | 0.002 | 0.002 | 0.001 | 0.002 | 0.002 | 0.001 | 0.001 | 0.002 | 0.001 | 0.001 | 0.001 | 0.002 | 0.001 | 0.001 | 0.002 |
| meta2326 |  | 860.562 | 110.700 | 0.005 | 0.005 | 0.004 | 0.004 | 0.000 | 0.003 | 0.000 | 0.004 | 0.004 | 0.004 | 0.002 | 0.005 | 0.003 | 0.002 | 0.000 |
| meta2327 |  | 861.544 | 177.345 | 0.005 | 0.005 | 0.006 | 0.002 | 0.001 | 0.001 | 0.001 | 0.005 | 0.004 | 0.004 | 0.004 | 0.006 | 0.004 | 0.002 | 0.002 |
| meta2328 |  | 862.557 | 113.011 | 0.043 | 0.044 | 0.047 | 0.082 | 0.062 | 0.035 | 0.039 | 0.068 | 0.026 | 0.027 | 0.038 | 0.060 | 0.036 | 0.034 | 0.040 |
| meta2329 |  | 863.170 | 200.349 | 0.001 | 0.002 | 0.002 | 0.003 | 0.002 | 0.001 | 0.002 | 0.002 | 0.001 | 0.001 | 0.002 | 0.002 | 0.002 | 0.002 | 0.002 |
| meta2330 |  | 865.232 | 429.499 | 0.001 | 0.001 | 0.000 | 0.001 | 0.000 | 0.000 | 0.000 | 0.001 | 0.000 | 0.001 | 0.001 | 0.001 | 0.000 | 0.000 | 0.000 |
| meta2331 |  | 865.501 | 39.783 | 0.297 | 0.232 | 0.278 | 0.699 | 0.773 | 0.428 | 0.575 | 0.334 | 0.383 | 0.558 | 1.205 | 0.854 | 0.338 | 0.588 | 1.009 |
| meta2332 |  | 871.531 | 174.789 | 0.003 | 0.004 | 0.004 | 0.001 | 0.001 | 0.001 | 0.001 | 0.002 | 0.002 | 0.002 | 0.004 | 0.005 | 0.002 | 0.001 | 0.002 |
| meta2333 |  | 873.133 | 150.806 | 0.001 | 0.000 | 0.000 | 0.001 | 0.001 | 0.000 | 0.001 | 0.001 | 0.000 | 0.000 | 0.000 | 0.000 | 0.000 | 0.000 | 0.000 |
| meta2334 |  | 874.093 | 273.911 | 0.012 | 0.010 | 0.010 | 0.014 | 0.013 | 0.011 | 0.010 | 0.016 | 0.009 | 0.016 | 0.009 | 0.006 | 0.011 | 0.010 | 0.010 |
| meta2335 |  | 876.235 | 371.752 | 0.001 | 0.001 | 0.000 | 0.001 | 0.001 | 0.001 | 0.001 | 0.001 | 0.000 | 0.001 | 0.001 | 0.001 | 0.000 | 0.000 | 0.000 |
| meta2336 |  | 876.396 | 170.919 | 0.000 | 0.000 | 0.000 | 0.000 | 0.001 | 0.000 | 0.000 | 0.000 | 0.000 | 0.000 | 0.000 | 0.000 | 0.000 | 0.000 | 0.000 |
| meta2337 |  | 876.521 | 46.638 | 0.000 | 0.001 | 0.001 | 0.000 | 0.001 | 0.001 | 0.001 | 0.000 | 0.001 | 0.001 | 0.001 | 0.001 | 0.001 | 0.001 | 0.001 |
| meta2338 |  | 879.497 | 174.798 | 0.000 | 0.000 | 0.000 | 0.000 | 0.000 | 0.000 | 0.000 | 0.000 | 0.001 | 0.000 | 0.000 | 0.000 | 0.000 | 0.000 | 0.000 |
| meta2339 |  | 880.391 | 170.261 | 0.000 | 0.000 | 0.000 | 0.000 | 0.001 | 0.000 | 0.000 | 0.000 | 0.000 | 0.000 | 0.000 | 0.001 | 0.000 | 0.000 | 0.001 |
| meta2340 |  | 880.544 | 250.888 | 0.000 | 0.000 | 0.000 | 0.000 | 0.000 | 0.000 | 0.000 | 0.000 | 0.000 | 0.000 | 0.000 | 0.000 | 0.000 | 0.000 | 0.000 |
| meta2341 |  | 881.494 | 43.267 | 0.002 | 0.002 | 0.002 | 0.006 | 0.006 | 0.005 | 0.006 | 0.004 | 0.002 | 0.004 | 0.011 | 0.009 | 0.003 | 0.005 | 0.007 |
| meta2342 |  | 881.516 | 174.324 | 0.005 | 0.005 | 0.005 | 0.005 | 0.003 | 0.003 | 0.004 | 0.006 | 0.007 | 0.006 | 0.007 | 0.005 | 0.005 | 0.004 | 0.004 |
| meta2343 |  | 882.173 | 333.924 | 0.002 | 0.002 | 0.001 | 0.002 | 0.002 | 0.002 | 0.001 | 0.004 | 0.001 | 0.003 | 0.001 | 0.001 | 0.001 | 0.001 | 0.001 |
| meta2344 |  | 882.800 | 173.684 | 0.001 | 0.001 | 0.001 | 0.000 | 0.000 | 0.000 | 0.000 | 0.000 | 0.000 | 0.000 | 0.001 | 0.001 | 0.000 | 0.000 | 0.000 |
| meta2345 |  | 883.532 | 173.673 | 0.024 | 0.023 | 0.022 | 0.015 | 0.011 | 0.009 | 0.010 | 0.020 | 0.016 | 0.020 | 0.026 | 0.034 | 0.027 | 0.019 | 0.023 |
| meta2346 |  | 883.530 | 45.935 | 0.005 | 0.004 | 0.004 | 0.002 | 0.002 | 0.001 | 0.001 | 0.002 | 0.002 | 0.003 | 0.007 | 0.003 | 0.003 | 0.001 | 0.003 |
| meta2347 |  | 884.156 | 420.701 | 0.001 | 0.002 | 0.002 | 0.002 | 0.001 | 0.001 | 0.002 | 0.002 | 0.002 | 0.002 | 0.002 | 0.001 | 0.001 | 0.002 | 0.002 |
| meta2348 |  | 884.563 | 110.333 | 0.011 | 0.009 | 0.010 | 0.011 | 0.008 | 0.009 | 0.002 | 0.009 | 0.010 | 0.007 | 0.009 | 0.009 | 0.006 | 0.010 | 0.002 |
| meta2349 |  | 885.548 | 173.693 | 0.297 | 0.268 | 0.274 | 0.091 | 0.063 | 0.054 | 0.082 | 0.159 | 0.113 | 0.112 | 0.260 | 0.334 | 0.138 | 0.067 | 0.123 |
| meta2350 |  | 887.110 | 420.644 | 0.000 | 0.001 | 0.001 | 0.000 | 0.000 | 0.000 | 0.000 | 0.001 | 0.001 | 0.001 | 0.000 | 0.000 | 0.001 | 0.001 | 0.001 |
| meta2351 |  | 887.286 | 485.762 | 0.001 | 0.000 | 0.000 | 0.000 | 0.000 | 0.001 | 0.000 | 0.001 | 0.000 | 0.001 | 0.000 | 0.001 | 0.000 | 0.000 | 0.000 |
| meta2352 |  | 888.573 | 123.684 | 0.011 | 0.012 | 0.012 | 0.024 | 0.031 | 0.016 | 0.026 | 0.015 | 0.023 | 0.017 | 0.019 | 0.016 | 0.021 | 0.021 | 0.031 |
| meta2353 |  | 888.705 | 287.428 | 0.001 | 0.001 | 0.000 | 0.001 | 0.001 | 0.001 | 0.001 | 0.001 | 0.001 | 0.001 | 0.001 | 0.001 | 0.001 | 0.001 | 0.001 |
| meta2354 |  | 890.155 | 432.829 | 0.002 | 0.002 | 0.002 | 0.002 | 0.002 | 0.002 | 0.002 | 0.003 | 0.003 | 0.002 | 0.002 | 0.002 | 0.003 | 0.002 | 0.002 |
| meta2355 |  | 890.260 | 485.412 | 0.001 | 0.001 | 0.000 | 0.001 | 0.000 | 0.000 | 0.000 | 0.001 | 0.000 | 0.001 | 0.001 | 0.001 | 0.000 | 0.000 | 0.000 |
| meta2356 |  | 892.603 | 123.821 | 0.059 | 0.053 | 0.083 | 0.041 | 0.067 | 0.048 | 0.047 | 0.049 | 0.085 | 0.033 | 0.128 | 0.050 | 0.038 | 0.025 | 0.045 |
| meta2357 |  | 892.666 | 288.003 | 0.001 | 0.001 | 0.001 | 0.001 | 0.001 | 0.001 | 0.001 | 0.001 | 0.001 | 0.001 | 0.001 | 0.001 | 0.001 | 0.001 | 0.001 |
| meta2358 |  | 893.528 | 45.294 | 0.001 | 0.000 | 0.000 | 0.000 | 0.000 | 0.000 | 0.000 | 0.001 | 0.000 | 0.000 | 0.001 | 0.000 | 0.000 | 0.000 | 0.000 |
| meta2359 |  | 896.074 | 274.006 | 0.002 | 0.001 | 0.002 | 0.002 | 0.002 | 0.001 | 0.001 | 0.001 | 0.002 | 0.002 | 0.002 | 0.001 | 0.002 | 0.002 | 0.002 |
| meta2360 |  | 896.629 | 288.699 | 0.001 | 0.001 | 0.001 | 0.001 | 0.001 | 0.001 | 0.001 | 0.001 | 0.001 | 0.001 | 0.001 | 0.001 | 0.001 | 0.001 | 0.001 |
| meta2361 |  | 897.490 | 42.348 | 0.003 | 0.003 | 0.004 | 0.005 | 0.011 | 0.007 | 0.012 | 0.007 | 0.005 | 0.004 | 0.019 | 0.020 | 0.005 | 0.008 | 0.014 |
| meta2362 |  | 898.276 | 370.763 | 0.000 | 0.000 | 0.000 | 0.000 | 0.001 | 0.001 | 0.000 | 0.000 | 0.000 | 0.000 | 0.000 | 0.001 | 0.000 | 0.000 | 0.000 |
| meta2363 |  | 902.475 | 41.994 | 0.001 | 0.001 | 0.002 | 0.002 | 0.001 | 0.001 | 0.001 | 0.001 | 0.002 | 0.001 | 0.001 | 0.001 | 0.002 | 0.002 | 0.001 |
| meta2364 |  | 902.609 | 173.457 | 0.001 | 0.000 | 0.001 | 0.000 | 0.000 | 0.000 | 0.001 | 0.000 | 0.000 | 0.001 | 0.000 | 0.001 | 0.000 | 0.001 | 0.000 |
| meta2365 |  | 904.155 | 333.988 | 0.002 | 0.002 | 0.002 | 0.003 | 0.003 | 0.002 | 0.001 | 0.005 | 0.001 | 0.003 | 0.002 | 0.001 | 0.002 | 0.002 | 0.002 |
| meta2366 |  | 904.209 | 433.907 | 0.001 | 0.001 | 0.001 | 0.001 | 0.001 | 0.001 | 0.001 | 0.001 | 0.001 | 0.001 | 0.001 | 0.000 | 0.001 | 0.001 | 0.001 |
| meta2367 |  | 904.566 | 174.297 | 0.002 | 0.002 | 0.002 | 0.004 | 0.003 | 0.002 | 0.004 | 0.003 | 0.001 | 0.002 | 0.002 | 0.002 | 0.002 | 0.002 | 0.003 |
| meta2368 |  | 905.172 | 428.849 | 0.001 | 0.001 | 0.001 | 0.001 | 0.001 | 0.001 | 0.001 | 0.001 | 0.001 | 0.001 | 0.001 | 0.001 | 0.001 | 0.000 | 0.000 |
| meta2369 |  | 907.529 | 172.876 | 0.001 | 0.001 | 0.001 | 0.001 | 0.000 | 0.000 | 0.000 | 0.001 | 0.001 | 0.001 | 0.001 | 0.001 | 0.001 | 0.001 | 0.001 |
| meta2370 |  | 908.299 | 468.703 | 0.000 | 0.000 | 0.000 | 0.000 | 0.000 | 0.001 | 0.000 | 0.001 | 0.000 | 0.000 | 0.000 | 0.001 | 0.000 | 0.000 | 0.000 |
| meta2371 |  | 908.562 | 110.333 | 0.012 | 0.010 | 0.012 | 0.014 | 0.014 | 0.013 | 0.007 | 0.012 | 0.012 | 0.009 | 0.011 | 0.008 | 0.009 | 0.013 | 0.006 |
| meta2372 |  | 909.547 | 172.920 | 0.003 | 0.003 | 0.004 | 0.002 | 0.001 | 0.001 | 0.001 | 0.003 | 0.003 | 0.002 | 0.003 | 0.004 | 0.002 | 0.001 | 0.002 |
| meta2373 |  | 909.959 | 86.826 | 0.001 | 0.001 | 0.001 | 0.002 | 0.002 | 0.002 | 0.002 | 0.001 | 0.001 | 0.001 | 0.001 | 0.000 | 0.001 | 0.001 | 0.002 |
| meta2374 |  | 910.183 | 418.964 | 0.006 | 0.006 | 0.006 | 0.006 | 0.005 | 0.005 | 0.006 | 0.008 | 0.008 | 0.008 | 0.006 | 0.006 | 0.007 | 0.005 | 0.007 |
| meta2375 |  | 910.183 | 433.591 | 0.001 | 0.001 | 0.001 | 0.001 | 0.001 | 0.001 | 0.001 | 0.001 | 0.001 | 0.001 | 0.001 | 0.001 | 0.001 | 0.001 | 0.001 |
| meta2376 |  | 910.519 | 147.656 | 0.001 | 0.001 | 0.001 | 0.002 | 0.002 | 0.001 | 0.002 | 0.002 | 0.002 | 0.002 | 0.002 | 0.002 | 0.001 | 0.001 | 0.002 |
| meta2377 |  | 913.485 | 44.103 | 0.000 | 0.000 | 0.000 | 0.001 | 0.001 | 0.001 | 0.001 | 0.001 | 0.001 | 0.000 | 0.001 | 0.002 | 0.000 | 0.001 | 0.001 |
| meta2378 |  | 914.554 | 51.124 | 0.000 | 0.000 | 0.000 | 0.000 | 0.000 | 0.000 | 0.000 | 0.001 | 0.001 | 0.001 | 0.000 | 0.001 | 0.001 | 0.000 | 0.000 |
| meta2379 |  | 915.332 | 362.508 | 0.000 | 0.000 | 0.000 | 0.000 | 0.000 | 0.000 | 0.000 | 0.000 | 0.000 | 0.000 | 0.000 | 0.000 | 0.000 | 0.000 | 0.000 |
| meta2380 |  | 920.127 | 333.988 | 0.001 | 0.000 | 0.000 | 0.001 | 0.001 | 0.000 | 0.000 | 0.001 | 0.000 | 0.001 | 0.000 | 0.000 | 0.000 | 0.000 | 0.000 |
| meta2381 |  | 920.577 | 98.671 | 0.002 | 0.002 | 0.002 | 0.003 | 0.001 | 0.002 | 0.000 | 0.003 | 0.002 | 0.004 | 0.001 | 0.003 | 0.002 | 0.002 | 0.000 |
| meta2382 |  | 920.621 | 154.672 | 0.001 | 0.001 | 0.001 | 0.000 | 0.000 | 0.000 | 0.000 | 0.001 | 0.000 | 0.000 | 0.001 | 0.001 | 0.000 | 0.000 | 0.000 |
| meta2383 |  | 922.351 | 250.609 | 0.000 | 0.000 | 0.000 | 0.000 | 0.000 | 0.000 | 0.000 | 0.000 | 0.000 | 0.000 | 0.000 | 0.000 | 0.000 | 0.000 | 0.000 |
| meta2384 |  | 922.576 | 113.681 | 0.035 | 0.032 | 0.032 | 0.021 | 0.032 | 0.016 | 0.024 | 0.036 | 0.020 | 0.007 | 0.029 | 0.012 | 0.013 | 0.012 | 0.022 |
| meta2385 |  | 923.181 | 200.946 | 0.001 | 0.001 | 0.001 | 0.001 | 0.001 | 0.001 | 0.001 | 0.001 | 0.001 | 0.001 | 0.001 | 0.001 | 0.001 | 0.001 | 0.001 |
| meta2386 |  | 923.503 | 208.329 | 0.001 | 0.001 | 0.001 | 0.002 | 0.001 | 0.001 | 0.001 | 0.001 | 0.001 | 0.001 | 0.001 | 0.001 | 0.001 | 0.001 | 0.001 |
| meta2387 |  | 924.367 | 249.451 | 0.001 | 0.001 | 0.001 | 0.001 | 0.001 | 0.001 | 0.001 | 0.001 | 0.000 | 0.001 | 0.000 | 0.001 | 0.000 | 0.000 | 0.000 |
| meta2388 |  | 924.557 | 159.975 | 0.023 | 0.022 | 0.022 | 0.010 | 0.009 | 0.007 | 0.007 | 0.012 | 0.008 | 0.008 | 0.014 | 0.028 | 0.012 | 0.008 | 0.008 |
| meta2389 |  | 925.115 | 434.169 | 0.001 | 0.000 | 0.001 | 0.000 | 0.000 | 0.000 | 0.000 | 0.000 | 0.001 | 0.001 | 0.001 | 0.001 | 0.001 | 0.000 | 0.001 |
| meta2390 |  | 925.159 | 371.516 | 0.000 | 0.000 | 0.000 | 0.000 | 0.000 | 0.000 | 0.000 | 0.000 | 0.000 | 0.001 | 0.001 | 0.000 | 0.000 | 0.000 | 0.000 |
| meta2391 |  | 925.240 | 485.122 | 0.000 | 0.000 | 0.000 | 0.000 | 0.000 | 0.000 | 0.000 | 0.000 | 0.000 | 0.000 | 0.000 | 0.001 | 0.000 | 0.000 | 0.000 |
| meta2392 |  | 926.137 | 333.885 | 0.001 | 0.001 | 0.001 | 0.001 | 0.001 | 0.001 | 0.000 | 0.001 | 0.001 | 0.001 | 0.001 | 0.000 | 0.001 | 0.001 | 0.001 |
| meta2393 |  | 926.160 | 200.691 | 0.000 | 0.000 | 0.000 | 0.001 | 0.000 | 0.000 | 0.000 | 0.000 | 0.000 | 0.000 | 0.000 | 0.000 | 0.000 | 0.000 | 0.000 |
| meta2394 |  | 926.191 | 434.099 | 0.001 | 0.001 | 0.001 | 0.001 | 0.001 | 0.001 | 0.001 | 0.001 | 0.001 | 0.001 | 0.001 | 0.001 | 0.001 | 0.001 | 0.001 |
| meta2395 |  | 926.570 | 159.307 | 0.011 | 0.010 | 0.010 | 0.004 | 0.004 | 0.003 | 0.002 | 0.005 | 0.003 | 0.003 | 0.007 | 0.015 | 0.006 | 0.004 | 0.004 |
| meta2396 |  | 928.588 | 159.957 | 0.010 | 0.009 | 0.009 | 0.002 | 0.001 | 0.002 | 0.001 | 0.004 | 0.002 | 0.002 | 0.005 | 0.011 | 0.003 | 0.001 | 0.002 |
| meta2397 |  | 930.625 | 288.063 | 0.001 | 0.000 | 0.000 | 0.001 | 0.001 | 0.001 | 0.001 | 0.001 | 0.001 | 0.001 | 0.001 | 0.001 | 0.001 | 0.001 | 0.000 |
| meta2398 |  | 930.641 | 170.919 | 0.001 | 0.001 | 0.001 | 0.000 | 0.001 | 0.000 | 0.003 | 0.001 | 0.001 | 0.003 | 0.001 | 0.002 | 0.001 | 0.002 | 0.001 |
| meta2399 |  | 932.165 | 418.985 | 0.001 | 0.001 | 0.001 | 0.001 | 0.001 | 0.001 | 0.001 | 0.001 | 0.001 | 0.001 | 0.001 | 0.001 | 0.001 | 0.001 | 0.001 |
| meta2400 |  | 934.526 | 46.019 | 0.003 | 0.003 | 0.003 | 0.002 | 0.002 | 0.001 | 0.002 | 0.003 | 0.004 | 0.003 | 0.003 | 0.002 | 0.002 | 0.003 | 0.002 |
| meta2401 |  | 934.586 | 288.516 | 0.000 | 0.000 | 0.000 | 0.001 | 0.001 | 0.000 | 0.000 | 0.001 | 0.001 | 0.000 | 0.000 | 0.000 | 0.000 | 0.000 | 0.000 |
| meta2402 |  | 939.516 | 176.058 | 0.000 | 0.000 | 0.000 | 0.000 | 0.000 | 0.000 | 0.000 | 0.001 | 0.000 | 0.000 | 0.001 | 0.001 | 0.000 | 0.000 | 0.000 |
| meta2403 |  | 940.553 | 119.041 | 0.009 | 0.008 | 0.008 | 0.002 | 0.002 | 0.004 | 0.002 | 0.005 | 0.005 | 0.004 | 0.004 | 0.004 | 0.007 | 0.003 | 0.002 |
| meta2404 |  | 942.564 | 98.082 | 0.002 | 0.002 | 0.001 | 0.001 | 0.000 | 0.000 | 0.000 | 0.004 | 0.003 | 0.003 | 0.000 | 0.002 | 0.002 | 0.000 | 0.000 |
| meta2405 |  | 944.009 | 162.450 | 0.000 | 0.000 | 0.001 | 0.001 | 0.001 | 0.001 | 0.000 | 0.000 | 0.001 | 0.000 | 0.000 | 0.000 | 0.000 | 0.000 | 0.001 |
| meta2406 |  | 944.577 | 98.290 | 0.015 | 0.013 | 0.011 | 0.005 | 0.001 | 0.003 | 0.000 | 0.027 | 0.020 | 0.016 | 0.003 | 0.015 | 0.010 | 0.003 | 0.000 |
| meta2407 |  | 944.621 | 154.672 | 0.000 | 0.000 | 0.000 | 0.000 | 0.000 | 0.000 | 0.000 | 0.000 | 0.000 | 0.000 | 0.000 | 0.000 | 0.000 | 0.000 | 0.000 |
| meta2408 |  | 946.352 | 30.357 | 0.001 | 0.001 | 0.001 | 0.002 | 0.001 | 0.001 | 0.002 | 0.002 | 0.002 | 0.001 | 0.002 | 0.001 | 0.001 | 0.001 | 0.001 |
| meta2409 |  | 947.607 | 174.221 | 0.000 | 0.000 | 0.000 | 0.001 | 0.001 | 0.000 | 0.001 | 0.001 | 0.000 | 0.000 | 0.001 | 0.000 | 0.000 | 0.000 | 0.001 |
| meta2410 |  | 948.172 | 434.173 | 0.001 | 0.001 | 0.001 | 0.001 | 0.001 | 0.000 | 0.001 | 0.001 | 0.001 | 0.001 | 0.001 | 0.001 | 0.001 | 0.001 | 0.001 |
| meta2411 |  | 948.367 | 248.846 | 0.001 | 0.001 | 0.001 | 0.002 | 0.002 | 0.001 | 0.001 | 0.001 | 0.000 | 0.001 | 0.001 | 0.001 | 0.001 | 0.001 | 0.001 |
| meta2412 |  | 950.573 | 158.134 | 0.006 | 0.006 | 0.006 | 0.003 | 0.002 | 0.002 | 0.002 | 0.002 | 0.002 | 0.001 | 0.004 | 0.007 | 0.003 | 0.003 | 0.002 |
| meta2413 |  | 953.134 | 419.907 | 0.001 | 0.001 | 0.001 | 0.001 | 0.001 | 0.001 | 0.002 | 0.002 | 0.002 | 0.001 | 0.001 | 0.001 | 0.002 | 0.001 | 0.002 |
| meta2414 |  | 956.551 | 128.904 | 0.005 | 0.004 | 0.006 | 0.003 | 0.003 | 0.002 | 0.002 | 0.003 | 0.003 | 0.004 | 0.003 | 0.003 | 0.003 | 0.002 | 0.003 |
| meta2415 |  | 958.598 | 104.822 | 0.017 | 0.015 | 0.014 | 0.017 | 0.008 | 0.010 | 0.006 | 0.012 | 0.012 | 0.011 | 0.008 | 0.016 | 0.008 | 0.011 | 0.006 |
| meta2416 |  | 959.484 | 45.351 | 0.001 | 0.002 | 0.002 | 0.002 | 0.003 | 0.002 | 0.002 | 0.002 | 0.002 | 0.001 | 0.001 | 0.001 | 0.003 | 0.003 | 0.003 |
| meta2417 |  | 960.627 | 171.086 | 0.000 | 0.000 | 0.000 | 0.001 | 0.001 | 0.000 | 0.001 | 0.001 | 0.000 | 0.000 | 0.001 | 0.000 | 0.000 | 0.000 | 0.001 |
| meta2418 |  | 962.558 | 46.019 | 0.002 | 0.002 | 0.002 | 0.001 | 0.000 | 0.001 | 0.001 | 0.001 | 0.001 | 0.001 | 0.001 | 0.001 | 0.001 | 0.001 | 0.001 |
| meta2419 |  | 966.450 | 37.868 | 0.000 | 0.001 | 0.001 | 0.001 | 0.000 | 0.001 | 0.000 | 0.001 | 0.001 | 0.001 | 0.000 | 0.001 | 0.001 | 0.001 | 0.000 |
| meta2420 |  | 968.466 | 45.951 | 0.001 | 0.001 | 0.001 | 0.001 | 0.000 | 0.000 | 0.000 | 0.001 | 0.001 | 0.001 | 0.001 | 0.001 | 0.000 | 0.001 | 0.000 |
| meta2421 |  | 968.577 | 98.082 | 0.021 | 0.020 | 0.017 | 0.001 | 0.001 | 0.001 | 0.000 | 0.040 | 0.029 | 0.019 | 0.005 | 0.015 | 0.016 | 0.002 | 0.000 |
| meta2422 |  | 970.525 | 174.061 | 0.001 | 0.001 | 0.001 | 0.000 | 0.000 | 0.000 | 0.000 | 0.000 | 0.000 | 0.000 | 0.001 | 0.001 | 0.000 | 0.000 | 0.000 |
| meta2423 |  | 971.606 | 173.531 | 0.001 | 0.000 | 0.001 | 0.001 | 0.001 | 0.001 | 0.001 | 0.001 | 0.000 | 0.001 | 0.001 | 0.001 | 0.001 | 0.001 | 0.001 |
| meta2424 |  | 972.367 | 247.108 | 0.000 | 0.000 | 0.000 | 0.000 | 0.000 | 0.000 | 0.000 | 0.000 | 0.000 | 0.000 | 0.000 | 0.000 | 0.000 | 0.000 | 0.000 |
| meta2425 |  | 972.605 | 98.008 | 0.006 | 0.005 | 0.005 | 0.002 | 0.001 | 0.001 | 0.000 | 0.006 | 0.004 | 0.003 | 0.002 | 0.003 | 0.003 | 0.001 | 0.000 |
| meta2426 |  | 975.115 | 420.274 | 0.001 | 0.001 | 0.001 | 0.001 | 0.001 | 0.001 | 0.001 | 0.001 | 0.001 | 0.001 | 0.001 | 0.001 | 0.001 | 0.001 | 0.001 |
| meta2427 |  | 975.618 | 46.558 | 0.000 | 0.000 | 0.000 | 0.000 | 0.000 | 0.000 | 0.001 | 0.000 | 0.000 | 0.000 | 0.000 | 0.001 | 0.000 | 0.000 | 0.001 |
| meta2428 |  | 975.639 | 176.615 | 0.000 | 0.000 | 0.000 | 0.001 | 0.000 | 0.000 | 0.001 | 0.001 | 0.000 | 0.000 | 0.001 | 0.001 | 0.000 | 0.000 | 0.000 |
| meta2429 |  | 982.515 | 157.949 | 0.001 | 0.001 | 0.001 | 0.001 | 0.000 | 0.000 | 0.000 | 0.000 | 0.000 | 0.000 | 0.000 | 0.001 | 0.000 | 0.000 | 0.000 |
| meta2430 |  | 982.599 | 104.184 | 0.018 | 0.014 | 0.015 | 0.019 | 0.013 | 0.013 | 0.011 | 0.016 | 0.016 | 0.010 | 0.013 | 0.013 | 0.013 | 0.015 | 0.008 |
| meta2431 |  | 986.628 | 104.124 | 0.004 | 0.003 | 0.005 | 0.002 | 0.001 | 0.001 | 0.001 | 0.002 | 0.003 | 0.002 | 0.004 | 0.004 | 0.002 | 0.002 | 0.002 |
| meta2432 |  | 986.682 | 287.112 | 0.000 | 0.000 | 0.000 | 0.000 | 0.000 | 0.000 | 0.000 | 0.000 | 0.000 | 0.000 | 0.000 | 0.001 | 0.000 | 0.000 | 0.000 |
| meta2433 |  | 987.300 | 477.012 | 0.001 | 0.001 | 0.001 | 0.001 | 0.001 | 0.001 | 0.001 | 0.001 | 0.001 | 0.001 | 0.001 | 0.001 | 0.001 | 0.001 | 0.001 |
| meta2434 |  | 989.317 | 493.912 | 0.001 | 0.001 | 0.001 | 0.001 | 0.001 | 0.001 | 0.001 | 0.001 | 0.001 | 0.001 | 0.001 | 0.001 | 0.001 | 0.001 | 0.001 |
| meta2435 |  | 989.653 | 174.343 | 0.001 | 0.001 | 0.001 | 0.001 | 0.001 | 0.001 | 0.002 | 0.001 | 0.000 | 0.001 | 0.001 | 0.001 | 0.001 | 0.001 | 0.001 |
| meta2436 |  | 990.643 | 287.923 | 0.001 | 0.000 | 0.000 | 0.001 | 0.001 | 0.000 | 0.001 | 0.001 | 0.000 | 0.000 | 0.001 | 0.001 | 0.001 | 0.001 | 0.001 |
| meta2437 |  | 991.206 | 460.902 | 0.001 | 0.001 | 0.001 | 0.001 | 0.001 | 0.001 | 0.001 | 0.001 | 0.001 | 0.001 | 0.001 | 0.001 | 0.001 | 0.001 | 0.001 |
| meta2438 |  | 991.265 | 426.819 | 0.000 | 0.000 | 0.000 | 0.000 | 0.000 | 0.001 | 0.000 | 0.000 | 0.000 | 0.000 | 0.000 | 0.000 | 0.000 | 0.000 | 0.000 |
| meta2439 |  | 992.472 | 45.935 | 0.000 | 0.000 | 0.000 | 0.000 | 0.000 | 0.000 | 0.000 | 0.000 | 0.000 | 0.000 | 0.000 | 0.000 | 0.000 | 0.000 | 0.000 |
| meta2440 |  | 992.576 | 98.334 | 0.002 | 0.002 | 0.002 | 0.001 | 0.001 | 0.000 | 0.000 | 0.005 | 0.005 | 0.002 | 0.001 | 0.001 | 0.003 | 0.000 | 0.000 |
| meta2441 |  | 992.623 | 288.205 | 0.000 | 0.000 | 0.001 | 0.001 | 0.000 | 0.000 | 0.000 | 0.000 | 0.000 | 0.000 | 0.000 | 0.000 | 0.001 | 0.000 | 0.000 |
| meta2442 |  | 994.563 | 124.896 | 0.009 | 0.009 | 0.007 | 0.016 | 0.015 | 0.011 | 0.009 | 0.009 | 0.010 | 0.011 | 0.007 | 0.005 | 0.010 | 0.007 | 0.014 |
| meta2443 |  | 994.606 | 288.670 | 0.000 | 0.000 | 0.000 | 0.001 | 0.000 | 0.000 | 0.000 | 0.000 | 0.000 | 0.000 | 0.000 | 0.000 | 0.000 | 0.000 | 0.000 |
| meta2444 |  | 994.934 | 686.995 | 0.002 | 0.002 | 0.003 | 0.003 | 0.005 | 0.003 | 0.001 | 0.003 | 0.002 | 0.003 | 0.002 | 0.002 | 0.002 | 0.002 | 0.003 |
| meta2445 |  | 995.606 | 174.993 | 0.001 | 0.001 | 0.002 | 0.003 | 0.002 | 0.002 | 0.003 | 0.002 | 0.002 | 0.002 | 0.002 | 0.002 | 0.003 | 0.003 | 0.002 |
| meta2446 |  | 997.097 | 420.311 | 0.000 | 0.000 | 0.000 | 0.001 | 0.000 | 0.000 | 0.000 | 0.000 | 0.000 | 0.000 | 0.000 | 0.000 | 0.000 | 0.000 | 0.000 |
| meta2447 |  | 999.107 | 273.911 | 0.008 | 0.005 | 0.007 | 0.008 | 0.009 | 0.007 | 0.006 | 0.009 | 0.006 | 0.009 | 0.006 | 0.003 | 0.006 | 0.007 | 0.006 |
| meta2448 |  | 999.609 | 273.814 | 0.000 | 0.000 | 0.000 | 0.000 | 0.000 | 0.000 | 0.000 | 0.001 | 0.000 | 0.001 | 0.001 | 0.000 | 0.000 | 0.000 | 0.000 |
| meta2449 |  | 999.637 | 172.239 | 0.000 | 0.000 | 0.000 | 0.000 | 0.000 | 0.000 | 0.000 | 0.000 | 0.000 | 0.000 | 0.000 | 0.000 | 0.001 | 0.000 | 0.000 |
| meta2450 |  | 999.734 | 118.209 | 0.000 | 0.000 | 0.000 | 0.000 | 0.000 | 0.000 | 0.000 | 0.000 | 0.000 | 0.000 | 0.000 | 0.001 | 0.000 | 0.000 | 0.000 |
| meta2451 |  | 1000.622 | 150.340 | 0.004 | 0.003 | 0.003 | 0.003 | 0.002 | 0.002 | 0.003 | 0.003 | 0.002 | 0.001 | 0.003 | 0.004 | 0.002 | 0.002 | 0.002 |
| meta2452 | Palmitoyl CoA | 1004.333 | 330.822 | 0.001 | 0.000 | 0.001 | 0.001 | 0.001 | 0.000 | 0.001 | 0.001 | 0.000 | 0.000 | 0.000 | 0.001 | 0.000 | 0.001 | 0.001 |
| meta2453 |  | 1006.620 | 121.810 | 0.001 | 0.001 | 0.001 | 0.001 | 0.001 | 0.001 | 0.001 | 0.001 | 0.001 | 0.000 | 0.001 | 0.001 | 0.000 | 0.000 | 0.001 |
| meta2454 |  | 1007.327 | 428.124 | 0.001 | 0.001 | 0.001 | 0.001 | 0.001 | 0.003 | 0.001 | 0.002 | 0.001 | 0.001 | 0.001 | 0.002 | 0.001 | 0.001 | 0.000 |
| meta2455 |  | 1009.161 | 420.040 | 0.001 | 0.001 | 0.001 | 0.001 | 0.001 | 0.001 | 0.001 | 0.001 | 0.001 | 0.001 | 0.000 | 0.001 | 0.002 | 0.001 | 0.001 |
| meta2456 |  | 1009.612 | 172.863 | 0.001 | 0.000 | 0.001 | 0.000 | 0.000 | 0.001 | 0.002 | 0.001 | 0.000 | 0.001 | 0.001 | 0.000 | 0.001 | 0.001 | 0.000 |
| meta2457 |  | 1015.314 | 427.505 | 0.001 | 0.001 | 0.001 | 0.002 | 0.001 | 0.002 | 0.001 | 0.001 | 0.001 | 0.001 | 0.001 | 0.002 | 0.001 | 0.001 | 0.000 |
| meta2458 |  | 1018.563 | 92.156 | 0.004 | 0.004 | 0.004 | 0.011 | 0.003 | 0.003 | 0.000 | 0.004 | 0.010 | 0.005 | 0.003 | 0.006 | 0.003 | 0.003 | 0.001 |
| meta2459 |  | 1019.564 | 136.572 | 0.001 | 0.001 | 0.001 | 0.001 | 0.001 | 0.002 | 0.003 | 0.001 | 0.000 | 0.004 | 0.002 | 0.001 | 0.002 | 0.002 | 0.002 |
| meta2460 |  | 1019.608 | 174.840 | 0.002 | 0.002 | 0.003 | 0.004 | 0.004 | 0.003 | 0.005 | 0.004 | 0.003 | 0.003 | 0.004 | 0.003 | 0.006 | 0.005 | 0.004 |
| meta2461 |  | 1021.088 | 273.896 | 0.001 | 0.001 | 0.001 | 0.001 | 0.002 | 0.001 | 0.002 | 0.002 | 0.001 | 0.002 | 0.001 | 0.001 | 0.001 | 0.001 | 0.002 |
| meta2462 |  | 1023.150 | 458.629 | 0.003 | 0.003 | 0.003 | 0.003 | 0.003 | 0.002 | 0.003 | 0.003 | 0.003 | 0.003 | 0.003 | 0.003 | 0.004 | 0.003 | 0.004 |
| meta2463 |  | 1023.638 | 170.748 | 0.001 | 0.001 | 0.001 | 0.002 | 0.002 | 0.001 | 0.002 | 0.002 | 0.001 | 0.001 | 0.002 | 0.002 | 0.002 | 0.001 | 0.001 |
| meta2464 |  | 1023.733 | 117.753 | 0.001 | 0.000 | 0.000 | 0.001 | 0.001 | 0.000 | 0.000 | 0.001 | 0.001 | 0.000 | 0.001 | 0.001 | 0.001 | 0.000 | 0.001 |
| meta2465 |  | 1024.620 | 150.234 | 0.001 | 0.001 | 0.001 | 0.001 | 0.001 | 0.001 | 0.001 | 0.001 | 0.001 | 0.000 | 0.001 | 0.001 | 0.001 | 0.001 | 0.001 |
| meta2466 |  | 1024.632 | 287.726 | 0.000 | 0.000 | 0.000 | 0.000 | 0.000 | 0.000 | 0.000 | 0.000 | 0.000 | 0.000 | 0.000 | 0.000 | 0.000 | 0.000 | 0.000 |
| meta2467 |  | 1025.633 | 174.042 | 0.001 | 0.001 | 0.001 | 0.001 | 0.002 | 0.002 | 0.002 | 0.001 | 0.001 | 0.001 | 0.001 | 0.001 | 0.001 | 0.002 | 0.001 |
| meta2468 |  | 1027.154 | 464.921 | 0.002 | 0.001 | 0.002 | 0.002 | 0.002 | 0.002 | 0.002 | 0.002 | 0.002 | 0.002 | 0.001 | 0.002 | 0.003 | 0.002 | 0.002 |
| meta2469 |  | 1027.556 | 161.840 | 0.001 | 0.001 | 0.001 | 0.000 | 0.000 | 0.001 | 0.003 | 0.002 | 0.000 | 0.002 | 0.001 | 0.001 | 0.002 | 0.003 | 0.000 |
| meta2470 | Linoleoyl-CoA | 1028.334 | 330.177 | 0.002 | 0.001 | 0.001 | 0.002 | 0.002 | 0.001 | 0.001 | 0.001 | 0.001 | 0.002 | 0.001 | 0.002 | 0.001 | 0.001 | 0.002 |
| meta2471 |  | 1030.235 | 371.111 | 0.000 | 0.000 | 0.000 | 0.000 | 0.000 | 0.000 | 0.000 | 0.000 | 0.000 | 0.000 | 0.000 | 0.000 | 0.000 | 0.000 | 0.000 |
| meta2472 | Oleoyl-CoA | 1030.348 | 328.621 | 0.002 | 0.001 | 0.001 | 0.002 | 0.002 | 0.001 | 0.001 | 0.002 | 0.001 | 0.002 | 0.001 | 0.002 | 0.001 | 0.001 | 0.002 |
| meta2473 |  | 1030.621 | 121.562 | 0.001 | 0.001 | 0.001 | 0.001 | 0.001 | 0.001 | 0.001 | 0.001 | 0.001 | 0.001 | 0.001 | 0.001 | 0.001 | 0.001 | 0.001 |
| meta2474 |  | 1031.351 | 328.416 | 0.001 | 0.001 | 0.001 | 0.001 | 0.001 | 0.000 | 0.001 | 0.001 | 0.000 | 0.001 | 0.000 | 0.001 | 0.001 | 0.001 | 0.001 |
| meta2475 |  | 1033.611 | 167.032 | 0.000 | 0.000 | 0.000 | 0.000 | 0.000 | 0.000 | 0.001 | 0.001 | 0.000 | 0.001 | 0.000 | 0.001 | 0.001 | 0.001 | 0.000 |
| meta2476 |  | 1035.627 | 170.926 | 0.001 | 0.001 | 0.001 | 0.000 | 0.000 | 0.001 | 0.001 | 0.001 | 0.000 | 0.002 | 0.001 | 0.001 | 0.001 | 0.001 | 0.000 |
| meta2477 |  | 1036.974 | 85.394 | 0.002 | 0.002 | 0.002 | 0.004 | 0.005 | 0.003 | 0.005 | 0.002 | 0.001 | 0.002 | 0.003 | 0.001 | 0.002 | 0.002 | 0.003 |
| meta2478 |  | 1037.645 | 170.906 | 0.003 | 0.003 | 0.003 | 0.001 | 0.002 | 0.004 | 0.006 | 0.005 | 0.001 | 0.006 | 0.003 | 0.002 | 0.003 | 0.003 | 0.002 |
| meta2479 |  | 1040.177 | 417.535 | 0.000 | 0.000 | 0.000 | 0.000 | 0.000 | 0.000 | 0.000 | 0.001 | 0.001 | 0.000 | 0.000 | 0.000 | 0.001 | 0.000 | 0.001 |
| meta2480 |  | 1042.146 | 479.713 | 0.001 | 0.001 | 0.001 | 0.001 | 0.001 | 0.001 | 0.001 | 0.001 | 0.001 | 0.001 | 0.001 | 0.001 | 0.001 | 0.001 | 0.001 |
| meta2481 |  | 1043.607 | 168.989 | 0.000 | 0.001 | 0.001 | 0.001 | 0.001 | 0.001 | 0.002 | 0.001 | 0.000 | 0.001 | 0.001 | 0.001 | 0.001 | 0.001 | 0.001 |
| meta2482 |  | 1045.132 | 458.657 | 0.001 | 0.000 | 0.001 | 0.001 | 0.001 | 0.000 | 0.001 | 0.001 | 0.001 | 0.001 | 0.000 | 0.001 | 0.001 | 0.001 | 0.001 |
| meta2483 |  | 1045.617 | 169.504 | 0.000 | 0.000 | 0.000 | 0.000 | 0.000 | 0.000 | 0.001 | 0.000 | 0.000 | 0.000 | 0.000 | 0.000 | 0.001 | 0.000 | 0.000 |
| meta2484 |  | 1045.716 | 171.040 | 0.000 | 0.000 | 0.000 | 0.000 | 0.000 | 0.000 | 0.000 | 0.000 | 0.000 | 0.000 | 0.000 | 0.000 | 0.000 | 0.000 | 0.000 |
| meta2485 |  | 1046.600 | 182.541 | 0.005 | 0.004 | 0.006 | 0.000 | 0.001 | 0.011 | 0.016 | 0.005 | 0.001 | 0.010 | 0.003 | 0.001 | 0.012 | 0.015 | 0.002 |
| meta2486 |  | 1047.637 | 170.270 | 0.001 | 0.000 | 0.000 | 0.001 | 0.001 | 0.001 | 0.001 | 0.001 | 0.001 | 0.001 | 0.001 | 0.001 | 0.001 | 0.001 | 0.001 |
| meta2487 |  | 1047.658 | 174.815 | 0.000 | 0.000 | 0.000 | 0.001 | 0.001 | 0.000 | 0.001 | 0.002 | 0.000 | 0.000 | 0.000 | 0.000 | 0.000 | 0.001 | 0.001 |
| meta2488 |  | 1047.734 | 117.692 | 0.000 | 0.000 | 0.000 | 0.001 | 0.001 | 0.000 | 0.001 | 0.001 | 0.001 | 0.000 | 0.000 | 0.000 | 0.000 | 0.000 | 0.001 |
| meta2489 |  | 1049.675 | 174.478 | 0.015 | 0.015 | 0.015 | 0.025 | 0.025 | 0.017 | 0.042 | 0.020 | 0.010 | 0.012 | 0.015 | 0.017 | 0.013 | 0.019 | 0.018 |
| meta2490 |  | 1051.553 | 182.812 | 0.016 | 0.018 | 0.016 | 0.003 | 0.006 | 0.019 | 0.023 | 0.016 | 0.004 | 0.022 | 0.016 | 0.007 | 0.023 | 0.024 | 0.009 |
| meta2491 |  | 1051.553 | 161.214 | 0.005 | 0.006 | 0.005 | 0.000 | 0.000 | 0.006 | 0.007 | 0.014 | 0.001 | 0.018 | 0.004 | 0.003 | 0.010 | 0.013 | 0.002 |
| meta2492 |  | 1052.333 | 328.228 | 0.001 | 0.001 | 0.001 | 0.001 | 0.001 | 0.001 | 0.001 | 0.001 | 0.001 | 0.001 | 0.001 | 0.001 | 0.001 | 0.001 | 0.001 |
| meta2493 |  | 1052.650 | 150.291 | 0.001 | 0.001 | 0.001 | 0.000 | 0.000 | 0.000 | 0.001 | 0.001 | 0.000 | 0.000 | 0.001 | 0.001 | 0.000 | 0.000 | 0.001 |
| meta2494 |  | 1052.948 | 85.738 | 0.001 | 0.001 | 0.001 | 0.003 | 0.003 | 0.002 | 0.003 | 0.001 | 0.001 | 0.001 | 0.002 | 0.000 | 0.001 | 0.001 | 0.002 |
| meta2495 |  | 1053.637 | 96.352 | 0.002 | 0.001 | 0.001 | 0.002 | 0.000 | 0.001 | 0.000 | 0.001 | 0.001 | 0.001 | 0.001 | 0.002 | 0.001 | 0.001 | 0.000 |
| meta2496 |  | 1054.621 | 121.732 | 0.001 | 0.001 | 0.001 | 0.001 | 0.001 | 0.001 | 0.001 | 0.001 | 0.001 | 0.001 | 0.001 | 0.001 | 0.001 | 0.001 | 0.001 |
| meta2497 |  | 1055.597 | 163.719 | 0.000 | 0.000 | 0.000 | 0.000 | 0.000 | 0.000 | 0.002 | 0.000 | 0.000 | 0.000 | 0.001 | 0.000 | 0.000 | 0.000 | 0.000 |
| meta2498 |  | 1057.613 | 163.114 | 0.002 | 0.001 | 0.001 | 0.000 | 0.001 | 0.002 | 0.003 | 0.003 | 0.001 | 0.003 | 0.002 | 0.001 | 0.002 | 0.002 | 0.001 |
| meta2499 |  | 1062.160 | 416.935 | 0.000 | 0.000 | 0.000 | 0.000 | 0.000 | 0.000 | 0.000 | 0.001 | 0.001 | 0.001 | 0.000 | 0.000 | 0.001 | 0.000 | 0.000 |
| meta2500 |  | 1067.526 | 182.724 | 0.002 | 0.004 | 0.003 | 0.001 | 0.001 | 0.004 | 0.004 | 0.003 | 0.001 | 0.005 | 0.003 | 0.001 | 0.003 | 0.004 | 0.001 |
| meta2501 |  | 1067.528 | 160.598 | 0.001 | 0.001 | 0.001 | 0.000 | 0.000 | 0.001 | 0.001 | 0.002 | 0.000 | 0.003 | 0.001 | 0.000 | 0.002 | 0.002 | 0.000 |
| meta2502 |  | 1071.171 | 482.270 | 0.001 | 0.001 | 0.001 | 0.002 | 0.002 | 0.002 | 0.002 | 0.001 | 0.001 | 0.001 | 0.001 | 0.002 | 0.003 | 0.002 | 0.001 |
| meta2503 |  | 1071.312 | 200.063 | 0.002 | 0.002 | 0.002 | 0.003 | 0.005 | 0.003 | 0.004 | 0.002 | 0.001 | 0.001 | 0.002 | 0.003 | 0.002 | 0.003 | 0.002 |
| meta2504 |  | 1073.675 | 173.542 | 0.002 | 0.002 | 0.003 | 0.003 | 0.003 | 0.003 | 0.005 | 0.004 | 0.003 | 0.002 | 0.003 | 0.004 | 0.003 | 0.003 | 0.003 |
| meta2505 |  | 1081.612 | 162.478 | 0.001 | 0.001 | 0.001 | 0.000 | 0.000 | 0.001 | 0.003 | 0.003 | 0.000 | 0.002 | 0.001 | 0.001 | 0.002 | 0.001 | 0.000 |
| meta2506 |  | 1081.692 | 171.517 | 0.000 | 0.000 | 0.000 | 0.000 | 0.000 | 0.000 | 0.001 | 0.001 | 0.000 | 0.000 | 0.000 | 0.000 | 0.000 | 0.000 | 0.001 |
| meta2507 |  | 1087.586 | 109.966 | 0.001 | 0.002 | 0.002 | 0.004 | 0.003 | 0.003 | 0.001 | 0.002 | 0.002 | 0.002 | 0.002 | 0.001 | 0.002 | 0.002 | 0.001 |
| meta2508 |  | 1091.615 | 174.150 | 0.001 | 0.001 | 0.001 | 0.001 | 0.000 | 0.000 | 0.001 | 0.001 | 0.000 | 0.000 | 0.001 | 0.001 | 0.001 | 0.000 | 0.000 |
| meta2509 |  | 1093.293 | 200.067 | 0.002 | 0.002 | 0.002 | 0.002 | 0.003 | 0.002 | 0.003 | 0.001 | 0.001 | 0.001 | 0.002 | 0.002 | 0.002 | 0.002 | 0.002 |
| meta2510 |  | 1097.675 | 167.653 | 0.001 | 0.000 | 0.001 | 0.001 | 0.001 | 0.001 | 0.002 | 0.001 | 0.001 | 0.001 | 0.001 | 0.001 | 0.001 | 0.001 | 0.001 |
| meta2511 |  | 1101.640 | 97.584 | 0.001 | 0.001 | 0.001 | 0.001 | 0.000 | 0.001 | 0.000 | 0.000 | 0.001 | 0.001 | 0.000 | 0.001 | 0.000 | 0.001 | 0.000 |
| meta2512 |  | 1101.705 | 171.703 | 0.001 | 0.001 | 0.001 | 0.001 | 0.001 | 0.001 | 0.002 | 0.002 | 0.001 | 0.001 | 0.001 | 0.001 | 0.001 | 0.001 | 0.002 |
| meta2513 |  | 1103.498 | 160.598 | 0.000 | 0.000 | 0.000 | 0.000 | 0.000 | 0.000 | 0.001 | 0.001 | 0.000 | 0.001 | 0.000 | 0.000 | 0.001 | 0.001 | 0.000 |
| meta2514 |  | 1105.736 | 170.961 | 0.003 | 0.003 | 0.004 | 0.004 | 0.005 | 0.003 | 0.008 | 0.005 | 0.003 | 0.003 | 0.005 | 0.004 | 0.003 | 0.003 | 0.007 |
| meta2515 |  | 1108.602 | 97.311 | 0.001 | 0.001 | 0.001 | 0.000 | 0.000 | 0.000 | 0.000 | 0.003 | 0.002 | 0.001 | 0.000 | 0.000 | 0.001 | 0.000 | 0.000 |
| meta2516 |  | 1109.264 | 200.071 | 0.001 | 0.001 | 0.001 | 0.001 | 0.001 | 0.001 | 0.001 | 0.001 | 0.000 | 0.001 | 0.001 | 0.001 | 0.001 | 0.001 | 0.001 |
| meta2517 |  | 1115.275 | 200.141 | 0.001 | 0.001 | 0.001 | 0.001 | 0.001 | 0.001 | 0.001 | 0.001 | 0.001 | 0.001 | 0.001 | 0.001 | 0.001 | 0.001 | 0.001 |
| meta2518 |  | 1121.673 | 168.362 | 0.000 | 0.000 | 0.000 | 0.000 | 0.000 | 0.000 | 0.000 | 0.000 | 0.000 | 0.000 | 0.000 | 0.000 | 0.000 | 0.000 | 0.000 |
| meta2519 |  | 1123.685 | 170.533 | 0.000 | 0.000 | 0.000 | 0.000 | 0.000 | 0.000 | 0.001 | 0.001 | 0.000 | 0.000 | 0.000 | 0.000 | 0.000 | 0.000 | 0.000 |
| meta2520 |  | 1124.122 | 273.881 | 0.005 | 0.004 | 0.004 | 0.006 | 0.006 | 0.005 | 0.004 | 0.007 | 0.004 | 0.007 | 0.004 | 0.002 | 0.004 | 0.005 | 0.004 |
| meta2521 |  | 1125.705 | 170.322 | 0.001 | 0.001 | 0.001 | 0.001 | 0.002 | 0.001 | 0.002 | 0.001 | 0.001 | 0.001 | 0.002 | 0.002 | 0.001 | 0.001 | 0.002 |
| meta2522 | Theaflavin | 1127.250 | 419.108 | 0.000 | 0.000 | 0.000 | 0.000 | 0.000 | 0.000 | 0.000 | 0.000 | 0.000 | 0.001 | 0.000 | 0.000 | 0.000 | 0.000 | 0.000 |
| meta2523 |  | 1130.584 | 97.442 | 0.000 | 0.001 | 0.000 | 0.000 | 0.000 | 0.000 | 0.000 | 0.002 | 0.001 | 0.001 | 0.000 | 0.000 | 0.000 | 0.000 | 0.000 |
[truncated: 3,604 more chars]
