# Supplementary material for: A Study on Gentiana dahurica Fisch Ethanol Extract Alleviating Alcoholic Liver Disease in Mice: A Metabolomic Analysis of the Liver
Source: Evid Based Complement Alternat Med. 2021 Jun 29;2021:5569538. doi: 10.1155/2021/5569538 (PMC8260312; doi:10.1155/2021/5569538)
Supplement: Supplementary Materials — “Supplementary information 1” contains the PCA results and Pearson correlation analysis of quality control samples. “Supplementary information 2” provides information on the content of all metabolites. [file 5569538.f1.zip › 5569538.f1/Supplementary information 2 (Positive ion mode).docx]

| **#ID** | **MS2 name** | **m/z (mass-to-charge ratio)** | **Rt (retention time,s)** | **QC-1** | **QC-2** | **QC-3** | **Control-1** | **Control-2** | **Control-3** | **Control-4** | **Alcohol-1** | **Alcohol-2** | **Alcohol-3** | **Alcohol-4** | **Alcohol+**  **GDEE-1** | **Alcohol+**  **GDEE-2** | **Alcohol+**  **GDEE-3** | **Alcohol+**  **GDEE-4** |
| --- | --- | --- | --- | --- | --- | --- | --- | --- | --- | --- | --- | --- | --- | --- | --- | --- | --- | --- |
| meta1 | Urea | 61.039 | 101.650 | 0.266 | 0.321 | 0.299 | 0.309 | 0.361 | 0.284 | 0.347 | 0.401 | 0.382 | 0.348 | 0.293 | 0.317 | 0.251 | 0.266 | 0.318 |
| meta2 | Imidazole | 69.044 | 77.813 | 0.063 | 0.078 | 0.085 | 0.096 | 0.087 | 0.069 | 0.113 | 0.091 | 0.111 | 0.079 | 0.072 | 0.079 | 0.303 | 0.102 | 0.102 |
| meta3 | 2-Amino-2-methyl-1,3-propanediol | 70.064 | 238.204 | 0.059 | 0.065 | 0.059 | 0.063 | 0.067 | 0.050 | 0.074 | 0.083 | 0.070 | 0.056 | 0.057 | 0.060 | 0.061 | 0.050 | 0.055 |
| meta4 |  | 72.080 | 263.587 | 0.096 | 0.110 | 0.099 | 0.087 | 0.087 | 0.081 | 0.144 | 0.064 | 0.064 | 0.087 | 0.065 | 0.188 | 0.159 | 0.076 | 0.135 |
| meta5 | Pyrrolidine | 72.080 | 280.222 | 0.377 | 0.438 | 0.428 | 0.314 | 0.445 | 0.406 | 0.588 | 0.509 | 0.408 | 0.481 | 0.547 | 0.704 | 0.337 | 0.435 | 0.470 |
| meta6 |  | 74.095 | 220.501 | 0.082 | 0.139 | 0.148 | 0.080 | 0.128 | 0.108 | 0.101 | 0.169 | 0.142 | 0.099 | 0.079 | 0.085 | 0.099 | 0.096 | 0.072 |
| meta7 | Trimethylamine N-oxide | 76.074 | 310.204 | 0.078 | 0.093 | 0.091 | 0.063 | 0.148 | 0.081 | 0.045 | 0.176 | 0.124 | 0.065 | 0.069 | 0.022 | 0.046 | 0.024 | 0.132 |
| meta8 | Glutaraldehyde | 83.048 | 358.912 | 0.005 | 0.007 | 0.004 | 0.007 | 0.005 | 0.006 | 0.005 | 0.008 | 0.009 | 0.008 | 0.005 | 0.009 | 0.006 | 0.004 | 0.010 |
| meta9 | 1-Aminocyclopropanecarboxylic acid | 84.043 | 382.072 | 0.844 | 0.756 | 0.716 | 0.686 | 0.968 | 0.524 | 0.833 | 1.007 | 0.833 | 0.880 | 1.044 | 0.738 | 0.727 | 0.835 | 0.648 |
| meta10 | gamma-Aminobutryic acid | 86.059 | 333.702 | 0.132 | 0.148 | 0.137 | 0.153 | 0.111 | 0.136 | 0.130 | 0.227 | 0.129 | 0.171 | 0.176 | 0.111 | 0.077 | 0.092 | 0.098 |
| meta11 |  | 86.095 | 467.194 | 0.174 | 0.273 | 0.180 | 0.247 | 0.229 | 0.174 | 0.218 | 0.369 | 0.235 | 0.217 | 0.217 | 0.191 | 0.219 | 0.140 | 0.238 |
| meta12 | 4-Hydroxybutanoic acid lactone | 87.043 | 329.789 | 0.162 | 0.166 | 0.183 | 0.180 | 0.177 | 0.120 | 0.162 | 0.271 | 0.219 | 0.234 | 0.165 | 0.130 | 0.117 | 0.129 | 0.090 |
| meta13 | L-Alanine | 90.054 | 324.987 | 1.577 | 1.604 | 1.426 | 2.062 | 1.855 | 1.610 | 1.716 | 1.467 | 1.439 | 1.393 | 1.566 | 1.378 | 1.809 | 1.294 | 1.563 |
| meta14 | beta-Alanine | 90.053 | 341.485 | 0.140 | 0.158 | 0.139 | 0.173 | 0.188 | 0.126 | 0.169 | 0.149 | 0.122 | 0.142 | 0.141 | 0.137 | 0.153 | 0.105 | 0.152 |
| meta15 |  | 93.081 | 348.812 | 0.466 | 0.462 | 0.469 | 0.478 | 0.455 | 0.471 | 0.477 | 0.471 | 0.486 | 0.445 | 0.457 | 0.489 | 0.486 | 0.457 | 0.464 |
| meta16 |  | 94.064 | 264.819 | 0.144 | 0.133 | 0.113 | 0.151 | 0.104 | 0.138 | 0.124 | 0.132 | 0.175 | 0.100 | 0.097 | 0.043 | 0.055 | 0.077 | 0.053 |
| meta17 |  | 95.059 | 293.918 | 0.089 | 0.084 | 0.079 | 0.076 | 0.073 | 0.055 | 0.081 | 0.085 | 0.088 | 0.112 | 0.082 | 0.082 | 0.089 | 0.051 | 0.075 |
| meta18 | 2(1H)-Pyridinone | 96.043 | 62.225 | 0.373 | 0.694 | 0.527 | 1.205 | 1.407 | 1.077 | 0.279 | 0.401 | 0.987 | 0.364 | 0.418 | 1.220 | 1.137 | 1.136 | 0.407 |
| meta19 | .beta.-Cyano-L-alanine | 97.038 | 49.203 | 0.022 | 0.036 | 0.034 | 0.034 | 0.030 | 0.032 | 0.033 | 0.035 | 0.053 | 0.034 | 0.029 | 0.018 | 0.028 | 0.036 | 0.035 |
| meta20 |  | 99.054 | 308.345 | 0.109 | 0.128 | 0.124 | 0.125 | 0.134 | 0.102 | 0.139 | 0.115 | 0.161 | 0.111 | 0.127 | 0.135 | 0.145 | 0.126 | 0.133 |
| meta21 |  | 100.074 | 329.596 | 0.328 | 0.381 | 0.333 | 0.267 | 0.342 | 0.237 | 0.341 | 0.460 | 0.460 | 0.422 | 0.288 | 0.242 | 0.192 | 0.256 | 0.206 |
| meta22 | DL-2,4-Diaminobutyric acid | 101.069 | 352.975 | 0.219 | 0.210 | 0.190 | 0.203 | 0.243 | 0.168 | 0.239 | 0.231 | 0.212 | 0.208 | 0.226 | 0.181 | 0.237 | 0.181 | 0.211 |
| meta23 | 3-Butynoic acid | 102.054 | 46.855 | 0.041 | 0.062 | 0.060 | 0.105 | 0.047 | 0.045 | 0.065 | 0.057 | 0.072 | 0.104 | 0.051 | 0.063 | 0.057 | 0.049 | 0.119 |
| meta24 | Betaine aldehyde | 102.090 | 268.066 | 0.671 | 0.770 | 0.788 | 0.681 | 0.892 | 0.731 | 0.897 | 0.599 | 0.522 | 0.684 | 0.798 | 0.728 | 0.616 | 0.858 | 0.757 |
| meta25 | 2-Ketobutyric acid | 103.037 | 330.594 | 0.945 | 0.990 | 0.939 | 0.945 | 1.043 | 0.761 | 0.989 | 1.017 | 0.747 | 0.866 | 0.958 | 0.877 | 0.721 | 0.808 | 0.940 |
| meta26 |  | 103.053 | 238.952 | 0.158 | 0.133 | 0.137 | 0.107 | 0.162 | 0.119 | 0.210 | 0.180 | 0.109 | 0.152 | 0.187 | 0.158 | 0.122 | 0.097 | 0.149 |
| meta27 | Dimethylglycine | 104.070 | 279.991 | 0.721 | 0.822 | 0.756 | 1.036 | 0.689 | 0.824 | 0.415 | 0.592 | 0.658 | 0.635 | 0.883 | 0.496 | 0.432 | 0.494 | 0.720 |
| meta28 |  | 104.106 | 244.735 | 41.292 | 43.154 | 44.566 | 48.657 | 51.945 | 37.065 | 49.487 | 48.754 | 39.125 | 40.819 | 40.356 | 46.258 | 40.538 | 42.892 | 44.675 |
| meta29 |  | 104.106 | 362.157 | 4.321 | 4.334 | 3.944 | 3.845 | 4.548 | 2.712 | 5.145 | 3.781 | 2.734 | 3.987 | 4.810 | 3.814 | 3.161 | 2.573 | 5.226 |
| meta30 | L-Serine | 106.048 | 356.356 | 0.018 | 0.018 | 0.017 | 0.016 | 0.023 | 0.017 | 0.021 | 0.028 | 0.018 | 0.018 | 0.019 | 0.024 | 0.020 | 0.014 | 0.017 |
| meta31 |  | 107.047 | 41.781 | 0.040 | 0.061 | 0.050 | 0.054 | 0.053 | 0.049 | 0.054 | 0.055 | 0.061 | 0.053 | 0.066 | 0.059 | 0.051 | 0.057 | 0.060 |
| meta32 |  | 108.010 | 275.211 | 0.498 | 0.492 | 0.435 | 0.441 | 0.546 | 0.412 | 0.425 | 0.593 | 0.472 | 0.622 | 0.480 | 0.306 | 0.482 | 0.479 | 0.486 |
| meta33 |  | 110.033 | 157.435 | 0.157 | 0.183 | 0.159 | 0.171 | 0.205 | 0.177 | 0.183 | 0.121 | 0.127 | 0.116 | 0.164 | 0.166 | 0.161 | 0.183 | 0.143 |
| meta34 |  | 110.070 | 380.742 | 2.837 | 4.167 | 4.251 | 4.182 | 4.828 | 3.591 | 3.472 | 3.671 | 5.231 | 4.986 | 4.605 | 4.451 | 3.584 | 4.032 | 4.640 |
| meta35 | Internal standard | 110.107 | 348.621 | 1.000 | 1.000 | 1.000 | 1.000 | 1.000 | 1.000 | 1.000 | 1.000 | 1.000 | 1.000 | 1.000 | 1.000 | 1.000 | 1.000 | 1.000 |
| meta36 | Cytosine | 112.049 | 229.970 | 2.885 | 1.825 | 2.843 | 3.080 | 3.209 | 2.429 | 2.933 | 2.888 | 0.874 | 1.179 | 2.783 | 1.449 | 1.431 | 2.623 | 2.810 |
| meta37 | Uracil | 113.033 | 152.591 | 0.281 | 0.326 | 0.315 | 0.271 | 0.442 | 0.237 | 0.562 | 0.310 | 0.209 | 0.156 | 0.248 | 0.282 | 0.240 | 0.260 | 0.291 |
| meta38 | Creatinine | 114.065 | 157.435 | 1.723 | 1.826 | 1.583 | 1.796 | 1.955 | 1.640 | 1.969 | 1.522 | 1.281 | 1.488 | 1.430 | 1.326 | 1.327 | 1.265 | 1.559 |
| meta39 | Dihydrouracil | 115.048 | 324.994 | 0.070 | 0.060 | 0.061 | 0.088 | 0.082 | 0.072 | 0.072 | 0.052 | 0.060 | 0.047 | 0.068 | 0.061 | 0.066 | 0.043 | 0.063 |
| meta40 | L-Proline | 116.070 | 291.492 | 1.301 | 1.360 | 0.968 | 1.290 | 1.142 | 0.789 | 1.385 | 1.241 | 0.791 | 1.218 | 1.378 | 1.070 | 0.728 | 0.592 | 1.200 |
| meta41 | Glycine | 117.064 | 338.543 | 0.172 | 0.190 | 0.177 | 0.186 | 0.227 | 0.123 | 0.197 | 0.220 | 0.207 | 0.185 | 0.224 | 0.181 | 0.203 | 0.161 | 0.157 |
| meta42 | Guanidoacetic acid | 118.059 | 329.593 | 0.298 | 0.284 | 0.301 | 0.298 | 0.184 | 0.241 | 0.279 | 0.249 | 0.428 | 0.340 | 0.379 | 0.330 | 0.566 | 0.269 | 0.274 |
| meta43 | Betaine | 118.085 | 280.707 | 1.135 | 1.568 | 1.300 | 1.515 | 1.382 | 1.177 | 1.159 | 1.166 | 1.347 | 1.083 | 1.410 | 1.223 | 0.851 | 0.978 | 9.280 |
| meta44 | Methylmalonic acid | 119.033 | 234.455 | 0.763 | 0.728 | 0.535 | 0.625 | 0.704 | 0.540 | 0.493 | 0.750 | 0.512 | 0.584 | 0.832 | 0.592 | 0.493 | 0.714 | 0.858 |
| meta45 | 2-Methyl-3-hydroxybutyric acid | 119.071 | 224.646 | 0.073 | 0.073 | 0.067 | 0.058 | 0.067 | 0.094 | 0.069 | 0.073 | 0.080 | 0.081 | 0.071 | 0.065 | 0.075 | 0.069 | 0.073 |
| meta46 | L-Threonine | 120.064 | 336.043 | 0.245 | 0.259 | 0.196 | 0.201 | 0.186 | 0.204 | 0.284 | 0.295 | 0.157 | 0.227 | 0.256 | 0.200 | 0.211 | 0.163 | 0.245 |
| meta47 | Tyramine | 120.079 | 238.918 | 1.831 | 1.561 | 1.476 | 1.215 | 1.730 | 1.345 | 2.354 | 2.094 | 1.218 | 1.668 | 2.052 | 1.940 | 1.348 | 1.078 | 1.699 |
| meta48 |  | 120.101 | 267.977 | 3.305 | 3.658 | 3.668 | 3.106 | 4.343 | 3.394 | 4.246 | 2.756 | 2.449 | 3.204 | 3.659 | 3.432 | 2.707 | 4.068 | 3.485 |
| meta49 |  | 121.038 | 270.303 | 0.021 | 0.024 | 0.023 | 0.023 | 0.024 | 0.019 | 0.025 | 0.027 | 0.021 | 0.023 | 0.020 | 0.021 | 0.022 | 0.020 | 0.026 |
| meta50 | Erythritol | 122.058 | 705.842 | 0.069 | 0.081 | 0.072 | 0.073 | 0.077 | 0.060 | 0.015 | 0.079 | 0.016 | 0.019 | 0.076 | 0.068 | 0.068 | 0.070 | 0.074 |
| meta51 | Nicotinamide | 123.054 | 60.512 | 286.418 | 438.519 | 365.211 | 388.717 | 267.673 | 194.868 | 273.385 | 250.777 | 321.759 | 204.266 | 183.438 | 243.902 | 347.103 | 302.290 | 383.968 |
| meta52 | Picolinic acid | 124.038 | 205.470 | 0.017 | 0.025 | 0.025 | 0.024 | 0.035 | 0.025 | 0.018 | 0.019 | 0.022 | 0.022 | 0.035 | 0.024 | 0.016 | 0.023 | 0.018 |
| meta53 | Nitrosobenzene | 125.069 | 8.333 | 0.030 | 0.033 | 0.031 | 0.030 | 0.032 | 0.024 | 0.028 | 0.032 | 0.031 | 0.032 | 0.032 | 0.026 | 0.030 | 0.031 | 0.031 |
| meta54 | Taurine | 126.021 | 275.147 | 9.076 | 8.506 | 7.937 | 7.803 | 9.528 | 7.173 | 7.243 | 11.100 | 8.422 | 11.482 | 8.414 | 5.256 | 8.281 | 8.508 | 8.471 |
| meta55 |  | 126.090 | 270.126 | 0.238 | 0.268 | 0.205 | 0.368 | 0.322 | 0.208 | 0.320 | 0.108 | 0.148 | 0.127 | 0.172 | 0.096 | 0.259 | 0.123 | 0.184 |
| meta56 |  | 127.037 | 393.051 | 0.024 | 0.019 | 0.029 | 0.019 | 0.047 | 0.015 | 0.024 | 0.021 | 0.021 | 0.016 | 0.032 | 0.019 | 0.034 | 0.019 | 0.019 |
| meta57 | Imidazoleacetic acid | 127.049 | 305.445 | 0.073 | 0.074 | 0.067 | 0.067 | 0.077 | 0.058 | 0.082 | 0.099 | 0.089 | 0.096 | 0.086 | 0.105 | 0.066 | 0.076 | 0.075 |
| meta58 |  | 128.952 | 318.614 | 0.997 | 0.957 | 1.184 | 0.894 | 0.954 | 1.189 | 1.030 | 0.943 | 1.155 | 0.989 | 1.003 | 0.928 | 1.268 | 1.359 | 0.972 |
| meta59 |  | 129.064 | 336.636 | 0.615 | 0.637 | 0.616 | 0.766 | 0.733 | 0.601 | 0.524 | 0.632 | 0.660 | 0.664 | 0.653 | 0.496 | 0.563 | 0.485 | 0.571 |
| meta60 | .beta.-Homoproline | 130.084 | 305.630 | 0.208 | 0.221 | 0.193 | 0.186 | 0.222 | 0.157 | 0.232 | 0.308 | 0.210 | 0.251 | 0.281 | 0.194 | 0.196 | 0.176 | 0.200 |
| meta61 |  | 130.096 | 238.195 | 0.061 | 0.064 | 0.058 | 0.068 | 0.075 | 0.049 | 0.063 | 0.088 | 0.068 | 0.063 | 0.064 | 0.069 | 0.058 | 0.057 | 0.062 |
| meta62 |  | 130.168 | 27.382 | 0.009 | 0.010 | 0.010 | 0.010 | 0.010 | 0.007 | 0.009 | 0.014 | 0.010 | 0.007 | 0.010 | 0.011 | 0.010 | 0.011 | 0.011 |
| meta63 | Phenyllactic acid | 131.047 | 238.872 | 0.026 | 0.021 | 0.023 | 0.018 | 0.023 | 0.020 | 0.033 | 0.027 | 0.019 | 0.022 | 0.030 | 0.028 | 0.021 | 0.017 | 0.024 |
| meta64 | DL-3-Phenyllactic acid | 131.047 | 166.336 | 0.006 | 0.009 | 0.008 | 0.008 | 0.002 | 0.007 | 0.006 | 0.015 | 0.011 | 0.013 | 0.012 | 0.004 | 0.002 | 0.004 | 0.003 |
| meta65 | Atrolactic acid | 131.051 | 295.161 | 0.028 | 0.026 | 0.022 | 0.024 | 0.028 | 0.019 | 0.024 | 0.033 | 0.021 | 0.032 | 0.036 | 0.026 | 0.021 | 0.017 | 0.029 |
| meta66 |  | 131.678 | 325.169 | 0.094 | 0.068 | 0.065 | 0.109 | 0.107 | 0.082 | 0.089 | 0.074 | 0.082 | 0.078 | 0.057 | 0.076 | 0.083 | 0.073 | 0.067 |
| meta67 | Creatine | 132.076 | 325.018 | 32.439 | 30.790 | 27.468 | 41.553 | 38.045 | 32.369 | 31.932 | 25.541 | 28.178 | 27.319 | 30.861 | 25.970 | 35.087 | 26.617 | 30.590 |
| meta68 |  | 132.100 | 240.838 | 0.886 | 0.943 | 0.922 | 1.260 | 1.104 | 1.224 | 0.978 | 0.820 | 0.636 | 0.729 | 1.140 | 0.670 | 0.608 | 0.469 | 1.678 |
| meta69 | L-Leucine | 132.100 | 152.070 | 0.016 | 0.011 | 0.011 | 0.014 | 0.010 | 0.012 | 0.009 | 0.009 | 0.008 | 0.010 | 0.012 | 0.008 | 0.010 | 0.008 | 0.010 |
| meta70 | 3-Ureidopropionate | 133.059 | 302.435 | 0.038 | 0.037 | 0.035 | 0.033 | 0.051 | 0.027 | 0.043 | 0.035 | 0.025 | 0.032 | 0.034 | 0.031 | 0.047 | 0.027 | 0.039 |
| meta71 | D-Aspartic acid | 134.043 | 389.040 | 0.065 | 0.061 | 0.057 | 0.053 | 0.061 | 0.054 | 0.057 | 0.066 | 0.073 | 0.082 | 0.068 | 0.071 | 0.073 | 0.051 | 0.061 |
| meta72 | Methyl acetoacetate | 134.079 | 324.994 | 0.115 | 0.150 | 0.138 | 0.179 | 0.196 | 0.148 | 0.129 | 0.115 | 0.143 | 0.126 | 0.119 | 0.108 | 0.151 | 0.128 | 0.150 |
| meta73 | 3-Hydroxyphenylacetic acid | 135.042 | 304.464 | 0.032 | 0.040 | 0.037 | 0.037 | 0.040 | 0.029 | 0.037 | 0.038 | 0.043 | 0.036 | 0.035 | 0.035 | 0.034 | 0.035 | 0.038 |
| meta74 | Linalool oxide | 135.119 | 245.219 | 0.247 | 0.215 | 0.230 | 0.184 | 0.220 | 0.225 | 0.223 | 0.234 | 0.231 | 0.270 | 0.241 | 0.226 | 0.242 | 0.214 | 0.259 |
| meta75 |  | 136.020 | 627.564 | 1.613 | 1.669 | 1.615 | 0.361 | 2.165 | 1.133 | 0.168 | 1.804 | 1.555 | 1.396 | 1.991 | 1.303 | 1.466 | 1.454 | 0.209 |
| meta76 | Adenine | 136.061 | 149.595 | 3.965 | 4.520 | 4.171 | 4.974 | 4.968 | 4.306 | 5.637 | 4.786 | 7.034 | 4.662 | 3.866 | 3.277 | 3.924 | 4.783 | 4.767 |
| meta77 |  | 136.632 | 201.986 | 0.057 | 0.061 | 0.059 | 0.054 | 0.075 | 0.057 | 0.069 | 0.052 | 0.041 | 0.048 | 0.045 | 0.056 | 0.052 | 0.071 | 0.054 |
| meta78 |  | 136.938 | 287.416 | 0.321 | 0.258 | 0.272 | 0.330 | 0.364 | 0.293 | 0.320 | 0.319 | 0.406 | 0.366 | 0.347 | 0.337 | 0.460 | 0.329 | 0.337 |
| meta79 | Hypoxanthine | 137.045 | 157.403 | 14.800 | 15.492 | 14.025 | 15.075 | 19.106 | 15.543 | 17.414 | 11.010 | 10.538 | 10.583 | 13.372 | 15.648 | 13.362 | 15.160 | 13.473 |
| meta80 | L-homoserine | 137.094 | 34.151 | 0.035 | 0.023 | 0.024 | 0.027 | 0.028 | 0.022 | 0.026 | 0.024 | 0.024 | 0.038 | 0.024 | 0.028 | 0.022 | 0.022 | 0.028 |
| meta81 |  | 137.105 | 91.406 | 0.071 | 0.062 | 0.067 | 0.134 | 0.245 | 0.111 | 0.284 | 0.082 | 0.079 | 0.068 | 0.075 | 0.185 | 0.174 | 0.207 | 0.287 |
| meta82 |  | 137.638 | 264.834 | 0.040 | 0.058 | 0.039 | 0.042 | 0.033 | 0.043 | 0.053 | 0.043 | 0.056 | 0.030 | 0.035 | 0.009 | 0.014 | 0.020 | 0.014 |
| meta83 |  | 138.020 | 200.752 | 0.011 | 0.011 | 0.008 | 0.899 | 0.041 | 0.877 | 0.028 | 0.828 | 0.608 | 0.723 | 0.889 | 1.000 | 0.014 | 0.005 | 0.834 |
| meta84 | Anthranilic acid (Vitamin L1) | 138.054 | 265.133 | 17.439 | 17.953 | 13.603 | 20.052 | 11.951 | 15.078 | 17.746 | 15.599 | 21.553 | 11.257 | 13.742 | 3.858 | 7.917 | 6.739 | 4.911 |
| meta85 |  | 139.049 | 81.950 | 0.265 | 0.350 | 0.320 | 0.443 | 0.350 | 0.434 | 0.258 | 0.256 | 0.341 | 0.309 | 0.450 | 0.347 | 0.185 | 0.260 | 0.121 |
| meta86 | Urocanic acid | 139.049 | 157.410 | 0.045 | 0.046 | 0.037 | 0.046 | 0.051 | 0.045 | 0.069 | 0.031 | 0.033 | 0.040 | 0.036 | 0.039 | 0.040 | 0.041 | 0.044 |
| meta87 | 6-Hydroxynicotinic acid | 140.032 | 81.950 | 0.013 | 0.026 | 0.021 | 0.027 | 0.024 | 0.029 | 0.017 | 0.019 | 0.021 | 0.020 | 0.031 | 0.023 | 0.012 | 0.016 | 0.007 |
| meta88 |  | 140.066 | 252.603 | 0.651 | 0.716 | 0.717 | 0.779 | 0.709 | 0.667 | 0.550 | 0.620 | 0.669 | 0.459 | 0.667 | 0.523 | 0.366 | 0.443 | 0.726 |
| meta89 |  | 141.010 | 352.027 | 0.040 | 0.050 | 0.044 | 0.036 | 0.043 | 0.029 | 0.046 | 0.054 | 0.074 | 0.060 | 0.051 | 0.052 | 0.054 | 0.049 | 0.059 |
| meta90 |  | 141.064 | 294.206 | 0.374 | 0.395 | 0.342 | 0.310 | 0.274 | 0.286 | 0.377 | 0.537 | 0.436 | 0.584 | 0.316 | 0.462 | 0.473 | 0.283 | 0.360 |
| meta91 | O-Phosphoethanolamine | 142.025 | 457.924 | 0.017 | 0.020 | 0.015 | 0.015 | 0.018 | 0.021 | 0.018 | 0.012 | 0.017 | 0.015 | 0.017 | 0.017 | 0.021 | 0.016 | 0.017 |
| meta92 |  | 143.116 | 270.303 | 0.105 | 0.123 | 0.093 | 0.152 | 0.139 | 0.098 | 0.153 | 0.046 | 0.068 | 0.053 | 0.071 | 0.049 | 0.110 | 0.060 | 0.076 |
| meta93 |  | 144.046 | 313.287 | 0.280 | 0.335 | 0.306 | 0.326 | 0.355 | 0.288 | 0.362 | 0.356 | 0.341 | 0.292 | 0.284 | 0.346 | 0.327 | 0.296 | 0.331 |
| meta94 |  | 144.046 | 43.585 | 0.108 | 0.123 | 0.123 | 0.124 | 0.102 | 0.077 | 0.054 | 0.002 | 0.136 | 0.165 | 0.194 | 0.148 | 0.107 | 0.077 | 0.162 |
| meta95 | 4-Hydroxy-6-methylpyran-2-one | 144.064 | 192.511 | 0.025 | 0.023 | 0.022 | 0.045 | 0.024 | 0.015 | 0.022 | 0.024 | 0.020 | 0.029 | 0.026 | 0.031 | 0.021 | 0.023 | 0.040 |
| meta96 | 1-Aminocyclohexanecarboxylic acid | 144.100 | 334.759 | 0.164 | 0.194 | 0.134 | 0.202 | 0.268 | 0.139 | 0.158 | 0.249 | 0.142 | 0.137 | 0.150 | 0.094 | 0.084 | 0.101 | 0.125 |
| meta97 |  | 144.100 | 454.765 | 0.074 | 0.075 | 0.076 | 0.072 | 0.078 | 0.078 | 0.077 | 0.077 | 0.083 | 0.070 | 0.075 | 0.079 | 0.104 | 0.080 | 0.083 |
| meta98 | 5-Hydroxy-L-lysine | 145.095 | 351.622 | 0.015 | 0.024 | 0.022 | 0.015 | 0.014 | 0.010 | 0.019 | 0.020 | 0.011 | 0.017 | 0.014 | 0.017 | 0.014 | 0.016 | 0.015 |
| meta99 |  | 146.025 | 215.081 | 0.065 | 0.064 | 0.062 | 0.080 | 0.031 | 0.063 | 0.027 | 0.074 | 0.041 | 0.066 | 0.127 | 0.068 | 0.040 | 0.053 | 0.099 |
| meta100 | Oxyquinoline | 146.058 | 43.816 | 0.051 | 0.064 | 0.064 | 0.061 | 0.059 | 0.073 | 0.066 | 0.067 | 0.061 | 0.064 | 0.074 | 0.060 | 0.053 | 0.054 | 0.061 |
| meta101 |  | 146.079 | 311.453 | 0.340 | 0.320 | 0.302 | 0.325 | 0.337 | 0.288 | 0.283 | 0.318 | 0.326 | 0.377 | 0.286 | 0.280 | 0.355 | 0.302 | 0.288 |
| meta102 | 4-Guanidinobutyric acid | 146.091 | 333.506 | 4.098 | 4.290 | 3.782 | 4.611 | 3.139 | 3.605 | 3.725 | 6.483 | 4.250 | 6.157 | 5.927 | 3.069 | 2.493 | 2.428 | 2.948 |
| meta103 |  | 146.116 | 352.712 | 4.099 | 4.124 | 3.713 | 4.979 | 4.778 | 3.552 | 3.815 | 4.966 | 3.126 | 3.944 | 3.717 | 3.551 | 2.958 | 2.332 | 3.853 |
| meta104 |  | 146.116 | 37.422 | 0.110 | 0.130 | 0.121 | 0.143 | 0.147 | 0.113 | 0.140 | 0.104 | 0.073 | 0.105 | 0.103 | 0.089 | 0.118 | 0.093 | 0.170 |
| meta105 | Phenylpyruvate | 147.042 | 279.877 | 0.015 | 0.014 | 0.014 | 0.012 | 0.012 | 0.015 | 0.016 | 0.015 | 0.010 | 0.014 | 0.014 | 0.015 | 0.015 | 0.013 | 0.012 |
| meta106 | Diacetyl | 147.063 | 268.643 | 0.008 | 0.006 | 0.006 | 0.010 | 0.004 | 0.007 | 0.011 | 0.007 | 0.006 | 0.007 | 0.008 | 0.009 | 0.008 | 0.008 | 0.010 |
| meta107 | L-Glutamine | 147.074 | 369.142 | 0.559 | 0.560 | 0.577 | 0.600 | 0.585 | 0.494 | 0.608 | 0.685 | 0.678 | 0.572 | 0.565 | 0.493 | 0.676 | 0.622 | 0.632 |
| meta108 | L-Lysine | 147.111 | 484.872 | 0.102 | 0.177 | 0.113 | 0.071 | 0.076 | 0.185 | 0.097 | 0.067 | 0.162 | 0.100 | 0.071 | 0.123 | 0.193 | 0.153 | 0.171 |
| meta109 | Formylanthranilic acid | 148.037 | 44.001 | 0.044 | 0.049 | 0.047 | 0.057 | 0.064 | 0.053 | 0.064 | 0.061 | 0.039 | 0.042 | 0.055 | 0.047 | 0.046 | 0.036 | 0.039 |
| meta110 |  | 148.041 | 344.800 | 0.123 | 0.135 | 0.124 | 0.156 | 0.139 | 0.145 | 0.158 | 0.127 | 0.120 | 0.114 | 0.136 | 0.150 | 0.103 | 0.125 | 0.137 |
| meta111 |  | 148.095 | 270.510 | 0.099 | 0.104 | 0.099 | 0.199 | 0.132 | 0.163 | 0.048 | 0.070 | 0.068 | 0.064 | 0.051 | 0.031 | 0.033 | 0.026 | 0.044 |
| meta112 |  | 149.034 | 28.102 | 0.313 | 0.430 | 0.384 | 0.789 | 0.434 | 0.256 | 0.360 | 0.653 | 0.387 | 0.356 | 0.414 | 0.565 | 0.395 | 0.684 | 0.405 |
| meta113 | Citramalic acid | 149.044 | 344.660 | 0.225 | 0.254 | 0.225 | 0.007 | 0.005 | 0.005 | 0.008 | 0.231 | 0.211 | 0.004 | 0.006 | 0.006 | 0.217 | 0.218 | 0.006 |
| meta114 | trans-cinnamate | 149.058 | 238.873 | 0.036 | 0.028 | 0.027 | 0.023 | 0.035 | 0.026 | 0.043 | 0.039 | 0.025 | 0.030 | 0.039 | 0.034 | 0.024 | 0.021 | 0.031 |
| meta115 | Dihydroxyfumarate | 149.079 | 352.975 | 0.049 | 0.057 | 0.050 | 0.055 | 0.052 | 0.043 | 0.057 | 0.055 | 0.053 | 0.053 | 0.049 | 0.046 | 0.064 | 0.046 | 0.045 |
| meta116 |  | 149.073 | 51.674 | 0.096 | 0.093 | 0.085 | 0.002 | 0.137 | 0.100 | 0.139 | 0.016 | 0.167 | 0.139 | 0.005 | 0.098 | 0.053 | 0.098 | 0.004 |
| meta117 | L-Arabinose | 150.053 | 96.976 | 0.092 | 0.153 | 0.122 | 0.146 | 0.136 | 0.157 | 0.120 | 0.098 | 0.151 | 0.122 | 0.096 | 0.086 | 0.104 | 0.120 | 0.110 |
| meta118 | Triethanolamine | 150.111 | 141.266 | 0.092 | 0.091 | 0.076 | 0.112 | 0.074 | 0.101 | 0.064 | 0.090 | 0.041 | 0.034 | 0.037 | 0.026 | 0.042 | 0.047 | 0.039 |
| meta119 | Methoxyacetic acid | 151.059 | 265.996 | 0.012 | 0.008 | 0.011 | 0.020 | 0.003 | 0.016 | 0.013 | 0.021 | 0.018 | 0.013 | 0.013 | 0.024 | 0.020 | 0.009 | 0.010 |
| meta120 |  | 151.133 | 319.812 | 0.020 | 0.018 | 0.015 | 0.014 | 0.013 | 0.015 | 0.014 | 0.019 | 0.026 | 0.024 | 0.023 | 0.025 | 0.024 | 0.019 | 0.018 |
| meta121 |  | 152.030 | 353.635 | 0.180 | 0.190 | 0.170 | 0.183 | 0.183 | 0.144 | 0.174 | 0.191 | 0.190 | 0.169 | 0.181 | 0.161 | 0.195 | 0.156 | 0.183 |
| meta122 | 2-Methoxybenzoic acid | 152.046 | 322.239 | 0.017 | 0.017 | 0.015 | 0.013 | 0.014 | 0.013 | 0.019 | 0.019 | 0.014 | 0.015 | 0.012 | 0.012 | 0.014 | 0.012 | 0.013 |
| meta123 | 2-Hydroxyadenine | 152.055 | 210.664 | 0.042 | 0.039 | 0.038 | 0.033 | 0.050 | 0.036 | 0.045 | 0.043 | 0.039 | 0.039 | 0.034 | 0.038 | 0.043 | 0.032 | 0.043 |
| meta124 | N-Methylanthranilic Acid | 152.069 | 235.765 | 0.156 | 0.171 | 0.099 | 0.073 | 0.115 | 0.054 | 0.078 | 0.067 | 0.063 | 0.183 | 0.150 | 0.162 | 0.208 | 0.136 | 0.191 |
| meta125 | Pyrocatechol | 152.069 | 258.184 | 0.047 | 0.047 | 0.050 | 0.045 | 0.038 | 0.035 | 0.048 | 0.070 | 0.095 | 0.061 | 0.049 | 0.037 | 0.046 | 0.042 | 0.039 |
| meta126 |  | 152.080 | 298.755 | 0.082 | 0.084 | 0.070 | 0.058 | 0.078 | 0.053 | 0.084 | 0.079 | 0.066 | 0.101 | 0.101 | 0.088 | 0.067 | 0.046 | 0.085 |
| meta127 |  | 152.117 | 315.574 | 0.035 | 0.041 | 0.040 | 0.028 | 0.045 | 0.025 | 0.038 | 0.044 | 0.058 | 0.048 | 0.043 | 0.042 | 0.038 | 0.041 | 0.045 |
| meta128 | Xanthine | 153.039 | 199.422 | 0.089 | 0.103 | 0.090 | 0.104 | 0.138 | 0.092 | 0.131 | 0.084 | 0.066 | 0.071 | 0.075 | 0.070 | 0.104 | 0.094 | 0.084 |
| meta129 |  | 154.040 | 224.463 | 2.114 | 2.061 | 1.978 | 1.739 | 2.053 | 2.537 | 2.249 | 2.255 | 2.456 | 2.409 | 2.107 | 2.130 | 2.225 | 2.008 | 2.182 |
| meta130 |  | 154.084 | 99.237 | 0.104 | 0.123 | 0.107 | 0.082 | 0.119 | 0.094 | 0.131 | 0.169 | 0.147 | 0.128 | 0.140 | 0.077 | 0.128 | 0.126 | 0.106 |
| meta131 |  | 154.132 | 369.819 | 0.040 | 0.037 | 0.035 | 0.024 | 0.031 | 0.021 | 0.032 | 0.047 | 0.050 | 0.047 | 0.039 | 0.034 | 0.032 | 0.031 | 0.033 |
| meta132 |  | 155.153 | 144.329 | 0.032 | 0.040 | 0.034 | 0.055 | 0.097 | 0.038 | 0.025 | 0.016 | 0.031 | 0.037 | 0.015 | 0.011 | 0.020 | 0.020 | 0.021 |
| meta133 |  | 156.040 | 287.076 | 0.086 | 0.090 | 0.076 | 0.121 | 0.099 | 0.095 | 0.071 | 0.077 | 0.087 | 0.079 | 0.097 | 0.080 | 0.056 | 0.070 | 0.075 |
| meta134 | L-Histidine | 156.075 | 299.454 | 0.183 | 0.192 | 0.152 | 0.138 | 0.168 | 0.101 | 0.219 | 0.251 | 0.199 | 0.213 | 0.241 | 0.223 | 0.152 | 0.103 | 0.174 |
| meta135 | Guanidine | 157.059 | 346.417 | 0.139 | 0.124 | 0.169 | 0.132 | 0.144 | 0.101 | 0.163 | 0.158 | 0.146 | 0.138 | 0.139 | 0.134 | 0.158 | 0.181 | 0.130 |
| meta136 | DL-Homocysteine | 158.025 | 216.578 | 0.003 | 0.004 | 0.005 | 0.006 | 0.007 | 0.003 | 0.002 | 0.008 | 0.006 | 0.008 | 0.003 | 0.003 | 0.001 | 0.004 | 0.004 |
| meta137 | N-Acetyl-L-aspartic acid | 158.043 | 302.267 | 0.065 | 0.079 | 0.060 | 0.056 | 0.060 | 0.043 | 0.077 | 0.113 | 0.061 | 0.066 | 0.089 | 0.083 | 0.059 | 0.039 | 0.071 |
| meta138 | Indoleacetic acid | 158.058 | 45.549 | 0.055 | 0.075 | 0.062 | 0.076 | 0.057 | 0.050 | 0.070 | 0.111 | 0.103 | 0.071 | 0.073 | 0.103 | 0.088 | 0.048 | 0.064 |
| meta139 | Methimazole | 158.995 | 261.733 | 0.090 | 0.095 | 0.085 | 0.093 | 0.087 | 0.083 | 0.088 | 0.060 | 0.095 | 0.062 | 0.091 | 0.089 | 0.096 | 0.098 | 0.102 |
| meta140 | Pectin (Galacturonic acid) | 159.026 | 157.370 | 1.350 | 1.476 | 1.254 | 1.508 | 1.810 | 1.288 | 1.611 | 1.082 | 1.061 | 1.135 | 1.288 | 1.451 | 1.382 | 1.365 | 1.396 |
| meta141 |  | 159.074 | 369.140 | 0.333 | 0.339 | 0.300 | 0.364 | 0.442 | 0.281 | 0.420 | 0.355 | 0.239 | 0.310 | 0.266 | 0.301 | 0.244 | 0.187 | 0.331 |
| meta142 | Phenelzine | 159.090 | 239.318 | 0.128 | 0.103 | 0.097 | 0.090 | 0.107 | 0.094 | 0.123 | 0.127 | 0.092 | 0.120 | 0.114 | 0.115 | 0.091 | 0.071 | 0.094 |
| meta143 | L-Valine | 159.111 | 248.017 | 0.013 | 0.019 | 0.009 | 0.005 | 0.005 | 0.004 | 0.007 | 0.013 | 0.024 | 0.021 | 0.028 | 0.026 | 0.014 | 0.004 | 0.015 |
| meta144 |  | 159.649 | 359.491 | 0.014 | 0.015 | 0.014 | 0.021 | 0.014 | 0.019 | 0.007 | 0.021 | 0.010 | 0.012 | 0.009 | 0.018 | 0.012 | 0.009 | 0.014 |
| meta145 | Acetyl-DL-Valine | 160.094 | 238.870 | 0.018 | 0.017 | 0.018 | 0.016 | 0.022 | 0.019 | 0.018 | 0.017 | 0.017 | 0.019 | 0.018 | 0.015 | 0.016 | 0.012 | 0.016 |
| meta146 | Isovalerylglycine | 160.095 | 181.845 | 0.008 | 0.008 | 0.007 | 0.007 | 0.010 | 0.006 | 0.012 | 0.008 | 0.005 | 0.004 | 0.005 | 0.008 | 0.004 | 0.005 | 0.007 |
| meta147 | Cyclohexylamine | 160.132 | 359.716 | 6.530 | 6.606 | 5.960 | 8.215 | 5.968 | 7.006 | 5.972 | 7.602 | 4.236 | 4.366 | 5.521 | 6.285 | 5.405 | 3.521 | 6.844 |
| meta148 | D-Alanyl-D-alanine (D-Ala-D-Ala) | 161.090 | 307.515 | 0.048 | 0.047 | 0.041 | 0.048 | 0.033 | 0.049 | 0.043 | 0.041 | 0.036 | 0.036 | 0.048 | 0.060 | 0.039 | 0.031 | 0.038 |
| meta149 | DL-Homoserine | 161.090 | 96.319 | 0.048 | 0.074 | 0.049 | 0.045 | 0.074 | 0.046 | 0.044 | 0.067 | 0.069 | 0.066 | 0.059 | 0.051 | 0.063 | 0.037 | 0.079 |
| meta150 |  | 161.621 | 330.604 | 0.349 | 0.372 | 0.350 | 0.323 | 0.418 | 0.261 | 0.383 | 0.397 | 0.278 | 0.273 | 0.344 | 0.343 | 0.292 | 0.292 | 0.331 |
| meta151 | Pyruvaldehyde | 162.074 | 368.074 | 0.325 | 0.423 | 0.415 | 0.191 | 0.432 | 0.213 | 0.210 | 0.259 | 0.174 | 0.296 | 0.414 | 0.374 | 0.413 | 0.739 | 0.468 |
| meta152 | DL-2-Aminoadipic acid | 162.074 | 410.448 | 0.144 | 0.168 | 0.167 | 0.151 | 0.248 | 0.166 | 0.183 | 0.240 | 0.161 | 0.179 | 0.183 | 0.191 | 0.164 | 0.165 | 0.146 |
| meta153 | L-Carnitine | 162.112 | 330.414 | 135.835 | 138.245 | 136.823 | 124.330 | 152.990 | 102.338 | 150.623 | 147.692 | 107.776 | 132.080 | 139.869 | 128.558 | 104.142 | 105.361 | 139.039 |
| meta154 |  | 163.041 | 96.619 | 0.061 | 0.102 | 0.077 | 0.096 | 0.078 | 0.084 | 0.084 | 0.091 | 0.122 | 0.097 | 0.091 | 0.130 | 0.119 | 0.115 | 0.239 |
| meta155 | L-Sorbose | 163.058 | 370.199 | 0.105 | 0.093 | 0.099 | 0.072 | 0.117 | 0.135 | 0.099 | 0.095 | 0.063 | 0.091 | 0.112 | 0.135 | 0.073 | 0.080 | 0.050 |
| meta156 | 2-Butoxyethanol | 163.070 | 422.549 | 0.018 | 0.025 | 0.025 | 0.023 | 0.027 | 0.017 | 0.022 | 0.018 | 0.020 | 0.022 | 0.019 | 0.020 | 0.021 | 0.018 | 0.025 |
| meta157 | L-Fucose | 164.072 | 38.807 | 0.039 | 0.039 | 0.038 | 0.037 | 0.044 | 0.031 | 0.046 | 0.039 | 0.065 | 0.030 | 0.033 | 0.036 | 0.039 | 0.035 | 0.052 |
| meta158 | Ala-Gly | 164.107 | 268.452 | 0.009 | 0.011 | 0.008 | 0.010 | 0.013 | 0.008 | 0.012 | 0.008 | 0.006 | 0.009 | 0.010 | 0.008 | 0.009 | 0.011 | 0.010 |
| meta159 |  | 165.053 | 279.897 | 0.087 | 0.063 | 0.074 | 0.060 | 0.068 | 0.077 | 0.084 | 0.089 | 0.049 | 0.080 | 0.086 | 0.073 | 0.070 | 0.054 | 0.065 |
| meta160 | DL-Methionine sulfoxide | 166.051 | 348.442 | 0.025 | 0.021 | 0.022 | 0.027 | 0.029 | 0.026 | 0.025 | 0.027 | 0.016 | 0.004 | 0.022 | 0.024 | 0.018 | 0.019 | 0.018 |
| meta161 | L-Phenylalanine | 166.085 | 238.918 | 0.905 | 0.738 | 0.692 | 0.594 | 0.800 | 0.694 | 1.068 | 1.022 | 0.577 | 0.819 | 1.005 | 0.930 | 0.616 | 0.511 | 0.793 |
| meta162 |  | 167.091 | 123.767 | 0.101 | 0.091 | 0.092 | 0.123 | 0.149 | 0.124 | 0.145 | 0.086 | 0.125 | 0.173 | 0.121 | 0.089 | 0.164 | 0.145 | 0.225 |
| meta163 | 3-Mercapto-2-butanone | 168.042 | 273.067 | 0.091 | 0.094 | 0.083 | 0.085 | 0.094 | 0.069 | 0.101 | 0.095 | 0.076 | 0.085 | 0.099 | 0.072 | 0.137 | 0.076 | 0.102 |
| meta164 | Pyridoxal (Vitamin B6) | 168.064 | 96.142 | 0.054 | 0.083 | 0.061 | 0.051 | 0.057 | 0.065 | 0.050 | 0.033 | 0.073 | 0.053 | 0.050 | 0.037 | 0.057 | 0.070 | 0.052 |
| meta165 | DL-a-Hydroxybutyric acid | 168.064 | 239.374 | 0.021 | 0.019 | 0.017 | 0.018 | 0.021 | 0.026 | 0.026 | 0.018 | 0.013 | 0.014 | 0.016 | 0.018 | 0.015 | 0.022 | 0.015 |
| meta166 | 4-Imidazoleacetic acid | 168.075 | 317.194 | 0.096 | 0.079 | 0.077 | 0.042 | 0.163 | 0.023 | 0.259 | 0.049 | 0.137 | 0.076 | 0.043 | 0.054 | 0.116 | 0.058 | 0.060 |
| meta167 |  | 168.111 | 322.239 | 0.026 | 0.028 | 0.027 | 0.037 | 0.026 | 0.020 | 0.024 | 0.039 | 0.050 | 0.042 | 0.046 | 0.030 | 0.028 | 0.031 | 0.036 |
| meta168 |  | 168.944 | 318.454 | 0.110 | 0.108 | 0.129 | 0.102 | 0.111 | 0.137 | 0.117 | 0.104 | 0.134 | 0.110 | 0.109 | 0.103 | 0.137 | 0.152 | 0.103 |
| meta169 |  | 169.057 | 353.628 | 0.616 | 0.658 | 0.572 | 0.629 | 0.637 | 0.489 | 0.601 | 0.628 | 0.638 | 0.575 | 0.594 | 0.538 | 0.635 | 0.513 | 0.597 |
| meta170 |  | 169.065 | 275.787 | 0.099 | 0.104 | 0.099 | 0.082 | 0.102 | 0.068 | 0.110 | 0.132 | 0.192 | 0.158 | 0.136 | 0.122 | 0.126 | 0.111 | 0.121 |
| meta171 | Norharmane | 169.074 | 43.231 | 0.068 | 0.082 | 0.074 | 0.029 | 0.142 | 0.017 | 0.478 | 0.030 | 0.069 | 0.023 | 0.025 | 0.028 | 0.095 | 0.033 | 0.023 |
| meta172 | N-Acetyl-L-alanine | 170.025 | 434.842 | 0.021 | 0.027 | 0.029 | 0.045 | 0.041 | 0.020 | 0.027 | 0.024 | 0.035 | 0.028 | 0.033 | 0.022 | 0.029 | 0.017 | 0.026 |
| meta173 | Hydroxyproline | 170.025 | 221.151 | 0.039 | 0.036 | 0.041 | 0.032 | 0.041 | 0.031 | 0.050 | 0.039 | 0.046 | 0.037 | 0.031 | 0.032 | 0.041 | 0.036 | 0.048 |
| meta174 | Glyceric acid | 170.038 | 272.720 | 0.004 | 0.005 | 0.006 | 0.005 | 0.003 | 0.004 | 0.005 | 0.008 | 0.009 | 0.004 | 0.008 | 0.003 | 0.007 | 0.004 | 0.005 |
| meta175 | Indoleacrylic acid | 170.058 | 239.489 | 0.004 | 0.002 | 0.003 | 0.003 | 0.003 | 0.003 | 0.004 | 0.003 | 0.002 | 0.003 | 0.004 | 0.003 | 0.009 | 0.005 | 0.003 |
| meta176 | 3-Methylhistamine | 170.068 | 275.650 | 0.006 | 0.006 | 0.009 | 0.005 | 0.005 | 0.003 | 0.005 | 0.009 | 0.014 | 0.004 | 0.008 | 0.006 | 0.007 | 0.007 | 0.010 |
| meta177 | 3-Methyl-L-histidine | 170.090 | 368.479 | 0.224 | 0.268 | 0.219 | 0.227 | 0.298 | 0.194 | 0.235 | 0.535 | 0.263 | 0.301 | 0.218 | 0.194 | 0.297 | 0.223 | 0.227 |
| meta178 | 3-Methylhistidine | 170.094 | 5.266 | 0.057 | 0.075 | 0.061 | 0.031 | 0.065 | 0.048 | 0.066 | 0.062 | 0.063 | 0.065 | 0.028 | 0.065 | 0.034 | 0.062 | 0.065 |
| meta179 |  | 170.116 | 206.140 | 0.030 | 0.030 | 0.030 | 0.023 | 0.028 | 0.024 | 0.019 | 0.034 | 0.029 | 0.027 | 0.039 | 0.059 | 0.026 | 0.029 | 0.026 |
| meta180 |  | 170.163 | 231.780 | 0.022 | 0.014 | 0.022 | 0.003 | 0.008 | 0.022 | 0.115 | 0.008 | 0.075 | 0.013 | 0.011 | 0.011 | 0.069 | 0.036 | 0.006 |
| meta181 |  | 171.147 | 37.503 | 0.137 | 0.177 | 0.147 | 0.122 | 0.157 | 0.104 | 0.156 | 0.187 | 0.158 | 0.156 | 0.150 | 0.140 | 0.126 | 0.137 | 0.185 |
| meta182 | (+-)-Mevalonolactone | 172.095 | 225.798 | 0.030 | 0.036 | 0.031 | 0.030 | 0.036 | 0.030 | 0.033 | 0.032 | 0.029 | 0.034 | 0.030 | 0.023 | 0.026 | 0.024 | 0.032 |
| meta183 | (+)-Methamphetamine | 172.113 | 17.439 | 0.008 | 0.010 | 0.010 | 0.009 | 0.010 | 0.015 | 0.009 | 0.009 | 0.002 | 0.020 | 0.010 | 0.010 | 0.022 | 0.009 | 0.015 |
| meta184 | Glycerol 3-phosphate | 173.019 | 421.836 | 0.269 | 0.256 | 0.228 | 0.208 | 0.254 | 0.189 | 0.264 | 0.263 | 0.252 | 0.241 | 0.260 | 0.249 | 0.359 | 0.235 | 0.246 |
| meta185 | Crotonic acid | 173.079 | 236.839 | 0.084 | 0.092 | 0.083 | 0.093 | 0.119 | 0.063 | 0.084 | 0.098 | 0.072 | 0.071 | 0.070 | 0.058 | 0.078 | 0.048 | 0.101 |
| meta186 | Pro-Gly | 173.090 | 335.426 | 0.047 | 0.055 | 0.041 | 0.046 | 0.044 | 0.045 | 0.041 | 0.046 | 0.039 | 0.052 | 0.044 | 0.026 | 0.049 | 0.043 | 0.043 |
| meta187 | L-Norleucine | 173.127 | 224.463 | 0.190 | 0.226 | 0.202 | 0.123 | 0.111 | 0.127 | 0.184 | 0.193 | 0.193 | 0.240 | 0.305 | 0.301 | 0.184 | 0.116 | 0.208 |
| meta188 | Arecoline | 173.127 | 340.595 | 0.043 | 0.043 | 0.042 | 0.039 | 0.153 | 0.018 | 0.094 | 0.067 | 0.035 | 0.069 | 0.016 | 0.014 | 0.057 | 0.028 | 0.032 |
| meta189 | Ethyl hydrogen malonate | 174.077 | 44.721 | 0.066 | 0.082 | 0.069 | 0.080 | 0.079 | 0.069 | 0.087 | 0.086 | 0.083 | 0.079 | 0.100 | 0.069 | 0.063 | 0.072 | 0.086 |
| meta190 | 3-Indoleacetonitrile | 174.101 | 181.202 | 0.011 | 0.012 | 0.008 | 0.022 | 0.019 | 0.010 | 0.014 | 0.012 | 0.017 | 0.011 | 0.013 | 0.018 | 0.008 | 0.009 | 0.018 |
| meta191 | Acetyl-DL-Leucine | 174.111 | 273.087 | 0.010 | 0.014 | 0.013 | 0.012 | 0.014 | 0.011 | 0.015 | 0.011 | 0.013 | 0.015 | 0.011 | 0.007 | 0.011 | 0.010 | 0.009 |
| meta192 | Butoxyacetic acid | 174.111 | 303.688 | 0.011 | 0.010 | 0.010 | 0.009 | 0.012 | 0.008 | 0.012 | 0.012 | 0.009 | 0.012 | 0.012 | 0.012 | 0.011 | 0.007 | 0.011 |
| meta193 |  | 174.122 | 291.492 | 0.071 | 0.076 | 0.071 | 0.081 | 0.093 | 0.077 | 0.087 | 0.086 | 0.059 | 0.086 | 0.065 | 0.057 | 0.057 | 0.036 | 0.087 |
| meta194 |  | 175.000 | 157.390 | 0.182 | 0.196 | 0.180 | 0.207 | 0.251 | 0.160 | 0.211 | 0.155 | 0.156 | 0.155 | 0.186 | 0.185 | 0.195 | 0.180 | 0.187 |
| meta195 |  | 175.015 | 365.314 | 0.084 | 0.108 | 0.096 | 0.077 | 0.074 | 0.081 | 0.072 | 0.089 | 0.112 | 0.121 | 0.086 | 0.072 | 0.083 | 0.075 | 0.086 |
| meta196 |  | 175.052 | 278.805 | 0.027 | 0.028 | 0.031 | 0.009 | 0.025 | 0.012 | 0.058 | 0.036 | 0.053 | 0.041 | 0.014 | 0.036 | 0.042 | 0.022 | 0.022 |
| meta197 | Iminodiacetic acid | 175.069 | 384.378 | 0.088 | 0.083 | 0.077 | 0.088 | 0.128 | 0.056 | 0.091 | 0.143 | 0.094 | 0.135 | 0.151 | 0.101 | 0.122 | 0.066 | 0.058 |
| meta198 | N-Carboxyethyl-.gamma.-aminobutyric acid | 175.081 | 355.910 | 0.037 | 0.040 | 0.040 | 0.041 | 0.043 | 0.038 | 0.028 | 0.027 | 0.052 | 0.039 | 0.026 | 0.031 | 0.050 | 0.060 | 0.016 |
| meta199 |  | 175.085 | 44.669 | 0.107 | 0.155 | 0.128 | 0.124 | 0.134 | 0.116 | 0.144 | 0.137 | 0.146 | 0.128 | 0.162 | 0.128 | 0.115 | 0.107 | 0.144 |
| meta200 | N2-Acetyl-L-ornithine | 175.106 | 337.312 | 0.106 | 0.109 | 0.089 | 0.111 | 0.127 | 0.082 | 0.120 | 0.162 | 0.109 | 0.127 | 0.117 | 0.113 | 0.108 | 0.089 | 0.116 |
| meta201 |  | 175.146 | 36.168 | 0.085 | 0.113 | 0.103 | 0.178 | 0.144 | 0.100 | 0.124 | 0.076 | 0.126 | 0.095 | 0.094 | 0.097 | 0.098 | 0.138 | 0.182 |
| meta202 | Nicotinyl | 176.009 | 264.200 | 0.002 | 0.002 | 0.002 | 0.003 | 0.002 | 0.002 | 0.002 | 0.002 | 0.003 | 0.002 | 0.002 | 0.001 | 0.001 | 0.001 | 0.001 |
| meta203 | Guanidinosuccinic acid | 176.065 | 388.310 | 0.228 | 0.242 | 0.214 | 0.170 | 0.223 | 0.150 | 0.154 | 0.343 | 0.361 | 0.399 | 0.305 | 0.236 | 0.298 | 0.190 | 0.261 |
| meta204 | L-Citrulline | 176.101 | 369.101 | 0.192 | 0.185 | 0.169 | 0.203 | 0.250 | 0.148 | 0.251 | 0.224 | 0.153 | 0.180 | 0.140 | 0.161 | 0.131 | 0.103 | 0.177 |
| meta205 |  | 176.126 | 355.615 | 0.027 | 0.028 | 0.024 | 0.030 | 0.028 | 0.021 | 0.026 | 0.031 | 0.016 | 0.023 | 0.022 | 0.017 | 0.024 | 0.019 | 0.024 |
| meta206 |  | 176.126 | 189.313 | 0.029 | 0.030 | 0.027 | 0.024 | 0.038 | 0.021 | 0.017 | 0.048 | 0.027 | 0.016 | 0.041 | 0.047 | 0.030 | 0.025 | 0.048 |
| meta207 |  | 176.145 | 222.731 | 0.041 | 0.044 | 0.042 | 0.026 | 0.019 | 0.032 | 0.030 | 0.035 | 0.060 | 0.055 | 0.054 | 0.059 | 0.056 | 0.041 | 0.038 |
| meta208 |  | 176.163 | 269.977 | 0.027 | 0.027 | 0.025 | 0.023 | 0.030 | 0.022 | 0.026 | 0.027 | 0.031 | 0.038 | 0.041 | 0.029 | 0.043 | 0.031 | 0.029 |
| meta209 |  | 176.874 | 287.951 | 2.763 | 2.761 | 2.824 | 3.067 | 3.301 | 2.747 | 3.093 | 3.080 | 3.659 | 3.268 | 3.229 | 3.088 | 3.670 | 3.176 | 3.081 |
| meta210 |  | 177.031 | 406.075 | 0.267 | 0.268 | 0.259 | 0.185 | 0.218 | 0.189 | 0.293 | 0.290 | 0.362 | 0.352 | 0.262 | 0.204 | 0.282 | 0.170 | 0.292 |
| meta211 | D-Glucuronolactone | 177.037 | 45.145 | 0.005 | 0.005 | 0.006 | 0.003 | 0.004 | 0.003 | 0.023 | 0.004 | 0.003 | 0.004 | 0.002 | 0.002 | 0.004 | 0.003 | 0.003 |
| meta212 | Ser-Ala | 177.086 | 299.189 | 0.008 | 0.010 | 0.009 | 0.009 | 0.008 | 0.007 | 0.009 | 0.011 | 0.014 | 0.014 | 0.011 | 0.008 | 0.006 | 0.008 | 0.007 |
| meta213 | Indole | 178.084 | 257.872 | 0.030 | 0.033 | 0.024 | 0.031 | 0.060 | 0.032 | 0.028 | 0.048 | 0.032 | 0.029 | 0.010 | 0.014 | 0.013 | 0.019 | 0.017 |
| meta214 | Cys-Gly | 179.047 | 380.060 | 0.101 | 0.078 | 0.081 | 0.029 | 0.124 | 0.036 | 0.323 | 0.037 | 0.160 | 0.066 | 0.034 | 0.033 | 0.137 | 0.055 | 0.036 |
| meta215 | Nicotinuric acid | 180.050 | 380.052 | 0.005 | 0.004 | 0.006 | 0.021 | 0.007 | 0.018 | 0.019 | 0.023 | 0.009 | 0.002 | 0.021 | 0.019 | 0.008 | 0.002 | 0.024 |
| meta216 | myo-Inositol | 180.064 | 145.148 | 0.028 | 0.027 | 0.019 | 0.027 | 0.018 | 0.028 | 0.026 | 0.033 | 0.026 | 0.022 | 0.028 | 0.038 | 0.026 | 0.027 | 0.063 |
| meta217 |  | 180.100 | 37.182 | 0.065 | 0.072 | 0.068 | 0.063 | 0.073 | 0.066 | 0.083 | 0.082 | 0.065 | 0.066 | 0.076 | 0.064 | 0.073 | 0.063 | 0.111 |
| meta218 | 3-Hydroxydodecanoic acid | 181.157 | 41.391 | 0.066 | 0.078 | 0.078 | 0.096 | 0.103 | 0.047 | 0.087 | 0.093 | 0.091 | 0.080 | 0.093 | 0.112 | 0.072 | 0.074 | 0.169 |
| meta219 |  | 182.046 | 187.356 | 0.022 | 0.021 | 0.022 | 0.016 | 0.029 | 0.017 | 0.013 | 0.026 | 0.020 | 0.035 | 0.022 | 0.011 | 0.021 | 0.021 | 0.017 |
| meta220 |  | 182.073 | 248.199 | 0.196 | 0.296 | 0.207 | 0.171 | 0.228 | 0.146 | 0.225 | 0.287 | 0.388 | 0.392 | 0.297 | 0.261 | 0.316 | 0.233 | 0.293 |
| meta221 | L-Tyrosine | 182.080 | 280.146 | 0.127 | 0.102 | 0.112 | 0.088 | 0.094 | 0.115 | 0.129 | 0.131 | 0.081 | 0.132 | 0.129 | 0.117 | 0.109 | 0.078 | 0.107 |
| meta222 | Gaboxadol | 182.091 | 51.560 | 0.045 | 0.050 | 0.036 | 0.007 | 0.072 | 0.008 | 0.076 | 0.006 | 0.048 | 0.038 | 0.009 | 0.014 | 0.053 | 0.017 | 0.017 |
| meta223 |  | 183.061 | 259.553 | 0.047 | 0.058 | 0.045 | 0.050 | 0.084 | 0.044 | 0.062 | 0.044 | 0.042 | 0.040 | 0.049 | 0.059 | 0.060 | 0.064 | 0.058 |
| meta224 | 4-Hydroxybenzaldehyde | 183.061 | 198.860 | 0.006 | 0.005 | 0.007 | 0.006 | 0.005 | 0.005 | 0.006 | 0.004 | 0.004 | 0.004 | 0.007 | 0.005 | 0.006 | 0.005 | 0.005 |
| meta225 |  | 184.059 | 390.923 | 0.072 | 0.073 | 0.074 | 0.075 | 0.123 | 0.062 | 0.020 | 0.065 | 0.039 | 0.049 | 0.057 | 0.090 | 0.107 | 0.092 | 0.090 |
| meta226 | 4-Pyridoxic acid | 184.062 | 141.133 | 0.017 | 0.014 | 0.013 | 0.026 | 0.012 | 0.024 | 0.012 | 0.038 | 0.029 | 0.027 | 0.006 | 0.007 | 0.008 | 0.014 | 0.011 |
| meta227 |  | 184.070 | 300.455 | 0.173 | 0.167 | 0.139 | 0.092 | 0.133 | 0.069 | 0.244 | 0.358 | 0.243 | 0.171 | 0.255 | 0.283 | 0.152 | 0.074 | 0.137 |
| meta228 | Phosphorylcholine | 184.073 | 467.253 | 35.678 | 46.328 | 41.027 | 40.477 | 45.301 | 29.341 | 33.580 | 68.181 | 38.080 | 35.566 | 38.413 | 36.934 | 32.743 | 33.799 | 37.397 |
| meta229 |  | 184.089 | 310.660 | 2.956 | 2.834 | 2.603 | 2.116 | 2.591 | 1.974 | 2.732 | 3.418 | 3.921 | 3.937 | 3.112 | 2.857 | 2.902 | 2.582 | 2.750 |
| meta230 |  | 184.089 | 275.695 | 0.759 | 0.844 | 0.708 | 0.584 | 0.804 | 0.500 | 0.781 | 1.007 | 1.429 | 1.140 | 0.920 | 0.903 | 0.935 | 0.836 | 0.901 |
| meta231 |  | 185.095 | 176.464 | 0.129 | 0.146 | 0.106 | 0.131 | 0.117 | 0.128 | 0.132 | 0.138 | 0.122 | 0.143 | 0.129 | 0.114 | 0.147 | 0.116 | 0.111 |
| meta232 | Pelletierine | 186.084 | 310.720 | 0.132 | 0.126 | 0.122 | 0.090 | 0.105 | 0.082 | 0.109 | 0.146 | 0.181 | 0.185 | 0.144 | 0.118 | 0.141 | 0.105 | 0.142 |
| meta233 |  | 186.111 | 204.934 | 0.027 | 0.030 | 0.025 | 0.014 | 0.061 | 0.013 | 0.098 | 0.020 | 0.019 | 0.017 | 0.016 | 0.015 | 0.020 | 0.013 | 0.019 |
| meta234 | Jasmine lactone | 186.147 | 38.879 | 0.017 | 0.027 | 0.027 | 0.023 | 0.017 | 0.021 | 0.023 | 0.019 | 0.030 | 0.019 | 0.017 | 0.016 | 0.024 | 0.024 | 0.026 |
| meta235 |  | 186.158 | 322.836 | 0.172 | 0.132 | 0.143 | 0.060 | 0.095 | 0.120 | 0.533 | 0.088 | 0.369 | 0.107 | 0.079 | 0.095 | 0.417 | 0.215 | 0.081 |
| meta236 | Hydroxyacetone | 187.035 | 279.502 | 0.010 | 0.011 | 0.009 | 0.007 | 0.014 | 0.006 | 0.009 | 0.014 | 0.006 | 0.010 | 0.018 | 0.013 | 0.005 | 0.008 | 0.013 |
| meta237 | Allocystathionine | 187.056 | 267.553 | 0.036 | 0.041 | 0.046 | 0.017 | 0.016 | 0.013 | 0.018 | 0.021 | 0.013 | 0.017 | 0.020 | 0.016 | 0.023 | 0.020 | 0.020 |
| meta238 | Pro-Ala | 187.106 | 322.257 | 0.072 | 0.046 | 0.050 | 0.044 | 0.047 | 0.070 | 0.032 | 0.060 | 0.040 | 0.046 | 0.053 | 0.049 | 0.058 | 0.038 | 0.052 |
| meta239 |  | 187.142 | 317.492 | 0.016 | 0.020 | 0.017 | 0.017 | 0.017 | 0.014 | 0.018 | 0.017 | 0.017 | 0.018 | 0.016 | 0.018 | 0.019 | 0.016 | 0.018 |
| meta240 | DL-Indole-3-lactic acid | 188.069 | 238.964 | 0.260 | 0.165 | 0.177 | 0.153 | 0.174 | 0.168 | 0.253 | 0.231 | 0.127 | 0.233 | 0.235 | 0.192 | 0.157 | 0.105 | 0.170 |
| meta241 | alpha-Guanidinoglutaric Acid | 189.072 | 238.919 | 0.030 | 0.019 | 0.025 | 0.021 | 0.024 | 0.021 | 0.031 | 0.028 | 0.018 | 0.029 | 0.028 | 0.024 | 0.018 | 0.014 | 0.022 |
| meta242 | N6-Acetyl-L-lysine | 189.122 | 364.350 | 0.215 | 0.244 | 0.190 | 0.203 | 0.210 | 0.175 | 0.206 | 0.287 | 0.268 | 0.252 | 0.178 | 0.212 | 0.215 | 0.225 | 0.166 |
| meta243 | Val-Ala | 189.122 | 45.145 | 0.030 | 0.024 | 0.032 | 0.029 | 0.025 | 0.033 | 0.023 | 0.021 | 0.024 | 0.024 | 0.027 | 0.027 | 0.031 | 0.024 | 0.018 |
| meta244 | L-Pyroglutamic acid | 190.069 | 375.850 | 0.196 | 0.196 | 0.164 | 0.169 | 0.239 | 0.119 | 0.205 | 0.211 | 0.151 | 0.214 | 0.224 | 0.177 | 0.161 | 0.125 | 0.192 |
| meta245 | 3-Methoxytyramine | 190.089 | 295.161 | 0.030 | 0.030 | 0.024 | 0.028 | 0.032 | 0.022 | 0.030 | 0.029 | 0.021 | 0.029 | 0.037 | 0.025 | 0.024 | 0.018 | 0.031 |
| meta246 |  | 191.047 | 380.052 | 0.166 | 0.126 | 0.126 | 0.041 | 0.206 | 0.042 | 0.589 | 0.045 | 0.269 | 0.117 | 0.043 | 0.048 | 0.223 | 0.077 | 0.055 |
| meta247 |  | 191.047 | 216.530 | 0.046 | 0.051 | 0.050 | 0.017 | 0.041 | 0.015 | 0.071 | 0.026 | 0.061 | 0.046 | 0.016 | 0.026 | 0.058 | 0.025 | 0.039 |
| meta248 | Quinaldic acid | 191.080 | 44.001 | 0.086 | 0.121 | 0.101 | 0.104 | 0.102 | 0.090 | 0.120 | 0.106 | 0.123 | 0.111 | 0.139 | 0.100 | 0.090 | 0.097 | 0.119 |
| meta249 | L-Methionine | 191.089 | 295.036 | 0.005 | 0.007 | 0.004 | 0.004 | 0.004 | 0.004 | 0.005 | 0.006 | 0.004 | 0.004 | 0.007 | 0.004 | 0.005 | 0.003 | 0.005 |
| meta250 | Ala-Thr | 191.101 | 309.969 | 0.015 | 0.018 | 0.017 | 0.022 | 0.015 | 0.025 | 0.023 | 0.010 | 0.014 | 0.011 | 0.013 | 0.020 | 0.020 | 0.013 | 0.021 |
| meta251 |  | 191.981 | 288.606 | 0.015 | 0.013 | 0.014 | 0.014 | 0.016 | 0.015 | 0.019 | 0.017 | 0.015 | 0.017 | 0.021 | 0.015 | 0.018 | 0.011 | 0.018 |
| meta252 | L-Glutamate | 192.022 | 382.072 | 0.033 | 0.042 | 0.037 | 0.037 | 0.042 | 0.027 | 0.040 | 0.046 | 0.040 | 0.040 | 0.041 | 0.037 | 0.029 | 0.034 | 0.038 |
| meta253 | Dopamine | 192.038 | 438.939 | 0.011 | 0.012 | 0.011 | 0.013 | 0.016 | 0.009 | 0.010 | 0.021 | 0.012 | 0.011 | 0.015 | 0.014 | 0.005 | 0.006 | 0.010 |
| meta254 |  | 193.026 | 406.201 | 0.025 | 0.034 | 0.028 | 0.026 | 0.023 | 0.022 | 0.031 | 0.028 | 0.034 | 0.029 | 0.026 | 0.020 | 0.029 | 0.022 | 0.029 |
| meta255 |  | 193.026 | 434.182 | 0.070 | 0.081 | 0.080 | 0.059 | 0.063 | 0.055 | 0.080 | 0.076 | 0.098 | 0.089 | 0.069 | 0.066 | 0.083 | 0.061 | 0.083 |
| meta256 |  | 193.157 | 36.155 | 0.117 | 0.130 | 0.118 | 0.230 | 0.169 | 0.132 | 0.163 | 0.098 | 0.151 | 0.128 | 0.105 | 0.106 | 0.124 | 0.158 | 0.233 |
| meta257 | Nicorandil | 194.056 | 455.133 | 0.041 | 0.037 | 0.042 | 0.030 | 0.045 | 0.035 | 0.042 | 0.037 | 0.044 | 0.042 | 0.042 | 0.042 | 0.048 | 0.047 | 0.031 |
| meta258 |  | 194.117 | 304.383 | 1.462 | 1.712 | 1.538 | 1.555 | 1.555 | 1.252 | 1.616 | 1.779 | 1.718 | 1.537 | 1.528 | 1.391 | 1.499 | 1.474 | 1.624 |
| meta259 |  | 195.001 | 372.261 | 0.057 | 0.052 | 0.048 | 0.054 | 0.055 | 0.038 | 0.066 | 0.060 | 0.037 | 0.058 | 0.073 | 0.042 | 0.036 | 0.032 | 0.059 |
| meta260 | 3-Hydroxyanthranilic acid | 195.075 | 342.890 | 0.009 | 0.008 | 0.005 | 0.011 | 0.010 | 0.007 | 0.017 | 0.048 | 0.050 | 0.049 | 0.006 | 0.007 | 0.010 | 0.006 | 0.008 |
| meta261 | Ethyl 3-hydroxybutyrate | 196.095 | 306.622 | 0.025 | 0.025 | 0.029 | 0.021 | 0.026 | 0.017 | 0.024 | 0.030 | 0.024 | 0.028 | 0.026 | 0.027 | 0.024 | 0.026 | 0.028 |
| meta262 | 2-Phenylacetamide | 196.095 | 44.001 | 0.044 | 0.055 | 0.052 | 0.054 | 0.062 | 0.050 | 0.056 | 0.057 | 0.044 | 0.051 | 0.052 | 0.043 | 0.054 | 0.046 | 0.080 |
| meta263 |  | 198.036 | 384.480 | 0.042 | 0.037 | 0.038 | 0.031 | 0.043 | 0.034 | 0.026 | 0.034 | 0.028 | 0.037 | 0.051 | 0.041 | 0.070 | 0.052 | 0.039 |
| meta264 | Phosphoglycolic acid | 198.068 | 312.365 | 0.302 | 0.365 | 0.340 | 0.273 | 0.356 | 0.244 | 0.368 | 0.397 | 0.606 | 0.453 | 0.396 | 0.373 | 0.365 | 0.389 | 0.437 |
| meta265 |  | 198.083 | 368.951 | 0.208 | 0.221 | 0.188 | 0.223 | 0.261 | 0.163 | 0.245 | 0.209 | 0.139 | 0.176 | 0.167 | 0.196 | 0.140 | 0.122 | 0.177 |
| meta266 | N-Acetyl-L-Histidine | 198.086 | 298.755 | 1.357 | 1.456 | 1.292 | 1.029 | 1.402 | 0.895 | 1.303 | 1.219 | 1.135 | 1.799 | 1.827 | 1.379 | 1.133 | 0.860 | 1.498 |
| meta267 | Citrulline | 198.087 | 467.891 | 0.048 | 0.033 | 0.033 | 0.032 | 0.032 | 0.038 | 0.054 | 0.031 | 0.029 | 0.030 | 0.024 | 0.040 | 0.056 | 0.036 | 0.033 |
| meta268 |  | 198.122 | 369.711 | 0.096 | 0.078 | 0.076 | 0.058 | 0.075 | 0.050 | 0.076 | 0.099 | 0.112 | 0.112 | 0.107 | 0.113 | 0.078 | 0.066 | 0.082 |
| meta269 |  | 198.133 | 399.821 | 0.091 | 0.097 | 0.105 | 0.100 | 0.101 | 0.066 | 0.080 | 0.110 | 0.156 | 0.124 | 0.117 | 0.098 | 0.118 | 0.119 | 0.097 |
| meta270 | .beta.-Citronellol | 198.184 | 41.781 | 0.123 | 0.136 | 0.137 | 0.184 | 0.169 | 0.087 | 0.147 | 0.162 | 0.159 | 0.135 | 0.161 | 0.196 | 0.125 | 0.129 | 0.290 |
| meta271 |  | 199.099 | 248.358 | 0.329 | 0.415 | 0.289 | 0.282 | 0.333 | 0.191 | 0.378 | 0.404 | 0.577 | 0.555 | 0.438 | 0.346 | 0.416 | 0.328 | 0.423 |
| meta272 | p-CHLOROPHENYLALANINE | 200.045 | 154.763 | 0.007 | 0.008 | 0.007 | 0.006 | 0.006 | 0.009 | 0.009 | 0.009 | 0.010 | 0.008 | 0.008 | 0.008 | 0.011 | 0.009 | 0.007 |
| meta273 |  | 200.083 | 206.031 | 0.106 | 0.107 | 0.107 | 0.068 | 0.119 | 0.074 | 0.102 | 0.129 | 0.175 | 0.155 | 0.118 | 0.114 | 0.120 | 0.111 | 0.120 |
| meta274 |  | 200.090 | 366.889 | 0.043 | 0.053 | 0.055 | 0.043 | 0.052 | 0.034 | 0.059 | 0.046 | 0.042 | 0.040 | 0.037 | 0.047 | 0.046 | 0.041 | 0.044 |
| meta275 |  | 200.174 | 233.610 | 0.014 | 0.013 | 0.012 | 0.002 | 0.008 | 0.002 | 0.023 | 0.020 | 0.016 | 0.010 | 0.007 | 0.014 | 0.015 | 0.007 | 0.006 |
| meta276 | D-Erythrose 4-phosphate | 201.014 | 428.192 | 0.006 | 0.006 | 0.006 | 0.004 | 0.016 | 0.003 | 0.024 | 0.003 | 0.007 | 0.005 | 0.003 | 0.003 | 0.013 | 0.005 | 0.005 |
| meta277 | Sarcosine | 201.086 | 303.624 | 0.075 | 0.082 | 0.079 | 0.074 | 0.071 | 0.050 | 0.094 | 0.099 | 0.100 | 0.086 | 0.095 | 0.087 | 0.040 | 0.052 | 0.063 |
| meta278 |  | 201.115 | 310.690 | 0.826 | 0.775 | 0.733 | 0.544 | 0.671 | 0.546 | 0.725 | 0.900 | 1.063 | 1.011 | 0.851 | 0.725 | 0.802 | 0.678 | 0.797 |
| meta279 |  | 201.197 | 384.145 | 0.020 | 0.017 | 0.017 | 0.019 | 0.020 | 0.021 | 0.018 | 0.020 | 0.019 | 0.020 | 0.018 | 0.018 | 0.016 | 0.019 | 0.018 |
| meta280 | 3-Hydroxybenzoate | 202.043 | 224.463 | 0.902 | 0.864 | 0.805 | 0.692 | 0.811 | 1.014 | 0.901 | 0.914 | 0.958 | 1.019 | 0.895 | 0.883 | 0.901 | 0.869 | 0.923 |
| meta281 |  | 202.069 | 390.562 | 0.165 | 0.168 | 0.171 | 0.167 | 0.272 | 0.144 | 0.114 | 0.123 | 0.138 | 0.117 | 0.137 | 0.211 | 0.239 | 0.206 | 0.175 |
| meta282 | 3,4-Dihydroxymandelic acid | 202.069 | 280.707 | 0.012 | 0.018 | 0.019 | 0.019 | 0.035 | 0.013 | 0.013 | 0.008 | 0.035 | 0.007 | 0.015 | 0.007 | 0.009 | 0.012 | 0.017 |
| meta283 | NG,NG-dimethyl-L-arginine(ADMA) | 202.142 | 181.918 | 0.015 | 0.012 | 0.016 | 0.015 | 0.049 | 0.010 | 0.054 | 0.005 | 0.005 | 0.003 | 0.010 | 0.010 | 0.008 | 0.003 | 0.006 |
| meta284 |  | 203.051 | 278.585 | 0.469 | 0.598 | 0.572 | 0.538 | 0.484 | 0.492 | 0.742 | 0.592 | 0.516 | 0.599 | 0.538 | 0.553 | 0.599 | 0.518 | 0.570 |
| meta285 |  | 203.047 | 204.492 | 0.035 | 0.037 | 0.034 | 0.025 | 0.012 | 0.021 | 0.020 | 0.035 | 0.045 | 0.040 | 0.036 | 0.037 | 0.037 | 0.026 | 0.036 |
| meta286 | Pro-Ser | 203.101 | 344.030 | 0.039 | 0.040 | 0.037 | 0.044 | 0.034 | 0.056 | 0.033 | 0.033 | 0.024 | 0.035 | 0.031 | 0.051 | 0.036 | 0.034 | 0.036 |
| meta287 | Ile-Ala | 203.137 | 219.165 | 0.008 | 0.009 | 0.012 | 0.013 | 0.008 | 0.012 | 0.018 | 0.003 | 0.003 | 0.005 | 0.004 | 0.006 | 0.010 | 0.005 | 0.009 |
| meta288 |  | 203.177 | 35.353 | 0.012 | 0.015 | 0.016 | 0.021 | 0.024 | 0.021 | 0.027 | 0.022 | 0.027 | 0.019 | 0.023 | 0.022 | 0.015 | 0.021 | 0.022 |
| meta289 |  | 204.001 | 344.251 | 0.037 | 0.038 | 0.032 | 0.033 | 0.041 | 0.026 | 0.047 | 0.032 | 0.028 | 0.033 | 0.045 | 0.036 | 0.024 | 0.022 | 0.066 |
| meta290 | N-Acetyl-D-glucosamine | 204.085 | 421.239 | 0.136 | 0.133 | 0.123 | 0.112 | 0.147 | 0.123 | 0.146 | 0.187 | 0.121 | 0.107 | 0.118 | 0.114 | 0.129 | 0.090 | 0.154 |
| meta291 | N-Acetylmannosamine | 204.085 | 239.458 | 0.088 | 0.086 | 0.075 | 0.064 | 0.098 | 0.099 | 0.104 | 0.065 | 0.052 | 0.055 | 0.063 | 0.077 | 0.059 | 0.084 | 0.057 |
| meta292 | mafenide | 204.085 | 152.154 | 0.035 | 0.046 | 0.043 | 0.045 | 0.036 | 0.050 | 0.039 | 0.032 | 0.024 | 0.043 | 0.033 | 0.037 | 0.038 | 0.032 | 0.038 |
| meta293 | Gly-Gln | 204.096 | 336.240 | 0.024 | 0.023 | 0.017 | 0.014 | 0.032 | 0.013 | 0.028 | 0.018 | 0.055 | 0.024 | 0.047 | 0.022 | 0.030 | 0.015 | 0.068 |
| meta294 | Acetylcarnitine | 204.122 | 283.049 | 16.136 | 17.968 | 16.102 | 13.444 | 23.672 | 8.704 | 23.105 | 20.694 | 20.173 | 14.236 | 10.571 | 8.874 | 25.227 | 9.671 | 21.302 |
| meta295 | Ala-Pro | 204.132 | 308.034 | 0.438 | 0.402 | 0.342 | 0.426 | 0.692 | 0.312 | 0.462 | 0.631 | 0.274 | 0.402 | 0.354 | 0.251 | 0.358 | 0.254 | 0.342 |
| meta296 |  | 205.051 | 393.236 | 0.102 | 0.095 | 0.093 | 0.071 | 0.131 | 0.072 | 0.100 | 0.083 | 0.074 | 0.075 | 0.109 | 0.100 | 0.155 | 0.102 | 0.114 |
| meta297 |  | 205.067 | 277.445 | 0.069 | 0.077 | 0.053 | 0.089 | 0.084 | 0.048 | 0.086 | 0.074 | 0.053 | 0.040 | 0.098 | 0.088 | 0.043 | 0.049 | 0.034 |
| meta298 |  | 205.085 | 33.198 | 0.760 | 0.877 | 0.854 | 1.369 | 1.811 | 1.325 | 2.002 | 1.896 | 1.973 | 1.757 | 1.707 | 1.932 | 1.251 | 1.779 | 2.109 |
| meta299 |  | 205.102 | 27.742 | 0.197 | 0.264 | 0.238 | 0.465 | 0.263 | 0.152 | 0.217 | 0.402 | 0.247 | 0.215 | 0.263 | 0.328 | 0.228 | 0.416 | 0.243 |
| meta300 |  | 206.053 | 467.898 | 0.697 | 0.840 | 0.737 | 0.813 | 0.828 | 0.479 | 0.783 | 1.281 | 0.861 | 0.945 | 0.836 | 0.716 | 0.545 | 0.676 | 0.744 |
| meta301 |  | 207.060 | 46.349 | 0.061 | 0.076 | 0.057 | 0.066 | 0.071 | 0.050 | 0.067 | 0.069 | 0.072 | 0.090 | 0.054 | 0.039 | 0.060 | 0.057 | 0.078 |
| meta302 | Kynurenic acid | 207.078 | 276.566 | 0.021 | 0.029 | 0.021 | 0.023 | 0.025 | 0.018 | 0.027 | 0.020 | 0.016 | 0.017 | 0.015 | 0.014 | 0.032 | 0.015 | 0.027 |
| meta303 | Glycerol | 207.086 | 335.410 | 0.195 | 0.237 | 0.197 | 0.246 | 0.244 | 0.154 | 0.146 | 0.261 | 0.221 | 0.136 | 0.208 | 0.178 | 0.230 | 0.116 | 0.114 |
| meta304 |  | 207.096 | 388.539 | 0.271 | 0.372 | 0.321 | 0.419 | 0.397 | 0.306 | 0.349 | 0.288 | 0.200 | 0.215 | 0.262 | 0.304 | 0.221 | 0.321 | 0.291 |
| meta305 | N-Acetyl-L-phenylalanine | 208.095 | 163.011 | 0.010 | 0.009 | 0.012 | 0.015 | 0.006 | 0.011 | 0.004 | 0.012 | 0.016 | 0.011 | 0.020 | 0.012 | 0.007 | 0.005 | 0.013 |
| meta306 |  | 208.168 | 120.556 | 0.057 | 0.064 | 0.043 | 0.090 | 0.075 | 0.056 | 0.070 | 0.063 | 0.066 | 0.063 | 0.050 | 0.053 | 0.070 | 0.074 | 0.121 |
| meta307 | (R)-3-Hydroxybutyric acid | 209.100 | 255.758 | 0.051 | 0.049 | 0.055 | 0.016 | 0.015 | 0.006 | 0.030 | 0.029 | 0.011 | 0.030 | 0.023 | 0.024 | 0.050 | 0.026 | 0.020 |
| meta308 |  | 210.041 | 140.706 | 0.029 | 0.030 | 0.033 | 0.013 | 0.097 | 0.026 | 0.038 | 0.029 | 0.027 | 0.037 | 0.010 | 0.009 | 0.038 | 0.040 | 0.028 |
| meta309 | Monomethyl glutaric acid | 210.077 | 187.310 | 0.055 | 0.082 | 0.076 | 0.063 | 0.067 | 0.048 | 0.070 | 0.064 | 0.078 | 0.058 | 0.057 | 0.056 | 0.060 | 0.072 | 0.063 |
| meta310 | Butyl lactate | 210.109 | 206.121 | 0.028 | 0.029 | 0.035 | 0.024 | 0.023 | 0.023 | 0.024 | 0.035 | 0.031 | 0.030 | 0.032 | 0.044 | 0.025 | 0.029 | 0.026 |
| meta311 | N-Methyl-D-aspartic acid | 211.069 | 312.134 | 0.092 | 0.113 | 0.104 | 0.081 | 0.093 | 0.071 | 0.108 | 0.098 | 0.116 | 0.108 | 0.092 | 0.082 | 0.091 | 0.086 | 0.109 |
| meta312 | O-Acetyl-L-serine | 211.068 | 295.571 | 0.081 | 0.110 | 0.069 | 0.088 | 0.084 | 0.056 | 0.072 | 0.081 | 0.070 | 0.086 | 0.096 | 0.071 | 0.066 | 0.052 | 0.082 |
| meta313 | Hippuric acid, methyl ester | 211.103 | 327.279 | 0.008 | 0.008 | 0.005 | 0.009 | 0.009 | 0.005 | 0.007 | 0.008 | 0.009 | 0.002 | 0.007 | 0.001 | 0.006 | 0.004 | 0.006 |
| meta314 | Phenylacetylglycine | 211.113 | 27.002 | 0.003 | 0.005 | 0.004 | 0.004 | 0.005 | 0.006 | 0.005 | 0.009 | 0.004 | 0.005 | 0.005 | 0.002 | 0.003 | 0.012 | 0.005 |
| meta315 | N-Acetyl-L-glutamate | 212.051 | 375.741 | 0.192 | 0.189 | 0.154 | 0.156 | 0.241 | 0.107 | 0.201 | 0.200 | 0.137 | 0.188 | 0.218 | 0.162 | 0.157 | 0.113 | 0.179 |
| meta316 |  | 212.057 | 122.016 | 0.034 | 0.027 | 0.028 | 0.073 | 0.099 | 0.056 | 0.026 | 0.015 | 0.022 | 0.031 | 0.028 | 0.007 | 0.038 | 0.046 | 0.014 |
| meta317 | Harmine | 212.099 | 358.111 | 0.007 | 0.007 | 0.006 | 0.007 | 0.007 | 0.006 | 0.004 | 0.009 | 0.004 | 0.008 | 0.006 | 0.006 | 0.006 | 0.007 | 0.004 |
| meta318 |  | 212.199 | 39.767 | 0.019 | 0.022 | 0.020 | 0.036 | 0.039 | 0.010 | 0.038 | 0.048 | 0.038 | 0.025 | 0.023 | 0.030 | 0.036 | 0.179 | 0.040 |
| meta319 | D-Ribulose 5-phosphate | 213.014 | 429.877 | 0.069 | 0.063 | 0.068 | 0.056 | 0.063 | 0.062 | 0.069 | 0.088 | 0.066 | 0.062 | 0.069 | 0.066 | 0.073 | 0.053 | 0.074 |
| meta320 | L-Arginine | 213.074 | 380.075 | 0.009 | 0.017 | 0.010 | 0.005 | 0.010 | 0.017 | 0.007 | 0.017 | 0.024 | 0.023 | 0.013 | 0.014 | 0.033 | 0.025 | 0.012 |
| meta321 |  | 213.169 | 226.424 | 0.008 | 0.005 | 0.005 | 0.005 | 0.011 | 0.002 | 0.023 | 0.028 | 0.009 | 0.004 | 0.004 | 0.014 | 0.011 | 0.004 | 0.002 |
| meta322 |  | 214.110 | 328.423 | 0.129 | 0.254 | 0.197 | 0.187 | 0.171 | 0.115 | 0.179 | 0.231 | 0.348 | 0.307 | 0.240 | 0.283 | 0.220 | 0.247 | 0.262 |
| meta323 | Thymol | 214.120 | 33.411 | 0.019 | 0.012 | 0.018 | 0.023 | 0.042 | 0.023 | 0.028 | 0.032 | 0.040 | 0.040 | 0.018 | 0.027 | 0.036 | 0.035 | 0.029 |
| meta324 |  | 214.178 | 37.514 | 0.075 | 0.089 | 0.087 | 0.221 | 0.088 | 0.068 | 0.159 | 0.037 | 0.098 | 0.060 | 0.071 | 0.061 | 0.079 | 0.103 | 0.140 |
| meta325 |  | 215.094 | 266.658 | 0.073 | 0.087 | 0.086 | 0.064 | 0.085 | 0.043 | 0.078 | 0.100 | 0.133 | 0.122 | 0.104 | 0.090 | 0.104 | 0.097 | 0.094 |
| meta326 |  | 215.101 | 88.813 | 0.040 | 0.032 | 0.028 | 0.046 | 0.057 | 0.040 | 0.062 | 0.048 | 0.039 | 0.019 | 0.015 | 0.035 | 0.023 | 0.048 | 0.047 |
| meta327 |  | 215.124 | 318.914 | 0.054 | 0.062 | 0.059 | 0.053 | 0.059 | 0.052 | 0.057 | 0.055 | 0.061 | 0.056 | 0.056 | 0.058 | 0.057 | 0.058 | 0.063 |
| meta328 | Pro-Val | 215.137 | 272.246 | 0.032 | 0.031 | 0.033 | 0.036 | 0.037 | 0.040 | 0.033 | 0.024 | 0.022 | 0.029 | 0.038 | 0.029 | 0.030 | 0.026 | 0.034 |
| meta329 | sn-Glycerol 3-phosphoethanolamine | 216.061 | 372.290 | 0.530 | 0.465 | 0.433 | 0.442 | 0.510 | 0.352 | 0.621 | 0.535 | 0.284 | 0.511 | 0.659 | 0.376 | 0.329 | 0.281 | 0.501 |
| meta330 |  | 216.195 | 41.586 | 0.332 | 0.390 | 0.392 | 0.474 | 0.450 | 0.225 | 0.417 | 0.485 | 0.464 | 0.373 | 0.445 | 0.555 | 0.357 | 0.377 | 0.867 |
| meta331 | N-.alpha.-Acetyl-L-arginine | 217.128 | 346.461 | 0.349 | 0.329 | 0.326 | 0.215 | 0.266 | 0.215 | 0.252 | 0.556 | 0.494 | 0.682 | 0.301 | 0.443 | 0.230 | 0.339 | 0.247 |
| meta332 |  | 217.124 | 27.218 | 0.079 | 0.111 | 0.102 | 0.111 | 0.111 | 0.071 | 0.095 | 0.129 | 0.114 | 0.090 | 0.101 | 0.105 | 0.108 | 0.109 | 0.107 |
| meta333 | Val-Val | 217.153 | 199.422 | 0.017 | 0.018 | 0.016 | 0.025 | 0.015 | 0.022 | 0.022 | 0.007 | 0.004 | 0.008 | 0.009 | 0.010 | 0.014 | 0.012 | 0.014 |
| meta334 |  | 217.475 | 258.268 | 0.027 | 0.027 | 0.025 | 0.026 | 0.025 | 0.025 | 0.036 | 0.033 | 0.018 | 0.013 | 0.011 | 0.015 | 0.019 | 0.013 | 0.024 |
| meta335 | 3,4-Dihydroxybenzoic acid | 218.044 | 152.965 | 0.004 | 0.005 | 0.004 | 0.002 | 0.008 | 0.002 | 0.002 | 0.006 | 0.004 | 0.006 | 0.006 | 0.003 | 0.004 | 0.004 | 0.006 |
| meta336 | L-Abrine | 218.101 | 137.859 | 0.035 | 0.044 | 0.036 | 0.034 | 0.049 | 0.033 | 0.145 | 0.133 | 0.030 | 0.041 | 0.024 | 0.036 | 0.057 | 0.037 | 0.060 |
| meta337 | Ala-Gln | 218.111 | 339.843 | 0.232 | 0.231 | 0.201 | 0.274 | 0.220 | 0.194 | 0.246 | 0.185 | 0.162 | 0.183 | 0.159 | 0.163 | 0.263 | 0.206 | 0.263 |
| meta338 |  | 218.137 | 145.039 | 0.047 | 0.047 | 0.043 | 0.037 | 0.064 | 0.029 | 0.027 | 0.081 | 0.051 | 0.026 | 0.067 | 0.070 | 0.048 | 0.038 | 0.086 |
| meta339 |  | 218.138 | 258.287 | 10.113 | 10.459 | 10.045 | 10.628 | 17.616 | 8.791 | 15.690 | 14.532 | 5.075 | 4.709 | 4.896 | 5.406 | 8.355 | 4.931 | 9.805 |
| meta340 | Ala-Lys | 218.148 | 409.701 | 0.079 | 0.093 | 0.105 | 0.088 | 0.063 | 0.112 | 0.111 | 0.053 | 0.084 | 0.062 | 0.067 | 0.067 | 0.120 | 0.074 | 0.079 |
| meta341 | Lavandulol | 218.153 | 34.162 | 0.010 | 0.008 | 0.011 | 0.021 | 0.015 | 0.011 | 0.021 | 0.058 | 0.028 | 0.013 | 0.022 | 0.016 | 0.014 | 0.010 | 0.009 |
| meta342 | Methyl 4-hydroxybenzoate | 219.004 | 456.245 | 0.018 | 0.014 | 0.017 | 0.015 | 0.017 | 0.017 | 0.014 | 0.021 | 0.016 | 0.015 | 0.016 | 0.017 | 0.017 | 0.014 | 0.016 |
| meta343 | Gly-Val | 219.074 | 46.855 | 0.008 | 0.009 | 0.009 | 0.008 | 0.009 | 0.007 | 0.032 | 0.013 | 0.025 | 0.010 | 0.011 | 0.038 | 0.007 | 0.009 | 0.011 |
| meta344 | 5-L-Glutamyl-L-alanine | 219.096 | 402.482 | 0.047 | 0.039 | 0.042 | 0.047 | 0.041 | 0.041 | 0.040 | 0.036 | 0.030 | 0.031 | 0.050 | 0.040 | 0.044 | 0.048 | 0.055 |
| meta345 | Ala-Glu | 219.096 | 374.930 | 0.047 | 0.046 | 0.045 | 0.040 | 0.069 | 0.045 | 0.075 | 0.036 | 0.044 | 0.035 | 0.049 | 0.041 | 0.042 | 0.025 | 0.030 |
| meta346 | N-Acetylserotonin | 219.111 | 40.012 | 0.030 | 0.030 | 0.027 | 0.044 | 0.039 | 0.028 | 0.035 | 0.031 | 0.031 | 0.037 | 0.038 | 0.031 | 0.035 | 0.029 | 0.053 |
| meta347 | Thr-Val | 219.132 | 439.617 | 0.030 | 0.034 | 0.033 | 0.030 | 0.037 | 0.030 | 0.036 | 0.027 | 0.035 | 0.026 | 0.027 | 0.030 | 0.039 | 0.027 | 0.035 |
| meta348 | Val-Thr | 219.132 | 345.642 | 0.013 | 0.015 | 0.013 | 0.015 | 0.018 | 0.020 | 0.016 | 0.019 | 0.021 | 0.019 | 0.019 | 0.015 | 0.013 | 0.018 | 0.015 |
| meta349 |  | 219.173 | 105.857 | 0.061 | 0.063 | 0.063 | 0.149 | 0.149 | 0.105 | 0.157 | 0.245 | 0.144 | 0.094 | 0.049 | 0.054 | 0.034 | 0.029 | 0.051 |
| meta350 |  | 220.104 | 351.990 | 0.253 | 0.320 | 0.277 | 0.224 | 0.267 | 0.172 | 0.238 | 0.274 | 0.404 | 0.332 | 0.269 | 0.323 | 0.263 | 0.290 | 0.315 |
| meta351 | Pantothenate | 220.116 | 256.257 | 0.216 | 0.253 | 0.179 | 0.116 | 0.360 | 0.092 | 0.360 | 0.365 | 0.192 | 0.347 | 0.333 | 0.393 | 0.232 | 0.175 | 0.300 |
| meta352 | Indole-3-pyruvic acid | 221.091 | 45.145 | 0.037 | 0.049 | 0.045 | 0.047 | 0.049 | 0.044 | 0.065 | 0.075 | 0.045 | 0.062 | 0.052 | 0.068 | 0.056 | 0.043 | 0.048 |
| meta353 | Thr-Thr | 221.112 | 304.465 | 0.022 | 0.026 | 0.022 | 0.029 | 0.025 | 0.023 | 0.029 | 0.016 | 0.013 | 0.015 | 0.014 | 0.016 | 0.025 | 0.017 | 0.027 |
| meta354 |  | 221.184 | 251.917 | 0.429 | 0.418 | 0.451 | 0.510 | 0.541 | 0.474 | 0.338 | 0.362 | 0.334 | 0.162 | 0.403 | 0.301 | 0.090 | 0.208 | 0.341 |
| meta355 |  | 222.078 | 393.210 | 0.233 | 0.227 | 0.224 | 0.178 | 0.303 | 0.180 | 0.230 | 0.186 | 0.185 | 0.187 | 0.265 | 0.228 | 0.383 | 0.248 | 0.273 |
| meta356 |  | 223.114 | 28.227 | 0.082 | 0.094 | 0.084 | 0.115 | 0.134 | 0.108 | 0.134 | 0.135 | 0.123 | 0.104 | 0.118 | 0.046 | 0.106 | 0.048 | 0.127 |
| meta357 |  | 223.994 | 26.992 | 0.066 | 0.078 | 0.071 | 0.076 | 0.079 | 0.049 | 0.067 | 0.085 | 0.069 | 0.068 | 0.072 | 0.074 | 0.077 | 0.077 | 0.077 |
| meta358 | Simazine | 224.065 | 437.641 | 0.083 | 0.072 | 0.093 | 0.058 | 0.069 | 0.074 | 0.068 | 0.071 | 0.069 | 0.085 | 0.066 | 0.093 | 0.069 | 0.079 | 0.092 |
| meta359 | L-Iditol | 224.111 | 336.240 | 0.375 | 0.330 | 0.328 | 0.406 | 0.437 | 0.311 | 0.355 | 0.425 | 0.406 | 0.276 | 0.297 | 0.282 | 0.454 | 0.375 | 0.228 |
| meta360 | Dulcitol | 224.111 | 314.607 | 0.289 | 0.261 | 0.303 | 0.250 | 0.353 | 0.207 | 0.296 | 0.452 | 0.182 | 0.154 | 0.143 | 0.222 | 0.238 | 0.301 | 0.263 |
| meta361 |  | 225.014 | 463.412 | 0.026 | 0.022 | 0.026 | 0.021 | 0.024 | 0.031 | 0.024 | 0.030 | 0.023 | 0.034 | 0.033 | 0.025 | 0.031 | 0.018 | 0.024 |
| meta362 |  | 226.082 | 362.500 | 5.073 | 6.593 | 5.394 | 6.110 | 6.892 | 3.978 | 7.410 | 5.161 | 4.624 | 5.346 | 6.650 | 5.587 | 4.485 | 4.244 | 7.852 |
| meta363 |  | 226.080 | 233.018 | 0.296 | 0.311 | 0.303 | 0.072 | 0.057 | 0.079 | 0.185 | 0.403 | 0.345 | 0.352 | 0.418 | 0.475 | 0.266 | 0.079 | 0.366 |
| meta364 | N1-Acetylspermidine | 226.128 | 490.442 | 0.006 | 0.008 | 0.006 | 0.025 | 0.008 | 0.005 | 0.007 | 0.007 | 0.011 | 0.011 | 0.008 | 0.008 | 0.007 | 0.007 | 0.007 |
| meta365 |  | 226.142 | 279.207 | 0.032 | 0.033 | 0.033 | 0.021 | 0.053 | 0.024 | 0.065 | 0.030 | 0.030 | 0.034 | 0.024 | 0.027 | 0.027 | 0.030 | 0.016 |
| meta366 |  | 226.178 | 120.556 | 0.632 | 0.552 | 0.611 | 0.858 | 0.733 | 0.589 | 0.555 | 0.732 | 0.758 | 0.745 | 0.472 | 0.622 | 0.809 | 0.652 | 1.365 |
| meta367 | Dimethyl sulfone | 226.986 | 318.923 | 0.014 | 0.013 | 0.015 | 0.011 | 0.013 | 0.017 | 0.014 | 0.011 | 0.017 | 0.013 | 0.013 | 0.013 | 0.019 | 0.017 | 0.013 |
| meta368 |  | 227.042 | 295.186 | 0.432 | 0.471 | 0.432 | 0.446 | 0.472 | 0.424 | 0.477 | 0.446 | 0.352 | 0.443 | 0.536 | 0.378 | 0.471 | 0.300 | 0.476 |
| meta369 | L-Carnosine | 227.112 | 396.812 | 0.057 | 0.043 | 0.043 | 0.054 | 0.052 | 0.032 | 0.036 | 0.060 | 0.046 | 0.051 | 0.049 | 0.058 | 0.041 | 0.040 | 0.041 |
| meta370 | Met-Leu | 227.124 | 156.925 | 0.020 | 0.022 | 0.020 | 0.018 | 0.024 | 0.022 | 0.026 | 0.027 | 0.025 | 0.020 | 0.023 | 0.012 | 0.015 | 0.013 | 0.020 |
| meta371 | Propoxur | 227.137 | 162.486 | 0.018 | 0.017 | 0.017 | 0.024 | 0.009 | 0.023 | 0.015 | 0.005 | 0.005 | 0.009 | 0.006 | 0.013 | 0.011 | 0.011 | 0.016 |
| meta372 | Lumazine | 228.044 | 294.946 | 0.036 | 0.035 | 0.036 | 0.035 | 0.041 | 0.034 | 0.037 | 0.036 | 0.029 | 0.034 | 0.042 | 0.031 | 0.039 | 0.024 | 0.039 |
| meta373 | D-Glucarate | 228.067 | 229.011 | 0.004 | 0.004 | 0.003 | 0.003 | 0.045 | 0.003 | 0.041 | 0.005 | 0.004 | 0.005 | 0.005 | 0.004 | 0.004 | 0.002 | 0.006 |
| meta374 | Carbimazole | 228.078 | 310.720 | 1.059 | 0.954 | 0.930 | 0.739 | 0.890 | 0.707 | 0.911 | 1.276 | 1.398 | 1.427 | 1.115 | 0.990 | 1.052 | 0.942 | 0.998 |
| meta375 | Deoxycytidine | 228.096 | 191.043 | 0.011 | 0.012 | 0.014 | 0.011 | 0.016 | 0.010 | 0.015 | 0.007 | 0.008 | 0.016 | 0.015 | 0.010 | 0.009 | 0.012 | 0.007 |
| meta376 | 2-Phenylbutyric acid | 228.096 | 304.389 | 0.035 | 0.032 | 0.032 | 0.034 | 0.023 | 0.026 | 0.022 | 0.030 | 0.037 | 0.031 | 0.029 | 0.029 | 0.035 | 0.020 | 0.025 |
| meta377 | N-epsilon,N-epsilon,N-epsilon-Trimethyllysine | 228.121 | 365.300 | 0.046 | 0.068 | 0.052 | 0.062 | 0.061 | 0.048 | 0.042 | 0.054 | 0.059 | 0.022 | 0.043 | 0.043 | 0.046 | 0.049 | 0.033 |
| meta378 |  | 228.169 | 316.742 | 0.016 | 0.014 | 0.014 | 0.004 | 0.009 | 0.002 | 0.026 | 0.035 | 0.023 | 0.010 | 0.007 | 0.018 | 0.013 | 0.005 | 0.007 |
| meta379 | Homogentisic acid | 229.068 | 49.213 | 0.013 | 0.021 | 0.017 | 0.018 | 0.089 | 0.014 | 0.112 | 0.014 | 0.015 | 0.020 | 0.017 | 0.015 | 0.014 | 0.014 | 0.017 |
| meta380 | Nicotine | 229.074 | 187.481 | 0.010 | 0.009 | 0.011 | 0.012 | 0.013 | 0.008 | 0.005 | 0.011 | 0.025 | 0.005 | 0.015 | 0.005 | 0.011 | 0.010 | 0.016 |
| meta381 |  | 229.117 | 401.969 | 0.380 | 0.371 | 0.334 | 0.478 | 0.387 | 0.374 | 0.401 | 0.299 | 0.194 | 0.262 | 0.260 | 0.230 | 0.288 | 0.223 | 0.246 |
| meta382 |  | 229.153 | 344.331 | 0.149 | 0.144 | 0.132 | 0.142 | 0.177 | 0.136 | 0.160 | 0.129 | 0.119 | 0.143 | 0.117 | 0.107 | 0.135 | 0.124 | 0.131 |
| meta383 |  | 229.153 | 280.886 | 0.172 | 0.181 | 0.194 | 0.174 | 0.206 | 0.157 | 0.196 | 0.155 | 0.154 | 0.187 | 0.155 | 0.126 | 0.170 | 0.159 | 0.149 |
| meta384 | Ergothioneine | 230.100 | 380.846 | 0.062 | 0.058 | 0.053 | 0.054 | 0.070 | 0.050 | 0.051 | 0.056 | 0.045 | 0.073 | 0.058 | 0.057 | 0.065 | 0.059 | 0.047 |
| meta385 | N6,N6,N6-Trimethyl-L-lysine | 230.184 | 503.766 | 0.010 | 0.011 | 0.011 | 0.008 | 0.014 | 0.011 | 0.023 | 0.006 | 0.021 | 0.007 | 0.006 | 0.005 | 0.020 | 0.013 | 0.007 |
| meta386 |  | 231.017 | 269.802 | 0.311 | 0.227 | 0.182 | 0.193 | 0.188 | 0.123 | 0.189 | 0.152 | 0.183 | 0.232 | 0.292 | 0.221 | 0.182 | 0.172 | 0.207 |
| meta387 |  | 231.089 | 230.900 | 0.400 | 0.503 | 0.492 | 0.355 | 0.434 | 0.321 | 0.428 | 0.502 | 0.875 | 0.607 | 0.510 | 0.497 | 0.537 | 0.541 | 0.613 |
| meta388 | Pro-Asp | 231.096 | 413.260 | 0.170 | 0.154 | 0.144 | 0.137 | 0.166 | 0.136 | 0.152 | 0.158 | 0.163 | 0.165 | 0.169 | 0.131 | 0.196 | 0.132 | 0.148 |
| meta389 | Levetiracetam | 231.132 | 264.153 | 0.040 | 0.039 | 0.040 | 0.023 | 0.051 | 0.020 | 0.042 | 0.049 | 0.028 | 0.040 | 0.047 | 0.040 | 0.030 | 0.031 | 0.024 |
| meta390 | Leu-Val | 231.168 | 183.763 | 0.064 | 0.063 | 0.051 | 0.076 | 0.056 | 0.053 | 0.078 | 0.024 | 0.020 | 0.016 | 0.027 | 0.036 | 0.048 | 0.026 | 0.074 |
| meta391 |  | 231.213 | 39.989 | 0.011 | 0.015 | 0.014 | 0.026 | 0.001 | 0.007 | 0.024 | 0.035 | 0.026 | 0.016 | 0.014 | 0.017 | 0.026 | 0.002 | 0.030 |
| meta392 | Pyridoxamine (PM) | 232.101 | 288.339 | 0.006 | 0.005 | 0.005 | 0.012 | 0.023 | 0.003 | 0.012 | 0.009 | 0.011 | 0.006 | 0.003 | 0.009 | 0.004 | 0.008 | 0.005 |
| meta393 | Gly-Arg | 232.135 | 263.942 | 0.003 | 0.003 | 0.003 | 0.015 | 0.020 | 0.012 | 0.003 | 0.015 | 0.013 | 0.004 | 0.004 | 0.005 | 0.004 | 0.003 | 0.019 |
| meta394 | N-(omega)-Hydroxyarginine | 232.139 | 413.361 | 0.032 | 0.049 | 0.052 | 0.050 | 0.041 | 0.044 | 0.044 | 0.037 | 0.047 | 0.030 | 0.032 | 0.035 | 0.058 | 0.047 | 0.041 |
| meta395 |  | 232.153 | 236.858 | 2.828 | 2.897 | 2.729 | 2.753 | 4.735 | 2.277 | 3.127 | 3.501 | 1.889 | 2.025 | 2.072 | 1.439 | 2.250 | 1.119 | 3.877 |
| meta396 |  | 232.189 | 50.212 | 0.024 | 0.023 | 0.026 | 0.030 | 0.028 | 0.014 | 0.027 | 0.025 | 0.020 | 0.017 | 0.023 | 0.025 | 0.023 | 0.019 | 0.037 |
| meta397 |  | 232.840 | 288.086 | 0.224 | 0.232 | 0.238 | 0.256 | 0.273 | 0.221 | 0.257 | 0.255 | 0.293 | 0.266 | 0.256 | 0.248 | 0.283 | 0.263 | 0.255 |
| meta398 |  | 233.061 | 268.936 | 0.152 | 0.170 | 0.144 | 0.167 | 0.163 | 0.145 | 0.162 | 0.211 | 0.100 | 0.140 | 0.174 | 0.162 | 0.140 | 0.117 | 0.116 |
| meta399 | Menadione (Vitamin K3) | 233.085 | 230.910 | 0.019 | 0.025 | 0.024 | 0.022 | 0.024 | 0.017 | 0.021 | 0.025 | 0.041 | 0.036 | 0.026 | 0.024 | 0.025 | 0.027 | 0.024 |
| meta400 | Nalidixic acid | 233.091 | 179.515 | 0.013 | 0.010 | 0.010 | 0.010 | 0.005 | 0.010 | 0.013 | 0.009 | 0.019 | 0.021 | 0.014 | 0.013 | 0.010 | 0.010 | 0.006 |
| meta401 | Kinetin | 233.115 | 36.086 | 0.022 | 0.016 | 0.018 | 0.024 | 0.017 | 0.019 | 0.024 | 0.029 | 0.017 | 0.013 | 0.020 | 0.015 | 0.016 | 0.014 | 0.012 |
| meta402 |  | 233.148 | 454.742 | 0.341 | 0.321 | 0.348 | 0.301 | 0.316 | 0.336 | 0.316 | 0.329 | 0.373 | 0.319 | 0.345 | 0.322 | 0.490 | 0.357 | 0.360 |
| meta403 |  | 233.152 | 120.629 | 0.099 | 0.097 | 0.074 | 0.185 | 0.144 | 0.159 | 0.237 | 0.155 | 0.145 | 0.146 | 0.072 | 0.096 | 0.083 | 0.062 | 0.119 |
| meta404 | Ile-Thr | 233.149 | 45.517 | 0.080 | 0.084 | 0.093 | 0.100 | 0.077 | 0.104 | 0.108 | 0.051 | 0.085 | 0.080 | 0.081 | 0.082 | 0.083 | 0.062 | 0.090 |
| meta405 | Propachlor | 234.065 | 269.583 | 0.011 | 0.016 | 0.010 | 0.011 | 0.014 | 0.010 | 0.013 | 0.018 | 0.008 | 0.011 | 0.013 | 0.017 | 0.009 | 0.010 | 0.012 |
| meta406 | Quinate | 234.096 | 424.550 | 1.294 | 2.290 | 2.166 | 0.802 | 2.505 | 3.171 | 2.452 | 0.828 | 0.854 | 2.158 | 2.182 | 3.604 | 2.506 | 0.617 | 1.003 |
| meta407 |  | 234.096 | 477.756 | 0.714 | 0.872 | 0.892 | 2.156 | 2.131 | 0.320 | 0.623 | 1.959 | 0.556 | 0.652 | 0.822 | 0.377 | 0.496 | 1.497 | 1.082 |
| meta408 |  | 234.916 | 318.454 | 0.278 | 0.271 | 0.342 | 0.267 | 0.285 | 0.340 | 0.296 | 0.262 | 0.347 | 0.283 | 0.295 | 0.259 | 0.356 | 0.402 | 0.277 |
| meta409 |  | 234.915 | 287.465 | 0.071 | 0.055 | 0.065 | 0.074 | 0.089 | 0.082 | 0.075 | 0.071 | 0.108 | 0.091 | 0.080 | 0.082 | 0.135 | 0.090 | 0.085 |
| meta410 |  | 235.012 | 156.795 | 0.025 | 0.030 | 0.027 | 0.030 | 0.036 | 0.027 | 0.031 | 0.019 | 0.020 | 0.023 | 0.026 | 0.024 | 0.029 | 0.023 | 0.020 |
| meta411 | 5-Methoxytryptamine | 235.077 | 311.453 | 0.010 | 0.016 | 0.013 | 0.018 | 0.015 | 0.008 | 0.016 | 0.010 | 0.011 | 0.009 | 0.008 | 0.009 | 0.012 | 0.012 | 0.005 |
| meta412 | Ser-Met | 236.078 | 352.027 | 0.094 | 0.107 | 0.077 | 0.085 | 0.095 | 0.068 | 0.087 | 0.102 | 0.143 | 0.109 | 0.098 | 0.108 | 0.095 | 0.095 | 0.102 |
| meta413 |  | 236.147 | 306.564 | 0.095 | 0.133 | 0.097 | 0.107 | 0.107 | 0.079 | 0.121 | 0.120 | 0.123 | 0.113 | 0.106 | 0.098 | 0.115 | 0.104 | 0.097 |
| meta414 | Phe-Ala | 237.121 | 192.278 | 0.007 | 0.006 | 0.006 | 0.007 | 0.006 | 0.009 | 0.010 | 0.005 | 0.003 | 0.005 | 0.005 | 0.006 | 0.006 | 0.004 | 0.004 |
| meta415 | Bethanechol cation | 238.043 | 372.261 | 0.291 | 0.299 | 0.257 | 0.302 | 0.322 | 0.217 | 0.364 | 0.326 | 0.197 | 0.316 | 0.383 | 0.220 | 0.196 | 0.189 | 0.319 |
| meta416 | Tyr-Gly | 238.092 | 142.508 | 0.088 | 0.136 | 0.117 | 0.157 | 0.157 | 0.069 | 0.164 | 0.109 | 0.103 | 0.129 | 0.113 | 0.042 | 0.123 | 0.105 | 0.152 |
| meta417 | Biopterin | 238.092 | 236.238 | 0.037 | 0.041 | 0.042 | 0.039 | 0.036 | 0.045 | 0.031 | 0.039 | 0.037 | 0.043 | 0.042 | 0.030 | 0.040 | 0.037 | 0.035 |
| meta418 |  | 238.874 | 286.566 | 0.050 | 0.045 | 0.037 | 0.037 | 0.052 | 0.039 | 0.043 | 0.047 | 0.047 | 0.048 | 0.047 | 0.054 | 0.048 | 0.039 | 0.047 |
| meta419 |  | 239.000 | 564.882 | 0.229 | 0.216 | 0.188 | 0.231 | 0.288 | 0.113 | 0.311 | 0.130 | 0.200 | 0.283 | 0.212 | 0.164 | 0.093 | 0.265 | 0.184 |
| meta420 | Bupropion | 239.108 | 27.038 | 0.018 | 0.020 | 0.022 | 0.019 | 0.020 | 0.014 | 0.018 | 0.023 | 0.019 | 0.019 | 0.019 | 0.021 | 0.026 | 0.023 | 0.024 |
| meta421 |  | 239.168 | 27.545 | 0.019 | 0.021 | 0.022 | 0.025 | 0.022 | 0.016 | 0.019 | 0.028 | 0.027 | 0.019 | 0.024 | 0.022 | 0.021 | 0.024 | 0.023 |
| meta422 | Mephentermine | 240.052 | 170.248 | 0.008 | 0.014 | 0.010 | 0.005 | 0.050 | 0.006 | 0.054 | 0.007 | 0.009 | 0.009 | 0.007 | 0.008 | 0.008 | 0.006 | 0.006 |
| meta423 | Gly-Thr | 240.100 | 199.881 | 0.286 | 0.280 | 0.294 | 0.214 | 0.299 | 0.187 | 0.244 | 0.345 | 0.317 | 0.295 | 0.346 | 0.312 | 0.292 | 0.287 | 0.393 |
| meta424 |  | 240.230 | 152.295 | 0.023 | 0.028 | 0.025 | 0.031 | 0.031 | 0.021 | 0.022 | 0.024 | 0.025 | 0.018 | 0.017 | 0.019 | 0.019 | 0.017 | 0.027 |
| meta425 |  | 241.153 | 349.970 | 0.095 | 0.090 | 0.083 | 0.099 | 0.128 | 0.096 | 0.086 | 0.077 | 0.067 | 0.060 | 0.060 | 0.056 | 0.077 | 0.105 | 0.072 |
| meta426 |  | 242.056 | 154.404 | 0.014 | 0.018 | 0.013 | 0.010 | 0.013 | 0.015 | 0.014 | 0.017 | 0.019 | 0.017 | 0.017 | 0.016 | 0.019 | 0.013 | 0.014 |
| meta427 |  | 242.078 | 391.419 | 1.358 | 1.732 | 1.520 | 1.537 | 1.848 | 0.881 | 2.282 | 1.299 | 0.914 | 1.503 | 2.313 | 1.563 | 0.843 | 0.766 | 2.600 |
| meta428 | 3-Hydroxykynurenine | 242.112 | 213.850 | 0.142 | 0.125 | 0.141 | 0.121 | 0.161 | 0.097 | 0.160 | 0.165 | 0.099 | 0.111 | 0.120 | 0.146 | 0.162 | 0.102 | 0.105 |
| meta429 |  | 242.159 | 271.818 | 0.021 | 0.019 | 0.016 | 0.015 | 0.007 | 0.013 | 0.047 | 0.037 | 0.029 | 0.012 | 0.011 | 0.034 | 0.018 | 0.006 | 0.008 |
| meta430 | D-Glucose 6-phosphate | 243.024 | 314.924 | 0.007 | 0.005 | 0.005 | 0.005 | 0.004 | 0.005 | 0.004 | 0.007 | 0.005 | 0.008 | 0.006 | 0.005 | 0.005 | 0.005 | 0.006 |
| meta431 | Mimosine | 243.041 | 434.576 | 0.072 | 0.125 | 0.086 | 0.074 | 0.132 | 0.063 | 0.077 | 0.088 | 0.158 | 0.156 | 0.149 | 0.075 | 0.088 | 0.067 | 0.087 |
| meta432 | 1-Methyluric acid | 243.072 | 371.901 | 0.025 | 0.025 | 0.021 | 0.028 | 0.027 | 0.017 | 0.028 | 0.030 | 0.013 | 0.021 | 0.032 | 0.019 | 0.037 | 0.014 | 0.026 |
| meta433 | Lumichrome | 243.086 | 48.770 | 0.106 | 0.176 | 0.153 | 0.233 | 0.267 | 0.193 | 0.230 | 0.160 | 0.205 | 0.158 | 0.193 | 0.191 | 0.176 | 0.186 | 0.256 |
| meta434 | His-Ser | 243.106 | 346.876 | 0.053 | 0.059 | 0.056 | 0.049 | 0.041 | 0.039 | 0.043 | 0.054 | 0.050 | 0.053 | 0.049 | 0.009 | 0.038 | 0.022 | 0.049 |
| meta435 | Dacarbazine | 243.118 | 318.779 | 0.012 | 0.017 | 0.015 | 0.015 | 0.016 | 0.011 | 0.012 | 0.014 | 0.016 | 0.015 | 0.014 | 0.014 | 0.017 | 0.014 | 0.015 |
| meta436 |  | 243.121 | 25.811 | 0.038 | 0.041 | 0.049 | 0.085 | 0.046 | 0.056 | 0.073 | 0.119 | 0.102 | 0.091 | 0.053 | 0.028 | 0.019 | 0.029 | 0.021 |
| meta437 |  | 243.143 | 306.625 | 0.075 | 0.071 | 0.057 | 0.015 | 0.036 | 0.037 | 0.024 | 0.137 | 0.138 | 0.217 | 0.059 | 0.096 | 0.050 | 0.096 | 0.044 |
| meta438 | Lys-Asn | 243.142 | 479.892 | 0.083 | 0.086 | 0.102 | 0.094 | 0.084 | 0.088 | 0.095 | 0.087 | 0.085 | 0.066 | 0.070 | 0.072 | 0.105 | 0.091 | 0.090 |
| meta439 |  | 243.331 | 352.162 | 0.009 | 0.010 | 0.007 | 0.007 | 0.009 | 0.006 | 0.005 | 0.007 | 0.011 | 0.010 | 0.008 | 0.008 | 0.007 | 0.009 | 0.010 |
| meta440 |  | 244.074 | 352.027 | 2.588 | 3.226 | 2.832 | 2.373 | 3.095 | 1.917 | 2.830 | 3.412 | 5.122 | 3.983 | 3.367 | 3.284 | 3.254 | 3.178 | 3.609 |
| meta441 | D-Mannose | 244.077 | 238.964 | 0.436 | 0.408 | 0.377 | 0.327 | 0.493 | 0.564 | 0.507 | 0.371 | 0.263 | 0.305 | 0.349 | 0.410 | 0.296 | 0.391 | 0.289 |
| meta442 | Cytidine | 244.091 | 222.482 | 0.123 | 0.123 | 0.108 | 0.085 | 0.111 | 0.113 | 0.117 | 0.100 | 0.106 | 0.123 | 0.142 | 0.144 | 0.112 | 0.114 | 0.094 |
| meta443 |  | 244.110 | 282.236 | 0.078 | 0.094 | 0.092 | 0.053 | 0.056 | 0.054 | 0.052 | 0.075 | 0.160 | 0.133 | 0.096 | 0.062 | 0.102 | 0.101 | 0.094 |
| meta444 |  | 244.189 | 120.519 | 0.211 | 0.188 | 0.201 | 0.315 | 0.291 | 0.234 | 0.231 | 0.227 | 0.274 | 0.267 | 0.154 | 0.177 | 0.287 | 0.278 | 0.540 |
| meta445 | Myristoleic acid | 244.225 | 38.946 | 0.024 | 0.026 | 0.029 | 0.032 | 0.036 | 0.025 | 0.037 | 0.039 | 0.038 | 0.035 | 0.031 | 0.047 | 0.030 | 0.033 | 0.069 |
| meta446 |  | 244.360 | 310.749 | 0.040 | 0.031 | 0.034 | 0.024 | 0.030 | 0.024 | 0.027 | 0.049 | 0.044 | 0.045 | 0.039 | 0.030 | 0.036 | 0.035 | 0.036 |
| meta447 |  | 245.057 | 434.685 | 0.234 | 0.280 | 0.310 | 0.293 | 0.358 | 0.205 | 0.411 | 0.163 | 0.321 | 0.244 | 0.343 | 0.231 | 0.313 | 0.142 | 0.183 |
| meta448 |  | 245.057 | 221.125 | 0.275 | 0.251 | 0.259 | 0.222 | 0.339 | 0.212 | 0.353 | 0.274 | 0.280 | 0.291 | 0.230 | 0.249 | 0.289 | 0.221 | 0.258 |
| meta449 | Uridine | 245.075 | 152.578 | 0.075 | 0.089 | 0.103 | 0.079 | 0.132 | 0.081 | 0.157 | 0.099 | 0.062 | 0.047 | 0.073 | 0.079 | 0.074 | 0.077 | 0.087 |
| meta450 | Monoethylglycylxylidide (MEGX) | 245.106 | 310.660 | 14.901 | 13.641 | 12.346 | 10.125 | 12.765 | 9.710 | 13.119 | 15.898 | 19.130 | 19.107 | 15.233 | 13.836 | 13.368 | 12.036 | 13.588 |
| meta451 | Pro-Glu | 245.112 | 404.027 | 0.273 | 0.278 | 0.260 | 0.269 | 0.277 | 0.235 | 0.276 | 0.280 | 0.241 | 0.261 | 0.263 | 0.219 | 0.270 | 0.214 | 0.248 |
| meta452 | Ile-Ile | 245.184 | 168.877 | 0.021 | 0.025 | 0.025 | 0.046 | 0.012 | 0.039 | 0.026 | 0.010 | 0.008 | 0.011 | 0.009 | 0.009 | 0.023 | 0.014 | 0.021 |
| meta453 | Leu-Leu | 245.185 | 37.571 | 0.052 | 0.056 | 0.050 | 0.077 | 0.057 | 0.008 | 0.043 | 0.036 | 0.032 | 0.039 | 0.062 | 0.019 | 0.054 | 0.060 | 0.079 |
| meta454 | 3,4-Methylenedioxyamphetamine | 246.042 | 31.806 | 0.003 | 0.004 | 0.004 | 0.001 | 0.002 | 0.001 | 0.006 | 0.002 | 0.007 | 0.001 | 0.007 | 0.002 | 0.007 | 0.004 | 0.002 |
| meta455 | Phenylpropionylglycine | 246.052 | 357.981 | 0.004 | 0.005 | 0.004 | 0.016 | 0.021 | 0.018 | 0.017 | 0.007 | 0.018 | 0.005 | 0.020 | 0.009 | 0.018 | 0.018 | 0.004 |
| meta456 | Mexiletine | 246.089 | 232.328 | 0.165 | 0.185 | 0.177 | 0.115 | 0.178 | 0.128 | 0.171 | 0.230 | 0.240 | 0.241 | 0.183 | 0.174 | 0.186 | 0.169 | 0.196 |
| meta457 | Pro-Met | 246.108 | 310.723 | 1.330 | 1.220 | 1.199 | 0.865 | 1.020 | 0.904 | 1.125 | 1.544 | 1.740 | 1.761 | 1.410 | 1.163 | 1.388 | 1.158 | 1.258 |
| meta458 | 2'-O-methyladenosine | 246.099 | 27.545 | 0.002 | 0.002 | 0.002 | 0.002 | 0.002 | 0.001 | 0.014 | 0.038 | 0.016 | 0.002 | 0.002 | 0.002 | 0.002 | 0.014 | 0.043 |
| meta459 | Val-Gln | 246.143 | 348.224 | 0.026 | 0.017 | 0.019 | 0.020 | 0.024 | 0.019 | 0.023 | 0.024 | 0.024 | 0.020 | 0.027 | 0.020 | 0.026 | 0.021 | 0.022 |
| meta460 | Arg-Ala | 246.154 | 391.964 | 0.085 | 0.109 | 0.136 | 0.110 | 0.083 | 0.105 | 0.113 | 0.076 | 0.098 | 0.072 | 0.080 | 0.082 | 0.116 | 0.084 | 0.098 |
| meta461 | 2-Methylbutyroylcarnitine | 246.169 | 221.001 | 2.211 | 2.368 | 2.234 | 2.778 | 4.071 | 1.913 | 4.431 | 2.257 | 1.009 | 0.917 | 1.126 | 1.080 | 1.180 | 0.874 | 1.820 |
| meta462 | Lys-Val | 246.180 | 355.231 | 0.015 | 0.011 | 0.012 | 0.012 | 0.010 | 0.015 | 0.013 | 0.006 | 0.011 | 0.006 | 0.008 | 0.007 | 0.014 | 0.008 | 0.011 |
| meta463 | Myristic acid | 246.241 | 50.190 | 0.018 | 0.017 | 0.016 | 0.019 | 0.016 | 0.038 | 0.021 | 0.007 | 0.031 | 0.016 | 0.016 | 0.019 | 0.030 | 0.037 | 0.022 |
| meta464 | L-homocysteic acid | 247.038 | 434.754 | 0.008 | 0.008 | 0.007 | 0.007 | 0.006 | 0.007 | 0.009 | 0.010 | 0.011 | 0.010 | 0.008 | 0.004 | 0.010 | 0.006 | 0.010 |
| meta465 |  | 247.056 | 331.033 | 0.285 | 0.265 | 0.263 | 0.200 | 0.252 | 0.198 | 0.278 | 0.289 | 0.261 | 0.349 | 0.338 | 0.219 | 0.208 | 0.190 | 0.262 |
| meta466 |  | 247.093 | 431.385 | 8.885 | 6.139 | 5.830 | 5.699 | 5.635 | 4.582 | 5.626 | 10.117 | 6.199 | 5.631 | 9.650 | 6.223 | 10.284 | 6.144 | 6.776 |
| meta467 |  | 247.092 | 417.084 | 1.856 | 1.897 | 1.869 | 1.702 | 1.913 | 1.592 | 1.991 | 2.047 | 1.735 | 1.668 | 1.980 | 2.068 | 1.895 | 1.757 | 1.921 |
| meta468 | gamma-L-Glutamyl-L-valine | 247.127 | 364.735 | 0.126 | 0.146 | 0.103 | 0.146 | 0.138 | 0.144 | 0.121 | 0.112 | 0.106 | 0.095 | 0.123 | 0.083 | 0.123 | 0.071 | 0.023 |
| meta469 | Lys-Thr | 247.157 | 392.303 | 0.009 | 0.012 | 0.013 | 0.014 | 0.009 | 0.011 | 0.012 | 0.005 | 0.009 | 0.005 | 0.009 | 0.008 | 0.012 | 0.010 | 0.011 |
| meta470 |  | 247.394 | 298.110 | 0.025 | 0.027 | 0.023 | 0.020 | 0.041 | 0.021 | 0.033 | 0.024 | 0.012 | 0.013 | 0.021 | 0.012 | 0.029 | 0.017 | 0.029 |
| meta471 |  | 248.112 | 375.685 | 1.262 | 1.281 | 1.110 | 1.154 | 1.800 | 0.863 | 1.458 | 1.094 | 0.982 | 1.123 | 0.994 | 1.134 | 1.310 | 0.936 | 1.322 |
| meta472 |  | 248.111 | 108.353 | 0.070 | 0.093 | 0.082 | 0.127 | 0.166 | 0.103 | 0.131 | 0.106 | 0.059 | 0.084 | 0.059 | 0.066 | 0.109 | 0.094 | 0.130 |
| meta473 |  | 248.149 | 298.018 | 9.936 | 9.979 | 8.915 | 8.142 | 15.931 | 7.520 | 12.720 | 10.924 | 5.403 | 5.642 | 8.385 | 4.509 | 11.553 | 6.430 | 12.617 |
| meta474 | N-Acetylglutamine | 249.106 | 403.199 | 0.072 | 0.069 | 0.067 | 0.071 | 0.083 | 0.084 | 0.088 | 0.064 | 0.062 | 0.048 | 0.072 | 0.079 | 0.076 | 0.033 | 0.051 |
| meta475 | Thr-Glu | 249.114 | 375.729 | 0.129 | 0.135 | 0.118 | 0.125 | 0.179 | 0.090 | 0.144 | 0.118 | 0.101 | 0.122 | 0.100 | 0.119 | 0.130 | 0.111 | 0.138 |
| meta476 | Val-Met | 249.125 | 188.489 | 0.009 | 0.008 | 0.007 | 0.007 | 0.011 | 0.010 | 0.009 | 0.009 | 0.007 | 0.005 | 0.008 | 0.007 | 0.007 | 0.007 | 0.007 |
| meta477 |  | 249.332 | 96.923 | 0.052 | 0.090 | 0.072 | 0.092 | 0.133 | 0.075 | 0.155 | 0.087 | 0.049 | 0.048 | 0.041 | 0.044 | 0.064 | 0.075 | 0.072 |
| meta478 |  | 250.090 | 340.548 | 0.163 | 0.167 | 0.163 | 0.214 | 0.218 | 0.146 | 0.197 | 0.187 | 0.119 | 0.144 | 0.166 | 0.184 | 0.165 | 0.118 | 0.096 |
| meta479 |  | 250.103 | 157.429 | 0.068 | 0.072 | 0.063 | 0.069 | 0.075 | 0.061 | 0.069 | 0.059 | 0.039 | 0.049 | 0.063 | 0.054 | 0.055 | 0.053 | 0.046 |
| meta480 |  | 250.109 | 385.078 | 0.082 | 0.072 | 0.073 | 0.077 | 0.096 | 0.067 | 0.086 | 0.068 | 0.056 | 0.063 | 0.054 | 0.051 | 0.081 | 0.069 | 0.051 |
| meta481 |  | 251.008 | 26.822 | 0.238 | 0.253 | 0.250 | 0.267 | 0.273 | 0.167 | 0.225 | 0.285 | 0.242 | 0.228 | 0.242 | 0.277 | 0.258 | 0.261 | 0.261 |
| meta482 |  | 251.035 | 275.257 | 1.143 | 1.081 | 0.997 | 0.956 | 1.240 | 0.939 | 0.826 | 1.545 | 1.006 | 1.643 | 1.016 | 0.519 | 1.028 | 1.109 | 1.029 |
| meta483 | Nname,cis-Vaccenic acid | 251.095 | 96.923 | 2.069 | 3.329 | 2.601 | 3.702 | 5.641 | 2.880 | 5.819 | 3.965 | 1.963 | 1.738 | 1.940 | 1.754 | 2.528 | 3.017 | 2.846 |
| meta484 |  | 251.205 | 27.918 | 0.040 | 0.050 | 0.046 | 0.056 | 0.051 | 0.033 | 0.044 | 0.062 | 0.050 | 0.042 | 0.050 | 0.055 | 0.046 | 0.052 | 0.051 |
| meta485 | D-Galactarate | 252.071 | 396.673 | 0.126 | 0.141 | 0.131 | 0.124 | 0.165 | 0.105 | 0.116 | 0.081 | 0.109 | 0.102 | 0.124 | 0.117 | 0.152 | 0.144 | 0.161 |
| meta486 |  | 252.076 | 304.755 | 0.100 | 0.073 | 0.076 | 0.037 | 0.049 | 0.049 | 0.057 | 0.088 | 0.076 | 0.110 | 0.063 | 0.053 | 0.074 | 0.042 | 0.061 |
| meta487 | Asn-Pro | 252.097 | 97.124 | 0.200 | 0.363 | 0.284 | 0.383 | 0.581 | 0.322 | 0.631 | 0.429 | 0.229 | 0.189 | 0.201 | 0.204 | 0.293 | 0.318 | 0.298 |
| meta488 |  | 252.926 | 318.614 | 0.033 | 0.034 | 0.039 | 0.031 | 0.033 | 0.043 | 0.035 | 0.030 | 0.041 | 0.033 | 0.034 | 0.029 | 0.038 | 0.047 | 0.031 |
| meta489 | Harmaline | 253.079 | 305.090 | 0.012 | 0.009 | 0.012 | 0.009 | 0.009 | 0.008 | 0.009 | 0.012 | 0.010 | 0.016 | 0.010 | 0.009 | 0.010 | 0.011 | 0.010 |
| meta490 | Deoxyinosine | 253.091 | 168.280 | 0.017 | 0.014 | 0.019 | 0.021 | 0.015 | 0.017 | 0.010 | 0.008 | 0.010 | 0.014 | 0.018 | 0.010 | 0.008 | 0.007 | 0.013 |
| meta491 | Phe-Ser | 253.116 | 219.962 | 0.018 | 0.017 | 0.017 | 0.023 | 0.017 | 0.022 | 0.021 | 0.010 | 0.009 | 0.012 | 0.010 | 0.010 | 0.019 | 0.012 | 0.015 |
| meta492 | 5-Amino-4-carbamoylimidazole (AICA) | 253.117 | 47.081 | 0.032 | 0.040 | 0.037 | 0.039 | 0.036 | 0.034 | 0.033 | 0.027 | 0.030 | 0.026 | 0.040 | 0.021 | 0.034 | 0.030 | 0.033 |
| meta493 |  | 253.126 | 248.179 | 0.035 | 0.046 | 0.052 | 0.040 | 0.021 | 0.032 | 0.041 | 0.029 | 0.031 | 0.037 | 0.029 | 0.030 | 0.034 | 0.025 | 0.028 |
| meta494 |  | 254.149 | 399.456 | 0.882 | 0.724 | 0.795 | 0.827 | 0.681 | 0.761 | 0.815 | 0.896 | 0.797 | 0.954 | 0.718 | 0.928 | 0.982 | 0.593 | 0.842 |
| meta495 | 3-Hydroxyisovaleric acid | 254.158 | 255.758 | 0.008 | 0.008 | 0.008 | 0.002 | 0.002 | 0.001 | 0.005 | 0.005 | 0.005 | 0.011 | 0.002 | 0.004 | 0.005 | 0.007 | 0.003 |
| meta496 |  | 254.171 | 49.715 | 0.046 | 0.035 | 0.037 | 0.041 | 0.032 | 0.020 | 0.031 | 0.032 | 0.025 | 0.023 | 0.039 | 0.032 | 0.032 | 0.029 | 0.046 |
| meta497 | Daidzein | 255.063 | 46.169 | 0.045 | 0.055 | 0.056 | 0.093 | 0.021 | 0.086 | 0.095 | 0.111 | 0.085 | 0.105 | 0.081 | 0.035 | 0.021 | 0.016 | 0.015 |
| meta498 | D-Pinitol | 255.107 | 318.967 | 0.047 | 0.045 | 0.040 | 0.017 | 0.077 | 0.013 | 0.096 | 0.014 | 0.034 | 0.029 | 0.008 | 0.019 | 0.064 | 0.017 | 0.023 |
| meta499 | His-Val | 255.143 | 299.875 | 0.031 | 0.043 | 0.036 | 0.049 | 0.029 | 0.044 | 0.036 | 0.020 | 0.019 | 0.024 | 0.023 | 0.022 | 0.034 | 0.023 | 0.041 |
| meta500 |  | 256.037 | 292.491 | 0.055 | 0.066 | 0.070 | 0.063 | 0.055 | 0.048 | 0.064 | 0.052 | 0.084 | 0.070 | 0.055 | 0.046 | 0.057 | 0.053 | 0.063 |
| meta501 |  | 256.068 | 339.263 | 0.040 | 0.037 | 0.030 | 0.049 | 0.036 | 0.034 | 0.024 | 0.034 | 0.023 | 0.031 | 0.391 | 0.261 | 0.043 | 0.032 | 0.509 |
| meta502 |  | 256.093 | 384.367 | 0.619 | 0.622 | 0.538 | 0.546 | 0.759 | 0.372 | 0.944 | 0.578 | 0.381 | 0.569 | 0.858 | 0.574 | 0.456 | 0.330 | 0.903 |
| meta503 | 5-Hydroxymethylcytidine | 256.093 | 340.607 | 0.852 | 0.891 | 0.797 | 0.865 | 1.094 | 0.584 | 1.311 | 0.887 | 0.512 | 0.844 | 1.248 | 0.822 | 0.609 | 0.503 | 1.414 |
| meta504 |  | 256.262 | 33.990 | 0.060 | 0.075 | 0.057 | 0.162 | 0.107 | 0.109 | 0.160 | 0.135 | 0.055 | 0.103 | 0.080 | 0.142 | 0.132 | 0.142 | 0.141 |
| meta505 |  | 256.298 | 119.863 | 1.749 | 2.157 | 1.858 | 1.276 | 1.628 | 1.203 | 2.379 | 2.030 | 1.611 | 1.394 | 1.417 | 1.538 | 1.745 | 1.934 | 1.395 |
| meta506 |  | 256.299 | 575.399 | 1.411 | 1.455 | 1.395 | 1.431 | 1.275 | 1.170 | 1.480 | 1.493 | 1.479 | 1.236 | 1.493 | 1.361 | 1.306 | 1.294 | 1.570 |
| meta507 |  | 256.320 | 27.918 | 0.166 | 0.215 | 0.233 | 0.228 | 0.212 | 0.164 | 0.195 | 0.270 | 0.214 | 0.177 | 0.211 | 0.208 | 0.200 | 0.239 | 0.216 |
| meta508 | His-Thr | 257.122 | 334.950 | 0.103 | 0.097 | 0.078 | 0.129 | 0.061 | 0.096 | 0.070 | 0.072 | 0.041 | 0.083 | 0.088 | 0.092 | 0.085 | 0.079 | 0.092 |
| meta509 |  | 257.145 | 252.519 | 2.088 | 2.271 | 2.170 | 2.159 | 2.079 | 2.060 | 1.665 | 2.007 | 2.116 | 1.462 | 2.137 | 1.614 | 0.899 | 1.405 | 2.198 |
| meta510 |  | 258.071 | 48.940 | 0.052 | 0.082 | 0.061 | 0.002 | 0.010 | 0.010 | 0.067 | 0.440 | 0.080 | 0.066 | 0.178 | 0.172 | 0.052 | 0.013 | 0.061 |
| meta511 | 5-Methylcytidine | 258.106 | 227.886 | 0.040 | 0.042 | 0.033 | 0.039 | 0.051 | 0.034 | 0.040 | 0.038 | 0.031 | 0.052 | 0.040 | 0.028 | 0.031 | 0.032 | 0.033 |
| meta512 | Glycerophosphocholine | 258.108 | 383.380 | 5.015 | 4.436 | 3.667 | 3.492 | 4.911 | 3.118 | 6.455 | 4.037 | 2.490 | 4.586 | 6.067 | 4.199 | 2.728 | 2.282 | 6.183 |
| meta513 |  | 258.110 | 362.157 | 230.921 | 249.290 | 226.012 | 220.733 | 267.873 | 166.059 | 296.342 | 225.143 | 167.577 | 219.737 | 260.483 | 229.286 | 176.804 | 146.600 | 307.133 |
| meta514 | 2'-O-methylcytidine | 258.107 | 160.570 | 0.029 | 0.029 | 0.017 | 0.017 | 0.037 | 0.015 | 0.037 | 0.027 | 0.015 | 0.016 | 0.026 | 0.027 | 0.010 | 0.013 | 0.026 |
| meta515 | N-Acetylcadaverine | 258.995 | 361.594 | 0.117 | 0.087 | 0.094 | 0.131 | 0.124 | 0.060 | 0.206 | 0.063 | 0.040 | 0.112 | 0.157 | 0.126 | 0.060 | 0.051 | 0.200 |
| meta516 |  | 259.036 | 312.009 | 0.363 | 0.397 | 0.430 | 0.370 | 0.304 | 0.266 | 0.437 | 0.373 | 0.470 | 0.472 | 0.337 | 0.293 | 0.399 | 0.304 | 0.389 |
| meta517 | Erythrono-1,4-lactone | 259.037 | 434.793 | 0.019 | 0.026 | 0.023 | 0.021 | 0.022 | 0.019 | 0.024 | 0.026 | 0.024 | 0.026 | 0.023 | 0.018 | 0.024 | 0.019 | 0.021 |
| meta518 | Ribothymidine | 259.091 | 336.019 | 0.227 | 0.239 | 0.212 | 0.134 | 0.175 | 0.131 | 0.290 | 0.359 | 0.295 | 0.337 | 0.370 | 0.257 | 0.162 | 0.237 | 0.198 |
| meta519 | L-Cysteine | 260.068 | 419.473 | 0.097 | 0.064 | 0.073 | 0.100 | 0.070 | 0.051 | 0.064 | 0.061 | 0.072 | 0.153 | 0.082 | 0.107 | 0.069 | 0.045 | 0.060 |
| meta520 | Gln-Asn | 260.113 | 362.180 | 2.671 | 2.919 | 2.352 | 2.483 | 3.017 | 1.933 | 3.316 | 2.609 | 1.959 | 2.608 | 3.126 | 2.466 | 2.124 | 1.744 | 3.392 |
| meta521 | Ile-Ser | 260.158 | 359.879 | 0.008 | 0.010 | 0.006 | 0.012 | 0.009 | 0.009 | 0.010 | 0.008 | 0.013 | 0.014 | 0.009 | 0.019 | 0.009 | 0.007 | 0.020 |
| meta522 | Lys-Leu | 260.195 | 490.683 | 0.015 | 0.020 | 0.024 | 0.019 | 0.019 | 0.024 | 0.022 | 0.017 | 0.022 | 0.016 | 0.016 | 0.015 | 0.029 | 0.024 | 0.021 |
| meta523 |  | 261.047 | 270.636 | 0.085 | 0.087 | 0.079 | 0.097 | 0.110 | 0.075 | 0.166 | 0.122 | 0.074 | 0.064 | 0.062 | 0.080 | 0.072 | 0.061 | 0.079 |
| meta524 |  | 261.126 | 51.747 | 0.090 | 0.087 | 0.094 | 0.099 | 0.106 | 0.096 | 0.099 | 0.092 | 0.125 | 0.097 | 0.091 | 0.081 | 0.071 | 0.086 | 0.063 |
| meta525 |  | 261.144 | 399.301 | 13.898 | 13.031 | 14.282 | 12.747 | 14.203 | 10.921 | 12.619 | 14.441 | 14.377 | 12.002 | 13.215 | 13.239 | 19.277 | 12.496 | 13.890 |
| meta526 |  | 261.179 | 252.498 | 0.395 | 0.407 | 0.460 | 0.478 | 0.543 | 0.423 | 0.154 | 0.330 | 0.618 | 0.400 | 0.370 | 0.076 | 0.054 | 0.123 | 0.475 |
| meta527 |  | 261.187 | 203.852 | 0.050 | 0.045 | 0.048 | 0.034 | 0.093 | 0.027 | 0.072 | 0.081 | 0.034 | 0.023 | 0.029 | 0.018 | 0.040 | 0.025 | 0.065 |
| meta528 |  | 261.215 | 244.735 | 0.104 | 0.096 | 0.119 | 0.157 | 0.258 | 0.140 | 0.053 | 0.068 | 0.104 | 0.073 | 0.081 | 0.029 | 0.006 | 0.025 | 0.059 |
| meta529 |  | 262.049 | 467.260 | 5.400 | 4.815 | 6.569 | 5.407 | 7.793 | 5.594 | 5.212 | 8.399 | 4.827 | 4.370 | 4.915 | 5.199 | 4.100 | 5.360 | 5.099 |
| meta530 | L-Fucose-1-phosphate | 262.067 | 232.290 | 0.014 | 0.010 | 0.010 | 0.007 | 0.010 | 0.009 | 0.012 | 0.015 | 0.009 | 0.014 | 0.018 | 0.014 | 0.011 | 0.011 | 0.007 |
| meta531 | D-Biotin | 262.128 | 384.845 | 2.176 | 2.012 | 1.859 | 1.686 | 2.636 | 1.244 | 1.904 | 4.334 | 1.542 | 1.464 | 1.796 | 3.821 | 2.248 | 1.235 | 3.798 |
| meta532 | Ser-Arg | 262.147 | 399.309 | 1.570 | 1.507 | 1.566 | 1.437 | 1.527 | 1.173 | 1.475 | 1.629 | 1.675 | 1.319 | 1.515 | 1.494 | 2.245 | 1.458 | 1.590 |
| meta533 |  | 262.163 | 283.399 | 0.762 | 0.940 | 0.854 | 0.871 | 1.464 | 0.627 | 0.718 | 0.896 | 0.796 | 0.675 | 0.639 | 0.543 | 0.362 | 0.482 | 0.706 |
| meta534 |  | 262.550 | 278.637 | 0.031 | 0.029 | 0.024 | 0.032 | 0.030 | 0.025 | 0.051 | 0.034 | 0.020 | 0.015 | 0.023 | 0.027 | 0.026 | 0.018 | 0.027 |
| meta535 |  | 263.075 | 344.197 | 0.355 | 0.380 | 0.322 | 0.339 | 0.385 | 0.274 | 0.463 | 0.321 | 0.255 | 0.327 | 0.431 | 0.354 | 0.268 | 0.231 | 0.606 |
| meta536 | Primidone | 263.079 | 36.747 | 0.022 | 0.027 | 0.033 | 0.024 | 0.022 | 0.040 | 0.024 | 0.024 | 0.025 | 0.040 | 0.275 | 0.025 | 0.025 | 0.024 | 0.025 |
| meta537 | Phe-Pro | 263.137 | 219.799 | 0.032 | 0.034 | 0.030 | 0.028 | 0.029 | 0.037 | 0.032 | 0.034 | 0.020 | 0.028 | 0.030 | 0.028 | 0.028 | 0.025 | 0.030 |
| meta538 |  | 263.235 | 34.876 | 0.035 | 0.041 | 0.036 | 0.071 | 0.061 | 0.053 | 0.068 | 0.071 | 0.047 | 0.055 | 0.049 | 0.064 | 0.056 | 0.062 | 0.057 |
| meta539 | Isopentenyl pyrophosphate | 264.038 | 435.816 | 0.067 | 0.071 | 0.066 | 0.067 | 0.071 | 0.060 | 0.078 | 0.064 | 0.054 | 0.045 | 0.061 | 0.056 | 0.056 | 0.054 | 0.056 |
| meta540 |  | 264.059 | 391.216 | 0.086 | 0.106 | 0.097 | 0.093 | 0.112 | 0.058 | 0.135 | 0.079 | 0.065 | 0.094 | 0.136 | 0.095 | 0.061 | 0.055 | 0.145 |
| meta541 | Terbutaline | 264.106 | 192.792 | 0.081 | 0.080 | 0.070 | 0.059 | 0.062 | 0.055 | 0.058 | 0.071 | 0.060 | 0.073 | 0.086 | 0.093 | 0.066 | 0.065 | 0.111 |
| meta542 |  | 265.025 | 27.300 | 0.196 | 0.230 | 0.211 | 0.224 | 0.224 | 0.137 | 0.201 | 0.245 | 0.198 | 0.192 | 0.199 | 0.206 | 0.204 | 0.225 | 0.215 |
| meta543 | Gly-Glu | 265.104 | 25.818 | 0.003 | 0.004 | 0.003 | 0.004 | 0.003 | 0.003 | 0.005 | 0.003 | 0.006 | 0.005 | 0.004 | 0.002 | 0.003 | 0.003 | 0.002 |
| meta544 | Thiamine | 265.110 | 312.890 | 1.227 | 1.630 | 1.526 | 1.591 | 1.733 | 1.406 | 1.747 | 1.699 | 1.555 | 1.555 | 1.385 | 1.589 | 1.602 | 1.407 | 1.612 |
| meta545 | Oleic acid | 265.251 | 34.136 | 0.014 | 0.018 | 0.015 | 0.033 | 0.032 | 0.024 | 0.030 | 0.029 | 0.018 | 0.027 | 0.023 | 0.026 | 0.019 | 0.024 | 0.025 |
| meta546 | Molsidomine | 266.104 | 460.415 | 0.050 | 0.038 | 0.054 | 0.052 | 0.054 | 0.061 | 0.049 | 0.050 | 0.052 | 0.050 | 0.053 | 0.053 | 0.048 | 0.042 | 0.051 |
| meta547 | Ser-Pro | 266.113 | 313.005 | 0.194 | 0.253 | 0.230 | 0.249 | 0.269 | 0.214 | 0.263 | 0.255 | 0.229 | 0.213 | 0.217 | 0.241 | 0.248 | 0.213 | 0.255 |
| meta548 | Diethylcarbamazine | 266.121 | 368.281 | 0.231 | 0.196 | 0.234 | 0.196 | 0.236 | 0.167 | 0.214 | 0.246 | 0.173 | 0.156 | 0.327 | 0.262 | 0.269 | 0.144 | 0.277 |
| meta549 |  | 266.171 | 120.597 | 0.059 | 0.056 | 0.049 | 0.117 | 0.116 | 0.121 | 0.093 | 0.073 | 0.119 | 0.110 | 0.041 | 0.041 | 0.116 | 0.134 | 0.231 |
| meta550 | Trihexyphenidyl | 266.232 | 260.138 | 0.152 | 0.108 | 0.170 | 0.029 | 0.063 | 0.121 | 2.175 | 0.011 | 0.883 | 0.052 | 0.065 | 0.035 | 0.867 | 0.350 | 0.056 |
| meta551 |  | 267.087 | 310.770 | 0.444 | 0.419 | 0.393 | 0.323 | 0.382 | 0.332 | 0.414 | 0.497 | 0.499 | 0.528 | 0.473 | 0.403 | 0.397 | 0.358 | 0.390 |
| meta552 | Phe-Thr | 267.132 | 197.951 | 0.024 | 0.027 | 0.025 | 0.034 | 0.036 | 0.045 | 0.024 | 0.015 | 0.007 | 0.014 | 0.018 | 0.024 | 0.024 | 0.025 | 0.023 |
| meta553 |  | 267.135 | 297.196 | 0.039 | 0.042 | 0.035 | 0.042 | 0.024 | 0.042 | 0.035 | 0.050 | 0.046 | 0.030 | 0.039 | 0.040 | 0.038 | 0.026 | 0.047 |
| meta554 | Lys-Cys | 267.143 | 260.679 | 0.007 | 0.009 | 0.008 | 0.004 | 0.006 | 0.003 | 0.028 | 0.005 | 0.005 | 0.003 | 0.006 | 0.008 | 0.010 | 0.004 | 0.007 |
| meta555 |  | 267.287 | 159.313 | 0.049 | 0.054 | 0.045 | 0.042 | 0.074 | 0.032 | 0.053 | 0.056 | 0.048 | 0.050 | 0.046 | 0.043 | 0.046 | 0.031 | 0.056 |
| meta556 |  | 268.050 | 304.625 | 0.464 | 0.317 | 0.372 | 0.192 | 0.270 | 0.273 | 0.296 | 0.463 | 0.401 | 0.553 | 0.341 | 0.287 | 0.454 | 0.228 | 0.311 |
| meta557 | Adenosine | 268.103 | 159.293 | 20.930 | 21.885 | 18.712 | 16.248 | 29.509 | 13.122 | 19.396 | 26.407 | 21.366 | 21.912 | 23.304 | 17.140 | 19.512 | 15.530 | 23.884 |
| meta558 | Pinacidil | 268.149 | 270.694 | 0.015 | 0.014 | 0.012 | 0.014 | 0.018 | 0.010 | 0.023 | 0.020 | 0.010 | 0.011 | 0.010 | 0.013 | 0.011 | 0.010 | 0.011 |
| meta559 | Inosine | 269.087 | 202.082 | 6.293 | 6.321 | 5.943 | 5.659 | 7.827 | 5.288 | 6.992 | 5.173 | 4.353 | 4.848 | 5.893 | 6.233 | 5.454 | 6.319 | 5.606 |
| meta560 | His-Ile | 269.159 | 279.132 | 0.021 | 0.025 | 0.027 | 0.035 | 0.026 | 0.045 | 0.035 | 0.015 | 0.016 | 0.020 | 0.014 | 0.020 | 0.035 | 0.026 | 0.027 |
| meta561 |  | 269.195 | 269.280 | 0.020 | 0.018 | 0.019 | 0.001 | 0.006 | 0.003 | 0.111 | 0.009 | 0.047 | 0.010 | 0.005 | 0.014 | 0.045 | 0.009 | 0.004 |
| meta562 |  | 270.000 | 146.203 | 0.006 | 0.010 | 0.008 | 0.013 | 0.016 | 0.010 | 0.008 | 0.010 | 0.013 | 0.015 | 0.008 | 0.003 | 0.012 | 0.020 | 0.013 |
| meta563 |  | 270.009 | 275.884 | 0.026 | 0.032 | 0.023 | 0.023 | 0.027 | 0.022 | 0.028 | 0.035 | 0.016 | 0.021 | 0.025 | 0.021 | 0.023 | 0.019 | 0.025 |
| meta564 |  | 270.108 | 395.897 | 0.070 | 0.071 | 0.072 | 0.045 | 0.049 | 0.042 | 0.084 | 0.044 | 0.079 | 0.034 | 0.036 | 0.120 | 0.290 | 0.102 | 0.333 |
| meta565 | (R)-mevalonic acid 5-Phosphate | 270.168 | 181.282 | 0.008 | 0.009 | 0.008 | 0.007 | 0.003 | 0.008 | 0.004 | 0.011 | 0.014 | 0.016 | 0.012 | 0.006 | 0.004 | 0.003 | 0.003 |
| meta566 |  | 270.241 | 36.283 | 0.019 | 0.022 | 0.021 | 0.032 | 0.023 | 0.022 | 0.031 | 0.040 | 0.025 | 0.031 | 0.076 | 0.071 | 0.032 | 0.034 | 0.033 |
| meta567 | Pro-Asn | 271.138 | 306.593 | 0.030 | 0.030 | 0.028 | 0.020 | 0.018 | 0.020 | 0.022 | 0.067 | 0.067 | 0.051 | 0.029 | 0.014 | 0.018 | 0.016 | 0.010 |
| meta568 |  | 272.059 | 335.403 | 0.101 | 0.123 | 0.098 | 0.124 | 0.076 | 0.117 | 0.065 | 0.169 | 0.090 | 0.139 | 0.105 | 0.072 | 0.088 | 0.102 | 0.079 |
| meta569 |  | 272.111 | 376.630 | 0.168 | 0.121 | 0.137 | 0.095 | 0.104 | 0.126 | 0.089 | 0.175 | 0.183 | 0.221 | 0.176 | 0.152 | 0.096 | 0.149 | 0.069 |
| meta570 |  | 272.111 | 217.159 | 0.015 | 0.021 | 0.017 | 0.014 | 0.013 | 0.014 | 0.018 | 0.026 | 0.018 | 0.021 | 0.022 | 0.017 | 0.014 | 0.015 | 0.007 |
| meta571 | Metaproterenol | 272.147 | 50.444 | 0.013 | 0.012 | 0.014 | 0.014 | 0.022 | 0.011 | 0.013 | 0.012 | 0.011 | 0.014 | 0.010 | 0.013 | 0.014 | 0.011 | 0.015 |
| meta572 |  | 272.160 | 399.353 | 3.145 | 2.747 | 3.134 | 2.782 | 3.096 | 3.116 | 3.162 | 3.563 | 3.278 | 3.043 | 3.556 | 3.272 | 3.826 | 2.429 | 3.338 |
| meta573 |  | 273.016 | 275.286 | 0.060 | 0.060 | 0.053 | 0.064 | 0.063 | 0.060 | 0.052 | 0.081 | 0.061 | 0.073 | 0.050 | 0.039 | 0.066 | 0.056 | 0.053 |
| meta574 |  | 273.057 | 123.272 | 0.026 | 0.016 | 0.025 | 0.065 | 0.065 | 0.086 | 0.074 | 0.023 | 0.038 | 0.039 | 0.009 | 0.016 | 0.069 | 0.063 | 0.059 |
| meta575 |  | 273.082 | 157.417 | 0.071 | 0.067 | 0.070 | 0.069 | 0.094 | 0.080 | 0.081 | 0.042 | 0.042 | 0.049 | 0.055 | 0.072 | 0.056 | 0.065 | 0.057 |
| meta576 | His-Gly | 273.114 | 376.020 | 0.022 | 0.017 | 0.013 | 0.013 | 0.014 | 0.011 | 0.014 | 0.024 | 0.020 | 0.025 | 0.019 | 0.016 | 0.014 | 0.015 | 0.010 |
| meta577 |  | 273.119 | 252.377 | 0.125 | 0.138 | 0.130 | 0.140 | 0.122 | 0.139 | 0.092 | 0.109 | 0.151 | 0.067 | 0.110 | 0.093 | 0.032 | 0.079 | 0.141 |
| meta578 |  | 273.165 | 318.914 | 0.055 | 0.050 | 0.049 | 0.042 | 0.048 | 0.040 | 0.051 | 0.047 | 0.048 | 0.049 | 0.048 | 0.046 | 0.048 | 0.048 | 0.053 |
| meta579 |  | 273.188 | 28.006 | 0.018 | 0.021 | 0.021 | 0.016 | 0.024 | 0.015 | 0.014 | 0.023 | 0.031 | 0.018 | 0.017 | 0.023 | 0.023 | 0.016 | 0.023 |
| meta580 |  | 274.047 | 292.201 | 0.151 | 0.200 | 0.172 | 0.155 | 0.137 | 0.115 | 0.160 | 0.146 | 0.193 | 0.174 | 0.167 | 0.121 | 0.128 | 0.150 | 0.161 |
| meta581 |  | 274.102 | 408.993 | 0.151 | 0.200 | 0.165 | 0.130 | 0.146 | 0.126 | 0.188 | 0.178 | 0.225 | 0.261 | 0.260 | 0.143 | 0.200 | 0.190 | 0.216 |
| meta582 | Acetyl Tyrosine Ethyl Ester | 274.109 | 276.962 | 0.003 | 0.005 | 0.005 | 0.006 | 0.005 | 0.005 | 0.008 | 0.005 | 0.009 | 0.008 | 0.007 | 0.008 | 0.007 | 0.006 | 0.008 |
| meta583 | Val-Asp | 274.137 | 263.587 | 0.006 | 0.005 | 0.005 | 0.002 | 0.005 | 0.003 | 0.005 | 0.003 | 0.002 | 0.004 | 0.003 | 0.007 | 0.006 | 0.004 | 0.004 |
| meta584 | Val-Arg | 274.185 | 336.248 | 0.068 | 0.102 | 0.098 | 0.125 | 0.067 | 0.101 | 0.124 | 0.081 | 0.088 | 0.071 | 0.082 | 0.081 | 0.125 | 0.088 | 0.112 |
| meta585 |  | 274.200 | 193.051 | 0.057 | 0.063 | 0.062 | 0.077 | 0.126 | 0.034 | 0.116 | 0.069 | 0.036 | 0.021 | 0.036 | 0.024 | 0.031 | 0.020 | 0.054 |
| meta586 | Palmitic acid | 274.273 | 49.692 | 0.689 | 0.600 | 0.642 | 0.959 | 1.025 | 0.729 | 1.254 | 1.123 | 0.961 | 0.717 | 0.728 | 0.654 | 1.390 | 0.888 | 0.819 |
| meta587 |  | 274.908 | 318.454 | 0.138 | 0.129 | 0.155 | 0.124 | 0.137 | 0.148 | 0.145 | 0.125 | 0.157 | 0.129 | 0.144 | 0.122 | 0.168 | 0.186 | 0.130 |
| meta588 |  | 275.031 | 406.180 | 0.023 | 0.028 | 0.029 | 0.025 | 0.026 | 0.018 | 0.031 | 0.026 | 0.035 | 0.028 | 0.024 | 0.020 | 0.030 | 0.026 | 0.031 |
| meta589 | Tacrine | 275.031 | 355.656 | 0.007 | 0.011 | 0.008 | 0.006 | 0.006 | 0.005 | 0.008 | 0.010 | 0.011 | 0.010 | 0.008 | 0.007 | 0.008 | 0.006 | 0.008 |
| meta590 | 1,2,3-Benzenetriol | 275.051 | 393.233 | 0.018 | 0.018 | 0.017 | 0.014 | 0.013 | 0.017 | 0.017 | 0.014 | 0.016 | 0.017 | 0.018 | 0.017 | 0.020 | 0.018 | 0.016 |
| meta591 |  | 275.108 | 248.223 | 0.018 | 0.018 | 0.023 | 0.016 | 0.010 | 0.009 | 0.014 | 0.018 | 0.017 | 0.014 | 0.009 | 0.013 | 0.018 | 0.011 | 0.013 |
| meta592 | Furegrelate | 276.063 | 311.645 | 0.396 | 0.429 | 0.434 | 0.382 | 0.314 | 0.291 | 0.456 | 0.395 | 0.513 | 0.447 | 0.360 | 0.306 | 0.396 | 0.336 | 0.405 |
| meta593 |  | 276.144 | 385.142 | 2.726 | 2.510 | 2.444 | 1.733 | 2.735 | 1.665 | 2.565 | 2.900 | 2.860 | 2.807 | 2.889 | 2.957 | 6.374 | 2.133 | 3.095 |
| meta594 | Lys-Phe | 276.164 | 395.993 | 0.019 | 0.016 | 0.021 | 0.020 | 0.009 | 0.019 | 0.013 | 0.011 | 0.012 | 0.014 | 0.010 | 0.008 | 0.018 | 0.016 | 0.016 |
| meta595 |  | 276.179 | 279.325 | 0.104 | 0.107 | 0.101 | 0.079 | 0.158 | 0.076 | 0.136 | 0.081 | 0.070 | 0.047 | 0.118 | 0.033 | 0.070 | 0.091 | 0.209 |
| meta596 | L-Pipecolic acid | 276.190 | 375.433 | 0.251 | 0.235 | 0.228 | 0.242 | 0.264 | 0.224 | 0.270 | 0.223 | 0.236 | 0.212 | 0.202 | 0.188 | 0.293 | 0.251 | 0.241 |
| meta597 |  | 276.830 | 288.086 | 0.151 | 0.159 | 0.157 | 0.160 | 0.168 | 0.154 | 0.161 | 0.169 | 0.195 | 0.180 | 0.173 | 0.169 | 0.199 | 0.175 | 0.168 |
| meta598 |  | 277.047 | 378.020 | 0.125 | 0.130 | 0.133 | 0.098 | 0.110 | 0.086 | 0.148 | 0.137 | 0.206 | 0.157 | 0.113 | 0.086 | 0.145 | 0.099 | 0.140 |
| meta599 |  | 277.047 | 311.990 | 0.168 | 0.186 | 0.198 | 0.166 | 0.141 | 0.126 | 0.181 | 0.182 | 0.203 | 0.179 | 0.142 | 0.122 | 0.156 | 0.135 | 0.187 |
| meta600 |  | 277.077 | 267.307 | 0.114 | 0.121 | 0.123 | 0.105 | 0.125 | 0.088 | 0.127 | 0.154 | 0.161 | 0.165 | 0.139 | 0.145 | 0.157 | 0.144 | 0.153 |
| meta601 | L-Saccharopine | 277.138 | 435.623 | 0.323 | 0.333 | 0.301 | 0.269 | 0.559 | 0.187 | 0.369 | 0.450 | 0.283 | 0.349 | 0.453 | 0.320 | 0.284 | 0.175 | 0.293 |
| meta602 | Met-Lys | 277.146 | 385.143 | 0.329 | 0.295 | 0.290 | 0.208 | 0.333 | 0.194 | 0.294 | 0.354 | 0.333 | 0.325 | 0.320 | 0.371 | 0.674 | 0.249 | 0.388 |
| meta603 | cis-9-Palmitoleic acid | 277.214 | 44.721 | 0.055 | 0.067 | 0.068 | 0.072 | 0.075 | 0.094 | 0.121 | 0.101 | 0.066 | 0.071 | 0.105 | 0.147 | 0.087 | 0.107 | 0.097 |
| meta604 |  | 278.952 | 287.796 | 0.014 | 0.020 | 0.017 | 0.015 | 0.012 | 0.014 | 0.012 | 0.014 | 0.013 | 0.018 | 0.019 | 0.019 | 0.015 | 0.020 | 0.014 |
| meta605 |  | 279.037 | 279.242 | 0.147 | 0.177 | 0.175 | 0.156 | 0.163 | 0.134 | 0.185 | 0.224 | 0.145 | 0.134 | 0.225 | 0.202 | 0.145 | 0.182 | 0.159 |
| meta606 | Phthalic acid Mono-2-ethylhexyl Ester | 279.158 | 32.857 | 0.581 | 0.638 | 0.631 | 0.570 | 1.108 | 0.856 | 1.234 | 1.232 | 1.101 | 1.072 | 1.060 | 1.212 | 0.828 | 1.016 | 1.199 |
| meta607 | Lys-Gly | 280.052 | 344.182 | 0.005 | 0.005 | 0.004 | 0.005 | 0.006 | 0.003 | 0.006 | 0.004 | 0.003 | 0.004 | 0.006 | 0.005 | 0.004 | 0.004 | 0.009 |
| meta608 |  | 280.091 | 363.012 | 8.464 | 9.066 | 8.009 | 8.117 | 8.272 | 6.587 | 9.844 | 7.994 | 7.505 | 7.817 | 8.314 | 7.854 | 7.310 | 7.170 | 8.429 |
| meta609 | Tyr-Val | 281.148 | 206.782 | 0.007 | 0.010 | 0.008 | 0.010 | 0.007 | 0.007 | 0.010 | 0.017 | 0.018 | 0.017 | 0.005 | 0.003 | 0.007 | 0.003 | 0.005 |
| meta610 | 3-Methoxy-4-Hydroxyphenylglycol Sulfate | 282.063 | 280.867 | 0.054 | 0.050 | 0.044 | 0.008 | 0.099 | 0.076 | 0.083 | 0.093 | 0.114 | 0.092 | 0.017 | 0.017 | 0.109 | 0.029 | 0.026 |
| meta611 | L-Histidinol phosphate | 282.081 | 310.390 | 0.059 | 0.061 | 0.059 | 0.065 | 0.037 | 0.058 | 0.044 | 0.057 | 0.039 | 0.077 | 0.045 | 0.044 | 0.038 | 0.041 | 0.072 |
| meta612 | Ethylmalonic acid | 282.116 | 418.023 | 0.238 | 0.182 | 0.185 | 0.147 | 0.231 | 0.095 | 0.212 | 0.184 | 0.083 | 0.134 | 0.169 | 0.167 | 0.132 | 0.174 | 0.154 |
| meta613 | 1-Methyladenosine | 282.118 | 265.386 | 0.186 | 0.238 | 0.218 | 0.241 | 0.257 | 0.186 | 0.277 | 0.230 | 0.216 | 0.221 | 0.209 | 0.202 | 0.260 | 0.218 | 0.241 |
| meta614 | 3'-O-methyladenosine | 282.118 | 114.934 | 0.017 | 0.015 | 0.017 | 0.022 | 0.057 | 0.030 | 0.034 | 0.084 | 0.045 | 0.059 | 0.030 | 0.010 | 0.041 | 0.029 | 0.046 |
| meta615 |  | 282.278 | 34.042 | 0.203 | 0.272 | 0.227 | 0.680 | 0.599 | 0.486 | 0.558 | 0.512 | 0.229 | 0.345 | 0.369 | 0.442 | 0.354 | 0.553 | 0.501 |
| meta616 | Ser-Thr | 283.012 | 22.392 | 0.003 | 0.003 | 0.003 | 0.003 | 0.002 | 0.002 | 0.002 | 0.005 | 0.002 | 0.004 | 0.003 | 0.003 | 0.004 | 0.003 | 0.002 |
| meta617 |  | 283.082 | 352.062 | 0.644 | 0.805 | 0.702 | 0.596 | 0.677 | 0.463 | 0.629 | 0.701 | 1.052 | 0.842 | 0.719 | 0.823 | 0.656 | 0.753 | 0.828 |
| meta618 | Ethyl glucuronide | 283.108 | 362.091 | 0.403 | 0.372 | 0.323 | 0.346 | 0.359 | 0.254 | 0.436 | 0.358 | 0.251 | 0.360 | 0.415 | 0.347 | 0.261 | 0.201 | 0.432 |
| meta619 | trans-Zeatin | 283.121 | 265.996 | 0.047 | 0.056 | 0.033 | 0.031 | 0.039 | 0.026 | 0.039 | 0.030 | 0.037 | 0.032 | 0.033 | 0.032 | 0.010 | 0.047 | 0.039 |
| meta620 |  | 284.031 | 468.583 | 0.127 | 0.212 | 0.147 | 0.152 | 0.182 | 0.106 | 0.138 | 0.232 | 0.162 | 0.200 | 0.161 | 0.174 | 0.112 | 0.128 | 0.140 |
| meta621 | Guanosine | 284.097 | 242.625 | 0.116 | 0.102 | 0.099 | 0.049 | 0.129 | 0.087 | 0.104 | 0.109 | 0.084 | 0.094 | 0.069 | 0.086 | 0.078 | 0.073 | 0.131 |
| meta622 | Ile-Asn | 284.105 | 362.091 | 1.464 | 1.568 | 1.358 | 1.317 | 1.609 | 0.940 | 1.784 | 1.343 | 1.051 | 1.314 | 1.588 | 1.340 | 1.074 | 0.842 | 1.812 |
| meta623 |  | 284.124 | 393.476 | 0.102 | 0.083 | 0.098 | 0.027 | 0.042 | 0.059 | 0.102 | 0.065 | 0.107 | 0.022 | 0.041 | 0.194 | 0.431 | 0.146 | 0.360 |
| meta624 | Promethazine | 284.132 | 340.093 | 0.008 | 0.009 | 0.007 | 0.007 | 0.008 | 0.005 | 0.011 | 0.009 | 0.008 | 0.008 | 0.006 | 0.006 | 0.007 | 0.008 | 0.007 |
| meta625 |  | 284.329 | 49.023 | 0.021 | 0.028 | 0.026 | 0.027 | 0.027 | 0.030 | 0.037 | 0.034 | 0.027 | 0.020 | 0.024 | 0.022 | 0.024 | 0.028 | 0.027 |
| meta626 | Glycitein | 285.073 | 45.145 | 0.014 | 0.015 | 0.016 | 0.030 | 0.004 | 0.053 | 0.021 | 0.046 | 0.017 | 0.029 | 0.017 | 0.004 | 0.016 | 0.011 | 0.004 |
| meta627 | Xanthosine | 285.081 | 204.677 | 0.012 | 0.015 | 0.019 | 0.018 | 0.027 | 0.019 | 0.020 | 0.024 | 0.018 | 0.011 | 0.021 | 0.018 | 0.015 | 0.016 | 0.014 |
| meta628 | Pro-Phe | 285.127 | 393.735 | 0.016 | 0.011 | 0.014 | 0.005 | 0.024 | 0.009 | 0.013 | 0.018 | 0.012 | 0.018 | 0.005 | 0.026 | 0.050 | 0.017 | 0.043 |
| meta629 | His-Glu | 285.126 | 324.794 | 0.025 | 0.022 | 0.018 | 0.041 | 0.037 | 0.020 | 0.021 | 0.012 | 0.019 | 0.018 | 0.013 | 0.020 | 0.021 | 0.018 | 0.014 |
| meta630 | Gemcitabine | 286.057 | 299.085 | 0.303 | 0.377 | 0.313 | 0.410 | 0.427 | 0.252 | 0.254 | 0.516 | 0.261 | 0.400 | 0.330 | 0.207 | 0.283 | 0.401 | 0.402 |
| meta631 |  | 286.088 | 192.911 | 0.304 | 0.308 | 0.320 | 0.216 | 0.203 | 0.229 | 0.196 | 0.363 | 0.257 | 0.310 | 0.394 | 0.488 | 0.277 | 0.257 | 0.549 |
| meta632 | Tetrahydro-L-biopterin | 286.085 | 467.243 | 0.280 | 0.240 | 0.241 | 0.259 | 0.286 | 0.236 | 0.243 | 0.444 | 0.249 | 0.242 | 0.272 | 0.261 | 0.207 | 0.185 | 0.323 |
| meta633 | Retinol (Vitamin A) | 286.227 | 32.682 | 0.028 | 0.047 | 0.035 | 0.086 | 0.105 | 0.141 | 0.114 | 0.123 | 0.141 | 0.102 | 0.131 | 0.090 | 0.102 | 0.113 | 0.092 |
| meta634 |  | 286.272 | 34.861 | 0.007 | 0.009 | 0.009 | 0.036 | 0.011 | 0.006 | 0.012 | 0.008 | 0.005 | 0.005 | 0.006 | 0.009 | 0.005 | 0.006 | 0.004 |
| meta635 |  | 287.213 | 40.590 | 0.039 | 0.051 | 0.052 | 0.134 | 0.042 | 0.056 | 0.041 | 0.047 | 0.055 | 0.056 | 0.147 | 0.075 | 0.028 | 0.061 | 0.075 |
| meta636 |  | 288.073 | 334.961 | 2.317 | 1.894 | 1.767 | 1.952 | 2.148 | 1.784 | 1.373 | 3.104 | 1.408 | 2.541 | 1.764 | 1.097 | 1.808 | 1.825 | 1.932 |
| meta637 | 5-Fluoro-5'-Deoxyuridine | 288.100 | 304.552 | 0.034 | 0.021 | 0.028 | 0.013 | 0.013 | 0.018 | 0.021 | 0.029 | 0.034 | 0.043 | 0.021 | 0.019 | 0.034 | 0.014 | 0.022 |
| meta638 | Zolmitriptan | 288.165 | 343.215 | 0.008 | 0.008 | 0.008 | 0.010 | 0.007 | 0.011 | 0.008 | 0.008 | 0.005 | 0.007 | 0.010 | 0.012 | 0.009 | 0.007 | 0.008 |
| meta639 |  | 288.179 | 239.609 | 0.027 | 0.025 | 0.021 | 0.011 | 0.031 | 0.012 | 0.015 | 0.028 | 0.023 | 0.016 | 0.021 | 0.031 | 0.052 | 0.031 | 0.038 |
| meta640 | Leu-Arg | 288.201 | 313.963 | 0.022 | 0.021 | 0.021 | 0.028 | 0.016 | 0.022 | 0.025 | 0.016 | 0.014 | 0.012 | 0.015 | 0.016 | 0.020 | 0.017 | 0.021 |
| meta641 |  | 288.215 | 183.941 | 0.041 | 0.047 | 0.040 | 0.033 | 0.081 | 0.012 | 0.030 | 0.078 | 0.042 | 0.025 | 0.031 | 0.031 | 0.028 | 0.014 | 0.056 |
| meta642 | Heptadecanoic acid | 288.288 | 41.316 | 0.578 | 0.707 | 0.666 | 2.748 | 0.710 | 0.394 | 0.859 | 0.366 | 0.346 | 0.350 | 0.489 | 0.918 | 0.381 | 0.414 | 0.314 |
| meta643 |  | 288.982 | 275.127 | 0.083 | 0.098 | 0.076 | 0.075 | 0.083 | 0.064 | 0.079 | 0.093 | 0.082 | 0.099 | 0.077 | 0.059 | 0.078 | 0.074 | 0.081 |
| meta644 |  | 289.139 | 287.796 | 0.056 | 0.059 | 0.055 | 0.061 | 0.059 | 0.046 | 0.052 | 0.060 | 0.064 | 0.058 | 0.068 | 0.055 | 0.059 | 0.057 | 0.061 |
| meta645 | Tebuthiuron | 289.138 | 370.440 | 0.053 | 0.050 | 0.066 | 0.072 | 0.050 | 0.048 | 0.050 | 0.040 | 0.061 | 0.076 | 0.054 | 0.051 | 0.053 | 0.034 | 0.069 |
| meta646 | Prometon | 289.174 | 353.005 | 0.020 | 0.020 | 0.016 | 0.014 | 0.019 | 0.013 | 0.019 | 0.034 | 0.038 | 0.034 | 0.027 | 0.016 | 0.019 | 0.003 | 0.013 |
| meta647 | N-Tris[hydroxymethyl]methyl-2-aminoethanesulfonic acid [TES] | 290.089 | 341.903 | 0.059 | 0.075 | 0.057 | 0.065 | 0.069 | 0.046 | 0.052 | 0.085 | 0.074 | 0.080 | 0.056 | 0.052 | 0.055 | 0.042 | 0.071 |
| meta648 | Ala-His | 290.122 | 215.081 | 0.008 | 0.009 | 0.008 | 0.010 | 0.014 | 0.009 | 0.036 | 0.011 | 0.011 | 0.010 | 0.009 | 0.004 | 0.009 | 0.009 | 0.005 |
| meta649 | Metyrapone | 290.128 | 27.218 | 0.009 | 0.012 | 0.010 | 0.011 | 0.010 | 0.008 | 0.010 | 0.012 | 0.010 | 0.008 | 0.010 | 0.010 | 0.010 | 0.008 | 0.010 |
| meta650 |  | 290.133 | 385.128 | 0.479 | 0.447 | 0.421 | 0.437 | 0.669 | 0.368 | 0.579 | 0.579 | 0.308 | 0.456 | 0.390 | 0.611 | 0.422 | 0.232 | 0.322 |
| meta651 | Sotalol | 290.159 | 380.310 | 3.654 | 3.433 | 3.607 | 1.795 | 4.240 | 2.083 | 2.361 | 5.339 | 5.006 | 2.707 | 3.733 | 6.166 | 14.241 | 4.852 | 5.427 |
| meta652 |  | 290.194 | 235.569 | 0.015 | 0.014 | 0.016 | 0.012 | 0.026 | 0.014 | 0.033 | 0.014 | 0.007 | 0.007 | 0.012 | 0.008 | 0.010 | 0.007 | 0.012 |
| meta653 |  | 290.267 | 50.251 | 0.029 | 0.019 | 0.025 | 0.024 | 0.017 | 0.019 | 0.019 | 0.024 | 0.023 | 0.026 | 0.028 | 0.022 | 0.024 | 0.021 | 0.019 |
| meta654 |  | 290.882 | 287.563 | 0.033 | 0.025 | 0.031 | 0.036 | 0.041 | 0.038 | 0.037 | 0.038 | 0.054 | 0.041 | 0.038 | 0.041 | 0.066 | 0.044 | 0.038 |
| meta655 |  | 291.069 | 202.206 | 4.812 | 5.432 | 5.048 | 4.632 | 5.724 | 3.969 | 5.661 | 4.932 | 4.285 | 4.316 | 4.876 | 5.000 | 4.621 | 4.909 | 4.865 |
| meta656 |  | 291.103 | 337.528 | 0.403 | 0.357 | 0.348 | 0.075 | 0.178 | 0.086 | 0.967 | 0.255 | 0.340 | 0.313 | 0.434 | 0.722 | 0.728 | 0.342 | 0.423 |
| meta657 | Bestatin | 291.168 | 292.894 | 0.007 | 0.005 | 0.006 | 0.003 | 0.002 | 0.002 | 0.008 | 0.002 | 0.004 | 0.002 | 0.002 | 0.012 | 0.016 | 0.006 | 0.041 |
| meta658 |  | 292.137 | 119.899 | 0.105 | 0.092 | 0.083 | 0.126 | 0.141 | 0.091 | 0.069 | 0.114 | 0.142 | 0.171 | 0.139 | 0.082 | 0.168 | 0.118 | 0.286 |
| meta659 |  | 292.137 | 78.559 | 0.125 | 0.141 | 0.127 | 0.141 | 0.164 | 0.100 | 0.115 | 0.178 | 0.081 | 0.106 | 0.113 | 0.118 | 0.133 | 0.096 | 0.210 |
| meta660 |  | 292.137 | 275.555 | 0.116 | 0.166 | 0.130 | 0.128 | 0.177 | 0.105 | 0.193 | 0.183 | 0.103 | 0.114 | 0.176 | 0.169 | 0.110 | 0.103 | 0.157 |
| meta661 | (-)-Medicarpin | 293.073 | 202.802 | 0.075 | 0.090 | 0.075 | 0.078 | 0.092 | 0.068 | 0.095 | 0.080 | 0.068 | 0.068 | 0.077 | 0.078 | 0.072 | 0.084 | 0.090 |
| meta662 | 5'-Deoxyadenosine | 293.140 | 79.063 | 0.016 | 0.023 | 0.021 | 0.023 | 0.025 | 0.026 | 0.023 | 0.027 | 0.030 | 0.017 | 0.017 | 0.020 | 0.023 | 0.017 | 0.030 |
| meta663 | (4Z,7Z,10Z,13Z,16Z,19Z)-4,7,10,13,1 6,19-Docosahexaenoic acid | 293.219 | 27.300 | 0.014 | 0.017 | 0.015 | 0.018 | 0.019 | 0.011 | 0.016 | 0.020 | 0.016 | 0.014 | 0.016 | 0.017 | 0.015 | 0.015 | 0.017 |
| meta664 | His-Pro | 294.153 | 73.202 | 0.069 | 0.078 | 0.080 | 0.113 | 0.091 | 0.094 | 0.094 | 0.077 | 0.029 | 0.056 | 0.052 | 0.002 | 0.060 | 0.065 | 0.073 |
| meta665 |  | 294.169 | 206.691 | 0.031 | 0.034 | 0.032 | 0.042 | 0.030 | 0.036 | 0.043 | 0.070 | 0.042 | 0.038 | 0.023 | 0.023 | 0.019 | 0.019 | 0.037 |
| meta666 | Stearidonic Acid | 294.241 | 37.302 | 0.037 | 0.036 | 0.038 | 0.061 | 0.062 | 0.068 | 0.075 | 0.072 | 0.041 | 0.058 | 0.069 | 0.055 | 0.067 | 0.081 | 0.061 |
| meta667 |  | 295.064 | 157.390 | 0.258 | 0.249 | 0.231 | 0.269 | 0.323 | 0.242 | 0.289 | 0.213 | 0.206 | 0.210 | 0.246 | 0.261 | 0.261 | 0.262 | 0.244 |
| meta668 | Ile-Cys | 295.127 | 338.626 | 0.188 | 0.176 | 0.159 | 0.149 | 0.193 | 0.129 | 0.166 | 0.199 | 0.157 | 0.200 | 0.192 | 0.173 | 0.193 | 0.185 | 0.193 |
| meta669 | Ile-Tyr | 295.163 | 191.442 | 0.015 | 0.015 | 0.018 | 0.022 | 0.012 | 0.019 | 0.033 | 0.017 | 0.010 | 0.012 | 0.008 | 0.009 | 0.020 | 0.012 | 0.016 |
| meta670 |  | 295.225 | 46.145 | 0.051 | 0.064 | 0.063 | 0.066 | 0.065 | 0.086 | 0.100 | 0.106 | 0.074 | 0.075 | 0.100 | 0.138 | 0.068 | 0.108 | 0.094 |
| meta671 |  | 296.064 | 362.810 | 0.887 | 0.978 | 0.876 | 0.907 | 1.059 | 0.754 | 1.031 | 0.895 | 0.901 | 0.862 | 0.970 | 0.870 | 0.921 | 0.775 | 0.929 |
| meta672 | Tyr-Asp | 296.097 | 105.238 | 0.029 | 0.054 | 0.043 | 0.061 | 0.083 | 0.052 | 0.070 | 0.043 | 0.123 | 0.068 | 0.034 | 0.027 | 0.067 | 0.159 | 0.087 |
| meta673 |  | 296.124 | 381.405 | 0.200 | 0.180 | 0.187 | 0.044 | 0.166 | 0.111 | 0.199 | 0.107 | 0.227 | 0.033 | 0.069 | 0.436 | 0.963 | 0.361 | 0.712 |
| meta674 | all cis-(6,9,12)-Linolenic acid | 296.256 | 36.142 | 0.072 | 0.084 | 0.089 | 0.169 | 0.149 | 0.152 | 0.174 | 0.167 | 0.096 | 0.128 | 0.137 | 0.117 | 0.133 | 0.174 | 0.148 |
| meta675 |  | 296.916 | 318.454 | 0.045 | 0.042 | 0.052 | 0.039 | 0.042 | 0.056 | 0.047 | 0.040 | 0.054 | 0.042 | 0.044 | 0.037 | 0.054 | 0.065 | 0.043 |
| meta676 | S-Methyl-5'-thioadenosine | 298.096 | 96.394 | 10.115 | 16.792 | 12.766 | 14.901 | 13.216 | 14.085 | 13.992 | 16.019 | 20.158 | 17.294 | 15.547 | 20.690 | 18.592 | 15.567 | 40.507 |
| meta677 | Primaquine | 298.138 | 293.943 | 0.005 | 0.007 | 0.004 | 0.005 | 0.003 | 0.003 | 0.016 | 0.012 | 0.013 | 0.007 | 0.006 | 0.003 | 0.005 | 0.009 | 0.014 |
| meta678 |  | 298.141 | 387.019 | 1.703 | 1.491 | 1.503 | 0.467 | 0.597 | 1.028 | 1.337 | 1.154 | 1.312 | 0.291 | 0.483 | 4.822 | 5.460 | 3.320 | 5.062 |
| meta679 | Linoleic acid | 298.272 | 36.142 | 0.055 | 0.070 | 0.070 | 0.122 | 0.122 | 0.116 | 0.133 | 0.133 | 0.089 | 0.124 | 0.138 | 0.112 | 0.118 | 0.134 | 0.128 |
| meta680 |  | 299.056 | 352.062 | 0.151 | 0.182 | 0.151 | 0.142 | 0.167 | 0.111 | 0.143 | 0.167 | 0.261 | 0.203 | 0.164 | 0.194 | 0.171 | 0.175 | 0.184 |
| meta681 | gamma-L-Glutamyl-L-glutamic acid | 299.085 | 473.466 | 0.015 | 0.010 | 0.011 | 0.011 | 0.006 | 0.015 | 0.012 | 0.008 | 0.017 | 0.017 | 0.019 | 0.018 | 0.021 | 0.010 | 0.013 |
| meta682 | N-Acetyl-L-tyrosine | 300.011 | 224.548 | 0.005 | 0.004 | 0.005 | 0.004 | 0.005 | 0.007 | 0.005 | 0.006 | 0.007 | 0.006 | 0.005 | 0.004 | 0.005 | 0.005 | 0.004 |
| meta683 |  | 300.073 | 279.377 | 0.192 | 0.227 | 0.197 | 0.031 | 0.483 | 0.016 | 0.387 | 0.019 | 0.271 | 0.206 | 0.014 | 0.031 | 0.260 | 0.104 | 0.057 |
| meta684 |  | 300.119 | 292.801 | 0.015 | 0.028 | 0.025 | 0.012 | 0.020 | 0.011 | 0.018 | 0.014 | 0.029 | 0.011 | 0.017 | 0.016 | 0.026 | 0.014 | 0.020 |
| meta685 | Palmitoyl ethanolamide | 300.288 | 35.418 | 0.045 | 0.047 | 0.056 | 0.059 | 0.083 | 0.071 | 0.165 | 0.126 | 0.054 | 0.071 | 0.073 | 0.095 | 0.037 | 0.056 | 0.069 |
| meta686 |  | 301.023 | 221.133 | 0.024 | 0.024 | 0.028 | 0.030 | 0.024 | 0.023 | 0.030 | 0.029 | 0.029 | 0.029 | 0.025 | 0.023 | 0.029 | 0.021 | 0.028 |
| meta687 | D-Xylose | 301.117 | 51.444 | 0.069 | 0.048 | 0.046 | 0.044 | 0.047 | 0.048 | 0.053 | 0.058 | 0.066 | 0.066 | 0.075 | 0.051 | 0.032 | 0.036 | 0.030 |
| meta688 | 4-Oxoretinol | 301.214 | 33.431 | 0.071 | 0.105 | 0.112 | 0.141 | 0.158 | 0.228 | 0.209 | 0.209 | 0.179 | 0.119 | 0.278 | 0.285 | 0.137 | 0.203 | 0.184 |
| meta689 | Methylthiouracil | 302.079 | 377.716 | 0.042 | 0.037 | 0.041 | 0.016 | 0.052 | 0.018 | 0.122 | 0.029 | 0.072 | 0.034 | 0.016 | 0.019 | 0.057 | 0.030 | 0.020 |
| meta690 | D-Mannose 1-phosphate | 302.069 | 278.716 | 0.024 | 0.035 | 0.033 | 0.021 | 0.041 | 0.019 | 0.039 | 0.022 | 0.039 | 0.029 | 0.023 | 0.027 | 0.033 | 0.030 | 0.028 |
| meta691 |  | 302.085 | 348.528 | 0.110 | 0.108 | 0.109 | 0.077 | 0.074 | 0.075 | 0.064 | 0.164 | 0.261 | 0.216 | 0.132 | 0.075 | 0.076 | 0.080 | 0.051 |
| meta692 | Nordihydroguaiaretic Acid | 302.158 | 355.512 | 0.043 | 0.043 | 0.037 | 0.023 | 0.043 | 0.018 | 0.033 | 0.041 | 0.034 | 0.039 | 0.045 | 0.023 | 0.081 | 0.023 | 0.048 |
| meta693 |  | 302.194 | 230.020 | 0.060 | 0.062 | 0.063 | 0.052 | 0.098 | 0.061 | 0.081 | 0.084 | 0.041 | 0.051 | 0.041 | 0.030 | 0.066 | 0.052 | 0.059 |
| meta694 |  | 302.213 | 163.824 | 0.006 | 0.006 | 0.006 | 0.010 | 0.009 | 0.006 | 0.012 | 0.011 | 0.006 | 0.004 | 0.004 | 0.006 | 0.005 | 0.005 | 0.004 |
| meta695 | Stearic acid | 302.304 | 118.004 | 0.007 | 0.008 | 0.006 | 0.003 | 0.007 | 0.006 | 0.013 | 0.006 | 0.003 | 0.004 | 0.006 | 0.006 | 0.006 | 0.004 | 0.005 |
| meta696 | Flumequine | 303.110 | 235.569 | 0.215 | 0.253 | 0.225 | 0.183 | 0.246 | 0.158 | 0.231 | 0.292 | 0.344 | 0.307 | 0.259 | 0.266 | 0.244 | 0.245 | 0.271 |
| meta697 |  | 303.153 | 327.272 | 0.009 | 0.016 | 0.010 | 0.012 | 0.008 | 0.009 | 0.007 | 0.006 | 0.014 | 0.008 | 0.013 | 0.011 | 0.011 | 0.014 | 0.005 |
| meta698 | Lys-Asp | 303.161 | 373.456 | 0.006 | 0.006 | 0.007 | 0.006 | 0.007 | 0.006 | 0.007 | 0.009 | 0.009 | 0.007 | 0.006 | 0.010 | 0.018 | 0.007 | 0.008 |
| meta699 |  | 303.190 | 262.363 | 0.007 | 0.009 | 0.006 | 0.005 | 0.013 | 0.004 | 0.015 | 0.011 | 0.003 | 0.006 | 0.006 | 0.012 | 0.005 | 0.003 | 0.014 |
| meta700 | Eicosapentaenoic acid | 303.230 | 38.178 | 0.090 | 0.114 | 0.110 | 0.157 | 0.160 | 0.252 | 0.151 | 0.154 | 0.214 | 0.154 | 0.198 | 0.166 | 0.131 | 0.207 | 0.110 |
| meta701 | Arg-Glu | 304.160 | 437.739 | 0.123 | 0.134 | 0.139 | 0.147 | 0.097 | 0.140 | 0.140 | 0.077 | 0.100 | 0.103 | 0.105 | 0.108 | 0.112 | 0.094 | 0.099 |
| meta702 | Ile-Met | 304.174 | 365.806 | 1.139 | 1.149 | 1.000 | 1.014 | 1.411 | 0.820 | 1.116 | 1.543 | 0.921 | 1.077 | 1.157 | 0.666 | 1.515 | 0.817 | 1.097 |
| meta703 | Lys-Pro | 304.185 | 446.224 | 0.074 | 0.101 | 0.106 | 0.093 | 0.104 | 0.092 | 0.114 | 0.123 | 0.138 | 0.066 | 0.073 | 0.093 | 0.121 | 0.098 | 0.112 |
| meta704 |  | 304.210 | 221.125 | 0.024 | 0.027 | 0.023 | 0.012 | 0.039 | 0.018 | 0.028 | 0.035 | 0.019 | 0.023 | 0.018 | 0.017 | 0.023 | 0.024 | 0.034 |
| meta705 |  | 304.298 | 90.265 | 0.071 | 0.066 | 0.065 | 0.065 | 0.053 | 0.047 | 0.050 | 0.069 | 0.059 | 0.073 | 0.071 | 0.069 | 0.060 | 0.050 | 0.053 |
| meta706 |  | 304.324 | 27.742 | 0.008 | 0.011 | 0.013 | 0.013 | 0.011 | 0.009 | 0.011 | 0.013 | 0.011 | 0.010 | 0.010 | 0.011 | 0.011 | 0.013 | 0.012 |
| meta707 | 2'-O-methylinosine | 305.084 | 138.605 | 0.020 | 0.022 | 0.015 | 0.032 | 0.045 | 0.029 | 0.054 | 0.012 | 0.034 | 0.024 | 0.022 | 0.012 | 0.025 | 0.021 | 0.040 |
| meta708 |  | 305.151 | 152.754 | 0.019 | 0.021 | 0.020 | 0.024 | 0.018 | 0.017 | 0.012 | 0.012 | 0.009 | 0.019 | 0.020 | 0.016 | 0.016 | 0.014 | 0.020 |
| meta709 | AG-17 | 305.155 | 74.062 | 0.050 | 0.046 | 0.058 | 0.042 | 0.027 | 0.028 | 0.460 | 0.030 | 0.037 | 0.021 | 0.017 | 0.024 | 0.040 | 0.032 | 0.030 |
| meta710 |  | 306.074 | 406.049 | 0.549 | 0.566 | 0.555 | 0.402 | 0.451 | 0.395 | 0.617 | 0.608 | 0.727 | 0.785 | 0.544 | 0.423 | 0.620 | 0.376 | 0.616 |
| meta711 | Nname,Clomipramine | 306.086 | 362.069 | 0.013 | 0.018 | 0.020 | 0.023 | 0.014 | 0.013 | 0.012 | 0.020 | 0.017 | 0.024 | 0.019 | 0.017 | 0.018 | 0.014 | 0.022 |
| meta712 |  | 306.154 | 395.349 | 0.757 | 0.727 | 0.704 | 0.590 | 1.097 | 0.480 | 0.610 | 0.680 | 0.821 | 0.512 | 0.785 | 0.800 | 2.361 | 0.622 | 0.960 |
| meta713 |  | 307.025 | 278.637 | 0.038 | 0.050 | 0.048 | 0.040 | 0.041 | 0.033 | 0.047 | 0.042 | 0.057 | 0.045 | 0.041 | 0.049 | 0.048 | 0.046 | 0.048 |
| meta714 |  | 307.042 | 202.172 | 1.975 | 2.140 | 1.982 | 1.880 | 2.197 | 1.617 | 2.246 | 1.979 | 1.658 | 1.772 | 1.967 | 1.977 | 1.852 | 2.061 | 1.897 |
| meta715 | Tyr-Ser | 307.068 | 236.415 | 0.040 | 0.040 | 0.051 | 0.033 | 0.029 | 0.027 | 0.043 | 0.035 | 0.055 | 0.045 | 0.033 | 0.030 | 0.037 | 0.034 | 0.037 |
| meta716 | Val-Leu | 307.077 | 337.963 | 0.054 | 0.037 | 0.040 | 0.024 | 0.018 | 0.008 | 0.108 | 0.030 | 0.038 | 0.034 | 0.048 | 0.088 | 0.088 | 0.038 | 0.047 |
| meta717 |  | 307.112 | 366.391 | 1.481 | 1.698 | 1.462 | 1.039 | 1.895 | 1.305 | 1.010 | 2.196 | 1.493 | 1.324 | 0.930 | 1.397 | 1.715 | 1.355 | 1.626 |
| meta718 | 3-Nonen-1-ol, (Z)- | 307.261 | 35.133 | 0.012 | 0.014 | 0.014 | 0.025 | 0.020 | 0.020 | 0.029 | 0.036 | 0.045 | 0.039 | 0.017 | 0.016 | 0.010 | 0.008 | 0.013 |
| meta719 | Glutathione | 308.089 | 388.453 | 0.301 | 0.329 | 0.360 | 0.224 | 0.348 | 0.465 | 0.536 | 0.364 | 0.556 | 0.410 | 0.430 | 0.427 | 0.486 | 0.422 | 0.548 |
| meta720 |  | 308.168 | 108.038 | 0.091 | 0.134 | 0.118 | 0.190 | 0.190 | 0.129 | 0.109 | 0.126 | 0.152 | 0.169 | 0.143 | 0.090 | 0.183 | 0.144 | 0.250 |
| meta721 |  | 308.168 | 51.780 | 0.143 | 0.137 | 0.129 | 0.227 | 0.245 | 0.142 | 0.174 | 0.136 | 0.054 | 0.101 | 0.105 | 0.152 | 0.119 | 0.090 | 0.183 |
| meta722 |  | 309.073 | 233.558 | 0.194 | 0.222 | 0.194 | 0.012 | 0.032 | 0.010 | 0.295 | 0.598 | 0.246 | 0.191 | 0.081 | 0.136 | 0.176 | 0.019 | 0.072 |
| meta723 |  | 309.241 | 110.010 | 0.236 | 0.269 | 0.182 | 0.667 | 0.710 | 0.342 | 0.702 | 0.300 | 0.420 | 0.151 | 0.096 | 0.142 | 0.033 | 0.059 | 0.179 |
| meta724 |  | 310.055 | 334.963 | 0.636 | 0.649 | 0.628 | 0.688 | 0.702 | 0.549 | 0.439 | 0.976 | 0.460 | 0.768 | 0.567 | 0.347 | 0.611 | 0.554 | 0.683 |
| meta725 | DL-Homocystine | 310.091 | 390.439 | 0.045 | 0.041 | 0.028 | 0.033 | 0.037 | 0.031 | 0.042 | 0.039 | 0.032 | 0.041 | 0.025 | 0.032 | 0.039 | 0.043 | 0.060 |
| meta726 | Ile-Asp | 310.130 | 417.409 | 1.117 | 0.971 | 0.938 | 0.995 | 1.139 | 0.941 | 1.237 | 0.906 | 0.802 | 1.001 | 0.802 | 0.981 | 2.361 | 0.852 | 1.024 |
| meta727 |  | 310.199 | 182.673 | 0.086 | 0.083 | 0.086 | 0.076 | 0.121 | 0.051 | 0.093 | 0.109 | 0.107 | 0.096 | 0.085 | 0.084 | 0.091 | 0.048 | 0.108 |
| meta728 | (3-Carboxypropyl)trimethylammonium cation | 310.272 | 40.261 | 0.004 | 0.003 | 0.004 | 0.061 | 0.007 | 0.004 | 0.008 | 0.059 | 0.009 | 0.007 | 0.007 | 0.006 | 0.003 | 0.006 | 0.005 |
| meta729 |  | 310.308 | 33.352 | 0.025 | 0.040 | 0.032 | 0.058 | 0.049 | 0.021 | 0.063 | 0.050 | 0.096 | 0.047 | 0.055 | 0.078 | 0.054 | 0.058 | 0.204 |
| meta730 | Glu-Pro | 311.058 | 335.556 | 0.062 | 0.065 | 0.059 | 0.063 | 0.081 | 0.044 | 0.043 | 0.093 | 0.043 | 0.078 | 0.055 | 0.039 | 0.060 | 0.048 | 0.075 |
| meta731 |  | 311.159 | 298.044 | 0.249 | 0.259 | 0.229 | 0.164 | 0.211 | 0.147 | 0.210 | 0.598 | 0.507 | 0.452 | 0.295 | 0.092 | 0.166 | 0.092 | 0.060 |
| meta732 | 2'-Deoxyguanosine 5'-monophosphate (dGMP) | 312.052 | 335.573 | 0.033 | 0.033 | 0.025 | 0.025 | 0.035 | 0.016 | 0.014 | 0.049 | 0.022 | 0.035 | 0.024 | 0.015 | 0.025 | 0.026 | 0.016 |
| meta733 |  | 312.105 | 377.212 | 0.278 | 0.195 | 0.205 | 0.115 | 0.122 | 0.088 | 0.103 | 0.249 | 0.184 | 0.305 | 0.259 | 0.111 | 0.133 | 0.165 | 0.081 |
| meta734 |  | 312.155 | 377.830 | 0.072 | 0.099 | 0.103 | 0.049 | 0.052 | 0.056 | 0.091 | 0.067 | 0.060 | 0.035 | 0.044 | 0.167 | 0.151 | 0.135 | 0.136 |
| meta735 |  | 312.215 | 178.547 | 0.007 | 0.012 | 0.010 | 0.005 | 0.017 | 0.005 | 0.017 | 0.014 | 0.012 | 0.010 | 0.006 | 0.006 | 0.011 | 0.005 | 0.008 |
| meta736 |  | 312.360 | 49.369 | 0.019 | 0.025 | 0.024 | 0.023 | 0.023 | 0.018 | 0.027 | 0.023 | 0.024 | 0.025 | 0.021 | 0.023 | 0.022 | 0.025 | 0.025 |
| meta737 |  | 313.062 | 438.697 | 0.022 | 0.018 | 0.015 | 0.015 | 0.022 | 0.018 | 0.017 | 0.014 | 0.020 | 0.019 | 0.019 | 0.017 | 0.019 | 0.015 | 0.022 |
| meta738 | Lys-Lys | 313.164 | 298.225 | 0.006 | 0.008 | 0.009 | 0.003 | 0.004 | 0.005 | 0.007 | 0.017 | 0.012 | 0.013 | 0.008 | 0.005 | 0.004 | 0.004 | 0.004 |
| meta739 | 1-Palmitoylglycerol | 313.272 | 33.548 | 0.064 | 0.051 | 0.047 | 0.018 | 0.017 | 0.035 | 0.040 | 0.086 | 0.018 | 0.025 | 0.042 | 0.048 | 0.029 | 0.011 | 0.016 |
| meta740 |  | 314.135 | 398.759 | 0.810 | 0.659 | 0.891 | 0.221 | 0.302 | 0.559 | 0.777 | 0.548 | 0.786 | 0.168 | 0.285 | 1.867 | 3.634 | 1.419 | 2.741 |
| meta741 |  | 314.144 | 202.172 | 0.160 | 0.167 | 0.151 | 0.146 | 0.180 | 0.129 | 0.163 | 0.129 | 0.122 | 0.138 | 0.153 | 0.163 | 0.145 | 0.149 | 0.148 |
| meta742 |  | 314.231 | 175.429 | 0.068 | 0.075 | 0.068 | 0.050 | 0.155 | 0.038 | 0.137 | 0.076 | 0.077 | 0.043 | 0.029 | 0.021 | 0.041 | 0.024 | 0.040 |
| meta743 |  | 314.786 | 287.956 | 0.423 | 0.412 | 0.435 | 0.463 | 0.476 | 0.405 | 0.469 | 0.466 | 0.565 | 0.494 | 0.484 | 0.451 | 0.553 | 0.480 | 0.471 |
| meta744 |  | 315.132 | 191.831 | 0.088 | 0.077 | 0.072 | 0.060 | 0.009 | 0.012 | 0.068 | 0.012 | 0.056 | 0.008 | 0.074 | 0.074 | 0.076 | 0.012 | 0.080 |
| meta745 |  | 315.193 | 105.178 | 0.027 | 0.057 | 0.043 | 0.052 | 0.049 | 0.087 | 0.069 | 0.163 | 0.082 | 0.073 | 0.058 | 0.079 | 0.021 | 0.162 | 0.100 |
| meta746 |  | 315.194 | 46.391 | 0.060 | 0.053 | 0.050 | 0.060 | 0.058 | 0.054 | 0.062 | 0.068 | 0.064 | 0.054 | 0.063 | 0.054 | 0.028 | 0.066 | 0.051 |
| meta747 |  | 316.174 | 352.822 | 0.077 | 0.075 | 0.067 | 0.033 | 0.074 | 0.036 | 0.041 | 0.128 | 0.103 | 0.089 | 0.092 | 0.084 | 0.216 | 0.075 | 0.099 |
| meta748 |  | 316.210 | 213.321 | 0.088 | 0.096 | 0.089 | 0.079 | 0.124 | 0.068 | 0.095 | 0.133 | 0.118 | 0.086 | 0.083 | 0.049 | 0.054 | 0.080 | 0.079 |
| meta749 | Decanoyl-L-carnitine | 316.246 | 171.524 | 0.039 | 0.043 | 0.037 | 0.021 | 0.068 | 0.022 | 0.053 | 0.056 | 0.041 | 0.042 | 0.045 | 0.029 | 0.037 | 0.028 | 0.039 |
| meta750 |  | 316.919 | 318.447 | 0.031 | 0.027 | 0.032 | 0.026 | 0.028 | 0.031 | 0.029 | 0.025 | 0.034 | 0.027 | 0.028 | 0.029 | 0.036 | 0.037 | 0.031 |
| meta751 | Pantetheine | 317.093 | 379.469 | 0.022 | 0.019 | 0.019 | 0.014 | 0.037 | 0.028 | 0.035 | 0.027 | 0.018 | 0.017 | 0.042 | 0.016 | 0.020 | 0.016 | 0.022 |
| meta752 |  | 317.210 | 47.634 | 0.315 | 0.457 | 0.429 | 0.466 | 0.522 | 0.429 | 0.777 | 0.608 | 0.621 | 0.415 | 0.453 | 0.436 | 0.255 | 0.482 | 0.448 |
| meta753 | 10-Hydroxycarbazepine | 318.116 | 287.076 | 0.057 | 0.045 | 0.039 | 0.044 | 0.048 | 0.031 | 0.039 | 0.043 | 0.030 | 0.040 | 0.059 | 0.043 | 0.037 | 0.039 | 0.041 |
| meta754 |  | 318.189 | 357.386 | 0.064 | 0.070 | 0.066 | 0.041 | 0.077 | 0.043 | 0.043 | 0.132 | 0.070 | 0.100 | 0.088 | 0.073 | 0.127 | 0.062 | 0.051 |
| meta755 |  | 318.189 | 278.885 | 0.020 | 0.025 | 0.023 | 0.016 | 0.018 | 0.014 | 0.017 | 0.028 | 0.053 | 0.027 | 0.062 | 0.013 | 0.010 | 0.015 | 0.030 |
| meta756 |  | 318.201 | 434.933 | 0.015 | 0.017 | 0.017 | 0.016 | 0.016 | 0.016 | 0.018 | 0.012 | 0.013 | 0.010 | 0.013 | 0.016 | 0.017 | 0.015 | 0.011 |
| meta757 |  | 318.200 | 471.062 | 0.034 | 0.042 | 0.038 | 0.039 | 0.043 | 0.032 | 0.038 | 0.035 | 0.044 | 0.035 | 0.034 | 0.042 | 0.044 | 0.037 | 0.040 |
| meta758 |  | 318.298 | 49.431 | 0.050 | 0.055 | 0.055 | 0.132 | 0.109 | 0.035 | 0.181 | 0.047 | 0.028 | 0.024 | 0.020 | 0.020 | 0.038 | 0.030 | 0.028 |
| meta759 | His-Tyr | 319.148 | 407.452 | 0.100 | 0.101 | 0.083 | 0.087 | 0.091 | 0.072 | 0.105 | 0.165 | 0.072 | 0.083 | 0.081 | 0.125 | 0.072 | 0.051 | 0.101 |
| meta760 | Pargyline | 319.224 | 34.602 | 0.032 | 0.037 | 0.041 | 0.069 | 0.073 | 0.092 | 0.094 | 0.090 | 0.109 | 0.067 | 0.112 | 0.110 | 0.065 | 0.065 | 0.050 |
| meta761 |  | 319.240 | 184.212 | 0.024 | 0.017 | 0.025 | 0.004 | 0.007 | 0.029 | 0.045 | 0.027 | 0.009 | 0.054 | 0.025 | 0.009 | 0.035 | 0.036 | 0.014 |
| meta762 |  | 320.053 | 392.556 | 0.028 | 0.032 | 0.030 | 0.032 | 0.030 | 0.019 | 0.035 | 0.032 | 0.028 | 0.035 | 0.038 | 0.028 | 0.023 | 0.022 | 0.037 |
| meta763 |  | 320.071 | 344.007 | 0.041 | 0.043 | 0.038 | 0.042 | 0.059 | 0.041 | 0.046 | 0.070 | 0.061 | 0.059 | 0.055 | 0.054 | 0.047 | 0.053 | 0.058 |
| meta764 |  | 320.090 | 380.052 | 0.817 | 0.622 | 0.700 | 0.211 | 1.042 | 0.204 | 3.218 | 0.237 | 1.495 | 0.571 | 0.215 | 0.247 | 1.158 | 0.398 | 0.279 |
| meta765 |  | 320.095 | 181.795 | 0.013 | 0.009 | 0.009 | 0.020 | 0.022 | 0.006 | 0.011 | 0.011 | 0.014 | 0.007 | 0.011 | 0.014 | 0.010 | 0.005 | 0.015 |
| meta766 |  | 320.121 | 387.019 | 0.071 | 0.064 | 0.060 | 0.021 | 0.036 | 0.049 | 0.054 | 0.068 | 0.052 | 0.014 | 0.022 | 0.164 | 0.188 | 0.137 | 0.192 |
| meta767 | Guanine | 320.125 | 50.416 | 0.012 | 0.013 | 0.012 | 0.014 | 0.012 | 0.009 | 0.015 | 0.010 | 0.009 | 0.011 | 0.015 | 0.013 | 0.012 | 0.011 | 0.017 |
| meta768 |  | 320.168 | 389.649 | 0.037 | 0.033 | 0.038 | 0.028 | 0.045 | 0.022 | 0.032 | 0.041 | 0.037 | 0.031 | 0.041 | 0.049 | 0.101 | 0.031 | 0.047 |
| meta769 |  | 321.076 | 225.719 | 0.017 | 0.020 | 0.016 | 0.022 | 0.024 | 0.018 | 0.033 | 0.020 | 0.010 | 0.010 | 0.016 | 0.014 | 0.013 | 0.017 | 0.011 |
| meta770 |  | 321.288 | 238.872 | 0.004 | 0.004 | 0.005 | 0.005 | 0.006 | 0.005 | 0.007 | 0.004 | 0.006 | 0.005 | 0.004 | 0.004 | 0.006 | 0.005 | 0.005 |
| meta771 |  | 321.313 | 33.421 | 0.013 | 0.020 | 0.018 | 0.029 | 0.017 | 0.008 | 0.029 | 0.018 | 0.023 | 0.011 | 0.019 | 0.018 | 0.015 | 0.028 | 0.049 |
| meta772 |  | 322.069 | 434.117 | 2.286 | 2.806 | 2.644 | 2.035 | 2.040 | 1.921 | 2.656 | 2.676 | 3.482 | 3.244 | 2.353 | 2.028 | 2.869 | 2.010 | 2.879 |
| meta773 |  | 322.105 | 374.196 | 0.158 | 0.142 | 0.138 | 0.116 | 0.143 | 0.120 | 0.158 | 0.166 | 0.161 | 0.185 | 0.166 | 0.105 | 0.131 | 0.108 | 0.159 |
| meta774 | Peucedanin | 322.111 | 336.165 | 0.028 | 0.029 | 0.030 | 0.043 | 0.039 | 0.033 | 0.031 | 0.032 | 0.002 | 0.016 | 0.033 | 0.032 | 0.022 | 0.031 | 0.040 |
| meta775 |  | 323.037 | 293.935 | 0.208 | 0.219 | 0.192 | 0.223 | 0.245 | 0.185 | 0.295 | 0.170 | 0.066 | 0.085 | 0.119 | 0.152 | 0.210 | 0.106 | 0.190 |
| meta776 |  | 323.079 | 279.314 | 0.022 | 0.023 | 0.029 | 0.021 | 0.020 | 0.018 | 0.025 | 0.020 | 0.024 | 0.018 | 0.021 | 0.029 | 0.020 | 0.026 | 0.025 |
| meta777 |  | 323.084 | 25.880 | 0.044 | 0.058 | 0.067 | 0.068 | 0.042 | 0.061 | 0.064 | 0.099 | 0.132 | 0.103 | 0.037 | 0.036 | 0.034 | 0.042 | 0.041 |
| meta778 |  | 323.256 | 34.363 | 0.030 | 0.048 | 0.032 | 0.077 | 0.057 | 0.068 | 0.098 | 0.088 | 0.090 | 0.069 | 0.036 | 0.028 | 0.016 | 0.012 | 0.024 |
| meta779 |  | 323.256 | 119.578 | 0.021 | 0.018 | 0.013 | 0.032 | 0.029 | 0.032 | 0.042 | 0.041 | 0.069 | 0.043 | 0.015 | 0.006 | 0.007 | 0.007 | 0.008 |
| meta780 | Cytidine 5'-monophosphate (CMP) | 324.057 | 446.468 | 0.070 | 0.063 | 0.063 | 0.051 | 0.003 | 0.003 | 0.067 | 0.066 | 0.072 | 0.077 | 0.075 | 0.075 | 0.003 | 0.051 | 0.002 |
| meta781 | Cytidine 5'-monophosphate | 324.063 | 433.605 | 0.094 | 0.140 | 0.138 | 0.111 | 0.096 | 0.096 | 0.153 | 0.136 | 0.167 | 0.161 | 0.132 | 0.113 | 0.170 | 0.089 | 0.141 |
| meta782 | Linoleoyl ethanolamide | 324.288 | 35.164 | 0.014 | 0.015 | 0.018 | 0.024 | 0.029 | 0.021 | 0.036 | 0.034 | 0.034 | 0.030 | 0.035 | 0.039 | 0.024 | 0.036 | 0.041 |
| meta783 |  | 325.015 | 152.481 | 0.016 | 0.015 | 0.013 | 0.016 | 0.019 | 0.012 | 0.018 | 0.013 | 0.014 | 0.012 | 0.012 | 0.014 | 0.013 | 0.013 | 0.015 |
| meta784 | Uridine 5'-monophosphate (UMP) | 325.041 | 429.577 | 0.373 | 0.346 | 0.364 | 0.299 | 0.332 | 0.279 | 0.399 | 0.495 | 0.414 | 0.322 | 0.343 | 0.355 | 0.415 | 0.313 | 0.438 |
| meta785 | Met-His | 325.069 | 434.842 | 0.014 | 0.014 | 0.015 | 0.009 | 0.012 | 0.009 | 0.008 | 0.009 | 0.021 | 0.017 | 0.009 | 0.012 | 0.014 | 0.014 | 0.014 |
| meta786 |  | 326.028 | 334.961 | 0.172 | 0.156 | 0.138 | 0.158 | 0.190 | 0.134 | 0.092 | 0.296 | 0.104 | 0.218 | 0.138 | 0.073 | 0.143 | 0.138 | 0.172 |
| meta787 | N-Oleoylethanolamine | 326.304 | 34.861 | 0.014 | 0.019 | 0.021 | 0.030 | 0.038 | 0.027 | 0.069 | 0.057 | 0.031 | 0.030 | 0.030 | 0.047 | 0.023 | 0.039 | 0.041 |
| meta788 |  | 326.376 | 82.085 | 0.053 | 0.060 | 0.059 | 0.050 | 0.054 | 0.048 | 0.066 | 0.059 | 0.058 | 0.056 | 0.069 | 0.051 | 0.055 | 0.052 | 0.051 |
| meta789 |  | 327.034 | 27.218 | 0.039 | 0.052 | 0.048 | 0.053 | 0.050 | 0.031 | 0.047 | 0.062 | 0.050 | 0.045 | 0.048 | 0.050 | 0.047 | 0.053 | 0.050 |
| meta790 |  | 327.066 | 152.693 | 0.026 | 0.027 | 0.024 | 0.020 | 0.030 | 0.016 | 0.025 | 0.018 | 0.016 | 0.024 | 0.017 | 0.018 | 0.014 | 0.016 | 0.019 |
| meta791 |  | 327.104 | 27.830 | 0.012 | 0.017 | 0.014 | 0.014 | 0.016 | 0.009 | 0.014 | 0.025 | 0.015 | 0.013 | 0.014 | 0.015 | 0.014 | 0.018 | 0.015 |
| meta792 |  | 327.139 | 202.147 | 0.593 | 0.559 | 0.486 | 0.496 | 0.678 | 0.466 | 0.601 | 0.430 | 0.338 | 0.410 | 0.513 | 0.559 | 0.463 | 0.597 | 0.484 |
| meta793 |  | 327.133 | 152.754 | 0.030 | 0.032 | 0.017 | 0.026 | 0.037 | 0.016 | 0.030 | 0.027 | 0.010 | 0.019 | 0.033 | 0.019 | 0.016 | 0.013 | 0.034 |
| meta794 |  | 327.199 | 75.848 | 0.202 | 0.228 | 0.157 | 0.227 | 0.139 | 0.175 | 0.176 | 0.183 | 0.178 | 0.159 | 0.135 | 0.198 | 0.215 | 0.183 | 0.142 |
| meta795 |  | 327.251 | 109.425 | 0.035 | 0.064 | 0.036 | 0.075 | 0.135 | 0.070 | 0.141 | 0.110 | 0.094 | 0.028 | 0.025 | 0.023 | 0.015 | 0.022 | 0.037 |
| meta796 | zeleplon | 328.112 | 298.823 | 0.036 | 0.050 | 0.034 | 0.061 | 0.103 | 0.067 | 0.149 | 0.102 | 0.056 | 0.024 | 0.097 | 0.018 | 0.052 | 0.029 | 0.077 |
| meta797 | Phe-Tyr | 328.141 | 202.172 | 0.107 | 0.101 | 0.090 | 0.094 | 0.119 | 0.090 | 0.112 | 0.077 | 0.070 | 0.082 | 0.103 | 0.098 | 0.087 | 0.103 | 0.087 |
| meta798 |  | 328.150 | 391.968 | 0.179 | 0.145 | 0.169 | 0.043 | 0.064 | 0.096 | 0.137 | 0.106 | 0.151 | 0.034 | 0.060 | 0.425 | 0.601 | 0.269 | 0.520 |
| meta799 |  | 329.029 | 290.136 | 0.171 | 0.227 | 0.192 | 0.168 | 0.150 | 0.127 | 0.189 | 0.170 | 0.218 | 0.194 | 0.173 | 0.139 | 0.150 | 0.154 | 0.197 |
| meta800 | Oxprenolol | 329.191 | 431.552 | 0.011 | 0.014 | 0.017 | 0.019 | 0.013 | 0.013 | 0.012 | 0.015 | 0.014 | 0.010 | 0.012 | 0.014 | 0.013 | 0.012 | 0.010 |
| meta801 | Adenosine 2',3'-cyclic monophosphate | 330.058 | 268.917 | 0.015 | 0.014 | 0.013 | 0.013 | 0.014 | 0.012 | 0.014 | 0.012 | 0.010 | 0.014 | 0.013 | 0.013 | 0.013 | 0.015 | 0.016 |
| meta802 |  | 330.128 | 343.112 | 0.729 | 0.733 | 0.679 | 0.295 | 1.776 | 0.158 | 2.684 | 0.184 | 0.784 | 0.408 | 0.197 | 0.276 | 1.090 | 0.364 | 0.320 |
| meta803 |  | 330.226 | 221.651 | 0.023 | 0.024 | 0.022 | 0.016 | 0.029 | 0.017 | 0.030 | 0.037 | 0.026 | 0.019 | 0.029 | 0.012 | 0.015 | 0.007 | 0.027 |
| meta804 | 9-Decen-1-ol | 330.334 | 47.962 | 0.034 | 0.040 | 0.030 | 0.032 | 0.077 | 0.041 | 0.095 | 0.035 | 0.017 | 0.015 | 0.031 | 0.044 | 0.018 | 0.051 | 0.020 |
| meta805 |  | 330.816 | 287.465 | 0.046 | 0.043 | 0.041 | 0.054 | 0.059 | 0.047 | 0.051 | 0.054 | 0.065 | 0.059 | 0.051 | 0.055 | 0.076 | 0.055 | 0.050 |
| meta806 | Deoxyguanosine | 331.109 | 403.928 | 0.450 | 0.288 | 0.391 | 0.398 | 0.461 | 0.355 | 0.437 | 0.549 | 0.256 | 0.405 | 0.448 | 0.370 | 0.180 | 0.302 | 0.442 |
| meta807 |  | 332.029 | 311.494 | 0.055 | 0.066 | 0.067 | 0.058 | 0.056 | 0.047 | 0.072 | 0.070 | 0.080 | 0.069 | 0.048 | 0.043 | 0.068 | 0.057 | 0.066 |
| meta808 |  | 332.051 | 406.265 | 0.054 | 0.067 | 0.060 | 0.052 | 0.049 | 0.042 | 0.061 | 0.059 | 0.076 | 0.065 | 0.058 | 0.044 | 0.057 | 0.042 | 0.066 |
| meta809 | SANGUINARINE | 332.096 | 343.853 | 0.011 | 0.014 | 0.011 | 0.010 | 0.016 | 0.012 | 0.011 | 0.019 | 0.015 | 0.014 | 0.015 | 0.015 | 0.018 | 0.010 | 0.011 |
| meta810 |  | 332.143 | 238.918 | 0.051 | 0.042 | 0.046 | 0.039 | 0.049 | 0.033 | 0.023 | 0.058 | 0.041 | 0.056 | 0.054 | 0.056 | 0.042 | 0.040 | 0.060 |
| meta811 |  | 332.154 | 397.480 | 0.034 | 0.030 | 0.036 | 0.031 | 0.040 | 0.034 | 0.036 | 0.024 | 0.022 | 0.041 | 0.026 | 0.033 | 0.074 | 0.043 | 0.021 |
| meta812 |  | 332.169 | 378.221 | 0.300 | 0.292 | 0.273 | 0.196 | 0.315 | 0.182 | 0.263 | 0.351 | 0.322 | 0.263 | 0.368 | 0.483 | 0.954 | 0.272 | 0.373 |
| meta813 |  | 332.205 | 343.293 | 0.006 | 0.009 | 0.008 | 0.008 | 0.010 | 0.008 | 0.010 | 0.013 | 0.006 | 0.009 | 0.007 | 0.007 | 0.008 | 0.006 | 0.007 |
| meta814 |  | 332.241 | 202.365 | 0.038 | 0.049 | 0.043 | 0.029 | 0.062 | 0.027 | 0.045 | 0.074 | 0.040 | 0.043 | 0.049 | 0.043 | 0.036 | 0.041 | 0.039 |
| meta815 |  | 332.329 | 87.466 | 0.023 | 0.023 | 0.025 | 0.035 | 0.020 | 0.026 | 0.024 | 0.024 | 0.022 | 0.026 | 0.040 | 0.023 | 0.024 | 0.023 | 0.022 |
| meta816 |  | 332.516 | 276.140 | 0.014 | 0.012 | 0.017 | 0.016 | 0.014 | 0.016 | 0.017 | 0.017 | 0.014 | 0.009 | 0.013 | 0.010 | 0.014 | 0.010 | 0.016 |
| meta817 |  | 332.892 | 287.768 | 0.017 | 0.013 | 0.016 | 0.019 | 0.024 | 0.021 | 0.018 | 0.019 | 0.029 | 0.025 | 0.021 | 0.022 | 0.039 | 0.024 | 0.018 |
| meta818 | Desipramine | 333.140 | 420.722 | 5.803 | 5.848 | 5.502 | 4.446 | 5.330 | 3.625 | 4.699 | 7.987 | 6.042 | 8.805 | 4.504 | 6.878 | 4.159 | 3.865 | 4.160 |
| meta819 |  | 334.140 | 50.854 | 0.027 | 0.032 | 0.025 | 0.032 | 0.031 | 0.024 | 0.026 | 0.024 | 0.031 | 0.026 | 0.030 | 0.029 | 0.037 | 0.025 | 0.039 |
| meta820 | Lomefloxacin | 334.142 | 420.722 | 0.724 | 0.712 | 0.682 | 0.516 | 0.630 | 0.452 | 0.588 | 1.006 | 0.749 | 1.045 | 0.516 | 0.862 | 0.514 | 0.513 | 0.548 |
| meta821 |  | 334.185 | 375.729 | 0.959 | 0.985 | 0.942 | 0.620 | 2.093 | 0.581 | 0.747 | 1.981 | 1.030 | 0.670 | 0.756 | 1.382 | 2.570 | 1.466 | 1.209 |
| meta822 |  | 335.020 | 32.322 | 0.017 | 0.020 | 0.019 | 0.026 | 0.003 | 0.030 | 0.009 | 0.043 | 0.035 | 0.032 | 0.030 | 0.004 | 0.002 | 0.003 | 0.003 |
| meta823 | Nicotinamide ribotide | 335.072 | 422.033 | 0.090 | 0.084 | 0.066 | 0.067 | 0.083 | 0.066 | 0.112 | 0.080 | 0.058 | 0.066 | 0.066 | 0.079 | 0.082 | 0.058 | 0.073 |
| meta824 |  | 335.179 | 359.680 | 0.033 | 0.034 | 0.031 | 0.030 | 0.112 | 0.019 | 0.003 | 0.007 | 0.014 | 0.008 | 0.011 | 0.011 | 0.037 | 0.022 | 0.023 |
| meta825 | N-Acetyl-D-galactosamine | 336.085 | 480.249 | 0.019 | 0.019 | 0.015 | 0.018 | 0.024 | 0.015 | 0.026 | 0.026 | 0.018 | 0.021 | 0.027 | 0.025 | 0.020 | 0.016 | 0.024 |
| meta826 | Ile-Lys | 336.116 | 398.826 | 0.039 | 0.035 | 0.052 | 0.017 | 0.017 | 0.031 | 0.039 | 0.025 | 0.036 | 0.012 | 0.020 | 0.082 | 0.157 | 0.072 | 0.110 |
| meta827 | Chlorpromazine | 336.129 | 296.828 | 0.016 | 0.020 | 0.015 | 0.016 | 0.021 | 0.014 | 0.018 | 0.016 | 0.018 | 0.014 | 0.016 | 0.016 | 0.015 | 0.015 | 0.017 |
| meta828 | Isopentenyladenosine | 336.163 | 49.549 | 0.011 | 0.018 | 0.009 | 0.015 | 0.017 | 0.010 | 0.019 | 0.018 | 0.014 | 0.005 | 0.015 | 0.017 | 0.009 | 0.012 | 0.016 |
| meta829 |  | 336.324 | 34.702 | 0.061 | 0.084 | 0.077 | 0.170 | 0.169 | 0.089 | 0.199 | 0.176 | 0.185 | 0.126 | 0.152 | 0.161 | 0.123 | 0.176 | 0.325 |
| meta830 |  | 336.338 | 26.981 | 0.007 | 0.009 | 0.008 | 0.016 | 0.011 | 0.016 | 0.013 | 0.034 | 0.012 | 0.012 | 0.009 | 0.010 | 0.013 | 0.018 | 0.017 |
| meta831 |  | 337.065 | 27.753 | 0.025 | 0.031 | 0.031 | 0.101 | 0.020 | 0.059 | 0.037 | 0.054 | 0.064 | 0.031 | 0.044 | 0.024 | 0.008 | 0.009 | 0.009 |
| meta832 | S-Nitroso-L-glutathione | 337.088 | 362.520 | 0.068 | 0.086 | 0.090 | 0.069 | 0.092 | 0.067 | 0.103 | 0.078 | 0.087 | 0.073 | 0.080 | 0.071 | 0.072 | 0.071 | 0.087 |
| meta833 |  | 337.250 | 184.553 | 0.052 | 0.051 | 0.050 | 0.009 | 0.016 | 0.073 | 0.099 | 0.048 | 0.013 | 0.087 | 0.055 | 0.018 | 0.064 | 0.075 | 0.024 |
| meta834 |  | 337.250 | 163.123 | 0.017 | 0.017 | 0.015 | 0.003 | 0.005 | 0.018 | 0.023 | 0.027 | 0.007 | 0.033 | 0.014 | 0.013 | 0.022 | 0.026 | 0.013 |
| meta835 | (2E,6E)-Farnesol | 337.249 | 27.658 | 0.004 | 0.004 | 0.003 | 0.005 | 0.004 | 0.034 | 0.004 | 0.091 | 0.003 | 0.005 | 0.004 | 0.004 | 0.045 | 0.003 | 0.004 |
| meta836 | MG(18:2(9Z,12Z)/0:0/0:0)[rac] | 337.272 | 42.030 | 0.205 | 0.244 | 0.294 | 0.355 | 0.325 | 0.232 | 0.427 | 0.296 | 0.274 | 0.278 | 0.387 | 0.460 | 0.393 | 0.348 | 0.330 |
| meta837 |  | 338.022 | 279.992 | 0.014 | 0.011 | 0.010 | 0.002 | 0.037 | 0.001 | 0.041 | 0.002 | 0.009 | 0.011 | 0.002 | 0.003 | 0.011 | 0.005 | 0.003 |
| meta838 |  | 338.074 | 467.897 | 0.052 | 0.063 | 0.053 | 0.065 | 0.067 | 0.043 | 0.077 | 0.042 | 0.035 | 0.054 | 0.067 | 0.051 | 0.055 | 0.037 | 0.099 |
| meta839 |  | 338.111 | 474.966 | 0.022 | 0.023 | 0.023 | 0.015 | 0.021 | 0.014 | 0.029 | 0.024 | 0.036 | 0.040 | 0.024 | 0.019 | 0.029 | 0.017 | 0.027 |
| meta840 | Erucamide | 338.341 | 32.950 | 0.627 | 0.876 | 0.703 | 1.218 | 0.833 | 0.342 | 1.228 | 0.884 | 0.977 | 0.529 | 0.971 | 1.123 | 0.541 | 1.134 | 2.569 |
| meta841 |  | 338.830 | 287.076 | 0.042 | 0.035 | 0.031 | 0.036 | 0.046 | 0.034 | 0.035 | 0.040 | 0.040 | 0.042 | 0.041 | 0.049 | 0.049 | 0.038 | 0.042 |
| meta842 |  | 339.058 | 279.697 | 0.041 | 0.051 | 0.057 | 0.052 | 0.041 | 0.039 | 0.050 | 0.048 | 0.041 | 0.032 | 0.054 | 0.056 | 0.041 | 0.045 | 0.049 |
| meta843 | 5'-Phosphoribosyl-5-amino-4-imidazolecarboxamide (AICAR) | 339.068 | 382.249 | 0.011 | 0.012 | 0.013 | 0.013 | 0.014 | 0.009 | 0.006 | 0.008 | 0.011 | 0.007 | 0.004 | 0.012 | 0.011 | 0.008 | 0.009 |
| meta844 |  | 339.095 | 434.172 | 0.364 | 0.459 | 0.443 | 0.333 | 0.350 | 0.314 | 0.430 | 0.445 | 0.556 | 0.514 | 0.396 | 0.334 | 0.486 | 0.338 | 0.481 |
| meta845 | 1-methylguanosine | 339.136 | 146.064 | 0.013 | 0.010 | 0.012 | 0.013 | 0.009 | 0.011 | 0.012 | 0.009 | 0.009 | 0.012 | 0.013 | 0.008 | 0.013 | 0.008 | 0.019 |
| meta846 |  | 339.288 | 132.191 | 0.013 | 0.013 | 0.017 | 0.050 | 0.053 | 0.050 | 0.083 | 0.025 | 0.021 | 0.042 | 0.025 | 0.022 | 0.024 | 0.035 | 0.052 |
| meta847 |  | 340.026 | 466.644 | 0.289 | 0.289 | 0.299 | 0.289 | 0.327 | 0.294 | 0.244 | 0.280 | 0.242 | 0.231 | 0.273 | 0.255 | 0.267 | 0.295 | 0.369 |
| meta848 |  | 340.033 | 341.477 | 0.017 | 0.018 | 0.020 | 0.023 | 0.022 | 0.016 | 0.021 | 0.016 | 0.016 | 0.018 | 0.020 | 0.017 | 0.017 | 0.014 | 0.019 |
| meta849 |  | 340.196 | 392.448 | 0.024 | 0.027 | 0.026 | 0.025 | 0.027 | 0.019 | 0.028 | 0.021 | 0.023 | 0.022 | 0.021 | 0.024 | 0.029 | 0.022 | 0.023 |
| meta850 |  | 340.285 | 41.391 | 0.081 | 0.108 | 0.108 | 0.067 | 0.095 | 0.107 | 0.128 | 0.128 | 0.097 | 0.121 | 0.171 | 0.151 | 0.130 | 0.147 | 0.173 |
| meta851 | Pristanic acid | 340.319 | 107.387 | 0.009 | 0.008 | 0.009 | 0.023 | 0.021 | 0.024 | 0.024 | 0.023 | 0.030 | 0.020 | 0.026 | 0.021 | 0.025 | 0.036 | 0.060 |
| meta852 |  | 340.880 | 318.447 | 0.073 | 0.073 | 0.084 | 0.069 | 0.072 | 0.088 | 0.076 | 0.066 | 0.092 | 0.070 | 0.075 | 0.065 | 0.087 | 0.097 | 0.070 |
| meta853 | 1-Stearoyl-rac-glycerol | 341.303 | 228.438 | 0.026 | 0.020 | 0.021 | 0.018 | 0.020 | 0.015 | 0.035 | 0.031 | 0.011 | 0.014 | 0.029 | 0.020 | 0.015 | 0.016 | 0.020 |
| meta854 |  | 342.071 | 380.100 | 0.319 | 0.271 | 0.302 | 0.090 | 0.436 | 0.084 | 1.247 | 0.091 | 0.577 | 0.224 | 0.090 | 0.102 | 0.482 | 0.171 | 0.111 |
| meta855 |  | 342.137 | 399.459 | 1.058 | 0.943 | 1.055 | 1.056 | 1.028 | 0.946 | 0.969 | 0.957 | 0.515 | 0.670 | 0.819 | 1.069 | 0.711 | 0.808 | 0.827 |
| meta856 | Pro-Tyr | 342.137 | 428.095 | 0.051 | 0.046 | 0.044 | 0.055 | 0.056 | 0.085 | 0.063 | 0.052 | 0.034 | 0.036 | 0.041 | 0.051 | 0.044 | 0.068 | 0.073 |
| meta857 | 5-Nitro-2-Phenylpropylaminobenzoic Acid [Nppb] | 342.153 | 109.663 | 0.033 | 0.062 | 0.042 | 0.079 | 0.085 | 0.063 | 0.051 | 0.063 | 0.057 | 0.067 | 0.051 | 0.042 | 0.063 | 0.062 | 0.109 |
| meta858 |  | 342.166 | 382.799 | 0.106 | 0.090 | 0.103 | 0.058 | 0.046 | 0.091 | 0.057 | 0.091 | 0.096 | 0.021 | 0.056 | 0.364 | 0.480 | 0.235 | 0.268 |
| meta859 |  | 342.262 | 165.713 | 0.016 | 0.020 | 0.019 | 0.015 | 0.037 | 0.012 | 0.034 | 0.046 | 0.021 | 0.027 | 0.014 | 0.022 | 0.019 | 0.012 | 0.031 |
| meta860 |  | 343.043 | 152.578 | 0.039 | 0.045 | 0.049 | 0.041 | 0.049 | 0.036 | 0.053 | 0.040 | 0.038 | 0.031 | 0.035 | 0.040 | 0.038 | 0.039 | 0.039 |
| meta861 | Metronidazole | 343.141 | 399.459 | 0.151 | 0.134 | 0.143 | 0.176 | 0.156 | 0.136 | 0.166 | 0.144 | 0.078 | 0.098 | 0.120 | 0.161 | 0.116 | 0.110 | 0.111 |
| meta862 | 20-Hydroxyarachidonic acid | 343.224 | 45.144 | 0.017 | 0.027 | 0.022 | 0.019 | 0.031 | 0.027 | 0.045 | 0.041 | 0.025 | 0.015 | 0.051 | 0.046 | 0.023 | 0.038 | 0.031 |
| meta863 |  | 343.294 | 152.255 | 0.071 | 0.080 | 0.070 | 0.093 | 0.096 | 0.067 | 0.072 | 0.066 | 0.072 | 0.066 | 0.052 | 0.055 | 0.060 | 0.055 | 0.079 |
| meta864 | Sertraline | 344.031 | 277.687 | 0.082 | 0.098 | 0.103 | 0.096 | 0.109 | 0.077 | 0.129 | 0.105 | 0.098 | 0.076 | 0.083 | 0.087 | 0.101 | 0.082 | 0.096 |
| meta865 |  | 344.088 | 275.213 | 0.110 | 0.111 | 0.106 | 0.085 | 0.103 | 0.111 | 0.108 | 0.141 | 0.101 | 0.125 | 0.116 | 0.140 | 0.112 | 0.095 | 0.101 |
| meta866 | (-)-Usnic acid | 344.089 | 353.815 | 0.073 | 0.072 | 0.059 | 0.061 | 0.069 | 0.065 | 0.065 | 0.058 | 0.059 | 0.065 | 0.061 | 0.069 | 0.065 | 0.061 | 0.067 |
| meta867 | Enterodiol | 344.180 | 351.747 | 0.007 | 0.008 | 0.009 | 0.007 | 0.008 | 0.009 | 0.008 | 0.004 | 0.024 | 0.022 | 0.008 | 0.009 | 0.023 | 0.006 | 0.025 |
| meta868 |  | 344.204 | 322.987 | 0.017 | 0.019 | 0.023 | 0.015 | 0.029 | 0.015 | 0.024 | 0.027 | 0.015 | 0.015 | 0.017 | 0.011 | 0.012 | 0.009 | 0.016 |
| meta869 |  | 344.226 | 76.026 | 0.436 | 0.539 | 0.378 | 0.542 | 0.328 | 0.399 | 0.402 | 0.458 | 0.428 | 0.374 | 0.315 | 0.456 | 0.507 | 0.428 | 0.338 |
| meta870 |  | 345.070 | 431.907 | 3.808 | 4.254 | 4.050 | 3.885 | 3.703 | 2.953 | 3.739 | 4.027 | 4.146 | 3.554 | 4.268 | 3.801 | 4.240 | 4.328 | 4.637 |
| meta871 |  | 345.068 | 25.818 | 0.017 | 0.019 | 0.021 | 0.020 | 0.014 | 0.016 | 0.023 | 0.034 | 0.030 | 0.028 | 0.026 | 0.010 | 0.009 | 0.013 | 0.012 |
| meta872 | Tyr-Pro | 345.076 | 478.790 | 0.457 | 0.470 | 0.492 | 0.409 | 0.479 | 0.436 | 0.462 | 0.446 | 0.477 | 0.447 | 0.432 | 0.541 | 0.585 | 0.520 | 0.477 |
| meta873 |  | 345.945 | 322.554 | 0.012 | 0.016 | 0.016 | 0.011 | 0.013 | 0.013 | 0.015 | 0.015 | 0.017 | 0.020 | 0.012 | 0.013 | 0.017 | 0.013 | 0.014 |
| meta874 |  | 346.022 | 61.164 | 0.270 | 0.257 | 0.389 | 0.216 | 0.278 | 0.203 | 0.225 | 0.246 | 0.250 | 0.219 | 0.350 | 0.236 | 0.263 | 0.259 | 0.258 |
| meta875 |  | 346.017 | 152.740 | 0.017 | 0.020 | 0.021 | 0.028 | 0.021 | 0.025 | 0.022 | 0.016 | 0.016 | 0.014 | 0.015 | 0.015 | 0.019 | 0.014 | 0.023 |
| meta876 |  | 346.159 | 376.539 | 0.015 | 0.012 | 0.010 | 0.011 | 0.013 | 0.009 | 0.009 | 0.013 | 0.012 | 0.018 | 0.013 | 0.010 | 0.015 | 0.014 | 0.014 |
| meta877 | Dihydrocapsaicin | 346.170 | 392.448 | 0.012 | 0.009 | 0.015 | 0.012 | 0.015 | 0.011 | 0.017 | 0.012 | 0.015 | 0.009 | 0.008 | 0.007 | 0.014 | 0.015 | 0.013 |
| meta878 |  | 347.100 | 388.913 | 0.230 | 0.206 | 0.218 | 0.144 | 0.113 | 0.144 | 0.154 | 0.210 | 0.267 | 0.303 | 0.239 | 0.229 | 0.246 | 0.175 | 0.196 |
| meta879 | Corticosterone | 347.220 | 40.981 | 0.172 | 0.227 | 0.204 | 0.218 | 0.508 | 0.192 | 0.084 | 0.545 | 0.041 | 0.189 | 0.223 | 0.168 | 0.175 | 0.207 | 0.145 |
| meta880 | Adenosine monophosphate (AMP) | 348.070 | 417.421 | 26.022 | 26.560 | 27.561 | 23.458 | 24.910 | 22.184 | 26.928 | 29.291 | 29.573 | 26.366 | 28.135 | 25.484 | 31.448 | 25.373 | 32.104 |
| meta881 | Adenosine 3'-monophosphate | 348.069 | 398.186 | 0.124 | 0.090 | 0.099 | 0.105 | 0.132 | 0.109 | 0.117 | 0.133 | 0.115 | 0.112 | 0.135 | 0.123 | 0.114 | 0.110 | 0.109 |
| meta882 | Tyr-Gln | 348.103 | 388.945 | 0.033 | 0.030 | 0.034 | 0.025 | 0.041 | 0.023 | 0.021 | 0.027 | 0.051 | 0.038 | 0.034 | 0.036 | 0.041 | 0.047 | 0.025 |
| meta883 | Tyr-Lys | 348.138 | 384.430 | 0.046 | 0.047 | 0.038 | 0.040 | 0.043 | 0.032 | 0.054 | 0.058 | 0.031 | 0.048 | 0.037 | 0.038 | 0.029 | 0.032 | 0.033 |
| meta884 |  | 348.168 | 295.161 | 0.022 | 0.020 | 0.020 | 0.022 | 0.019 | 0.024 | 0.013 | 0.016 | 0.009 | 0.020 | 0.026 | 0.027 | 0.015 | 0.019 | 0.017 |
| meta885 |  | 348.211 | 339.453 | 0.033 | 0.028 | 0.029 | 0.023 | 0.048 | 0.021 | 0.095 | 0.019 | 0.054 | 0.017 | 0.017 | 0.018 | 0.057 | 0.032 | 0.016 |
| meta886 |  | 348.236 | 264.816 | 0.087 | 0.096 | 0.088 | 0.054 | 0.141 | 0.041 | 0.114 | 0.142 | 0.016 | 0.043 | 0.050 | 0.055 | 0.058 | 0.022 | 0.267 |
| meta887 | Inosine 5'-monophosphate (IMP) | 349.053 | 435.388 | 0.093 | 0.140 | 0.123 | 0.106 | 0.120 | 0.110 | 0.101 | 0.143 | 0.144 | 0.147 | 0.116 | 0.136 | 0.142 | 0.135 | 0.081 |
| meta888 |  | 349.071 | 278.885 | 0.037 | 0.056 | 0.054 | 0.043 | 0.040 | 0.032 | 0.041 | 0.041 | 0.073 | 0.046 | 0.059 | 0.045 | 0.051 | 0.055 | 0.052 |
| meta889 | N-Acetylaspartylglutamate (NAAG) | 349.071 | 441.858 | 0.137 | 0.206 | 0.217 | 0.172 | 0.185 | 0.101 | 0.158 | 0.669 | 0.238 | 0.148 | 0.138 | 0.210 | 0.259 | 0.193 | 0.597 |
| meta890 |  | 349.116 | 371.464 | 0.423 | 0.451 | 0.519 | 0.322 | 0.426 | 0.360 | 0.428 | 0.395 | 0.650 | 0.539 | 0.403 | 0.454 | 0.602 | 0.447 | 0.397 |
| meta891 |  | 349.156 | 307.586 | 0.010 | 0.006 | 0.008 | 0.008 | 0.005 | 0.010 | 0.004 | 0.008 | 0.005 | 0.009 | 0.012 | 0.011 | 0.005 | 0.005 | 0.009 |
| meta892 |  | 350.099 | 364.281 | 0.091 | 0.102 | 0.095 | 0.047 | 0.092 | 0.051 | 0.072 | 0.138 | 0.122 | 0.117 | 0.122 | 0.117 | 0.121 | 0.057 | 0.138 |
| meta893 |  | 350.179 | 401.160 | 0.423 | 0.378 | 0.395 | 0.317 | 0.503 | 0.256 | 0.309 | 0.414 | 0.443 | 0.275 | 0.408 | 0.516 | 0.848 | 0.436 | 0.413 |
| meta894 |  | 350.303 | 35.775 | 0.012 | 0.016 | 0.013 | 0.029 | 0.027 | 0.017 | 0.031 | 0.037 | 0.026 | 0.024 | 0.026 | 0.029 | 0.026 | 0.026 | 0.042 |
| meta895 | Tyr-Met | 351.075 | 417.512 | 0.038 | 0.047 | 0.042 | 0.035 | 0.051 | 0.042 | 0.043 | 0.053 | 0.057 | 0.041 | 0.039 | 0.041 | 0.045 | 0.040 | 0.054 |
| meta896 |  | 351.095 | 406.049 | 0.070 | 0.073 | 0.052 | 0.033 | 0.065 | 0.020 | 0.107 | 0.263 | 0.101 | 0.075 | 0.109 | 0.111 | 0.068 | 0.025 | 0.050 |
| meta897 | Argininosuccinic acid | 351.158 | 358.404 | 0.066 | 0.058 | 0.062 | 0.046 | 0.049 | 0.045 | 0.047 | 0.061 | 0.074 | 0.076 | 0.058 | 0.064 | 0.072 | 0.061 | 0.070 |
| meta898 | Phe-Trp | 352.163 | 155.010 | 0.009 | 0.009 | 0.007 | 0.013 | 0.004 | 0.007 | 0.008 | 0.003 | 0.005 | 0.011 | 0.005 | 0.006 | 0.009 | 0.007 | 0.010 |
| meta899 |  | 352.242 | 252.377 | 0.169 | 0.207 | 0.190 | 0.351 | 0.172 | 0.365 | 0.056 | 0.101 | 0.162 | 0.038 | 0.162 | 0.076 | 0.007 | 0.026 | 0.161 |
| meta900 |  | 352.246 | 41.626 | 0.072 | 0.082 | 0.097 | 0.095 | 0.093 | 0.141 | 0.101 | 0.101 | 0.123 | 0.099 | 0.128 | 0.146 | 0.128 | 0.153 | 0.124 |
| meta901 |  | 352.319 | 34.702 | 0.046 | 0.061 | 0.057 | 0.115 | 0.127 | 0.062 | 0.165 | 0.128 | 0.118 | 0.096 | 0.097 | 0.115 | 0.092 | 0.106 | 0.208 |
| meta902 |  | 353.061 | 27.218 | 0.007 | 0.008 | 0.009 | 0.008 | 0.005 | 0.008 | 0.004 | 0.024 | 0.059 | 0.009 | 0.019 | 0.005 | 0.005 | 0.003 | 0.003 |
| meta903 |  | 353.110 | 422.504 | 0.025 | 0.022 | 0.027 | 0.010 | 0.013 | 0.015 | 0.012 | 0.040 | 0.048 | 0.070 | 0.021 | 0.025 | 0.026 | 0.026 | 0.020 |
| meta904 |  | 353.185 | 152.450 | 0.025 | 0.026 | 0.021 | 0.050 | 0.007 | 0.023 | 0.011 | 0.016 | 0.011 | 0.016 | 0.048 | 0.029 | 0.007 | 0.022 | 0.059 |
| meta905 | Arg-Cys | 354.048 | 347.665 | 0.006 | 0.007 | 0.007 | 0.006 | 0.008 | 0.005 | 0.008 | 0.007 | 0.008 | 0.006 | 0.006 | 0.007 | 0.008 | 0.008 | 0.006 |
| meta906 |  | 354.154 | 227.192 | 0.011 | 0.014 | 0.016 | 0.007 | 0.016 | 0.008 | 0.025 | 0.009 | 0.013 | 0.011 | 0.007 | 0.006 | 0.016 | 0.008 | 0.006 |
| meta907 | Phytanic acid | 354.335 | 34.136 | 0.022 | 0.027 | 0.031 | 0.069 | 0.063 | 0.037 | 0.072 | 0.083 | 0.055 | 0.045 | 0.059 | 0.066 | 0.047 | 0.066 | 0.118 |
| meta908 | 4,6-Dioxoheptanoic acid | 355.071 | 372.854 | 0.020 | 0.019 | 0.015 | 0.016 | 0.015 | 0.013 | 0.020 | 0.023 | 0.023 | 0.024 | 0.018 | 0.017 | 0.018 | 0.015 | 0.018 |
| meta909 |  | 355.090 | 406.057 | 0.264 | 0.322 | 0.295 | 0.249 | 0.256 | 0.203 | 0.297 | 0.280 | 0.374 | 0.319 | 0.283 | 0.228 | 0.293 | 0.231 | 0.332 |
| meta910 |  | 355.134 | 222.511 | 0.049 | 0.049 | 0.042 | 0.040 | 0.048 | 0.045 | 0.048 | 0.040 | 0.037 | 0.045 | 0.057 | 0.055 | 0.043 | 0.048 | 0.046 |
| meta911 |  | 355.161 | 396.799 | 0.016 | 0.015 | 0.016 | 0.015 | 0.013 | 0.012 | 0.012 | 0.015 | 0.009 | 0.011 | 0.014 | 0.012 | 0.013 | 0.007 | 0.010 |
| meta912 |  | 356.074 | 443.246 | 0.240 | 0.251 | 0.223 | 0.192 | 0.209 | 0.165 | 0.248 | 0.250 | 0.309 | 0.278 | 0.221 | 0.189 | 0.253 | 0.182 | 0.252 |
| meta913 |  | 356.123 | 49.984 | 0.012 | 0.011 | 0.011 | 0.009 | 0.011 | 0.010 | 0.009 | 0.008 | 0.008 | 0.018 | 0.009 | 0.011 | 0.014 | 0.009 | 0.011 |
| meta914 | (+-)5,6-DHET | 356.278 | 40.981 | 0.056 | 0.066 | 0.061 | 0.065 | 0.056 | 0.057 | 0.077 | 0.085 | 0.071 | 0.069 | 0.080 | 0.071 | 0.081 | 0.081 | 0.070 |
| meta915 |  | 356.936 | 286.486 | 0.021 | 0.015 | 0.012 | 0.014 | 0.021 | 0.015 | 0.016 | 0.018 | 0.013 | 0.017 | 0.018 | 0.019 | 0.016 | 0.013 | 0.015 |
| meta916 |  | 357.164 | 341.335 | 0.025 | 0.020 | 0.022 | 0.017 | 0.021 | 0.018 | 0.019 | 0.025 | 0.022 | 0.030 | 0.019 | 0.015 | 0.026 | 0.012 | 0.014 |
| meta917 |  | 357.211 | 375.002 | 0.254 | 0.249 | 0.235 | 0.211 | 0.270 | 0.252 | 0.179 | 0.195 | 0.132 | 0.208 | 0.189 | 0.210 | 0.256 | 0.225 | 0.227 |
| meta918 |  | 357.247 | 488.918 | 0.180 | 0.262 | 0.284 | 0.208 | 0.238 | 0.243 | 0.263 | 0.198 | 0.254 | 0.160 | 0.159 | 0.182 | 0.290 | 0.252 | 0.249 |
| meta919 |  | 357.261 | 45.096 | 0.037 | 0.033 | 0.029 | 0.032 | 0.037 | 0.040 | 0.039 | 0.062 | 0.047 | 0.040 | 0.039 | 0.031 | 0.023 | 0.028 | 0.017 |
| meta920 |  | 357.298 | 34.136 | 0.007 | 0.010 | 0.006 | 0.010 | 0.012 | 0.010 | 0.015 | 0.019 | 0.023 | 0.016 | 0.014 | 0.013 | 0.011 | 0.024 | 0.015 |
| meta921 |  | 358.045 | 380.257 | 0.074 | 0.048 | 0.056 | 0.020 | 0.097 | 0.018 | 0.250 | 0.021 | 0.124 | 0.049 | 0.020 | 0.021 | 0.100 | 0.035 | 0.019 |
| meta922 | Phe-Glu | 358.132 | 266.871 | 0.107 | 0.148 | 0.152 | 0.077 | 0.094 | 0.194 | 0.081 | 0.139 | 0.117 | 0.131 | 0.159 | 0.183 | 0.073 | 0.150 | 0.087 |
| meta923 |  | 358.204 | 47.943 | 0.052 | 0.035 | 0.047 | 0.019 | 0.018 | 0.015 | 0.025 | 0.023 | 0.024 | 0.024 | 0.023 | 0.023 | 0.026 | 0.022 | 0.024 |
| meta924 |  | 358.232 | 162.493 | 0.044 | 0.045 | 0.049 | 0.067 | 0.025 | 0.075 | 0.056 | 0.018 | 0.014 | 0.038 | 0.018 | 0.045 | 0.036 | 0.036 | 0.061 |
| meta925 |  | 358.257 | 191.285 | 0.027 | 0.033 | 0.028 | 0.018 | 0.048 | 0.020 | 0.025 | 0.049 | 0.027 | 0.033 | 0.025 | 0.028 | 0.023 | 0.021 | 0.040 |
| meta926 | Prunasin | 359.122 | 427.389 | 0.114 | 0.158 | 0.152 | 0.175 | 0.148 | 0.107 | 0.132 | 0.132 | 0.137 | 0.123 | 0.140 | 0.125 | 0.107 | 0.130 | 0.131 |
| meta927 | (-)-Riboflavin | 359.133 | 96.335 | 0.112 | 0.194 | 0.145 | 0.196 | 0.254 | 0.190 | 0.252 | 0.187 | 0.146 | 0.151 | 0.183 | 0.174 | 0.106 | 0.121 | 0.235 |
| meta928 | Tolazoline | 359.172 | 306.295 | 0.002 | 0.003 | 0.002 | 0.013 | 0.018 | 0.040 | 0.275 | 0.004 | 0.003 | 0.003 | 0.003 | 0.003 | 0.140 | 0.058 | 0.022 |
| meta929 |  | 359.201 | 348.892 | 0.092 | 0.091 | 0.083 | 0.071 | 0.073 | 0.094 | 0.053 | 0.102 | 0.054 | 0.101 | 0.138 | 0.230 | 0.082 | 0.089 | 0.080 |
| meta930 |  | 359.227 | 300.701 | 0.106 | 0.094 | 0.099 | 0.029 | 0.045 | 0.093 | 0.519 | 0.047 | 0.265 | 0.075 | 0.058 | 0.054 | 0.256 | 0.125 | 0.043 |
| meta931 | Norethindrone | 359.227 | 236.250 | 0.032 | 0.024 | 0.025 | 0.004 | 0.010 | 0.010 | 0.163 | 0.020 | 0.068 | 0.021 | 0.010 | 0.013 | 0.061 | 0.018 | 0.007 |
| meta932 |  | 360.010 | 280.040 | 0.005 | 0.007 | 0.006 | 0.002 | 0.018 | 0.002 | 0.013 | 0.002 | 0.010 | 0.006 | 0.001 | 0.002 | 0.008 | 0.003 | 0.002 |
| meta933 | Isomaltose | 360.148 | 370.656 | 1.116 | 1.047 | 0.956 | 0.774 | 1.135 | 1.413 | 0.963 | 1.092 | 0.634 | 0.982 | 1.148 | 1.379 | 0.758 | 0.841 | 0.556 |
| meta934 | Ellipticine | 360.992 | 293.716 | 0.026 | 0.029 | 0.027 | 0.029 | 0.029 | 0.026 | 0.035 | 0.023 | 0.009 | 0.011 | 0.015 | 0.020 | 0.029 | 0.015 | 0.025 |
| meta935 | Indole-3-carboxylic acid | 361.065 | 295.978 | 0.028 | 0.021 | 0.018 | 0.020 | 0.016 | 0.015 | 0.016 | 0.020 | 0.023 | 0.037 | 0.023 | 0.019 | 0.015 | 0.020 | 0.022 |
| meta936 | Tyr-Leu | 361.116 | 389.542 | 0.045 | 0.050 | 0.056 | 0.024 | 0.041 | 0.023 | 0.104 | 0.037 | 0.100 | 0.049 | 0.019 | 0.037 | 0.086 | 0.031 | 0.029 |
| meta937 | Pyridoxine | 361.131 | 232.420 | 0.040 | 0.049 | 0.034 | 0.038 | 0.048 | 0.033 | 0.050 | 0.053 | 0.056 | 0.059 | 0.055 | 0.049 | 0.039 | 0.042 | 0.048 |
| meta938 |  | 361.271 | 220.760 | 0.085 | 0.089 | 0.078 | 0.083 | 0.100 | 0.071 | 0.165 | 0.105 | 0.064 | 0.072 | 0.110 | 0.090 | 0.072 | 0.080 | 0.093 |
| meta939 |  | 362.082 | 362.180 | 3.941 | 4.483 | 4.026 | 3.917 | 4.291 | 2.972 | 4.545 | 3.879 | 3.346 | 3.892 | 4.416 | 3.791 | 3.410 | 3.064 | 4.826 |
| meta940 | Enoxacin | 362.154 | 232.297 | 0.007 | 0.007 | 0.004 | 0.003 | 0.003 | 0.002 | 0.024 | 0.004 | 0.012 | 0.015 | 0.004 | 0.005 | 0.003 | 0.003 | 0.003 |
| meta941 |  | 362.215 | 345.464 | 0.078 | 0.079 | 0.069 | 0.078 | 0.099 | 0.070 | 0.079 | 0.123 | 0.053 | 0.055 | 0.086 | 0.039 | 0.052 | 0.053 | 0.073 |
| meta942 |  | 363.095 | 418.854 | 0.117 | 0.127 | 0.132 | 0.110 | 0.087 | 0.102 | 0.119 | 0.114 | 0.173 | 0.135 | 0.139 | 0.174 | 0.135 | 0.120 | 0.116 |
| meta943 | 3-Amino-3-(4-hydroxyphenyl)propanoate | 363.160 | 48.770 | 0.017 | 0.013 | 0.014 | 0.015 | 0.021 | 0.009 | 0.013 | 0.013 | 0.011 | 0.008 | 0.007 | 0.008 | 0.008 | 0.008 | 0.011 |
| meta944 | Hydrocortisone | 363.214 | 156.155 | 0.047 | 0.049 | 0.043 | 0.028 | 0.121 | 0.022 | 0.067 | 0.057 | 0.012 | 0.040 | 0.055 | 0.020 | 0.030 | 0.023 | 0.087 |
| meta945 |  | 363.308 | 33.573 | 0.013 | 0.015 | 0.013 | 0.018 | 0.013 | 0.016 | 0.051 | 0.015 | 0.016 | 0.023 | 0.015 | 0.012 | 0.015 | 0.013 | 0.013 |
| meta946 | Xanthylic acid (XMP) | 364.049 | 280.506 | 0.002 | 0.003 | 0.003 | 0.004 | 0.015 | 0.041 | 0.031 | 0.050 | 0.004 | 0.004 | 0.005 | 0.006 | 0.004 | 0.007 | 0.004 |
| meta947 |  | 364.063 | 452.458 | 0.159 | 0.147 | 0.157 | 0.140 | 0.148 | 0.145 | 0.161 | 0.199 | 0.159 | 0.160 | 0.158 | 0.156 | 0.194 | 0.146 | 0.186 |
| meta948 |  | 364.083 | 32.322 | 0.010 | 0.008 | 0.008 | 0.007 | 0.104 | 0.007 | 0.003 | 0.007 | 0.007 | 0.005 | 0.014 | 0.005 | 0.003 | 0.038 | 0.013 |
| meta949 |  | 364.120 | 399.492 | 5.368 | 5.304 | 5.362 | 6.389 | 5.607 | 4.858 | 4.858 | 5.229 | 2.874 | 3.636 | 4.637 | 4.895 | 3.725 | 4.186 | 4.021 |
| meta950 |  | 364.127 | 387.317 | 0.775 | 0.730 | 0.793 | 0.497 | 0.396 | 0.507 | 0.538 | 0.588 | 0.964 | 0.915 | 0.724 | 0.763 | 0.931 | 0.613 | 0.584 |
| meta951 |  | 364.172 | 27.382 | 0.025 | 0.032 | 0.028 | 0.033 | 0.042 | 0.022 | 0.027 | 0.035 | 0.029 | 0.026 | 0.029 | 0.033 | 0.027 | 0.032 | 0.029 |
| meta952 |  | 364.231 | 314.738 | 0.005 | 0.006 | 0.004 | 0.004 | 0.009 | 0.002 | 0.008 | 0.004 | 0.001 | 0.002 | 0.003 | 0.003 | 0.003 | 0.001 | 0.014 |
| meta953 |  | 364.282 | 106.578 | 0.019 | 0.024 | 0.020 | 0.033 | 0.076 | 0.029 | 0.043 | 0.023 | 0.013 | 0.016 | 0.007 | 0.024 | 0.009 | 0.015 | 0.018 |
| meta954 |  | 365.104 | 370.179 | 2.943 | 3.280 | 2.954 | 2.964 | 3.581 | 3.713 | 2.843 | 3.129 | 2.480 | 2.858 | 3.275 | 3.574 | 2.330 | 3.011 | 2.017 |
| meta955 |  | 365.190 | 106.793 | 0.038 | 0.058 | 0.053 | 0.104 | 0.075 | 0.087 | 0.056 | 0.036 | 0.018 | 0.047 | 0.032 | 0.029 | 0.053 | 0.056 | 0.057 |
| meta956 |  | 365.275 | 152.481 | 0.006 | 0.008 | 0.006 | 0.008 | 0.008 | 0.006 | 0.007 | 0.005 | 0.009 | 0.006 | 0.006 | 0.006 | 0.006 | 0.005 | 0.009 |
| meta957 |  | 366.094 | 441.026 | 0.082 | 0.073 | 0.080 | 0.063 | 0.081 | 0.075 | 0.089 | 0.075 | 0.069 | 0.072 | 0.067 | 0.069 | 0.088 | 0.063 | 0.072 |
| meta958 |  | 366.098 | 33.991 | 0.031 | 0.026 | 0.040 | 0.014 | 0.014 | 0.023 | 0.033 | 0.061 | 0.055 | 0.041 | 0.028 | 0.006 | 0.026 | 0.028 | 0.009 |
| meta959 | Famciclovir | 366.107 | 369.975 | 0.408 | 0.426 | 0.389 | 0.383 | 0.457 | 0.471 | 0.382 | 0.460 | 0.319 | 0.376 | 0.389 | 0.491 | 0.325 | 0.402 | 0.270 |
| meta960 | Phe-His | 366.155 | 390.875 | 0.012 | 0.013 | 0.015 | 0.009 | 0.015 | 0.009 | 0.016 | 0.013 | 0.011 | 0.009 | 0.012 | 0.020 | 0.015 | 0.013 | 0.028 |
| meta961 |  | 366.985 | 288.556 | 0.034 | 0.037 | 0.035 | 0.031 | 0.032 | 0.026 | 0.037 | 0.037 | 0.044 | 0.043 | 0.031 | 0.028 | 0.035 | 0.030 | 0.040 |
| meta962 | Trimethoprim | 367.050 | 431.385 | 0.053 | 0.052 | 0.050 | 0.058 | 0.051 | 0.038 | 0.046 | 0.051 | 0.055 | 0.041 | 0.051 | 0.048 | 0.047 | 0.051 | 0.058 |
| meta963 |  | 367.053 | 25.928 | 0.010 | 0.015 | 0.012 | 0.018 | 0.012 | 0.012 | 0.013 | 0.016 | 0.020 | 0.018 | 0.016 | 0.011 | 0.009 | 0.010 | 0.010 |
| meta964 |  | 367.054 | 202.206 | 0.052 | 0.055 | 0.058 | 0.053 | 0.072 | 0.049 | 0.067 | 0.046 | 0.048 | 0.048 | 0.056 | 0.057 | 0.057 | 0.053 | 0.050 |
| meta965 |  | 367.141 | 340.893 | 0.225 | 0.204 | 0.202 | 0.186 | 0.235 | 0.181 | 0.303 | 0.234 | 0.156 | 0.178 | 0.199 | 0.176 | 0.228 | 0.197 | 0.215 |
| meta966 |  | 367.137 | 466.644 | 0.442 | 0.460 | 0.376 | 0.550 | 0.556 | 0.306 | 0.315 | 1.577 | 0.489 | 0.311 | 0.475 | 0.376 | 0.205 | 0.247 | 0.436 |
| meta967 | 25-Hydroxycholesterol | 367.333 | 33.382 | 0.017 | 0.021 | 0.019 | 0.011 | 0.011 | 0.006 | 0.021 | 0.017 | 0.011 | 0.012 | 0.020 | 0.018 | 0.011 | 0.007 | 0.014 |
| meta968 |  | 367.365 | 26.383 | 0.013 | 0.015 | 0.012 | 0.022 | 0.015 | 0.012 | 0.023 | 0.014 | 0.013 | 0.018 | 0.019 | 0.023 | 0.010 | 0.013 | 0.021 |
| meta969 |  | 368.092 | 346.105 | 0.038 | 0.029 | 0.033 | 0.014 | 0.053 | 0.022 | 0.078 | 0.021 | 0.080 | 0.030 | 0.031 | 0.050 | 0.021 | 0.026 | 0.009 |
| meta970 |  | 368.108 | 362.324 | 0.060 | 0.077 | 0.081 | 0.066 | 0.092 | 0.065 | 0.066 | 0.065 | 0.066 | 0.081 | 0.073 | 0.071 | 0.053 | 0.054 | 0.080 |
| meta971 |  | 368.350 | 102.048 | 0.049 | 0.044 | 0.042 | 0.048 | 0.066 | 0.053 | 0.079 | 0.082 | 0.090 | 0.053 | 0.095 | 0.146 | 0.078 | 0.100 | 0.098 |
| meta972 |  | 369.105 | 413.272 | 0.035 | 0.029 | 0.026 | 0.014 | 0.019 | 0.014 | 0.015 | 0.053 | 0.050 | 0.081 | 0.025 | 0.038 | 0.022 | 0.035 | 0.020 |
| meta973 |  | 369.211 | 370.606 | 0.165 | 0.172 | 0.167 | 0.174 | 0.297 | 0.167 | 0.138 | 0.124 | 0.103 | 0.128 | 0.106 | 0.135 | 0.142 | 0.154 | 0.157 |
| meta974 |  | 369.247 | 493.285 | 0.043 | 0.065 | 0.069 | 0.052 | 0.058 | 0.062 | 0.064 | 0.054 | 0.063 | 0.040 | 0.039 | 0.047 | 0.071 | 0.059 | 0.068 |
| meta975 |  | 369.349 | 32.682 | 0.105 | 0.115 | 0.094 | 0.025 | 0.023 | 0.013 | 0.023 | 0.071 | 0.025 | 0.029 | 0.086 | 0.115 | 0.031 | 0.011 | 0.025 |
| meta976 |  | 370.752 | 287.956 | 0.066 | 0.062 | 0.069 | 0.075 | 0.073 | 0.064 | 0.072 | 0.073 | 0.087 | 0.074 | 0.078 | 0.073 | 0.085 | 0.079 | 0.076 |
| meta977 |  | 371.030 | 152.578 | 0.027 | 0.034 | 0.027 | 0.027 | 0.043 | 0.018 | 0.040 | 0.029 | 0.019 | 0.015 | 0.020 | 0.023 | 0.022 | 0.023 | 0.027 |
| meta978 |  | 371.034 | 435.671 | 0.034 | 0.039 | 0.042 | 0.031 | 0.040 | 0.034 | 0.036 | 0.036 | 0.032 | 0.039 | 0.041 | 0.041 | 0.042 | 0.032 | 0.041 |
| meta979 | Tyr-Glu | 371.153 | 368.401 | 0.067 | 0.080 | 0.071 | 0.067 | 0.025 | 0.025 | 0.008 | 0.090 | 0.003 | 0.065 | 0.018 | 0.019 | 0.008 | 0.020 | 0.008 |
| meta980 |  | 371.227 | 105.756 | 0.030 | 0.037 | 0.029 | 0.048 | 0.042 | 0.036 | 0.063 | 0.027 | 0.034 | 0.039 | 0.030 | 0.026 | 0.044 | 0.044 | 0.065 |
| meta981 |  | 371.252 | 209.920 | 0.014 | 0.014 | 0.013 | 0.008 | 0.014 | 0.007 | 0.018 | 0.020 | 0.015 | 0.014 | 0.014 | 0.011 | 0.008 | 0.005 | 0.019 |
| meta982 |  | 371.346 | 27.545 | 0.053 | 0.070 | 0.064 | 0.076 | 0.066 | 0.041 | 0.061 | 0.081 | 0.067 | 0.057 | 0.071 | 0.068 | 0.067 | 0.071 | 0.069 |
| meta983 |  | 372.076 | 159.907 | 0.118 | 0.136 | 0.115 | 0.127 | 0.135 | 0.091 | 0.109 | 0.131 | 0.147 | 0.125 | 0.137 | 0.113 | 0.136 | 0.112 | 0.137 |
| meta984 |  | 372.176 | 388.123 | 0.020 | 0.021 | 0.020 | 0.020 | 0.019 | 0.020 | 0.019 | 0.023 | 0.016 | 0.014 | 0.012 | 0.020 | 0.016 | 0.017 | 0.012 |
| meta985 |  | 372.236 | 221.036 | 0.013 | 0.012 | 0.013 | 0.007 | 0.008 | 0.009 | 0.010 | 0.033 | 0.029 | 0.023 | 0.024 | 0.007 | 0.006 | 0.005 | 0.009 |
| meta986 |  | 372.309 | 158.663 | 0.189 | 0.164 | 0.118 | 0.095 | 0.311 | 0.095 | 0.308 | 0.335 | 0.261 | 0.127 | 0.125 | 0.098 | 0.211 | 0.194 | 0.170 |
| meta987 |  | 372.827 | 287.459 | 0.015 | 0.011 | 0.012 | 0.015 | 0.018 | 0.018 | 0.016 | 0.016 | 0.022 | 0.020 | 0.016 | 0.018 | 0.028 | 0.019 | 0.016 |
| meta988 |  | 373.156 | 151.873 | 0.007 | 0.009 | 0.006 | 0.007 | 0.007 | 0.007 | 0.003 | 0.005 | 0.005 | 0.008 | 0.011 | 0.009 | 0.008 | 0.007 | 0.011 |
| meta989 |  | 373.151 | 28.018 | 0.006 | 0.005 | 0.005 | 0.007 | 0.007 | 0.004 | 0.004 | 0.024 | 0.031 | 0.008 | 0.003 | 0.003 | 0.005 | 0.033 | 0.006 |
| meta990 |  | 374.224 | 252.280 | 0.059 | 0.060 | 0.052 | 0.089 | 0.051 | 0.085 | 0.016 | 0.026 | 0.057 | 0.006 | 0.062 | 0.030 | 0.002 | 0.008 | 0.056 |
| meta991 |  | 374.251 | 215.895 | 0.065 | 0.069 | 0.068 | 0.047 | 0.054 | 0.057 | 0.063 | 0.149 | 0.114 | 0.090 | 0.110 | 0.036 | 0.043 | 0.037 | 0.045 |
| meta992 |  | 374.252 | 299.334 | 0.009 | 0.010 | 0.008 | 0.008 | 0.009 | 0.007 | 0.017 | 0.013 | 0.008 | 0.009 | 0.009 | 0.005 | 0.014 | 0.005 | 0.004 |
| meta993 |  | 375.079 | 431.462 | 0.046 | 0.042 | 0.052 | 0.047 | 0.057 | 0.027 | 0.031 | 0.027 | 0.027 | 0.031 | 0.036 | 0.044 | 0.051 | 0.057 | 0.040 |
| meta994 | Arg-Arg | 375.186 | 280.290 | 0.003 | 0.004 | 0.002 | 0.002 | 0.004 | 0.003 | 0.002 | 0.002 | 0.002 | 0.003 | 0.004 | 0.015 | 0.017 | 0.003 | 0.012 |
| meta995 |  | 375.197 | 369.286 | 0.022 | 0.023 | 0.026 | 0.023 | 0.026 | 0.024 | 0.028 | 0.023 | 0.018 | 0.026 | 0.024 | 0.025 | 0.023 | 0.018 | 0.021 |
| meta996 |  | 375.222 | 476.337 | 0.172 | 0.200 | 0.184 | 0.160 | 0.191 | 0.147 | 0.189 | 0.184 | 0.170 | 0.154 | 0.161 | 0.170 | 0.189 | 0.160 | 0.192 |
| meta997 |  | 376.049 | 275.213 | 0.458 | 0.418 | 0.372 | 0.372 | 0.498 | 0.388 | 0.308 | 0.612 | 0.378 | 0.646 | 0.384 | 0.204 | 0.418 | 0.444 | 0.409 |
| meta998 | Matairesinol | 376.174 | 323.972 | 0.002 | 0.003 | 0.002 | 0.003 | 0.003 | 0.002 | 0.002 | 0.004 | 0.004 | 0.005 | 0.003 | 0.005 | 0.003 | 0.003 | 0.003 |
| meta999 |  | 376.194 | 389.455 | 0.074 | 0.072 | 0.068 | 0.063 | 0.095 | 0.050 | 0.071 | 0.091 | 0.068 | 0.068 | 0.064 | 0.086 | 0.116 | 0.049 | 0.075 |
| meta1000 |  | 376.206 | 431.337 | 0.024 | 0.021 | 0.017 | 0.018 | 0.019 | 0.014 | 0.020 | 0.022 | 0.014 | 0.019 | 0.018 | 0.016 | 0.018 | 0.018 | 0.020 |
| meta1001 |  | 376.258 | 33.198 | 0.133 | 0.162 | 0.116 | 0.480 | 0.310 | 0.172 | 0.347 | 0.269 | 0.275 | 0.212 | 0.278 | 0.316 | 0.213 | 0.321 | 0.286 |
| meta1002 |  | 376.786 | 287.956 | 0.177 | 0.176 | 0.186 | 0.194 | 0.213 | 0.179 | 0.190 | 0.206 | 0.221 | 0.212 | 0.203 | 0.192 | 0.238 | 0.201 | 0.203 |
| meta1003 | 2'-Deoxyinosine 5'-monophosphate | 377.027 | 382.130 | 0.003 | 0.004 | 0.004 | 0.005 | 0.006 | 0.004 | 0.002 | 0.005 | 0.003 | 0.004 | 0.006 | 0.005 | 0.005 | 0.005 | 0.003 |
| meta1004 | Demethoxycurcumin | 377.072 | 406.057 | 0.052 | 0.068 | 0.056 | 0.062 | 0.051 | 0.044 | 0.062 | 0.061 | 0.076 | 0.063 | 0.057 | 0.046 | 0.058 | 0.047 | 0.072 |
| meta1005 | Riboflavin | 377.144 | 196.926 | 0.052 | 0.065 | 0.056 | 0.056 | 0.067 | 0.051 | 0.074 | 0.068 | 0.049 | 0.052 | 0.056 | 0.055 | 0.049 | 0.052 | 0.063 |
| meta1006 |  | 377.207 | 288.908 | 0.009 | 0.013 | 0.013 | 0.012 | 0.013 | 0.011 | 0.014 | 0.013 | 0.016 | 0.012 | 0.015 | 0.012 | 0.014 | 0.012 | 0.012 |
| meta1007 |  | 378.114 | 396.208 | 0.261 | 0.217 | 0.210 | 0.214 | 0.242 | 0.176 | 0.250 | 0.291 | 0.152 | 0.237 | 0.293 | 0.188 | 0.165 | 0.129 | 0.241 |
| meta1008 | Ranitidine | 378.148 | 372.901 | 0.108 | 0.111 | 0.097 | 0.082 | 0.108 | 0.083 | 0.113 | 0.124 | 0.128 | 0.111 | 0.121 | 0.089 | 0.135 | 0.099 | 0.081 |
| meta1009 |  | 378.209 | 385.625 | 0.010 | 0.008 | 0.011 | 0.012 | 0.017 | 0.007 | 0.009 | 0.014 | 0.009 | 0.009 | 0.006 | 0.017 | 0.017 | 0.014 | 0.007 |
| meta1010 |  | 378.262 | 40.087 | 0.042 | 0.064 | 0.054 | 0.053 | 0.076 | 0.065 | 0.092 | 0.073 | 0.077 | 0.062 | 0.099 | 0.088 | 0.055 | 0.087 | 0.078 |
| meta1011 |  | 379.115 | 26.383 | 0.016 | 0.018 | 0.014 | 0.022 | 0.008 | 0.013 | 0.031 | 0.012 | 0.022 | 0.015 | 0.018 | 0.026 | 0.022 | 0.028 | 0.020 |
| meta1012 |  | 379.294 | 192.883 | 0.003 | 0.003 | 0.002 | 0.002 | 0.003 | 0.002 | 0.006 | 0.010 | 0.003 | 0.004 | 0.003 | 0.004 | 0.001 | 0.002 | 0.002 |
| meta1013 |  | 380.058 | 451.732 | 0.030 | 0.030 | 0.031 | 0.028 | 0.026 | 0.022 | 0.028 | 0.029 | 0.030 | 0.027 | 0.032 | 0.029 | 0.031 | 0.031 | 0.034 |
| meta1014 |  | 380.093 | 399.754 | 0.473 | 0.489 | 0.445 | 0.550 | 0.528 | 0.451 | 0.468 | 0.479 | 0.281 | 0.345 | 0.424 | 0.449 | 0.401 | 0.383 | 0.414 |
| meta1015 | S-Lactoylglutathione | 380.110 | 376.541 | 0.111 | 0.106 | 0.097 | 0.105 | 0.284 | 0.075 | 0.152 | 0.070 | 0.091 | 0.109 | 0.085 | 0.062 | 0.102 | 0.096 | 0.094 |
| meta1016 |  | 380.122 | 418.663 | 1.383 | 1.789 | 1.654 | 1.404 | 0.898 | 1.014 | 1.257 | 1.269 | 2.153 | 1.572 | 1.641 | 1.694 | 1.752 | 1.509 | 1.485 |
| meta1017 |  | 380.200 | 426.666 | 0.041 | 0.042 | 0.047 | 0.056 | 0.034 | 0.054 | 0.047 | 0.020 | 0.024 | 0.023 | 0.029 | 0.026 | 0.046 | 0.028 | 0.039 |
| meta1018 |  | 380.368 | 27.545 | 0.006 | 0.008 | 0.007 | 0.014 | 0.010 | 0.010 | 0.011 | 0.006 | 0.009 | 0.010 | 0.008 | 0.008 | 0.007 | 0.009 | 0.012 |
| meta1019 |  | 380.872 | 318.454 | 0.031 | 0.029 | 0.035 | 0.029 | 0.031 | 0.034 | 0.031 | 0.028 | 0.038 | 0.029 | 0.030 | 0.029 | 0.037 | 0.040 | 0.031 |
| meta1020 |  | 380.898 | 318.454 | 0.024 | 0.022 | 0.026 | 0.021 | 0.022 | 0.027 | 0.022 | 0.020 | 0.028 | 0.021 | 0.024 | 0.021 | 0.027 | 0.030 | 0.021 |
| meta1021 |  | 381.077 | 370.504 | 0.358 | 0.395 | 0.334 | 0.316 | 0.361 | 0.409 | 0.314 | 0.347 | 0.267 | 0.338 | 0.355 | 0.423 | 0.256 | 0.332 | 0.203 |
| meta1022 | N-Carbamylglutamate | 381.131 | 261.302 | 0.023 | 0.019 | 0.021 | 0.012 | 0.014 | 0.010 | 0.011 | 0.019 | 0.022 | 0.033 | 0.036 | 0.035 | 0.028 | 0.018 | 0.039 |
| meta1023 | Papaverine | 381.186 | 123.717 | 0.014 | 0.010 | 0.011 | 0.038 | 0.014 | 0.039 | 0.032 | 0.007 | 0.006 | 0.017 | 0.007 | 0.001 | 0.011 | 0.011 | 0.021 |
| meta1024 | Ile-Trp | 381.184 | 151.728 | 0.011 | 0.012 | 0.011 | 0.013 | 0.010 | 0.011 | 0.009 | 0.009 | 0.007 | 0.009 | 0.010 | 0.011 | 0.010 | 0.007 | 0.010 |
| meta1025 |  | 382.028 | 431.033 | 0.016 | 0.016 | 0.013 | 0.016 | 0.016 | 0.013 | 0.020 | 0.013 | 0.013 | 0.012 | 0.012 | 0.014 | 0.012 | 0.012 | 0.015 |
| meta1026 | Clopidogrel | 382.094 | 399.837 | 0.049 | 0.048 | 0.046 | 0.051 | 0.053 | 0.045 | 0.045 | 0.054 | 0.033 | 0.035 | 0.048 | 0.049 | 0.039 | 0.040 | 0.040 |
| meta1027 | Capsaicin | 382.126 | 27.353 | 0.002 | 0.003 | 0.004 | 0.004 | 0.002 | 0.002 | 0.003 | 0.006 | 0.004 | 0.003 | 0.004 | 0.001 | 0.003 | 0.003 | 0.002 |
| meta1028 | Homovanillic acid | 382.143 | 400.530 | 0.007 | 0.007 | 0.008 | 0.009 | 0.005 | 0.008 | 0.035 | 0.041 | 0.021 | 0.003 | 0.007 | 0.010 | 0.026 | 0.007 | 0.004 |
| meta1029 |  | 382.293 | 158.053 | 0.043 | 0.055 | 0.042 | 0.041 | 0.073 | 0.049 | 0.080 | 0.114 | 0.083 | 0.083 | 0.045 | 0.056 | 0.040 | 0.037 | 0.075 |
| meta1030 |  | 382.874 | 573.666 | 0.024 | 0.024 | 0.021 | 0.028 | 0.032 | 0.025 | 0.031 | 0.030 | 0.030 | 0.030 | 0.034 | 0.019 | 0.028 | 0.030 | 0.027 |
| meta1031 |  | 383.027 | 25.928 | 0.005 | 0.006 | 0.007 | 0.008 | 0.006 | 0.005 | 0.006 | 0.007 | 0.008 | 0.008 | 0.006 | 0.005 | 0.004 | 0.005 | 0.003 |
| meta1032 |  | 383.039 | 382.079 | 0.014 | 0.017 | 0.014 | 0.013 | 0.016 | 0.011 | 0.015 | 0.015 | 0.014 | 0.015 | 0.019 | 0.014 | 0.011 | 0.013 | 0.014 |
| meta1033 | sn-Glycerol 1-phosphate | 383.111 | 380.454 | 0.062 | 0.072 | 0.066 | 0.100 | 0.093 | 0.057 | 0.095 | 0.070 | 0.059 | 0.062 | 0.073 | 0.055 | 0.059 | 0.059 | 0.059 |
| meta1034 | 25-hydroxyvitamin D3 | 383.328 | 33.411 | 0.025 | 0.031 | 0.028 | 0.028 | 0.029 | 0.016 | 0.032 | 0.034 | 0.021 | 0.035 | 0.046 | 0.033 | 0.016 | 0.011 | 0.021 |
| meta1035 |  | 384.064 | 362.500 | 0.122 | 0.201 | 0.161 | 0.160 | 0.181 | 0.110 | 0.159 | 0.153 | 0.138 | 0.149 | 0.167 | 0.142 | 0.136 | 0.129 | 0.170 |
| meta1036 |  | 384.096 | 38.946 | 0.011 | 0.011 | 0.008 | 0.009 | 0.009 | 0.005 | 0.013 | 0.011 | 0.009 | 0.013 | 0.012 | 0.012 | 0.010 | 0.009 | 0.018 |
| meta1037 | Leu-Trp | 384.130 | 418.680 | 0.092 | 0.107 | 0.094 | 0.091 | 0.102 | 0.084 | 0.109 | 0.090 | 0.081 | 0.093 | 0.069 | 0.111 | 0.179 | 0.125 | 0.149 |
| meta1038 | N-Acetyl-D-lactosamine | 384.148 | 348.905 | 0.035 | 0.035 | 0.032 | 0.031 | 0.039 | 0.036 | 0.035 | 0.041 | 0.034 | 0.040 | 0.030 | 0.030 | 0.032 | 0.022 | 0.037 |
| meta1039 |  | 384.154 | 179.803 | 0.007 | 0.008 | 0.006 | 0.005 | 0.005 | 0.003 | 0.007 | 0.008 | 0.006 | 0.008 | 0.010 | 0.011 | 0.007 | 0.007 | 0.006 |
| meta1040 | (-)-Epinephrine | 384.220 | 230.530 | 0.004 | 0.004 | 0.002 | 0.003 | 0.004 | 0.002 | 0.003 | 0.004 | 0.003 | 0.003 | 0.004 | 0.007 | 0.007 | 0.003 | 0.005 |
| meta1041 | S-Adenosyl-L-homocysteine | 385.127 | 366.674 | 2.507 | 2.844 | 2.588 | 2.640 | 2.875 | 2.142 | 2.660 | 2.707 | 2.681 | 2.544 | 2.491 | 1.895 | 2.833 | 2.106 | 2.332 |
| meta1042 |  | 385.233 | 46.820 | 0.020 | 0.023 | 0.022 | 0.021 | 0.032 | 0.022 | 0.034 | 0.027 | 0.017 | 0.015 | 0.029 | 0.034 | 0.027 | 0.025 | 0.027 |
| meta1043 |  | 386.040 | 148.440 | 0.021 | 0.021 | 0.022 | 0.024 | 0.029 | 0.023 | 0.029 | 0.021 | 0.026 | 0.025 | 0.019 | 0.015 | 0.023 | 0.028 | 0.031 |
| meta1044 |  | 386.144 | 278.517 | 0.073 | 0.104 | 0.100 | 0.088 | 0.084 | 0.082 | 0.101 | 0.085 | 0.089 | 0.073 | 0.078 | 0.086 | 0.093 | 0.092 | 0.095 |
| meta1045 |  | 386.215 | 342.866 | 0.010 | 0.009 | 0.009 | 0.012 | 0.010 | 0.011 | 0.010 | 0.017 | 0.010 | 0.008 | 0.009 | 0.011 | 0.007 | 0.007 | 0.009 |
| meta1046 |  | 386.288 | 183.291 | 0.042 | 0.048 | 0.047 | 0.036 | 0.096 | 0.023 | 0.039 | 0.084 | 0.052 | 0.049 | 0.035 | 0.054 | 0.044 | 0.026 | 0.075 |
| meta1047 |  | 386.324 | 155.725 | 0.018 | 0.025 | 0.019 | 0.020 | 0.055 | 0.009 | 0.059 | 0.041 | 0.035 | 0.024 | 0.023 | 0.016 | 0.041 | 0.019 | 0.027 |
| meta1048 |  | 387.004 | 152.578 | 0.007 | 0.006 | 0.005 | 0.006 | 0.010 | 0.004 | 0.007 | 0.007 | 0.005 | 0.004 | 0.005 | 0.005 | 0.005 | 0.004 | 0.005 |
| meta1049 |  | 388.076 | 441.061 | 0.019 | 0.016 | 0.017 | 0.016 | 0.017 | 0.014 | 0.019 | 0.013 | 0.014 | 0.014 | 0.014 | 0.016 | 0.016 | 0.012 | 0.017 |
| meta1050 |  | 388.303 | 182.622 | 0.050 | 0.041 | 0.040 | 0.042 | 0.100 | 0.031 | 0.060 | 0.067 | 0.047 | 0.037 | 0.048 | 0.058 | 0.041 | 0.036 | 0.051 |
| meta1051 |  | 389.237 | 415.459 | 0.016 | 0.018 | 0.016 | 0.019 | 0.021 | 0.016 | 0.014 | 0.025 | 0.015 | 0.015 | 0.021 | 0.018 | 0.022 | 0.015 | 0.019 |
| meta1052 | Adenosine 3',5'-cyclic phosphate (cAMP) | 390.086 | 159.783 | 0.010 | 0.012 | 0.013 | 0.011 | 0.012 | 0.006 | 0.008 | 0.010 | 0.011 | 0.010 | 0.010 | 0.010 | 0.008 | 0.010 | 0.013 |
| meta1053 |  | 391.100 | 278.637 | 0.360 | 0.487 | 0.477 | 0.449 | 0.432 | 0.358 | 0.540 | 0.471 | 0.484 | 0.380 | 0.442 | 0.422 | 0.464 | 0.420 | 0.496 |
| meta1054 | Loxapine | 391.131 | 374.660 | 0.011 | 0.009 | 0.010 | 0.007 | 0.008 | 0.010 | 0.004 | 0.009 | 0.008 | 0.010 | 0.010 | 0.010 | 0.007 | 0.005 | 0.009 |
| meta1055 |  | 391.205 | 261.268 | 0.048 | 0.048 | 0.056 | 0.039 | 0.059 | 0.043 | 0.058 | 0.057 | 0.021 | 0.052 | 0.045 | 0.051 | 0.055 | 0.035 | 0.055 |
| meta1056 | RU-0211 | 391.223 | 211.473 | 0.009 | 0.008 | 0.007 | 0.005 | 0.009 | 0.005 | 0.007 | 0.002 | 0.005 | 0.008 | 0.003 | 0.003 | 0.006 | 0.005 | 0.002 |
| meta1057 | Dioctyl phthalate | 391.282 | 18.807 | 0.072 | 0.082 | 0.078 | 0.079 | 0.084 | 0.060 | 0.076 | 0.081 | 0.041 | 0.078 | 0.077 | 0.068 | 0.075 | 0.074 | 0.059 |
| meta1058 |  | 391.316 | 28.184 | 0.025 | 0.037 | 0.031 | 0.085 | 0.042 | 0.018 | 0.030 | 0.051 | 0.037 | 0.029 | 0.043 | 0.053 | 0.028 | 0.042 | 0.039 |
| meta1059 | 2'-Deoxyadenosine 5'-monophosphate (dAMP) | 392.104 | 278.637 | 0.050 | 0.068 | 0.066 | 0.063 | 0.062 | 0.048 | 0.078 | 0.065 | 0.067 | 0.052 | 0.060 | 0.062 | 0.063 | 0.059 | 0.067 |
| meta1060 |  | 393.105 | 457.603 | 0.017 | 0.018 | 0.019 | 0.017 | 0.016 | 0.018 | 0.015 | 0.021 | 0.017 | 0.027 | 0.022 | 0.019 | 0.018 | 0.015 | 0.019 |
| meta1061 |  | 393.285 | 33.352 | 0.197 | 0.258 | 0.234 | 0.771 | 0.404 | 0.215 | 0.497 | 0.401 | 0.402 | 0.305 | 0.364 | 0.456 | 0.315 | 0.407 | 0.456 |
| meta1062 |  | 394.085 | 480.750 | 0.102 | 0.095 | 0.100 | 0.087 | 0.095 | 0.094 | 0.099 | 0.109 | 0.103 | 0.088 | 0.093 | 0.099 | 0.113 | 0.088 | 0.107 |
| meta1063 |  | 394.191 | 436.111 | 0.084 | 0.088 | 0.081 | 0.090 | 0.073 | 0.082 | 0.064 | 0.084 | 0.068 | 0.081 | 0.075 | 0.101 | 0.087 | 0.084 | 0.090 |
| meta1064 |  | 394.294 | 156.774 | 0.013 | 0.014 | 0.013 | 0.012 | 0.034 | 0.009 | 0.021 | 0.039 | 0.018 | 0.017 | 0.016 | 0.024 | 0.013 | 0.017 | 0.027 |
| meta1065 |  | 395.023 | 276.006 | 0.074 | 0.068 | 0.074 | 0.073 | 0.089 | 0.067 | 0.102 | 0.099 | 0.057 | 0.074 | 0.071 | 0.054 | 0.069 | 0.069 | 0.072 |
| meta1066 |  | 395.071 | 470.543 | 0.020 | 0.023 | 0.021 | 0.022 | 0.023 | 0.017 | 0.015 | 0.011 | 0.022 | 0.029 | 0.022 | 0.020 | 0.021 | 0.020 | 0.027 |
| meta1067 |  | 395.122 | 404.639 | 0.034 | 0.035 | 0.029 | 0.026 | 0.044 | 0.023 | 0.039 | 0.031 | 0.036 | 0.033 | 0.034 | 0.038 | 0.040 | 0.036 | 0.034 |
| meta1068 |  | 395.524 | 276.006 | 0.013 | 0.013 | 0.014 | 0.013 | 0.014 | 0.013 | 0.019 | 0.017 | 0.015 | 0.015 | 0.011 | 0.010 | 0.011 | 0.009 | 0.018 |
| meta1069 |  | 396.116 | 387.718 | 0.105 | 0.142 | 0.121 | 0.100 | 0.071 | 0.073 | 0.085 | 0.092 | 0.170 | 0.143 | 0.118 | 0.138 | 0.138 | 0.131 | 0.099 |
| meta1070 |  | 396.143 | 26.383 | 0.041 | 0.051 | 0.050 | 0.071 | 0.026 | 0.040 | 0.103 | 0.040 | 0.070 | 0.050 | 0.060 | 0.087 | 0.075 | 0.103 | 0.069 |
| meta1071 |  | 396.174 | 225.190 | 0.008 | 0.009 | 0.010 | 0.005 | 0.004 | 0.007 | 0.004 | 0.009 | 0.008 | 0.009 | 0.015 | 0.019 | 0.014 | 0.015 | 0.007 |
| meta1072 | Lys-Trp | 396.195 | 449.119 | 0.015 | 0.013 | 0.015 | 0.015 | 0.010 | 0.019 | 0.011 | 0.010 | 0.014 | 0.012 | 0.014 | 0.014 | 0.014 | 0.011 | 0.014 |
| meta1073 |  | 397.100 | 377.201 | 0.017 | 0.018 | 0.014 | 0.016 | 0.013 | 0.010 | 0.014 | 0.018 | 0.019 | 0.016 | 0.017 | 0.013 | 0.019 | 0.014 | 0.017 |
| meta1074 |  | 397.180 | 204.018 | 0.015 | 0.016 | 0.015 | 0.016 | 0.020 | 0.015 | 0.022 | 0.017 | 0.018 | 0.019 | 0.020 | 0.018 | 0.016 | 0.014 | 0.019 |
| meta1075 |  | 397.377 | 33.665 | 0.003 | 0.002 | 0.003 | 0.004 | 0.004 | 0.002 | 0.005 | 0.004 | 0.004 | 0.003 | 0.003 | 0.003 | 0.003 | 0.004 | 0.006 |
| meta1076 |  | 398.324 | 155.695 | 0.215 | 0.240 | 0.229 | 0.234 | 0.756 | 0.173 | 0.378 | 0.615 | 0.191 | 0.236 | 0.154 | 0.186 | 0.274 | 0.305 | 0.430 |
| meta1077 |  | 399.079 | 280.087 | 0.060 | 0.111 | 0.102 | 0.092 | 0.102 | 0.064 | 0.120 | 0.099 | 0.081 | 0.078 | 0.070 | 0.063 | 0.105 | 0.089 | 0.106 |
| meta1078 |  | 399.125 | 197.061 | 0.014 | 0.015 | 0.016 | 0.013 | 0.017 | 0.012 | 0.018 | 0.017 | 0.012 | 0.013 | 0.013 | 0.015 | 0.014 | 0.011 | 0.015 |
| meta1079 | L-NG-Monomethylarginine | 399.247 | 306.774 | 0.003 | 0.004 | 0.004 | 0.004 | 0.004 | 0.003 | 0.005 | 0.006 | 0.005 | 0.005 | 0.003 | 0.004 | 0.003 | 0.003 | 0.001 |
| meta1080 |  | 399.616 | 480.138 | 0.013 | 0.009 | 0.009 | 0.007 | 0.008 | 0.010 | 0.011 | 0.012 | 0.013 | 0.012 | 0.010 | 0.019 | 0.018 | 0.007 | 0.013 |
| meta1081 |  | 400.026 | 275.462 | 0.024 | 0.017 | 0.014 | 0.019 | 0.020 | 0.020 | 0.016 | 0.024 | 0.015 | 0.019 | 0.015 | 0.014 | 0.020 | 0.017 | 0.018 |
| meta1082 |  | 400.037 | 442.996 | 0.015 | 0.016 | 0.018 | 0.013 | 0.013 | 0.010 | 0.015 | 0.015 | 0.017 | 0.016 | 0.012 | 0.011 | 0.012 | 0.012 | 0.014 |
| meta1083 |  | 400.212 | 44.110 | 0.024 | 0.027 | 0.024 | 0.034 | 0.015 | 0.033 | 0.037 | 0.024 | 0.023 | 0.022 | 0.100 | 0.016 | 0.019 | 0.033 | 0.009 |
| meta1084 | L-Palmitoylcarnitine | 400.341 | 154.809 | 0.766 | 0.880 | 0.846 | 0.848 | 2.292 | 0.589 | 1.891 | 1.508 | 0.986 | 0.599 | 0.625 | 0.526 | 1.093 | 1.207 | 1.127 |
| meta1085 |  | 401.060 | 428.754 | 0.070 | 0.078 | 0.079 | 0.075 | 0.075 | 0.055 | 0.051 | 0.096 | 0.065 | 0.119 | 0.102 | 0.083 | 0.057 | 0.060 | 0.068 |
| meta1086 | Pentamidine | 401.212 | 382.980 | 0.234 | 0.217 | 0.214 | 0.242 | 0.232 | 0.197 | 0.272 | 0.264 | 0.266 | 0.217 | 0.254 | 0.231 | 0.311 | 0.207 | 0.182 |
| meta1087 |  | 401.212 | 368.951 | 0.127 | 0.123 | 0.125 | 0.117 | 0.106 | 0.110 | 0.143 | 0.137 | 0.116 | 0.109 | 0.117 | 0.112 | 0.101 | 0.113 | 0.086 |
| meta1088 |  | 401.248 | 290.721 | 0.016 | 0.017 | 0.013 | 0.013 | 0.014 | 0.009 | 0.010 | 0.012 | 0.008 | 0.017 | 0.017 | 0.017 | 0.011 | 0.012 | 0.014 |
| meta1089 | 7-Oxocholesterol | 401.339 | 32.818 | 0.035 | 0.051 | 0.046 | 0.064 | 0.058 | 0.041 | 0.065 | 0.070 | 0.045 | 0.057 | 0.072 | 0.055 | 0.042 | 0.039 | 0.059 |
| meta1090 |  | 402.008 | 446.493 | 0.018 | 0.017 | 0.017 | 0.011 | 0.012 | 0.014 | 0.012 | 0.017 | 0.022 | 0.032 | 0.026 | 0.024 | 0.022 | 0.022 | 0.023 |
| meta1091 |  | 402.283 | 273.176 | 0.020 | 0.022 | 0.022 | 0.014 | 0.029 | 0.010 | 0.028 | 0.034 | 0.016 | 0.022 | 0.027 | 0.013 | 0.028 | 0.016 | 0.011 |
| meta1092 |  | 402.283 | 221.769 | 0.016 | 0.019 | 0.019 | 0.015 | 0.052 | 0.014 | 0.034 | 0.026 | 0.008 | 0.015 | 0.011 | 0.011 | 0.010 | 0.006 | 0.013 |
| meta1093 |  | 402.318 | 179.803 | 0.003 | 0.005 | 0.004 | 0.003 | 0.006 | 0.002 | 0.004 | 0.005 | 0.004 | 0.004 | 0.005 | 0.004 | 0.004 | 0.003 | 0.003 |
| meta1094 |  | 402.334 | 140.974 | 0.011 | 0.013 | 0.014 | 0.014 | 0.018 | 0.012 | 0.017 | 0.030 | 0.042 | 0.033 | 0.035 | 0.031 | 0.035 | 0.031 | 0.042 |
| meta1095 |  | 402.880 | 318.454 | 0.090 | 0.090 | 0.103 | 0.081 | 0.089 | 0.106 | 0.095 | 0.082 | 0.114 | 0.086 | 0.089 | 0.081 | 0.109 | 0.124 | 0.085 |
| meta1096 |  | 403.087 | 406.219 | 0.028 | 0.039 | 0.027 | 0.025 | 0.027 | 0.024 | 0.027 | 0.031 | 0.032 | 0.040 | 0.033 | 0.027 | 0.030 | 0.020 | 0.036 |
| meta1097 |  | 403.282 | 34.628 | 0.012 | 0.020 | 0.015 | 0.028 | 0.030 | 0.023 | 0.040 | 0.033 | 0.027 | 0.027 | 0.034 | 0.035 | 0.027 | 0.023 | 0.024 |
| meta1098 |  | 404.128 | 362.255 | 0.760 | 0.859 | 0.774 | 0.812 | 0.785 | 0.605 | 0.867 | 0.781 | 0.739 | 0.770 | 0.831 | 0.803 | 0.725 | 0.570 | 0.895 |
| meta1099 |  | 404.140 | 158.663 | 0.582 | 0.565 | 0.521 | 0.453 | 0.731 | 0.397 | 0.541 | 0.560 | 0.484 | 0.526 | 0.565 | 0.483 | 0.543 | 0.429 | 0.577 |
| meta1100 |  | 404.140 | 202.722 | 0.732 | 0.799 | 0.749 | 0.722 | 0.888 | 0.627 | 0.822 | 0.703 | 0.646 | 0.580 | 0.722 | 0.836 | 0.749 | 0.815 | 0.773 |
| meta1101 | Uridine 5'-diphosphate (UDP) | 405.007 | 434.243 | 0.187 | 0.175 | 0.178 | 0.090 | 0.148 | 0.148 | 0.121 | 0.183 | 0.224 | 0.363 | 0.259 | 0.215 | 0.252 | 0.196 | 0.251 |
| meta1102 | His-Trp | 405.159 | 383.530 | 0.042 | 0.042 | 0.045 | 0.044 | 0.042 | 0.054 | 0.032 | 0.034 | 0.022 | 0.036 | 0.048 | 0.043 | 0.048 | 0.035 | 0.056 |
| meta1103 | Arg-Trp | 405.166 | 313.926 | 0.004 | 0.003 | 0.003 | 0.004 | 0.004 | 0.005 | 0.005 | 0.002 | 0.002 | 0.002 | 0.003 | 0.003 | 0.002 | 0.003 | 0.003 |
| meta1104 |  | 405.258 | 318.447 | 0.018 | 0.017 | 0.018 | 0.016 | 0.020 | 0.017 | 0.022 | 0.019 | 0.020 | 0.017 | 0.020 | 0.016 | 0.018 | 0.018 | 0.017 |
| meta1105 |  | 405.284 | 180.806 | 0.006 | 0.006 | 0.005 | 0.002 | 0.008 | 0.002 | 0.008 | 0.004 | 0.010 | 0.007 | 0.003 | 0.006 | 0.009 | 0.005 | 0.006 |
| meta1106 |  | 406.143 | 475.531 | 0.019 | 0.020 | 0.021 | 0.013 | 0.016 | 0.025 | 0.019 | 0.015 | 0.009 | 0.015 | 0.021 | 0.022 | 0.016 | 0.020 | 0.020 |
| meta1107 |  | 406.199 | 44.721 | 0.030 | 0.031 | 0.032 | 0.033 | 0.043 | 0.029 | 0.028 | 0.026 | 0.027 | 0.041 | 0.031 | 0.031 | 0.030 | 0.030 | 0.045 |
| meta1108 |  | 407.074 | 279.992 | 0.129 | 0.184 | 0.171 | 0.176 | 0.151 | 0.136 | 0.205 | 0.175 | 0.186 | 0.152 | 0.169 | 0.163 | 0.166 | 0.168 | 0.173 |
| meta1109 |  | 407.136 | 268.887 | 0.037 | 0.044 | 0.038 | 0.030 | 0.042 | 0.032 | 0.039 | 0.048 | 0.049 | 0.059 | 0.049 | 0.047 | 0.042 | 0.042 | 0.046 |
| meta1110 |  | 407.201 | 168.259 | 0.021 | 0.025 | 0.025 | 0.016 | 0.039 | 0.027 | 0.027 | 0.036 | 0.023 | 0.018 | 0.032 | 0.012 | 0.010 | 0.013 | 0.016 |
| meta1111 | Salicyluric acid | 408.134 | 348.105 | 0.006 | 0.009 | 0.007 | 0.008 | 0.007 | 0.010 | 0.010 | 0.010 | 0.007 | 0.005 | 0.003 | 0.007 | 0.006 | 0.005 | 0.005 |
| meta1112 |  | 408.232 | 428.475 | 0.024 | 0.023 | 0.024 | 0.023 | 0.029 | 0.088 | 0.024 | 0.035 | 0.029 | 0.028 | 0.037 | 0.023 | 0.023 | 0.023 | 0.028 |
| meta1113 |  | 408.321 | 162.520 | 0.006 | 0.006 | 0.005 | 0.006 | 0.009 | 0.005 | 0.008 | 0.009 | 0.011 | 0.006 | 0.005 | 0.004 | 0.006 | 0.006 | 0.006 |
| meta1114 | Cholesterol | 409.340 | 181.248 | 0.001 | 0.001 | 0.001 | 0.002 | 0.003 | 0.001 | 0.004 | 0.009 | 0.003 | 0.001 | 0.002 | 0.001 | 0.002 | 0.001 | 0.001 |
| meta1115 |  | 410.102 | 327.271 | 0.055 | 0.064 | 0.050 | 0.024 | 0.040 | 0.026 | 0.093 | 0.032 | 0.110 | 0.074 | 0.055 | 0.051 | 0.028 | 0.028 | 0.016 |
| meta1116 |  | 410.287 | 181.434 | 0.003 | 0.002 | 0.003 | 0.003 | 0.010 | 0.002 | 0.010 | 0.005 | 0.003 | 0.004 | 0.003 | 0.005 | 0.007 | 0.002 | 0.004 |
| meta1117 |  | 410.324 | 152.917 | 0.010 | 0.012 | 0.009 | 0.009 | 0.023 | 0.005 | 0.014 | 0.023 | 0.016 | 0.015 | 0.020 | 0.013 | 0.012 | 0.007 | 0.018 |
| meta1118 |  | 411.356 | 180.211 | 0.003 | 0.002 | 0.002 | 0.001 | 0.002 | 0.001 | 0.007 | 0.001 | 0.001 | 0.000 | 0.002 | 0.014 | 0.005 | 0.003 | 0.004 |
| meta1119 |  | 412.144 | 233.970 | 0.022 | 0.022 | 0.021 | 0.019 | 0.035 | 0.022 | 0.039 | 0.026 | 0.009 | 0.014 | 0.015 | 0.020 | 0.010 | 0.017 | 0.011 |
| meta1120 |  | 412.207 | 221.785 | 0.052 | 0.042 | 0.042 | 0.043 | 0.029 | 0.045 | 0.033 | 0.033 | 0.036 | 0.039 | 0.027 | 0.031 | 0.044 | 0.031 | 0.029 |
| meta1121 |  | 412.304 | 179.698 | 0.037 | 0.041 | 0.038 | 0.034 | 0.072 | 0.035 | 0.032 | 0.049 | 0.039 | 0.047 | 0.048 | 0.067 | 0.040 | 0.041 | 0.059 |
| meta1122 |  | 412.340 | 151.175 | 0.038 | 0.040 | 0.040 | 0.042 | 0.119 | 0.024 | 0.089 | 0.095 | 0.046 | 0.048 | 0.065 | 0.037 | 0.046 | 0.022 | 0.059 |
| meta1123 |  | 413.077 | 152.578 | 0.020 | 0.023 | 0.016 | 0.021 | 0.024 | 0.016 | 0.025 | 0.020 | 0.018 | 0.016 | 0.017 | 0.017 | 0.020 | 0.020 | 0.023 |
| meta1124 |  | 413.082 | 279.862 | 0.008 | 0.012 | 0.013 | 0.011 | 0.009 | 0.009 | 0.014 | 0.011 | 0.013 | 0.008 | 0.012 | 0.010 | 0.010 | 0.011 | 0.014 |
| meta1125 |  | 413.122 | 300.731 | 0.179 | 0.188 | 0.155 | 0.128 | 0.189 | 0.093 | 0.290 | 0.288 | 0.129 | 0.186 | 0.150 | 0.138 | 0.079 | 0.082 | 0.232 |
| meta1126 |  | 413.171 | 26.662 | 0.013 | 0.011 | 0.015 | 0.010 | 0.006 | 0.010 | 0.016 | 0.008 | 0.011 | 0.009 | 0.011 | 0.019 | 0.015 | 0.023 | 0.017 |
| meta1127 |  | 413.996 | 275.662 | 0.121 | 0.121 | 0.115 | 0.114 | 0.114 | 0.088 | 0.118 | 0.140 | 0.103 | 0.119 | 0.107 | 0.092 | 0.113 | 0.103 | 0.112 |
| meta1128 |  | 414.122 | 158.673 | 0.025 | 0.031 | 0.021 | 0.019 | 0.027 | 0.019 | 0.021 | 0.027 | 0.029 | 0.030 | 0.029 | 0.025 | 0.020 | 0.024 | 0.031 |
| meta1129 |  | 414.319 | 177.927 | 0.107 | 0.099 | 0.102 | 0.123 | 0.202 | 0.118 | 0.114 | 0.186 | 0.060 | 0.116 | 0.098 | 0.137 | 0.111 | 0.108 | 0.135 |
| meta1130 |  | 414.741 | 288.135 | 0.175 | 0.178 | 0.184 | 0.190 | 0.203 | 0.172 | 0.192 | 0.196 | 0.243 | 0.219 | 0.206 | 0.196 | 0.235 | 0.208 | 0.198 |
| meta1131 |  | 415.209 | 35.432 | 0.084 | 0.100 | 0.096 | 0.243 | 0.371 | 0.022 | 0.758 | 0.163 | 0.038 | 0.025 | 0.020 | 0.061 | 0.207 | 0.043 | 0.044 |
| meta1132 |  | 416.064 | 238.939 | 0.005 | 0.005 | 0.004 | 0.004 | 0.005 | 0.004 | 0.006 | 0.005 | 0.003 | 0.005 | 0.005 | 0.005 | 0.004 | 0.003 | 0.005 |
| meta1133 |  | 416.262 | 238.919 | 0.007 | 0.008 | 0.006 | 0.003 | 0.004 | 0.003 | 0.006 | 0.026 | 0.028 | 0.015 | 0.017 | 0.003 | 0.004 | 0.003 | 0.003 |
| meta1134 |  | 416.298 | 211.331 | 0.018 | 0.018 | 0.014 | 0.014 | 0.025 | 0.016 | 0.022 | 0.041 | 0.004 | 0.012 | 0.007 | 0.015 | 0.009 | 0.007 | 0.015 |
| meta1135 |  | 417.034 | 428.820 | 0.016 | 0.017 | 0.018 | 0.018 | 0.017 | 0.013 | 0.013 | 0.020 | 0.015 | 0.029 | 0.022 | 0.019 | 0.015 | 0.014 | 0.016 |
| meta1136 |  | 417.243 | 315.725 | 0.042 | 0.044 | 0.042 | 0.029 | 0.031 | 0.053 | 0.022 | 0.036 | 0.025 | 0.043 | 0.058 | 0.074 | 0.055 | 0.051 | 0.038 |
| meta1137 |  | 417.333 | 35.671 | 0.020 | 0.026 | 0.022 | 0.036 | 0.040 | 0.024 | 0.034 | 0.050 | 0.028 | 0.042 | 0.037 | 0.031 | 0.027 | 0.032 | 0.034 |
| meta1138 |  | 418.173 | 34.097 | 0.013 | 0.019 | 0.013 | 0.015 | 0.018 | 0.015 | 0.017 | 0.016 | 0.017 | 0.009 | 0.012 | 0.013 | 0.015 | 0.021 | 0.016 |
| meta1139 |  | 419.174 | 372.845 | 0.058 | 0.057 | 0.055 | 0.067 | 0.046 | 0.066 | 0.045 | 0.041 | 0.031 | 0.036 | 0.048 | 0.054 | 0.057 | 0.047 | 0.070 |
| meta1140 |  | 420.097 | 362.091 | 0.040 | 0.043 | 0.034 | 0.030 | 0.043 | 0.023 | 0.060 | 0.040 | 0.016 | 0.033 | 0.046 | 0.034 | 0.023 | 0.015 | 0.034 |
| meta1141 |  | 420.160 | 417.393 | 0.101 | 0.082 | 0.077 | 0.109 | 0.079 | 0.072 | 0.112 | 0.084 | 0.056 | 0.102 | 0.118 | 0.092 | 0.078 | 0.050 | 0.154 |
| meta1142 |  | 420.196 | 344.865 | 0.005 | 0.006 | 0.005 | 0.001 | 0.005 | 0.005 | 0.006 | 0.006 | 0.004 | 0.004 | 0.006 | 0.005 | 0.006 | 0.003 | 0.005 |
| meta1143 |  | 421.082 | 434.510 | 0.028 | 0.029 | 0.028 | 0.025 | 0.028 | 0.025 | 0.031 | 0.035 | 0.035 | 0.034 | 0.030 | 0.029 | 0.030 | 0.023 | 0.032 |
| meta1144 |  | 421.233 | 288.221 | 0.039 | 0.041 | 0.042 | 0.041 | 0.040 | 0.032 | 0.040 | 0.041 | 0.043 | 0.042 | 0.046 | 0.044 | 0.036 | 0.041 | 0.041 |
| meta1145 |  | 421.315 | 33.613 | 0.024 | 0.033 | 0.027 | 0.077 | 0.048 | 0.033 | 0.066 | 0.044 | 0.066 | 0.043 | 0.037 | 0.067 | 0.035 | 0.056 | 0.062 |
| meta1146 |  | 422.103 | 348.490 | 0.040 | 0.043 | 0.034 | 0.042 | 0.050 | 0.042 | 0.060 | 0.051 | 0.033 | 0.039 | 0.035 | 0.041 | 0.034 | 0.026 | 0.038 |
| meta1147 |  | 422.125 | 404.639 | 0.190 | 0.209 | 0.158 | 0.184 | 0.178 | 0.129 | 0.235 | 0.290 | 0.099 | 0.127 | 0.152 | 0.160 | 0.134 | 0.092 | 0.151 |
| meta1148 |  | 423.046 | 432.017 | 0.333 | 0.391 | 0.351 | 0.340 | 0.333 | 0.250 | 0.320 | 0.333 | 0.357 | 0.305 | 0.363 | 0.351 | 0.355 | 0.371 | 0.401 |
| meta1149 |  | 423.041 | 153.299 | 0.002 | 0.002 | 0.002 | 0.002 | 0.007 | 0.002 | 0.002 | 0.003 | 0.003 | 0.002 | 0.002 | 0.002 | 0.002 | 0.002 | 0.002 |
| meta1150 |  | 423.052 | 479.186 | 0.060 | 0.067 | 0.059 | 0.058 | 0.067 | 0.051 | 0.057 | 0.056 | 0.065 | 0.059 | 0.060 | 0.066 | 0.070 | 0.074 | 0.066 |
| meta1151 |  | 423.083 | 26.662 | 0.006 | 0.005 | 0.006 | 0.006 | 0.003 | 0.004 | 0.009 | 0.004 | 0.006 | 0.006 | 0.006 | 0.010 | 0.008 | 0.012 | 0.008 |
| meta1152 | Pentosidine | 423.169 | 404.181 | 0.008 | 0.008 | 0.007 | 0.008 | 0.007 | 0.009 | 0.008 | 0.007 | 0.004 | 0.007 | 0.008 | 0.006 | 0.009 | 0.006 | 0.008 |
| meta1153 |  | 424.100 | 473.656 | 0.287 | 0.213 | 0.239 | 0.200 | 0.226 | 0.214 | 0.193 | 0.346 | 0.266 | 0.657 | 0.367 | 0.292 | 0.279 | 0.180 | 0.275 |
| meta1154 |  | 424.118 | 380.615 | 0.032 | 0.031 | 0.027 | 0.030 | 0.028 | 0.022 | 0.023 | 0.061 | 0.043 | 0.053 | 0.032 | 0.025 | 0.028 | 0.026 | 0.049 |
| meta1155 |  | 424.137 | 263.488 | 0.017 | 0.020 | 0.018 | 0.001 | 0.003 | 0.001 | 0.046 | 0.032 | 0.018 | 0.011 | 0.010 | 0.023 | 0.014 | 0.003 | 0.005 |
| meta1156 | Trp-Arg | 424.201 | 438.896 | 0.113 | 0.114 | 0.123 | 0.124 | 0.098 | 0.123 | 0.089 | 0.097 | 0.082 | 0.103 | 0.111 | 0.124 | 0.125 | 0.102 | 0.129 |
| meta1157 |  | 424.341 | 153.369 | 0.513 | 0.581 | 0.553 | 0.696 | 2.168 | 0.507 | 0.785 | 0.976 | 0.707 | 0.691 | 0.517 | 0.703 | 0.731 | 0.669 | 0.906 |
| meta1158 |  | 424.398 | 28.102 | 0.006 | 0.007 | 0.006 | 0.012 | 0.009 | 0.009 | 0.010 | 0.009 | 0.009 | 0.007 | 0.006 | 0.007 | 0.006 | 0.007 | 0.010 |
| meta1159 |  | 424.862 | 318.447 | 0.038 | 0.034 | 0.041 | 0.034 | 0.036 | 0.042 | 0.036 | 0.032 | 0.047 | 0.035 | 0.036 | 0.032 | 0.042 | 0.046 | 0.034 |
| meta1160 | Thiamine pyrophosphate (TPP) | 425.035 | 486.091 | 0.047 | 0.043 | 0.038 | 0.035 | 0.044 | 0.039 | 0.053 | 0.034 | 0.043 | 0.043 | 0.041 | 0.042 | 0.055 | 0.048 | 0.058 |
| meta1161 |  | 426.046 | 417.393 | 1.572 | 1.772 | 1.698 | 1.615 | 1.588 | 1.389 | 1.771 | 1.652 | 1.851 | 1.575 | 1.755 | 1.534 | 1.881 | 1.684 | 1.987 |
| meta1162 | Capecitabine | 426.100 | 473.643 | 0.017 | 0.015 | 0.015 | 0.009 | 0.017 | 0.016 | 0.011 | 0.027 | 0.014 | 0.036 | 0.023 | 0.018 | 0.018 | 0.013 | 0.016 |
| meta1163 |  | 426.140 | 225.029 | 0.011 | 0.010 | 0.011 | 0.007 | 0.013 | 0.012 | 0.012 | 0.012 | 0.011 | 0.012 | 0.008 | 0.014 | 0.012 | 0.008 | 0.005 |
| meta1164 | Cholic acid | 426.319 | 206.632 | 0.093 | 0.099 | 0.094 | 0.006 | 0.022 | 0.025 | 0.450 | 0.014 | 0.050 | 0.127 | 0.035 | 0.079 | 0.101 | 0.175 | 0.044 |
| meta1165 |  | 426.356 | 151.929 | 0.705 | 0.808 | 0.802 | 0.834 | 3.096 | 0.602 | 1.436 | 1.882 | 0.901 | 0.986 | 0.635 | 0.714 | 1.141 | 0.888 | 1.789 |
| meta1166 |  | 427.103 | 467.253 | 0.092 | 0.094 | 0.077 | 0.106 | 0.108 | 0.065 | 0.063 | 0.275 | 0.097 | 0.079 | 0.102 | 0.080 | 0.041 | 0.053 | 0.086 |
| meta1167 |  | 427.137 | 299.875 | 0.279 | 0.308 | 0.260 | 0.268 | 0.451 | 0.194 | 0.396 | 0.341 | 0.138 | 0.228 | 0.307 | 0.195 | 0.213 | 0.153 | 0.173 |
| meta1168 |  | 427.194 | 187.073 | 0.011 | 0.008 | 0.008 | 0.006 | 0.004 | 0.009 | 0.017 | 0.014 | 0.007 | 0.008 | 0.007 | 0.006 | 0.002 | 0.004 | 0.008 |
| meta1169 | Adenosine 5'-diphosphate (ADP) | 428.046 | 417.421 | 0.108 | 0.106 | 0.116 | 0.099 | 0.111 | 0.088 | 0.076 | 0.100 | 0.129 | 0.090 | 0.106 | 0.096 | 0.124 | 0.112 | 0.138 |
| meta1170 |  | 428.246 | 45.984 | 0.008 | 0.012 | 0.011 | 0.017 | 0.013 | 0.009 | 0.013 | 0.017 | 0.019 | 0.010 | 0.007 | 0.012 | 0.013 | 0.011 | 0.009 |
| meta1171 | Stearoylcarnitine | 428.372 | 151.873 | 0.630 | 0.710 | 0.698 | 0.766 | 1.379 | 0.413 | 1.544 | 1.098 | 1.017 | 0.649 | 0.642 | 0.399 | 0.915 | 0.380 | 1.035 |
| meta1172 |  | 428.386 | 44.217 | 0.008 | 0.010 | 0.007 | 0.022 | 0.020 | 0.010 | 0.020 | 0.017 | 0.025 | 0.027 | 0.023 | 0.041 | 0.012 | 0.015 | 0.023 |
| meta1173 |  | 429.051 | 152.603 | 0.006 | 0.005 | 0.004 | 0.006 | 0.006 | 0.004 | 0.006 | 0.006 | 0.005 | 0.005 | 0.005 | 0.004 | 0.006 | 0.005 | 0.006 |
| meta1174 |  | 429.056 | 281.167 | 0.008 | 0.012 | 0.009 | 0.011 | 0.008 | 0.007 | 0.014 | 0.008 | 0.011 | 0.009 | 0.011 | 0.010 | 0.009 | 0.009 | 0.012 |
| meta1175 |  | 430.159 | 427.962 | 0.076 | 0.092 | 0.095 | 0.111 | 0.084 | 0.304 | 0.082 | 0.078 | 0.079 | 0.069 | 0.278 | 0.066 | 0.064 | 0.080 | 0.085 |
| meta1176 |  | 430.366 | 47.927 | 0.181 | 0.233 | 0.196 | 0.193 | 0.231 | 0.169 | 0.238 | 0.267 | 0.395 | 0.241 | 0.300 | 0.343 | 0.278 | 0.273 | 0.459 |
| meta1177 | alpha-Tocopherol (Vitamin E) | 430.377 | 31.044 | 0.397 | 0.327 | 0.217 | 0.093 | 0.125 | 0.098 | 0.126 | 0.219 | 0.131 | 0.002 | 0.279 | 0.205 | 0.080 | 0.074 | 0.105 |
| meta1178 |  | 430.402 | 126.362 | 0.007 | 0.006 | 0.005 | 0.021 | 0.024 | 0.017 | 0.029 | 0.014 | 0.038 | 0.028 | 0.015 | 0.012 | 0.017 | 0.027 | 0.042 |
| meta1179 | Ala-Asp | 431.138 | 324.772 | 0.052 | 0.056 | 0.057 | 0.049 | 0.082 | 0.069 | 0.061 | 0.047 | 0.026 | 0.040 | 0.074 | 0.109 | 0.039 | 0.070 | 0.030 |
| meta1180 |  | 431.248 | 381.310 | 0.028 | 0.028 | 0.026 | 0.020 | 0.024 | 0.019 | 0.028 | 0.032 | 0.022 | 0.026 | 0.035 | 0.033 | 0.043 | 0.039 | 0.024 |
| meta1181 |  | 432.278 | 92.026 | 0.231 | 0.308 | 0.226 | 0.337 | 0.201 | 0.284 | 0.239 | 0.288 | 0.236 | 0.127 | 0.214 | 0.302 | 0.278 | 0.254 | 0.184 |
| meta1182 |  | 432.381 | 100.527 | 0.052 | 0.068 | 0.066 | 0.056 | 0.082 | 0.066 | 0.068 | 0.106 | 0.098 | 0.080 | 0.105 | 0.100 | 0.078 | 0.079 | 0.123 |
| meta1183 |  | 432.381 | 85.729 | 0.056 | 0.038 | 0.044 | 0.032 | 0.054 | 0.040 | 0.040 | 0.059 | 0.055 | 0.054 | 0.072 | 0.076 | 0.053 | 0.044 | 0.055 |
| meta1184 |  | 433.245 | 275.127 | 0.037 | 0.042 | 0.037 | 0.040 | 0.050 | 0.042 | 0.041 | 0.024 | 0.016 | 0.030 | 0.045 | 0.051 | 0.035 | 0.034 | 0.031 |
| meta1185 |  | 434.186 | 342.674 | 0.074 | 0.066 | 0.051 | 0.070 | 0.073 | 0.047 | 0.064 | 0.074 | 0.056 | 0.074 | 0.075 | 0.071 | 0.057 | 0.051 | 0.062 |
| meta1186 | L-Kynurenine | 434.193 | 295.041 | 0.003 | 0.003 | 0.004 | 0.003 | 0.003 | 0.006 | 0.004 | 0.003 | 0.003 | 0.003 | 0.003 | 0.004 | 0.003 | 0.003 | 0.003 |
| meta1187 |  | 434.221 | 390.261 | 0.017 | 0.019 | 0.019 | 0.022 | 0.020 | 0.017 | 0.019 | 0.016 | 0.015 | 0.017 | 0.015 | 0.016 | 0.022 | 0.012 | 0.023 |
| meta1188 |  | 434.234 | 39.302 | 0.051 | 0.052 | 0.050 | 0.067 | 0.127 | 0.063 | 0.110 | 0.102 | 0.083 | 0.105 | 0.041 | 0.071 | 0.035 | 0.063 | 0.138 |
| meta1189 |  | 434.325 | 194.986 | 0.002 | 0.004 | 0.003 | 0.003 | 0.007 | 0.008 | 0.004 | 0.006 | 0.002 | 0.003 | 0.005 | 0.002 | 0.002 | 0.002 | 0.008 |
| meta1190 |  | 434.397 | 163.123 | 0.004 | 0.004 | 0.003 | 0.005 | 0.005 | 0.004 | 0.005 | 0.008 | 0.007 | 0.005 | 0.007 | 0.010 | 0.006 | 0.005 | 0.011 |
| meta1191 |  | 435.055 | 279.715 | 0.007 | 0.008 | 0.008 | 0.008 | 0.006 | 0.006 | 0.011 | 0.008 | 0.005 | 0.003 | 0.006 | 0.008 | 0.008 | 0.005 | 0.008 |
| meta1192 |  | 436.136 | 337.986 | 0.011 | 0.012 | 0.010 | 0.003 | 0.020 | 0.002 | 0.031 | 0.002 | 0.019 | 0.010 | 0.003 | 0.003 | 0.014 | 0.006 | 0.005 |
| meta1193 |  | 436.158 | 198.005 | 0.011 | 0.011 | 0.012 | 0.010 | 0.005 | 0.009 | 0.010 | 0.025 | 0.016 | 0.019 | 0.009 | 0.004 | 0.005 | 0.004 | 0.002 |
| meta1194 |  | 436.237 | 376.119 | 0.019 | 0.018 | 0.017 | 0.041 | 0.023 | 0.018 | 0.017 | 0.014 | 0.009 | 0.023 | 0.014 | 0.013 | 0.020 | 0.018 | 0.023 |
| meta1195 |  | 436.280 | 181.768 | 0.034 | 0.039 | 0.042 | 0.034 | 0.032 | 0.027 | 0.056 | 0.057 | 0.032 | 0.031 | 0.046 | 0.042 | 0.031 | 0.033 | 0.044 |
| meta1196 |  | 437.032 | 224.479 | 0.007 | 0.005 | 0.005 | 0.004 | 0.006 | 0.010 | 0.005 | 0.006 | 0.007 | 0.008 | 0.007 | 0.007 | 0.007 | 0.007 | 0.006 |
| meta1197 |  | 438.217 | 427.990 | 0.028 | 0.028 | 0.032 | 0.040 | 0.022 | 0.057 | 0.037 | 0.011 | 0.016 | 0.019 | 0.022 | 0.020 | 0.030 | 0.037 | 0.028 |
| meta1198 |  | 438.319 | 173.342 | 0.014 | 0.015 | 0.009 | 0.012 | 0.038 | 0.026 | 0.025 | 0.020 | 0.016 | 0.027 | 0.016 | 0.021 | 0.023 | 0.005 | 0.017 |
| meta1199 |  | 438.355 | 149.364 | 0.019 | 0.022 | 0.020 | 0.024 | 0.062 | 0.013 | 0.053 | 0.050 | 0.029 | 0.031 | 0.038 | 0.020 | 0.030 | 0.013 | 0.050 |
| meta1200 |  | 438.785 | 287.075 | 0.031 | 0.027 | 0.023 | 0.025 | 0.033 | 0.024 | 0.024 | 0.031 | 0.031 | 0.030 | 0.031 | 0.031 | 0.033 | 0.024 | 0.029 |
| meta1201 |  | 439.099 | 384.726 | 0.056 | 0.055 | 0.052 | 0.069 | 0.059 | 0.042 | 0.053 | 0.063 | 0.058 | 0.053 | 0.061 | 0.056 | 0.059 | 0.049 | 0.060 |
| meta1202 |  | 439.098 | 275.757 | 0.024 | 0.024 | 0.023 | 0.019 | 0.023 | 0.022 | 0.024 | 0.020 | 0.031 | 0.025 | 0.028 | 0.026 | 0.025 | 0.030 | 0.028 |
| meta1203 |  | 439.140 | 408.318 | 0.046 | 0.046 | 0.045 | 0.038 | 0.041 | 0.042 | 0.043 | 0.073 | 0.048 | 0.053 | 0.040 | 0.060 | 0.029 | 0.027 | 0.042 |
| meta1204 |  | 440.059 | 362.326 | 0.131 | 0.178 | 0.169 | 0.143 | 0.145 | 0.133 | 0.165 | 0.151 | 0.140 | 0.134 | 0.141 | 0.147 | 0.140 | 0.140 | 0.193 |
| meta1205 |  | 440.094 | 478.370 | 0.005 | 0.005 | 0.005 | 0.005 | 0.005 | 0.003 | 0.003 | 0.006 | 0.007 | 0.010 | 0.007 | 0.006 | 0.005 | 0.002 | 0.006 |
| meta1206 |  | 440.182 | 44.944 | 0.006 | 0.011 | 0.007 | 0.008 | 0.010 | 0.012 | 0.007 | 0.006 | 0.007 | 0.007 | 0.008 | 0.006 | 0.008 | 0.008 | 0.009 |
| meta1207 |  | 440.335 | 175.214 | 0.088 | 0.093 | 0.081 | 0.114 | 0.126 | 0.106 | 0.061 | 0.077 | 0.061 | 0.107 | 0.104 | 0.180 | 0.073 | 0.117 | 0.067 |
| meta1208 |  | 440.351 | 34.434 | 0.021 | 0.013 | 0.015 | 0.017 | 0.023 | 0.024 | 0.030 | 0.028 | 0.029 | 0.018 | 0.022 | 0.026 | 0.026 | 0.018 | 0.026 |
| meta1209 |  | 441.135 | 272.996 | 0.048 | 0.044 | 0.031 | 0.033 | 0.041 | 0.017 | 0.039 | 0.052 | 0.093 | 0.080 | 0.055 | 0.052 | 0.053 | 0.038 | 0.054 |
| meta1210 |  | 441.295 | 569.985 | 0.021 | 0.026 | 0.018 | 0.024 | 0.018 | 0.015 | 0.023 | 0.020 | 0.020 | 0.025 | 0.021 | 0.020 | 0.019 | 0.021 | 0.025 |
| meta1211 |  | 442.057 | 164.326 | 0.018 | 0.018 | 0.021 | 0.016 | 0.024 | 0.019 | 0.020 | 0.010 | 0.030 | 0.012 | 0.019 | 0.017 | 0.024 | 0.019 | 0.021 |
| meta1212 |  | 442.117 | 209.911 | 0.007 | 0.007 | 0.007 | 0.006 | 0.013 | 0.005 | 0.009 | 0.007 | 0.007 | 0.007 | 0.006 | 0.007 | 0.008 | 0.007 | 0.007 |
| meta1213 |  | 442.177 | 372.899 | 0.013 | 0.011 | 0.010 | 0.009 | 0.010 | 0.008 | 0.015 | 0.013 | 0.010 | 0.010 | 0.010 | 0.008 | 0.011 | 0.007 | 0.011 |
| meta1214 |  | 442.289 | 41.316 | 0.013 | 0.018 | 0.020 | 0.015 | 0.019 | 0.015 | 0.024 | 0.036 | 0.023 | 0.036 | 0.022 | 0.030 | 0.022 | 0.024 | 0.023 |
| meta1215 |  | 442.350 | 173.342 | 0.155 | 0.163 | 0.140 | 0.200 | 0.295 | 0.194 | 0.156 | 0.232 | 0.092 | 0.209 | 0.200 | 0.272 | 0.194 | 0.193 | 0.172 |
| meta1216 |  | 442.365 | 34.628 | 0.036 | 0.047 | 0.038 | 0.077 | 0.080 | 0.072 | 0.125 | 0.079 | 0.120 | 0.103 | 0.119 | 0.109 | 0.071 | 0.083 | 0.146 |
| meta1217 |  | 444.055 | 164.326 | 0.050 | 0.057 | 0.060 | 0.047 | 0.063 | 0.053 | 0.048 | 0.029 | 0.084 | 0.038 | 0.052 | 0.051 | 0.064 | 0.052 | 0.053 |
| meta1218 |  | 444.066 | 441.025 | 0.012 | 0.013 | 0.015 | 0.012 | 0.014 | 0.012 | 0.013 | 0.014 | 0.013 | 0.011 | 0.011 | 0.011 | 0.014 | 0.012 | 0.012 |
| meta1219 |  | 444.161 | 279.267 | 0.005 | 0.004 | 0.006 | 0.004 | 0.006 | 0.004 | 0.010 | 0.006 | 0.005 | 0.007 | 0.005 | 0.006 | 0.006 | 0.006 | 0.003 |
| meta1220 |  | 444.221 | 145.687 | 0.004 | 0.005 | 0.006 | 0.009 | 0.004 | 0.008 | 0.006 | 0.007 | 0.006 | 0.006 | 0.004 | 0.003 | 0.003 | 0.003 | 0.003 |
| meta1221 |  | 444.365 | 172.739 | 0.073 | 0.091 | 0.084 | 0.069 | 0.138 | 0.058 | 0.130 | 0.129 | 0.079 | 0.098 | 0.100 | 0.056 | 0.075 | 0.061 | 0.089 |
| meta1222 |  | 445.029 | 283.464 | 0.036 | 0.039 | 0.037 | 0.049 | 0.038 | 0.027 | 0.051 | 0.036 | 0.036 | 0.032 | 0.034 | 0.040 | 0.025 | 0.035 | 0.043 |
| meta1223 |  | 445.118 | 32.173 | 0.016 | 0.027 | 0.023 | 0.018 | 0.025 | 0.022 | 0.017 | 0.020 | 0.024 | 0.017 | 0.021 | 0.015 | 0.019 | 0.019 | 0.013 |
| meta1224 | Methyldopa | 445.159 | 355.835 | 0.005 | 0.007 | 0.007 | 0.005 | 0.012 | 0.004 | 0.007 | 0.009 | 0.005 | 0.010 | 0.005 | 0.005 | 0.005 | 0.003 | 0.003 |
| meta1225 |  | 446.081 | 473.643 | 0.031 | 0.026 | 0.025 | 0.022 | 0.027 | 0.020 | 0.023 | 0.031 | 0.023 | 0.072 | 0.043 | 0.030 | 0.028 | 0.017 | 0.032 |
| meta1226 |  | 446.361 | 203.440 | 0.027 | 0.026 | 0.023 | 0.019 | 0.036 | 0.015 | 0.023 | 0.033 | 0.025 | 0.020 | 0.039 | 0.030 | 0.027 | 0.026 | 0.043 |
| meta1227 |  | 446.396 | 150.025 | 0.046 | 0.051 | 0.044 | 0.050 | 0.067 | 0.045 | 0.063 | 0.074 | 0.066 | 0.052 | 0.071 | 0.084 | 0.050 | 0.044 | 0.077 |
| meta1228 |  | 446.396 | 96.121 | 0.064 | 0.098 | 0.080 | 0.064 | 0.095 | 0.083 | 0.076 | 0.123 | 0.143 | 0.115 | 0.116 | 0.152 | 0.122 | 0.115 | 0.158 |
| meta1229 |  | 446.396 | 76.734 | 0.072 | 0.063 | 0.067 | 0.063 | 0.081 | 0.060 | 0.070 | 0.108 | 0.123 | 0.119 | 0.132 | 0.138 | 0.101 | 0.095 | 0.126 |
| meta1230 |  | 446.844 | 318.454 | 0.029 | 0.027 | 0.033 | 0.027 | 0.028 | 0.032 | 0.027 | 0.026 | 0.035 | 0.027 | 0.028 | 0.026 | 0.033 | 0.037 | 0.026 |
| meta1231 | CDP-ethanolamine | 447.065 | 432.045 | 0.057 | 0.053 | 0.053 | 0.037 | 0.054 | 0.046 | 0.059 | 0.055 | 0.049 | 0.053 | 0.044 | 0.045 | 0.065 | 0.039 | 0.058 |
| meta1232 |  | 447.107 | 463.390 | 0.029 | 0.030 | 0.029 | 0.022 | 0.032 | 0.030 | 0.034 | 0.028 | 0.026 | 0.026 | 0.031 | 0.038 | 0.035 | 0.028 | 0.032 |
| meta1233 |  | 447.242 | 339.237 | 0.105 | 0.095 | 0.084 | 0.021 | 0.048 | 0.017 | 0.179 | 0.187 | 0.132 | 0.065 | 0.059 | 0.156 | 0.121 | 0.031 | 0.056 |
| meta1234 |  | 447.261 | 48.403 | 0.050 | 0.059 | 0.055 | 0.172 | 0.098 | 0.003 | 0.037 | 0.003 | 0.041 | 0.023 | 0.024 | 0.006 | 0.008 | 0.006 | 0.005 |
| meta1235 |  | 447.307 | 46.472 | 0.088 | 0.103 | 0.091 | 0.009 | 0.138 | 0.101 | 0.083 | 0.011 | 0.090 | 0.012 | 0.011 | 0.016 | 0.010 | 0.013 | 0.013 |
| meta1236 |  | 448.027 | 415.468 | 0.007 | 0.007 | 0.007 | 0.011 | 0.011 | 0.007 | 0.007 | 0.008 | 0.010 | 0.011 | 0.010 | 0.009 | 0.012 | 0.009 | 0.009 |
| meta1237 |  | 448.131 | 304.321 | 0.016 | 0.017 | 0.016 | 0.014 | 0.019 | 0.014 | 0.017 | 0.020 | 0.020 | 0.019 | 0.016 | 0.016 | 0.016 | 0.015 | 0.015 |
| meta1238 |  | 448.140 | 360.181 | 0.033 | 0.032 | 0.046 | 0.043 | 0.026 | 0.030 | 0.034 | 0.037 | 0.050 | 0.026 | 0.022 | 0.029 | 0.032 | 0.039 | 0.029 |
| meta1239 |  | 448.340 | 149.884 | 0.206 | 0.223 | 0.230 | 0.316 | 0.739 | 0.366 | 0.390 | 0.395 | 0.301 | 0.269 | 0.322 | 0.290 | 0.215 | 0.239 | 0.309 |
| meta1240 |  | 448.376 | 130.912 | 0.049 | 0.035 | 0.046 | 0.047 | 0.069 | 0.051 | 0.059 | 0.086 | 0.094 | 0.069 | 0.063 | 0.067 | 0.086 | 0.065 | 0.106 |
| meta1241 |  | 448.375 | 182.673 | 0.014 | 0.012 | 0.012 | 0.013 | 0.017 | 0.007 | 0.010 | 0.020 | 0.018 | 0.012 | 0.017 | 0.025 | 0.012 | 0.011 | 0.024 |
| meta1242 |  | 448.413 | 157.032 | 0.015 | 0.015 | 0.015 | 0.011 | 0.020 | 0.014 | 0.019 | 0.025 | 0.026 | 0.029 | 0.028 | 0.033 | 0.018 | 0.018 | 0.029 |
| meta1243 |  | 449.285 | 319.049 | 0.025 | 0.023 | 0.026 | 0.022 | 0.026 | 0.023 | 0.023 | 0.023 | 0.027 | 0.024 | 0.026 | 0.021 | 0.027 | 0.027 | 0.028 |
| meta1244 | Adenosine 3',5'-diphosphate (PAP) | 450.016 | 484.226 | 0.027 | 0.018 | 0.019 | 0.023 | 0.024 | 0.023 | 0.023 | 0.027 | 0.020 | 0.011 | 0.020 | 0.024 | 0.021 | 0.025 | 0.031 |
| meta1245 | Pantoprazole | 450.025 | 224.716 | 0.005 | 0.006 | 0.005 | 0.005 | 0.005 | 0.007 | 0.005 | 0.006 | 0.009 | 0.007 | 0.006 | 0.006 | 0.005 | 0.007 | 0.007 |
| meta1246 |  | 450.082 | 417.421 | 0.134 | 0.141 | 0.143 | 0.118 | 0.129 | 0.116 | 0.129 | 0.156 | 0.146 | 0.138 | 0.141 | 0.121 | 0.143 | 0.130 | 0.149 |
| meta1247 |  | 450.125 | 386.368 | 0.222 | 0.175 | 0.174 | 0.150 | 0.188 | 0.163 | 0.109 | 0.281 | 0.103 | 0.180 | 0.147 | 0.094 | 0.142 | 0.154 | 0.157 |
| meta1248 |  | 450.156 | 428.116 | 0.018 | 0.025 | 0.024 | 0.032 | 0.021 | 0.023 | 0.020 | 0.018 | 0.022 | 0.020 | 0.021 | 0.019 | 0.017 | 0.022 | 0.024 |
| meta1249 |  | 450.253 | 355.274 | 0.010 | 0.009 | 0.010 | 0.019 | 0.008 | 0.011 | 0.009 | 0.011 | 0.009 | 0.006 | 0.008 | 0.008 | 0.011 | 0.009 | 0.013 |
| meta1250 |  | 450.264 | 163.120 | 0.013 | 0.013 | 0.013 | 0.002 | 0.003 | 0.013 | 0.015 | 0.024 | 0.005 | 0.025 | 0.014 | 0.013 | 0.020 | 0.026 | 0.011 |
| meta1251 |  | 450.392 | 190.615 | 0.016 | 0.017 | 0.018 | 0.011 | 0.015 | 0.013 | 0.018 | 0.025 | 0.021 | 0.023 | 0.028 | 0.026 | 0.023 | 0.019 | 0.025 |
| meta1252 |  | 451.212 | 447.001 | 0.029 | 0.027 | 0.029 | 0.027 | 0.022 | 0.034 | 0.024 | 0.026 | 0.019 | 0.025 | 0.027 | 0.029 | 0.030 | 0.025 | 0.031 |
| meta1253 | Tyr-Arg | 452.042 | 417.996 | 0.110 | 0.109 | 0.113 | 0.099 | 0.094 | 0.089 | 0.110 | 0.133 | 0.122 | 0.101 | 0.119 | 0.109 | 0.114 | 0.118 | 0.124 |
| meta1254 |  | 452.218 | 141.266 | 0.055 | 0.059 | 0.060 | 0.042 | 0.056 | 0.041 | 0.051 | 0.049 | 0.028 | 0.054 | 0.072 | 0.061 | 0.053 | 0.052 | 0.060 |
| meta1255 |  | 452.275 | 182.979 | 0.051 | 0.053 | 0.058 | 0.056 | 0.068 | 0.049 | 0.093 | 0.073 | 0.031 | 0.049 | 0.048 | 0.053 | 0.033 | 0.048 | 0.057 |
| meta1256 |  | 452.392 | 34.670 | 0.006 | 0.008 | 0.009 | 0.008 | 0.016 | 0.010 | 0.021 | 0.009 | 0.010 | 0.014 | 0.009 | 0.007 | 0.005 | 0.010 | 0.007 |
| meta1257 |  | 452.432 | 28.010 | 0.003 | 0.003 | 0.003 | 0.006 | 0.005 | 0.005 | 0.005 | 0.007 | 0.005 | 0.007 | 0.003 | 0.003 | 0.003 | 0.005 | 0.006 |
| meta1258 |  | 453.022 | 280.659 | 0.003 | 0.003 | 0.002 | 0.004 | 0.004 | 0.002 | 0.003 | 0.004 | 0.004 | 0.004 | 0.004 | 0.003 | 0.003 | 0.003 | 0.003 |
| meta1259 |  | 453.067 | 277.087 | 0.021 | 0.016 | 0.020 | 0.019 | 0.018 | 0.015 | 0.022 | 0.023 | 0.013 | 0.013 | 0.016 | 0.015 | 0.017 | 0.011 | 0.018 |
| meta1260 |  | 453.064 | 304.832 | 0.011 | 0.008 | 0.009 | 0.006 | 0.008 | 0.006 | 0.008 | 0.010 | 0.012 | 0.011 | 0.011 | 0.007 | 0.008 | 0.009 | 0.010 |
| meta1261 |  | 453.099 | 372.356 | 0.033 | 0.031 | 0.027 | 0.033 | 0.034 | 0.020 | 0.044 | 0.035 | 0.018 | 0.033 | 0.046 | 0.023 | 0.021 | 0.016 | 0.039 |
| meta1262 |  | 453.120 | 475.017 | 0.017 | 0.016 | 0.019 | 0.015 | 0.016 | 0.012 | 0.022 | 0.018 | 0.020 | 0.016 | 0.017 | 0.017 | 0.017 | 0.017 | 0.016 |
| meta1263 |  | 453.126 | 387.548 | 0.019 | 0.013 | 0.014 | 0.013 | 0.013 | 0.010 | 0.012 | 0.016 | 0.011 | 0.012 | 0.013 | 0.008 | 0.011 | 0.011 | 0.014 |
| meta1264 | Atrazine | 453.177 | 212.811 | 0.005 | 0.003 | 0.004 | 0.003 | 0.004 | 0.004 | 0.003 | 0.003 | 0.002 | 0.003 | 0.003 | 0.003 | 0.002 | 0.003 | 0.004 |
| meta1265 |  | 454.124 | 323.332 | 0.039 | 0.042 | 0.034 | 0.028 | 0.056 | 0.033 | 0.043 | 0.035 | 0.019 | 0.032 | 0.048 | 0.071 | 0.030 | 0.037 | 0.023 |
| meta1266 | 1-Palmitoyl-2-hydroxy-sn-glycero-3-phosphoethanolamine | 454.292 | 181.822 | 0.875 | 0.968 | 0.922 | 0.895 | 0.884 | 0.649 | 1.380 | 1.513 | 0.785 | 0.834 | 1.244 | 1.112 | 0.771 | 0.853 | 1.212 |
| meta1267 |  | 454.350 | 206.545 | 0.003 | 0.004 | 0.003 | 0.001 | 0.001 | 0.001 | 0.014 | 0.003 | 0.002 | 0.004 | 0.001 | 0.002 | 0.003 | 0.005 | 0.002 |
| meta1268 |  | 454.386 | 149.224 | 0.133 | 0.151 | 0.146 | 0.118 | 0.387 | 0.056 | 0.444 | 0.321 | 0.219 | 0.120 | 0.186 | 0.084 | 0.304 | 0.091 | 0.492 |
| meta1269 |  | 456.216 | 38.975 | 0.011 | 0.015 | 0.011 | 0.011 | 0.018 | 0.011 | 0.014 | 0.018 | 0.020 | 0.016 | 0.011 | 0.015 | 0.009 | 0.013 | 0.023 |
| meta1270 |  | 456.329 | 240.994 | 0.023 | 0.024 | 0.018 | 0.013 | 0.029 | 0.010 | 0.036 | 0.033 | 0.013 | 0.017 | 0.027 | 0.015 | 0.019 | 0.018 | 0.012 |
| meta1271 |  | 457.059 | 435.048 | 0.006 | 0.006 | 0.006 | 0.005 | 0.005 | 0.004 | 0.003 | 0.005 | 0.007 | 0.011 | 0.010 | 0.008 | 0.007 | 0.008 | 0.007 |
| meta1272 | Flavin mononucleotide (FMN) | 457.109 | 395.895 | 0.013 | 0.014 | 0.013 | 0.012 | 0.012 | 0.010 | 0.014 | 0.013 | 0.014 | 0.011 | 0.010 | 0.013 | 0.014 | 0.011 | 0.016 |
| meta1273 |  | 458.175 | 430.737 | 0.020 | 0.027 | 0.020 | 0.016 | 0.019 | 0.017 | 0.021 | 0.015 | 0.008 | 0.015 | 0.016 | 0.032 | 0.015 | 0.022 | 0.026 |
| meta1274 |  | 458.344 | 235.290 | 0.010 | 0.008 | 0.006 | 0.009 | 0.012 | 0.007 | 0.015 | 0.018 | 0.010 | 0.010 | 0.013 | 0.006 | 0.009 | 0.004 | 0.004 |
| meta1275 |  | 458.360 | 47.221 | 0.058 | 0.079 | 0.052 | 0.048 | 0.062 | 0.046 | 0.072 | 0.079 | 0.071 | 0.064 | 0.083 | 0.143 | 0.062 | 0.100 | 0.139 |
| meta1276 |  | 458.360 | 143.511 | 0.015 | 0.013 | 0.017 | 0.017 | 0.022 | 0.011 | 0.024 | 0.028 | 0.029 | 0.022 | 0.028 | 0.036 | 0.032 | 0.021 | 0.031 |
| meta1277 |  | 459.099 | 279.877 | 0.033 | 0.041 | 0.038 | 0.040 | 0.037 | 0.034 | 0.047 | 0.041 | 0.035 | 0.031 | 0.036 | 0.036 | 0.034 | 0.035 | 0.035 |
| meta1278 |  | 460.249 | 164.760 | 0.011 | 0.007 | 0.009 | 0.002 | 0.002 | 0.007 | 0.027 | 0.008 | 0.005 | 0.007 | 0.019 | 0.015 | 0.014 | 0.018 | 0.004 |
| meta1279 |  | 460.267 | 35.677 | 0.019 | 0.029 | 0.024 | 0.049 | 0.073 | 0.023 | 0.144 | 0.032 | 0.009 | 0.021 | 0.022 | 0.015 | 0.047 | 0.014 | 0.015 |
| meta1280 |  | 460.376 | 190.013 | 0.027 | 0.027 | 0.024 | 0.018 | 0.035 | 0.016 | 0.021 | 0.035 | 0.027 | 0.017 | 0.042 | 0.033 | 0.028 | 0.027 | 0.030 |
| meta1281 |  | 460.989 | 285.836 | 0.004 | 0.003 | 0.003 | 0.004 | 0.003 | 0.002 | 0.004 | 0.004 | 0.002 | 0.003 | 0.004 | 0.005 | 0.002 | 0.004 | 0.003 |
| meta1282 |  | 461.077 | 408.408 | 0.015 | 0.017 | 0.015 | 0.013 | 0.014 | 0.011 | 0.017 | 0.015 | 0.019 | 0.019 | 0.015 | 0.013 | 0.015 | 0.012 | 0.016 |
| meta1283 |  | 461.079 | 482.158 | 0.017 | 0.016 | 0.015 | 0.014 | 0.017 | 0.013 | 0.013 | 0.014 | 0.013 | 0.012 | 0.012 | 0.016 | 0.023 | 0.019 | 0.018 |
| meta1284 |  | 461.251 | 260.863 | 0.009 | 0.006 | 0.008 | 0.004 | 0.006 | 0.007 | 0.010 | 0.006 | 0.004 | 0.014 | 0.010 | 0.013 | 0.012 | 0.010 | 0.005 |
| meta1285 |  | 462.170 | 408.937 | 0.014 | 0.015 | 0.018 | 0.015 | 0.016 | 0.018 | 0.018 | 0.015 | 0.010 | 0.017 | 0.016 | 0.012 | 0.011 | 0.010 | 0.012 |
| meta1286 |  | 462.265 | 184.269 | 1.679 | 1.672 | 1.562 | 0.284 | 0.599 | 2.305 | 2.865 | 1.555 | 0.468 | 2.682 | 1.512 | 0.547 | 2.335 | 2.534 | 0.792 |
| meta1287 |  | 462.265 | 163.117 | 0.321 | 0.343 | 0.352 | 0.053 | 0.100 | 0.309 | 0.403 | 0.598 | 0.145 | 0.685 | 0.325 | 0.230 | 0.472 | 0.668 | 0.283 |
| meta1288 |  | 462.295 | 178.993 | 0.008 | 0.009 | 0.010 | 0.008 | 0.009 | 0.006 | 0.014 | 0.016 | 0.008 | 0.009 | 0.013 | 0.014 | 0.008 | 0.012 | 0.011 |
| meta1289 |  | 462.391 | 225.000 | 0.016 | 0.019 | 0.017 | 0.012 | 0.020 | 0.012 | 0.017 | 0.022 | 0.020 | 0.019 | 0.024 | 0.024 | 0.021 | 0.016 | 0.023 |
| meta1290 |  | 462.390 | 45.536 | 0.008 | 0.010 | 0.013 | 0.007 | 0.018 | 0.007 | 0.019 | 0.013 | 0.019 | 0.014 | 0.008 | 0.023 | 0.007 | 0.015 | 0.009 |
| meta1291 |  | 462.391 | 135.298 | 0.012 | 0.018 | 0.014 | 0.014 | 0.018 | 0.017 | 0.020 | 0.019 | 0.031 | 0.024 | 0.021 | 0.017 | 0.027 | 0.024 | 0.030 |
| meta1292 |  | 463.033 | 279.242 | 0.007 | 0.007 | 0.006 | 0.001 | 0.015 | 0.001 | 0.017 | 0.001 | 0.006 | 0.004 | 0.001 | 0.001 | 0.007 | 0.002 | 0.002 |
| meta1293 |  | 463.092 | 413.328 | 0.024 | 0.021 | 0.015 | 0.018 | 0.013 | 0.019 | 0.007 | 0.025 | 0.019 | 0.032 | 0.028 | 0.016 | 0.022 | 0.024 | 0.029 |
| meta1294 | Adenylsuccinic acid | 464.078 | 478.798 | 0.105 | 0.091 | 0.091 | 0.090 | 0.118 | 0.099 | 0.086 | 0.062 | 0.089 | 0.082 | 0.073 | 0.107 | 0.147 | 0.106 | 0.120 |
| meta1295 | CDP-Ethanolamine | 464.097 | 413.305 | 0.013 | 0.009 | 0.010 | 0.010 | 0.012 | 0.011 | 0.011 | 0.013 | 0.010 | 0.012 | 0.010 | 0.008 | 0.010 | 0.011 | 0.011 |
| meta1296 |  | 464.280 | 137.975 | 0.387 | 0.402 | 0.371 | 0.180 | 0.227 | 0.392 | 0.687 | 0.450 | 0.191 | 0.757 | 0.577 | 0.298 | 0.502 | 0.570 | 0.367 |
| meta1297 |  | 464.281 | 121.407 | 0.037 | 0.048 | 0.036 | 0.036 | 0.039 | 0.052 | 0.112 | 0.038 | 0.014 | 0.071 | 0.066 | 0.027 | 0.083 | 0.092 | 0.081 |
| meta1298 |  | 464.334 | 170.332 | 0.009 | 0.010 | 0.006 | 0.008 | 0.016 | 0.006 | 0.009 | 0.009 | 0.012 | 0.007 | 0.013 | 0.010 | 0.008 | 0.010 | 0.013 |
| meta1299 |  | 464.370 | 147.839 | 0.019 | 0.026 | 0.021 | 0.030 | 0.058 | 0.018 | 0.045 | 0.033 | 0.036 | 0.020 | 0.038 | 0.030 | 0.031 | 0.023 | 0.037 |
| meta1300 |  | 464.880 | 318.454 | 0.045 | 0.043 | 0.051 | 0.040 | 0.044 | 0.051 | 0.046 | 0.041 | 0.057 | 0.042 | 0.044 | 0.040 | 0.055 | 0.060 | 0.042 |
| meta1301 |  | 465.092 | 431.589 | 0.120 | 0.143 | 0.130 | 0.125 | 0.117 | 0.096 | 0.118 | 0.130 | 0.129 | 0.120 | 0.126 | 0.116 | 0.123 | 0.137 | 0.146 |
| meta1302 |  | 465.126 | 469.163 | 0.020 | 0.014 | 0.016 | 0.014 | 0.020 | 0.014 | 0.015 | 0.019 | 0.021 | 0.036 | 0.023 | 0.026 | 0.023 | 0.020 | 0.021 |
| meta1303 |  | 465.195 | 304.586 | 0.006 | 0.006 | 0.005 | 0.007 | 0.005 | 0.006 | 0.006 | 0.005 | 0.003 | 0.004 | 0.003 | 0.005 | 0.005 | 0.004 | 0.005 |
| meta1304 |  | 465.259 | 288.046 | 0.091 | 0.094 | 0.094 | 0.104 | 0.107 | 0.084 | 0.100 | 0.100 | 0.101 | 0.101 | 0.096 | 0.097 | 0.101 | 0.095 | 0.100 |
| meta1305 |  | 466.212 | 458.224 | 0.032 | 0.030 | 0.032 | 0.032 | 0.022 | 0.037 | 0.032 | 0.023 | 0.025 | 0.034 | 0.031 | 0.038 | 0.031 | 0.025 | 0.030 |
| meta1306 |  | 466.326 | 168.879 | 0.007 | 0.008 | 0.007 | 0.007 | 0.007 | 0.006 | 0.005 | 0.005 | 0.007 | 0.007 | 0.010 | 0.010 | 0.009 | 0.005 | 0.007 |
| meta1307 | Cytidine 5'-diphosphate (CDP) | 467.044 | 414.683 | 0.096 | 0.091 | 0.075 | 0.084 | 0.086 | 0.060 | 0.078 | 0.088 | 0.065 | 0.067 | 0.086 | 0.094 | 0.073 | 0.070 | 0.075 |
| meta1308 |  | 467.275 | 144.626 | 0.010 | 0.011 | 0.012 | 0.012 | 0.011 | 0.015 | 0.025 | 0.015 | 0.012 | 0.018 | 0.017 | 0.013 | 0.012 | 0.015 | 0.013 |
| meta1309 |  | 468.092 | 417.388 | 0.497 | 0.557 | 0.559 | 0.510 | 0.449 | 0.432 | 0.552 | 0.630 | 0.621 | 0.504 | 0.512 | 0.516 | 0.590 | 0.506 | 0.612 |
| meta1310 |  | 468.163 | 205.381 | 0.015 | 0.015 | 0.016 | 0.010 | 0.015 | 0.009 | 0.014 | 0.017 | 0.022 | 0.018 | 0.018 | 0.016 | 0.019 | 0.018 | 0.019 |
| meta1311 |  | 468.238 | 45.712 | 0.005 | 0.006 | 0.007 | 0.005 | 0.009 | 0.005 | 0.008 | 0.007 | 0.008 | 0.007 | 0.005 | 0.005 | 0.003 | 0.004 | 0.006 |
| meta1312 |  | 468.233 | 167.674 | 0.004 | 0.007 | 0.006 | 0.009 | 0.009 | 0.006 | 0.009 | 0.012 | 0.006 | 0.007 | 0.005 | 0.006 | 0.005 | 0.006 | 0.006 |
| meta1313 | 1-Myristoyl-sn-glycero-3-phosphocholine | 468.306 | 179.012 | 0.094 | 0.110 | 0.104 | 0.105 | 0.138 | 0.092 | 0.182 | 0.171 | 0.071 | 0.081 | 0.109 | 0.112 | 0.083 | 0.096 | 0.092 |
| meta1314 |  | 468.366 | 171.030 | 0.010 | 0.007 | 0.006 | 0.004 | 0.027 | 0.008 | 0.016 | 0.011 | 0.012 | 0.008 | 0.016 | 0.006 | 0.028 | 0.008 | 0.020 |
| meta1315 |  | 468.386 | 36.451 | 0.040 | 0.032 | 0.037 | 0.040 | 0.047 | 0.036 | 0.093 | 0.038 | 0.031 | 0.046 | 0.031 | 0.025 | 0.029 | 0.027 | 0.034 |
| meta1316 |  | 468.428 | 29.103 | 0.003 | 0.007 | 0.005 | 0.007 | 0.005 | 0.037 | 0.004 | 0.007 | 0.007 | 0.005 | 0.007 | 0.008 | 0.004 | 0.005 | 0.008 |
| meta1317 |  | 469.045 | 277.153 | 0.046 | 0.054 | 0.053 | 0.055 | 0.055 | 0.042 | 0.062 | 0.058 | 0.046 | 0.043 | 0.049 | 0.046 | 0.051 | 0.041 | 0.051 |
| meta1318 |  | 469.094 | 323.972 | 0.089 | 0.094 | 0.095 | 0.078 | 0.158 | 0.076 | 0.101 | 0.081 | 0.054 | 0.084 | 0.124 | 0.157 | 0.070 | 0.090 | 0.055 |
| meta1319 |  | 469.187 | 348.582 | 0.008 | 0.009 | 0.009 | 0.009 | 0.009 | 0.009 | 0.008 | 0.009 | 0.010 | 0.010 | 0.010 | 0.008 | 0.009 | 0.010 | 0.011 |
| meta1320 |  | 469.294 | 46.224 | 0.051 | 0.068 | 0.064 | 0.228 | 0.125 | 0.081 | 0.041 | 0.024 | 0.040 | 0.019 | 0.021 | 0.032 | 0.022 | 0.025 | 0.016 |
| meta1321 |  | 470.045 | 398.179 | 0.417 | 0.373 | 0.401 | 0.336 | 0.365 | 0.296 | 0.398 | 0.454 | 0.387 | 0.387 | 0.429 | 0.437 | 0.446 | 0.330 | 0.384 |
| meta1322 |  | 470.141 | 479.046 | 0.173 | 0.344 | 0.211 | 0.336 | 0.243 | 0.350 | 0.472 | 0.232 | 0.261 | 0.284 | 0.207 | 0.177 | 0.315 | 0.209 | 0.224 |
| meta1323 |  | 470.382 | 170.184 | 0.024 | 0.032 | 0.030 | 0.013 | 0.063 | 0.013 | 0.065 | 0.052 | 0.026 | 0.027 | 0.033 | 0.019 | 0.066 | 0.019 | 0.057 |
| meta1324 |  | 472.107 | 386.374 | 0.035 | 0.032 | 0.029 | 0.032 | 0.042 | 0.032 | 0.024 | 0.050 | 0.021 | 0.035 | 0.031 | 0.018 | 0.027 | 0.028 | 0.028 |
| meta1325 |  | 472.215 | 140.299 | 0.017 | 0.025 | 0.018 | 0.011 | 0.003 | 0.012 | 0.016 | 0.022 | 0.027 | 0.011 | 0.028 | 0.143 | 0.011 | 0.057 | 0.044 |
| meta1326 |  | 472.285 | 305.104 | 0.005 | 0.005 | 0.005 | 0.004 | 0.005 | 0.004 | 0.004 | 0.006 | 0.004 | 0.006 | 0.007 | 0.008 | 0.006 | 0.005 | 0.008 |
| meta1327 |  | 472.781 | 287.595 | 0.013 | 0.012 | 0.013 | 0.015 | 0.015 | 0.014 | 0.016 | 0.017 | 0.021 | 0.020 | 0.016 | 0.016 | 0.025 | 0.018 | 0.015 |
| meta1328 |  | 473.174 | 401.147 | 0.046 | 0.038 | 0.050 | 0.040 | 0.034 | 0.040 | 0.016 | 0.129 | 0.072 | 0.021 | 0.057 | 0.031 | 0.029 | 0.038 | 0.021 |
| meta1329 |  | 473.240 | 348.365 | 0.011 | 0.011 | 0.011 | 0.012 | 0.012 | 0.012 | 0.013 | 0.012 | 0.010 | 0.011 | 0.012 | 0.013 | 0.011 | 0.011 | 0.012 |
| meta1330 |  | 473.996 | 26.822 | 0.048 | 0.048 | 0.046 | 0.048 | 0.051 | 0.031 | 0.042 | 0.045 | 0.044 | 0.045 | 0.043 | 0.048 | 0.049 | 0.044 | 0.047 |
| meta1331 | Folinic acid | 474.167 | 362.081 | 0.012 | 0.011 | 0.010 | 0.016 | 0.009 | 0.003 | 0.015 | 0.010 | 0.010 | 0.012 | 0.011 | 0.011 | 0.012 | 0.013 | 0.017 |
| meta1332 |  | 474.355 | 46.391 | 0.056 | 0.065 | 0.061 | 0.041 | 0.073 | 0.040 | 0.076 | 0.083 | 0.085 | 0.068 | 0.084 | 0.098 | 0.070 | 0.064 | 0.075 |
| meta1333 |  | 474.354 | 148.982 | 0.010 | 0.010 | 0.011 | 0.013 | 0.029 | 0.012 | 0.022 | 0.019 | 0.018 | 0.015 | 0.017 | 0.013 | 0.013 | 0.016 | 0.029 |
| meta1334 |  | 474.891 | 577.308 | 0.017 | 0.022 | 0.024 | 0.029 | 0.025 | 0.021 | 0.024 | 0.024 | 0.025 | 0.024 | 0.030 | 0.027 | 0.031 | 0.025 | 0.023 |
| meta1335 |  | 475.080 | 372.341 | 0.017 | 0.017 | 0.015 | 0.019 | 0.020 | 0.010 | 0.023 | 0.020 | 0.012 | 0.018 | 0.022 | 0.013 | 0.012 | 0.010 | 0.023 |
| meta1336 |  | 475.093 | 434.587 | 0.016 | 0.018 | 0.019 | 0.017 | 0.016 | 0.017 | 0.018 | 0.020 | 0.023 | 0.020 | 0.018 | 0.017 | 0.019 | 0.016 | 0.019 |
| meta1337 |  | 475.137 | 35.671 | 0.024 | 0.024 | 0.016 | 0.019 | 0.023 | 0.015 | 0.017 | 0.020 | 0.018 | 0.022 | 0.027 | 0.019 | 0.018 | 0.016 | 0.020 |
| meta1338 |  | 475.337 | 26.574 | 0.016 | 0.011 | 0.010 | 0.023 | 0.037 | 0.022 | 0.046 | 0.021 | 0.020 | 0.028 | 0.026 | 0.031 | 0.014 | 0.018 | 0.036 |
| meta1339 |  | 476.226 | 338.543 | 0.024 | 0.020 | 0.018 | 0.011 | 0.029 | 0.013 | 0.034 | 0.022 | 0.018 | 0.017 | 0.019 | 0.018 | 0.036 | 0.025 | 0.029 |
| meta1340 |  | 476.243 | 45.517 | 0.008 | 0.009 | 0.013 | 0.011 | 0.014 | 0.011 | 0.019 | 0.009 | 0.014 | 0.008 | 0.012 | 0.014 | 0.014 | 0.014 | 0.015 |
| meta1341 |  | 476.304 | 96.767 | 0.222 | 0.192 | 0.246 | 0.207 | 0.199 | 0.194 | 0.162 | 0.222 | 0.158 | 0.143 | 0.127 | 0.193 | 0.174 | 0.214 | 0.129 |
| meta1342 |  | 476.741 | 288.135 | 0.134 | 0.139 | 0.146 | 0.149 | 0.159 | 0.134 | 0.142 | 0.153 | 0.190 | 0.157 | 0.158 | 0.153 | 0.187 | 0.161 | 0.156 |
| meta1343 |  | 478.188 | 205.981 | 0.007 | 0.013 | 0.009 | 0.007 | 0.009 | 0.009 | 0.006 | 0.008 | 0.008 | 0.007 | 0.011 | 0.020 | 0.010 | 0.007 | 0.007 |
| meta1344 |  | 478.259 | 164.408 | 0.024 | 0.034 | 0.022 | 0.013 | 0.024 | 0.024 | 0.067 | 0.039 | 0.010 | 0.029 | 0.034 | 0.030 | 0.029 | 0.046 | 0.013 |
| meta1345 |  | 478.259 | 46.342 | 0.021 | 0.022 | 0.021 | 0.023 | 0.025 | 0.023 | 0.032 | 0.023 | 0.031 | 0.032 | 0.021 | 0.025 | 0.026 | 0.027 | 0.025 |
| meta1346 |  | 478.327 | 174.647 | 0.169 | 0.183 | 0.179 | 0.195 | 0.203 | 0.149 | 0.243 | 0.225 | 0.147 | 0.142 | 0.175 | 0.195 | 0.176 | 0.155 | 0.190 |
| meta1347 |  | 478.386 | 148.945 | 0.009 | 0.009 | 0.011 | 0.011 | 0.024 | 0.007 | 0.033 | 0.021 | 0.016 | 0.008 | 0.012 | 0.009 | 0.014 | 0.010 | 0.023 |
| meta1348 |  | 478.805 | 163.129 | 0.003 | 0.004 | 0.003 | 0.000 | 0.001 | 0.003 | 0.003 | 0.006 | 0.001 | 0.006 | 0.003 | 0.002 | 0.004 | 0.006 | 0.002 |
| meta1349 |  | 479.049 | 152.045 | 0.012 | 0.011 | 0.009 | 0.015 | 0.014 | 0.009 | 0.011 | 0.010 | 0.010 | 0.013 | 0.008 | 0.010 | 0.012 | 0.007 | 0.013 |
| meta1350 |  | 479.087 | 198.620 | 0.016 | 0.016 | 0.016 | 0.019 | 0.025 | 0.016 | 0.021 | 0.016 | 0.008 | 0.009 | 0.014 | 0.014 | 0.020 | 0.015 | 0.016 |
| meta1351 |  | 479.089 | 482.304 | 0.007 | 0.008 | 0.008 | 0.009 | 0.008 | 0.007 | 0.007 | 0.006 | 0.008 | 0.007 | 0.007 | 0.007 | 0.010 | 0.009 | 0.010 |
| meta1352 |  | 480.213 | 38.350 | 0.004 | 0.008 | 0.005 | 0.010 | 0.024 | 0.004 | 0.011 | 0.007 | 0.006 | 0.007 | 0.008 | 0.007 | 0.005 | 0.005 | 0.007 |
| meta1353 |  | 480.307 | 179.006 | 0.353 | 0.393 | 0.379 | 0.380 | 0.406 | 0.297 | 0.567 | 0.600 | 0.329 | 0.405 | 0.523 | 0.614 | 0.389 | 0.506 | 0.533 |
| meta1354 |  | 480.342 | 166.363 | 0.012 | 0.020 | 0.020 | 0.020 | 0.031 | 0.010 | 0.035 | 0.033 | 0.020 | 0.015 | 0.026 | 0.031 | 0.019 | 0.017 | 0.031 |
| meta1355 |  | 480.403 | 32.591 | 0.006 | 0.008 | 0.008 | 0.006 | 0.008 | 0.006 | 0.008 | 0.013 | 0.005 | 0.002 | 0.006 | 0.011 | 0.005 | 0.006 | 0.003 |
| meta1356 |  | 481.103 | 152.603 | 0.005 | 0.005 | 0.004 | 0.005 | 0.005 | 0.003 | 0.005 | 0.005 | 0.004 | 0.004 | 0.005 | 0.004 | 0.006 | 0.005 | 0.005 |
| meta1357 |  | 481.186 | 371.206 | 0.010 | 0.009 | 0.008 | 0.004 | 0.006 | 0.004 | 0.024 | 0.007 | 0.008 | 0.005 | 0.007 | 0.017 | 0.009 | 0.012 | 0.009 |
| meta1358 |  | 482.141 | 416.108 | 0.139 | 0.118 | 0.098 | 0.043 | 0.185 | 0.038 | 0.346 | 0.084 | 0.156 | 0.118 | 0.038 | 0.081 | 0.143 | 0.066 | 0.055 |
| meta1359 |  | 482.212 | 295.337 | 0.003 | 0.005 | 0.003 | 0.007 | 0.002 | 0.004 | 0.004 | 0.002 | 0.003 | 0.003 | 0.003 | 0.002 | 0.004 | 0.002 | 0.003 |
| meta1360 |  | 482.402 | 36.639 | 0.017 | 0.013 | 0.013 | 0.010 | 0.007 | 0.009 | 0.027 | 0.016 | 0.013 | 0.012 | 0.013 | 0.019 | 0.013 | 0.009 | 0.013 |
| meta1361 |  | 482.417 | 147.835 | 0.023 | 0.027 | 0.026 | 0.031 | 0.058 | 0.013 | 0.077 | 0.070 | 0.052 | 0.022 | 0.038 | 0.018 | 0.027 | 0.015 | 0.051 |
| meta1362 | IBMX | 483.184 | 362.091 | 0.115 | 0.133 | 0.131 | 0.154 | 0.147 | 0.071 | 0.186 | 0.103 | 0.083 | 0.128 | 0.190 | 0.136 | 0.092 | 0.071 | 0.242 |
| meta1363 |  | 483.304 | 47.767 | 0.013 | 0.015 | 0.013 | 0.034 | 0.022 | 0.012 | 0.018 | 0.008 | 0.018 | 0.012 | 0.010 | 0.010 | 0.014 | 0.006 | 0.012 |
| meta1364 | Glycocholic acid | 483.340 | 234.500 | 0.006 | 0.005 | 0.006 | 0.002 | 0.003 | 0.013 | 0.014 | 0.004 | 0.001 | 0.007 | 0.005 | 0.011 | 0.011 | 0.011 | 0.006 |
| meta1365 |  | 483.361 | 179.025 | 0.051 | 0.050 | 0.046 | 0.044 | 0.031 | 0.060 | 0.076 | 0.045 | 0.031 | 0.027 | 0.062 | 0.065 | 0.069 | 0.067 | 0.020 |
| meta1366 |  | 484.114 | 478.370 | 0.071 | 0.055 | 0.073 | 0.059 | 0.064 | 0.069 | 0.071 | 0.071 | 0.082 | 0.090 | 0.069 | 0.064 | 0.092 | 0.044 | 0.066 |
| meta1367 |  | 484.237 | 347.982 | 0.010 | 0.010 | 0.009 | 0.015 | 0.011 | 0.012 | 0.009 | 0.006 | 0.005 | 0.007 | 0.007 | 0.009 | 0.010 | 0.007 | 0.011 |
| meta1368 |  | 484.274 | 479.585 | 0.071 | 0.084 | 0.084 | 0.077 | 0.078 | 0.074 | 0.072 | 0.080 | 0.076 | 0.059 | 0.066 | 0.064 | 0.086 | 0.075 | 0.079 |
| meta1369 |  | 485.055 | 387.660 | 0.013 | 0.013 | 0.011 | 0.010 | 0.012 | 0.008 | 0.010 | 0.014 | 0.013 | 0.011 | 0.011 | 0.017 | 0.013 | 0.012 | 0.011 |
| meta1370 |  | 485.055 | 414.716 | 0.028 | 0.032 | 0.027 | 0.024 | 0.032 | 0.021 | 0.028 | 0.034 | 0.025 | 0.026 | 0.030 | 0.032 | 0.028 | 0.027 | 0.030 |
| meta1371 |  | 485.055 | 38.195 | 0.005 | 0.005 | 0.006 | 0.006 | 0.008 | 0.005 | 0.008 | 0.005 | 0.007 | 0.007 | 0.008 | 0.007 | 0.007 | 0.009 | 0.008 |
| meta1372 |  | 485.147 | 346.849 | 0.040 | 0.044 | 0.044 | 0.040 | 0.042 | 0.063 | 0.047 | 0.047 | 0.033 | 0.040 | 0.046 | 0.072 | 0.040 | 0.032 | 0.026 |
| meta1373 | Val-Asn | 485.233 | 200.092 | 0.006 | 0.007 | 0.006 | 0.008 | 0.011 | 0.007 | 0.009 | 0.002 | 0.001 | 0.003 | 0.003 | 0.003 | 0.004 | 0.005 | 0.005 |
| meta1374 |  | 485.288 | 34.097 | 0.144 | 0.178 | 0.182 | 0.484 | 0.558 | 0.323 | 0.723 | 0.638 | 0.557 | 0.364 | 0.537 | 0.439 | 0.392 | 0.451 | 0.637 |
| meta1375 |  | 485.285 | 144.736 | 0.014 | 0.018 | 0.017 | 0.021 | 0.017 | 0.022 | 0.029 | 0.023 | 0.019 | 0.024 | 0.024 | 0.020 | 0.014 | 0.017 | 0.021 |
| meta1376 |  | 486.039 | 405.888 | 0.023 | 0.024 | 0.018 | 0.021 | 0.022 | 0.014 | 0.023 | 0.023 | 0.021 | 0.019 | 0.024 | 0.024 | 0.020 | 0.021 | 0.020 |
| meta1377 |  | 486.039 | 431.391 | 0.017 | 0.018 | 0.016 | 0.014 | 0.014 | 0.015 | 0.017 | 0.023 | 0.016 | 0.015 | 0.019 | 0.020 | 0.017 | 0.013 | 0.018 |
| meta1378 |  | 486.196 | 280.087 | 0.007 | 0.008 | 0.008 | 0.007 | 0.007 | 0.006 | 0.010 | 0.009 | 0.008 | 0.010 | 0.009 | 0.009 | 0.007 | 0.007 | 0.009 |
| meta1379 |  | 486.231 | 302.914 | 0.004 | 0.003 | 0.004 | 0.005 | 0.002 | 0.005 | 0.002 | 0.002 | 0.002 | 0.003 | 0.003 | 0.002 | 0.003 | 0.003 | 0.004 |
| meta1380 |  | 486.861 | 318.447 | 0.061 | 0.062 | 0.074 | 0.060 | 0.062 | 0.072 | 0.064 | 0.055 | 0.082 | 0.060 | 0.067 | 0.058 | 0.075 | 0.081 | 0.058 |
| meta1381 |  | 487.092 | 352.355 | 0.198 | 0.226 | 0.220 | 0.277 | 0.319 | 0.288 | 0.363 | 0.375 | 0.373 | 0.384 | 0.332 | 0.267 | 0.328 | 0.198 | 0.358 |
| meta1382 |  | 487.093 | 435.356 | 0.034 | 0.042 | 0.044 | 0.047 | 0.048 | 0.034 | 0.042 | 0.040 | 0.046 | 0.040 | 0.048 | 0.038 | 0.041 | 0.031 | 0.041 |
| meta1383 |  | 487.140 | 304.625 | 0.078 | 0.065 | 0.074 | 0.045 | 0.052 | 0.045 | 0.072 | 0.079 | 0.098 | 0.101 | 0.066 | 0.053 | 0.079 | 0.050 | 0.072 |
| meta1384 |  | 488.214 | 204.074 | 0.021 | 0.019 | 0.014 | 0.015 | 0.031 | 0.017 | 0.031 | 0.020 | 0.008 | 0.015 | 0.011 | 0.014 | 0.017 | 0.016 | 0.010 |
| meta1385 |  | 488.399 | 27.682 | 0.074 | 0.092 | 0.088 | 0.100 | 0.088 | 0.062 | 0.078 | 0.116 | 0.092 | 0.073 | 0.087 | 0.087 | 0.073 | 0.090 | 0.084 |
| meta1386 | Cytidine 5'-diphosphocholine (CDP-choline) | 489.113 | 426.932 | 0.901 | 0.861 | 0.921 | 0.892 | 1.009 | 0.755 | 1.149 | 1.008 | 0.828 | 0.649 | 0.786 | 0.712 | 0.892 | 0.663 | 0.804 |
| meta1387 |  | 489.203 | 310.648 | 0.049 | 0.035 | 0.033 | 0.021 | 0.035 | 0.026 | 0.030 | 0.056 | 0.060 | 0.086 | 0.055 | 0.037 | 0.043 | 0.030 | 0.039 |
| meta1388 |  | 489.318 | 47.221 | 0.014 | 0.020 | 0.013 | 0.026 | 0.026 | 0.017 | 0.031 | 0.025 | 0.015 | 0.019 | 0.016 | 0.036 | 0.016 | 0.022 | 0.032 |
| meta1389 |  | 490.174 | 300.235 | 0.036 | 0.045 | 0.032 | 0.037 | 0.015 | 0.062 | 0.035 | 0.026 | 0.037 | 0.046 | 0.036 | 0.050 | 0.030 | 0.032 | 0.018 |
| meta1390 |  | 490.223 | 428.352 | 0.010 | 0.010 | 0.010 | 0.010 | 0.010 | 0.009 | 0.013 | 0.010 | 0.013 | 0.009 | 0.010 | 0.011 | 0.012 | 0.009 | 0.009 |
| meta1391 |  | 490.289 | 179.349 | 0.010 | 0.010 | 0.009 | 0.010 | 0.014 | 0.009 | 0.017 | 0.016 | 0.007 | 0.009 | 0.011 | 0.011 | 0.008 | 0.010 | 0.009 |
| meta1392 |  | 491.018 | 304.755 | 0.008 | 0.008 | 0.010 | 0.007 | 0.009 | 0.006 | 0.007 | 0.010 | 0.009 | 0.010 | 0.008 | 0.008 | 0.008 | 0.006 | 0.007 |
| meta1393 |  | 491.289 | 23.234 | 0.008 | 0.011 | 0.009 | 0.026 | 0.034 | 0.017 | 0.030 | 0.030 | 0.025 | 0.018 | 0.025 | 0.020 | 0.020 | 0.022 | 0.027 |
| meta1394 |  | 491.303 | 252.323 | 0.145 | 0.180 | 0.126 | 0.235 | 0.148 | 0.204 | 0.040 | 0.069 | 0.138 | 0.013 | 0.146 | 0.063 | 0.001 | 0.010 | 0.155 |
| meta1395 |  | 491.306 | 47.361 | 0.059 | 0.072 | 0.068 | 0.239 | 0.164 | 0.077 | 0.034 | 0.014 | 0.041 | 0.013 | 0.015 | 0.015 | 0.015 | 0.012 | 0.013 |
| meta1396 |  | 492.121 | 411.188 | 0.022 | 0.022 | 0.020 | 0.016 | 0.026 | 0.018 | 0.031 | 0.019 | 0.023 | 0.020 | 0.020 | 0.022 | 0.021 | 0.017 | 0.022 |
| meta1397 |  | 492.137 | 304.724 | 0.007 | 0.006 | 0.007 | 0.005 | 0.006 | 0.005 | 0.007 | 0.008 | 0.008 | 0.010 | 0.006 | 0.006 | 0.007 | 0.004 | 0.006 |
| meta1398 |  | 493.135 | 26.662 | 0.003 | 0.003 | 0.003 | 0.003 | 0.002 | 0.002 | 0.005 | 0.002 | 0.004 | 0.003 | 0.004 | 0.004 | 0.004 | 0.006 | 0.005 |
| meta1399 |  | 493.248 | 348.513 | 0.016 | 0.014 | 0.013 | 0.014 | 0.016 | 0.017 | 0.014 | 0.018 | 0.013 | 0.014 | 0.013 | 0.014 | 0.012 | 0.008 | 0.013 |
| meta1400 |  | 493.274 | 206.640 | 0.006 | 0.006 | 0.007 | 0.005 | 0.006 | 0.005 | 0.006 | 0.004 | 0.002 | 0.005 | 0.005 | 0.008 | 0.005 | 0.006 | 0.005 |
| meta1401 |  | 493.310 | 318.954 | 0.040 | 0.039 | 0.042 | 0.035 | 0.039 | 0.035 | 0.040 | 0.037 | 0.042 | 0.038 | 0.039 | 0.037 | 0.041 | 0.045 | 0.039 |
| meta1402 |  | 494.088 | 417.996 | 0.039 | 0.044 | 0.044 | 0.035 | 0.035 | 0.036 | 0.043 | 0.040 | 0.051 | 0.041 | 0.038 | 0.039 | 0.039 | 0.042 | 0.046 |
| meta1403 |  | 495.073 | 278.716 | 0.008 | 0.013 | 0.012 | 0.011 | 0.010 | 0.008 | 0.012 | 0.010 | 0.018 | 0.012 | 0.011 | 0.011 | 0.012 | 0.012 | 0.013 |
| meta1404 |  | 495.125 | 305.156 | 0.033 | 0.032 | 0.026 | 0.018 | 0.021 | 0.032 | 0.020 | 0.027 | 0.024 | 0.035 | 0.020 | 0.036 | 0.024 | 0.016 | 0.019 |
| meta1405 |  | 495.145 | 372.076 | 0.031 | 0.028 | 0.026 | 0.025 | 0.031 | 0.014 | 0.046 | 0.031 | 0.012 | 0.028 | 0.047 | 0.019 | 0.011 | 0.011 | 0.045 |
| meta1406 |  | 496.270 | 126.857 | 0.419 | 0.400 | 0.344 | 0.070 | 0.074 | 0.545 | 1.022 | 0.430 | 0.094 | 0.844 | 0.415 | 0.543 | 0.764 | 0.750 | 0.270 |
| meta1407 | 1-Palmitoyl-sn-glycero-3-phosphocholine | 496.339 | 174.799 | 51.025 | 55.367 | 53.510 | 59.168 | 61.005 | 44.091 | 83.039 | 63.325 | 42.758 | 45.092 | 53.104 | 53.938 | 47.055 | 51.461 | 56.976 |
| meta1408 |  | 496.361 | 46.436 | 0.005 | 0.007 | 0.008 | 0.009 | 0.009 | 0.008 | 0.016 | 0.009 | 0.013 | 0.010 | 0.009 | 0.010 | 0.007 | 0.010 | 0.006 |
| meta1409 |  | 496.418 | 35.579 | 0.007 | 0.008 | 0.008 | 0.009 | 0.021 | 0.010 | 0.024 | 0.009 | 0.010 | 0.017 | 0.010 | 0.010 | 0.006 | 0.008 | 0.006 |
| meta1410 |  | 496.759 | 163.129 | 0.002 | 0.002 | 0.002 | 0.000 | 0.001 | 0.002 | 0.002 | 0.002 | 0.000 | 0.004 | 0.002 | 0.001 | 0.003 | 0.003 | 0.001 |
| meta1411 |  | 497.057 | 164.387 | 0.122 | 0.130 | 0.144 | 0.110 | 0.144 | 0.128 | 0.128 | 0.076 | 0.202 | 0.091 | 0.124 | 0.127 | 0.159 | 0.129 | 0.131 |
| meta1412 |  | 497.055 | 344.679 | 0.007 | 0.006 | 0.004 | 0.003 | 0.006 | 0.001 | 0.003 | 0.063 | 0.008 | 0.010 | 0.036 | 0.036 | 0.005 | 0.002 | 0.011 |
| meta1413 |  | 497.055 | 428.694 | 0.018 | 0.019 | 0.019 | 0.018 | 0.020 | 0.015 | 0.025 | 0.021 | 0.021 | 0.016 | 0.018 | 0.019 | 0.022 | 0.017 | 0.016 |
| meta1414 |  | 497.062 | 372.319 | 0.009 | 0.011 | 0.009 | 0.012 | 0.011 | 0.006 | 0.012 | 0.011 | 0.009 | 0.011 | 0.011 | 0.009 | 0.008 | 0.009 | 0.014 |
| meta1415 |  | 497.131 | 333.518 | 0.056 | 0.069 | 0.058 | 0.039 | 0.023 | 0.037 | 0.091 | 0.026 | 0.099 | 0.073 | 0.172 | 0.074 | 0.086 | 0.051 | 0.020 |
| meta1416 |  | 497.129 | 152.652 | 0.008 | 0.008 | 0.008 | 0.007 | 0.011 | 0.006 | 0.013 | 0.008 | 0.004 | 0.003 | 0.004 | 0.007 | 0.005 | 0.005 | 0.006 |
| meta1417 |  | 497.233 | 287.936 | 0.011 | 0.012 | 0.012 | 0.014 | 0.012 | 0.011 | 0.012 | 0.011 | 0.013 | 0.012 | 0.012 | 0.014 | 0.011 | 0.013 | 0.012 |
| meta1418 | Cytochalasin B | 497.302 | 163.129 | 0.183 | 0.207 | 0.173 | 0.029 | 0.046 | 0.163 | 0.254 | 0.364 | 0.076 | 0.385 | 0.175 | 0.153 | 0.268 | 0.389 | 0.177 |
| meta1419 |  | 498.086 | 421.276 | 0.021 | 0.026 | 0.021 | 0.023 | 0.027 | 0.018 | 0.024 | 0.021 | 0.020 | 0.016 | 0.022 | 0.024 | 0.021 | 0.025 | 0.028 |
| meta1420 |  | 498.279 | 226.660 | 0.017 | 0.021 | 0.020 | 0.019 | 0.020 | 0.016 | 0.026 | 0.029 | 0.013 | 0.018 | 0.019 | 0.017 | 0.019 | 0.019 | 0.022 |
| meta1421 |  | 498.287 | 163.123 | 0.597 | 0.662 | 0.621 | 0.097 | 0.173 | 0.582 | 0.816 | 1.140 | 0.262 | 1.327 | 0.601 | 0.442 | 0.934 | 1.224 | 0.533 |
| meta1422 |  | 498.353 | 201.462 | 0.007 | 0.011 | 0.007 | 0.005 | 0.005 | 0.004 | 0.016 | 0.008 | 0.004 | 0.008 | 0.008 | 0.009 | 0.006 | 0.008 | 0.009 |
| meta1423 |  | 499.141 | 352.415 | 0.014 | 0.009 | 0.010 | 0.013 | 0.014 | 0.009 | 0.015 | 0.013 | 0.012 | 0.010 | 0.012 | 0.010 | 0.014 | 0.010 | 0.013 |
| meta1424 |  | 500.055 | 413.276 | 0.122 | 0.116 | 0.104 | 0.102 | 0.114 | 0.103 | 0.152 | 0.155 | 0.125 | 0.115 | 0.132 | 0.144 | 0.133 | 0.091 | 0.121 |
| meta1425 |  | 500.246 | 295.224 | 0.014 | 0.014 | 0.011 | 0.016 | 0.004 | 0.018 | 0.010 | 0.008 | 0.006 | 0.008 | 0.012 | 0.008 | 0.010 | 0.010 | 0.010 |
| meta1426 | Taurodeoxycholic acid | 500.311 | 47.182 | 0.011 | 0.016 | 0.015 | 0.054 | 0.033 | 0.024 | 0.014 | 0.018 | 0.020 | 0.011 | 0.015 | 0.015 | 0.019 | 0.017 | 0.028 |
| meta1427 |  | 500.376 | 37.600 | 0.006 | 0.007 | 0.004 | 0.006 | 0.010 | 0.008 | 0.009 | 0.009 | 0.008 | 0.008 | 0.005 | 0.010 | 0.008 | 0.005 | 0.004 |
| meta1428 | Cytidine triphosphate (CTP) | 501.023 | 432.607 | 0.014 | 0.016 | 0.015 | 0.017 | 0.014 | 0.011 | 0.014 | 0.012 | 0.015 | 0.011 | 0.017 | 0.015 | 0.015 | 0.018 | 0.020 |
| meta1429 |  | 501.063 | 275.257 | 0.654 | 0.604 | 0.512 | 0.541 | 0.725 | 0.540 | 0.452 | 0.878 | 0.571 | 0.966 | 0.524 | 0.291 | 0.576 | 0.600 | 0.584 |
| meta1430 |  | 502.104 | 337.466 | 0.498 | 0.433 | 0.416 | 0.459 | 0.399 | 0.394 | 0.414 | 0.448 | 0.502 | 0.553 | 0.406 | 0.422 | 0.401 | 0.381 | 0.435 |
| meta1431 |  | 502.314 | 34.097 | 0.082 | 0.115 | 0.108 | 0.265 | 0.284 | 0.192 | 0.389 | 0.313 | 0.313 | 0.208 | 0.298 | 0.259 | 0.234 | 0.252 | 0.352 |
| meta1432 |  | 502.371 | 35.677 | 0.029 | 0.037 | 0.042 | 0.146 | 0.099 | 0.106 | 0.050 | 0.027 | 0.031 | 0.021 | 0.023 | 0.013 | 0.020 | 0.018 | 0.028 |
| meta1433 |  | 503.158 | 345.523 | 0.060 | 0.061 | 0.057 | 0.050 | 0.059 | 0.086 | 0.062 | 0.063 | 0.044 | 0.051 | 0.064 | 0.080 | 0.050 | 0.046 | 0.039 |
| meta1434 |  | 504.022 | 417.382 | 0.047 | 0.064 | 0.064 | 0.059 | 0.054 | 0.048 | 0.053 | 0.055 | 0.053 | 0.047 | 0.066 | 0.059 | 0.066 | 0.058 | 0.077 |
| meta1435 |  | 504.123 | 415.405 | 0.035 | 0.037 | 0.040 | 0.019 | 0.065 | 0.011 | 0.093 | 0.023 | 0.060 | 0.031 | 0.013 | 0.024 | 0.047 | 0.023 | 0.020 |
| meta1436 |  | 504.302 | 177.670 | 0.120 | 0.092 | 0.084 | 0.077 | 0.072 | 0.071 | 0.122 | 0.162 | 0.093 | 0.111 | 0.161 | 0.161 | 0.087 | 0.097 | 0.132 |
| meta1437 |  | 504.401 | 146.286 | 0.004 | 0.005 | 0.005 | 0.007 | 0.024 | 0.003 | 0.020 | 0.010 | 0.014 | 0.004 | 0.006 | 0.003 | 0.005 | 0.002 | 0.009 |
| meta1438 |  | 505.104 | 434.758 | 0.035 | 0.041 | 0.038 | 0.039 | 0.041 | 0.033 | 0.038 | 0.037 | 0.039 | 0.036 | 0.038 | 0.032 | 0.039 | 0.029 | 0.037 |
| meta1439 |  | 505.166 | 362.382 | 0.086 | 0.157 | 0.120 | 0.127 | 0.133 | 0.097 | 0.149 | 0.102 | 0.103 | 0.112 | 0.164 | 0.112 | 0.091 | 0.116 | 0.154 |
| meta1440 |  | 505.386 | 44.721 | 0.008 | 0.011 | 0.012 | 0.051 | 0.035 | 0.016 | 0.018 | 0.011 | 0.012 | 0.004 | 0.010 | 0.008 | 0.006 | 0.008 | 0.010 |
| meta1441 |  | 506.345 | 47.221 | 0.009 | 0.012 | 0.009 | 0.021 | 0.016 | 0.010 | 0.019 | 0.026 | 0.015 | 0.012 | 0.013 | 0.022 | 0.014 | 0.011 | 0.020 |
| meta1442 |  | 506.358 | 171.514 | 0.070 | 0.078 | 0.085 | 0.103 | 0.106 | 0.059 | 0.108 | 0.094 | 0.065 | 0.081 | 0.105 | 0.102 | 0.086 | 0.058 | 0.124 |
| meta1443 |  | 507.270 | 33.352 | 0.005 | 0.005 | 0.005 | 0.020 | 0.030 | 0.015 | 0.030 | 0.018 | 0.024 | 0.014 | 0.019 | 0.014 | 0.014 | 0.021 | 0.027 |
| meta1444 | 3'-Phosphoadenosine 5'-phosphosulfate (PAPS) | 508.000 | 465.079 | 0.017 | 0.019 | 0.017 | 0.013 | 0.026 | 0.016 | 0.035 | 0.014 | 0.019 | 0.014 | 0.039 | 0.014 | 0.025 | 0.015 | 0.026 |
| meta1445 |  | 508.114 | 411.843 | 0.039 | 0.042 | 0.038 | 0.037 | 0.042 | 0.034 | 0.043 | 0.041 | 0.041 | 0.041 | 0.054 | 0.035 | 0.036 | 0.032 | 0.031 |
| meta1446 | Troglitazone | 508.114 | 205.426 | 0.014 | 0.018 | 0.016 | 0.015 | 0.017 | 0.012 | 0.011 | 0.018 | 0.018 | 0.015 | 0.020 | 0.017 | 0.018 | 0.018 | 0.016 |
| meta1447 |  | 508.116 | 278.517 | 0.009 | 0.008 | 0.006 | 0.008 | 0.008 | 0.007 | 0.008 | 0.009 | 0.007 | 0.007 | 0.008 | 0.008 | 0.006 | 0.008 | 0.008 |
| meta1448 |  | 508.309 | 43.893 | 0.012 | 0.015 | 0.015 | 0.041 | 0.016 | 0.015 | 0.015 | 0.014 | 0.013 | 0.002 | 0.012 | 0.010 | 0.008 | 0.010 | 0.011 |
| meta1449 |  | 508.336 | 173.976 | 0.056 | 0.058 | 0.049 | 0.071 | 0.063 | 0.052 | 0.120 | 0.101 | 0.073 | 0.086 | 0.074 | 0.086 | 0.052 | 0.073 | 0.067 |
| meta1450 |  | 508.373 | 175.833 | 0.130 | 0.123 | 0.133 | 0.150 | 0.177 | 0.143 | 0.095 | 0.128 | 0.080 | 0.110 | 0.160 | 0.171 | 0.133 | 0.123 | 0.213 |
| meta1451 |  | 508.844 | 318.447 | 0.020 | 0.021 | 0.024 | 0.019 | 0.020 | 0.023 | 0.021 | 0.019 | 0.026 | 0.018 | 0.022 | 0.019 | 0.025 | 0.028 | 0.021 |
| meta1452 |  | 509.285 | 135.928 | 0.047 | 0.082 | 0.066 | 0.072 | 0.078 | 0.070 | 0.118 | 0.057 | 0.053 | 0.061 | 0.051 | 0.053 | 0.073 | 0.061 | 0.064 |
| meta1453 |  | 509.285 | 286.775 | 0.177 | 0.187 | 0.183 | 0.190 | 0.196 | 0.151 | 0.187 | 0.190 | 0.212 | 0.197 | 0.199 | 0.178 | 0.212 | 0.181 | 0.193 |
| meta1454 |  | 510.353 | 173.336 | 0.725 | 0.868 | 0.848 | 0.778 | 0.728 | 0.620 | 1.395 | 0.966 | 0.750 | 0.844 | 1.071 | 1.046 | 0.794 | 0.630 | 0.888 |
| meta1455 | 1-O-Octadecyl-sn-glyceryl-3-phosphorylcholine | 510.390 | 176.452 | 0.038 | 0.053 | 0.058 | 0.049 | 0.051 | 0.051 | 0.077 | 0.056 | 0.029 | 0.040 | 0.041 | 0.062 | 0.057 | 0.049 | 0.067 |
| meta1456 |  | 511.093 | 426.589 | 0.019 | 0.020 | 0.018 | 0.023 | 0.023 | 0.022 | 0.029 | 0.022 | 0.020 | 0.014 | 0.022 | 0.017 | 0.021 | 0.015 | 0.021 |
| meta1457 |  | 511.103 | 301.391 | 0.029 | 0.040 | 0.034 | 0.042 | 0.039 | 0.049 | 0.040 | 0.035 | 0.027 | 0.029 | 0.041 | 0.048 | 0.026 | 0.026 | 0.017 |
| meta1458 |  | 511.120 | 152.520 | 0.025 | 0.024 | 0.029 | 0.023 | 0.034 | 0.025 | 0.035 | 0.025 | 0.020 | 0.014 | 0.021 | 0.024 | 0.023 | 0.021 | 0.025 |
| meta1459 |  | 511.171 | 368.921 | 0.026 | 0.029 | 0.022 | 0.028 | 0.033 | 0.028 | 0.032 | 0.025 | 0.022 | 0.027 | 0.034 | 0.029 | 0.019 | 0.031 | 0.014 |
| meta1460 |  | 511.185 | 310.355 | 0.027 | 0.018 | 0.018 | 0.010 | 0.018 | 0.012 | 0.015 | 0.032 | 0.039 | 0.048 | 0.033 | 0.022 | 0.021 | 0.020 | 0.022 |
| meta1461 |  | 512.172 | 222.621 | 0.004 | 0.004 | 0.004 | 0.003 | 0.005 | 0.006 | 0.006 | 0.003 | 0.003 | 0.004 | 0.004 | 0.006 | 0.004 | 0.006 | 0.004 |
| meta1462 |  | 512.334 | 42.531 | 0.025 | 0.034 | 0.031 | 0.046 | 0.058 | 0.039 | 0.075 | 0.079 | 0.012 | 0.019 | 0.037 | 0.069 | 0.024 | 0.032 | 0.058 |
| meta1463 |  | 512.332 | 191.188 | 0.043 | 0.047 | 0.045 | 0.041 | 0.047 | 0.038 | 0.061 | 0.047 | 0.031 | 0.040 | 0.049 | 0.042 | 0.045 | 0.049 | 0.038 |
| meta1464 |  | 512.413 | 37.266 | 0.040 | 0.028 | 0.038 | 0.033 | 0.040 | 0.033 | 0.083 | 0.028 | 0.028 | 0.045 | 0.030 | 0.020 | 0.018 | 0.029 | 0.033 |
| meta1465 |  | 512.717 | 287.465 | 0.013 | 0.013 | 0.013 | 0.015 | 0.016 | 0.012 | 0.016 | 0.015 | 0.016 | 0.016 | 0.014 | 0.014 | 0.019 | 0.014 | 0.016 |
| meta1466 |  | 513.320 | 47.491 | 0.050 | 0.093 | 0.063 | 0.247 | 0.121 | 0.087 | 0.058 | 0.047 | 0.043 | 0.006 | 0.030 | 0.046 | 0.010 | 0.024 | 0.031 |
| meta1467 |  | 513.942 | 613.034 | 0.167 | 0.329 | 0.242 | 0.132 | 0.164 | 0.151 | 0.098 | 0.219 | 0.160 | 0.174 | 0.182 | 0.097 | 0.172 | 0.210 | 0.199 |
| meta1468 |  | 513.942 | 50.190 | 0.006 | 0.006 | 0.008 | 0.005 | 0.006 | 0.010 | 0.006 | 0.005 | 0.008 | 0.005 | 0.006 | 0.004 | 0.007 | 0.004 | 0.007 |
| meta1469 |  | 514.068 | 151.878 | 0.004 | 0.005 | 0.004 | 0.005 | 0.006 | 0.004 | 0.003 | 0.005 | 0.005 | 0.004 | 0.003 | 0.002 | 0.004 | 0.005 | 0.005 |
| meta1470 |  | 514.215 | 223.025 | 0.002 | 0.002 | 0.002 | 0.004 | 0.002 | 0.002 | 0.002 | 0.002 | 0.001 | 0.002 | 0.002 | 0.002 | 0.003 | 0.001 | 0.002 |
| meta1471 |  | 514.281 | 164.398 | 0.143 | 0.170 | 0.138 | 0.016 | 0.037 | 0.096 | 0.455 | 0.132 | 0.070 | 0.159 | 0.222 | 0.195 | 0.194 | 0.241 | 0.101 |
| meta1472 |  | 514.281 | 127.042 | 0.257 | 0.253 | 0.261 | 0.049 | 0.046 | 0.370 | 0.698 | 0.293 | 0.067 | 0.541 | 0.253 | 0.392 | 0.541 | 0.533 | 0.193 |
| meta1473 |  | 514.697 | 288.135 | 0.092 | 0.097 | 0.100 | 0.104 | 0.109 | 0.098 | 0.105 | 0.108 | 0.127 | 0.109 | 0.113 | 0.101 | 0.130 | 0.111 | 0.113 |
| meta1474 |  | 514.715 | 163.118 | 0.005 | 0.004 | 0.004 | 0.000 | 0.001 | 0.005 | 0.006 | 0.008 | 0.002 | 0.009 | 0.004 | 0.003 | 0.006 | 0.008 | 0.004 |
| meta1475 |  | 515.211 | 362.081 | 3.924 | 3.718 | 3.118 | 3.391 | 4.370 | 2.054 | 5.626 | 3.216 | 1.835 | 3.712 | 5.051 | 3.489 | 2.264 | 1.599 | 5.643 |
| meta1476 |  | 515.313 | 45.342 | 0.036 | 0.043 | 0.028 | 0.035 | 0.046 | 0.029 | 0.032 | 0.039 | 0.041 | 0.027 | 0.034 | 0.036 | 0.052 | 0.046 | 0.086 |
| meta1477 |  | 515.312 | 134.649 | 0.061 | 0.060 | 0.046 | 0.014 | 0.021 | 0.078 | 0.144 | 0.079 | 0.041 | 0.158 | 0.080 | 0.061 | 0.124 | 0.147 | 0.046 |
| meta1478 |  | 515.312 | 105.939 | 0.024 | 0.043 | 0.039 | 0.022 | 0.022 | 0.174 | 0.153 | 0.097 | 0.020 | 0.203 | 0.035 | 0.022 | 0.086 | 0.079 | 0.044 |
| meta1479 |  | 515.351 | 42.293 | 0.064 | 0.071 | 0.059 | 0.017 | 0.023 | 0.029 | 0.072 | 0.092 | 0.017 | 0.028 | 0.024 | 0.030 | 0.077 | 0.030 | 0.030 |
| meta1480 |  | 515.410 | 573.148 | 0.016 | 0.020 | 0.017 | 0.018 | 0.020 | 0.015 | 0.019 | 0.019 | 0.020 | 0.019 | 0.019 | 0.019 | 0.019 | 0.018 | 0.020 |
| meta1481 | Taurocholate | 516.297 | 184.581 | 2.190 | 2.372 | 2.277 | 0.451 | 0.798 | 3.336 | 4.074 | 2.387 | 0.809 | 3.420 | 2.817 | 1.124 | 3.176 | 3.498 | 1.295 |
| meta1482 |  | 516.297 | 163.073 | 1.582 | 1.729 | 1.827 | 0.234 | 0.436 | 1.549 | 2.114 | 3.073 | 0.654 | 3.452 | 1.609 | 1.125 | 2.623 | 3.126 | 1.407 |
| meta1483 |  | 516.311 | 308.865 | 0.005 | 0.006 | 0.005 | 0.003 | 0.004 | 0.006 | 0.003 | 0.004 | 0.003 | 0.003 | 0.010 | 0.013 | 0.005 | 0.009 | 0.004 |
| meta1484 |  | 517.114 | 322.284 | 0.045 | 0.048 | 0.046 | 0.050 | 0.039 | 0.033 | 0.047 | 0.052 | 0.054 | 0.056 | 0.057 | 0.039 | 0.039 | 0.039 | 0.051 |
| meta1485 |  | 517.277 | 348.316 | 0.005 | 0.005 | 0.006 | 0.005 | 0.006 | 0.006 | 0.005 | 0.006 | 0.005 | 0.005 | 0.006 | 0.006 | 0.004 | 0.005 | 0.005 |
| meta1486 |  | 518.065 | 413.260 | 0.018 | 0.016 | 0.016 | 0.013 | 0.019 | 0.013 | 0.022 | 0.021 | 0.019 | 0.017 | 0.018 | 0.022 | 0.022 | 0.015 | 0.016 |
| meta1487 |  | 518.089 | 275.376 | 0.060 | 0.054 | 0.052 | 0.049 | 0.081 | 0.046 | 0.055 | 0.080 | 0.044 | 0.076 | 0.048 | 0.026 | 0.060 | 0.056 | 0.048 |
| meta1488 |  | 518.169 | 226.963 | 0.013 | 0.016 | 0.021 | 0.014 | 0.016 | 0.022 | 0.015 | 0.019 | 0.014 | 0.012 | 0.018 | 0.019 | 0.015 | 0.014 | 0.009 |
| meta1489 |  | 518.491 | 34.042 | 0.017 | 0.020 | 0.014 | 0.004 | 0.004 | 0.002 | 0.004 | 0.010 | 0.003 | 0.004 | 0.012 | 0.015 | 0.004 | 0.002 | 0.002 |
| meta1490 |  | 519.111 | 152.578 | 0.030 | 0.030 | 0.024 | 0.027 | 0.052 | 0.019 | 0.045 | 0.030 | 0.012 | 0.009 | 0.016 | 0.024 | 0.016 | 0.020 | 0.022 |
| meta1491 |  | 519.112 | 333.701 | 0.026 | 0.020 | 0.027 | 0.016 | 0.011 | 0.012 | 0.021 | 0.016 | 0.024 | 0.028 | 0.066 | 0.018 | 0.022 | 0.020 | 0.011 |
| meta1492 | Fexofenadine | 519.323 | 175.212 | 0.686 | 0.708 | 0.708 | 0.803 | 0.911 | 0.756 | 1.003 | 0.687 | 0.816 | 0.846 | 0.637 | 0.699 | 0.814 | 0.902 | 0.842 |
| meta1493 |  | 520.185 | 346.772 | 0.632 | 0.666 | 0.635 | 0.545 | 0.668 | 1.002 | 0.669 | 0.679 | 0.474 | 0.548 | 0.665 | 0.931 | 0.565 | 0.493 | 0.374 |
| meta1494 |  | 520.330 | 102.065 | 0.079 | 0.101 | 0.127 | 0.121 | 0.079 | 0.112 | 0.086 | 0.106 | 0.085 | 0.084 | 0.068 | 0.111 | 0.057 | 0.084 | 0.070 |
| meta1495 |  | 521.098 | 421.381 | 0.404 | 0.440 | 0.453 | 0.379 | 0.362 | 0.327 | 0.436 | 0.418 | 0.524 | 0.436 | 0.400 | 0.323 | 0.400 | 0.366 | 0.456 |
| meta1496 |  | 521.103 | 304.755 | 0.007 | 0.005 | 0.005 | 0.002 | 0.003 | 0.003 | 0.003 | 0.006 | 0.005 | 0.008 | 0.004 | 0.004 | 0.006 | 0.003 | 0.003 |
| meta1497 |  | 521.327 | 46.678 | 0.046 | 0.067 | 0.057 | 0.326 | 0.178 | 0.102 | 0.052 | 0.026 | 0.055 | 0.015 | 0.019 | 0.035 | 0.015 | 0.016 | 0.019 |
| meta1498 | Maltotriose | 522.201 | 428.352 | 1.031 | 0.854 | 0.965 | 0.708 | 0.841 | 1.479 | 0.924 | 1.080 | 0.657 | 0.915 | 0.989 | 1.300 | 0.889 | 0.612 | 0.491 |
| meta1499 | Tauroursodeoxycholic acid | 522.283 | 138.417 | 0.182 | 0.216 | 0.186 | 0.123 | 0.127 | 0.295 | 0.416 | 0.213 | 0.132 | 0.428 | 0.325 | 0.122 | 0.306 | 0.397 | 0.301 |
| meta1500 | 1-Oleoyl-sn-glycero-3-phosphocholine | 522.353 | 172.168 | 4.001 | 4.940 | 4.575 | 4.194 | 5.463 | 4.274 | 7.075 | 6.150 | 3.722 | 5.036 | 5.492 | 6.418 | 4.892 | 5.604 | 6.438 |
| meta1501 |  | 522.761 | 171.524 | 0.056 | 0.061 | 0.062 | 0.049 | 0.052 | 0.036 | 0.069 | 0.067 | 0.048 | 0.048 | 0.066 | 0.051 | 0.048 | 0.043 | 0.088 |
| meta1502 |  | 523.045 | 275.376 | 0.130 | 0.147 | 0.128 | 0.120 | 0.139 | 0.112 | 0.125 | 0.169 | 0.138 | 0.169 | 0.113 | 0.095 | 0.131 | 0.128 | 0.129 |
| meta1503 |  | 523.124 | 278.502 | 0.019 | 0.024 | 0.025 | 0.018 | 0.018 | 0.030 | 0.032 | 0.021 | 0.026 | 0.026 | 0.025 | 0.032 | 0.021 | 0.020 | 0.015 |
| meta1504 |  | 523.196 | 361.848 | 0.291 | 0.311 | 0.263 | 0.284 | 0.366 | 0.156 | 0.455 | 0.269 | 0.204 | 0.300 | 0.386 | 0.304 | 0.162 | 0.179 | 0.438 |
| meta1505 |  | 524.093 | 278.417 | 0.026 | 0.027 | 0.028 | 0.029 | 0.028 | 0.025 | 0.037 | 0.030 | 0.024 | 0.021 | 0.023 | 0.024 | 0.025 | 0.023 | 0.029 |
| meta1506 |  | 524.272 | 177.664 | 0.031 | 0.040 | 0.041 | 0.039 | 0.042 | 0.032 | 0.043 | 0.038 | 0.038 | 0.048 | 0.054 | 0.050 | 0.057 | 0.064 | 0.052 |
| meta1507 |  | 524.319 | 38.284 | 0.025 | 0.030 | 0.026 | 0.174 | 0.067 | 0.053 | 0.013 | 0.015 | 0.024 | 0.006 | 0.011 | 0.010 | 0.011 | 0.010 | 0.010 |
| meta1508 | 1-Stearoyl-sn-glycerol 3-phosphocholine | 524.370 | 171.524 | 22.993 | 26.763 | 26.646 | 25.699 | 28.133 | 19.540 | 40.060 | 36.192 | 22.120 | 24.864 | 32.808 | 30.928 | 24.936 | 19.750 | 41.914 |
| meta1509 |  | 524.391 | 33.431 | 0.008 | 0.006 | 0.005 | 0.009 | 0.009 | 0.022 | 0.019 | 0.004 | 0.004 | 0.005 | 0.017 | 0.009 | 0.004 | 0.004 | 0.007 |
| meta1510 |  | 525.140 | 474.494 | 0.045 | 0.045 | 0.039 | 0.029 | 0.027 | 0.030 | 0.028 | 0.044 | 0.042 | 0.049 | 0.042 | 0.046 | 0.058 | 0.036 | 0.041 |
| meta1511 |  | 525.141 | 344.079 | 1.490 | 1.642 | 1.512 | 1.598 | 1.726 | 2.082 | 1.559 | 1.631 | 1.089 | 1.293 | 1.612 | 2.162 | 1.186 | 1.124 | 0.948 |
| meta1512 |  | 526.172 | 450.757 | 0.037 | 0.039 | 0.044 | 0.038 | 0.047 | 0.055 | 0.036 | 0.045 | 0.021 | 0.032 | 0.041 | 0.053 | 0.025 | 0.031 | 0.025 |
| meta1513 |  | 526.311 | 223.029 | 0.087 | 0.087 | 0.083 | 0.069 | 0.084 | 0.059 | 0.106 | 0.129 | 0.069 | 0.085 | 0.104 | 0.092 | 0.075 | 0.064 | 0.110 |
| meta1514 |  | 526.428 | 36.973 | 0.009 | 0.009 | 0.009 | 0.008 | 0.006 | 0.007 | 0.028 | 0.014 | 0.009 | 0.008 | 0.006 | 0.011 | 0.008 | 0.008 | 0.007 |
| meta1515 |  | 527.091 | 152.578 | 0.031 | 0.033 | 0.037 | 0.033 | 0.044 | 0.028 | 0.047 | 0.030 | 0.025 | 0.019 | 0.027 | 0.030 | 0.030 | 0.028 | 0.033 |
| meta1516 | Raffinose | 527.156 | 428.120 | 0.807 | 0.881 | 0.868 | 0.886 | 0.801 | 1.100 | 0.785 | 0.838 | 0.596 | 0.768 | 0.855 | 0.935 | 0.624 | 0.587 | 0.450 |
| meta1517 |  | 527.401 | 45.309 | 0.008 | 0.010 | 0.009 | 0.056 | 0.034 | 0.014 | 0.013 | 0.004 | 0.009 | 0.007 | 0.009 | 0.012 | 0.006 | 0.005 | 0.006 |
| meta1518 |  | 528.135 | 464.785 | 0.024 | 0.027 | 0.019 | 0.042 | 0.027 | 0.018 | 0.014 | 0.040 | 0.019 | 0.017 | 0.023 | 0.026 | 0.023 | 0.038 | 0.025 |
| meta1519 |  | 528.177 | 96.117 | 0.017 | 0.021 | 0.022 | 0.027 | 0.029 | 0.021 | 0.032 | 0.031 | 0.017 | 0.016 | 0.016 | 0.014 | 0.023 | 0.025 | 0.018 |
| meta1520 |  | 530.018 | 417.393 | 0.032 | 0.029 | 0.031 | 0.029 | 0.026 | 0.026 | 0.025 | 0.037 | 0.032 | 0.032 | 0.029 | 0.026 | 0.031 | 0.033 | 0.044 |
| meta1521 |  | 530.314 | 43.717 | 0.027 | 0.016 | 0.023 | 0.039 | 0.027 | 0.017 | 0.035 | 0.008 | 0.014 | 0.007 | 0.014 | 0.005 | 0.002 | 0.006 | 0.006 |
| meta1522 |  | 531.110 | 300.435 | 0.005 | 0.003 | 0.004 | 0.003 | 0.006 | 0.002 | 0.004 | 0.004 | 0.002 | 0.003 | 0.004 | 0.003 | 0.003 | 0.002 | 0.002 |
| meta1523 |  | 531.134 | 465.976 | 0.145 | 0.135 | 0.139 | 0.150 | 0.128 | 0.105 | 0.130 | 0.190 | 0.157 | 0.111 | 0.132 | 0.087 | 0.116 | 0.124 | 0.169 |
| meta1524 |  | 531.307 | 165.027 | 0.413 | 0.458 | 0.413 | 0.047 | 0.101 | 0.231 | 1.362 | 0.327 | 0.197 | 0.409 | 0.713 | 0.493 | 0.521 | 0.682 | 0.245 |
| meta1525 |  | 531.686 | 184.553 | 0.031 | 0.032 | 0.031 | 0.005 | 0.013 | 0.042 | 0.060 | 0.030 | 0.009 | 0.045 | 0.026 | 0.011 | 0.056 | 0.051 | 0.018 |
| meta1526 |  | 531.689 | 162.986 | 0.010 | 0.013 | 0.010 | 0.002 | 0.003 | 0.012 | 0.014 | 0.018 | 0.005 | 0.021 | 0.010 | 0.008 | 0.018 | 0.020 | 0.009 |
| meta1527 |  | 532.482 | 26.662 | 0.006 | 0.007 | 0.006 | 0.007 | 0.059 | 0.004 | 0.006 | 0.007 | 0.004 | 0.005 | 0.005 | 0.005 | 0.007 | 0.005 | 0.015 |
| meta1528 |  | 533.107 | 152.578 | 0.018 | 0.018 | 0.016 | 0.019 | 0.024 | 0.013 | 0.023 | 0.017 | 0.014 | 0.010 | 0.012 | 0.017 | 0.015 | 0.014 | 0.018 |
| meta1529 |  | 533.264 | 401.346 | 0.008 | 0.008 | 0.006 | 0.005 | 0.007 | 0.008 | 0.003 | 0.010 | 0.003 | 0.008 | 0.007 | 0.009 | 0.005 | 0.008 | 0.005 |
| meta1530 |  | 533.325 | 163.029 | 4.919 | 5.356 | 5.085 | 0.655 | 1.297 | 4.706 | 6.302 | 8.933 | 1.962 | 10.583 | 4.783 | 3.251 | 7.993 | 9.606 | 4.152 |
| meta1531 |  | 534.141 | 389.542 | 0.098 | 0.103 | 0.101 | 0.095 | 0.089 | 0.072 | 0.094 | 0.091 | 0.113 | 0.101 | 0.091 | 0.083 | 0.091 | 0.088 | 0.103 |
| meta1532 |  | 534.140 | 321.457 | 0.010 | 0.014 | 0.018 | 0.009 | 0.010 | 0.006 | 0.010 | 0.013 | 0.012 | 0.012 | 0.011 | 0.010 | 0.007 | 0.010 | 0.013 |
| meta1533 |  | 534.448 | 572.368 | 0.026 | 0.024 | 0.034 | 0.020 | 0.033 | 0.001 | 0.003 | 0.003 | 0.031 | 0.003 | 0.003 | 0.003 | 0.003 | 0.004 | 0.028 |
| meta1534 |  | 535.124 | 347.438 | 0.057 | 0.062 | 0.057 | 0.045 | 0.048 | 0.046 | 0.063 | 0.061 | 0.079 | 0.074 | 0.052 | 0.046 | 0.060 | 0.045 | 0.067 |
| meta1535 |  | 535.394 | 45.771 | 0.013 | 0.017 | 0.018 | 0.040 | 0.034 | 0.015 | 0.017 | 0.013 | 0.017 | 0.011 | 0.016 | 0.012 | 0.012 | 0.011 | 0.011 |
| meta1536 |  | 536.109 | 408.408 | 0.168 | 0.193 | 0.183 | 0.155 | 0.159 | 0.134 | 0.195 | 0.191 | 0.219 | 0.208 | 0.178 | 0.150 | 0.176 | 0.142 | 0.198 |
| meta1537 |  | 536.211 | 27.218 | 0.174 | 0.214 | 0.194 | 0.237 | 0.212 | 0.134 | 0.186 | 0.265 | 0.214 | 0.184 | 0.213 | 0.222 | 0.190 | 0.206 | 0.215 |
| meta1538 |  | 536.336 | 47.634 | 0.012 | 0.014 | 0.016 | 0.036 | 0.021 | 0.016 | 0.011 | 0.005 | 0.011 | 0.007 | 0.004 | 0.014 | 0.005 | 0.003 | 0.006 |
| meta1539 |  | 536.501 | 34.242 | 0.021 | 0.021 | 0.020 | 0.007 | 0.005 | 0.004 | 0.006 | 0.015 | 0.005 | 0.006 | 0.014 | 0.019 | 0.007 | 0.003 | 0.006 |
| meta1540 |  | 536.762 | 286.861 | 0.008 | 0.007 | 0.007 | 0.008 | 0.009 | 0.006 | 0.009 | 0.009 | 0.007 | 0.008 | 0.009 | 0.010 | 0.009 | 0.007 | 0.009 |
| meta1541 |  | 537.140 | 408.227 | 0.107 | 0.123 | 0.111 | 0.109 | 0.100 | 0.083 | 0.114 | 0.121 | 0.129 | 0.120 | 0.116 | 0.104 | 0.104 | 0.083 | 0.116 |
| meta1542 |  | 537.193 | 362.091 | 5.307 | 6.722 | 5.711 | 5.574 | 6.178 | 4.354 | 5.958 | 5.529 | 5.377 | 5.551 | 5.963 | 5.256 | 4.787 | 4.645 | 6.380 |
| meta1543 |  | 537.336 | 318.948 | 0.028 | 0.026 | 0.029 | 0.025 | 0.026 | 0.027 | 0.026 | 0.028 | 0.029 | 0.027 | 0.027 | 0.025 | 0.031 | 0.030 | 0.026 |
| meta1544 |  | 537.392 | 573.148 | 0.018 | 0.022 | 0.019 | 0.019 | 0.020 | 0.016 | 0.022 | 0.020 | 0.021 | 0.021 | 0.021 | 0.021 | 0.021 | 0.020 | 0.022 |
| meta1545 |  | 538.124 | 369.466 | 0.046 | 0.064 | 0.051 | 0.045 | 0.048 | 0.041 | 0.048 | 0.056 | 0.065 | 0.057 | 0.049 | 0.038 | 0.055 | 0.038 | 0.073 |
| meta1546 |  | 538.209 | 27.382 | 0.059 | 0.073 | 0.064 | 0.081 | 0.074 | 0.045 | 0.061 | 0.084 | 0.074 | 0.064 | 0.069 | 0.076 | 0.068 | 0.076 | 0.067 |
| meta1547 |  | 538.278 | 162.504 | 0.232 | 0.227 | 0.217 | 0.047 | 0.073 | 0.180 | 0.258 | 0.311 | 0.125 | 0.389 | 0.229 | 0.179 | 0.259 | 0.303 | 0.196 |
| meta1548 |  | 538.347 | 152.729 | 0.017 | 0.021 | 0.017 | 0.030 | 0.028 | 0.024 | 0.017 | 0.024 | 0.014 | 0.019 | 0.028 | 0.019 | 0.016 | 0.016 | 0.021 |
| meta1549 | 1-Palmitoyllysophosphatidylcholine | 538.384 | 170.264 | 0.420 | 0.441 | 0.402 | 0.355 | 0.290 | 0.286 | 0.606 | 0.528 | 0.390 | 0.447 | 0.685 | 0.517 | 0.459 | 0.346 | 0.465 |
| meta1550 | N-Palmitoylsphingosine | 538.517 | 33.990 | 0.100 | 0.116 | 0.089 | 0.018 | 0.008 | 0.008 | 0.009 | 0.064 | 0.010 | 0.023 | 0.079 | 0.079 | 0.030 | 0.005 | 0.016 |
| meta1551 |  | 538.742 | 287.221 | 0.038 | 0.033 | 0.031 | 0.035 | 0.042 | 0.031 | 0.037 | 0.042 | 0.041 | 0.043 | 0.048 | 0.041 | 0.042 | 0.037 | 0.043 |
| meta1552 |  | 539.015 | 275.462 | 0.018 | 0.021 | 0.023 | 0.018 | 0.024 | 0.017 | 0.021 | 0.023 | 0.020 | 0.025 | 0.018 | 0.017 | 0.020 | 0.021 | 0.022 |
| meta1553 |  | 539.096 | 320.759 | 0.032 | 0.029 | 0.036 | 0.027 | 0.023 | 0.023 | 0.031 | 0.034 | 0.035 | 0.035 | 0.029 | 0.025 | 0.032 | 0.024 | 0.054 |
| meta1554 |  | 540.150 | 338.188 | 0.074 | 0.097 | 0.083 | 0.085 | 0.084 | 0.107 | 0.087 | 0.085 | 0.083 | 0.076 | 0.101 | 0.118 | 0.077 | 0.080 | 0.060 |
| meta1555 |  | 540.363 | 186.511 | 0.061 | 0.058 | 0.048 | 0.035 | 0.044 | 0.041 | 0.060 | 0.063 | 0.028 | 0.058 | 0.072 | 0.059 | 0.073 | 0.068 | 0.075 |
| meta1556 |  | 541.114 | 343.908 | 0.308 | 0.290 | 0.286 | 0.332 | 0.354 | 0.413 | 0.297 | 0.337 | 0.231 | 0.263 | 0.320 | 0.415 | 0.261 | 0.248 | 0.213 |
| meta1557 |  | 541.259 | 287.956 | 0.008 | 0.008 | 0.009 | 0.010 | 0.009 | 0.009 | 0.008 | 0.008 | 0.009 | 0.009 | 0.010 | 0.008 | 0.008 | 0.008 | 0.008 |
| meta1558 |  | 541.456 | 33.588 | 0.007 | 0.007 | 0.004 | 0.043 | 0.007 | 0.004 | 0.008 | 0.006 | 0.003 | 0.008 | 0.007 | 0.007 | 0.003 | 0.055 | 0.052 |
| meta1559 |  | 542.319 | 173.336 | 0.466 | 0.547 | 0.373 | 0.439 | 0.372 | 0.552 | 0.698 | 0.497 | 0.702 | 0.715 | 0.448 | 0.724 | 0.604 | 0.636 | 0.531 |
| meta1560 |  | 542.668 | 170.330 | 0.012 | 0.010 | 0.014 | 0.015 | 0.017 | 0.009 | 0.033 | 0.014 | 0.016 | 0.010 | 0.016 | 0.016 | 0.010 | 0.010 | 0.016 |
| meta1561 |  | 543.130 | 428.352 | 0.109 | 0.107 | 0.110 | 0.117 | 0.110 | 0.128 | 0.100 | 0.107 | 0.080 | 0.100 | 0.110 | 0.128 | 0.081 | 0.080 | 0.062 |
| meta1562 |  | 544.265 | 138.806 | 0.047 | 0.052 | 0.034 | 0.041 | 0.041 | 0.087 | 0.118 | 0.045 | 0.037 | 0.116 | 0.071 | 0.029 | 0.082 | 0.113 | 0.089 |
| meta1563 |  | 545.152 | 202.206 | 0.013 | 0.013 | 0.012 | 0.013 | 0.017 | 0.012 | 0.018 | 0.011 | 0.013 | 0.013 | 0.011 | 0.011 | 0.013 | 0.016 | 0.013 |
| meta1564 |  | 545.176 | 361.610 | 0.061 | 0.062 | 0.055 | 0.048 | 0.077 | 0.050 | 0.064 | 0.065 | 0.029 | 0.066 | 0.069 | 0.047 | 0.037 | 0.030 | 0.063 |
| meta1565 |  | 545.301 | 370.064 | 0.005 | 0.005 | 0.005 | 0.004 | 0.006 | 0.004 | 0.005 | 0.005 | 0.004 | 0.004 | 0.005 | 0.005 | 0.006 | 0.005 | 0.005 |
| meta1566 |  | 546.069 | 416.763 | 0.017 | 0.021 | 0.017 | 0.017 | 0.020 | 0.015 | 0.012 | 0.010 | 0.017 | 0.018 | 0.021 | 0.014 | 0.017 | 0.013 | 0.018 |
| meta1567 |  | 546.187 | 159.425 | 0.005 | 0.006 | 0.007 | 0.004 | 0.008 | 0.005 | 0.008 | 0.011 | 0.007 | 0.009 | 0.006 | 0.005 | 0.005 | 0.004 | 0.008 |
| meta1568 |  | 546.397 | 37.422 | 0.043 | 0.055 | 0.050 | 0.231 | 0.133 | 0.125 | 0.065 | 0.040 | 0.050 | 0.042 | 0.035 | 0.031 | 0.031 | 0.029 | 0.047 |
| meta1569 |  | 547.090 | 152.255 | 0.003 | 0.004 | 0.003 | 0.004 | 0.005 | 0.003 | 0.005 | 0.003 | 0.003 | 0.002 | 0.003 | 0.003 | 0.003 | 0.003 | 0.004 |
| meta1570 |  | 547.269 | 304.724 | 0.006 | 0.006 | 0.006 | 0.004 | 0.005 | 0.005 | 0.007 | 0.006 | 0.005 | 0.005 | 0.007 | 0.007 | 0.006 | 0.006 | 0.005 |
| meta1571 |  | 548.215 | 344.901 | 0.021 | 0.020 | 0.021 | 0.018 | 0.023 | 0.028 | 0.021 | 0.022 | 0.015 | 0.018 | 0.021 | 0.029 | 0.016 | 0.017 | 0.013 |
| meta1572 |  | 548.439 | 434.466 | 0.007 | 0.007 | 0.009 | 0.007 | 0.009 | 0.007 | 0.007 | 0.009 | 0.010 | 0.010 | 0.009 | 0.007 | 0.010 | 0.007 | 0.008 |
| meta1573 |  | 548.862 | 318.447 | 0.027 | 0.027 | 0.034 | 0.025 | 0.028 | 0.033 | 0.028 | 0.026 | 0.036 | 0.028 | 0.027 | 0.025 | 0.034 | 0.038 | 0.027 |
| meta1574 |  | 549.275 | 169.603 | 0.007 | 0.007 | 0.008 | 0.010 | 0.012 | 0.009 | 0.013 | 0.010 | 0.023 | 0.010 | 0.007 | 0.008 | 0.017 | 0.010 | 0.009 |
| meta1575 |  | 550.010 | 473.656 | 0.023 | 0.025 | 0.021 | 0.020 | 0.031 | 0.024 | 0.025 | 0.025 | 0.020 | 0.022 | 0.026 | 0.032 | 0.031 | 0.029 | 0.029 |
| meta1576 |  | 550.060 | 164.326 | 0.022 | 0.028 | 0.029 | 0.024 | 0.036 | 0.029 | 0.025 | 0.015 | 0.045 | 0.016 | 0.024 | 0.027 | 0.035 | 0.028 | 0.028 |
| meta1577 |  | 550.124 | 467.917 | 0.386 | 0.475 | 0.447 | 0.330 | 0.430 | 0.373 | 0.443 | 0.518 | 0.412 | 0.551 | 0.488 | 0.365 | 0.476 | 0.355 | 0.389 |
| meta1578 |  | 550.126 | 434.172 | 3.176 | 3.237 | 3.217 | 2.873 | 3.136 | 2.868 | 3.383 | 3.877 | 4.094 | 3.918 | 3.474 | 3.064 | 3.588 | 2.677 | 3.404 |
| meta1579 |  | 550.141 | 344.083 | 0.008 | 0.009 | 0.007 | 0.007 | 0.009 | 0.006 | 0.007 | 0.010 | 0.006 | 0.011 | 0.010 | 0.005 | 0.005 | 0.006 | 0.014 |
| meta1580 |  | 550.231 | 427.990 | 0.036 | 0.033 | 0.033 | 0.029 | 0.033 | 0.039 | 0.031 | 0.035 | 0.022 | 0.029 | 0.040 | 0.040 | 0.030 | 0.025 | 0.018 |
| meta1581 |  | 550.349 | 163.129 | 0.070 | 0.089 | 0.098 | 0.014 | 0.029 | 0.099 | 0.106 | 0.138 | 0.042 | 0.176 | 0.087 | 0.048 | 0.135 | 0.148 | 0.068 |
| meta1582 | 1-O-(cis-9-Octadecenyl)-2-O-acetyl-sn-glycero-3-phosphocholine | 550.384 | 169.634 | 0.117 | 0.124 | 0.143 | 0.132 | 0.117 | 0.098 | 0.173 | 0.258 | 0.146 | 0.153 | 0.182 | 0.213 | 0.135 | 0.185 | 0.217 |
| meta1583 |  | 551.342 | 45.551 | 0.024 | 0.023 | 0.027 | 0.160 | 0.076 | 0.054 | 0.024 | 0.009 | 0.027 | 0.016 | 0.011 | 0.011 | 0.014 | 0.012 | 0.008 |
| meta1584 |  | 551.402 | 279.877 | 0.003 | 0.003 | 0.003 | 0.000 | 0.000 | 0.001 | 0.003 | 0.004 | 0.001 | 0.000 | 0.002 | 0.005 | 0.006 | 0.009 | 0.008 |
| meta1585 |  | 552.362 | 148.300 | 0.008 | 0.010 | 0.009 | 0.020 | 0.015 | 0.022 | 0.011 | 0.013 | 0.005 | 0.005 | 0.011 | 0.011 | 0.005 | 0.007 | 0.007 |
| meta1586 |  | 552.653 | 288.152 | 0.012 | 0.012 | 0.014 | 0.013 | 0.016 | 0.013 | 0.012 | 0.013 | 0.017 | 0.014 | 0.015 | 0.014 | 0.016 | 0.016 | 0.014 |
| meta1587 |  | 552.808 | 318.444 | 0.012 | 0.012 | 0.014 | 0.011 | 0.013 | 0.014 | 0.012 | 0.011 | 0.015 | 0.012 | 0.013 | 0.012 | 0.015 | 0.015 | 0.012 |
| meta1588 |  | 553.135 | 454.375 | 0.054 | 0.058 | 0.057 | 0.049 | 0.051 | 0.046 | 0.059 | 0.059 | 0.066 | 0.059 | 0.052 | 0.043 | 0.059 | 0.045 | 0.061 |
| meta1589 |  | 553.166 | 362.091 | 0.420 | 0.439 | 0.419 | 0.407 | 0.458 | 0.314 | 0.455 | 0.398 | 0.353 | 0.425 | 0.417 | 0.377 | 0.357 | 0.289 | 0.482 |
| meta1590 |  | 553.311 | 287.221 | 0.233 | 0.261 | 0.251 | 0.257 | 0.258 | 0.202 | 0.248 | 0.254 | 0.282 | 0.251 | 0.256 | 0.230 | 0.274 | 0.240 | 0.248 |
| meta1591 |  | 554.119 | 427.309 | 0.042 | 0.060 | 0.061 | 0.051 | 0.053 | 0.047 | 0.051 | 0.050 | 0.076 | 0.072 | 0.054 | 0.048 | 0.052 | 0.049 | 0.064 |
| meta1592 |  | 554.248 | 184.553 | 0.172 | 0.164 | 0.153 | 0.048 | 0.079 | 0.162 | 0.200 | 0.168 | 0.070 | 0.216 | 0.160 | 0.081 | 0.186 | 0.206 | 0.108 |
| meta1593 |  | 554.250 | 162.533 | 0.052 | 0.052 | 0.046 | 0.008 | 0.013 | 0.043 | 0.062 | 0.090 | 0.024 | 0.090 | 0.051 | 0.040 | 0.063 | 0.074 | 0.040 |
| meta1594 |  | 554.285 | 150.292 | 0.006 | 0.006 | 0.005 | 0.009 | 0.006 | 0.006 | 0.007 | 0.005 | 0.007 | 0.007 | 0.007 | 0.007 | 0.006 | 0.009 | 0.007 |
| meta1595 |  | 554.306 | 217.170 | 0.007 | 0.008 | 0.009 | 0.013 | 0.009 | 0.008 | 0.006 | 0.005 | 0.008 | 0.008 | 0.005 | 0.004 | 0.007 | 0.006 | 0.006 |
| meta1596 |  | 554.390 | 174.738 | 0.134 | 0.129 | 0.123 | 0.137 | 0.149 | 0.112 | 0.177 | 0.153 | 0.123 | 0.110 | 0.146 | 0.151 | 0.128 | 0.120 | 0.132 |
| meta1597 |  | 555.103 | 468.523 | 0.042 | 0.047 | 0.040 | 0.037 | 0.038 | 0.034 | 0.047 | 0.044 | 0.058 | 0.047 | 0.039 | 0.032 | 0.043 | 0.033 | 0.053 |
| meta1598 |  | 555.290 | 232.585 | 0.051 | 0.053 | 0.045 | 0.042 | 0.036 | 0.033 | 0.072 | 0.072 | 0.028 | 0.040 | 0.046 | 0.042 | 0.032 | 0.044 | 0.034 |
| meta1599 |  | 555.305 | 184.082 | 0.021 | 0.023 | 0.024 | 0.007 | 0.014 | 0.019 | 0.034 | 0.025 | 0.011 | 0.024 | 0.023 | 0.015 | 0.024 | 0.022 | 0.015 |
| meta1600 |  | 556.125 | 338.838 | 0.018 | 0.024 | 0.018 | 0.025 | 0.021 | 0.028 | 0.021 | 0.019 | 0.017 | 0.016 | 0.023 | 0.035 | 0.022 | 0.025 | 0.013 |
| meta1601 |  | 556.135 | 477.229 | 0.034 | 0.031 | 0.032 | 0.026 | 0.029 | 0.030 | 0.031 | 0.031 | 0.032 | 0.029 | 0.035 | 0.031 | 0.037 | 0.022 | 0.026 |
| meta1602 |  | 556.439 | 38.106 | 0.032 | 0.025 | 0.027 | 0.027 | 0.030 | 0.026 | 0.066 | 0.026 | 0.025 | 0.031 | 0.028 | 0.021 | 0.019 | 0.022 | 0.024 |
| meta1603 |  | 557.321 | 239.843 | 0.007 | 0.005 | 0.004 | 0.005 | 0.006 | 0.006 | 0.009 | 0.004 | 0.002 | 0.004 | 0.003 | 0.004 | 0.007 | 0.004 | 0.005 |
| meta1604 |  | 557.348 | 47.051 | 0.044 | 0.049 | 0.047 | 0.137 | 0.084 | 0.050 | 0.018 | 0.010 | 0.024 | 0.010 | 0.010 | 0.015 | 0.010 | 0.010 | 0.008 |
| meta1605 |  | 558.061 | 422.524 | 0.021 | 0.019 | 0.018 | 0.018 | 0.017 | 0.014 | 0.021 | 0.009 | 0.019 | 0.019 | 0.012 | 0.013 | 0.023 | 0.014 | 0.018 |
| meta1606 |  | 558.091 | 408.817 | 0.062 | 0.067 | 0.063 | 0.052 | 0.057 | 0.048 | 0.064 | 0.070 | 0.070 | 0.078 | 0.069 | 0.058 | 0.060 | 0.045 | 0.062 |
| meta1607 |  | 558.292 | 173.342 | 0.062 | 0.059 | 0.044 | 0.113 | 0.098 | 0.070 | 0.112 | 0.064 | 0.098 | 0.094 | 0.079 | 0.103 | 0.052 | 0.079 | 0.086 |
| meta1608 |  | 558.719 | 288.144 | 0.003 | 0.004 | 0.003 | 0.005 | 0.004 | 0.004 | 0.004 | 0.004 | 0.004 | 0.004 | 0.004 | 0.004 | 0.005 | 0.004 | 0.003 |
| meta1609 |  | 559.149 | 201.717 | 3.313 | 3.181 | 2.967 | 2.967 | 3.651 | 2.481 | 3.522 | 2.917 | 2.094 | 2.569 | 3.021 | 3.113 | 2.702 | 3.094 | 3.113 |
| meta1610 | ADP-ribose | 560.076 | 410.555 | 0.068 | 0.066 | 0.063 | 0.047 | 0.059 | 0.052 | 0.072 | 0.070 | 0.093 | 0.075 | 0.073 | 0.094 | 0.106 | 0.054 | 0.071 |
| meta1611 |  | 560.106 | 381.704 | 0.018 | 0.017 | 0.018 | 0.017 | 0.018 | 0.013 | 0.021 | 0.018 | 0.022 | 0.019 | 0.016 | 0.014 | 0.017 | 0.015 | 0.017 |
| meta1612 |  | 560.239 | 139.059 | 0.010 | 0.009 | 0.008 | 0.010 | 0.010 | 0.024 | 0.036 | 0.011 | 0.010 | 0.028 | 0.017 | 0.007 | 0.024 | 0.031 | 0.021 |
| meta1613 |  | 560.260 | 184.852 | 0.071 | 0.062 | 0.059 | 0.036 | 0.056 | 0.054 | 0.068 | 0.073 | 0.049 | 0.081 | 0.062 | 0.055 | 0.069 | 0.061 | 0.058 |
| meta1614 |  | 560.260 | 162.493 | 0.033 | 0.027 | 0.023 | 0.009 | 0.014 | 0.017 | 0.022 | 0.028 | 0.017 | 0.028 | 0.025 | 0.023 | 0.024 | 0.024 | 0.027 |
| meta1615 |  | 560.308 | 172.124 | 0.052 | 0.041 | 0.056 | 0.057 | 0.071 | 0.046 | 0.065 | 0.064 | 0.038 | 0.059 | 0.046 | 0.054 | 0.053 | 0.063 | 0.072 |
| meta1616 |  | 560.366 | 170.432 | 0.017 | 0.024 | 0.027 | 0.028 | 0.018 | 0.020 | 0.030 | 0.027 | 0.027 | 0.031 | 0.046 | 0.030 | 0.030 | 0.024 | 0.033 |
| meta1617 |  | 560.412 | 37.390 | 0.008 | 0.013 | 0.012 | 0.026 | 0.017 | 0.014 | 0.009 | 0.015 | 0.014 | 0.010 | 0.012 | 0.013 | 0.012 | 0.013 | 0.013 |
| meta1618 |  | 560.468 | 573.148 | 0.010 | 0.011 | 0.009 | 0.011 | 0.010 | 0.008 | 0.011 | 0.011 | 0.011 | 0.011 | 0.012 | 0.010 | 0.011 | 0.010 | 0.011 |
| meta1619 |  | 561.308 | 33.352 | 0.006 | 0.009 | 0.007 | 0.016 | 0.011 | 0.006 | 0.015 | 0.010 | 0.013 | 0.009 | 0.008 | 0.009 | 0.010 | 0.012 | 0.028 |
| meta1620 |  | 561.354 | 184.269 | 0.072 | 0.069 | 0.055 | 0.011 | 0.033 | 0.055 | 0.085 | 0.069 | 0.020 | 0.096 | 0.066 | 0.027 | 0.073 | 0.072 | 0.038 |
| meta1621 |  | 563.067 | 152.578 | 0.010 | 0.012 | 0.011 | 0.013 | 0.016 | 0.009 | 0.018 | 0.010 | 0.009 | 0.008 | 0.010 | 0.010 | 0.012 | 0.010 | 0.012 |
| meta1622 |  | 563.180 | 224.359 | 0.005 | 0.004 | 0.004 | 0.003 | 0.005 | 0.007 | 0.005 | 0.006 | 0.006 | 0.006 | 0.005 | 0.005 | 0.006 | 0.003 | 0.004 |
| meta1623 |  | 563.263 | 159.290 | 0.125 | 0.126 | 0.126 | 0.126 | 0.123 | 0.086 | 0.175 | 0.150 | 0.104 | 0.086 | 0.135 | 0.118 | 0.111 | 0.104 | 0.117 |
| meta1624 |  | 563.401 | 419.906 | 0.011 | 0.014 | 0.009 | 0.010 | 0.011 | 0.010 | 0.012 | 0.011 | 0.016 | 0.012 | 0.010 | 0.011 | 0.008 | 0.011 | 0.008 |
| meta1625 |  | 563.569 | 274.571 | 0.001 | 0.002 | 0.001 | 0.001 | 0.003 | 0.001 | 0.001 | 0.002 | 0.002 | 0.004 | 0.000 | 0.000 | 0.001 | 0.001 | 0.001 |
| meta1626 |  | 564.060 | 446.326 | 0.020 | 0.020 | 0.018 | 0.011 | 0.012 | 0.015 | 0.012 | 0.018 | 0.021 | 0.031 | 0.028 | 0.023 | 0.022 | 0.023 | 0.025 |
| meta1627 |  | 564.104 | 389.570 | 0.038 | 0.044 | 0.038 | 0.031 | 0.034 | 0.029 | 0.038 | 0.041 | 0.042 | 0.042 | 0.038 | 0.033 | 0.033 | 0.033 | 0.042 |
| meta1628 |  | 564.356 | 107.514 | 0.080 | 0.062 | 0.076 | 0.074 | 0.048 | 0.077 | 0.050 | 0.065 | 0.053 | 0.064 | 0.029 | 0.052 | 0.063 | 0.059 | 0.042 |
| meta1629 |  | 564.426 | 571.632 | 0.006 | 0.007 | 0.006 | 0.005 | 0.005 | 0.005 | 0.006 | 0.006 | 0.005 | 0.007 | 0.006 | 0.005 | 0.006 | 0.005 | 0.006 |
| meta1630 |  | 565.179 | 410.481 | 0.012 | 0.010 | 0.011 | 0.008 | 0.012 | 0.012 | 0.006 | 0.012 | 0.004 | 0.008 | 0.010 | 0.011 | 0.008 | 0.017 | 0.011 |
| meta1631 |  | 565.311 | 228.620 | 0.007 | 0.007 | 0.007 | 0.007 | 0.006 | 0.006 | 0.011 | 0.011 | 0.004 | 0.006 | 0.008 | 0.007 | 0.005 | 0.005 | 0.007 |
| meta1632 |  | 566.006 | 480.719 | 0.008 | 0.006 | 0.007 | 0.007 | 0.007 | 0.004 | 0.006 | 0.005 | 0.005 | 0.005 | 0.006 | 0.009 | 0.006 | 0.007 | 0.007 |
| meta1633 |  | 566.138 | 419.361 | 1.053 | 1.135 | 1.117 | 0.964 | 1.090 | 0.952 | 1.121 | 1.220 | 1.322 | 1.235 | 1.139 | 0.953 | 1.076 | 0.921 | 1.158 |
| meta1634 |  | 566.378 | 145.732 | 0.008 | 0.010 | 0.009 | 0.018 | 0.018 | 0.013 | 0.012 | 0.013 | 0.009 | 0.006 | 0.014 | 0.008 | 0.006 | 0.008 | 0.010 |
| meta1635 |  | 566.393 | 434.718 | 0.011 | 0.012 | 0.011 | 0.010 | 0.012 | 0.011 | 0.009 | 0.015 | 0.014 | 0.014 | 0.009 | 0.009 | 0.013 | 0.008 | 0.013 |
| meta1636 |  | 566.416 | 168.562 | 0.006 | 0.005 | 0.006 | 0.004 | 0.003 | 0.007 | 0.003 | 0.005 | 0.009 | 0.005 | 0.007 | 0.006 | 0.002 | 0.002 | 0.005 |
| meta1637 |  | 567.134 | 201.652 | 0.020 | 0.015 | 0.012 | 0.014 | 0.018 | 0.011 | 0.014 | 0.012 | 0.006 | 0.011 | 0.017 | 0.014 | 0.013 | 0.016 | 0.013 |
| meta1638 |  | 567.279 | 461.438 | 0.009 | 0.008 | 0.009 | 0.008 | 0.008 | 0.009 | 0.009 | 0.006 | 0.007 | 0.011 | 0.008 | 0.010 | 0.010 | 0.006 | 0.011 |
| meta1639 |  | 567.282 | 40.684 | 0.006 | 0.008 | 0.008 | 0.010 | 0.011 | 0.010 | 0.009 | 0.008 | 0.007 | 0.008 | 0.011 | 0.009 | 0.010 | 0.010 | 0.015 |
| meta1640 |  | 568.137 | 434.642 | 4.825 | 5.130 | 5.045 | 4.331 | 4.774 | 4.615 | 5.174 | 6.018 | 6.275 | 6.200 | 5.440 | 5.024 | 5.701 | 4.369 | 5.392 |
| meta1641 |  | 568.136 | 418.656 | 0.133 | 0.127 | 0.119 | 0.111 | 0.124 | 0.104 | 0.132 | 0.141 | 0.152 | 0.141 | 0.131 | 0.113 | 0.130 | 0.112 | 0.135 |
| meta1642 |  | 568.182 | 411.975 | 0.040 | 0.039 | 0.043 | 0.047 | 0.034 | 0.040 | 0.040 | 0.036 | 0.033 | 0.040 | 0.039 | 0.037 | 0.037 | 0.028 | 0.031 |
| meta1643 |  | 568.418 | 156.396 | 0.006 | 0.007 | 0.007 | 0.007 | 0.009 | 0.007 | 0.008 | 0.010 | 0.010 | 0.011 | 0.011 | 0.020 | 0.008 | 0.010 | 0.014 |
| meta1644 |  | 568.600 | 480.234 | 0.014 | 0.010 | 0.012 | 0.008 | 0.012 | 0.011 | 0.013 | 0.009 | 0.014 | 0.011 | 0.012 | 0.019 | 0.024 | 0.016 | 0.012 |
| meta1645 |  | 569.082 | 152.481 | 0.005 | 0.004 | 0.005 | 0.004 | 0.006 | 0.004 | 0.006 | 0.005 | 0.005 | 0.003 | 0.004 | 0.005 | 0.004 | 0.004 | 0.004 |
| meta1646 |  | 569.167 | 473.775 | 0.003 | 0.004 | 0.003 | 0.004 | 0.005 | 0.002 | 0.006 | 0.003 | 0.007 | 0.004 | 0.005 | 0.005 | 0.005 | 0.004 | 0.004 |
| meta1647 |  | 569.381 | 571.750 | 0.006 | 0.007 | 0.006 | 0.007 | 0.006 | 0.005 | 0.006 | 0.007 | 0.006 | 0.006 | 0.007 | 0.007 | 0.006 | 0.005 | 0.007 |
| meta1648 |  | 570.454 | 38.247 | 0.007 | 0.009 | 0.010 | 0.008 | 0.005 | 0.009 | 0.026 | 0.008 | 0.010 | 0.007 | 0.007 | 0.008 | 0.008 | 0.006 | 0.007 |
| meta1649 |  | 570.758 | 287.531 | 0.005 | 0.004 | 0.005 | 0.007 | 0.007 | 0.006 | 0.006 | 0.006 | 0.010 | 0.008 | 0.006 | 0.007 | 0.012 | 0.007 | 0.006 |
| meta1650 |  | 570.844 | 318.454 | 0.048 | 0.047 | 0.052 | 0.042 | 0.049 | 0.055 | 0.048 | 0.041 | 0.062 | 0.045 | 0.045 | 0.041 | 0.055 | 0.062 | 0.045 |
| meta1651 |  | 571.134 | 152.578 | 0.007 | 0.006 | 0.006 | 0.006 | 0.008 | 0.004 | 0.010 | 0.005 | 0.005 | 0.003 | 0.004 | 0.006 | 0.005 | 0.004 | 0.005 |
| meta1652 |  | 571.354 | 276.102 | 0.058 | 0.061 | 0.053 | 0.028 | 0.069 | 0.039 | 0.198 | 0.017 | 0.045 | 0.035 | 0.015 | 0.037 | 0.080 | 0.028 | 0.054 |
| meta1653 | ADP-glucose | 572.088 | 391.833 | 0.010 | 0.008 | 0.008 | 0.007 | 0.011 | 0.006 | 0.010 | 0.010 | 0.009 | 0.006 | 0.008 | 0.009 | 0.010 | 0.007 | 0.010 |
| meta1654 |  | 572.140 | 220.104 | 0.003 | 0.004 | 0.004 | 0.004 | 0.001 | 0.005 | 0.005 | 0.004 | 0.003 | 0.004 | 0.002 | 0.002 | 0.002 | 0.004 | 0.003 |
| meta1655 |  | 572.331 | 145.705 | 0.009 | 0.007 | 0.008 | 0.008 | 0.014 | 0.008 | 0.008 | 0.012 | 0.010 | 0.016 | 0.010 | 0.006 | 0.010 | 0.017 | 0.007 |
| meta1656 |  | 573.301 | 232.585 | 0.070 | 0.068 | 0.070 | 0.063 | 0.058 | 0.043 | 0.107 | 0.101 | 0.041 | 0.064 | 0.069 | 0.066 | 0.050 | 0.066 | 0.045 |
| meta1657 |  | 573.355 | 45.999 | 0.018 | 0.024 | 0.021 | 0.145 | 0.082 | 0.045 | 0.016 | 0.011 | 0.026 | 0.008 | 0.004 | 0.011 | 0.006 | 0.007 | 0.011 |
| meta1658 |  | 573.485 | 44.707 | 0.010 | 0.013 | 0.013 | 0.009 | 0.015 | 0.012 | 0.013 | 0.023 | 0.019 | 0.018 | 0.032 | 0.027 | 0.012 | 0.011 | 0.015 |
| meta1659 |  | 574.091 | 380.780 | 0.226 | 0.188 | 0.201 | 0.159 | 0.238 | 0.122 | 0.284 | 0.276 | 0.194 | 0.167 | 0.136 | 0.137 | 0.186 | 0.107 | 0.248 |
| meta1660 |  | 574.108 | 152.578 | 0.003 | 0.003 | 0.003 | 0.003 | 0.004 | 0.003 | 0.005 | 0.004 | 0.002 | 0.002 | 0.003 | 0.003 | 0.003 | 0.002 | 0.003 |
| meta1661 |  | 574.315 | 175.212 | 0.089 | 0.086 | 0.097 | 0.081 | 0.108 | 0.069 | 0.105 | 0.082 | 0.078 | 0.086 | 0.079 | 0.064 | 0.084 | 0.085 | 0.104 |
| meta1662 |  | 574.349 | 184.269 | 0.026 | 0.022 | 0.032 | 0.004 | 0.010 | 0.038 | 0.032 | 0.027 | 0.008 | 0.041 | 0.025 | 0.010 | 0.038 | 0.035 | 0.013 |
| meta1663 |  | 574.718 | 287.257 | 0.027 | 0.021 | 0.021 | 0.029 | 0.030 | 0.023 | 0.029 | 0.029 | 0.035 | 0.029 | 0.027 | 0.026 | 0.038 | 0.026 | 0.026 |
| meta1664 |  | 575.087 | 409.633 | 0.020 | 0.019 | 0.017 | 0.013 | 0.015 | 0.014 | 0.020 | 0.020 | 0.024 | 0.018 | 0.021 | 0.023 | 0.021 | 0.015 | 0.017 |
| meta1665 |  | 575.122 | 202.147 | 0.398 | 0.394 | 0.359 | 0.382 | 0.473 | 0.313 | 0.450 | 0.366 | 0.279 | 0.324 | 0.376 | 0.391 | 0.350 | 0.392 | 0.375 |
| meta1666 |  | 575.500 | 180.396 | 0.032 | 0.034 | 0.030 | 0.012 | 0.006 | 0.005 | 0.008 | 0.033 | 0.021 | 0.016 | 0.035 | 0.036 | 0.016 | 0.010 | 0.009 |
| meta1667 |  | 576.101 | 442.561 | 0.020 | 0.016 | 0.015 | 0.012 | 0.012 | 0.016 | 0.013 | 0.014 | 0.020 | 0.017 | 0.015 | 0.012 | 0.015 | 0.012 | 0.024 |
| meta1668 |  | 576.233 | 184.785 | 0.023 | 0.018 | 0.022 | 0.015 | 0.022 | 0.018 | 0.022 | 0.023 | 0.018 | 0.029 | 0.021 | 0.018 | 0.022 | 0.022 | 0.021 |
| meta1669 |  | 576.304 | 47.180 | 0.016 | 0.022 | 0.020 | 0.019 | 0.030 | 0.020 | 0.024 | 0.020 | 0.019 | 0.020 | 0.021 | 0.022 | 0.018 | 0.017 | 0.029 |
| meta1670 |  | 576.460 | 27.742 | 0.106 | 0.133 | 0.132 | 0.120 | 0.124 | 0.122 | 0.112 | 0.134 | 0.145 | 0.119 | 0.115 | 0.120 | 0.117 | 0.112 | 0.117 |
| meta1671 |  | 576.504 | 31.453 | 0.020 | 0.020 | 0.013 | 0.068 | 0.070 | 0.073 | 0.057 | 0.066 | 0.064 | 0.120 | 0.077 | 0.081 | 0.042 | 0.007 | 0.054 |
| meta1672 |  | 576.698 | 288.086 | 0.162 | 0.166 | 0.171 | 0.182 | 0.201 | 0.163 | 0.181 | 0.183 | 0.223 | 0.204 | 0.198 | 0.177 | 0.227 | 0.196 | 0.191 |
| meta1673 |  | 577.065 | 423.257 | 0.047 | 0.052 | 0.054 | 0.053 | 0.043 | 0.039 | 0.051 | 0.045 | 0.057 | 0.046 | 0.048 | 0.039 | 0.044 | 0.044 | 0.051 |
| meta1674 |  | 577.517 | 46.391 | 0.489 | 0.534 | 0.494 | 0.186 | 0.112 | 0.082 | 0.128 | 0.399 | 0.150 | 0.202 | 0.394 | 0.603 | 0.187 | 0.092 | 0.218 |
| meta1675 |  | 578.190 | 343.503 | 0.011 | 0.012 | 0.012 | 0.010 | 0.012 | 0.017 | 0.012 | 0.014 | 0.009 | 0.011 | 0.011 | 0.018 | 0.010 | 0.008 | 0.006 |
| meta1676 |  | 578.328 | 175.144 | 0.030 | 0.023 | 0.024 | 0.030 | 0.045 | 0.023 | 0.051 | 0.031 | 0.029 | 0.023 | 0.042 | 0.016 | 0.020 | 0.034 | 0.024 |
| meta1677 |  | 578.390 | 173.409 | 0.011 | 0.020 | 0.011 | 0.021 | 0.025 | 0.024 | 0.035 | 0.038 | 0.020 | 0.026 | 0.021 | 0.025 | 0.031 | 0.015 | 0.017 |
| meta1678 |  | 579.079 | 277.803 | 0.004 | 0.004 | 0.003 | 0.003 | 0.005 | 0.003 | 0.007 | 0.005 | 0.003 | 0.002 | 0.004 | 0.003 | 0.003 | 0.003 | 0.005 |
| meta1679 |  | 579.148 | 278.637 | 0.010 | 0.015 | 0.015 | 0.013 | 0.014 | 0.010 | 0.013 | 0.013 | 0.014 | 0.012 | 0.014 | 0.012 | 0.013 | 0.012 | 0.015 |
| meta1680 |  | 579.592 | 480.454 | 0.002 | 0.003 | 0.003 | 0.002 | 0.002 | 0.001 | 0.002 | 0.003 | 0.002 | 0.003 | 0.003 | 0.005 | 0.004 | 0.004 | 0.002 |
| meta1681 |  | 580.158 | 371.943 | 0.054 | 0.050 | 0.042 | 0.048 | 0.059 | 0.048 | 0.058 | 0.058 | 0.027 | 0.052 | 0.063 | 0.049 | 0.033 | 0.033 | 0.035 |
| meta1682 |  | 580.358 | 155.692 | 0.106 | 0.116 | 0.114 | 0.189 | 0.178 | 0.131 | 0.096 | 0.042 | 0.129 | 0.075 | 0.051 | 0.031 | 0.070 | 0.097 | 0.078 |
| meta1683 |  | 580.430 | 167.658 | 0.024 | 0.027 | 0.029 | 0.022 | 0.015 | 0.011 | 0.019 | 0.031 | 0.020 | 0.018 | 0.026 | 0.024 | 0.014 | 0.029 | 0.020 |
| meta1684 |  | 581.130 | 203.522 | 0.024 | 0.020 | 0.018 | 0.021 | 0.024 | 0.018 | 0.020 | 0.020 | 0.019 | 0.017 | 0.022 | 0.023 | 0.019 | 0.017 | 0.025 |
| meta1685 |  | 581.236 | 34.205 | 0.004 | 0.006 | 0.004 | 0.006 | 0.007 | 0.012 | 0.008 | 0.010 | 0.006 | 0.005 | 0.003 | 0.003 | 0.006 | 0.007 | 0.004 |
| meta1686 |  | 581.306 | 229.619 | 0.021 | 0.022 | 0.021 | 0.014 | 0.018 | 0.012 | 0.023 | 0.030 | 0.013 | 0.022 | 0.018 | 0.023 | 0.025 | 0.028 | 0.015 |
| meta1687 |  | 581.363 | 318.770 | 0.021 | 0.023 | 0.024 | 0.022 | 0.022 | 0.022 | 0.022 | 0.023 | 0.024 | 0.019 | 0.022 | 0.021 | 0.025 | 0.025 | 0.022 |
| meta1688 |  | 582.161 | 474.979 | 0.212 | 0.248 | 0.235 | 0.230 | 0.245 | 0.159 | 0.233 | 0.246 | 0.259 | 0.282 | 0.244 | 0.208 | 0.218 | 0.203 | 0.239 |
| meta1689 |  | 582.293 | 170.248 | 0.035 | 0.046 | 0.045 | 0.060 | 0.057 | 0.036 | 0.046 | 0.046 | 0.051 | 0.053 | 0.056 | 0.062 | 0.037 | 0.052 | 0.062 |
| meta1690 |  | 582.421 | 171.835 | 0.066 | 0.074 | 0.068 | 0.075 | 0.085 | 0.051 | 0.122 | 0.104 | 0.065 | 0.074 | 0.105 | 0.090 | 0.065 | 0.056 | 0.135 |
| meta1691 |  | 582.544 | 275.662 | 0.021 | 0.018 | 0.016 | 0.019 | 0.021 | 0.016 | 0.020 | 0.027 | 0.020 | 0.022 | 0.020 | 0.013 | 0.024 | 0.017 | 0.017 |
| meta1692 |  | 583.045 | 275.462 | 0.003 | 0.006 | 0.003 | 0.005 | 0.004 | 0.003 | 0.005 | 0.005 | 0.003 | 0.008 | 0.005 | 0.004 | 0.004 | 0.007 | 0.004 |
| meta1693 |  | 583.147 | 455.901 | 0.194 | 0.181 | 0.182 | 0.158 | 0.170 | 0.156 | 0.197 | 0.208 | 0.227 | 0.215 | 0.184 | 0.160 | 0.203 | 0.147 | 0.198 |
| meta1694 |  | 583.141 | 364.411 | 0.028 | 0.029 | 0.025 | 0.022 | 0.016 | 0.018 | 0.028 | 0.025 | 0.031 | 0.024 | 0.021 | 0.023 | 0.032 | 0.014 | 0.024 |
| meta1695 |  | 583.321 | 228.590 | 0.153 | 0.149 | 0.144 | 0.122 | 0.131 | 0.101 | 0.217 | 0.194 | 0.087 | 0.106 | 0.154 | 0.135 | 0.092 | 0.105 | 0.134 |
| meta1696 |  | 584.074 | 412.638 | 0.014 | 0.016 | 0.014 | 0.011 | 0.020 | 0.009 | 0.013 | 0.017 | 0.014 | 0.017 | 0.022 | 0.020 | 0.012 | 0.014 | 0.010 |
| meta1697 | UDP-D-Galactose | 584.086 | 434.172 | 0.178 | 0.165 | 0.165 | 0.080 | 0.106 | 0.135 | 0.100 | 0.172 | 0.216 | 0.347 | 0.256 | 0.202 | 0.244 | 0.179 | 0.235 |
| meta1698 |  | 584.131 | 472.304 | 0.821 | 0.847 | 0.735 | 0.682 | 0.725 | 0.746 | 0.841 | 0.850 | 0.972 | 0.985 | 0.765 | 0.717 | 0.961 | 0.617 | 0.856 |
| meta1699 |  | 584.189 | 324.603 | 0.012 | 0.010 | 0.012 | 0.012 | 0.020 | 0.012 | 0.015 | 0.007 | 0.002 | 0.007 | 0.012 | 0.017 | 0.007 | 0.012 | 0.003 |
| meta1700 |  | 584.354 | 34.861 | 0.019 | 0.025 | 0.023 | 0.190 | 0.071 | 0.040 | 0.009 | 0.012 | 0.029 | 0.006 | 0.009 | 0.004 | 0.003 | 0.009 | 0.003 |
| meta1701 |  | 584.450 | 157.219 | 0.002 | 0.002 | 0.002 | 0.001 | 0.003 | 0.002 | 0.003 | 0.004 | 0.003 | 0.003 | 0.004 | 0.006 | 0.004 | 0.003 | 0.005 |
| meta1702 |  | 584.471 | 38.106 | 0.008 | 0.008 | 0.007 | 0.014 | 0.016 | 0.009 | 0.014 | 0.009 | 0.009 | 0.017 | 0.011 | 0.008 | 0.007 | 0.008 | 0.009 |
| meta1703 |  | 585.050 | 152.578 | 0.006 | 0.006 | 0.007 | 0.007 | 0.009 | 0.004 | 0.008 | 0.006 | 0.006 | 0.005 | 0.005 | 0.005 | 0.006 | 0.005 | 0.006 |
| meta1704 |  | 585.368 | 196.406 | 0.003 | 0.004 | 0.005 | 0.002 | 0.003 | 0.004 | 0.014 | 0.002 | 0.002 | 0.002 | 0.002 | 0.002 | 0.003 | 0.002 | 0.004 |
| meta1705 |  | 586.041 | 446.776 | 0.008 | 0.008 | 0.007 | 0.005 | 0.005 | 0.006 | 0.005 | 0.007 | 0.009 | 0.011 | 0.011 | 0.009 | 0.009 | 0.009 | 0.010 |
| meta1706 |  | 586.152 | 480.288 | 0.022 | 0.020 | 0.024 | 0.019 | 0.025 | 0.018 | 0.019 | 0.030 | 0.020 | 0.032 | 0.022 | 0.026 | 0.031 | 0.018 | 0.023 |
| meta1707 |  | 586.348 | 146.941 | 0.004 | 0.006 | 0.005 | 0.011 | 0.009 | 0.009 | 0.006 | 0.008 | 0.005 | 0.006 | 0.009 | 0.009 | 0.005 | 0.005 | 0.010 |
| meta1708 |  | 587.117 | 420.041 | 0.224 | 0.244 | 0.245 | 0.243 | 0.247 | 0.177 | 0.227 | 0.250 | 0.265 | 0.227 | 0.254 | 0.211 | 0.208 | 0.209 | 0.239 |
| meta1709 |  | 587.112 | 152.603 | 0.005 | 0.005 | 0.005 | 0.005 | 0.006 | 0.005 | 0.007 | 0.005 | 0.004 | 0.003 | 0.004 | 0.004 | 0.004 | 0.004 | 0.005 |
| meta1710 |  | 587.316 | 230.370 | 0.004 | 0.004 | 0.004 | 0.002 | 0.003 | 0.002 | 0.005 | 0.005 | 0.003 | 0.004 | 0.004 | 0.004 | 0.004 | 0.003 | 0.003 |
| meta1711 |  | 587.364 | 46.971 | 0.037 | 0.044 | 0.039 | 0.223 | 0.113 | 0.064 | 0.028 | 0.009 | 0.031 | 0.005 | 0.005 | 0.012 | 0.009 | 0.009 | 0.008 |
| meta1712 |  | 590.086 | 152.578 | 0.011 | 0.011 | 0.014 | 0.012 | 0.014 | 0.012 | 0.016 | 0.010 | 0.009 | 0.008 | 0.010 | 0.011 | 0.011 | 0.010 | 0.012 |
| meta1713 |  | 590.118 | 435.477 | 0.847 | 0.933 | 0.940 | 0.949 | 0.936 | 0.794 | 0.904 | 0.989 | 1.015 | 0.851 | 0.919 | 0.810 | 0.840 | 0.759 | 0.858 |
| meta1714 |  | 590.179 | 365.689 | 0.030 | 0.024 | 0.033 | 0.023 | 0.021 | 0.019 | 0.025 | 0.019 | 0.020 | 0.021 | 0.032 | 0.030 | 0.012 | 0.020 | 0.027 |
| meta1715 |  | 590.423 | 38.106 | 0.036 | 0.042 | 0.043 | 0.236 | 0.131 | 0.133 | 0.057 | 0.025 | 0.038 | 0.028 | 0.021 | 0.016 | 0.020 | 0.018 | 0.029 |
| meta1716 |  | 590.608 | 287.941 | 0.016 | 0.016 | 0.016 | 0.019 | 0.021 | 0.017 | 0.018 | 0.019 | 0.021 | 0.019 | 0.017 | 0.017 | 0.022 | 0.019 | 0.018 |
| meta1717 |  | 591.269 | 162.993 | 0.006 | 0.006 | 0.005 | 0.001 | 0.002 | 0.004 | 0.005 | 0.008 | 0.002 | 0.009 | 0.005 | 0.005 | 0.005 | 0.008 | 0.005 |
| meta1718 |  | 592.205 | 341.997 | 0.014 | 0.016 | 0.015 | 0.013 | 0.019 | 0.021 | 0.016 | 0.015 | 0.009 | 0.014 | 0.017 | 0.020 | 0.014 | 0.010 | 0.011 |
| meta1719 |  | 592.329 | 168.933 | 0.010 | 0.014 | 0.016 | 0.018 | 0.023 | 0.016 | 0.018 | 0.016 | 0.018 | 0.015 | 0.014 | 0.014 | 0.014 | 0.016 | 0.017 |
| meta1720 |  | 592.456 | 32.293 | 0.010 | 0.012 | 0.006 | 0.034 | 0.007 | 0.017 | 0.009 | 0.025 | 0.008 | 0.007 | 0.019 | 0.013 | 0.013 | 0.013 | 0.015 |
| meta1721 |  | 592.507 | 30.754 | 0.008 | 0.011 | 0.011 | 0.012 | 0.007 | 0.045 | 0.012 | 0.019 | 0.011 | 0.006 | 0.012 | 0.009 | 0.004 | 0.004 | 0.005 |
| meta1722 |  | 592.826 | 318.444 | 0.021 | 0.021 | 0.023 | 0.019 | 0.021 | 0.023 | 0.021 | 0.019 | 0.025 | 0.020 | 0.020 | 0.019 | 0.026 | 0.027 | 0.020 |
| meta1723 |  | 593.085 | 427.217 | 0.022 | 0.023 | 0.023 | 0.023 | 0.023 | 0.018 | 0.028 | 0.027 | 0.023 | 0.015 | 0.019 | 0.018 | 0.022 | 0.018 | 0.020 |
| meta1724 |  | 593.188 | 362.081 | 0.031 | 0.036 | 0.027 | 0.025 | 0.027 | 0.018 | 0.049 | 0.032 | 0.015 | 0.038 | 0.049 | 0.034 | 0.019 | 0.014 | 0.046 |
| meta1725 |  | 594.114 | 467.899 | 0.052 | 0.041 | 0.052 | 0.050 | 0.048 | 0.055 | 0.061 | 0.065 | 0.074 | 0.066 | 0.055 | 0.068 | 0.073 | 0.043 | 0.059 |
| meta1726 |  | 594.306 | 174.693 | 0.044 | 0.056 | 0.040 | 0.078 | 0.066 | 0.034 | 0.071 | 0.053 | 0.043 | 0.037 | 0.043 | 0.041 | 0.029 | 0.056 | 0.058 |
| meta1727 |  | 594.374 | 152.865 | 0.129 | 0.135 | 0.140 | 0.227 | 0.256 | 0.156 | 0.111 | 0.038 | 0.171 | 0.093 | 0.054 | 0.037 | 0.079 | 0.128 | 0.096 |
| meta1728 |  | 595.122 | 278.997 | 0.004 | 0.007 | 0.005 | 0.005 | 0.005 | 0.003 | 0.005 | 0.005 | 0.006 | 0.005 | 0.005 | 0.005 | 0.006 | 0.005 | 0.006 |
| meta1729 |  | 595.146 | 430.874 | 0.031 | 0.032 | 0.034 | 0.029 | 0.050 | 0.026 | 0.040 | 0.035 | 0.044 | 0.035 | 0.062 | 0.036 | 0.043 | 0.028 | 0.033 |
| meta1730 |  | 595.282 | 232.420 | 0.009 | 0.010 | 0.010 | 0.008 | 0.008 | 0.006 | 0.015 | 0.012 | 0.006 | 0.008 | 0.010 | 0.008 | 0.006 | 0.009 | 0.007 |
| meta1731 |  | 596.073 | 380.887 | 0.021 | 0.020 | 0.019 | 0.017 | 0.026 | 0.010 | 0.028 | 0.026 | 0.021 | 0.014 | 0.013 | 0.013 | 0.015 | 0.012 | 0.023 |
| meta1732 |  | 596.353 | 227.913 | 0.045 | 0.045 | 0.062 | 0.074 | 0.078 | 0.060 | 0.031 | 0.029 | 0.064 | 0.026 | 0.023 | 0.017 | 0.039 | 0.038 | 0.033 |
| meta1733 |  | 596.359 | 47.350 | 0.014 | 0.015 | 0.015 | 0.063 | 0.034 | 0.022 | 0.009 | 0.005 | 0.011 | 0.003 | 0.002 | 0.008 | 0.005 | 0.004 | 0.004 |
| meta1734 |  | 597.121 | 334.961 | 0.225 | 0.181 | 0.145 | 0.170 | 0.197 | 0.121 | 0.092 | 0.320 | 0.095 | 0.271 | 0.145 | 0.061 | 0.132 | 0.133 | 0.183 |
| meta1735 |  | 597.161 | 473.094 | 0.017 | 0.012 | 0.018 | 0.012 | 0.011 | 0.016 | 0.010 | 0.011 | 0.019 | 0.017 | 0.019 | 0.022 | 0.019 | 0.013 | 0.011 |
| meta1736 |  | 597.337 | 286.864 | 0.189 | 0.206 | 0.202 | 0.210 | 0.210 | 0.180 | 0.210 | 0.206 | 0.229 | 0.211 | 0.210 | 0.193 | 0.218 | 0.196 | 0.207 |
| meta1737 | Uridine 5'-diphosphoglucuronic acid (UDP-D-glucuronate) | 598.065 | 467.260 | 0.031 | 0.023 | 0.026 | 0.012 | 0.023 | 0.018 | 0.018 | 0.042 | 0.038 | 0.043 | 0.038 | 0.032 | 0.057 | 0.024 | 0.043 |
| meta1738 |  | 598.284 | 46.307 | 0.003 | 0.004 | 0.005 | 0.005 | 0.007 | 0.006 | 0.008 | 0.005 | 0.005 | 0.006 | 0.007 | 0.006 | 0.006 | 0.004 | 0.007 |
| meta1739 |  | 598.315 | 167.686 | 0.020 | 0.032 | 0.027 | 0.039 | 0.040 | 0.030 | 0.062 | 0.052 | 0.023 | 0.027 | 0.034 | 0.036 | 0.038 | 0.037 | 0.038 |
| meta1740 |  | 598.428 | 162.554 | 0.004 | 0.004 | 0.004 | 0.004 | 0.004 | 0.008 | 0.006 | 0.009 | 0.002 | 0.006 | 0.003 | 0.003 | 0.003 | 0.002 | 0.003 |
| meta1741 |  | 598.430 | 186.029 | 0.002 | 0.003 | 0.003 | 0.004 | 0.004 | 0.003 | 0.002 | 0.003 | 0.004 | 0.003 | 0.002 | 0.004 | 0.003 | 0.002 | 0.003 |
| meta1742 |  | 599.316 | 229.649 | 0.038 | 0.038 | 0.035 | 0.027 | 0.032 | 0.020 | 0.042 | 0.057 | 0.024 | 0.037 | 0.032 | 0.042 | 0.044 | 0.046 | 0.034 |
| meta1743 |  | 599.388 | 47.350 | 0.005 | 0.004 | 0.005 | 0.007 | 0.005 | 0.005 | 0.007 | 0.007 | 0.009 | 0.006 | 0.004 | 0.022 | 0.006 | 0.006 | 0.006 |
| meta1744 |  | 599.500 | 44.396 | 0.044 | 0.064 | 0.062 | 0.046 | 0.050 | 0.033 | 0.040 | 0.063 | 0.052 | 0.045 | 0.069 | 0.079 | 0.043 | 0.035 | 0.047 |
| meta1745 |  | 599.501 | 120.267 | 0.120 | 0.095 | 0.105 | 0.021 | 0.011 | 0.006 | 0.013 | 0.076 | 0.025 | 0.042 | 0.099 | 0.072 | 0.049 | 0.011 | 0.014 |
| meta1746 |  | 599.500 | 176.463 | 0.135 | 0.131 | 0.146 | 0.065 | 0.041 | 0.025 | 0.048 | 0.129 | 0.083 | 0.098 | 0.149 | 0.159 | 0.062 | 0.057 | 0.068 |
| meta1747 |  | 600.325 | 172.113 | 0.030 | 0.033 | 0.034 | 0.038 | 0.058 | 0.032 | 0.080 | 0.047 | 0.030 | 0.028 | 0.037 | 0.038 | 0.037 | 0.031 | 0.053 |
| meta1748 |  | 600.465 | 39.492 | 0.021 | 0.019 | 0.020 | 0.021 | 0.023 | 0.020 | 0.051 | 0.018 | 0.016 | 0.026 | 0.020 | 0.016 | 0.014 | 0.013 | 0.017 |
| meta1749 |  | 601.066 | 164.387 | 0.015 | 0.018 | 0.019 | 0.015 | 0.020 | 0.016 | 0.014 | 0.008 | 0.028 | 0.009 | 0.015 | 0.014 | 0.022 | 0.016 | 0.018 |
| meta1750 |  | 601.137 | 417.439 | 0.127 | 0.122 | 0.111 | 0.102 | 0.117 | 0.106 | 0.129 | 0.131 | 0.120 | 0.112 | 0.156 | 0.169 | 0.069 | 0.130 | 0.094 |
| meta1751 |  | 601.332 | 228.590 | 0.236 | 0.240 | 0.234 | 0.214 | 0.221 | 0.167 | 0.365 | 0.329 | 0.140 | 0.165 | 0.257 | 0.240 | 0.146 | 0.160 | 0.216 |
| meta1752 |  | 601.332 | 475.431 | 0.018 | 0.018 | 0.016 | 0.018 | 0.017 | 0.017 | 0.020 | 0.017 | 0.021 | 0.018 | 0.020 | 0.020 | 0.021 | 0.027 | 0.019 |
| meta1753 |  | 601.370 | 47.051 | 0.019 | 0.023 | 0.025 | 0.089 | 0.050 | 0.028 | 0.014 | 0.006 | 0.016 | 0.005 | 0.005 | 0.008 | 0.007 | 0.005 | 0.006 |
| meta1754 |  | 602.140 | 371.913 | 0.027 | 0.029 | 0.026 | 0.028 | 0.030 | 0.027 | 0.028 | 0.028 | 0.021 | 0.028 | 0.030 | 0.028 | 0.019 | 0.024 | 0.022 |
| meta1755 |  | 602.392 | 222.360 | 0.005 | 0.007 | 0.007 | 0.002 | 0.002 | 0.002 | 0.002 | 0.002 | 0.001 | 0.001 | 0.001 | 0.015 | 0.018 | 0.015 | 0.033 |
| meta1756 |  | 603.064 | 164.326 | 0.059 | 0.063 | 0.072 | 0.055 | 0.080 | 0.059 | 0.071 | 0.037 | 0.103 | 0.050 | 0.064 | 0.062 | 0.080 | 0.070 | 0.067 |
| meta1757 | Tyr-Thr | 603.220 | 386.140 | 0.028 | 0.027 | 0.026 | 0.028 | 0.038 | 0.023 | 0.030 | 0.034 | 0.022 | 0.029 | 0.037 | 0.028 | 0.042 | 0.036 | 0.027 |
| meta1758 |  | 603.352 | 163.176 | 0.022 | 0.029 | 0.035 | 0.016 | 0.009 | 0.032 | 0.037 | 0.072 | 0.016 | 0.056 | 0.028 | 0.023 | 0.044 | 0.054 | 0.025 |
| meta1759 |  | 603.532 | 179.074 | 0.014 | 0.017 | 0.015 | 0.003 | 0.002 | 0.001 | 0.002 | 0.018 | 0.006 | 0.007 | 0.012 | 0.020 | 0.007 | 0.002 | 0.003 |
| meta1760 |  | 603.532 | 46.307 | 0.156 | 0.165 | 0.146 | 0.050 | 0.041 | 0.024 | 0.037 | 0.130 | 0.065 | 0.056 | 0.158 | 0.172 | 0.054 | 0.023 | 0.058 |
| meta1761 |  | 604.492 | 31.205 | 0.006 | 0.006 | 0.007 | 0.002 | 0.005 | 0.007 | 0.005 | 0.011 | 0.005 | 0.005 | 0.004 | 0.008 | 0.003 | 0.008 | 0.004 |
| meta1762 |  | 604.600 | 34.042 | 0.015 | 0.019 | 0.011 | 0.002 | 0.001 | 0.000 | 0.001 | 0.008 | 0.002 | 0.005 | 0.008 | 0.021 | 0.003 | 0.001 | 0.002 |
| meta1763 |  | 605.080 | 462.769 | 0.012 | 0.007 | 0.008 | 0.044 | 0.011 | 0.007 | 0.053 | 0.047 | 0.055 | 0.053 | 0.043 | 0.040 | 0.010 | 0.037 | 0.051 |
| meta1764 |  | 605.128 | 455.945 | 0.043 | 0.038 | 0.044 | 0.039 | 0.044 | 0.040 | 0.047 | 0.047 | 0.051 | 0.050 | 0.041 | 0.037 | 0.046 | 0.034 | 0.047 |
| meta1765 |  | 606.090 | 435.146 | 0.149 | 0.164 | 0.156 | 0.155 | 0.164 | 0.135 | 0.160 | 0.167 | 0.174 | 0.146 | 0.156 | 0.138 | 0.144 | 0.128 | 0.144 |
| meta1766 |  | 606.111 | 472.331 | 0.105 | 0.113 | 0.103 | 0.090 | 0.099 | 0.089 | 0.101 | 0.099 | 0.111 | 0.140 | 0.096 | 0.100 | 0.108 | 0.081 | 0.110 |
| meta1767 |  | 606.163 | 409.825 | 0.498 | 0.505 | 0.470 | 0.375 | 0.318 | 0.354 | 0.333 | 0.453 | 0.577 | 0.640 | 0.606 | 0.665 | 0.566 | 0.439 | 0.420 |
| meta1768 |  | 606.446 | 165.935 | 0.009 | 0.009 | 0.008 | 0.009 | 0.007 | 0.005 | 0.006 | 0.010 | 0.005 | 0.005 | 0.009 | 0.007 | 0.003 | 0.004 | 0.005 |
| meta1769 |  | 607.245 | 162.561 | 0.010 | 0.010 | 0.010 | 0.002 | 0.003 | 0.008 | 0.010 | 0.015 | 0.005 | 0.014 | 0.011 | 0.011 | 0.012 | 0.014 | 0.008 |
| meta1770 |  | 608.209 | 364.196 | 0.116 | 0.109 | 0.119 | 0.082 | 0.133 | 0.161 | 0.114 | 0.118 | 0.066 | 0.109 | 0.125 | 0.144 | 0.088 | 0.065 | 0.068 |
| meta1771 |  | 608.389 | 152.450 | 0.042 | 0.050 | 0.054 | 0.068 | 0.077 | 0.051 | 0.061 | 0.041 | 0.065 | 0.039 | 0.043 | 0.031 | 0.036 | 0.034 | 0.049 |
| meta1772 |  | 608.485 | 41.197 | 0.005 | 0.008 | 0.006 | 0.011 | 0.019 | 0.010 | 0.010 | 0.010 | 0.009 | 0.006 | 0.011 | 0.015 | 0.007 | 0.017 | 0.009 |
| meta1773 |  | 608.699 | 288.606 | 0.004 | 0.005 | 0.005 | 0.005 | 0.006 | 0.005 | 0.005 | 0.004 | 0.007 | 0.006 | 0.007 | 0.006 | 0.007 | 0.006 | 0.006 |
| meta1774 |  | 609.163 | 423.879 | 1.202 | 1.205 | 1.273 | 0.968 | 0.725 | 0.901 | 0.856 | 1.055 | 1.442 | 1.262 | 1.299 | 1.518 | 1.448 | 1.163 | 1.002 |
| meta1775 |  | 609.311 | 477.666 | 0.052 | 0.038 | 0.039 | 0.036 | 0.060 | 0.034 | 0.049 | 0.058 | 0.036 | 0.047 | 0.048 | 0.049 | 0.049 | 0.024 | 0.066 |
| meta1776 |  | 609.378 | 47.221 | 0.026 | 0.042 | 0.036 | 0.166 | 0.096 | 0.060 | 0.020 | 0.006 | 0.027 | 0.005 | 0.007 | 0.012 | 0.010 | 0.008 | 0.006 |
| meta1777 |  | 610.369 | 229.649 | 0.055 | 0.059 | 0.074 | 0.091 | 0.084 | 0.077 | 0.036 | 0.037 | 0.088 | 0.039 | 0.031 | 0.025 | 0.050 | 0.051 | 0.041 |
| meta1778 |  | 610.537 | 31.992 | 0.081 | 0.059 | 0.068 | 0.007 | 0.007 | 0.009 | 0.012 | 0.030 | 0.013 | 0.022 | 0.044 | 0.044 | 0.021 | 0.003 | 0.010 |
| meta1779 |  | 611.023 | 434.933 | 0.009 | 0.009 | 0.009 | 0.006 | 0.007 | 0.007 | 0.005 | 0.009 | 0.010 | 0.013 | 0.012 | 0.010 | 0.011 | 0.010 | 0.011 |
| meta1780 |  | 612.068 | 152.520 | 0.009 | 0.012 | 0.010 | 0.011 | 0.011 | 0.010 | 0.012 | 0.008 | 0.009 | 0.007 | 0.010 | 0.009 | 0.010 | 0.009 | 0.011 |
| meta1781 |  | 612.125 | 466.640 | 0.275 | 0.234 | 0.259 | 0.213 | 0.268 | 0.238 | 0.270 | 0.298 | 0.323 | 0.336 | 0.273 | 0.232 | 0.318 | 0.201 | 0.292 |
| meta1782 |  | 612.235 | 27.545 | 0.053 | 0.067 | 0.066 | 0.073 | 0.069 | 0.047 | 0.056 | 0.082 | 0.069 | 0.056 | 0.066 | 0.066 | 0.064 | 0.067 | 0.069 |
| meta1783 |  | 612.259 | 348.365 | 0.016 | 0.019 | 0.018 | 0.015 | 0.018 | 0.029 | 0.017 | 0.020 | 0.011 | 0.015 | 0.019 | 0.026 | 0.015 | 0.014 | 0.011 |
| meta1784 |  | 612.296 | 372.011 | 0.067 | 0.061 | 0.059 | 0.068 | 0.052 | 0.088 | 0.047 | 0.045 | 0.030 | 0.057 | 0.082 | 0.070 | 0.048 | 0.062 | 0.051 |
| meta1785 |  | 612.374 | 229.220 | 0.003 | 0.004 | 0.005 | 0.007 | 0.006 | 0.005 | 0.003 | 0.002 | 0.008 | 0.002 | 0.002 | 0.001 | 0.003 | 0.004 | 0.003 |
| meta1786 |  | 612.371 | 40.761 | 0.041 | 0.051 | 0.050 | 0.316 | 0.115 | 0.075 | 0.023 | 0.007 | 0.046 | 0.007 | 0.004 | 0.007 | 0.006 | 0.004 | 0.009 |
| meta1787 |  | 612.674 | 287.765 | 0.010 | 0.011 | 0.009 | 0.012 | 0.013 | 0.010 | 0.012 | 0.010 | 0.014 | 0.013 | 0.011 | 0.012 | 0.015 | 0.011 | 0.010 |
| meta1788 | Glutathione disulfide | 613.158 | 478.317 | 2.489 | 1.754 | 2.094 | 1.755 | 2.199 | 2.074 | 2.262 | 2.514 | 2.699 | 2.835 | 2.231 | 2.339 | 3.039 | 1.705 | 2.430 |
| meta1789 |  | 613.279 | 244.402 | 0.069 | 0.089 | 0.098 | 0.030 | 0.035 | 0.084 | 0.099 | 0.097 | 0.071 | 0.117 | 0.097 | 0.051 | 0.118 | 0.116 | 0.048 |
| meta1790 |  | 614.653 | 288.285 | 0.046 | 0.047 | 0.052 | 0.051 | 0.056 | 0.047 | 0.056 | 0.051 | 0.063 | 0.058 | 0.057 | 0.052 | 0.070 | 0.060 | 0.057 |
| meta1791 |  | 616.148 | 404.059 | 0.050 | 0.051 | 0.045 | 0.038 | 0.043 | 0.042 | 0.049 | 0.050 | 0.048 | 0.043 | 0.061 | 0.051 | 0.029 | 0.047 | 0.031 |
| meta1792 |  | 616.343 | 215.131 | 0.066 | 0.062 | 0.063 | 0.046 | 0.049 | 0.046 | 0.079 | 0.104 | 0.037 | 0.049 | 0.079 | 0.090 | 0.044 | 0.058 | 0.065 |
| meta1793 |  | 616.354 | 152.828 | 0.022 | 0.029 | 0.023 | 0.041 | 0.055 | 0.025 | 0.028 | 0.011 | 0.028 | 0.016 | 0.012 | 0.012 | 0.015 | 0.023 | 0.022 |
| meta1794 |  | 616.407 | 217.740 | 0.004 | 0.004 | 0.004 | 0.001 | 0.001 | 0.001 | 0.001 | 0.001 | 0.003 | 0.001 | 0.000 | 0.017 | 0.012 | 0.008 | 0.022 |
| meta1795 |  | 617.298 | 433.568 | 0.022 | 0.026 | 0.028 | 0.022 | 0.023 | 0.027 | 0.021 | 0.026 | 0.020 | 0.028 | 0.032 | 0.035 | 0.027 | 0.026 | 0.028 |
| meta1796 |  | 617.385 | 45.544 | 0.011 | 0.018 | 0.019 | 0.103 | 0.060 | 0.032 | 0.013 | 0.011 | 0.022 | 0.006 | 0.008 | 0.010 | 0.004 | 0.004 | 0.006 |
| meta1797 |  | 617.916 | 612.544 | 0.072 | 0.073 | 0.046 | 0.125 | 0.051 | 0.052 | 0.131 | 0.058 | 0.071 | 0.067 | 0.082 | 0.051 | 0.067 | 0.073 | 0.081 |
| meta1798 |  | 618.081 | 396.749 | 0.031 | 0.031 | 0.031 | 0.023 | 0.038 | 0.030 | 0.022 | 0.042 | 0.022 | 0.031 | 0.034 | 0.054 | 0.024 | 0.033 | 0.030 |
| meta1799 |  | 618.112 | 427.309 | 0.016 | 0.017 | 0.018 | 0.013 | 0.015 | 0.010 | 0.021 | 0.024 | 0.020 | 0.015 | 0.022 | 0.018 | 0.015 | 0.012 | 0.015 |
| meta1800 |  | 618.357 | 228.388 | 0.020 | 0.024 | 0.017 | 0.015 | 0.018 | 0.018 | 0.027 | 0.026 | 0.012 | 0.015 | 0.019 | 0.026 | 0.013 | 0.015 | 0.022 |
| meta1801 |  | 619.103 | 334.971 | 0.087 | 0.079 | 0.077 | 0.079 | 0.093 | 0.066 | 0.053 | 0.115 | 0.058 | 0.093 | 0.078 | 0.040 | 0.068 | 0.068 | 0.088 |
| meta1802 |  | 619.157 | 202.206 | 0.010 | 0.008 | 0.008 | 0.009 | 0.011 | 0.007 | 0.009 | 0.007 | 0.006 | 0.006 | 0.010 | 0.011 | 0.008 | 0.008 | 0.007 |
| meta1803 |  | 619.184 | 362.081 | 0.102 | 0.107 | 0.100 | 0.079 | 0.116 | 0.054 | 0.132 | 0.090 | 0.044 | 0.102 | 0.133 | 0.079 | 0.055 | 0.048 | 0.135 |
| meta1804 |  | 620.268 | 184.698 | 0.004 | 0.004 | 0.004 | 0.002 | 0.003 | 0.004 | 0.005 | 0.005 | 0.003 | 0.004 | 0.005 | 0.004 | 0.003 | 0.004 | 0.005 |
| meta1805 |  | 620.434 | 56.005 | 0.273 | 0.234 | 0.234 | 0.177 | 0.220 | 0.370 | 0.271 | 0.303 | 0.232 | 0.195 | 0.229 | 0.175 | 0.269 | 0.288 | 0.172 |
| meta1806 |  | 620.489 | 27.918 | 0.044 | 0.060 | 0.055 | 0.052 | 0.055 | 0.041 | 0.050 | 0.069 | 0.064 | 0.043 | 0.053 | 0.065 | 0.056 | 0.058 | 0.055 |
| meta1807 |  | 620.594 | 33.665 | 0.027 | 0.039 | 0.024 | 0.007 | 0.005 | 0.002 | 0.005 | 0.025 | 0.004 | 0.006 | 0.023 | 0.041 | 0.005 | 0.007 | 0.008 |
| meta1808 |  | 621.330 | 240.532 | 0.002 | 0.003 | 0.002 | 0.007 | 0.010 | 0.009 | 0.002 | 0.002 | 0.005 | 0.004 | 0.002 | 0.003 | 0.002 | 0.003 | 0.003 |
| meta1809 |  | 621.358 | 278.965 | 0.008 | 0.009 | 0.010 | 0.008 | 0.007 | 0.008 | 0.006 | 0.007 | 0.006 | 0.014 | 0.014 | 0.012 | 0.008 | 0.013 | 0.008 |
| meta1810 |  | 622.085 | 472.353 | 0.010 | 0.010 | 0.012 | 0.012 | 0.009 | 0.009 | 0.010 | 0.009 | 0.013 | 0.016 | 0.012 | 0.011 | 0.016 | 0.008 | 0.012 |
| meta1811 |  | 622.206 | 365.183 | 0.402 | 0.434 | 0.367 | 0.346 | 0.455 | 0.432 | 0.421 | 0.351 | 0.255 | 0.411 | 0.444 | 0.476 | 0.298 | 0.312 | 0.277 |
| meta1812 |  | 622.336 | 171.609 | 0.017 | 0.020 | 0.016 | 0.025 | 0.022 | 0.020 | 0.029 | 0.029 | 0.021 | 0.022 | 0.027 | 0.017 | 0.015 | 0.011 | 0.038 |
| meta1813 |  | 622.404 | 150.430 | 0.070 | 0.081 | 0.073 | 0.105 | 0.125 | 0.062 | 0.071 | 0.047 | 0.090 | 0.056 | 0.054 | 0.042 | 0.051 | 0.063 | 0.077 |
| meta1814 |  | 623.086 | 152.578 | 0.012 | 0.012 | 0.010 | 0.010 | 0.016 | 0.009 | 0.011 | 0.013 | 0.007 | 0.006 | 0.007 | 0.009 | 0.007 | 0.008 | 0.010 |
| meta1815 |  | 623.449 | 48.132 | 0.009 | 0.014 | 0.011 | 0.040 | 0.029 | 0.047 | 0.012 | 0.057 | 0.061 | 0.033 | 0.069 | 0.008 | 0.039 | 0.035 | 0.043 |
| meta1816 |  | 623.500 | 43.692 | 0.051 | 0.043 | 0.037 | 0.044 | 0.064 | 0.030 | 0.052 | 0.057 | 0.055 | 0.040 | 0.068 | 0.071 | 0.041 | 0.035 | 0.049 |
| meta1817 |  | 623.501 | 119.182 | 0.383 | 0.241 | 0.287 | 0.074 | 0.049 | 0.040 | 0.061 | 0.222 | 0.108 | 0.148 | 0.220 | 0.260 | 0.164 | 0.067 | 0.063 |
| meta1818 |  | 623.612 | 34.434 | 0.008 | 0.009 | 0.006 | 0.005 | 0.004 | 0.001 | 0.002 | 0.006 | 0.003 | 0.002 | 0.006 | 0.010 | 0.001 | 0.003 | 0.002 |
| meta1819 |  | 624.172 | 444.764 | 0.052 | 0.046 | 0.049 | 0.033 | 0.027 | 0.035 | 0.037 | 0.045 | 0.072 | 0.068 | 0.056 | 0.057 | 0.065 | 0.042 | 0.047 |
| meta1820 |  | 624.384 | 225.029 | 0.016 | 0.016 | 0.017 | 0.028 | 0.024 | 0.016 | 0.012 | 0.012 | 0.022 | 0.010 | 0.010 | 0.007 | 0.010 | 0.009 | 0.012 |
| meta1821 | UDP-N-acetylglucosamine | 625.113 | 420.722 | 0.067 | 0.068 | 0.065 | 0.051 | 0.066 | 0.057 | 0.087 | 0.069 | 0.082 | 0.068 | 0.066 | 0.065 | 0.072 | 0.053 | 0.078 |
| meta1822 |  | 625.156 | 467.275 | 0.217 | 0.198 | 0.226 | 0.146 | 0.115 | 0.126 | 0.154 | 0.143 | 0.263 | 0.219 | 0.203 | 0.220 | 0.266 | 0.174 | 0.182 |
| meta1823 |  | 625.361 | 239.559 | 0.005 | 0.004 | 0.004 | 0.007 | 0.009 | 0.008 | 0.005 | 0.006 | 0.006 | 0.005 | 0.006 | 0.005 | 0.002 | 0.004 | 0.008 |
| meta1824 |  | 625.389 | 318.923 | 0.013 | 0.013 | 0.014 | 0.012 | 0.013 | 0.013 | 0.014 | 0.015 | 0.014 | 0.012 | 0.013 | 0.012 | 0.015 | 0.014 | 0.012 |
| meta1825 |  | 625.517 | 174.594 | 0.050 | 0.048 | 0.044 | 0.016 | 0.016 | 0.005 | 0.010 | 0.049 | 0.019 | 0.035 | 0.044 | 0.070 | 0.035 | 0.020 | 0.034 |
| meta1826 |  | 626.078 | 275.257 | 0.465 | 0.413 | 0.364 | 0.383 | 0.500 | 0.373 | 0.310 | 0.681 | 0.389 | 0.710 | 0.372 | 0.189 | 0.415 | 0.441 | 0.427 |
| meta1827 |  | 626.311 | 274.717 | 0.011 | 0.014 | 0.018 | 0.018 | 0.013 | 0.016 | 0.010 | 0.010 | 0.005 | 0.009 | 0.015 | 0.014 | 0.009 | 0.014 | 0.013 |
| meta1828 |  | 626.389 | 169.001 | 0.005 | 0.006 | 0.005 | 0.006 | 0.009 | 0.004 | 0.008 | 0.008 | 0.008 | 0.005 | 0.006 | 0.009 | 0.006 | 0.005 | 0.007 |
| meta1829 |  | 627.170 | 362.081 | 0.081 | 0.073 | 0.068 | 0.076 | 0.086 | 0.047 | 0.100 | 0.069 | 0.040 | 0.077 | 0.088 | 0.066 | 0.061 | 0.040 | 0.108 |
| meta1830 | 1-Stearoyl-2-arachidonoyl-sn-glycerol | 627.532 | 175.210 | 0.572 | 0.653 | 0.583 | 0.158 | 0.135 | 0.078 | 0.116 | 0.504 | 0.184 | 0.223 | 0.621 | 0.622 | 0.245 | 0.086 | 0.192 |
| meta1831 |  | 628.094 | 472.154 | 0.005 | 0.007 | 0.006 | 0.006 | 0.006 | 0.003 | 0.005 | 0.005 | 0.010 | 0.008 | 0.006 | 0.007 | 0.007 | 0.004 | 0.007 |
| meta1832 |  | 628.381 | 36.142 | 0.028 | 0.033 | 0.039 | 0.293 | 0.095 | 0.064 | 0.018 | 0.006 | 0.042 | 0.005 | 0.006 | 0.008 | 0.007 | 0.005 | 0.005 |
| meta1833 |  | 628.599 | 33.382 | 0.037 | 0.043 | 0.033 | 0.007 | 0.002 | 0.001 | 0.003 | 0.023 | 0.002 | 0.007 | 0.031 | 0.044 | 0.009 | 0.002 | 0.003 |
| meta1834 |  | 628.675 | 288.784 | 0.004 | 0.004 | 0.006 | 0.004 | 0.005 | 0.006 | 0.005 | 0.004 | 0.006 | 0.005 | 0.005 | 0.005 | 0.008 | 0.007 | 0.006 |
| meta1835 |  | 629.128 | 409.819 | 0.017 | 0.016 | 0.015 | 0.012 | 0.025 | 0.013 | 0.012 | 0.023 | 0.014 | 0.027 | 0.019 | 0.018 | 0.017 | 0.012 | 0.015 |
| meta1836 |  | 629.204 | 434.088 | 0.009 | 0.010 | 0.009 | 0.007 | 0.007 | 0.012 | 0.006 | 0.011 | 0.006 | 0.014 | 0.011 | 0.008 | 0.008 | 0.008 | 0.005 |
| meta1837 |  | 630.423 | 215.160 | 0.001 | 0.001 | 0.002 | 0.001 | 0.001 | 0.001 | 0.001 | 0.002 | 0.000 | 0.001 | 0.001 | 0.006 | 0.004 | 0.004 | 0.009 |
| meta1838 |  | 630.471 | 41.837 | 0.007 | 0.005 | 0.006 | 0.009 | 0.010 | 0.011 | 0.011 | 0.009 | 0.007 | 0.007 | 0.007 | 0.013 | 0.009 | 0.014 | 0.010 |
| meta1839 |  | 630.506 | 32.443 | 0.013 | 0.013 | 0.010 | 0.011 | 0.020 | 0.009 | 0.014 | 0.008 | 0.016 | 0.007 | 0.006 | 0.010 | 0.009 | 0.017 | 0.014 |
| meta1840 |  | 630.615 | 34.097 | 0.084 | 0.082 | 0.071 | 0.008 | 0.006 | 0.001 | 0.006 | 0.055 | 0.008 | 0.022 | 0.064 | 0.099 | 0.024 | 0.001 | 0.011 |
| meta1841 |  | 631.120 | 198.620 | 0.021 | 0.021 | 0.024 | 0.024 | 0.027 | 0.024 | 0.030 | 0.019 | 0.012 | 0.014 | 0.019 | 0.021 | 0.026 | 0.021 | 0.023 |
| meta1842 |  | 631.147 | 444.121 | 0.047 | 0.040 | 0.041 | 0.033 | 0.044 | 0.036 | 0.042 | 0.051 | 0.043 | 0.050 | 0.049 | 0.042 | 0.050 | 0.032 | 0.050 |
| meta1843 |  | 631.216 | 425.901 | 0.044 | 0.049 | 0.047 | 0.041 | 0.050 | 0.057 | 0.043 | 0.062 | 0.042 | 0.044 | 0.053 | 0.056 | 0.054 | 0.035 | 0.028 |
| meta1844 |  | 631.391 | 46.480 | 0.024 | 0.027 | 0.030 | 0.116 | 0.070 | 0.039 | 0.014 | 0.006 | 0.021 | 0.004 | 0.005 | 0.010 | 0.007 | 0.005 | 0.004 |
| meta1845 |  | 632.128 | 423.218 | 0.119 | 0.108 | 0.109 | 0.132 | 0.102 | 0.092 | 0.088 | 0.111 | 0.110 | 0.131 | 0.110 | 0.099 | 0.122 | 0.094 | 0.112 |
| meta1846 |  | 632.214 | 348.582 | 0.011 | 0.008 | 0.010 | 0.009 | 0.008 | 0.012 | 0.010 | 0.009 | 0.011 | 0.012 | 0.010 | 0.010 | 0.010 | 0.009 | 0.010 |
| meta1847 |  | 632.485 | 40.726 | 0.018 | 0.020 | 0.020 | 0.021 | 0.021 | 0.030 | 0.021 | 0.022 | 0.028 | 0.024 | 0.032 | 0.037 | 0.029 | 0.037 | 0.026 |
| meta1848 |  | 632.522 | 31.890 | 0.068 | 0.042 | 0.053 | 0.009 | 0.025 | 0.025 | 0.031 | 0.019 | 0.026 | 0.025 | 0.029 | 0.042 | 0.027 | 0.009 | 0.021 |
| meta1849 |  | 632.844 | 318.444 | 0.032 | 0.032 | 0.036 | 0.032 | 0.031 | 0.036 | 0.033 | 0.029 | 0.041 | 0.031 | 0.033 | 0.028 | 0.039 | 0.043 | 0.031 |
| meta1850 |  | 633.324 | 209.270 | 0.004 | 0.005 | 0.006 | 0.003 | 0.005 | 0.004 | 0.005 | 0.004 | 0.003 | 0.004 | 0.004 | 0.005 | 0.004 | 0.004 | 0.004 |
| meta1851 |  | 634.107 | 466.643 | 0.035 | 0.028 | 0.029 | 0.038 | 0.044 | 0.033 | 0.042 | 0.039 | 0.043 | 0.048 | 0.040 | 0.040 | 0.047 | 0.028 | 0.037 |
| meta1852 |  | 634.216 | 400.076 | 0.020 | 0.018 | 0.024 | 0.028 | 0.026 | 0.023 | 0.026 | 0.028 | 0.012 | 0.015 | 0.022 | 0.020 | 0.013 | 0.011 | 0.011 |
| meta1853 |  | 634.278 | 371.889 | 0.018 | 0.019 | 0.018 | 0.020 | 0.015 | 0.026 | 0.012 | 0.014 | 0.010 | 0.017 | 0.023 | 0.021 | 0.012 | 0.019 | 0.016 |
| meta1854 |  | 634.363 | 229.011 | 0.005 | 0.005 | 0.005 | 0.010 | 0.007 | 0.006 | 0.005 | 0.004 | 0.008 | 0.004 | 0.004 | 0.004 | 0.006 | 0.005 | 0.005 |
| meta1855 |  | 634.405 | 145.906 | 0.019 | 0.021 | 0.022 | 0.033 | 0.033 | 0.022 | 0.019 | 0.013 | 0.021 | 0.018 | 0.015 | 0.015 | 0.015 | 0.020 | 0.018 |
| meta1856 |  | 634.450 | 39.492 | 0.034 | 0.044 | 0.046 | 0.217 | 0.133 | 0.113 | 0.050 | 0.021 | 0.039 | 0.026 | 0.020 | 0.018 | 0.017 | 0.014 | 0.023 |
| meta1857 |  | 634.739 | 287.765 | 0.002 | 0.002 | 0.002 | 0.003 | 0.002 | 0.003 | 0.002 | 0.003 | 0.003 | 0.004 | 0.003 | 0.003 | 0.005 | 0.003 | 0.003 |
| meta1858 |  | 635.138 | 478.254 | 0.218 | 0.174 | 0.185 | 0.166 | 0.204 | 0.161 | 0.198 | 0.233 | 0.238 | 0.249 | 0.221 | 0.212 | 0.222 | 0.150 | 0.217 |
| meta1859 |  | 635.134 | 202.206 | 0.024 | 0.025 | 0.021 | 0.024 | 0.033 | 0.019 | 0.025 | 0.018 | 0.019 | 0.018 | 0.020 | 0.026 | 0.019 | 0.024 | 0.023 |
| meta1860 |  | 635.354 | 184.082 | 0.007 | 0.010 | 0.007 | 0.003 | 0.005 | 0.009 | 0.011 | 0.011 | 0.003 | 0.011 | 0.009 | 0.004 | 0.007 | 0.006 | 0.005 |
| meta1861 |  | 636.312 | 146.439 | 0.005 | 0.006 | 0.007 | 0.010 | 0.009 | 0.005 | 0.022 | 0.006 | 0.007 | 0.004 | 0.005 | 0.006 | 0.005 | 0.008 | 0.008 |
| meta1862 |  | 636.384 | 224.646 | 0.037 | 0.042 | 0.042 | 0.062 | 0.071 | 0.048 | 0.023 | 0.024 | 0.052 | 0.029 | 0.026 | 0.015 | 0.029 | 0.027 | 0.028 |
| meta1863 |  | 637.465 | 47.344 | 0.010 | 0.018 | 0.015 | 0.067 | 0.039 | 0.018 | 0.012 | 0.005 | 0.012 | 0.006 | 0.004 | 0.005 | 0.005 | 0.004 | 0.007 |
| meta1864 |  | 637.556 | 32.443 | 0.056 | 0.071 | 0.037 | 0.008 | 0.005 | 0.001 | 0.002 | 0.003 | 0.002 | 0.012 | 0.054 | 0.070 | 0.002 | 0.001 | 0.002 |
| meta1865 |  | 638.326 | 221.412 | 0.065 | 0.108 | 0.067 | 0.061 | 0.115 | 0.055 | 0.161 | 0.084 | 0.047 | 0.078 | 0.084 | 0.116 | 0.079 | 0.062 | 0.074 |
| meta1866 |  | 638.698 | 288.086 | 0.039 | 0.038 | 0.041 | 0.043 | 0.047 | 0.040 | 0.039 | 0.041 | 0.050 | 0.055 | 0.046 | 0.045 | 0.054 | 0.046 | 0.044 |
| meta1867 |  | 639.494 | 32.651 | 0.013 | 0.014 | 0.009 | 0.004 | 0.009 | 0.006 | 0.009 | 0.008 | 0.002 | 0.007 | 0.011 | 0.014 | 0.009 | 0.009 | 0.001 |
| meta1868 |  | 640.167 | 476.936 | 0.034 | 0.030 | 0.035 | 0.025 | 0.034 | 0.028 | 0.041 | 0.037 | 0.040 | 0.034 | 0.032 | 0.038 | 0.040 | 0.025 | 0.037 |
| meta1869 |  | 640.342 | 213.181 | 0.044 | 0.041 | 0.041 | 0.017 | 0.017 | 0.019 | 0.022 | 0.033 | 0.013 | 0.020 | 0.035 | 0.063 | 0.056 | 0.036 | 0.040 |
| meta1870 |  | 640.390 | 46.145 | 0.015 | 0.013 | 0.016 | 0.051 | 0.030 | 0.017 | 0.011 | 0.005 | 0.010 | 0.004 | 0.004 | 0.005 | 0.005 | 0.006 | 0.004 |
| meta1871 |  | 640.400 | 239.581 | 0.021 | 0.018 | 0.015 | 0.016 | 0.025 | 0.013 | 0.016 | 0.010 | 0.015 | 0.024 | 0.025 | 0.015 | 0.021 | 0.013 | 0.020 |
| meta1872 |  | 640.439 | 144.025 | 0.030 | 0.031 | 0.027 | 0.055 | 0.029 | 0.055 | 0.038 | 0.040 | 0.029 | 0.026 | 0.028 | 0.033 | 0.016 | 0.018 | 0.027 |
| meta1873 |  | 641.085 | 335.402 | 0.057 | 0.067 | 0.057 | 0.060 | 0.073 | 0.046 | 0.052 | 0.066 | 0.049 | 0.060 | 0.067 | 0.033 | 0.060 | 0.059 | 0.070 |
| meta1874 |  | 641.139 | 202.337 | 0.009 | 0.010 | 0.012 | 0.010 | 0.014 | 0.009 | 0.011 | 0.009 | 0.007 | 0.008 | 0.008 | 0.009 | 0.008 | 0.011 | 0.009 |
| meta1875 |  | 641.152 | 473.461 | 0.026 | 0.029 | 0.019 | 0.027 | 0.024 | 0.021 | 0.025 | 0.047 | 0.030 | 0.031 | 0.035 | 0.028 | 0.028 | 0.024 | 0.032 |
| meta1876 |  | 641.166 | 362.086 | 0.076 | 0.101 | 0.075 | 0.098 | 0.091 | 0.061 | 0.091 | 0.092 | 0.064 | 0.092 | 0.082 | 0.088 | 0.072 | 0.050 | 0.104 |
| meta1877 |  | 642.358 | 212.437 | 0.036 | 0.034 | 0.032 | 0.027 | 0.027 | 0.028 | 0.035 | 0.051 | 0.016 | 0.027 | 0.041 | 0.057 | 0.028 | 0.035 | 0.033 |
| meta1878 |  | 642.367 | 287.270 | 0.034 | 0.038 | 0.038 | 0.039 | 0.037 | 0.034 | 0.040 | 0.038 | 0.039 | 0.041 | 0.039 | 0.036 | 0.040 | 0.035 | 0.041 |
| meta1879 |  | 643.103 | 275.419 | 0.024 | 0.023 | 0.016 | 0.017 | 0.026 | 0.017 | 0.014 | 0.026 | 0.020 | 0.027 | 0.021 | 0.008 | 0.022 | 0.019 | 0.019 |
| meta1880 |  | 643.282 | 222.322 | 0.027 | 0.049 | 0.026 | 0.024 | 0.046 | 0.021 | 0.068 | 0.031 | 0.021 | 0.022 | 0.023 | 0.028 | 0.032 | 0.019 | 0.044 |
| meta1881 |  | 643.364 | 309.247 | 0.004 | 0.004 | 0.003 | 0.005 | 0.005 | 0.007 | 0.002 | 0.003 | 0.002 | 0.005 | 0.006 | 0.003 | 0.003 | 0.004 | 0.001 |
| meta1882 |  | 643.526 | 32.682 | 0.013 | 0.009 | 0.011 | 0.005 | 0.004 | 0.003 | 0.008 | 0.013 | 0.004 | 0.005 | 0.010 | 0.008 | 0.009 | 0.004 | 0.003 |
| meta1883 |  | 644.492 | 40.646 | 0.015 | 0.015 | 0.015 | 0.017 | 0.021 | 0.017 | 0.036 | 0.017 | 0.012 | 0.019 | 0.018 | 0.011 | 0.010 | 0.012 | 0.012 |
| meta1884 |  | 645.295 | 227.669 | 0.012 | 0.011 | 0.008 | 0.007 | 0.011 | 0.008 | 0.011 | 0.019 | 0.012 | 0.012 | 0.006 | 0.009 | 0.007 | 0.011 | 0.012 |
| meta1885 |  | 645.329 | 239.540 | 0.003 | 0.002 | 0.002 | 0.005 | 0.007 | 0.010 | 0.003 | 0.002 | 0.003 | 0.003 | 0.003 | 0.003 | 0.001 | 0.003 | 0.003 |
| meta1886 |  | 646.172 | 372.125 | 0.008 | 0.006 | 0.005 | 0.006 | 0.007 | 0.003 | 0.011 | 0.007 | 0.003 | 0.007 | 0.010 | 0.005 | 0.003 | 0.003 | 0.006 |
| meta1887 |  | 646.314 | 168.933 | 0.008 | 0.010 | 0.009 | 0.006 | 0.009 | 0.008 | 0.010 | 0.009 | 0.011 | 0.007 | 0.009 | 0.007 | 0.007 | 0.011 | 0.009 |
| meta1888 |  | 646.403 | 145.286 | 0.015 | 0.026 | 0.025 | 0.038 | 0.030 | 0.033 | 0.026 | 0.022 | 0.024 | 0.021 | 0.023 | 0.021 | 0.018 | 0.026 | 0.026 |
| meta1889 |  | 646.610 | 33.352 | 0.061 | 0.072 | 0.061 | 0.008 | 0.004 | 0.001 | 0.003 | 0.043 | 0.006 | 0.011 | 0.050 | 0.070 | 0.015 | 0.003 | 0.006 |
| meta1890 |  | 647.200 | 406.808 | 0.016 | 0.016 | 0.015 | 0.013 | 0.014 | 0.024 | 0.015 | 0.017 | 0.013 | 0.015 | 0.019 | 0.021 | 0.014 | 0.010 | 0.009 |
| meta1891 |  | 647.343 | 46.356 | 0.007 | 0.008 | 0.004 | 0.009 | 0.011 | 0.007 | 0.006 | 0.009 | 0.007 | 0.004 | 0.004 | 0.007 | 0.004 | 0.008 | 0.009 |
| meta1892 |  | 648.059 | 275.376 | 0.047 | 0.048 | 0.040 | 0.038 | 0.047 | 0.042 | 0.044 | 0.059 | 0.044 | 0.053 | 0.040 | 0.030 | 0.047 | 0.046 | 0.040 |
| meta1893 |  | 648.101 | 423.391 | 0.009 | 0.011 | 0.010 | 0.011 | 0.012 | 0.009 | 0.009 | 0.010 | 0.011 | 0.011 | 0.009 | 0.011 | 0.009 | 0.009 | 0.012 |
| meta1894 |  | 648.312 | 164.408 | 0.004 | 0.006 | 0.004 | 0.009 | 0.006 | 0.007 | 0.004 | 0.006 | 0.005 | 0.005 | 0.005 | 0.007 | 0.005 | 0.004 | 0.006 |
| meta1895 |  | 648.372 | 159.074 | 0.013 | 0.010 | 0.010 | 0.012 | 0.007 | 0.017 | 0.033 | 0.018 | 0.005 | 0.004 | 0.012 | 0.020 | 0.008 | 0.011 | 0.017 |
| meta1896 |  | 649.227 | 351.718 | 0.015 | 0.015 | 0.013 | 0.013 | 0.016 | 0.022 | 0.014 | 0.017 | 0.012 | 0.014 | 0.017 | 0.019 | 0.014 | 0.009 | 0.008 |
| meta1897 |  | 650.387 | 172.082 | 0.024 | 0.022 | 0.018 | 0.016 | 0.017 | 0.012 | 0.061 | 0.061 | 0.007 | 0.004 | 0.022 | 0.065 | 0.027 | 0.027 | 0.022 |
| meta1898 |  | 650.400 | 219.815 | 0.032 | 0.030 | 0.027 | 0.051 | 0.044 | 0.029 | 0.015 | 0.019 | 0.032 | 0.023 | 0.020 | 0.013 | 0.018 | 0.017 | 0.018 |
| meta1899 |  | 651.351 | 184.562 | 0.028 | 0.029 | 0.031 | 0.006 | 0.013 | 0.026 | 0.031 | 0.022 | 0.009 | 0.035 | 0.034 | 0.015 | 0.033 | 0.038 | 0.024 |
| meta1900 |  | 652.050 | 421.247 | 0.010 | 0.012 | 0.010 | 0.012 | 0.012 | 0.009 | 0.011 | 0.009 | 0.011 | 0.007 | 0.009 | 0.010 | 0.010 | 0.008 | 0.011 |
| meta1901 |  | 652.226 | 369.157 | 0.038 | 0.039 | 0.039 | 0.035 | 0.032 | 0.055 | 0.033 | 0.042 | 0.029 | 0.034 | 0.045 | 0.054 | 0.034 | 0.024 | 0.021 |
| meta1902 |  | 653.128 | 444.061 | 0.008 | 0.007 | 0.008 | 0.006 | 0.008 | 0.006 | 0.008 | 0.009 | 0.007 | 0.009 | 0.009 | 0.008 | 0.009 | 0.005 | 0.008 |
| meta1903 |  | 653.152 | 460.520 | 0.031 | 0.027 | 0.031 | 0.021 | 0.024 | 0.027 | 0.030 | 0.029 | 0.040 | 0.035 | 0.033 | 0.037 | 0.046 | 0.026 | 0.030 |
| meta1904 |  | 653.403 | 48.041 | 0.017 | 0.022 | 0.021 | 0.080 | 0.050 | 0.026 | 0.014 | 0.006 | 0.014 | 0.004 | 0.004 | 0.006 | 0.006 | 0.005 | 0.004 |
| meta1905 |  | 654.058 | 465.970 | 0.020 | 0.017 | 0.019 | 0.015 | 0.023 | 0.015 | 0.017 | 0.018 | 0.019 | 0.026 | 0.020 | 0.021 | 0.025 | 0.016 | 0.025 |
| meta1906 |  | 654.827 | 318.444 | 0.030 | 0.030 | 0.035 | 0.030 | 0.031 | 0.036 | 0.031 | 0.027 | 0.037 | 0.029 | 0.031 | 0.027 | 0.035 | 0.040 | 0.028 |
| meta1907 |  | 655.140 | 304.755 | 0.012 | 0.010 | 0.011 | 0.006 | 0.009 | 0.008 | 0.010 | 0.012 | 0.012 | 0.015 | 0.009 | 0.008 | 0.011 | 0.007 | 0.009 |
| meta1908 |  | 656.356 | 163.123 | 0.015 | 0.017 | 0.020 | 0.002 | 0.005 | 0.019 | 0.023 | 0.029 | 0.009 | 0.033 | 0.018 | 0.011 | 0.028 | 0.027 | 0.014 |
| meta1909 |  | 656.433 | 163.155 | 0.005 | 0.007 | 0.006 | 0.009 | 0.004 | 0.012 | 0.005 | 0.005 | 0.005 | 0.003 | 0.005 | 0.006 | 0.002 | 0.006 | 0.004 |
| meta1910 |  | 656.523 | 32.293 | 0.027 | 0.016 | 0.024 | 0.020 | 0.020 | 0.021 | 0.030 | 0.012 | 0.013 | 0.016 | 0.013 | 0.028 | 0.012 | 0.006 | 0.012 |
| meta1911 |  | 656.803 | 286.775 | 0.007 | 0.006 | 0.006 | 0.006 | 0.009 | 0.006 | 0.008 | 0.007 | 0.007 | 0.007 | 0.008 | 0.009 | 0.008 | 0.006 | 0.008 |
| meta1912 |  | 657.094 | 278.585 | 0.002 | 0.003 | 0.003 | 0.003 | 0.003 | 0.002 | 0.004 | 0.003 | 0.002 | 0.002 | 0.003 | 0.003 | 0.002 | 0.002 | 0.001 |
| meta1913 |  | 657.119 | 478.344 | 0.020 | 0.022 | 0.022 | 0.021 | 0.024 | 0.019 | 0.023 | 0.026 | 0.024 | 0.026 | 0.025 | 0.024 | 0.021 | 0.017 | 0.022 |
| meta1914 |  | 657.182 | 369.430 | 0.035 | 0.039 | 0.040 | 0.036 | 0.031 | 0.048 | 0.035 | 0.040 | 0.033 | 0.034 | 0.041 | 0.049 | 0.032 | 0.028 | 0.023 |
| meta1915 |  | 658.366 | 224.716 | 0.004 | 0.005 | 0.005 | 0.006 | 0.007 | 0.006 | 0.002 | 0.002 | 0.005 | 0.003 | 0.002 | 0.002 | 0.003 | 0.002 | 0.003 |
| meta1916 |  | 658.403 | 144.313 | 0.005 | 0.007 | 0.007 | 0.008 | 0.008 | 0.007 | 0.006 | 0.003 | 0.007 | 0.005 | 0.005 | 0.003 | 0.005 | 0.007 | 0.006 |
| meta1917 |  | 659.296 | 392.303 | 0.007 | 0.008 | 0.008 | 0.008 | 0.009 | 0.009 | 0.006 | 0.006 | 0.006 | 0.007 | 0.008 | 0.009 | 0.007 | 0.007 | 0.007 |
| meta1918 |  | 660.061 | 164.326 | 0.125 | 0.135 | 0.144 | 0.130 | 0.161 | 0.134 | 0.135 | 0.070 | 0.207 | 0.097 | 0.132 | 0.123 | 0.162 | 0.144 | 0.146 |
| meta1919 |  | 660.249 | 162.993 | 0.008 | 0.007 | 0.007 | 0.001 | 0.002 | 0.006 | 0.009 | 0.009 | 0.003 | 0.010 | 0.007 | 0.004 | 0.009 | 0.009 | 0.007 |
| meta1920 |  | 660.421 | 143.119 | 0.024 | 0.023 | 0.014 | 0.040 | 0.039 | 0.027 | 0.027 | 0.020 | 0.025 | 0.024 | 0.020 | 0.018 | 0.018 | 0.029 | 0.024 |
| meta1921 |  | 660.788 | 411.197 | 0.021 | 0.019 | 0.017 | 0.013 | 0.023 | 0.018 | 0.019 | 0.026 | 0.020 | 0.026 | 0.033 | 0.035 | 0.030 | 0.025 | 0.022 |
| meta1922 |  | 661.409 | 46.439 | 0.009 | 0.012 | 0.011 | 0.059 | 0.037 | 0.019 | 0.009 | 0.004 | 0.013 | 0.004 | 0.004 | 0.006 | 0.005 | 0.004 | 0.002 |
| meta1923 |  | 662.211 | 399.368 | 0.021 | 0.020 | 0.021 | 0.016 | 0.017 | 0.029 | 0.023 | 0.023 | 0.018 | 0.018 | 0.022 | 0.033 | 0.019 | 0.014 | 0.012 |
| meta1924 |  | 663.122 | 202.324 | 0.021 | 0.022 | 0.021 | 0.020 | 0.021 | 0.017 | 0.019 | 0.022 | 0.017 | 0.021 | 0.022 | 0.021 | 0.019 | 0.021 | 0.020 |
| meta1925 |  | 664.026 | 275.853 | 0.010 | 0.014 | 0.014 | 0.013 | 0.013 | 0.009 | 0.013 | 0.014 | 0.013 | 0.014 | 0.012 | 0.012 | 0.012 | 0.013 | 0.012 |
| meta1926 | Nicotinamide adenine dinucleotide (NAD) | 664.114 | 421.870 | 0.652 | 0.592 | 0.618 | 0.494 | 0.633 | 0.446 | 0.726 | 0.998 | 0.893 | 0.559 | 0.435 | 0.647 | 1.223 | 0.538 | 0.955 |
| meta1927 |  | 664.169 | 469.834 | 0.088 | 0.078 | 0.077 | 0.078 | 0.091 | 0.122 | 0.076 | 0.097 | 0.085 | 0.088 | 0.096 | 0.095 | 0.067 | 0.095 | 0.064 |
| meta1928 |  | 664.343 | 210.005 | 0.090 | 0.086 | 0.095 | 0.080 | 0.080 | 0.096 | 0.102 | 0.105 | 0.051 | 0.076 | 0.120 | 0.134 | 0.095 | 0.109 | 0.078 |
| meta1929 |  | 664.415 | 220.475 | 0.011 | 0.010 | 0.012 | 0.016 | 0.015 | 0.010 | 0.007 | 0.007 | 0.012 | 0.008 | 0.007 | 0.004 | 0.008 | 0.005 | 0.007 |
| meta1930 |  | 664.459 | 57.604 | 0.090 | 0.110 | 0.070 | 0.094 | 0.099 | 0.072 | 0.104 | 0.111 | 0.100 | 0.073 | 0.100 | 0.088 | 0.128 | 0.096 | 0.129 |
| meta1931 |  | 665.211 | 406.617 | 0.093 | 0.083 | 0.083 | 0.070 | 0.083 | 0.127 | 0.090 | 0.098 | 0.067 | 0.084 | 0.102 | 0.118 | 0.085 | 0.055 | 0.049 |
| meta1932 |  | 666.065 | 413.260 | 0.011 | 0.008 | 0.007 | 0.006 | 0.011 | 0.006 | 0.007 | 0.010 | 0.007 | 0.010 | 0.012 | 0.012 | 0.006 | 0.008 | 0.006 |
| meta1933 |  | 666.381 | 188.889 | 0.011 | 0.017 | 0.014 | 0.007 | 0.016 | 0.006 | 0.048 | 0.018 | 0.003 | 0.002 | 0.039 | 0.072 | 0.014 | 0.011 | 0.017 |
| meta1934 |  | 666.431 | 217.556 | 0.043 | 0.050 | 0.054 | 0.075 | 0.059 | 0.054 | 0.036 | 0.036 | 0.047 | 0.033 | 0.031 | 0.023 | 0.038 | 0.037 | 0.032 |
| meta1935 |  | 667.080 | 433.502 | 0.099 | 0.091 | 0.083 | 0.079 | 0.093 | 0.087 | 0.092 | 0.124 | 0.102 | 0.117 | 0.086 | 0.067 | 0.118 | 0.078 | 0.099 |
| meta1936 | Stachyose | 667.227 | 468.554 | 0.163 | 0.151 | 0.166 | 0.132 | 0.155 | 0.249 | 0.177 | 0.209 | 0.147 | 0.173 | 0.169 | 0.203 | 0.167 | 0.094 | 0.110 |
| meta1937 | Maltotetraose | 667.245 | 478.523 | 0.003 | 0.002 | 0.002 | 0.002 | 0.003 | 0.003 | 0.004 | 0.003 | 0.001 | 0.004 | 0.002 | 0.003 | 0.003 | 0.002 | 0.004 |
| meta1938 |  | 668.070 | 20.950 | 0.006 | 0.008 | 0.008 | 0.013 | 0.008 | 0.006 | 0.011 | 0.044 | 0.010 | 0.020 | 0.013 | 0.008 | 0.052 | 0.017 | 0.021 |
| meta1939 |  | 668.153 | 372.076 | 0.011 | 0.010 | 0.008 | 0.011 | 0.012 | 0.006 | 0.015 | 0.012 | 0.006 | 0.011 | 0.018 | 0.008 | 0.008 | 0.007 | 0.013 |
| meta1940 |  | 669.076 | 477.926 | 0.058 | 0.041 | 0.038 | 0.034 | 0.034 | 0.035 | 0.047 | 0.050 | 0.044 | 0.055 | 0.045 | 0.043 | 0.041 | 0.026 | 0.046 |
| meta1941 |  | 669.182 | 352.552 | 0.017 | 0.014 | 0.017 | 0.016 | 0.015 | 0.012 | 0.017 | 0.018 | 0.020 | 0.018 | 0.018 | 0.015 | 0.014 | 0.013 | 0.016 |
| meta1942 |  | 670.607 | 32.979 | 0.011 | 0.016 | 0.011 | 0.002 | 0.006 | 0.003 | 0.005 | 0.011 | 0.002 | 0.003 | 0.012 | 0.015 | 0.004 | 0.004 | 0.006 |
| meta1943 |  | 671.061 | 428.487 | 0.006 | 0.007 | 0.006 | 0.006 | 0.006 | 0.006 | 0.007 | 0.009 | 0.007 | 0.005 | 0.005 | 0.005 | 0.008 | 0.005 | 0.007 |
| meta1944 |  | 671.108 | 432.715 | 0.006 | 0.006 | 0.008 | 0.006 | 0.005 | 0.007 | 0.010 | 0.010 | 0.009 | 0.006 | 0.007 | 0.008 | 0.009 | 0.005 | 0.008 |
| meta1945 |  | 671.162 | 480.112 | 0.070 | 0.075 | 0.076 | 0.075 | 0.094 | 0.088 | 0.082 | 0.110 | 0.090 | 0.101 | 0.077 | 0.083 | 0.097 | 0.070 | 0.088 |
| meta1946 |  | 671.328 | 168.384 | 0.007 | 0.008 | 0.006 | 0.003 | 0.007 | 0.004 | 0.010 | 0.020 | 0.010 | 0.007 | 0.008 | 0.010 | 0.005 | 0.003 | 0.005 |
| meta1947 |  | 671.380 | 314.725 | 0.005 | 0.005 | 0.005 | 0.005 | 0.003 | 0.006 | 0.003 | 0.004 | 0.003 | 0.004 | 0.006 | 0.006 | 0.004 | 0.004 | 0.004 |
| meta1948 |  | 672.292 | 388.395 | 0.027 | 0.023 | 0.028 | 0.019 | 0.026 | 0.023 | 0.021 | 0.021 | 0.014 | 0.026 | 0.024 | 0.026 | 0.031 | 0.023 | 0.036 |
| meta1949 |  | 672.407 | 37.344 | 0.036 | 0.047 | 0.047 | 0.399 | 0.143 | 0.084 | 0.017 | 0.006 | 0.051 | 0.004 | 0.003 | 0.005 | 0.005 | 0.003 | 0.004 |
| meta1950 |  | 672.696 | 287.595 | 0.006 | 0.007 | 0.006 | 0.008 | 0.008 | 0.007 | 0.009 | 0.007 | 0.009 | 0.009 | 0.008 | 0.008 | 0.012 | 0.008 | 0.007 |
| meta1951 |  | 673.198 | 352.422 | 0.006 | 0.007 | 0.005 | 0.007 | 0.008 | 0.006 | 0.006 | 0.007 | 0.009 | 0.007 | 0.009 | 0.007 | 0.008 | 0.006 | 0.007 |
| meta1952 |  | 673.392 | 149.211 | 0.012 | 0.013 | 0.012 | 0.005 | 0.018 | 0.005 | 0.012 | 0.015 | 0.014 | 0.011 | 0.005 | 0.010 | 0.010 | 0.010 | 0.007 |
| meta1953 |  | 673.513 | 41.039 | 0.008 | 0.008 | 0.008 | 0.013 | 0.012 | 0.017 | 0.018 | 0.012 | 0.015 | 0.016 | 0.020 | 0.019 | 0.019 | 0.020 | 0.019 |
| meta1954 |  | 674.459 | 32.853 | 0.004 | 0.004 | 0.004 | 0.009 | 0.011 | 0.005 | 0.008 | 0.012 | 0.006 | 0.006 | 0.007 | 0.004 | 0.021 | 0.006 | 0.020 |
| meta1955 |  | 674.675 | 287.796 | 0.023 | 0.025 | 0.021 | 0.028 | 0.028 | 0.022 | 0.027 | 0.025 | 0.031 | 0.027 | 0.029 | 0.026 | 0.031 | 0.025 | 0.025 |
| meta1956 |  | 675.416 | 46.913 | 0.011 | 0.014 | 0.015 | 0.060 | 0.032 | 0.014 | 0.006 | 0.004 | 0.009 | 0.003 | 0.003 | 0.004 | 0.003 | 0.004 | 0.003 |
| meta1957 |  | 675.540 | 163.830 | 0.019 | 0.019 | 0.017 | 0.010 | 0.010 | 0.005 | 0.005 | 0.013 | 0.007 | 0.009 | 0.014 | 0.014 | 0.011 | 0.007 | 0.006 |
| meta1958 |  | 676.244 | 33.706 | 0.001 | 0.001 | 0.002 | 0.002 | 0.002 | 0.002 | 0.003 | 0.002 | 0.003 | 0.002 | 0.002 | 0.002 | 0.003 | 0.003 | 0.002 |
| meta1959 |  | 676.416 | 215.082 | 0.017 | 0.016 | 0.017 | 0.029 | 0.029 | 0.019 | 0.011 | 0.009 | 0.014 | 0.011 | 0.010 | 0.006 | 0.011 | 0.008 | 0.012 |
| meta1960 |  | 676.808 | 318.444 | 0.015 | 0.016 | 0.017 | 0.015 | 0.015 | 0.018 | 0.016 | 0.015 | 0.018 | 0.015 | 0.015 | 0.014 | 0.020 | 0.020 | 0.016 |
| meta1961 |  | 677.143 | 152.185 | 0.001 | 0.002 | 0.002 | 0.002 | 0.002 | 0.002 | 0.004 | 0.001 | 0.001 | 0.001 | 0.002 | 0.002 | 0.001 | 0.001 | 0.002 |
| meta1962 |  | 677.149 | 471.067 | 0.022 | 0.015 | 0.015 | 0.015 | 0.017 | 0.015 | 0.016 | 0.011 | 0.011 | 0.022 | 0.013 | 0.016 | 0.026 | 0.011 | 0.018 |
| meta1963 |  | 677.184 | 434.031 | 0.018 | 0.023 | 0.021 | 0.023 | 0.015 | 0.012 | 0.023 | 0.020 | 0.029 | 0.023 | 0.016 | 0.014 | 0.018 | 0.013 | 0.020 |
| meta1964 |  | 677.372 | 206.016 | 0.004 | 0.004 | 0.005 | 0.000 | 0.001 | 0.001 | 0.027 | 0.000 | 0.001 | 0.004 | 0.002 | 0.004 | 0.004 | 0.010 | 0.002 |
| meta1965 |  | 678.066 | 380.950 | 0.010 | 0.011 | 0.010 | 0.007 | 0.013 | 0.007 | 0.014 | 0.013 | 0.009 | 0.007 | 0.006 | 0.005 | 0.008 | 0.006 | 0.010 |
| meta1966 |  | 678.383 | 171.462 | 0.009 | 0.012 | 0.010 | 0.002 | 0.002 | 0.003 | 0.034 | 0.010 | 0.016 | 0.012 | 0.018 | 0.014 | 0.044 | 0.011 | 0.009 |
| meta1967 |  | 678.467 | 139.952 | 0.055 | 0.064 | 0.049 | 0.103 | 0.073 | 0.063 | 0.059 | 0.044 | 0.064 | 0.055 | 0.049 | 0.046 | 0.048 | 0.046 | 0.053 |
| meta1968 |  | 678.476 | 40.796 | 0.033 | 0.042 | 0.041 | 0.234 | 0.113 | 0.091 | 0.041 | 0.016 | 0.036 | 0.025 | 0.020 | 0.016 | 0.017 | 0.017 | 0.017 |
| meta1969 |  | 678.652 | 288.285 | 0.072 | 0.070 | 0.079 | 0.077 | 0.074 | 0.071 | 0.074 | 0.077 | 0.097 | 0.090 | 0.082 | 0.080 | 0.105 | 0.086 | 0.080 |
| meta1970 |  | 679.101 | 478.468 | 0.006 | 0.008 | 0.006 | 0.007 | 0.006 | 0.006 | 0.007 | 0.007 | 0.007 | 0.009 | 0.009 | 0.007 | 0.005 | 0.006 | 0.007 |
| meta1971 |  | 679.124 | 442.420 | 0.047 | 0.048 | 0.045 | 0.042 | 0.050 | 0.034 | 0.053 | 0.068 | 0.062 | 0.040 | 0.034 | 0.051 | 0.082 | 0.044 | 0.068 |
| meta1972 |  | 680.398 | 185.298 | 0.007 | 0.012 | 0.010 | 0.004 | 0.002 | 0.003 | 0.017 | 0.013 | 0.013 | 0.008 | 0.012 | 0.009 | 0.015 | 0.009 | 0.004 |
| meta1973 |  | 680.477 | 572.450 | 0.045 | 0.053 | 0.047 | 0.049 | 0.053 | 0.039 | 0.052 | 0.049 | 0.050 | 0.049 | 0.055 | 0.048 | 0.048 | 0.047 | 0.056 |
| meta1974 |  | 680.477 | 646.641 | 0.018 | 0.023 | 0.014 | 0.015 | 0.038 | 0.016 | 0.064 | 0.019 | 0.047 | 0.020 | 0.004 | 0.026 | 0.019 | 0.015 | 0.017 |
| meta1975 |  | 682.238 | 406.822 | 0.544 | 0.528 | 0.504 | 0.426 | 0.473 | 0.779 | 0.531 | 0.620 | 0.467 | 0.499 | 0.614 | 0.744 | 0.502 | 0.354 | 0.292 |
| meta1976 |  | 682.237 | 433.046 | 0.040 | 0.058 | 0.040 | 0.035 | 0.036 | 0.056 | 0.041 | 0.048 | 0.029 | 0.037 | 0.061 | 0.082 | 0.038 | 0.026 | 0.021 |
| meta1977 |  | 682.557 | 49.081 | 0.005 | 0.010 | 0.008 | 0.003 | 0.001 | 0.001 | 0.001 | 0.004 | 0.001 | 0.002 | 0.007 | 0.006 | 0.003 | 0.001 | 0.002 |
| meta1978 |  | 684.414 | 46.143 | 0.011 | 0.013 | 0.013 | 0.039 | 0.024 | 0.014 | 0.007 | 0.004 | 0.008 | 0.002 | 0.003 | 0.004 | 0.003 | 0.003 | 0.004 |
| meta1979 |  | 684.515 | 40.457 | 0.003 | 0.004 | 0.004 | 0.005 | 0.004 | 0.005 | 0.008 | 0.007 | 0.005 | 0.004 | 0.008 | 0.007 | 0.006 | 0.007 | 0.007 |
| meta1980 |  | 685.389 | 287.075 | 0.035 | 0.040 | 0.039 | 0.038 | 0.042 | 0.031 | 0.041 | 0.043 | 0.039 | 0.040 | 0.039 | 0.038 | 0.039 | 0.039 | 0.040 |
| meta1981 |  | 685.433 | 572.450 | 0.009 | 0.011 | 0.009 | 0.010 | 0.011 | 0.009 | 0.011 | 0.010 | 0.010 | 0.010 | 0.010 | 0.010 | 0.010 | 0.009 | 0.012 |
| meta1982 |  | 685.432 | 278.637 | 0.002 | 0.003 | 0.003 | 0.002 | 0.014 | 0.002 | 0.002 | 0.002 | 0.003 | 0.003 | 0.002 | 0.003 | 0.002 | 0.002 | 0.003 |
| meta1983 |  | 686.152 | 470.489 | 0.011 | 0.008 | 0.008 | 0.008 | 0.006 | 0.009 | 0.010 | 0.004 | 0.011 | 0.011 | 0.012 | 0.009 | 0.014 | 0.011 | 0.017 |
| meta1984 |  | 686.260 | 27.382 | 0.030 | 0.038 | 0.037 | 0.039 | 0.037 | 0.025 | 0.035 | 0.042 | 0.038 | 0.032 | 0.039 | 0.038 | 0.038 | 0.030 | 0.036 |
| meta1985 |  | 686.436 | 139.886 | 0.014 | 0.016 | 0.022 | 0.024 | 0.022 | 0.021 | 0.023 | 0.019 | 0.018 | 0.023 | 0.020 | 0.015 | 0.019 | 0.031 | 0.028 |
| meta1986 |  | 687.194 | 406.174 | 0.555 | 0.648 | 0.593 | 0.537 | 0.525 | 0.829 | 0.561 | 0.625 | 0.439 | 0.517 | 0.655 | 0.774 | 0.523 | 0.365 | 0.345 |
| meta1987 |  | 687.299 | 168.314 | 0.001 | 0.002 | 0.001 | 0.000 | 0.002 | 0.001 | 0.002 | 0.003 | 0.002 | 0.002 | 0.002 | 0.002 | 0.002 | 0.002 | 0.001 |
| meta1988 | Dephosphocoenzyme A (Dephospho-CoA) | 688.153 | 339.225 | 0.042 | 0.029 | 0.032 | 0.021 | 0.029 | 0.030 | 0.044 | 0.025 | 0.023 | 0.031 | 0.025 | 0.028 | 0.030 | 0.024 | 0.029 |
| meta1989 |  | 688.343 | 208.681 | 0.073 | 0.081 | 0.085 | 0.072 | 0.082 | 0.073 | 0.104 | 0.091 | 0.051 | 0.072 | 0.104 | 0.113 | 0.125 | 0.111 | 0.075 |
| meta1990 |  | 689.062 | 434.189 | 0.010 | 0.010 | 0.009 | 0.009 | 0.009 | 0.007 | 0.010 | 0.013 | 0.010 | 0.011 | 0.007 | 0.008 | 0.010 | 0.008 | 0.009 |
| meta1991 |  | 689.208 | 469.159 | 0.159 | 0.174 | 0.168 | 0.147 | 0.157 | 0.198 | 0.179 | 0.172 | 0.149 | 0.191 | 0.192 | 0.225 | 0.156 | 0.106 | 0.100 |
| meta1992 |  | 689.556 | 163.170 | 0.061 | 0.072 | 0.061 | 0.023 | 0.015 | 0.010 | 0.009 | 0.038 | 0.021 | 0.030 | 0.051 | 0.053 | 0.028 | 0.014 | 0.018 |
| meta1993 |  | 690.135 | 372.229 | 0.012 | 0.012 | 0.011 | 0.011 | 0.012 | 0.007 | 0.017 | 0.013 | 0.006 | 0.013 | 0.019 | 0.007 | 0.006 | 0.005 | 0.014 |
| meta1994 |  | 690.366 | 277.437 | 0.002 | 0.002 | 0.002 | 0.001 | 0.002 | 0.001 | 0.002 | 0.002 | 0.002 | 0.003 | 0.002 | 0.003 | 0.003 | 0.003 | 0.002 |
| meta1995 |  | 690.431 | 211.230 | 0.015 | 0.018 | 0.018 | 0.026 | 0.025 | 0.015 | 0.011 | 0.011 | 0.018 | 0.015 | 0.011 | 0.009 | 0.014 | 0.010 | 0.011 |
| meta1996 |  | 690.467 | 138.439 | 0.016 | 0.023 | 0.023 | 0.053 | 0.041 | 0.033 | 0.027 | 0.012 | 0.029 | 0.022 | 0.022 | 0.023 | 0.012 | 0.024 | 0.026 |
| meta1997 |  | 692.140 | 24.675 | 0.001 | 0.001 | 0.001 | 0.002 | 0.001 | 0.001 | 0.001 | 0.005 | 0.001 | 0.002 | 0.002 | 0.001 | 0.007 | 0.003 | 0.003 |
| meta1998 |  | 692.457 | 189.743 | 0.004 | 0.004 | 0.003 | 0.007 | 0.006 | 0.004 | 0.004 | 0.004 | 0.004 | 0.003 | 0.003 | 0.002 | 0.002 | 0.002 | 0.002 |
| meta1999 |  | 693.190 | 351.655 | 0.019 | 0.023 | 0.021 | 0.024 | 0.025 | 0.028 | 0.022 | 0.022 | 0.015 | 0.019 | 0.021 | 0.031 | 0.016 | 0.014 | 0.014 |
| meta2000 |  | 694.135 | 23.092 | 0.018 | 0.020 | 0.018 | 0.020 | 0.016 | 0.013 | 0.018 | 0.074 | 0.020 | 0.042 | 0.029 | 0.020 | 0.088 | 0.030 | 0.034 |
| meta2001 |  | 694.196 | 26.326 | 0.003 | 0.002 | 0.003 | 0.004 | 0.002 | 0.002 | 0.003 | 0.007 | 0.004 | 0.003 | 0.003 | 0.005 | 0.006 | 0.004 | 0.005 |
| meta2002 |  | 694.274 | 388.429 | 0.026 | 0.021 | 0.024 | 0.017 | 0.025 | 0.022 | 0.020 | 0.020 | 0.014 | 0.024 | 0.024 | 0.024 | 0.033 | 0.024 | 0.033 |
| meta2003 |  | 694.462 | 213.935 | 0.010 | 0.009 | 0.010 | 0.015 | 0.008 | 0.007 | 0.006 | 0.008 | 0.009 | 0.008 | 0.008 | 0.006 | 0.007 | 0.005 | 0.009 |
| meta2004 |  | 694.558 | 31.117 | 0.004 | 0.004 | 0.004 | 0.002 | 0.003 | 0.002 | 0.003 | 0.007 | 0.003 | 0.003 | 0.004 | 0.006 | 0.003 | 0.002 | 0.003 |
| meta2005 |  | 695.162 | 462.141 | 0.156 | 0.158 | 0.145 | 0.158 | 0.170 | 0.129 | 0.166 | 0.232 | 0.159 | 0.166 | 0.167 | 0.155 | 0.167 | 0.127 | 0.182 |
| meta2006 |  | 695.327 | 167.706 | 0.008 | 0.012 | 0.011 | 0.010 | 0.010 | 0.006 | 0.022 | 0.034 | 0.012 | 0.016 | 0.015 | 0.013 | 0.012 | 0.004 | 0.010 |
| meta2007 |  | 695.375 | 149.595 | 0.005 | 0.004 | 0.005 | 0.002 | 0.007 | 0.002 | 0.006 | 0.004 | 0.006 | 0.004 | 0.002 | 0.003 | 0.004 | 0.003 | 0.002 |
| meta2008 |  | 696.106 | 437.057 | 0.007 | 0.007 | 0.007 | 0.006 | 0.005 | 0.005 | 0.007 | 0.009 | 0.008 | 0.007 | 0.006 | 0.008 | 0.014 | 0.007 | 0.010 |
| meta2009 |  | 696.193 | 466.359 | 0.052 | 0.044 | 0.049 | 0.030 | 0.025 | 0.034 | 0.030 | 0.033 | 0.046 | 0.072 | 0.055 | 0.051 | 0.080 | 0.032 | 0.052 |
| meta2010 |  | 697.512 | 40.408 | 0.003 | 0.007 | 0.006 | 0.009 | 0.009 | 0.009 | 0.009 | 0.011 | 0.012 | 0.008 | 0.011 | 0.012 | 0.008 | 0.010 | 0.009 |
| meta2011 |  | 698.790 | 318.447 | 0.007 | 0.007 | 0.008 | 0.006 | 0.007 | 0.008 | 0.007 | 0.006 | 0.008 | 0.007 | 0.007 | 0.007 | 0.009 | 0.009 | 0.007 |
| meta2012 |  | 699.167 | 434.007 | 0.011 | 0.016 | 0.015 | 0.013 | 0.013 | 0.008 | 0.015 | 0.012 | 0.019 | 0.019 | 0.009 | 0.008 | 0.011 | 0.009 | 0.013 |
| meta2013 |  | 699.357 | 206.167 | 0.009 | 0.011 | 0.010 | 0.004 | 0.003 | 0.004 | 0.058 | 0.002 | 0.005 | 0.010 | 0.005 | 0.009 | 0.011 | 0.020 | 0.005 |
| meta2014 |  | 700.569 | 49.086 | 0.022 | 0.034 | 0.030 | 0.013 | 0.005 | 0.005 | 0.004 | 0.017 | 0.006 | 0.009 | 0.025 | 0.025 | 0.013 | 0.003 | 0.010 |
| meta2015 |  | 701.427 | 43.923 | 0.027 | 0.035 | 0.031 | 0.167 | 0.076 | 0.048 | 0.018 | 0.004 | 0.025 | 0.003 | 0.003 | 0.007 | 0.003 | 0.003 | 0.003 |
| meta2016 |  | 701.557 | 162.504 | 0.097 | 0.115 | 0.108 | 0.059 | 0.050 | 0.032 | 0.047 | 0.080 | 0.044 | 0.045 | 0.082 | 0.108 | 0.049 | 0.044 | 0.040 |
| meta2017 |  | 702.129 | 432.741 | 0.026 | 0.024 | 0.024 | 0.026 | 0.034 | 0.025 | 0.036 | 0.024 | 0.022 | 0.015 | 0.021 | 0.025 | 0.043 | 0.026 | 0.043 |
| meta2018 |  | 702.133 | 392.315 | 0.010 | 0.013 | 0.012 | 0.009 | 0.012 | 0.010 | 0.018 | 0.011 | 0.007 | 0.010 | 0.010 | 0.013 | 0.009 | 0.008 | 0.011 |
| meta2019 |  | 702.376 | 357.162 | 0.007 | 0.008 | 0.008 | 0.007 | 0.006 | 0.009 | 0.005 | 0.006 | 0.003 | 0.006 | 0.010 | 0.007 | 0.006 | 0.005 | 0.005 |
| meta2020 |  | 703.166 | 406.265 | 0.073 | 0.087 | 0.074 | 0.073 | 0.074 | 0.108 | 0.076 | 0.081 | 0.063 | 0.070 | 0.087 | 0.104 | 0.074 | 0.052 | 0.046 |
| meta2021 |  | 703.572 | 162.493 | 1.084 | 1.215 | 1.106 | 0.306 | 0.222 | 0.157 | 0.183 | 0.662 | 0.219 | 0.302 | 0.836 | 0.826 | 0.309 | 0.206 | 0.296 |
| meta2022 |  | 703.572 | 184.572 | 0.190 | 0.193 | 0.195 | 0.045 | 0.040 | 0.042 | 0.064 | 0.154 | 0.043 | 0.074 | 0.157 | 0.178 | 0.092 | 0.036 | 0.069 |
| meta2023 |  | 704.393 | 171.609 | 0.014 | 0.015 | 0.020 | 0.022 | 0.026 | 0.015 | 0.020 | 0.019 | 0.014 | 0.020 | 0.020 | 0.026 | 0.018 | 0.011 | 0.030 |
| meta2024 |  | 704.518 | 42.485 | 0.026 | 0.034 | 0.027 | 0.023 | 0.016 | 0.012 | 0.012 | 0.013 | 0.006 | 0.007 | 0.061 | 0.074 | 0.008 | 0.005 | 0.009 |
| meta2025 |  | 705.130 | 287.221 | 0.005 | 0.004 | 0.004 | 0.006 | 0.006 | 0.005 | 0.005 | 0.005 | 0.005 | 0.005 | 0.006 | 0.004 | 0.006 | 0.004 | 0.005 |
| meta2026 |  | 705.185 | 467.262 | 0.043 | 0.031 | 0.039 | 0.024 | 0.032 | 0.044 | 0.032 | 0.034 | 0.037 | 0.040 | 0.046 | 0.066 | 0.037 | 0.042 | 0.026 |
| meta2027 | Triflupromazine | 705.241 | 361.594 | 0.033 | 0.022 | 0.025 | 0.024 | 0.023 | 0.021 | 0.021 | 0.026 | 0.026 | 0.020 | 0.022 | 0.025 | 0.017 | 0.032 | 0.022 |
| meta2028 |  | 706.334 | 426.040 | 0.005 | 0.005 | 0.005 | 0.005 | 0.005 | 0.006 | 0.008 | 0.008 | 0.003 | 0.005 | 0.007 | 0.007 | 0.004 | 0.005 | 0.005 |
| meta2029 |  | 706.463 | 206.681 | 0.011 | 0.014 | 0.011 | 0.020 | 0.014 | 0.010 | 0.008 | 0.009 | 0.012 | 0.010 | 0.008 | 0.008 | 0.009 | 0.007 | 0.007 |
| meta2030 |  | 706.535 | 134.059 | 0.071 | 0.121 | 0.106 | 0.038 | 0.030 | 0.023 | 0.024 | 0.074 | 0.014 | 0.042 | 0.075 | 0.096 | 0.021 | 0.010 | 0.025 |
| meta2031 |  | 707.073 | 164.326 | 0.019 | 0.021 | 0.021 | 0.018 | 0.022 | 0.019 | 0.020 | 0.011 | 0.032 | 0.012 | 0.019 | 0.018 | 0.023 | 0.021 | 0.020 |
| meta2032 |  | 707.219 | 370.573 | 0.199 | 0.180 | 0.177 | 0.154 | 0.237 | 0.310 | 0.169 | 0.202 | 0.107 | 0.186 | 0.191 | 0.274 | 0.128 | 0.161 | 0.069 |
| meta2033 |  | 707.561 | 275.376 | 0.012 | 0.018 | 0.011 | 0.012 | 0.014 | 0.011 | 0.013 | 0.015 | 0.012 | 0.017 | 0.011 | 0.007 | 0.013 | 0.013 | 0.012 |
| meta2034 |  | 708.062 | 275.376 | 0.004 | 0.005 | 0.004 | 0.004 | 0.006 | 0.005 | 0.004 | 0.004 | 0.004 | 0.004 | 0.003 | 0.003 | 0.004 | 0.004 | 0.003 |
| meta2035 |  | 708.486 | 58.305 | 0.026 | 0.029 | 0.031 | 0.007 | 0.024 | 0.014 | 0.021 | 0.028 | 0.026 | 0.015 | 0.020 | 0.020 | 0.017 | 0.020 | 0.017 |
| meta2036 |  | 708.508 | 572.755 | 0.030 | 0.037 | 0.032 | 0.029 | 0.036 | 0.025 | 0.036 | 0.034 | 0.037 | 0.036 | 0.034 | 0.030 | 0.033 | 0.033 | 0.036 |
| meta2037 |  | 708.572 | 29.427 | 0.030 | 0.043 | 0.023 | 0.059 | 0.044 | 0.014 | 0.060 | 0.044 | 0.041 | 0.021 | 0.032 | 0.035 | 0.021 | 0.044 | 0.058 |
| meta2038 |  | 708.655 | 288.784 | 0.006 | 0.006 | 0.006 | 0.006 | 0.008 | 0.007 | 0.008 | 0.006 | 0.009 | 0.007 | 0.006 | 0.007 | 0.009 | 0.009 | 0.008 |
| meta2039 |  | 710.269 | 406.239 | 0.011 | 0.012 | 0.009 | 0.009 | 0.011 | 0.013 | 0.011 | 0.011 | 0.008 | 0.010 | 0.013 | 0.013 | 0.009 | 0.006 | 0.006 |
| meta2040 |  | 711.067 | 164.326 | 0.152 | 0.179 | 0.190 | 0.153 | 0.195 | 0.168 | 0.161 | 0.099 | 0.247 | 0.122 | 0.151 | 0.144 | 0.198 | 0.169 | 0.163 |
| meta2041 |  | 711.420 | 287.076 | 0.002 | 0.002 | 0.002 | 0.000 | 0.000 | 0.001 | 0.001 | 0.001 | 0.002 | 0.000 | 0.001 | 0.010 | 0.010 | 0.006 | 0.008 |
| meta2042 |  | 711.494 | 41.702 | 0.009 | 0.010 | 0.006 | 0.015 | 0.015 | 0.005 | 0.005 | 0.007 | 0.004 | 0.004 | 0.013 | 0.013 | 0.007 | 0.009 | 0.009 |
| meta2043 |  | 712.007 | 49.731 | 0.003 | 0.003 | 0.003 | 0.010 | 0.004 | 0.002 | 0.001 | 0.000 | 0.001 | 0.000 | 0.000 | 0.000 | 0.000 | 0.000 | 0.001 |
| meta2044 |  | 712.059 | 421.238 | 0.006 | 0.005 | 0.007 | 0.006 | 0.007 | 0.005 | 0.009 | 0.005 | 0.006 | 0.005 | 0.006 | 0.006 | 0.006 | 0.004 | 0.008 |
| meta2045 |  | 712.117 | 372.319 | 0.009 | 0.009 | 0.008 | 0.010 | 0.008 | 0.005 | 0.013 | 0.010 | 0.006 | 0.010 | 0.013 | 0.006 | 0.005 | 0.005 | 0.013 |
| meta2046 |  | 712.146 | 279.456 | 0.003 | 0.004 | 0.004 | 0.002 | 0.007 | 0.001 | 0.007 | 0.001 | 0.004 | 0.003 | 0.001 | 0.001 | 0.005 | 0.002 | 0.001 |
| meta2047 |  | 712.189 | 478.370 | 0.029 | 0.025 | 0.036 | 0.025 | 0.029 | 0.034 | 0.026 | 0.035 | 0.034 | 0.029 | 0.030 | 0.043 | 0.038 | 0.027 | 0.040 |
| meta2048 |  | 712.631 | 287.595 | 0.006 | 0.006 | 0.008 | 0.008 | 0.009 | 0.007 | 0.009 | 0.008 | 0.009 | 0.009 | 0.008 | 0.008 | 0.010 | 0.008 | 0.008 |
| meta2049 |  | 714.364 | 393.992 | 0.005 | 0.005 | 0.006 | 0.005 | 0.005 | 0.004 | 0.004 | 0.005 | 0.004 | 0.005 | 0.004 | 0.004 | 0.004 | 0.004 | 0.007 |
| meta2050 |  | 714.504 | 132.299 | 0.049 | 0.070 | 0.064 | 0.047 | 0.045 | 0.021 | 0.060 | 0.065 | 0.032 | 0.064 | 0.053 | 0.070 | 0.040 | 0.032 | 0.046 |
| meta2051 |  | 714.526 | 32.319 | 0.007 | 0.006 | 0.006 | 0.005 | 0.009 | 0.006 | 0.010 | 0.008 | 0.011 | 0.004 | 0.009 | 0.013 | 0.006 | 0.004 | 0.006 |
| meta2052 |  | 714.610 | 288.221 | 0.021 | 0.020 | 0.023 | 0.024 | 0.025 | 0.023 | 0.025 | 0.024 | 0.032 | 0.026 | 0.024 | 0.025 | 0.032 | 0.028 | 0.026 |
| meta2053 |  | 715.205 | 371.021 | 0.007 | 0.005 | 0.005 | 0.005 | 0.007 | 0.008 | 0.005 | 0.005 | 0.003 | 0.006 | 0.007 | 0.008 | 0.003 | 0.004 | 0.002 |
| meta2054 |  | 716.159 | 406.201 | 0.005 | 0.007 | 0.006 | 0.006 | 0.008 | 0.006 | 0.008 | 0.005 | 0.004 | 0.006 | 0.006 | 0.006 | 0.006 | 0.006 | 0.006 |
| meta2055 |  | 716.261 | 480.138 | 0.008 | 0.007 | 0.006 | 0.007 | 0.009 | 0.006 | 0.009 | 0.005 | 0.005 | 0.005 | 0.005 | 0.011 | 0.008 | 0.007 | 0.007 |
| meta2056 |  | 716.433 | 38.807 | 0.042 | 0.053 | 0.058 | 0.488 | 0.177 | 0.100 | 0.022 | 0.003 | 0.064 | 0.002 | 0.004 | 0.006 | 0.004 | 0.004 | 0.004 |
| meta2057 |  | 716.447 | 205.426 | 0.009 | 0.011 | 0.010 | 0.019 | 0.018 | 0.009 | 0.006 | 0.008 | 0.011 | 0.009 | 0.009 | 0.006 | 0.008 | 0.007 | 0.007 |
| meta2058 |  | 716.520 | 132.926 | 0.882 | 0.704 | 0.604 | 0.213 | 0.180 | 0.080 | 0.140 | 0.997 | 0.184 | 0.249 | 0.696 | 0.734 | 0.338 | 0.062 | 0.280 |
| meta2059 |  | 716.826 | 318.444 | 0.031 | 0.031 | 0.037 | 0.028 | 0.033 | 0.037 | 0.031 | 0.027 | 0.040 | 0.029 | 0.032 | 0.028 | 0.035 | 0.041 | 0.031 |
| meta2060 |  | 717.112 | 416.758 | 0.025 | 0.021 | 0.021 | 0.019 | 0.021 | 0.017 | 0.020 | 0.030 | 0.029 | 0.027 | 0.027 | 0.025 | 0.026 | 0.017 | 0.030 |
| meta2061 |  | 717.144 | 462.005 | 0.016 | 0.015 | 0.017 | 0.015 | 0.019 | 0.013 | 0.020 | 0.020 | 0.015 | 0.016 | 0.016 | 0.016 | 0.020 | 0.013 | 0.019 |
| meta2062 |  | 718.462 | 207.101 | 0.007 | 0.007 | 0.007 | 0.010 | 0.009 | 0.004 | 0.004 | 0.006 | 0.007 | 0.005 | 0.006 | 0.003 | 0.004 | 0.004 | 0.007 |
| meta2063 | 1-Palmitoyl-2-oleoyl-sn-glycero-3-phosphoethanolamine | 718.531 | 134.177 | 0.095 | 0.116 | 0.108 | 0.057 | 0.039 | 0.017 | 0.037 | 0.191 | 0.053 | 0.063 | 0.100 | 0.109 | 0.094 | 0.025 | 0.028 |
| meta2064 |  | 718.805 | 286.775 | 0.009 | 0.008 | 0.006 | 0.006 | 0.010 | 0.007 | 0.007 | 0.008 | 0.007 | 0.011 | 0.010 | 0.011 | 0.007 | 0.007 | 0.009 |
| meta2065 |  | 719.159 | 392.448 | 0.026 | 0.028 | 0.028 | 0.021 | 0.027 | 0.025 | 0.039 | 0.023 | 0.018 | 0.024 | 0.027 | 0.024 | 0.020 | 0.019 | 0.025 |
| meta2066 |  | 720.113 | 380.824 | 0.003 | 0.003 | 0.003 | 0.003 | 0.004 | 0.003 | 0.005 | 0.005 | 0.003 | 0.003 | 0.003 | 0.001 | 0.002 | 0.002 | 0.005 |
| meta2067 |  | 722.503 | 42.055 | 0.032 | 0.037 | 0.038 | 0.200 | 0.108 | 0.073 | 0.039 | 0.020 | 0.037 | 0.023 | 0.020 | 0.020 | 0.019 | 0.010 | 0.016 |
| meta2068 |  | 722.550 | 49.549 | 0.008 | 0.012 | 0.011 | 0.004 | 0.004 | 0.002 | 0.003 | 0.008 | 0.003 | 0.007 | 0.009 | 0.008 | 0.003 | 0.002 | 0.001 |
| meta2069 |  | 723.192 | 370.788 | 0.006 | 0.005 | 0.004 | 0.005 | 0.007 | 0.008 | 0.005 | 0.005 | 0.003 | 0.005 | 0.006 | 0.008 | 0.004 | 0.004 | 0.002 |
| meta2070 |  | 724.391 | 175.844 | 0.008 | 0.012 | 0.013 | 0.015 | 0.015 | 0.021 | 0.013 | 0.009 | 0.030 | 0.017 | 0.011 | 0.007 | 0.012 | 0.018 | 0.017 |
| meta2071 |  | 725.173 | 476.337 | 0.012 | 0.012 | 0.011 | 0.013 | 0.014 | 0.008 | 0.014 | 0.015 | 0.011 | 0.013 | 0.013 | 0.023 | 0.014 | 0.019 | 0.012 |
| meta2072 |  | 727.082 | 474.824 | 0.024 | 0.023 | 0.018 | 0.016 | 0.017 | 0.015 | 0.019 | 0.021 | 0.017 | 0.021 | 0.022 | 0.021 | 0.019 | 0.014 | 0.020 |
| meta2073 |  | 729.078 | 476.107 | 0.011 | 0.009 | 0.008 | 0.008 | 0.010 | 0.006 | 0.010 | 0.011 | 0.009 | 0.007 | 0.010 | 0.012 | 0.010 | 0.006 | 0.012 |
| meta2074 |  | 729.416 | 287.941 | 0.012 | 0.013 | 0.010 | 0.013 | 0.011 | 0.012 | 0.011 | 0.011 | 0.013 | 0.013 | 0.011 | 0.012 | 0.011 | 0.013 | 0.011 |
| meta2075 |  | 729.724 | 288.579 | 0.003 | 0.002 | 0.003 | 0.003 | 0.003 | 0.004 | 0.003 | 0.003 | 0.003 | 0.003 | 0.003 | 0.003 | 0.003 | 0.003 | 0.003 |
| meta2076 |  | 730.188 | 459.817 | 0.026 | 0.029 | 0.038 | 0.023 | 0.033 | 0.030 | 0.066 | 0.037 | 0.033 | 0.033 | 0.037 | 0.032 | 0.048 | 0.048 | 0.036 |
| meta2077 |  | 730.462 | 202.147 | 0.013 | 0.013 | 0.013 | 0.022 | 0.021 | 0.008 | 0.009 | 0.008 | 0.016 | 0.011 | 0.008 | 0.007 | 0.011 | 0.009 | 0.009 |
| meta2078 |  | 732.444 | 35.420 | 0.010 | 0.012 | 0.013 | 0.139 | 0.046 | 0.027 | 0.006 | 0.017 | 0.021 | 0.015 | 0.023 | 0.025 | 0.018 | 0.015 | 0.024 |
| meta2079 |  | 732.551 | 131.631 | 1.559 | 1.987 | 1.799 | 1.683 | 0.945 | 0.945 | 0.970 | 3.182 | 0.483 | 1.006 | 2.150 | 2.302 | 0.649 | 0.893 | 1.047 |
| meta2080 |  | 732.545 | 42.485 | 0.026 | 0.017 | 0.022 | 0.015 | 0.013 | 0.015 | 0.023 | 0.010 | 0.013 | 0.014 | 0.027 | 0.024 | 0.017 | 0.007 | 0.012 |
| meta2081 |  | 734.099 | 372.221 | 0.007 | 0.009 | 0.007 | 0.008 | 0.007 | 0.005 | 0.009 | 0.008 | 0.007 | 0.009 | 0.010 | 0.005 | 0.005 | 0.005 | 0.011 |
| meta2082 |  | 736.189 | 454.055 | 0.012 | 0.011 | 0.011 | 0.010 | 0.010 | 0.010 | 0.010 | 0.016 | 0.012 | 0.013 | 0.016 | 0.017 | 0.015 | 0.010 | 0.012 |
| meta2083 |  | 736.455 | 221.841 | 0.002 | 0.003 | 0.003 | 0.005 | 0.004 | 0.004 | 0.004 | 0.002 | 0.001 | 0.001 | 0.005 | 0.003 | 0.001 | 0.002 | 0.003 |
| meta2084 |  | 738.504 | 120.591 | 0.825 | 0.567 | 0.569 | 0.220 | 0.160 | 0.200 | 0.131 | 0.318 | 0.156 | 0.472 | 0.352 | 0.585 | 0.336 | 0.165 | 0.176 |
| meta2085 |  | 738.530 | 44.759 | 0.051 | 0.061 | 0.057 | 0.067 | 0.096 | 0.045 | 0.078 | 0.076 | 0.074 | 0.037 | 0.027 | 0.038 | 0.093 | 0.082 | 0.185 |
| meta2086 |  | 738.654 | 287.956 | 0.023 | 0.021 | 0.022 | 0.024 | 0.025 | 0.021 | 0.020 | 0.023 | 0.029 | 0.027 | 0.025 | 0.024 | 0.028 | 0.026 | 0.026 |
| meta2087 |  | 738.809 | 318.444 | 0.022 | 0.022 | 0.024 | 0.021 | 0.021 | 0.024 | 0.021 | 0.020 | 0.028 | 0.022 | 0.022 | 0.020 | 0.026 | 0.028 | 0.022 |
| meta2088 |  | 741.080 | 481.114 | 0.009 | 0.007 | 0.008 | 0.007 | 0.009 | 0.007 | 0.009 | 0.008 | 0.011 | 0.007 | 0.005 | 0.009 | 0.017 | 0.011 | 0.017 |
| meta2089 |  | 741.139 | 392.469 | 0.003 | 0.003 | 0.004 | 0.002 | 0.005 | 0.003 | 0.005 | 0.003 | 0.002 | 0.004 | 0.003 | 0.004 | 0.003 | 0.003 | 0.003 |
| meta2090 |  | 742.247 | 340.548 | 0.006 | 0.005 | 0.005 | 0.006 | 0.005 | 0.006 | 0.005 | 0.005 | 0.002 | 0.004 | 0.004 | 0.004 | 0.006 | 0.004 | 0.002 |
| meta2091 |  | 742.561 | 164.464 | 0.004 | 0.006 | 0.006 | 0.004 | 0.005 | 0.006 | 0.004 | 0.001 | 0.009 | 0.001 | 0.004 | 0.004 | 0.007 | 0.004 | 0.005 |
| meta2092 |  | 743.183 | 478.185 | 0.015 | 0.013 | 0.013 | 0.013 | 0.018 | 0.014 | 0.015 | 0.018 | 0.016 | 0.015 | 0.014 | 0.015 | 0.020 | 0.013 | 0.018 |
| meta2093 |  | 743.327 | 168.932 | 0.002 | 0.002 | 0.002 | 0.002 | 0.003 | 0.001 | 0.004 | 0.006 | 0.003 | 0.002 | 0.002 | 0.003 | 0.002 | 0.001 | 0.001 |
| meta2094 | Nicotinamide adenine dinucleotide phosphate (NADP) | 744.080 | 479.754 | 0.046 | 0.041 | 0.044 | 0.037 | 0.050 | 0.044 | 0.051 | 0.051 | 0.062 | 0.042 | 0.034 | 0.052 | 0.087 | 0.058 | 0.076 |
| meta2095 |  | 744.178 | 313.766 | 0.007 | 0.006 | 0.008 | 0.009 | 0.010 | 0.008 | 0.010 | 0.004 | 0.003 | 0.003 | 0.003 | 0.004 | 0.006 | 0.005 | 0.005 |
| meta2096 |  | 744.450 | 44.926 | 0.065 | 0.080 | 0.072 | 0.429 | 0.182 | 0.120 | 0.036 | 0.011 | 0.050 | 0.008 | 0.004 | 0.013 | 0.012 | 0.007 | 0.005 |
| meta2097 |  | 744.550 | 48.111 | 0.021 | 0.015 | 0.014 | 0.431 | 0.181 | 0.002 | 0.023 | 0.014 | 0.004 | 0.007 | 0.017 | 0.017 | 0.005 | 0.004 | 0.005 |
| meta2098 |  | 745.417 | 351.622 | 0.054 | 0.052 | 0.051 | 0.031 | 0.035 | 0.066 | 0.026 | 0.047 | 0.031 | 0.042 | 0.082 | 0.098 | 0.065 | 0.084 | 0.047 |
| meta2099 |  | 748.295 | 462.080 | 0.007 | 0.006 | 0.006 | 0.006 | 0.006 | 0.006 | 0.006 | 0.006 | 0.008 | 0.008 | 0.008 | 0.007 | 0.009 | 0.006 | 0.009 |
| meta2100 |  | 748.708 | 287.939 | 0.003 | 0.003 | 0.003 | 0.004 | 0.004 | 0.003 | 0.004 | 0.003 | 0.004 | 0.004 | 0.005 | 0.004 | 0.003 | 0.003 | 0.004 |
| meta2101 |  | 749.279 | 446.776 | 0.018 | 0.017 | 0.015 | 0.014 | 0.018 | 0.016 | 0.020 | 0.020 | 0.015 | 0.018 | 0.017 | 0.016 | 0.019 | 0.013 | 0.019 |
| meta2102 |  | 749.530 | 46.598 | 0.107 | 0.119 | 0.112 | 0.048 | 0.030 | 0.025 | 0.034 | 0.095 | 0.040 | 0.053 | 0.093 | 0.132 | 0.045 | 0.028 | 0.052 |
| meta2103 |  | 750.241 | 152.450 | 0.004 | 0.006 | 0.006 | 0.004 | 0.010 | 0.005 | 0.015 | 0.007 | 0.002 | 0.001 | 0.003 | 0.006 | 0.003 | 0.004 | 0.005 |
| meta2104 |  | 751.092 | 275.257 | 0.118 | 0.103 | 0.102 | 0.100 | 0.117 | 0.099 | 0.090 | 0.154 | 0.110 | 0.176 | 0.097 | 0.052 | 0.103 | 0.110 | 0.113 |
| meta2105 |  | 751.260 | 429.782 | 0.013 | 0.009 | 0.010 | 0.010 | 0.011 | 0.013 | 0.011 | 0.014 | 0.008 | 0.012 | 0.013 | 0.016 | 0.013 | 0.007 | 0.007 |
| meta2106 |  | 752.362 | 218.501 | 0.005 | 0.004 | 0.004 | 0.004 | 0.004 | 0.003 | 0.005 | 0.002 | 0.002 | 0.003 | 0.004 | 0.003 | 0.004 | 0.003 | 0.006 |
| meta2107 |  | 752.519 | 130.911 | 0.030 | 0.037 | 0.032 | 0.057 | 0.072 | 0.041 | 0.081 | 0.054 | 0.045 | 0.060 | 0.042 | 0.041 | 0.049 | 0.050 | 0.121 |
| meta2108 |  | 752.519 | 118.302 | 0.057 | 0.054 | 0.065 | 0.061 | 0.064 | 0.052 | 0.069 | 0.084 | 0.115 | 0.097 | 0.078 | 0.132 | 0.048 | 0.064 | 0.062 |
| meta2109 |  | 752.567 | 288.221 | 0.007 | 0.007 | 0.009 | 0.008 | 0.007 | 0.007 | 0.009 | 0.008 | 0.010 | 0.009 | 0.009 | 0.008 | 0.010 | 0.009 | 0.008 |
| meta2110 |  | 753.445 | 217.129 | 0.004 | 0.004 | 0.004 | 0.010 | 0.001 | 0.010 | 0.004 | 0.005 | 0.002 | 0.002 | 0.010 | 0.003 | 0.004 | 0.003 | 0.002 |
| meta2111 |  | 754.536 | 129.148 | 0.261 | 0.352 | 0.321 | 0.365 | 0.677 | 0.334 | 1.024 | 0.409 | 0.436 | 0.378 | 0.277 | 0.353 | 0.297 | 0.390 | 0.331 |
| meta2112 |  | 755.097 | 417.377 | 0.011 | 0.010 | 0.010 | 0.010 | 0.009 | 0.007 | 0.010 | 0.013 | 0.013 | 0.011 | 0.010 | 0.011 | 0.013 | 0.009 | 0.014 |
| meta2113 |  | 755.183 | 477.508 | 0.023 | 0.025 | 0.026 | 0.026 | 0.035 | 0.026 | 0.025 | 0.040 | 0.031 | 0.031 | 0.026 | 0.031 | 0.042 | 0.025 | 0.034 |
| meta2114 |  | 755.184 | 152.578 | 0.015 | 0.014 | 0.016 | 0.015 | 0.023 | 0.014 | 0.026 | 0.016 | 0.010 | 0.006 | 0.011 | 0.015 | 0.010 | 0.011 | 0.014 |
| meta2115 |  | 756.478 | 198.005 | 0.005 | 0.006 | 0.005 | 0.008 | 0.010 | 0.004 | 0.003 | 0.007 | 0.007 | 0.006 | 0.005 | 0.003 | 0.004 | 0.004 | 0.005 |
| meta2116 | PC(16:0/16:0) | 756.552 | 129.745 | 4.032 | 4.073 | 5.212 | 4.800 | 8.613 | 2.528 | 8.812 | 5.550 | 3.580 | 5.851 | 3.631 | 5.064 | 6.750 | 3.783 | 3.611 |
| meta2117 | Diadenosine triphosphate | 757.087 | 436.825 | 0.005 | 0.004 | 0.003 | 0.004 | 0.004 | 0.004 | 0.006 | 0.005 | 0.006 | 0.004 | 0.004 | 0.004 | 0.006 | 0.004 | 0.008 |
| meta2118 |  | 757.122 | 454.360 | 0.009 | 0.008 | 0.008 | 0.007 | 0.007 | 0.005 | 0.011 | 0.009 | 0.006 | 0.007 | 0.010 | 0.008 | 0.006 | 0.004 | 0.009 |
| meta2119 |  | 757.406 | 333.518 | 0.019 | 0.024 | 0.017 | 0.015 | 0.016 | 0.030 | 0.011 | 0.019 | 0.008 | 0.015 | 0.040 | 0.018 | 0.016 | 0.021 | 0.009 |
| meta2120 | Thioetheramide-PC | 758.566 | 184.638 | 0.167 | 0.184 | 0.176 | 0.083 | 0.094 | 0.060 | 0.122 | 0.151 | 0.078 | 0.120 | 0.250 | 0.243 | 0.107 | 0.083 | 0.121 |
| meta2121 |  | 758.637 | 29.651 | 0.003 | 0.005 | 0.005 | 0.003 | 0.003 | 0.004 | 0.006 | 0.005 | 0.003 | 0.002 | 0.004 | 0.005 | 0.006 | 0.002 | 0.003 |
| meta2122 |  | 759.091 | 481.583 | 0.011 | 0.012 | 0.011 | 0.011 | 0.012 | 0.010 | 0.013 | 0.013 | 0.016 | 0.009 | 0.008 | 0.013 | 0.020 | 0.014 | 0.021 |
| meta2123 |  | 759.397 | 371.189 | 0.026 | 0.022 | 0.020 | 0.020 | 0.028 | 0.020 | 0.022 | 0.022 | 0.014 | 0.020 | 0.030 | 0.026 | 0.018 | 0.020 | 0.025 |
| meta2124 |  | 760.151 | 468.519 | 0.012 | 0.016 | 0.016 | 0.013 | 0.015 | 0.009 | 0.011 | 0.020 | 0.018 | 0.013 | 0.010 | 0.018 | 0.010 | 0.011 | 0.014 |
| meta2125 |  | 760.286 | 27.382 | 0.016 | 0.019 | 0.020 | 0.019 | 0.018 | 0.013 | 0.016 | 0.023 | 0.019 | 0.017 | 0.019 | 0.017 | 0.015 | 0.014 | 0.017 |
| meta2126 |  | 760.512 | 44.721 | 0.027 | 0.027 | 0.023 | 0.033 | 0.051 | 0.023 | 0.033 | 0.034 | 0.036 | 0.019 | 0.013 | 0.020 | 0.045 | 0.039 | 0.061 |
| meta2127 |  | 760.791 | 318.447 | 0.007 | 0.008 | 0.008 | 0.007 | 0.007 | 0.009 | 0.007 | 0.006 | 0.008 | 0.008 | 0.007 | 0.006 | 0.008 | 0.009 | 0.008 |
| meta2128 |  | 761.422 | 213.300 | 0.002 | 0.002 | 0.002 | 0.002 | 0.003 | 0.002 | 0.003 | 0.003 | 0.001 | 0.002 | 0.004 | 0.001 | 0.002 | 0.001 | 0.003 |
| meta2129 |  | 761.464 | 39.777 | 0.022 | 0.028 | 0.031 | 0.217 | 0.124 | 0.058 | 0.013 | 0.024 | 0.034 | 0.002 | 0.015 | 0.020 | 0.002 | 0.024 | 0.002 |
| meta2130 |  | 762.269 | 362.056 | 0.065 | 0.085 | 0.073 | 0.078 | 0.087 | 0.037 | 0.108 | 0.058 | 0.055 | 0.062 | 0.098 | 0.071 | 0.045 | 0.052 | 0.099 |
| meta2131 |  | 762.504 | 118.199 | 0.710 | 0.604 | 0.661 | 0.333 | 0.254 | 0.220 | 0.240 | 0.570 | 0.394 | 0.539 | 0.591 | 0.427 | 0.485 | 0.272 | 0.262 |
| meta2132 |  | 764.521 | 118.817 | 7.031 | 8.523 | 9.167 | 2.345 | 1.512 | 1.925 | 2.868 | 4.882 | 2.385 | 4.600 | 9.199 | 8.536 | 3.744 | 1.353 | 2.058 |
| meta2133 |  | 764.542 | 45.536 | 0.177 | 0.207 | 0.178 | 0.117 | 0.099 | 0.065 | 0.102 | 0.231 | 0.202 | 0.148 | 0.216 | 0.259 | 0.109 | 0.112 | 0.102 |
| meta2134 |  | 765.228 | 344.223 | 0.045 | 0.051 | 0.044 | 0.041 | 0.055 | 0.061 | 0.067 | 0.043 | 0.029 | 0.044 | 0.062 | 0.067 | 0.032 | 0.027 | 0.053 |
| meta2135 | Bumetanide | 767.196 | 471.455 | 0.010 | 0.010 | 0.009 | 0.009 | 0.008 | 0.012 | 0.009 | 0.009 | 0.015 | 0.009 | 0.008 | 0.008 | 0.009 | 0.006 | 0.008 |
| meta2136 |  | 768.088 | 422.537 | 0.010 | 0.012 | 0.015 | 0.012 | 0.012 | 0.009 | 0.020 | 0.021 | 0.014 | 0.012 | 0.010 | 0.012 | 0.021 | 0.010 | 0.017 |
| meta2137 |  | 768.120 | 275.257 | 0.038 | 0.035 | 0.035 | 0.036 | 0.037 | 0.027 | 0.028 | 0.061 | 0.032 | 0.057 | 0.032 | 0.014 | 0.035 | 0.031 | 0.030 |
| meta2138 | Coenzyme A (CoA) | 768.118 | 479.458 | 0.010 | 0.007 | 0.008 | 0.008 | 0.016 | 0.008 | 0.023 | 0.005 | 0.010 | 0.007 | 0.008 | 0.007 | 0.018 | 0.012 | 0.015 |
| meta2139 |  | 768.551 | 129.768 | 1.606 | 1.830 | 1.600 | 0.790 | 0.951 | 0.263 | 0.943 | 1.454 | 0.657 | 0.784 | 1.666 | 1.858 | 0.751 | 0.281 | 0.549 |
| meta2140 |  | 768.688 | 287.796 | 0.002 | 0.003 | 0.002 | 0.003 | 0.002 | 0.003 | 0.003 | 0.002 | 0.003 | 0.004 | 0.003 | 0.003 | 0.005 | 0.003 | 0.002 |
| meta2141 |  | 770.068 | 275.462 | 0.013 | 0.011 | 0.013 | 0.012 | 0.015 | 0.012 | 0.010 | 0.011 | 0.008 | 0.014 | 0.012 | 0.006 | 0.015 | 0.013 | 0.013 |
| meta2142 |  | 770.278 | 461.346 | 0.005 | 0.005 | 0.004 | 0.003 | 0.003 | 0.003 | 0.003 | 0.003 | 0.005 | 0.004 | 0.006 | 0.004 | 0.005 | 0.004 | 0.004 |
| meta2143 |  | 771.160 | 152.578 | 0.040 | 0.045 | 0.049 | 0.048 | 0.073 | 0.039 | 0.079 | 0.045 | 0.029 | 0.019 | 0.031 | 0.044 | 0.036 | 0.035 | 0.041 |
| meta2144 |  | 771.261 | 447.024 | 0.040 | 0.038 | 0.039 | 0.035 | 0.039 | 0.036 | 0.046 | 0.044 | 0.035 | 0.043 | 0.038 | 0.039 | 0.044 | 0.028 | 0.044 |
| meta2145 |  | 771.513 | 44.721 | 0.098 | 0.126 | 0.118 | 0.083 | 0.078 | 0.048 | 0.094 | 0.109 | 0.073 | 0.081 | 0.112 | 0.156 | 0.108 | 0.075 | 0.087 |
| meta2146 |  | 772.211 | 294.096 | 0.006 | 0.005 | 0.005 | 0.006 | 0.006 | 0.004 | 0.006 | 0.003 | 0.002 | 0.002 | 0.003 | 0.003 | 0.002 | 0.002 | 0.003 |
| meta2147 |  | 772.313 | 361.959 | 0.206 | 0.159 | 0.127 | 0.140 | 0.204 | 0.068 | 0.311 | 0.133 | 0.063 | 0.197 | 0.306 | 0.155 | 0.076 | 0.042 | 0.323 |
| meta2148 |  | 772.612 | 39.847 | 0.002 | 0.003 | 0.002 | 0.010 | 0.007 | 0.004 | 0.004 | 0.005 | 0.003 | 0.002 | 0.002 | 0.002 | 0.002 | 0.002 | 0.002 |
| meta2149 |  | 772.653 | 287.595 | 0.005 | 0.004 | 0.005 | 0.008 | 0.007 | 0.005 | 0.006 | 0.006 | 0.007 | 0.007 | 0.006 | 0.007 | 0.009 | 0.007 | 0.006 |
| meta2150 |  | 773.075 | 275.367 | 0.184 | 0.192 | 0.174 | 0.153 | 0.201 | 0.159 | 0.170 | 0.245 | 0.182 | 0.232 | 0.158 | 0.117 | 0.177 | 0.174 | 0.171 |
| meta2151 |  | 773.194 | 478.546 | 0.025 | 0.022 | 0.030 | 0.021 | 0.024 | 0.023 | 0.027 | 0.034 | 0.026 | 0.026 | 0.026 | 0.026 | 0.033 | 0.020 | 0.027 |
| meta2152 |  | 774.348 | 370.851 | 0.008 | 0.006 | 0.007 | 0.007 | 0.005 | 0.008 | 0.004 | 0.006 | 0.003 | 0.007 | 0.012 | 0.009 | 0.004 | 0.007 | 0.007 |
| meta2153 |  | 774.631 | 287.595 | 0.018 | 0.018 | 0.017 | 0.021 | 0.019 | 0.017 | 0.022 | 0.018 | 0.022 | 0.021 | 0.020 | 0.020 | 0.024 | 0.019 | 0.019 |
| meta2154 |  | 775.209 | 482.702 | 0.118 | 0.117 | 0.130 | 0.118 | 0.143 | 0.131 | 0.143 | 0.155 | 0.140 | 0.121 | 0.115 | 0.129 | 0.168 | 0.123 | 0.139 |
| meta2155 |  | 776.470 | 36.142 | 0.014 | 0.015 | 0.016 | 0.174 | 0.061 | 0.041 | 0.008 | 0.011 | 0.024 | 0.002 | 0.005 | 0.006 | 0.005 | 0.006 | 0.005 |
| meta2156 |  | 776.519 | 118.062 | 0.037 | 0.047 | 0.028 | 0.054 | 0.060 | 0.032 | 0.072 | 0.051 | 0.060 | 0.128 | 0.077 | 0.088 | 0.041 | 0.061 | 0.052 |
| meta2157 |  | 776.573 | 136.209 | 0.050 | 0.058 | 0.060 | 0.067 | 0.060 | 0.032 | 0.089 | 0.095 | 0.055 | 0.050 | 0.070 | 0.077 | 0.044 | 0.056 | 0.060 |
| meta2158 |  | 776.611 | 288.556 | 0.070 | 0.070 | 0.073 | 0.073 | 0.079 | 0.073 | 0.074 | 0.073 | 0.096 | 0.082 | 0.080 | 0.075 | 0.100 | 0.094 | 0.083 |
| meta2159 |  | 777.165 | 478.212 | 0.008 | 0.006 | 0.007 | 0.008 | 0.008 | 0.006 | 0.009 | 0.009 | 0.007 | 0.009 | 0.008 | 0.013 | 0.011 | 0.006 | 0.009 |
| meta2160 |  | 777.503 | 42.937 | 0.012 | 0.017 | 0.015 | 0.022 | 0.024 | 0.015 | 0.026 | 0.021 | 0.023 | 0.017 | 0.024 | 0.027 | 0.022 | 0.022 | 0.022 |
| meta2161 |  | 778.367 | 432.224 | 0.005 | 0.005 | 0.005 | 0.005 | 0.006 | 0.005 | 0.004 | 0.004 | 0.003 | 0.006 | 0.006 | 0.005 | 0.004 | 0.006 | 0.006 |
| meta2162 |  | 778.560 | 45.551 | 0.011 | 0.008 | 0.008 | 0.003 | 0.004 | 0.003 | 0.005 | 0.006 | 0.006 | 0.006 | 0.007 | 0.009 | 0.005 | 0.003 | 0.003 |
| meta2163 |  | 780.100 | 421.238 | 0.014 | 0.011 | 0.010 | 0.009 | 0.012 | 0.009 | 0.015 | 0.012 | 0.012 | 0.011 | 0.015 | 0.012 | 0.017 | 0.007 | 0.010 |
| meta2164 |  | 780.161 | 127.815 | 0.127 | 0.157 | 0.194 | 0.111 | 0.166 | 0.059 | 0.062 | 0.097 | 0.165 | 0.076 | 0.111 | 0.151 | 0.091 | 0.128 | 0.104 |
| meta2165 |  | 780.228 | 343.229 | 0.022 | 0.024 | 0.022 | 0.023 | 0.030 | 0.028 | 0.034 | 0.023 | 0.014 | 0.023 | 0.034 | 0.032 | 0.017 | 0.014 | 0.026 |
| meta2166 |  | 780.280 | 362.008 | 0.071 | 0.088 | 0.066 | 0.097 | 0.094 | 0.046 | 0.096 | 0.062 | 0.043 | 0.088 | 0.097 | 0.070 | 0.041 | 0.039 | 0.109 |
| meta2167 |  | 781.148 | 287.239 | 0.004 | 0.003 | 0.004 | 0.004 | 0.003 | 0.003 | 0.003 | 0.003 | 0.004 | 0.005 | 0.004 | 0.003 | 0.003 | 0.003 | 0.003 |
| meta2168 |  | 782.099 | 464.715 | 0.016 | 0.012 | 0.012 | 0.008 | 0.013 | 0.007 | 0.017 | 0.010 | 0.016 | 0.011 | 0.013 | 0.013 | 0.016 | 0.015 | 0.020 |
| meta2169 |  | 782.243 | 349.321 | 0.007 | 0.008 | 0.005 | 0.007 | 0.008 | 0.009 | 0.013 | 0.010 | 0.003 | 0.007 | 0.007 | 0.014 | 0.006 | 0.004 | 0.006 |
| meta2170 |  | 782.569 | 126.960 | 62.432 | 48.310 | 66.788 | 39.516 | 27.775 | 20.311 | 46.670 | 57.017 | 72.091 | 31.121 | 62.856 | 60.805 | 62.357 | 56.441 | 64.515 |
| meta2171 |  | 783.162 | 399.459 | 0.032 | 0.038 | 0.033 | 0.036 | 0.034 | 0.031 | 0.032 | 0.038 | 0.027 | 0.031 | 0.033 | 0.035 | 0.036 | 0.034 | 0.040 |
| meta2172 |  | 783.168 | 466.635 | 0.004 | 0.005 | 0.004 | 0.003 | 0.002 | 0.003 | 0.005 | 0.001 | 0.006 | 0.006 | 0.004 | 0.007 | 0.005 | 0.007 | 0.005 |
| meta2173 |  | 783.337 | 162.777 | 0.012 | 0.017 | 0.021 | 0.003 | 0.006 | 0.016 | 0.015 | 0.033 | 0.006 | 0.033 | 0.017 | 0.008 | 0.017 | 0.035 | 0.015 |
| meta2174 |  | 783.588 | 44.245 | 0.054 | 0.055 | 0.054 | 0.037 | 0.056 | 0.025 | 0.048 | 0.052 | 0.044 | 0.044 | 0.050 | 0.023 | 0.073 | 0.062 | 0.111 |
| meta2175 |  | 784.210 | 475.543 | 0.004 | 0.004 | 0.003 | 0.004 | 0.004 | 0.004 | 0.004 | 0.004 | 0.005 | 0.005 | 0.006 | 0.006 | 0.006 | 0.004 | 0.003 |
| meta2176 | Flavin adenine dinucleotide (FAD) | 786.163 | 384.430 | 0.371 | 0.325 | 0.310 | 0.272 | 0.362 | 0.272 | 0.350 | 0.377 | 0.353 | 0.363 | 0.361 | 0.352 | 0.393 | 0.293 | 0.403 |
| meta2177 |  | 786.211 | 406.201 | 0.010 | 0.011 | 0.011 | 0.009 | 0.010 | 0.014 | 0.011 | 0.013 | 0.008 | 0.011 | 0.011 | 0.015 | 0.009 | 0.006 | 0.006 |
| meta2178 |  | 786.503 | 118.003 | 0.302 | 0.379 | 0.420 | 0.188 | 0.134 | 0.224 | 0.161 | 0.192 | 0.207 | 0.240 | 0.288 | 0.340 | 0.280 | 0.229 | 0.171 |
| meta2179 |  | 787.250 | 462.109 | 0.013 | 0.017 | 0.012 | 0.010 | 0.013 | 0.014 | 0.015 | 0.012 | 0.012 | 0.010 | 0.008 | 0.016 | 0.016 | 0.011 | 0.020 |
| meta2180 |  | 788.228 | 468.547 | 0.013 | 0.010 | 0.012 | 0.009 | 0.010 | 0.015 | 0.013 | 0.009 | 0.010 | 0.013 | 0.013 | 0.017 | 0.015 | 0.006 | 0.006 |
| meta2181 |  | 788.541 | 44.001 | 0.097 | 0.104 | 0.097 | 0.086 | 0.103 | 0.061 | 0.173 | 0.119 | 0.102 | 0.112 | 0.133 | 0.176 | 0.187 | 0.137 | 0.119 |
| meta2182 |  | 788.760 | 287.270 | 0.003 | 0.003 | 0.002 | 0.002 | 0.003 | 0.002 | 0.002 | 0.002 | 0.002 | 0.003 | 0.003 | 0.003 | 0.003 | 0.002 | 0.002 |
| meta2183 |  | 789.042 | 275.926 | 0.012 | 0.012 | 0.015 | 0.015 | 0.017 | 0.011 | 0.017 | 0.018 | 0.014 | 0.013 | 0.012 | 0.013 | 0.014 | 0.012 | 0.012 |
| meta2184 |  | 789.178 | 472.034 | 0.017 | 0.026 | 0.020 | 0.018 | 0.018 | 0.014 | 0.021 | 0.029 | 0.026 | 0.024 | 0.025 | 0.020 | 0.015 | 0.014 | 0.019 |
| meta2185 |  | 789.426 | 281.474 | 0.005 | 0.005 | 0.005 | 0.004 | 0.004 | 0.004 | 0.003 | 0.003 | 0.003 | 0.005 | 0.004 | 0.006 | 0.004 | 0.005 | 0.005 |
| meta2186 |  | 791.204 | 479.074 | 0.016 | 0.015 | 0.016 | 0.015 | 0.019 | 0.016 | 0.015 | 0.020 | 0.017 | 0.016 | 0.014 | 0.018 | 0.019 | 0.013 | 0.016 |
| meta2187 |  | 791.255 | 464.690 | 0.005 | 0.005 | 0.006 | 0.004 | 0.004 | 0.008 | 0.004 | 0.008 | 0.004 | 0.009 | 0.008 | 0.005 | 0.006 | 0.004 | 0.003 |
| meta2188 |  | 791.378 | 162.333 | 0.001 | 0.002 | 0.001 | 0.000 | 0.000 | 0.002 | 0.002 | 0.005 | 0.001 | 0.004 | 0.002 | 0.001 | 0.003 | 0.004 | 0.002 |
| meta2189 |  | 792.550 | 116.593 | 2.796 | 2.463 | 2.656 | 0.571 | 0.440 | 0.301 | 0.514 | 1.284 | 1.224 | 1.251 | 2.590 | 1.120 | 0.896 | 0.352 | 0.875 |
| meta2190 |  | 793.140 | 152.578 | 0.008 | 0.008 | 0.009 | 0.010 | 0.013 | 0.008 | 0.011 | 0.009 | 0.007 | 0.006 | 0.007 | 0.008 | 0.008 | 0.007 | 0.009 |
| meta2191 |  | 793.475 | 232.324 | 0.002 | 0.003 | 0.002 | 0.002 | 0.003 | 0.002 | 0.002 | 0.003 | 0.001 | 0.001 | 0.003 | 0.002 | 0.002 | 0.001 | 0.002 |
| meta2192 |  | 794.296 | 362.081 | 1.579 | 1.827 | 1.439 | 1.478 | 1.838 | 0.925 | 2.234 | 1.406 | 0.982 | 1.762 | 2.001 | 1.606 | 1.039 | 0.790 | 2.067 |
| meta2193 |  | 794.564 | 126.683 | 0.405 | 0.367 | 0.467 | 0.535 | 0.461 | 0.301 | 0.263 | 0.395 | 0.370 | 0.335 | 0.338 | 1.112 | 0.561 | 0.392 | 0.506 |
| meta2194 |  | 795.213 | 371.913 | 0.004 | 0.004 | 0.003 | 0.003 | 0.004 | 0.003 | 0.004 | 0.004 | 0.002 | 0.003 | 0.007 | 0.004 | 0.002 | 0.002 | 0.002 |
| meta2195 |  | 795.285 | 481.194 | 0.031 | 0.026 | 0.029 | 0.026 | 0.032 | 0.042 | 0.030 | 0.038 | 0.031 | 0.030 | 0.033 | 0.037 | 0.041 | 0.024 | 0.023 |
| meta2196 |  | 795.514 | 43.231 | 0.054 | 0.066 | 0.064 | 0.074 | 0.074 | 0.048 | 0.087 | 0.069 | 0.056 | 0.059 | 0.071 | 0.089 | 0.081 | 0.065 | 0.066 |
| meta2197 |  | 796.069 | 434.587 | 0.018 | 0.016 | 0.016 | 0.013 | 0.016 | 0.013 | 0.018 | 0.021 | 0.021 | 0.018 | 0.016 | 0.024 | 0.019 | 0.016 | 0.020 |
| meta2198 |  | 796.330 | 371.351 | 0.004 | 0.004 | 0.003 | 0.005 | 0.003 | 0.004 | 0.002 | 0.004 | 0.001 | 0.003 | 0.007 | 0.006 | 0.003 | 0.003 | 0.003 |
| meta2199 |  | 796.524 | 133.318 | 0.516 | 0.866 | 0.498 | 0.400 | 0.309 | 0.321 | 0.536 | 0.311 | 0.564 | 0.522 | 0.824 | 0.895 | 0.591 | 0.254 | 0.300 |
| meta2200 |  | 796.590 | 289.276 | 0.004 | 0.005 | 0.005 | 0.006 | 0.005 | 0.006 | 0.005 | 0.005 | 0.009 | 0.006 | 0.005 | 0.005 | 0.009 | 0.007 | 0.006 |
| meta2201 |  | 797.191 | 482.743 | 0.011 | 0.011 | 0.012 | 0.012 | 0.012 | 0.010 | 0.012 | 0.016 | 0.013 | 0.012 | 0.014 | 0.012 | 0.014 | 0.011 | 0.012 |
| meta2202 | Sphingomyelin (d18:1/18:0) | 797.541 | 171.524 | 0.008 | 0.011 | 0.008 | 0.012 | 0.016 | 0.008 | 0.019 | 0.018 | 0.007 | 0.010 | 0.014 | 0.013 | 0.011 | 0.004 | 0.020 |
| meta2203 |  | 798.913 | 286.164 | 0.004 | 0.003 | 0.003 | 0.002 | 0.004 | 0.003 | 0.003 | 0.004 | 0.002 | 0.003 | 0.004 | 0.004 | 0.003 | 0.003 | 0.003 |
| meta2204 |  | 799.543 | 44.406 | 0.019 | 0.024 | 0.022 | 0.019 | 0.019 | 0.006 | 0.017 | 0.020 | 0.014 | 0.013 | 0.021 | 0.023 | 0.016 | 0.012 | 0.017 |
| meta2205 |  | 800.371 | 228.410 | 0.006 | 0.005 | 0.006 | 0.003 | 0.006 | 0.004 | 0.008 | 0.007 | 0.003 | 0.005 | 0.005 | 0.005 | 0.004 | 0.004 | 0.004 |
| meta2206 |  | 800.809 | 318.444 | 0.023 | 0.023 | 0.028 | 0.023 | 0.024 | 0.028 | 0.025 | 0.022 | 0.030 | 0.023 | 0.025 | 0.022 | 0.028 | 0.034 | 0.024 |
| meta2207 |  | 801.174 | 399.920 | 0.015 | 0.016 | 0.021 | 0.015 | 0.022 | 0.016 | 0.014 | 0.030 | 0.016 | 0.020 | 0.017 | 0.016 | 0.018 | 0.014 | 0.019 |
| meta2208 |  | 801.503 | 42.531 | 0.029 | 0.040 | 0.037 | 0.061 | 0.058 | 0.043 | 0.067 | 0.045 | 0.056 | 0.052 | 0.079 | 0.088 | 0.062 | 0.075 | 0.074 |
| meta2209 |  | 802.081 | 421.247 | 0.008 | 0.006 | 0.007 | 0.007 | 0.008 | 0.005 | 0.008 | 0.006 | 0.007 | 0.007 | 0.009 | 0.009 | 0.007 | 0.005 | 0.007 |
| meta2210 |  | 802.156 | 389.000 | 0.013 | 0.011 | 0.011 | 0.010 | 0.012 | 0.009 | 0.014 | 0.013 | 0.010 | 0.009 | 0.011 | 0.014 | 0.010 | 0.015 | 0.013 |
| meta2211 |  | 802.281 | 361.838 | 0.036 | 0.046 | 0.031 | 0.043 | 0.045 | 0.014 | 0.046 | 0.026 | 0.012 | 0.038 | 0.050 | 0.034 | 0.017 | 0.015 | 0.057 |
| meta2212 |  | 802.476 | 119.586 | 0.038 | 0.071 | 0.045 | 0.034 | 0.021 | 0.023 | 0.027 | 0.061 | 0.033 | 0.018 | 0.058 | 0.061 | 0.077 | 0.026 | 0.028 |
| meta2213 |  | 803.441 | 210.507 | 0.003 | 0.002 | 0.003 | 0.002 | 0.003 | 0.002 | 0.002 | 0.002 | 0.002 | 0.002 | 0.003 | 0.003 | 0.003 | 0.002 | 0.003 |
| meta2214 |  | 804.549 | 152.603 | 0.112 | 0.118 | 0.096 | 0.127 | 0.128 | 0.403 | 0.132 | 0.104 | 0.071 | 0.578 | 0.092 | 0.109 | 0.142 | 0.553 | 0.555 |
| meta2215 |  | 805.319 | 162.527 | 0.003 | 0.004 | 0.003 | 0.001 | 0.002 | 0.003 | 0.003 | 0.007 | 0.002 | 0.005 | 0.003 | 0.002 | 0.002 | 0.005 | 0.004 |
| meta2216 |  | 806.570 | 125.862 | 21.817 | 29.572 | 27.706 | 31.548 | 43.864 | 18.766 | 34.147 | 31.001 | 28.297 | 20.385 | 28.640 | 24.618 | 19.519 | 21.881 | 26.758 |
| meta2217 |  | 808.143 | 384.399 | 0.037 | 0.034 | 0.034 | 0.031 | 0.041 | 0.029 | 0.040 | 0.039 | 0.035 | 0.038 | 0.036 | 0.039 | 0.039 | 0.030 | 0.041 |
| meta2218 |  | 808.347 | 313.915 | 0.003 | 0.003 | 0.003 | 0.002 | 0.002 | 0.003 | 0.002 | 0.002 | 0.001 | 0.002 | 0.004 | 0.003 | 0.001 | 0.003 | 0.001 |
| meta2219 |  | 808.510 | 175.212 | 0.159 | 0.175 | 0.164 | 0.128 | 0.091 | 0.069 | 0.090 | 0.165 | 0.099 | 0.109 | 0.159 | 0.194 | 0.131 | 0.082 | 0.093 |
| meta2220 | Acetyl coenzyme A (Acetyl-CoA) | 810.131 | 416.748 | 0.023 | 0.022 | 0.020 | 0.023 | 0.024 | 0.017 | 0.031 | 0.011 | 0.026 | 0.014 | 0.013 | 0.016 | 0.043 | 0.019 | 0.032 |
| meta2221 |  | 810.269 | 361.959 | 0.090 | 0.097 | 0.058 | 0.067 | 0.100 | 0.044 | 0.146 | 0.075 | 0.046 | 0.073 | 0.117 | 0.077 | 0.041 | 0.032 | 0.127 |
| meta2222 |  | 810.609 | 288.416 | 0.005 | 0.005 | 0.005 | 0.006 | 0.006 | 0.005 | 0.006 | 0.006 | 0.008 | 0.006 | 0.009 | 0.006 | 0.008 | 0.006 | 0.005 |
| meta2223 | N-(15Z-Tetracosenoyl)-1-.beta.-galactosylsphing-4-enine | 810.676 | 49.217 | 0.005 | 0.006 | 0.006 | 0.001 | 0.001 | 0.001 | 0.001 | 0.003 | 0.001 | 0.002 | 0.005 | 0.007 | 0.003 | 0.001 | 0.002 |
| meta2224 |  | 811.745 | 477.604 | 0.052 | 0.034 | 0.039 | 0.034 | 0.059 | 0.028 | 0.049 | 0.052 | 0.041 | 0.042 | 0.047 | 0.050 | 0.051 | 0.042 | 0.063 |
| meta2225 |  | 814.220 | 476.847 | 0.016 | 0.016 | 0.015 | 0.011 | 0.014 | 0.012 | 0.011 | 0.018 | 0.016 | 0.016 | 0.015 | 0.019 | 0.020 | 0.015 | 0.015 |
| meta2226 |  | 814.556 | 42.855 | 0.083 | 0.083 | 0.073 | 0.094 | 0.089 | 0.054 | 0.100 | 0.090 | 0.057 | 0.069 | 0.088 | 0.106 | 0.095 | 0.064 | 0.090 |
| meta2227 |  | 814.567 | 288.425 | 0.027 | 0.029 | 0.032 | 0.031 | 0.032 | 0.033 | 0.031 | 0.031 | 0.043 | 0.035 | 0.035 | 0.030 | 0.042 | 0.036 | 0.033 |
| meta2228 |  | 816.105 | 471.017 | 0.012 | 0.008 | 0.007 | 0.009 | 0.010 | 0.009 | 0.014 | 0.010 | 0.010 | 0.008 | 0.012 | 0.009 | 0.013 | 0.012 | 0.014 |
| meta2229 |  | 817.308 | 480.892 | 0.002 | 0.002 | 0.002 | 0.002 | 0.003 | 0.003 | 0.003 | 0.001 | 0.001 | 0.001 | 0.002 | 0.004 | 0.002 | 0.004 | 0.003 |
| meta2230 |  | 817.497 | 42.557 | 0.032 | 0.034 | 0.039 | 0.061 | 0.054 | 0.041 | 0.063 | 0.039 | 0.044 | 0.040 | 0.050 | 0.056 | 0.048 | 0.051 | 0.051 |
| meta2231 |  | 818.719 | 33.023 | 0.003 | 0.002 | 0.002 | 0.001 | 0.001 | 0.001 | 0.000 | 0.002 | 0.001 | 0.001 | 0.001 | 0.002 | 0.001 | 0.000 | 0.001 |
| meta2232 |  | 819.515 | 42.055 | 0.095 | 0.127 | 0.122 | 0.210 | 0.195 | 0.138 | 0.227 | 0.134 | 0.159 | 0.155 | 0.232 | 0.253 | 0.204 | 0.206 | 0.223 |
| meta2233 |  | 820.496 | 38.471 | 0.018 | 0.025 | 0.028 | 0.323 | 0.086 | 0.043 | 0.055 | 0.056 | 0.062 | 0.004 | 0.007 | 0.030 | 0.062 | 0.081 | 0.046 |
| meta2234 |  | 820.581 | 125.107 | 0.251 | 0.341 | 0.369 | 0.561 | 0.411 | 0.153 | 0.320 | 0.308 | 0.238 | 0.593 | 0.596 | 0.370 | 0.212 | 0.298 | 0.248 |
| meta2235 |  | 820.582 | 107.832 | 0.402 | 0.562 | 0.392 | 0.241 | 0.152 | 0.024 | 0.235 | 0.439 | 0.319 | 0.243 | 0.377 | 0.506 | 0.213 | 0.124 | 0.193 |
| meta2236 |  | 821.400 | 275.741 | 0.003 | 0.004 | 0.002 | 0.002 | 0.002 | 0.003 | 0.002 | 0.003 | 0.001 | 0.003 | 0.006 | 0.007 | 0.003 | 0.004 | 0.003 |
| meta2237 |  | 822.792 | 318.447 | 0.019 | 0.018 | 0.022 | 0.018 | 0.020 | 0.022 | 0.020 | 0.016 | 0.025 | 0.017 | 0.019 | 0.017 | 0.023 | 0.025 | 0.020 |
| meta2238 |  | 824.112 | 384.430 | 0.003 | 0.004 | 0.003 | 0.004 | 0.004 | 0.003 | 0.005 | 0.004 | 0.004 | 0.004 | 0.003 | 0.004 | 0.002 | 0.003 | 0.005 |
| meta2239 |  | 824.146 | 404.674 | 0.023 | 0.022 | 0.021 | 0.028 | 0.026 | 0.020 | 0.030 | 0.016 | 0.013 | 0.010 | 0.012 | 0.015 | 0.026 | 0.016 | 0.021 |
| meta2240 |  | 825.504 | 41.391 | 0.010 | 0.010 | 0.011 | 0.025 | 0.031 | 0.019 | 0.028 | 0.016 | 0.024 | 0.017 | 0.024 | 0.026 | 0.016 | 0.022 | 0.022 |
| meta2241 |  | 826.221 | 478.644 | 0.004 | 0.006 | 0.006 | 0.005 | 0.004 | 0.006 | 0.005 | 0.007 | 0.005 | 0.007 | 0.006 | 0.008 | 0.006 | 0.007 | 0.005 |
| meta2242 |  | 827.217 | 336.672 | 0.003 | 0.005 | 0.003 | 0.003 | 0.004 | 0.006 | 0.002 | 0.008 | 0.003 | 0.006 | 0.005 | 0.004 | 0.003 | 0.003 | 0.002 |
| meta2243 |  | 827.222 | 202.042 | 0.006 | 0.005 | 0.006 | 0.009 | 0.009 | 0.005 | 0.007 | 0.004 | 0.004 | 0.004 | 0.005 | 0.009 | 0.005 | 0.006 | 0.005 |
| meta2244 |  | 827.387 | 408.232 | 0.021 | 0.020 | 0.018 | 0.018 | 0.021 | 0.020 | 0.021 | 0.021 | 0.017 | 0.021 | 0.026 | 0.021 | 0.020 | 0.019 | 0.021 |
| meta2245 |  | 828.407 | 367.496 | 0.002 | 0.003 | 0.002 | 0.003 | 0.002 | 0.004 | 0.002 | 0.003 | 0.001 | 0.002 | 0.004 | 0.004 | 0.002 | 0.004 | 0.003 |
| meta2246 |  | 829.133 | 461.310 | 0.013 | 0.013 | 0.014 | 0.011 | 0.013 | 0.012 | 0.016 | 0.020 | 0.014 | 0.022 | 0.018 | 0.011 | 0.015 | 0.010 | 0.016 |
| meta2247 |  | 829.210 | 429.147 | 0.014 | 0.010 | 0.012 | 0.009 | 0.010 | 0.015 | 0.011 | 0.015 | 0.010 | 0.011 | 0.014 | 0.015 | 0.011 | 0.006 | 0.007 |
| meta2248 | Maltopentaose | 829.279 | 485.286 | 0.013 | 0.011 | 0.016 | 0.010 | 0.013 | 0.020 | 0.014 | 0.018 | 0.011 | 0.015 | 0.012 | 0.017 | 0.014 | 0.008 | 0.009 |
| meta2249 |  | 830.125 | 384.404 | 0.009 | 0.009 | 0.009 | 0.008 | 0.010 | 0.008 | 0.010 | 0.010 | 0.010 | 0.011 | 0.010 | 0.011 | 0.009 | 0.007 | 0.010 |
| meta2250 | 1,2-dioleoyl-sn-glycero-3-phosphatidylcholine | 830.565 | 124.501 | 1.091 | 1.018 | 1.014 | 1.696 | 2.492 | 1.126 | 1.924 | 1.171 | 2.062 | 1.390 | 1.365 | 1.285 | 1.407 | 1.552 | 2.153 |
| meta2251 |  | 831.455 | 344.806 | 0.005 | 0.005 | 0.004 | 0.003 | 0.004 | 0.003 | 0.003 | 0.006 | 0.006 | 0.007 | 0.004 | 0.005 | 0.006 | 0.003 | 0.004 |
| meta2252 |  | 832.502 | 44.512 | 0.050 | 0.062 | 0.059 | 0.279 | 0.148 | 0.089 | 0.034 | 0.011 | 0.045 | 0.011 | 0.017 | 0.025 | 0.013 | 0.016 | 0.017 |
| meta2253 | 1-Stearoyl-2-oleoyl-sn-glycerol 3-phosphocholine (SOPC) | 832.580 | 152.603 | 0.182 | 0.199 | 0.164 | 0.089 | 0.703 | 0.037 | 0.589 | 0.143 | 0.746 | 0.522 | 0.165 | 0.186 | 0.119 | 0.043 | 0.756 |
| meta2254 |  | 834.312 | 27.682 | 0.006 | 0.009 | 0.008 | 0.009 | 0.008 | 0.007 | 0.007 | 0.010 | 0.008 | 0.006 | 0.008 | 0.008 | 0.008 | 0.008 | 0.009 |
| meta2255 |  | 834.524 | 42.263 | 0.023 | 0.031 | 0.027 | 0.095 | 0.072 | 0.059 | 0.076 | 0.048 | 0.043 | 0.055 | 0.063 | 0.070 | 0.054 | 0.067 | 0.067 |
| meta2256 |  | 835.662 | 153.529 | 0.007 | 0.006 | 0.005 | 0.000 | 0.001 | 0.000 | 0.000 | 0.003 | 0.000 | 0.001 | 0.005 | 0.009 | 0.001 | 0.000 | 0.001 |
| meta2257 |  | 836.541 | 173.964 | 0.437 | 0.451 | 0.445 | 0.174 | 0.110 | 0.081 | 0.135 | 0.402 | 0.228 | 0.177 | 0.444 | 0.502 | 0.180 | 0.076 | 0.149 |
| meta2258 |  | 836.542 | 41.837 | 0.252 | 0.327 | 0.303 | 0.593 | 0.635 | 0.424 | 0.751 | 0.413 | 0.420 | 0.508 | 0.704 | 0.813 | 0.629 | 0.658 | 0.677 |
| meta2259 |  | 836.607 | 107.202 | 0.861 | 1.267 | 1.046 | 0.424 | 0.333 | 0.162 | 0.351 | 0.920 | 0.347 | 0.395 | 0.654 | 0.995 | 0.378 | 0.191 | 0.340 |
| meta2260 |  | 837.248 | 474.540 | 0.003 | 0.004 | 0.005 | 0.002 | 0.002 | 0.004 | 0.003 | 0.006 | 0.006 | 0.004 | 0.006 | 0.004 | 0.004 | 0.003 | 0.006 |
| meta2261 | Isobutyryl-CoA | 838.162 | 393.389 | 0.016 | 0.015 | 0.014 | 0.016 | 0.018 | 0.012 | 0.019 | 0.015 | 0.014 | 0.012 | 0.012 | 0.012 | 0.022 | 0.012 | 0.022 |
| meta2262 |  | 838.610 | 288.556 | 0.027 | 0.027 | 0.033 | 0.031 | 0.033 | 0.032 | 0.033 | 0.030 | 0.040 | 0.036 | 0.034 | 0.031 | 0.043 | 0.037 | 0.036 |
| meta2263 |  | 839.482 | 42.114 | 0.006 | 0.006 | 0.008 | 0.013 | 0.011 | 0.009 | 0.011 | 0.008 | 0.010 | 0.010 | 0.010 | 0.010 | 0.010 | 0.012 | 0.010 |
| meta2264 |  | 840.621 | 129.447 | 0.116 | 0.103 | 0.113 | 0.084 | 0.085 | 0.046 | 0.050 | 0.076 | 0.097 | 0.050 | 0.098 | 0.162 | 0.121 | 0.068 | 0.076 |
| meta2265 |  | 841.401 | 367.794 | 0.003 | 0.002 | 0.003 | 0.002 | 0.002 | 0.003 | 0.003 | 0.004 | 0.004 | 0.003 | 0.003 | 0.003 | 0.007 | 0.006 | 0.006 |
| meta2266 |  | 841.496 | 41.837 | 0.062 | 0.070 | 0.079 | 0.106 | 0.096 | 0.083 | 0.117 | 0.070 | 0.091 | 0.096 | 0.115 | 0.124 | 0.111 | 0.123 | 0.123 |
| meta2267 |  | 842.583 | 42.105 | 0.027 | 0.031 | 0.026 | 0.023 | 0.025 | 0.013 | 0.019 | 0.028 | 0.017 | 0.016 | 0.035 | 0.031 | 0.018 | 0.013 | 0.017 |
| meta2268 |  | 843.194 | 202.147 | 0.013 | 0.014 | 0.015 | 0.016 | 0.015 | 0.012 | 0.013 | 0.013 | 0.007 | 0.011 | 0.014 | 0.013 | 0.011 | 0.017 | 0.015 |
| meta2269 |  | 844.177 | 478.905 | 0.005 | 0.004 | 0.006 | 0.005 | 0.005 | 0.006 | 0.006 | 0.006 | 0.007 | 0.007 | 0.006 | 0.005 | 0.005 | 0.003 | 0.004 |
| meta2270 |  | 844.291 | 453.083 | 0.076 | 0.067 | 0.073 | 0.064 | 0.072 | 0.101 | 0.075 | 0.101 | 0.058 | 0.069 | 0.074 | 0.101 | 0.063 | 0.040 | 0.048 |
| meta2271 |  | 844.523 | 126.376 | 0.227 | 0.209 | 0.191 | 0.322 | 0.375 | 0.450 | 0.341 | 0.228 | 0.239 | 0.426 | 0.166 | 0.155 | 0.153 | 0.176 | 0.263 |
| meta2272 |  | 844.728 | 32.195 | 0.011 | 0.011 | 0.011 | 0.003 | 0.002 | 0.001 | 0.002 | 0.007 | 0.002 | 0.003 | 0.004 | 0.020 | 0.003 | 0.001 | 0.002 |
| meta2273 |  | 845.434 | 367.794 | 0.004 | 0.004 | 0.003 | 0.004 | 0.003 | 0.006 | 0.002 | 0.003 | 0.003 | 0.003 | 0.008 | 0.007 | 0.005 | 0.004 | 0.002 |
| meta2274 |  | 846.751 | 32.195 | 0.030 | 0.026 | 0.019 | 0.002 | 0.003 | 0.002 | 0.003 | 0.037 | 0.002 | 0.011 | 0.014 | 0.030 | 0.008 | 0.002 | 0.004 |
| meta2275 |  | 848.513 | 42.557 | 0.055 | 0.065 | 0.063 | 0.559 | 0.220 | 0.134 | 0.035 | 0.013 | 0.076 | 0.009 | 0.015 | 0.013 | 0.012 | 0.007 | 0.005 |
| meta2276 |  | 848.663 | 288.144 | 0.003 | 0.003 | 0.002 | 0.003 | 0.004 | 0.002 | 0.002 | 0.002 | 0.003 | 0.003 | 0.003 | 0.003 | 0.004 | 0.003 | 0.004 |
| meta2277 |  | 849.211 | 202.642 | 0.008 | 0.010 | 0.011 | 0.010 | 0.012 | 0.008 | 0.010 | 0.009 | 0.008 | 0.008 | 0.011 | 0.010 | 0.010 | 0.010 | 0.009 |
| meta2278 |  | 849.245 | 452.370 | 0.048 | 0.054 | 0.053 | 0.048 | 0.050 | 0.071 | 0.053 | 0.071 | 0.044 | 0.049 | 0.056 | 0.071 | 0.042 | 0.031 | 0.035 |
| meta2279 |  | 849.368 | 408.283 | 0.004 | 0.005 | 0.006 | 0.005 | 0.005 | 0.005 | 0.005 | 0.004 | 0.003 | 0.005 | 0.006 | 0.005 | 0.004 | 0.005 | 0.005 |
| meta2280 |  | 850.540 | 179.722 | 0.006 | 0.006 | 0.006 | 0.003 | 0.003 | 0.002 | 0.003 | 0.006 | 0.005 | 0.004 | 0.007 | 0.007 | 0.004 | 0.004 | 0.002 |
| meta2281 |  | 850.572 | 45.864 | 0.008 | 0.007 | 0.007 | 0.005 | 0.005 | 0.003 | 0.007 | 0.010 | 0.005 | 0.006 | 0.006 | 0.011 | 0.006 | 0.005 | 0.005 |
| meta2282 |  | 851.191 | 429.147 | 0.009 | 0.007 | 0.008 | 0.006 | 0.006 | 0.009 | 0.007 | 0.010 | 0.006 | 0.007 | 0.009 | 0.009 | 0.008 | 0.005 | 0.005 |
| meta2283 | 3-Methylbutanoyl-CoA | 852.177 | 384.851 | 0.023 | 0.021 | 0.018 | 0.024 | 0.027 | 0.017 | 0.030 | 0.018 | 0.017 | 0.012 | 0.015 | 0.014 | 0.019 | 0.014 | 0.024 |
| meta2284 |  | 852.587 | 45.275 | 0.004 | 0.007 | 0.006 | 0.006 | 0.005 | 0.003 | 0.003 | 0.004 | 0.004 | 0.003 | 0.006 | 0.007 | 0.003 | 0.004 | 0.004 |
| meta2285 |  | 853.560 | 180.405 | 0.028 | 0.031 | 0.030 | 0.010 | 0.007 | 0.006 | 0.008 | 0.032 | 0.020 | 0.018 | 0.036 | 0.032 | 0.014 | 0.009 | 0.009 |
| meta2286 |  | 854.157 | 397.480 | 0.011 | 0.010 | 0.011 | 0.008 | 0.025 | 0.003 | 0.034 | 0.003 | 0.008 | 0.004 | 0.004 | 0.007 | 0.018 | 0.012 | 0.005 |
| meta2287 |  | 856.579 | 151.968 | 0.028 | 0.027 | 0.026 | 0.022 | 0.022 | 0.008 | 0.021 | 0.032 | 0.013 | 0.012 | 0.032 | 0.027 | 0.013 | 0.011 | 0.019 |
| meta2288 |  | 856.719 | 287.075 | 0.004 | 0.003 | 0.002 | 0.003 | 0.003 | 0.003 | 0.003 | 0.004 | 0.004 | 0.004 | 0.003 | 0.003 | 0.003 | 0.004 | 0.002 |
| meta2289 |  | 857.268 | 482.702 | 0.004 | 0.003 | 0.003 | 0.003 | 0.005 | 0.002 | 0.006 | 0.002 | 0.004 | 0.003 | 0.002 | 0.003 | 0.004 | 0.004 | 0.007 |
| meta2290 |  | 857.470 | 42.055 | 0.014 | 0.017 | 0.016 | 0.025 | 0.021 | 0.021 | 0.025 | 0.017 | 0.021 | 0.021 | 0.025 | 0.029 | 0.026 | 0.026 | 0.022 |
| meta2291 |  | 858.525 | 41.197 | 0.015 | 0.018 | 0.018 | 0.032 | 0.029 | 0.024 | 0.029 | 0.023 | 0.028 | 0.031 | 0.038 | 0.044 | 0.022 | 0.039 | 0.053 |
| meta2292 |  | 859.530 | 176.504 | 0.070 | 0.086 | 0.086 | 0.042 | 0.026 | 0.020 | 0.030 | 0.084 | 0.052 | 0.051 | 0.085 | 0.090 | 0.036 | 0.038 | 0.041 |
| meta2293 |  | 860.541 | 40.842 | 0.082 | 0.096 | 0.099 | 0.245 | 0.318 | 0.173 | 0.255 | 0.130 | 0.148 | 0.139 | 0.192 | 0.222 | 0.147 | 0.181 | 0.198 |
| meta2294 |  | 860.766 | 33.352 | 0.004 | 0.003 | 0.002 | 0.000 | 0.000 | 0.000 | 0.000 | 0.001 | 0.000 | 0.001 | 0.004 | 0.005 | 0.001 | 0.000 | 0.001 |
| meta2295 |  | 861.241 | 351.718 | 0.004 | 0.005 | 0.004 | 0.005 | 0.005 | 0.006 | 0.005 | 0.005 | 0.003 | 0.004 | 0.005 | 0.007 | 0.004 | 0.003 | 0.003 |
| meta2296 |  | 861.300 | 485.194 | 0.005 | 0.006 | 0.005 | 0.004 | 0.008 | 0.005 | 0.006 | 0.005 | 0.004 | 0.003 | 0.003 | 0.007 | 0.005 | 0.005 | 0.005 |
| meta2297 |  | 863.481 | 41.781 | 0.007 | 0.007 | 0.010 | 0.009 | 0.012 | 0.010 | 0.012 | 0.008 | 0.012 | 0.011 | 0.014 | 0.015 | 0.011 | 0.015 | 0.017 |
| meta2298 |  | 864.619 | 126.776 | 0.045 | 0.073 | 0.056 | 0.084 | 0.126 | 0.035 | 0.079 | 0.057 | 0.045 | 0.042 | 0.075 | 0.076 | 0.066 | 0.042 | 0.043 |
| meta2299 |  | 865.497 | 40.912 | 0.028 | 0.025 | 0.036 | 0.054 | 0.104 | 0.039 | 0.064 | 0.035 | 0.035 | 0.043 | 0.057 | 0.061 | 0.047 | 0.051 | 0.062 |
| meta2300 |  | 865.526 | 141.136 | 0.092 | 0.101 | 0.102 | 0.097 | 0.079 | 0.024 | 0.038 | 0.077 | 0.146 | 0.035 | 0.096 | 0.104 | 0.032 | 0.031 | 0.105 |
| meta2301 |  | 866.193 | 373.710 | 0.010 | 0.011 | 0.008 | 0.011 | 0.013 | 0.008 | 0.015 | 0.010 | 0.008 | 0.007 | 0.009 | 0.008 | 0.014 | 0.007 | 0.013 |
| meta2302 |  | 867.514 | 40.457 | 0.089 | 0.110 | 0.115 | 0.249 | 0.244 | 0.205 | 0.219 | 0.175 | 0.224 | 0.230 | 0.374 | 0.309 | 0.145 | 0.247 | 0.344 |
| meta2303 |  | 868.171 | 386.966 | 0.003 | 0.003 | 0.003 | 0.002 | 0.007 | 0.001 | 0.011 | 0.001 | 0.005 | 0.002 | 0.001 | 0.002 | 0.006 | 0.002 | 0.002 |
| meta2304 |  | 868.735 | 32.530 | 0.007 | 0.005 | 0.006 | 0.001 | 0.001 | 0.000 | 0.001 | 0.002 | 0.001 | 0.002 | 0.003 | 0.005 | 0.002 | 0.000 | 0.001 |
| meta2305 |  | 870.203 | 478.414 | 0.005 | 0.004 | 0.003 | 0.003 | 0.006 | 0.004 | 0.004 | 0.004 | 0.004 | 0.005 | 0.003 | 0.005 | 0.006 | 0.003 | 0.004 |
| meta2306 |  | 870.751 | 33.392 | 0.036 | 0.025 | 0.026 | 0.010 | 0.009 | 0.005 | 0.007 | 0.010 | 0.013 | 0.013 | 0.015 | 0.038 | 0.013 | 0.006 | 0.014 |
| meta2307 |  | 871.217 | 429.690 | 0.004 | 0.003 | 0.002 | 0.004 | 0.002 | 0.003 | 0.002 | 0.003 | 0.002 | 0.003 | 0.003 | 0.004 | 0.002 | 0.003 | 0.002 |
| meta2308 |  | 871.671 | 355.584 | 0.004 | 0.005 | 0.006 | 0.002 | 0.005 | 0.005 | 0.005 | 0.005 | 0.004 | 0.006 | 0.006 | 0.006 | 0.008 | 0.002 | 0.005 |
| meta2309 |  | 872.767 | 33.382 | 0.119 | 0.093 | 0.093 | 0.005 | 0.004 | 0.004 | 0.004 | 0.034 | 0.013 | 0.040 | 0.057 | 0.179 | 0.032 | 0.005 | 0.013 |
| meta2310 |  | 874.072 | 164.453 | 0.005 | 0.006 | 0.009 | 0.004 | 0.008 | 0.007 | 0.005 | 0.002 | 0.011 | 0.002 | 0.006 | 0.005 | 0.009 | 0.007 | 0.004 |
| meta2311 |  | 874.542 | 177.692 | 0.009 | 0.007 | 0.011 | 0.007 | 0.004 | 0.003 | 0.004 | 0.008 | 0.006 | 0.009 | 0.012 | 0.014 | 0.006 | 0.006 | 0.007 |
| meta2312 |  | 874.589 | 287.936 | 0.010 | 0.010 | 0.011 | 0.011 | 0.012 | 0.009 | 0.011 | 0.010 | 0.013 | 0.011 | 0.013 | 0.011 | 0.014 | 0.013 | 0.013 |
| meta2313 |  | 876.107 | 275.257 | 0.119 | 0.100 | 0.087 | 0.094 | 0.127 | 0.089 | 0.074 | 0.161 | 0.103 | 0.170 | 0.093 | 0.045 | 0.102 | 0.105 | 0.107 |
| meta2314 |  | 876.558 | 176.459 | 0.271 | 0.328 | 0.311 | 0.172 | 0.093 | 0.083 | 0.130 | 0.304 | 0.193 | 0.243 | 0.358 | 0.321 | 0.178 | 0.108 | 0.119 |
| meta2315 |  | 876.568 | 288.606 | 0.041 | 0.042 | 0.047 | 0.046 | 0.045 | 0.045 | 0.044 | 0.045 | 0.060 | 0.049 | 0.048 | 0.045 | 0.065 | 0.056 | 0.049 |
| meta2316 |  | 877.535 | 44.721 | 0.018 | 0.025 | 0.026 | 0.089 | 0.050 | 0.029 | 0.015 | 0.006 | 0.014 | 0.005 | 0.008 | 0.012 | 0.006 | 0.007 | 0.006 |
| meta2317 |  | 877.725 | 32.934 | 0.006 | 0.005 | 0.005 | 0.001 | 0.001 | 0.000 | 0.000 | 0.001 | 0.001 | 0.002 | 0.002 | 0.010 | 0.002 | 0.000 | 0.001 |
| meta2318 |  | 879.234 | 335.163 | 0.009 | 0.007 | 0.008 | 0.009 | 0.010 | 0.007 | 0.001 | 0.020 | 0.004 | 0.014 | 0.008 | 0.001 | 0.006 | 0.007 | 0.008 |
| meta2319 |  | 879.309 | 363.604 | 0.072 | 0.070 | 0.084 | 0.074 | 0.094 | 0.081 | 0.078 | 0.056 | 0.049 | 0.056 | 0.087 | 0.072 | 0.050 | 0.054 | 0.043 |
| meta2320 |  | 879.816 | 31.313 | 0.008 | 0.011 | 0.009 | 0.001 | 0.001 | 0.000 | 0.000 | 0.008 | 0.001 | 0.004 | 0.006 | 0.016 | 0.003 | 0.001 | 0.002 |
| meta2321 |  | 880.586 | 178.978 | 0.034 | 0.036 | 0.036 | 0.009 | 0.006 | 0.004 | 0.007 | 0.048 | 0.015 | 0.019 | 0.033 | 0.044 | 0.020 | 0.005 | 0.008 |
| meta2322 |  | 881.475 | 40.477 | 0.006 | 0.007 | 0.008 | 0.016 | 0.010 | 0.011 | 0.012 | 0.010 | 0.007 | 0.009 | 0.018 | 0.007 | 0.008 | 0.007 | 0.016 |
| meta2323 |  | 881.512 | 177.072 | 0.024 | 0.035 | 0.023 | 0.014 | 0.011 | 0.008 | 0.009 | 0.033 | 0.019 | 0.019 | 0.034 | 0.023 | 0.014 | 0.008 | 0.014 |
| meta2324 |  | 883.207 | 372.221 | 0.007 | 0.005 | 0.004 | 0.005 | 0.006 | 0.003 | 0.009 | 0.007 | 0.002 | 0.006 | 0.010 | 0.003 | 0.003 | 0.002 | 0.006 |
| meta2325 |  | 884.542 | 40.261 | 0.324 | 0.387 | 0.404 | 1.090 | 1.070 | 0.791 | 0.888 | 0.664 | 0.848 | 0.914 | 1.550 | 1.402 | 0.570 | 0.945 | 1.514 |
| meta2326 |  | 884.791 | 318.447 | 0.023 | 0.023 | 0.027 | 0.022 | 0.023 | 0.026 | 0.023 | 0.021 | 0.029 | 0.022 | 0.024 | 0.021 | 0.027 | 0.032 | 0.022 |
| meta2327 |  | 885.544 | 174.638 | 0.024 | 0.026 | 0.025 | 0.009 | 0.007 | 0.005 | 0.007 | 0.027 | 0.014 | 0.017 | 0.032 | 0.038 | 0.022 | 0.010 | 0.017 |
| meta2328 |  | 887.562 | 175.210 | 0.244 | 0.273 | 0.250 | 0.081 | 0.053 | 0.040 | 0.051 | 0.203 | 0.083 | 0.094 | 0.254 | 0.260 | 0.122 | 0.038 | 0.083 |
| meta2329 |  | 888.476 | 376.438 | 0.011 | 0.010 | 0.011 | 0.012 | 0.011 | 0.012 | 0.008 | 0.010 | 0.007 | 0.010 | 0.014 | 0.014 | 0.009 | 0.011 | 0.010 |
| meta2330 |  | 889.497 | 40.379 | 0.082 | 0.094 | 0.110 | 0.167 | 0.156 | 0.157 | 0.145 | 0.118 | 0.182 | 0.171 | 0.237 | 0.211 | 0.118 | 0.181 | 0.250 |
| meta2331 |  | 890.137 | 384.457 | 0.009 | 0.008 | 0.007 | 0.007 | 0.012 | 0.008 | 0.011 | 0.011 | 0.010 | 0.010 | 0.010 | 0.010 | 0.011 | 0.007 | 0.011 |
| meta2332 |  | 892.288 | 484.277 | 0.013 | 0.011 | 0.013 | 0.012 | 0.011 | 0.010 | 0.012 | 0.014 | 0.013 | 0.016 | 0.012 | 0.011 | 0.011 | 0.009 | 0.014 |
| meta2333 |  | 892.533 | 42.045 | 0.065 | 0.081 | 0.080 | 0.485 | 0.248 | 0.154 | 0.071 | 0.038 | 0.086 | 0.030 | 0.037 | 0.048 | 0.031 | 0.035 | 0.038 |
| meta2334 |  | 892.540 | 44.932 | 0.057 | 0.073 | 0.070 | 0.464 | 0.207 | 0.135 | 0.044 | 0.015 | 0.062 | 0.005 | 0.028 | 0.029 | 0.015 | 0.014 | 0.019 |
| meta2335 |  | 893.134 | 275.213 | 0.032 | 0.031 | 0.027 | 0.031 | 0.039 | 0.030 | 0.023 | 0.046 | 0.029 | 0.057 | 0.027 | 0.014 | 0.029 | 0.036 | 0.028 |
| meta2336 |  | 894.752 | 32.934 | 0.012 | 0.007 | 0.009 | 0.004 | 0.002 | 0.001 | 0.001 | 0.003 | 0.004 | 0.003 | 0.005 | 0.012 | 0.004 | 0.001 | 0.004 |
| meta2337 |  | 896.166 | 460.450 | 0.002 | 0.002 | 0.003 | 0.001 | 0.002 | 0.002 | 0.003 | 0.002 | 0.003 | 0.002 | 0.002 | 0.004 | 0.009 | 0.004 | 0.003 |
| meta2338 |  | 896.550 | 289.316 | 0.003 | 0.005 | 0.003 | 0.004 | 0.004 | 0.004 | 0.004 | 0.003 | 0.005 | 0.004 | 0.004 | 0.004 | 0.008 | 0.004 | 0.005 |
| meta2339 |  | 896.766 | 33.103 | 0.035 | 0.025 | 0.029 | 0.007 | 0.003 | 0.002 | 0.002 | 0.009 | 0.004 | 0.011 | 0.015 | 0.041 | 0.011 | 0.002 | 0.005 |
| meta2340 |  | 898.269 | 361.896 | 0.043 | 0.042 | 0.037 | 0.045 | 0.039 | 0.025 | 0.042 | 0.027 | 0.016 | 0.039 | 0.048 | 0.028 | 0.019 | 0.010 | 0.038 |
| meta2341 |  | 900.536 | 41.557 | 0.005 | 0.007 | 0.006 | 0.007 | 0.009 | 0.006 | 0.008 | 0.005 | 0.005 | 0.008 | 0.012 | 0.009 | 0.006 | 0.008 | 0.009 |
| meta2342 |  | 900.557 | 175.212 | 0.030 | 0.026 | 0.030 | 0.014 | 0.016 | 0.015 | 0.011 | 0.042 | 0.035 | 0.035 | 0.055 | 0.030 | 0.011 | 0.011 | 0.015 |
| meta2343 |  | 902.573 | 174.500 | 0.167 | 0.159 | 0.185 | 0.070 | 0.049 | 0.033 | 0.039 | 0.147 | 0.076 | 0.098 | 0.157 | 0.198 | 0.127 | 0.074 | 0.131 |
| meta2344 |  | 902.571 | 47.305 | 0.009 | 0.010 | 0.009 | 0.005 | 0.004 | 0.002 | 0.006 | 0.009 | 0.007 | 0.005 | 0.012 | 0.012 | 0.005 | 0.004 | 0.005 |
| meta2345 |  | 904.588 | 175.212 | 1.376 | 1.394 | 1.387 | 0.448 | 0.328 | 0.203 | 0.314 | 1.033 | 0.480 | 0.544 | 1.330 | 1.552 | 0.612 | 0.262 | 0.477 |
| meta2346 |  | 905.150 | 434.213 | 0.006 | 0.007 | 0.006 | 0.004 | 0.006 | 0.005 | 0.004 | 0.005 | 0.010 | 0.014 | 0.008 | 0.007 | 0.008 | 0.005 | 0.009 |
| meta2347 |  | 905.186 | 372.221 | 0.008 | 0.006 | 0.005 | 0.006 | 0.007 | 0.004 | 0.011 | 0.008 | 0.002 | 0.009 | 0.014 | 0.004 | 0.003 | 0.002 | 0.009 |
| meta2348 |  | 905.472 | 40.196 | 0.022 | 0.021 | 0.025 | 0.042 | 0.035 | 0.037 | 0.035 | 0.032 | 0.044 | 0.039 | 0.054 | 0.052 | 0.030 | 0.045 | 0.053 |
| meta2349 |  | 905.754 | 33.753 | 0.003 | 0.003 | 0.003 | 0.008 | 0.005 | 0.003 | 0.003 | 0.002 | 0.003 | 0.001 | 0.002 | 0.005 | 0.004 | 0.001 | 0.002 |
| meta2350 |  | 905.834 | 31.749 | 0.007 | 0.008 | 0.007 | 0.001 | 0.001 | 0.001 | 0.001 | 0.005 | 0.001 | 0.003 | 0.004 | 0.013 | 0.003 | 0.000 | 0.001 |
| meta2351 |  | 906.774 | 318.444 | 0.016 | 0.016 | 0.017 | 0.014 | 0.015 | 0.017 | 0.016 | 0.013 | 0.019 | 0.014 | 0.017 | 0.014 | 0.017 | 0.020 | 0.015 |
| meta2352 |  | 907.173 | 335.389 | 0.015 | 0.010 | 0.011 | 0.012 | 0.014 | 0.007 | 0.006 | 0.023 | 0.004 | 0.018 | 0.009 | 0.004 | 0.010 | 0.007 | 0.012 |
| meta2353 |  | 907.341 | 28.501 | 0.003 | 0.004 | 0.005 | 0.006 | 0.008 | 0.009 | 0.009 | 0.007 | 0.008 | 0.007 | 0.007 | 0.005 | 0.007 | 0.005 | 0.007 |
| meta2354 |  | 908.284 | 477.194 | 0.003 | 0.005 | 0.002 | 0.003 | 0.004 | 0.003 | 0.003 | 0.004 | 0.003 | 0.005 | 0.004 | 0.003 | 0.004 | 0.003 | 0.003 |
| meta2355 |  | 908.571 | 289.276 | 0.003 | 0.003 | 0.004 | 0.004 | 0.004 | 0.005 | 0.005 | 0.003 | 0.005 | 0.004 | 0.004 | 0.004 | 0.007 | 0.004 | 0.006 |
| meta2356 |  | 909.542 | 175.212 | 0.092 | 0.092 | 0.101 | 0.028 | 0.022 | 0.013 | 0.017 | 0.087 | 0.043 | 0.041 | 0.091 | 0.098 | 0.037 | 0.015 | 0.037 |
| meta2357 |  | 911.334 | 477.449 | 0.005 | 0.005 | 0.004 | 0.005 | 0.005 | 0.004 | 0.006 | 0.004 | 0.005 | 0.004 | 0.006 | 0.005 | 0.008 | 0.005 | 0.006 |
| meta2358 |  | 911.947 | 468.515 | 0.006 | 0.005 | 0.006 | 0.007 | 0.005 | 0.009 | 0.006 | 0.003 | 0.003 | 0.007 | 0.003 | 0.007 | 0.005 | 0.005 | 0.007 |
| meta2359 |  | 912.200 | 419.287 | 0.013 | 0.014 | 0.015 | 0.012 | 0.012 | 0.014 | 0.015 | 0.022 | 0.023 | 0.019 | 0.016 | 0.015 | 0.017 | 0.017 | 0.020 |
| meta2360 |  | 912.546 | 288.152 | 0.006 | 0.006 | 0.007 | 0.007 | 0.007 | 0.006 | 0.009 | 0.006 | 0.008 | 0.008 | 0.007 | 0.007 | 0.010 | 0.007 | 0.007 |
| meta2361 |  | 914.056 | 275.897 | 0.013 | 0.014 | 0.012 | 0.013 | 0.014 | 0.009 | 0.016 | 0.018 | 0.015 | 0.013 | 0.014 | 0.012 | 0.014 | 0.011 | 0.014 |
| meta2362 |  | 914.525 | 288.626 | 0.014 | 0.014 | 0.016 | 0.015 | 0.016 | 0.017 | 0.015 | 0.015 | 0.023 | 0.016 | 0.016 | 0.015 | 0.021 | 0.020 | 0.017 |
| meta2363 |  | 914.584 | 39.777 | 0.009 | 0.009 | 0.013 | 0.018 | 0.012 | 0.025 | 0.015 | 0.011 | 0.016 | 0.014 | 0.013 | 0.011 | 0.008 | 0.016 | 0.014 |
| meta2364 |  | 916.182 | 434.758 | 0.007 | 0.007 | 0.008 | 0.006 | 0.007 | 0.004 | 0.007 | 0.007 | 0.007 | 0.007 | 0.009 | 0.007 | 0.008 | 0.006 | 0.007 |
| meta2365 |  | 916.530 | 42.055 | 0.005 | 0.007 | 0.007 | 0.012 | 0.020 | 0.011 | 0.023 | 0.013 | 0.012 | 0.011 | 0.028 | 0.029 | 0.011 | 0.017 | 0.026 |
| meta2366 |  | 918.750 | 32.667 | 0.003 | 0.003 | 0.003 | 0.002 | 0.001 | 0.000 | 0.001 | 0.007 | 0.002 | 0.004 | 0.002 | 0.003 | 0.001 | 0.001 | 0.002 |
| meta2367 |  | 920.558 | 45.544 | 0.024 | 0.029 | 0.028 | 0.106 | 0.063 | 0.035 | 0.016 | 0.007 | 0.016 | 0.004 | 0.005 | 0.011 | 0.004 | 0.005 | 0.006 |
| meta2368 |  | 920.767 | 32.979 | 0.024 | 0.017 | 0.016 | 0.005 | 0.001 | 0.000 | 0.001 | 0.006 | 0.005 | 0.007 | 0.014 | 0.023 | 0.009 | 0.000 | 0.002 |
| meta2369 |  | 922.141 | 335.498 | 0.010 | 0.009 | 0.006 | 0.012 | 0.011 | 0.007 | 0.003 | 0.020 | 0.003 | 0.017 | 0.011 | 0.003 | 0.010 | 0.007 | 0.007 |
| meta2370 |  | 924.536 | 40.261 | 0.004 | 0.004 | 0.005 | 0.015 | 0.012 | 0.008 | 0.011 | 0.009 | 0.010 | 0.009 | 0.019 | 0.014 | 0.006 | 0.010 | 0.014 |
| meta2371 |  | 925.516 | 175.214 | 0.025 | 0.024 | 0.025 | 0.006 | 0.012 | 0.004 | 0.004 | 0.022 | 0.015 | 0.012 | 0.034 | 0.026 | 0.008 | 0.003 | 0.017 |
| meta2372 |  | 926.383 | 249.257 | 0.008 | 0.007 | 0.006 | 0.008 | 0.016 | 0.005 | 0.007 | 0.007 | 0.003 | 0.005 | 0.004 | 0.004 | 0.004 | 0.006 | 0.005 |
| meta2373 |  | 926.573 | 161.150 | 0.273 | 0.267 | 0.290 | 0.090 | 0.071 | 0.056 | 0.059 | 0.198 | 0.055 | 0.079 | 0.203 | 0.277 | 0.092 | 0.057 | 0.067 |
| meta2374 |  | 926.782 | 318.444 | 0.006 | 0.006 | 0.007 | 0.005 | 0.006 | 0.007 | 0.006 | 0.005 | 0.008 | 0.006 | 0.006 | 0.005 | 0.007 | 0.009 | 0.006 |
| meta2375 |  | 927.131 | 434.587 | 0.006 | 0.006 | 0.006 | 0.003 | 0.005 | 0.004 | 0.005 | 0.006 | 0.010 | 0.009 | 0.007 | 0.006 | 0.008 | 0.005 | 0.008 |
| meta2376 |  | 927.166 | 372.221 | 0.008 | 0.010 | 0.007 | 0.009 | 0.008 | 0.004 | 0.011 | 0.009 | 0.003 | 0.008 | 0.014 | 0.005 | 0.004 | 0.003 | 0.011 |
| meta2377 |  | 927.454 | 39.248 | 0.002 | 0.003 | 0.003 | 0.003 | 0.003 | 0.004 | 0.004 | 0.002 | 0.004 | 0.003 | 0.005 | 0.005 | 0.003 | 0.005 | 0.004 |
| meta2378 |  | 928.152 | 334.963 | 0.042 | 0.033 | 0.026 | 0.031 | 0.036 | 0.027 | 0.015 | 0.048 | 0.014 | 0.040 | 0.029 | 0.014 | 0.025 | 0.019 | 0.032 |
| meta2379 |  | 928.207 | 434.436 | 0.006 | 0.007 | 0.008 | 0.008 | 0.007 | 0.005 | 0.009 | 0.009 | 0.011 | 0.008 | 0.007 | 0.005 | 0.006 | 0.006 | 0.007 |
| meta2380 |  | 930.849 | 31.453 | 0.004 | 0.005 | 0.005 | 0.001 | 0.001 | 0.000 | 0.001 | 0.003 | 0.001 | 0.003 | 0.003 | 0.009 | 0.002 | 0.001 | 0.001 |
| meta2381 |  | 931.606 | 161.445 | 0.059 | 0.051 | 0.062 | 0.009 | 0.006 | 0.003 | 0.005 | 0.042 | 0.006 | 0.010 | 0.048 | 0.062 | 0.012 | 0.004 | 0.008 |
| meta2382 |  | 932.564 | 44.714 | 0.004 | 0.004 | 0.005 | 0.002 | 0.004 | 0.002 | 0.002 | 0.003 | 0.003 | 0.003 | 0.004 | 0.005 | 0.001 | 0.003 | 0.003 |
| meta2383 |  | 933.315 | 477.407 | 0.005 | 0.004 | 0.003 | 0.004 | 0.005 | 0.003 | 0.006 | 0.005 | 0.006 | 0.008 | 0.005 | 0.006 | 0.008 | 0.004 | 0.007 |
| meta2384 |  | 936.566 | 44.714 | 0.034 | 0.040 | 0.041 | 0.302 | 0.142 | 0.082 | 0.024 | 0.005 | 0.039 | 0.004 | 0.005 | 0.011 | 0.006 | 0.006 | 0.005 |
| meta2385 |  | 937.262 | 482.842 | 0.026 | 0.021 | 0.020 | 0.021 | 0.033 | 0.019 | 0.033 | 0.037 | 0.028 | 0.019 | 0.024 | 0.029 | 0.028 | 0.021 | 0.024 |
| meta2386 |  | 938.566 | 288.556 | 0.008 | 0.009 | 0.010 | 0.010 | 0.010 | 0.010 | 0.009 | 0.009 | 0.011 | 0.010 | 0.010 | 0.009 | 0.013 | 0.011 | 0.011 |
| meta2387 |  | 940.350 | 361.870 | 0.014 | 0.013 | 0.014 | 0.012 | 0.017 | 0.013 | 0.012 | 0.022 | 0.012 | 0.012 | 0.017 | 0.012 | 0.016 | 0.011 | 0.014 |
| meta2388 |  | 942.201 | 404.096 | 0.004 | 0.005 | 0.004 | 0.004 | 0.006 | 0.006 | 0.005 | 0.004 | 0.003 | 0.003 | 0.004 | 0.005 | 0.003 | 0.005 | 0.004 |
| meta2389 |  | 942.544 | 209.232 | 0.004 | 0.006 | 0.004 | 0.005 | 0.004 | 0.004 | 0.004 | 0.005 | 0.004 | 0.005 | 0.007 | 0.004 | 0.002 | 0.003 | 0.005 |
| meta2390 |  | 942.634 | 46.556 | 0.004 | 0.003 | 0.004 | 0.012 | 0.009 | 0.005 | 0.004 | 0.002 | 0.003 | 0.002 | 0.002 | 0.003 | 0.002 | 0.001 | 0.002 |
| meta2391 |  | 943.139 | 372.245 | 0.004 | 0.004 | 0.003 | 0.004 | 0.005 | 0.002 | 0.006 | 0.005 | 0.002 | 0.005 | 0.008 | 0.002 | 0.003 | 0.002 | 0.006 |
| meta2392 |  | 944.123 | 335.397 | 0.013 | 0.011 | 0.011 | 0.011 | 0.010 | 0.008 | 0.006 | 0.017 | 0.007 | 0.014 | 0.008 | 0.005 | 0.012 | 0.008 | 0.016 |
| meta2393 |  | 944.768 | 33.453 | 0.005 | 0.003 | 0.004 | 0.002 | 0.002 | 0.001 | 0.002 | 0.003 | 0.003 | 0.003 | 0.003 | 0.008 | 0.004 | 0.002 | 0.001 |
| meta2394 |  | 944.852 | 29.572 | 0.009 | 0.015 | 0.012 | 0.001 | 0.002 | 0.000 | 0.001 | 0.004 | 0.002 | 0.002 | 0.011 | 0.021 | 0.005 | 0.001 | 0.002 |
| meta2395 |  | 945.201 | 393.139 | 0.005 | 0.009 | 0.005 | 0.005 | 0.006 | 0.006 | 0.007 | 0.006 | 0.004 | 0.004 | 0.006 | 0.006 | 0.004 | 0.005 | 0.006 |
| meta2396 |  | 946.350 | 486.047 | 0.002 | 0.002 | 0.003 | 0.001 | 0.002 | 0.001 | 0.001 | 0.001 | 0.002 | 0.001 | 0.001 | 0.002 | 0.003 | 0.002 | 0.002 |
| meta2397 |  | 948.201 | 404.027 | 0.006 | 0.006 | 0.006 | 0.008 | 0.006 | 0.007 | 0.007 | 0.006 | 0.004 | 0.006 | 0.005 | 0.007 | 0.005 | 0.005 | 0.006 |
| meta2398 |  | 948.554 | 159.430 | 0.020 | 0.014 | 0.018 | 0.005 | 0.006 | 0.001 | 0.003 | 0.010 | 0.003 | 0.004 | 0.011 | 0.022 | 0.004 | 0.002 | 0.004 |
| meta2399 |  | 948.576 | 47.634 | 0.004 | 0.004 | 0.004 | 0.007 | 0.005 | 0.003 | 0.004 | 0.003 | 0.003 | 0.002 | 0.002 | 0.002 | 0.002 | 0.001 | 0.002 |
| meta2400 |  | 948.882 | 29.465 | 0.008 | 0.013 | 0.011 | 0.001 | 0.001 | 0.000 | 0.000 | 0.005 | 0.002 | 0.002 | 0.009 | 0.020 | 0.004 | 0.000 | 0.002 |
| meta2401 |  | 949.148 | 372.221 | 0.009 | 0.008 | 0.006 | 0.008 | 0.008 | 0.004 | 0.010 | 0.008 | 0.005 | 0.009 | 0.011 | 0.005 | 0.005 | 0.004 | 0.011 |
| meta2402 |  | 950.135 | 335.376 | 0.023 | 0.027 | 0.023 | 0.026 | 0.028 | 0.015 | 0.021 | 0.024 | 0.018 | 0.029 | 0.024 | 0.011 | 0.020 | 0.025 | 0.024 |
| meta2403 |  | 950.383 | 248.797 | 0.013 | 0.012 | 0.012 | 0.016 | 0.017 | 0.010 | 0.009 | 0.012 | 0.003 | 0.014 | 0.006 | 0.013 | 0.007 | 0.009 | 0.009 |
| meta2404 |  | 952.575 | 40.571 | 0.014 | 0.017 | 0.020 | 0.181 | 0.074 | 0.040 | 0.014 | 0.004 | 0.028 | 0.003 | 0.003 | 0.005 | 0.004 | 0.003 | 0.002 |
| meta2405 |  | 952.589 | 159.549 | 0.086 | 0.074 | 0.066 | 0.024 | 0.019 | 0.013 | 0.013 | 0.045 | 0.017 | 0.020 | 0.057 | 0.092 | 0.033 | 0.023 | 0.020 |
| meta2406 |  | 954.710 | 487.637 | 0.003 | 0.002 | 0.003 | 0.003 | 0.003 | 0.003 | 0.003 | 0.004 | 0.003 | 0.004 | 0.004 | 0.005 | 0.003 | 0.004 | 0.003 |
| meta2407 |  | 955.150 | 420.579 | 0.020 | 0.021 | 0.024 | 0.016 | 0.022 | 0.020 | 0.024 | 0.018 | 0.027 | 0.022 | 0.022 | 0.021 | 0.024 | 0.016 | 0.033 |
| meta2408 |  | 955.175 | 201.240 | 0.004 | 0.002 | 0.002 | 0.001 | 0.004 | 0.000 | 0.003 | 0.001 | 0.002 | 0.003 | 0.001 | 0.002 | 0.003 | 0.002 | 0.004 |
| meta2409 |  | 958.409 | 473.630 | 0.003 | 0.003 | 0.004 | 0.005 | 0.005 | 0.003 | 0.004 | 0.003 | 0.003 | 0.005 | 0.006 | 0.005 | 0.005 | 0.003 | 0.005 |
| meta2410 |  | 958.590 | 275.327 | 0.005 | 0.007 | 0.006 | 0.006 | 0.007 | 0.005 | 0.004 | 0.007 | 0.007 | 0.009 | 0.006 | 0.005 | 0.009 | 0.004 | 0.005 |
| meta2411 |  | 960.551 | 289.601 | 0.006 | 0.007 | 0.008 | 0.007 | 0.007 | 0.007 | 0.007 | 0.007 | 0.009 | 0.007 | 0.006 | 0.007 | 0.009 | 0.009 | 0.008 |
| meta2412 |  | 963.639 | 178.346 | 0.002 | 0.002 | 0.002 | 0.002 | 0.002 | 0.001 | 0.004 | 0.003 | 0.001 | 0.002 | 0.004 | 0.003 | 0.001 | 0.001 | 0.003 |
| meta2413 |  | 964.580 | 45.677 | 0.018 | 0.020 | 0.019 | 0.071 | 0.038 | 0.021 | 0.012 | 0.003 | 0.011 | 0.002 | 0.004 | 0.005 | 0.004 | 0.003 | 0.003 |
| meta2414 |  | 965.120 | 372.330 | 0.007 | 0.006 | 0.006 | 0.006 | 0.006 | 0.003 | 0.008 | 0.006 | 0.003 | 0.007 | 0.008 | 0.005 | 0.004 | 0.003 | 0.009 |
| meta2415 |  | 968.602 | 287.951 | 0.003 | 0.002 | 0.002 | 0.003 | 0.003 | 0.003 | 0.004 | 0.003 | 0.003 | 0.003 | 0.003 | 0.004 | 0.004 | 0.003 | 0.003 |
| meta2416 |  | 968.774 | 318.447 | 0.020 | 0.022 | 0.023 | 0.020 | 0.022 | 0.024 | 0.021 | 0.017 | 0.026 | 0.019 | 0.020 | 0.019 | 0.023 | 0.027 | 0.020 |
| meta2417 |  | 971.137 | 372.260 | 0.005 | 0.006 | 0.005 | 0.006 | 0.005 | 0.003 | 0.007 | 0.005 | 0.004 | 0.006 | 0.006 | 0.004 | 0.004 | 0.004 | 0.007 |
| meta2418 |  | 974.345 | 489.090 | 0.008 | 0.008 | 0.008 | 0.005 | 0.009 | 0.007 | 0.009 | 0.008 | 0.008 | 0.008 | 0.004 | 0.012 | 0.019 | 0.011 | 0.013 |
| meta2419 |  | 974.546 | 288.285 | 0.009 | 0.010 | 0.010 | 0.010 | 0.011 | 0.008 | 0.011 | 0.010 | 0.013 | 0.011 | 0.010 | 0.010 | 0.013 | 0.011 | 0.010 |
| meta2420 |  | 976.526 | 288.766 | 0.020 | 0.022 | 0.025 | 0.022 | 0.024 | 0.024 | 0.021 | 0.022 | 0.034 | 0.022 | 0.022 | 0.024 | 0.032 | 0.028 | 0.025 |
| meta2421 |  | 977.131 | 420.588 | 0.007 | 0.007 | 0.010 | 0.007 | 0.008 | 0.008 | 0.008 | 0.011 | 0.008 | 0.008 | 0.009 | 0.008 | 0.007 | 0.008 | 0.011 |
| meta2422 |  | 979.765 | 318.444 | 0.006 | 0.006 | 0.008 | 0.006 | 0.007 | 0.007 | 0.007 | 0.005 | 0.009 | 0.006 | 0.007 | 0.005 | 0.007 | 0.010 | 0.006 |
| meta2423 |  | 980.592 | 46.507 | 0.022 | 0.027 | 0.025 | 0.166 | 0.081 | 0.048 | 0.016 | 0.004 | 0.026 | 0.003 | 0.002 | 0.007 | 0.006 | 0.003 | 0.004 |
| meta2424 |  | 982.363 | 28.496 | 0.001 | 0.002 | 0.002 | 0.004 | 0.007 | 0.004 | 0.002 | 0.005 | 0.002 | 0.002 | 0.003 | 0.003 | 0.002 | 0.002 | 0.003 |
| meta2425 |  | 989.582 | 140.307 | 0.007 | 0.009 | 0.010 | 0.006 | 0.011 | 0.003 | 0.003 | 0.012 | 0.006 | 0.005 | 0.006 | 0.005 | 0.005 | 0.006 | 0.005 |
| meta2426 |  | 989.654 | 175.215 | 0.026 | 0.038 | 0.036 | 0.038 | 0.040 | 0.021 | 0.065 | 0.057 | 0.011 | 0.020 | 0.026 | 0.044 | 0.022 | 0.033 | 0.028 |
| meta2427 |  | 990.174 | 474.979 | 0.004 | 0.004 | 0.004 | 0.005 | 0.006 | 0.003 | 0.005 | 0.006 | 0.007 | 0.007 | 0.008 | 0.005 | 0.007 | 0.004 | 0.006 |
| meta2428 |  | 990.220 | 466.045 | 0.009 | 0.007 | 0.011 | 0.007 | 0.011 | 0.011 | 0.011 | 0.007 | 0.006 | 0.007 | 0.010 | 0.009 | 0.009 | 0.008 | 0.011 |
| meta2429 |  | 990.757 | 318.444 | 0.013 | 0.012 | 0.014 | 0.012 | 0.012 | 0.014 | 0.014 | 0.011 | 0.017 | 0.013 | 0.014 | 0.012 | 0.015 | 0.017 | 0.013 |
| meta2430 |  | 993.222 | 461.403 | 0.031 | 0.021 | 0.025 | 0.024 | 0.030 | 0.032 | 0.032 | 0.030 | 0.022 | 0.025 | 0.027 | 0.029 | 0.027 | 0.021 | 0.027 |
| meta2431 |  | 994.309 | 152.450 | 0.001 | 0.002 | 0.002 | 0.001 | 0.003 | 0.001 | 0.004 | 0.002 | 0.000 | 0.000 | 0.001 | 0.001 | 0.001 | 0.001 | 0.001 |
| meta2432 |  | 996.601 | 42.055 | 0.014 | 0.017 | 0.018 | 0.156 | 0.062 | 0.035 | 0.011 | 0.003 | 0.022 | 0.002 | 0.003 | 0.006 | 0.002 | 0.002 | 0.003 |
| meta2433 |  | 999.263 | 152.549 | 0.002 | 0.002 | 0.003 | 0.002 | 0.003 | 0.002 | 0.005 | 0.004 | 0.001 | 0.001 | 0.002 | 0.004 | 0.002 | 0.002 | 0.003 |
| meta2434 |  | 1001.121 | 275.034 | 0.013 | 0.009 | 0.010 | 0.008 | 0.012 | 0.008 | 0.009 | 0.016 | 0.012 | 0.015 | 0.009 | 0.005 | 0.009 | 0.011 | 0.010 |
| meta2435 |  | 1002.657 | 174.799 | 0.034 | 0.049 | 0.042 | 0.139 | 0.145 | 0.098 | 0.155 | 0.070 | 0.080 | 0.058 | 0.047 | 0.020 | 0.057 | 0.108 | 0.093 |
| meta2436 |  | 1003.159 | 174.738 | 0.054 | 0.038 | 0.054 | 0.173 | 0.181 | 0.115 | 0.182 | 0.082 | 0.083 | 0.079 | 0.054 | 0.030 | 0.059 | 0.096 | 0.117 |
| meta2437 |  | 1006.343 | 476.997 | 0.020 | 0.018 | 0.018 | 0.016 | 0.020 | 0.025 | 0.022 | 0.025 | 0.017 | 0.017 | 0.019 | 0.023 | 0.020 | 0.014 | 0.013 |
| meta2438 |  | 1008.358 | 494.105 | 0.015 | 0.012 | 0.014 | 0.011 | 0.013 | 0.019 | 0.014 | 0.018 | 0.012 | 0.015 | 0.015 | 0.016 | 0.014 | 0.010 | 0.008 |
| meta2439 |  | 1010.156 | 465.347 | 0.109 | 0.102 | 0.096 | 0.093 | 0.118 | 0.076 | 0.134 | 0.106 | 0.101 | 0.095 | 0.106 | 0.099 | 0.125 | 0.098 | 0.132 |
| meta2440 |  | 1010.270 | 371.889 | 0.003 | 0.003 | 0.002 | 0.003 | 0.003 | 0.002 | 0.004 | 0.004 | 0.001 | 0.002 | 0.004 | 0.002 | 0.001 | 0.001 | 0.001 |
| meta2441 |  | 1010.613 | 47.634 | 0.003 | 0.003 | 0.003 | 0.010 | 0.006 | 0.003 | 0.002 | 0.001 | 0.002 | 0.001 | 0.001 | 0.002 | 0.001 | 0.001 | 0.001 |
| meta2442 |  | 1011.298 | 477.074 | 0.005 | 0.005 | 0.005 | 0.005 | 0.007 | 0.005 | 0.004 | 0.007 | 0.006 | 0.007 | 0.006 | 0.008 | 0.007 | 0.004 | 0.005 |
| meta2443 |  | 1013.234 | 461.940 | 0.005 | 0.005 | 0.005 | 0.005 | 0.006 | 0.005 | 0.007 | 0.003 | 0.005 | 0.004 | 0.005 | 0.006 | 0.005 | 0.004 | 0.004 |
| meta2444 |  | 1013.315 | 494.203 | 0.006 | 0.005 | 0.005 | 0.004 | 0.005 | 0.007 | 0.006 | 0.006 | 0.005 | 0.006 | 0.006 | 0.006 | 0.006 | 0.004 | 0.004 |
| meta2445 |  | 1013.573 | 183.933 | 0.005 | 0.004 | 0.006 | 0.001 | 0.001 | 0.009 | 0.018 | 0.003 | 0.000 | 0.008 | 0.003 | 0.001 | 0.007 | 0.011 | 0.001 |
| meta2446 |  | 1014.484 | 289.104 | 0.006 | 0.006 | 0.008 | 0.007 | 0.008 | 0.007 | 0.007 | 0.007 | 0.010 | 0.008 | 0.007 | 0.008 | 0.010 | 0.008 | 0.008 |
| meta2447 |  | 1015.231 | 152.520 | 0.005 | 0.006 | 0.008 | 0.007 | 0.010 | 0.007 | 0.012 | 0.007 | 0.004 | 0.003 | 0.004 | 0.007 | 0.005 | 0.005 | 0.006 |
| meta2448 |  | 1018.149 | 275.257 | 0.098 | 0.083 | 0.070 | 0.078 | 0.110 | 0.074 | 0.067 | 0.128 | 0.074 | 0.136 | 0.073 | 0.038 | 0.084 | 0.087 | 0.084 |
| meta2449 |  | 1019.372 | 361.901 | 0.009 | 0.011 | 0.008 | 0.011 | 0.011 | 0.004 | 0.020 | 0.006 | 0.004 | 0.010 | 0.014 | 0.010 | 0.003 | 0.004 | 0.013 |
| meta2450 |  | 1021.588 | 41.659 | 0.002 | 0.003 | 0.002 | 0.004 | 0.004 | 0.003 | 0.005 | 0.004 | 0.003 | 0.004 | 0.005 | 0.005 | 0.004 | 0.005 | 0.005 |
| meta2451 |  | 1023.105 | 275.367 | 0.037 | 0.043 | 0.040 | 0.034 | 0.045 | 0.040 | 0.036 | 0.048 | 0.037 | 0.049 | 0.037 | 0.024 | 0.039 | 0.040 | 0.038 |
| meta2452 |  | 1024.618 | 151.965 | 0.011 | 0.011 | 0.011 | 0.006 | 0.006 | 0.004 | 0.007 | 0.009 | 0.005 | 0.004 | 0.009 | 0.013 | 0.006 | 0.004 | 0.004 |
| meta2453 |  | 1024.617 | 46.170 | 0.014 | 0.016 | 0.018 | 0.100 | 0.054 | 0.030 | 0.010 | 0.003 | 0.014 | 0.003 | 0.004 | 0.006 | 0.003 | 0.003 | 0.003 |
| meta2454 |  | 1025.168 | 459.785 | 0.119 | 0.120 | 0.121 | 0.101 | 0.134 | 0.098 | 0.149 | 0.122 | 0.122 | 0.104 | 0.112 | 0.121 | 0.159 | 0.127 | 0.149 |
| meta2455 |  | 1025.654 | 170.980 | 0.010 | 0.009 | 0.010 | 0.013 | 0.019 | 0.014 | 0.042 | 0.023 | 0.011 | 0.011 | 0.024 | 0.017 | 0.014 | 0.013 | 0.018 |
| meta2456 |  | 1026.368 | 427.999 | 0.013 | 0.009 | 0.012 | 0.007 | 0.009 | 0.026 | 0.011 | 0.014 | 0.005 | 0.009 | 0.012 | 0.021 | 0.010 | 0.005 | 0.003 |
| meta2457 |  | 1027.293 | 344.793 | 0.005 | 0.007 | 0.006 | 0.006 | 0.008 | 0.017 | 0.006 | 0.008 | 0.003 | 0.005 | 0.008 | 0.013 | 0.005 | 0.005 | 0.001 |
| meta2458 |  | 1028.167 | 465.345 | 0.125 | 0.131 | 0.135 | 0.114 | 0.161 | 0.112 | 0.162 | 0.152 | 0.130 | 0.102 | 0.120 | 0.138 | 0.164 | 0.129 | 0.174 |
| meta2459 |  | 1029.571 | 163.411 | 0.011 | 0.010 | 0.007 | 0.000 | 0.001 | 0.006 | 0.033 | 0.011 | 0.002 | 0.018 | 0.012 | 0.010 | 0.016 | 0.025 | 0.004 |
| meta2460 |  | 1030.774 | 318.447 | 0.006 | 0.005 | 0.007 | 0.005 | 0.006 | 0.006 | 0.006 | 0.005 | 0.008 | 0.006 | 0.005 | 0.005 | 0.007 | 0.008 | 0.005 |
| meta2461 |  | 1031.326 | 427.990 | 0.045 | 0.035 | 0.041 | 0.032 | 0.034 | 0.075 | 0.037 | 0.042 | 0.023 | 0.036 | 0.044 | 0.059 | 0.031 | 0.019 | 0.013 |
| meta2462 |  | 1031.588 | 163.011 | 0.148 | 0.175 | 0.174 | 0.004 | 0.013 | 0.188 | 0.278 | 0.451 | 0.031 | 0.656 | 0.140 | 0.084 | 0.370 | 0.608 | 0.112 |
| meta2463 | Oleoyl-CoA | 1032.363 | 327.914 | 0.012 | 0.014 | 0.014 | 0.013 | 0.028 | 0.010 | 0.011 | 0.019 | 0.007 | 0.020 | 0.008 | 0.023 | 0.016 | 0.014 | 0.028 |
| meta2464 |  | 1033.083 | 164.387 | 0.007 | 0.006 | 0.010 | 0.004 | 0.008 | 0.008 | 0.006 | 0.003 | 0.012 | 0.004 | 0.006 | 0.005 | 0.008 | 0.006 | 0.006 |
| meta2465 |  | 1038.526 | 288.786 | 0.005 | 0.006 | 0.006 | 0.006 | 0.007 | 0.006 | 0.005 | 0.006 | 0.009 | 0.007 | 0.008 | 0.007 | 0.010 | 0.007 | 0.007 |
| meta2466 |  | 1039.075 | 275.327 | 0.007 | 0.006 | 0.006 | 0.005 | 0.007 | 0.004 | 0.006 | 0.007 | 0.005 | 0.006 | 0.005 | 0.005 | 0.007 | 0.005 | 0.006 |
| meta2467 |  | 1039.667 | 172.879 | 0.026 | 0.046 | 0.045 | 0.046 | 0.044 | 0.042 | 0.053 | 0.096 | 0.055 | 0.042 | 0.059 | 0.076 | 0.054 | 0.051 | 0.065 |
| meta2468 |  | 1040.524 | 288.804 | 0.004 | 0.004 | 0.006 | 0.005 | 0.006 | 0.006 | 0.006 | 0.005 | 0.008 | 0.006 | 0.006 | 0.005 | 0.008 | 0.007 | 0.005 |
| meta2469 |  | 1040.625 | 43.392 | 0.010 | 0.010 | 0.011 | 0.091 | 0.048 | 0.024 | 0.008 | 0.002 | 0.016 | 0.002 | 0.002 | 0.004 | 0.003 | 0.003 | 0.002 |
| meta2470 |  | 1042.484 | 42.055 | 0.003 | 0.004 | 0.004 | 0.005 | 0.005 | 0.004 | 0.006 | 0.004 | 0.005 | 0.004 | 0.005 | 0.005 | 0.006 | 0.005 | 0.005 |
| meta2471 |  | 1042.633 | 43.962 | 0.002 | 0.003 | 0.003 | 0.024 | 0.009 | 0.007 | 0.002 | 0.001 | 0.004 | 0.001 | 0.001 | 0.001 | 0.001 | 0.001 | 0.001 |
| meta2472 |  | 1043.924 | 476.976 | 0.001 | 0.001 | 0.001 | 0.001 | 0.001 | 0.003 | 0.001 | 0.001 | 0.001 | 0.002 | 0.002 | 0.003 | 0.002 | 0.002 | 0.001 |
| meta2473 |  | 1044.162 | 479.421 | 0.036 | 0.034 | 0.036 | 0.036 | 0.044 | 0.028 | 0.043 | 0.031 | 0.041 | 0.031 | 0.041 | 0.040 | 0.052 | 0.039 | 0.052 |
| meta2474 |  | 1045.623 | 169.584 | 0.003 | 0.004 | 0.004 | 0.009 | 0.007 | 0.007 | 0.020 | 0.009 | 0.004 | 0.006 | 0.009 | 0.008 | 0.007 | 0.011 | 0.009 |
| meta2475 |  | 1045.717 | 172.097 | 0.037 | 0.064 | 0.064 | 0.062 | 0.066 | 0.035 | 0.152 | 0.109 | 0.037 | 0.045 | 0.092 | 0.091 | 0.053 | 0.046 | 0.140 |
| meta2476 |  | 1046.599 | 163.743 | 0.006 | 0.007 | 0.007 | 0.000 | 0.000 | 0.004 | 0.024 | 0.008 | 0.001 | 0.014 | 0.009 | 0.008 | 0.013 | 0.016 | 0.003 |
| meta2477 |  | 1047.148 | 459.788 | 0.009 | 0.010 | 0.009 | 0.009 | 0.010 | 0.008 | 0.013 | 0.008 | 0.008 | 0.009 | 0.008 | 0.010 | 0.012 | 0.011 | 0.012 |
| meta2478 | 1-Stearoyl-2-hydroxy-sn-glycero-3-phosphocholine | 1047.733 | 171.835 | 0.092 | 0.152 | 0.137 | 0.132 | 0.150 | 0.079 | 0.361 | 0.295 | 0.106 | 0.104 | 0.245 | 0.214 | 0.128 | 0.082 | 0.377 |
| meta2479 |  | 1048.615 | 163.123 | 0.103 | 0.125 | 0.111 | 0.003 | 0.010 | 0.133 | 0.182 | 0.320 | 0.020 | 0.440 | 0.097 | 0.057 | 0.244 | 0.381 | 0.074 |
| meta2480 |  | 1048.616 | 184.553 | 0.521 | 0.561 | 0.557 | 0.027 | 0.113 | 1.060 | 1.389 | 0.511 | 0.067 | 1.034 | 0.497 | 0.101 | 0.936 | 1.238 | 0.199 |
| meta2481 |  | 1048.824 | 342.674 | 0.018 | 0.014 | 0.015 | 0.011 | 0.011 | 0.011 | 0.019 | 0.017 | 0.019 | 0.020 | 0.014 | 0.014 | 0.022 | 0.010 | 0.015 |
| meta2482 |  | 1049.074 | 342.595 | 0.023 | 0.018 | 0.020 | 0.012 | 0.016 | 0.015 | 0.022 | 0.023 | 0.022 | 0.024 | 0.020 | 0.014 | 0.027 | 0.015 | 0.021 |
| meta2483 |  | 1049.118 | 184.212 | 0.020 | 0.017 | 0.013 | 0.000 | 0.001 | 0.037 | 0.048 | 0.013 | 0.001 | 0.026 | 0.009 | 0.002 | 0.029 | 0.035 | 0.006 |
| meta2484 |  | 1049.384 | 493.565 | 0.005 | 0.004 | 0.005 | 0.006 | 0.004 | 0.005 | 0.005 | 0.006 | 0.006 | 0.006 | 0.005 | 0.005 | 0.006 | 0.004 | 0.006 |
| meta2485 |  | 1050.148 | 465.415 | 0.014 | 0.014 | 0.015 | 0.015 | 0.022 | 0.012 | 0.019 | 0.013 | 0.015 | 0.011 | 0.018 | 0.019 | 0.023 | 0.017 | 0.020 |
| meta2486 |  | 1051.398 | 361.896 | 0.174 | 0.143 | 0.123 | 0.126 | 0.196 | 0.065 | 0.274 | 0.143 | 0.057 | 0.177 | 0.241 | 0.145 | 0.079 | 0.048 | 0.254 |
| meta2487 |  | 1052.325 | 478.741 | 0.004 | 0.004 | 0.002 | 0.007 | 0.003 | 0.003 | 0.005 | 0.003 | 0.003 | 0.005 | 0.004 | 0.003 | 0.003 | 0.004 | 0.004 |
| meta2488 |  | 1052.636 | 47.401 | 0.007 | 0.009 | 0.007 | 0.026 | 0.013 | 0.008 | 0.003 | 0.001 | 0.004 | 0.001 | 0.001 | 0.003 | 0.001 | 0.001 | 0.001 |
| meta2489 |  | 1052.650 | 152.115 | 0.005 | 0.006 | 0.006 | 0.002 | 0.001 | 0.000 | 0.002 | 0.007 | 0.002 | 0.001 | 0.006 | 0.007 | 0.002 | 0.001 | 0.002 |
| meta2490 |  | 1052.757 | 318.447 | 0.017 | 0.018 | 0.020 | 0.016 | 0.017 | 0.019 | 0.018 | 0.016 | 0.024 | 0.016 | 0.019 | 0.017 | 0.020 | 0.024 | 0.017 |
| meta2491 | Taurocholic acid | 1053.570 | 184.553 | 0.133 | 0.138 | 0.127 | 0.012 | 0.040 | 0.151 | 0.199 | 0.124 | 0.028 | 0.191 | 0.131 | 0.043 | 0.155 | 0.171 | 0.058 |
| meta2492 |  | 1053.570 | 162.537 | 0.042 | 0.048 | 0.044 | 0.001 | 0.004 | 0.042 | 0.067 | 0.087 | 0.011 | 0.107 | 0.037 | 0.026 | 0.059 | 0.087 | 0.032 |
| meta2493 |  | 1054.341 | 494.123 | 0.018 | 0.016 | 0.015 | 0.018 | 0.017 | 0.016 | 0.018 | 0.021 | 0.017 | 0.026 | 0.023 | 0.018 | 0.021 | 0.011 | 0.023 |
| meta2494 |  | 1058.716 | 171.514 | 0.018 | 0.020 | 0.012 | 0.032 | 0.049 | 0.011 | 0.067 | 0.036 | 0.013 | 0.017 | 0.053 | 0.037 | 0.018 | 0.005 | 0.099 |
| meta2495 |  | 1059.540 | 403.222 | 0.005 | 0.005 | 0.005 | 0.005 | 0.004 | 0.004 | 0.004 | 0.004 | 0.004 | 0.006 | 0.006 | 0.005 | 0.004 | 0.003 | 0.005 |
| meta2496 |  | 1059.627 | 163.805 | 0.006 | 0.006 | 0.003 | 0.001 | 0.003 | 0.009 | 0.018 | 0.014 | 0.002 | 0.010 | 0.006 | 0.007 | 0.007 | 0.008 | 0.005 |
| meta2497 |  | 1060.507 | 290.029 | 0.005 | 0.005 | 0.007 | 0.005 | 0.005 | 0.005 | 0.006 | 0.005 | 0.007 | 0.005 | 0.004 | 0.005 | 0.006 | 0.007 | 0.006 |
| meta2498 |  | 1063.626 | 163.769 | 0.002 | 0.003 | 0.003 | 0.000 | 0.000 | 0.002 | 0.008 | 0.005 | 0.000 | 0.005 | 0.003 | 0.002 | 0.004 | 0.007 | 0.001 |
| meta2499 |  | 1063.670 | 169.376 | 0.004 | 0.008 | 0.006 | 0.007 | 0.010 | 0.008 | 0.027 | 0.019 | 0.011 | 0.005 | 0.008 | 0.019 | 0.013 | 0.010 | 0.021 |
| meta2500 |  | 1065.642 | 163.073 | 0.047 | 0.053 | 0.050 | 0.002 | 0.005 | 0.066 | 0.084 | 0.148 | 0.008 | 0.221 | 0.049 | 0.026 | 0.114 | 0.151 | 0.037 |
| meta2501 |  | 1065.684 | 172.086 | 0.009 | 0.014 | 0.014 | 0.019 | 0.023 | 0.013 | 0.033 | 0.028 | 0.013 | 0.015 | 0.025 | 0.030 | 0.015 | 0.008 | 0.042 |
| meta2502 |  | 1066.143 | 479.434 | 0.004 | 0.004 | 0.005 | 0.004 | 0.006 | 0.004 | 0.008 | 0.005 | 0.004 | 0.006 | 0.005 | 0.006 | 0.008 | 0.005 | 0.007 |
| meta2503 |  | 1067.701 | 171.467 | 0.037 | 0.065 | 0.052 | 0.058 | 0.073 | 0.036 | 0.113 | 0.103 | 0.061 | 0.043 | 0.089 | 0.104 | 0.053 | 0.044 | 0.157 |
| meta2504 |  | 1068.100 | 184.008 | 0.004 | 0.004 | 0.005 | 0.000 | 0.002 | 0.006 | 0.009 | 0.003 | 0.002 | 0.008 | 0.006 | 0.001 | 0.009 | 0.009 | 0.003 |
| meta2505 |  | 1068.644 | 46.226 | 0.009 | 0.010 | 0.009 | 0.050 | 0.030 | 0.014 | 0.005 | 0.002 | 0.008 | 0.001 | 0.002 | 0.003 | 0.002 | 0.001 | 0.001 |
| meta2506 |  | 1069.203 | 170.844 | 0.007 | 0.006 | 0.005 | 0.012 | 0.017 | 0.007 | 0.022 | 0.030 | 0.009 | 0.012 | 0.027 | 0.020 | 0.006 | 0.004 | 0.033 |
[truncated: 9,886 more chars]
